# Extensive Gains and Losses of Olfactory Receptor Genes in Mammalian Evolution

**DOI:** 10.1371/journal.pone.0000708

**Published:** 2007-08-08

**Authors:** Yoshihito Niimura, Masatoshi Nei

**Affiliations:** 1 Department of Bioinformatics, Medical Research Institute, Tokyo Medical and Dental University, Tokyo, Japan; 2 Institute of Molecular Evolutionary Genetics and Department of Biology, The Pennsylvania State University, University Park, Pennsylvania, United States of America; Indiana University, United States of America

## Abstract

Odor perception in mammals is mediated by a large multigene family of olfactory receptor (OR) genes. The number of OR genes varies extensively among different species of mammals, and most species have a substantial number of pseudogenes. To gain some insight into the evolutionary dynamics of mammalian OR genes, we identified the entire set of OR genes in platypuses, opossums, cows, dogs, rats, and macaques and studied the evolutionary change of the genes together with those of humans and mice. We found that platypuses and primates have <400 functional OR genes while the other species have 800–1,200 functional OR genes. We then estimated the numbers of gains and losses of OR genes for each branch of the phylogenetic tree of mammals. This analysis showed that (i) gene expansion occurred in the placental lineage each time after it diverged from monotremes and from marsupials and (ii) hundreds of gains and losses of OR genes have occurred in an order-specific manner, making the gene repertoires highly variable among different orders. It appears that the number of OR genes is determined primarily by the functional requirement for each species, but once the number reaches the required level, it fluctuates by random duplication and deletion of genes. This fluctuation seems to have been aided by the stochastic nature of OR gene expression.

## Introduction

Vertebrate olfactory receptors (ORs) are G-protein-coupled receptors (GPCRs) containing seven transmembrane α-helical regions and function as the receptors for various odor molecules in the environment [1]–[3]. It is known that OR genes form the largest multigene family in vertebrates. However, the numbers of OR genes are quite different among different species, and each species has a large number of pseudogenes in addition to functional genes. For example, humans have ∼800 OR genes, but ∼50% of them are pseudogenes [4]–[6]. By contrast mice have ∼1,400 OR genes, and the fraction of pseudogenes is 20–25% [7]–[9]. Therefore, the number of functional genes is ∼2.7 times larger in mice than in humans. OR genes are present as genomic clusters that are scattered on many different chromosomes. Despite the difference in the number of genes between humans and mice, the organization of OR genomic clusters is well conserved between the two species [9].

Previously we studied the evolutionary change of OR genes in vertebrates using zebrafish, pufferfish, frog, chicken, mouse, and human data [10] and showed that the OR gene family is considerably smaller (∼100) [11] but is more diversified in fishes than in mammals. We also showed that particular groups of genes have expanded and others were completely lost in the tetrapod lineage. It therefore appears that the OR gene family is subject to an extreme form of birth-and-death evolution [12], [13].

To understand the evolutionary mechanism of this multigene family, it is important to study the variation of OR genes among mammalian species living in diverse environments, because they have much larger repertoires than non-mammalian species do. Now that the draft genome sequences are available for at least six different orders of mammals including two early-diverged lineages, monotremes (platypus) and marsupials (opossum), we conducted comparative and evolutionary analyses of OR genes from eight mammalian species.

## Results

Table 1 shows the numbers of OR genes identified from platypuses, opossums, cows, dogs, rats, and macaques as well as those from humans and mice. The number of functional OR genes having intact coding sequences is considerably smaller in primates and platypuses than in other species. The numbers in Table 1 are minimum estimates of the numbers of functional OR genes because we used draft genome sequences that were incomplete. It is possible that some functional OR genes were misannotated as pseudogenes because of sequencing errors or nearly identical copies of genes were collapsed into one sequence because of assembly errors. Moreover, genome sequences containing short contigs tend to give an underestimate of the number of functional genes, because a functional OR gene located at the end of a contig is truncated. For this reason, we counted the numbers of truncated genes that could become functional when the genome sequence is completed. We identified large numbers of truncated genes from the cow and platypus genomes (Table 1), reflecting a relatively low quality of the genome sequences of these species. The fraction of pseudogenes in platypuses is estimated to be ∼50% under the assumption that truncated genes are functional, and this fraction is similar to that in humans. By contrast, opossums showed the lowest fraction of pseudogenes (<20%) in the species examined.

**Table 1:** OR genes in eight mammalian species.

Order	Species	Functional genes	Truncated genes	Pseudogenes	Total	Fraction of pseudogenes (%)^a^
Monotremata	Platypus	265	83	370	718	51.5
Marsupialia	Opossum	1,188	10	294	1,492	19.7
Cetartiodactyla	Cow	970	182	977	2,129	45.9
Carnivora	Dog	811	11	278	1,100	25.3
Rodentia	Mouse^b^	1,035	28	328	1,391	23.6
	Rat	1,207	52	508	1,767	28.7
Primates	Macaque	309	17	280	606	46.2
	Human^b^	387	0	415	802	51.7

a Truncated genes were assumed to be functional for this calculation.

b Human and mouse data were taken from references [6] and [9], respectively, with slight modification. See Protocol S1.

The nearly complete OR gene repertoires in dogs, rats, and opossums were also reported in Quignon et al. [14] and Aloni et al. [15]. Although the method of OR gene identification in this study is different from theirs, the numbers of genes identified are generally similar to each other. Quignon et al. [14] identified 1,094 and 1,493 OR genes from dogs and rats, respectively, on the basis of the presence of five amino acid motifs that were extracted from already annotated OR genes. They estimated the fractions of pseudogenes to be 20.3% and 19.5% and for dogs and rats, respectively, which are considerably lower than our estimates. One reason for the discrepancy would be that we used more stringent criteria for the identification of putatively functional OR gene. In our criteria, a functional gene should have initiation and stop codons at proper positions and should not contain any nonsense or frameshift mutations or long deletions (see Protocol S1), while Quignon et al. [14] regarded the sequences other than mutation-containing ones as functional. It is also possible that a considerable number of pseudogenes that are fragmented or do not retain the motifs were not contained in their datasets. We distinguished OR genes from non-OR GPCR genes by constructing phylogenetic trees and did not use the information of motif sequences. However, the motif sequences characteristic to OR genes such as the MA(Y/F)DRYVAIC (single-letter amino acid notation) motif [5], [7] were well conserved among the functional OR genes identified in this study. Aloni et al. [15] identified 1,518 OR genes from opossums, which is similar to our result, but they did not mention the fraction of pseudogenes. We used more recent versions of the genome sequences than the previous studies, and therefore our results are expected to be more accurate.

To investigate the evolutionary change of the number of OR genes in mammals, we estimated the numbers of genes in the ancestral species and the numbers of gene gains and losses for each branch of the evolutionary tree of the eight species using parsimony principle (see Materials and Methods). To estimate these numbers, we classified OR genes into several groups, because the number of genes was very large (>6,000). Mammalian OR genes can be divided into Class I and Class II genes by sequence similarities [6], [9], [16]. A majority of the genes belong to Class II. (Class I genes are 10–20%; see Table 2.) We therefore divided Class II OR genes into subgroups by considering phylogenetic relationships. This generated 34 phylogenetic clades that were supported with high (>90%) bootstrap values (Figure S1) [6], [9]. Note that a considerable number of Class II genes remained unclassified, because the phylogenetic relationships were not completely resolved.

**Table 2:** Number of functional OR genes belonging to each clade.

Clade	Bootstrap value^a^	Platypus	Opossum	Cow	Dog	Mouse	Rat	Macaque	Human
Class I^b^	98.9	**31** (11.7)	**221** (18.6)	**142** (14.6)	**160** (19.7)	**113** (10.9)	**134** (11.1)	**36** (11.7)	**58** (15.0)
Class II									
A	99.0	**35**	**127**	**111**	**114**	**145**	**154**	**45**	**53**
B	96.3	10	19	**56**	**39**	40	55	**19**	**33**
C	95.5	11	34	**50**	17	**48**	43	**20**	**15**
D	88.4	3	30	25	26	14	15	9	**13**
E	94.8	**12**	41	40	**37**	**49**	**69**	6	**13**
F	94.9	1	37	20	23	11	17	**12**	11
G	99.8	1	41	46	24	**76**	**89**	10	10
H	94.4	0	24	**71**	**47**	42	**63**	4	11
I	99.4	0	11	14	15	19	31	8	10
J	93.3	0	23	21	13	18	20	9	10
K	100	0	8	12	10	9	7	5	9
L	99.3	**52**	**63**	2	2	20	25	6	7
M	98.1	0	17	12	8	24	16	3	3
N	99.3	2	15	8	8	15	9	3	6
O	99.4	4	20	8	11	13	9	1	6
P	99.4	0	7	5	8	7	8	5	5
Q	82.2	0	0	15	2	3	3	2	5
R	99.1	0	**47**	15	11	29	20	3	5
S	100	1	21	18	14	12	12	4	5
AA	97.7	**17**	18	9	3	27	26	2	1
AB	100	0	2	8	3	19	24	2	2
AC	99.8	1	23	4	3	17	22	2	1
AD	99.0	3	**59**	4	6	16	16	0	1
AE	99.5	0	2	12	0	15	18	1	3
AF	100	0	4	8	4	13	17	1	1
AG	98.8	1	5	9	4	13	12	3	0
AH	95.7	5	15	7	10	10	10	4	3
AI	90.3	1	12	4	7	8	7	2	3
AJ	100	0	18	6	8	7	9	1	2
AT	97.2	0	3	11	14	5	5	2	1
BA	100	0	0	15	13	4	7	0	0
BB	100	1	5	11	0	2	5	3	2
BC	99.8	0	1	2	0	1	20	2	1
BD	99.1	1	12	0	2	1	0	0	0
Un	–	72	203	169	145	170	210	74	78
Total	–	265	1,188	970	811	1,035	1,207	309	387

Bold characters show the five largest clades for each species. Un, unclassified.

a This value was calculated by taking the average among the bootstrap values in 28 phylogenetic trees constructed using the functional OR genes from all possible combinations of two species out of eight species (see Materials and Methods).

b The percentage of Class I genes is shown in parentheses.

**Figure 1:**
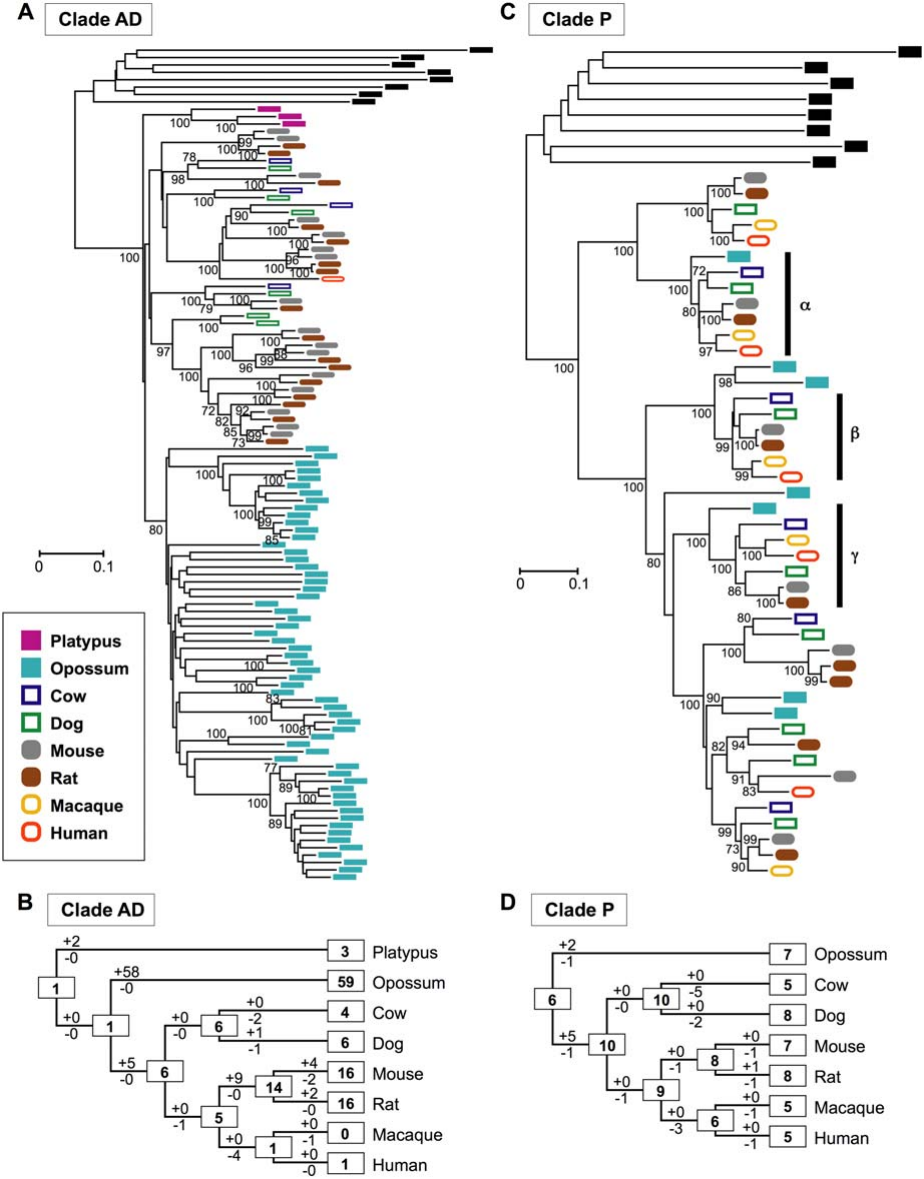
Gains and losses of OR genes during mammalian evolution. (A) NJ tree for 105 Clade AD genes and eight outgroup genes. The outgroup genes used are human Class II genes belonging to Clades A–H and are shown in black. The number of amino acids used is 288. Bootstrap values were obtained by 500 resamplings, and only the values that are >70% are shown. A scale bar indicates the number of amino acid substitutions per site. (B) Evolutionary changes of the number of Clade AD genes inferred from data in (A). The Euarchontoglires tree topology and a 70% bootstrap condensed tree were used for the estimation. The numbers in rectangular boxes are those of Clade AD functional genes for the extant or ancestral species. The numbers with plus and minus signs for a branch indicate gene gains and losses, respectively. (C) NJ tree for 45 Clade P genes and eight outgroup genes. The number of amino acids used is 290. Bootstrap values >70% are shown. Phylogenetic clades indicated by α and γ contain one gene from each of the seven species (opossums and placentals), and a clade shown by β contains one gene from each of the six placental mammals. (D) Evolutionary changes of the number of Clade P genes inferred from data in (C). Platypuses are not shown, because they lack Clade P genes. A 70% bootstrap condensed tree was used.

The results of this classification are shown in Table 2. Note that the number of genes belonging to one clade is often highly variable among different species. For example, platypuses have 52 functional genes belonging to Clade L, which is the largest clade for this species, and opossums have 63 Clade L genes. By contrast, cows and dogs have only two Clade L genes. Similarly, opossums have 59 Clade AD genes, but humans have only one gene and macaques have no gene belonging to this clade. A phylogenetic tree in Figure 1A shows that all of the Clade AD genes in opossums form a monophyletic clade, suggesting that marsupial-specific gene expansion has occurred. This tree also indicates that several gene duplications have occurred in the rodent lineage before the divergence of mice and rats. In accordance with these observations, it was estimated that a large number of gene gains (+58) in the opossum lineage, a moderate number of gene gains (+9) in the rodent lineage before mouse-rat divergence, and some gene losses (-4) in the primate lineage before human-macaque divergence have occurred (Figure 1B).

In contrast, Clade P shows a relatively stable number of genes in the evolutionary process (Figure 1C, D). The numbers of genes in Clade P are similar (5–8) for all the species except platypuses, which lack Clade P genes. Moreover, phylogenetic clades indicated by α and γ in Figure 1C contain one gene from each of the seven species, and clade β contains one gene from each of the six species of placental mammals, suggesting that the occurrence of gene gains and losses in this clade was not frequent (Figure 1D). However, this kind of one-to-one orthologous relationships among different mammalian species are rare for OR genes. We found only four, 14, and 19 phylogenetic clades that contained one gene from each of the eight, seven (opossums and placentals), and six (placentals) species, respectively, and were supported with >90% bootstrap values. It therefore appears that the dynamic change of the number of OR genes was the general rule.

**Figure 2:**
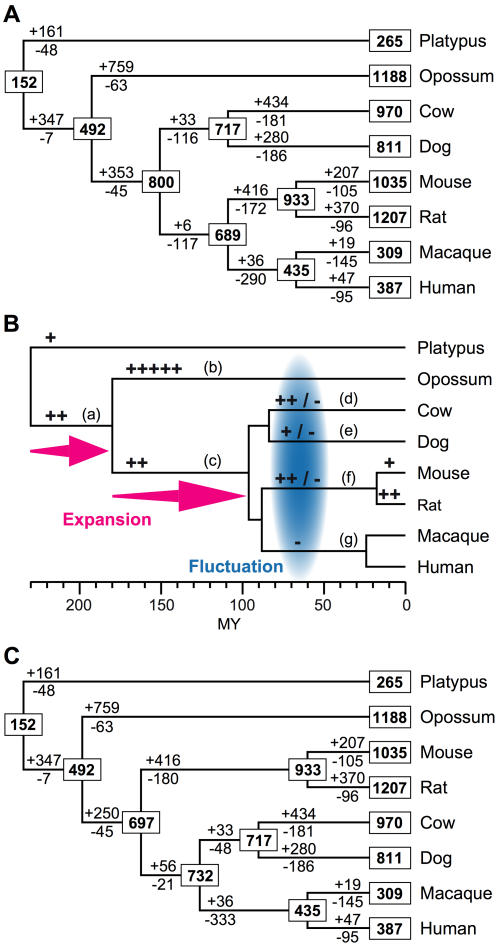
Evolutionary changes of the number of OR genes in mammals. (A) The numbers in rectangular boxes are those of functional OR genes for the extant or ancestral species. The Euarchontoglires tree topology is used. We used a 70% bootstrap condensed tree of OR genes, but the results were essentially the same when 50%, 60%, 80%, and 90% bootstrap condensed trees were used (see Table S1 and Figure S4A). (B) Schematic representation of the results of (A). A plus sign and a minus sign for a branch represent 150 gene gains and losses, respectively. The evolutionary timescale is shown at the bottom in million years (MY). The divergence times were obtained from Murphy et al. [43] except for the time of the human-macaque divergence, which was taken from Glazko and Nei [44]. (C) Results obtained by using the mouse-outside tree and a 70% bootstrap condensed tree. The results for 50%, 60%, 80%, and 90% bootstrap condensed trees were given in Table S2 and Figure S4B.

Figure 2A shows estimates of the evolutionary changes of the number of OR genes when the currently popular mammalian phylogenetic tree is used. These estimates are obtained by considering all clades of genes and unclassified genes. The results suggest that the number of OR genes in the most recent common ancestor (MRCA) for all placental mammals is much larger than that in the MRCA between marsupials and placentals, and the latter is in turn much larger than that in the MRCA for all the species. It was estimated that >300 gene gains have occurred in branches a and c of Figure 2B. Furthermore, hundreds of gene gains and losses occurred in an order-specific manner. Apparently >750 gene gains occurred in the marsupial lineage (branch b in Figure 2B) and >400 gene gains occurred in the cetartiodactyl (branch d) and rodent (branch f) lineages. Moreover, >170 gene losses occurred in each of the branches leading to different placental orders (branches d–g). These findings indicate that although the current numbers of functional OR genes in several mammalian species are similar (∼1,000), their OR gene repertoires have been highly variable. Interestingly, order-specific expansions or contractions of multigene families have been reported for other chemosensory receptors such as vomeronasal receptors [17] and bitter taste receptors [18]. Demuth et al. [19] reported that such lineage-specific expansions or contractions are frequently observed in mammalian gene families. Because our method is expected to give underestimates of the numbers of genes in the ancestral species, it is possible that the estimates will increase when the additional species are used for the analysis. Nevertheless, our estimates for the MRCA between humans and mice (∼690) is fairly close to the number (∼750) obtained by a different method using both functional genes and pseudogenes from the two species [20].

The branching patterns of the four placental mammalian orders examined are not fully resolved. Murphy et al. [21] proposed that primates and rodents are sister groups and they form a clade named Euarchontoglires together with several other orders. This topology shown in Figure 2A (Euarchontoglires tree) has been supported by some other studies as well [22], [23]. However, several authors suggested the topology in Figure 2C (rodent-outside tree) [24], [25]. We therefore conducted the same statistical analysis using the rodent-outside tree. Our data favored the rodent-outside tree, because the total number of gene gains and losses (4,968) is smaller in this tree than in the Euarchontoglires tree (5,134). However, our general conclusion about gene gains and losses was essentially the same for the two trees.

## Discussion

In this study, we showed that the numbers of OR genes have changed extensively in mammalian evolution. Why did the number change so frequently in mammalian evolution? One obvious factor would be the requirement for a species to adapt to a particular environmental condition. For most mammalian species, detection of millions of different odorants is crucial for their survival. Yet, animals living in different environments require different numbers of ORs. For example, olfaction seems to be less important for the primate species that are endowed with trichromatic vision than for other dichromatic mammalian species, because trichromatic color vision is very powerful for perceiving environment signals. This could be the reason why humans or macaques have a smaller number of OR genes than rodents [26]. Platypuses also show a small number of functional genes and a large fraction of pseudogenes. The real reason is unclear, but it may have to do with their semi-aquatic lifestyle [10]. Platypuses have the bill sense, which is a sophisticated combination of electroreception and mechanoreception, and they can find prey with their eyes, ears, and nostrils closed [27]. This situation is similar to that of toothed whales, which have apparently lost the olfactory system and developed the echolocation system to adapt to the full aquatic life. In fact, there are data suggesting that the fraction of OR pseudogenes in toothed whales is extremely high (Go et al., unpublished).

Previously we proposed that the dynamic expansion of OR genes has occurred in the tetrapod lineage during the process of the terrestrial adaptation [10]. This expansion has happened presumably because olfaction is more important in terrestrial life than in aquatic life. Our results (Figure 2B) suggest that the expansion of OR genes continued until the time of mammalian radiation approximately 100 million years ago. As mentioned above, a particular group of genes have often expanded in one lineage (Table 2; Figure 1A). This might have happened because this group of genes is useful specifically for the lineage. For example, Clade AD genes may be able to detect odors that are essential for opossums. At the present time, however, information about the ligands for mammalian OR genes is quite limited. One of the mouse genes belonging to Clade B, which has many genes in humans, is known to detect the smell of lemons (limonene), and one of the mouse genes in Clade G, which is abundant in rodents, perceives floral or woody smell (acetophenone) [28].

Nevertheless, the relationship between the number of OR genes and the environmental factor is not always clear. Dogs, which are supposed to have good sense of smell, do not have the largest number of functional OR genes. It is also difficult to explain why cows have nearly 1,000 functional genes and nearly the same number of pseudogenes. Furthermore, it is known that in rats up to 80 percent of the glomerular layer in the olfactory bulb can be removed without significant effect on olfactory detection and discrimination [29]. Shepherd [29] pointed out the importance of processing of odor distinction in the brain, stating that the expansion of higher brain mechanisms may offset the reduced repertoire of OR genes in humans.

If we consider there factors, it appears that the number of OR genes in a species is not directly related to the environmental requirement or life style, and there are random elements that determine the number of OR genes [30]. These random elements are of course caused by random duplication and random inactivation of genes. In other words, the number of OR genes may fluctuate around the most appropriate number of the genes for a given species, and this fluctuation appears to be quite high if we consider the existence of a large number of pseudogenes in many species.

Figure 2 shows that the evolutionary change of the number of OR genes is exceptionally high. Many multigene families show some evolutionary change of the number of member genes, but the extent of the change is much smaller except for a few other sensory receptor genes [13]. Even with OR genes, the evolutionary change in insects is not as extensive as in mammals. In a group of 12 *Drosophila* species encompassing the divergence times up to about 60 million years the number of OR genes is known to have been quite stable during the evolution [31].

Why then did the number of OR genes change so dramatically in mammals but not in *Drosophila*? One possible explanation is the difference in the mechanism of gene expression system between mammals and *Drosophila*. In *Drosophila*, a specific OR gene tends to be expressed deterministically in a given olfactory neuron [32], [33]. Therefore, if an OR gene is duplicated or lost from the genome, the gene expression system may be disturbed. In mammals, however, one of the clustered OR genes in the genome is stochastically chosen to be expressed in each olfactory neuron [34]. Therefore, the expression pattern of OR genes appears to be considerably different among different individuals, and consequently the number of OR genes may change relatively easily in the evolutionary process [31]. Of course, this is a hypothesis at present, and it should be tested by experiments.

## Materials and Methods

### Data

The draft genome sequences of rhesus macaques (*Macaca mulatta*; rheMac2, released in Jan. 2006; 5.1× coverage), rats (*Rattus norvegicus*; rn4, released in Nov. 2004; 7× coverage), dogs (*Canis familiaris*; canFam2, released in May 2005; 7.6× coverage), and cows (*Bos taurus*; bosTau2, released in Mar. 2005; 6.2× coverage) were downloaded from the UCSC Genome Bioinformatics Site (http://genome.ucsc.edu). The opossum genome sequences (*Monodelphis domestica*; monDom4, released in Jan. 2006; 6.5× coverage) were downloaded from the Ensembl Genome Browser (http://www.ensembl.org). The platypus genome sequences (*Ornithorhynchus anatinus*, released in Dec. 2005; 6× coverage) were retrieved from the website of the Genome Sequencing Center at Washington University School of Medicine (http://genome.wustl.edu). We did not use the sequences in the ‘bin0’ category for the cow genome, because they were not assembled.

### OR Gene Identification

The method to identify functional OR genes from draft genome sequences of platypuses, opossums, dogs, cows, rats, and macaques is essentially the same as that used in our previous studies [6], [9], but we improved it to be applicable to any mammalian species. Details of the method are provided in Protocol S1 and Figure S2. OR pseudogenes and truncated genes were identified in the following way. We first conducted TBLASTN [35] searches against the genome sequences using all functional genes in each species identified in this study as queries with the E-value below 1e-20. We then extracted the non-overlapping blast-hits showing the lowest E-values among the hits to a given genomic region. After excluding functional OR genes identified, we regarded all remaining sequences as pseudogenes or truncated genes. The reason we used the cutoff E-value of 1e-20 is as follows. First, the lowest E-value for non-OR blast-hits was around 1e-17 or 1e-18. Second, we confirmed that all blast-hits showing the E-value below 1e-20 are more similar to OR genes than to known non-OR genes. Therefore, OR pseudogenes and non-OR genes are distinguishable by using the E-value of 1e-20. To identify truncated genes from these sequences, we extracted the sequences that did not have any nonsense or frameshift mutations and were located close (<30 base pairs) to the contig end. We then constructed a multiple alignment of these sequences together with functional OR genes by the program E-INS-i in MAFFT version 5.8 [36]. From the alignment, we extracted truncated sequences that meet the following condition. When the C-terminal portion of an OR gene is missing from the genome sequence, the N-terminal portion should contain an initiation codon at a proper position and should not contain any nonsense mutations, frameshifts, or long gaps. When the N-terminal portion is missing, the C-terminal portion should have a stop codon at a proper position and should not contain any nonsense mutations, frameshifts, or long gaps. Amino acid sequences of all OR genes identified in this study are available in Dataset S1.

### Estimation of the numbers of genes in the ancestral species and those of gene gains and losses

To estimate these numbers, we used the reconciled tree method [37]–[39], in which the topology of a gene tree is reconciled with that of a species tree. A simple example is shown in Protocol S1 and Figure S3. Since phylogenetic relationships of genes are not completely resolved due to low bootstrap values, we considered a condensed tree with a given bootstrap value level as a gene tree [39], [40]. To apply this method to OR genes, we developed a computer program, which is available on request to Y. N.

### Classification of OR genes

In the previous studies [6], [9], we classified human and mouse OR genes into phylogenetic clades that were supported with >90% bootstrap values. We classified functional OR genes identified from six mammalian species into these clades. For this purpose, we constructed phylogenetic trees for all functional genes from each of the six species together with those from humans or mice. Using these trees, the assignment of clades could be conducted without any ambiguity, because all human or mouse genes belonging to one clade were always contained in a larger clade supported with a high bootstrap value. We then constructed phylogenetic trees using all genes in any pairs of species out of the six species. In every tree obtained, genes assigned to the same clade formed a monophyletic clade supported with a high bootstrap value, almost all of which was >90% (Table 2; Figure S1), showing that the classification is robust. We identified four new clades (BA–BD) with >90% bootstrap supports that contained ten or more member genes from at least one species. We did not use Clades AJ–AS in reference [9], because the numbers of genes belonging to these clades are small. We used Class I gene clade and 34 Class II gene clades (A–S, AA–AJ, AT, BA–BD) to apply the reconciled tree method. Several Class II genes remained unclassified and were examined separately. The names of functional OR genes belonging to each clade are provided in Dataset S2.

### Evolutionary changes of the number of OR genes

We first constructed a phylogenetic tree using all genes belonging to each of the 35 clades (Class I gene clade and 34 Class II gene clades) together with eight outgroup genes each of which was chosen from Clades A–H. The reconciled tree method was applied to the 35 phylogenetic trees. We used 50%, 60%, 70%, 80%, and 90% bootstrap condensed trees of OR genes. Unclassified Class II genes were examined in the following way. We constructed a phylogenetic tree using all unclassified genes together with 34 representative genes each of which was randomly chosen from the 34 Class II gene clades. Five Class I genes were also selected randomly and were used as outgroup genes. Because the tree topology slightly changed depending on the genes used, we repeated tree construction for 20 times. Out of the 20 trees, we selected one tree of which the phylogenetic relationships were best resolved in the following way. The total of the numbers of clades with >50%, >70%, >80%, >90%, and >95% bootstrap supports was calculated for each of the 20 trees, and the tree showing the largest value was regarded to be the best tree. Numbers in Figure 2A,C were obtained by summing up the results for the 35 clades of genes and unclassified genes.

### Phylogenetic Tree Construction

Translated amino acid sequences of OR genes were aligned by the program E-INS-i in MAFFT version 5.8 [36]. Poisson correction distances were calculated after all alignment gaps were eliminated. A phylogenetic tree was constructed from these distances using the neighbor-joining (NJ) method [41] by the program LINTREE [42] available at http://www.bio.psu.edu/People/Faculty/Nei/Lab.

## Supporting Information

Protocol S1 Supplementary materials and methods.
(0.04 MB DOC)

Table S1 Estimated numbers of genes in the ancestral species and those of gene gains and losses for the Euarchontoglires tree and various bootstrap condensed trees.
(0.03 MB PDF)

Table S2 Estimated numbers of genes in the ancestral species and those of gene gains and losses for the mouse-outside tree and various bootstrap condensed trees.
(0.03 MB PDF)

Figure S1 (A) A neighbor-joining (NJ) phylogenetic tree for 265 functional OR genes in platypuses and 1,188 genes in opossums. Purple and blue lines represent branches for platypuses and opossums, respectively. Bootstrap values obtained from 500 replications are shown for the branches determining Class I clade and 34 Class II clades. The scale bar indicates the estimated number of amino acid substitutions per site. (B) An NJ tree for 811 functional OR genes in dogs and 387 genes in humans. Green and orange lines represent branches for dogs and humans, respectively.
(0.47 MB PDF)

Figure S2 Flowchart for the identification of functional OR genes and OR pseudogenes. See Materials and Methods and Protocol S1 for details.
(0.30 MB PDF)

Figure S3 Estimation of the numbers of genes in the ancestral species and those of gene gains and losses by the reconciled tree method. See Protocol S1. (A) A species tree. (B) A gene tree. (C) A gene tree for estimating the number of genes α in (A). A diamond represents the divergence between marsupials and placentals. A dashed line indicates a gene loss. (D) A gene tree for estimating the number of genes β in (A). A diamond represents the divergence between rodents and primates. (E) Evolutionary changes of the number of genes inferred from (B). “-1” indicates a gene loss. There are no gene gains in this case.
(0.24 MB PDF)

Figure S4 Names of nodes and branches for (A) Table S1 and (B) Table S2.
(0.22 MB PDF)

Dataset S1 Amino acid sequences of OR genes from six mammalian species. “Oran”, “Modo”, “Bota”, “Cafa”, “Rano”, and “Mamu” represent platypus, opossum, cow, dog, rat, and macaque OR genes, respectively. A gene name with “P” and “T” indicate a pseudogene and a truncated gene, respectively. An asterisk and a slash in an amino acid sequence represent a stop codon and a frameshift mutation, respectively.
(6.30 MB DOC)

Dataset S2 Names of functional OR genes belonging to each clade.
(0.47 MB DOC)
