# Supplementary material for: Extensive Gains and Losses of Olfactory Receptor Genes in Mammalian Evolution
Source: PLoS One. 2007 Aug 8;2(8):e708. doi: 10.1371/journal.pone.0000708 (PMC1933591; doi:10.1371/journal.pone.0000708)
Supplement: Protocol S1 — Supplementary materials and methods. (0.04 MB DOC) [file pone.0000708.s001.doc]

**Protocol S1.** Supplementary Materials and Methods

**Identification of functional OR genes.** We identified functional OR genes from six mammalian species (platypus, opossum, dog, cow, rat, and macaque) in the following way (see Figure S2). We first conducted TBLASTN [1] searches against the genome sequences of these species with the E-value below 1e-10. As queries, we used 920 representative functional OR genes identified from humans, mice, chickens, frogs, zebrafish, and pufferfish [2–4]. Because many queries hit to the same genomic region, we extracted non-overlapping sequences each of which showed the lowest E-value among the hits to a given region. Functional OR genes were identified from these blast-hit sequences using the following five criteria. Each criterion was applied to exclude nonfunctional OR genes or non-OR genes from the blast-hit sequences. We first discarded the blast-hits that were <250 amino acids long (criterion 1). Here we used a conservative cutoff length of 250 amino acids, which is sufficiently shorter than any known functional OR genes, to avoid incorrect exclusion of functional genes. (All functional OR genes identified in this study are actually >270 amino acids long.) Each of the blast-hit sequences was extended to both directions along the genome sequence and the longest coding sequence from the AUG to the stop codon was extracted. If a coding sequence was <250 amino acids long after extension, it was discarded (criterion 2). For the remaining sequences, we constructed a multiple alignment using the program E-INS-i in MAFFT version 5.8 [5] and assigned seven transmembrane (TM) regions according to Man et al. [6]. We excluded sequences in which there was a gap of ≥5 amino acids within a TM region (criterion 3). When a sequence contained two or more methionines in the N-terminal tail (the upstream of the first TM region), the most proper methionine was chosen as the initiation codon in the following way, because the N-terminal tail is 21–34 amino acids long for most of known functional OR genes. (i) When methionines were present between the positions -34 and -21, the most downstream one among the methionines was chosen. (Here the position of a methionine is indicated as the relative position to the boundary between the N-terminal tail and the first TM region.) (ii) When methionines were present only at the position -35 or its upstream, the most downstream methionine was chosen. (iii) When methionines were present only at the position -20 or its downstream, the most upstream methionine was chosen. Next we constructed a phylogenetic tree for the remaining sequences together with several non-OR GPCR genes as outgroups using the neighbor-joining (NJ) method [7] by the program LINTREE [8]. We used the following genes as the outgroups, because they are relatively close to OR genes among GPCR genes in the rhodopsin family [9]: alpha-1A-adrenergic receptor isoform 1 (GenBank protein id, NP_000671), beta-1-adrenergic receptor (NP_000675), adenosine A2b receptor (NP_000667), histamine receptor H2 (NP_071640), 5-hydroxytryptamine (serotonin) receptor 1B (NP_000854), 5-hydroxytryptamine (serotonin) receptor 1F (NP_000857), 5-hydroxytryptamine (serotonin) receptor 6 (NP_000862), galanin receptor 1 (NP_001471), somatostatin receptor 4 (NP_001043). In the phylogenetic tree, the OR gene clade was always supported with a high bootstrap value. Therefore, non-OR genes were easily distinguishable from OR genes and were excluded (criterion 4). Finally, we constructed a multiple alignment by E-INS-i and excluded the sequences having gaps in the TM or other highly conserved regions by visual inspection (criterion 5). The remaining sequences were regarded as functional OR genes.

We applied the above criteria to OR genes previously identified from humans and mice [2,3]. We found that two functional genes (HsOR11.18.22, HsOR17.1.13) and one pseudogene (HsOR11.3.49P) for humans [2] should be regarded as pseudogenes and a functional gene, respectively, under the new criteria. Similarly, two functional genes from mice (MmOR7.5.26, MmOR7.5.31) [3] were regarded as pseudogenes in this study.

**The reconciled tree method.** As a simple example, let us consider the evolutionary change of the number of genes in five species, opossums, mice, rats, macaques, and humans (Figure S3A). Suppose that the tree in Figure S3B represents a gene tree for a given gene family. In this example, mice, opossums, and humans have one gene belonging to this gene family, which are named A, B, and C, respectively, while rats and macaques do not have any member genes of this family. The minimum number of genes in the MRCA between marsupials and placentals (α in Figure S3A) is obtained in the following way. Since gene B is from marsupials and gene C is from placentals, the separation of genes B and C is assumed to correspond to the divergence between marsupials and placentals. Figure S3B shows that the separation between gene A and the clade containing genes B and C has occurred before the marsupial-placental divergence. Therefore, gene A has originated from another ancestral gene in the MRCA between marsupials and placentals, and the orthologous gene A’ has been lost in the marsupial lineage (Figure S3C). In this way, α is estimated to be two, and one gene loss is assumed to have occurred in the marsupial lineage (see Figure S3E). Next, let us consider the number of genes in the MRCA between rodents and primates (β in Figure S3A). In this case, we have to assume that the rodent gene (C’’ in Figure S3D) orthologous to the primate gene C has been lost, because the separation between genes B and C is more ancient than the divergence between rodents and primates. It is also assumed that the rodent gene A has originated from another ancestral gene in the rodent-primate MRCA, and the primate gene A’’ orthologous to the gene A has been lost (Figure S3D), since the separation of gene A from the clade containing genes B and C is more ancient than the rodent-primate divergence. Therefore, it is estimated that β is equal to two, and that one gene loss has occurred in each of the rodent and primate lineages (Figure S3E). By repeating this kind of analysis for all interior nodes, we eventually obtain the result shown in Figure S3E.

1. Altschul SF, Madden TL, Schaffer AA, Zhang J, Zhang Z, et al. (1997) Gapped BLAST and PSI-BLAST: a new generation of protein database search programs. Nucleic Acids Res 25: 3389–3402.

2. Niimura Y, Nei M (2003) Evolution of olfactory receptor genes in the human genome. Proc Natl Acad Sci U S A 100: 12235–12240.

3. Niimura Y, Nei M (2005) Comparative evolutionary analysis of olfactory receptor gene clusters between humans and mice. Gene 346: 13–21.

4. Niimura Y, Nei M (2005) Evolutionary dynamics of olfactory receptor genes in fishes and tetrapods. Proc Natl Acad Sci U S A 102: 6039–6044.

5. Katoh K, Kuma K, Toh H, Miyata T (2005) MAFFT version 5: improvement in accuracy of multiple sequence alignment. Nucleic Acids Res 33: 511–518.

6. Man O, Gilad Y, Lancet D (2004) Prediction of the odorant binding site of olfactory receptor proteins by human-mouse comparisons. Protein Sci 13: 240–254.

7. Saitou N, Nei M (1987) The neighbor-joining method: a new method for reconstructing phylogenetic trees. Mol Biol Evol 4: 406–425.

8. Takezaki N, Rzhetsky A, Nei M (1995) Phylogenetic test of molecular clock and linearized trees. Mol Biol Evol 12: 823–833.

9. Fredriksson R, Lagerström MC, Lundin LG, Schiöth HB (2003) The G-protein-coupled receptors in the human genome form five main families. Phylogenetic analysis, paralogon groups, and fingerprints. Mol Pharmacol 63: 1256–1272.
