# Supplementary material for: Extensive Gains and Losses of Olfactory Receptor Genes in Mammalian Evolution
Source: PLoS One. 2007 Aug 8;2(8):e708. doi: 10.1371/journal.pone.0000708 (PMC1933591; doi:10.1371/journal.pone.0000708)
Supplement: Table S1 — Estimated numbers of genes in the ancestral species and those of gene gains and losses for the Euarchontoglires tree and various bootstrap condensed trees. (0.03 MB PDF) [file pone.0000708.s002.pdf]

Table S1. Estimated numbers of genes in the ancestral species and those of gene gains and losses for the Euarchontoglires tree and various bootstrap condensed trees

Node/branch	Bootstrap value (%)				
	50	60	70	80	90
A	185	167	152	132	118
a1	+153 / -73	+158 / -60	+161 / -48	+165 / -32	+169 / -22
a2	+388 / -12	+362 / -9	+347 / -7	+340 / -6	+319 / -4
B	561	520	492	466	433
b1	+737 / -110	+751 / -83	+759 / -63	+775 / -53	+797 / -42
b2	+474 / -64	+417 / -53	+353 / -45	+300 / -34	+241 / -25
C	971	884	800	732	649
c1	+37 / -208	+32 / -160	+33 / -116	+31 / -92	+25 / -55
c2	+6 / -189	+6 / -154	+6 / -117	+6 / -89	+8 / -69
D	788	736	689	649	588
d1	+463 / -251	+443 / -210	+416 / -172	+392 / -140	+367 / -97
d2	+40 / -384	+37 / -334	+36 / -290	+33 / -257	+29 / -202
E	800	756	717	671	619
e1	+412 / -242	+423 / -209	+434 / -181	+446 / -147	+470 / -119
e2	+262 / -251	+270 / -215	+280 / -186	+296 / -156	+320 / -128
F	1,000	969	933	901	858
f1	+174 / -139	+187 / -121	+207 / -105	+226 / -92	+249 / -72
f2	+327 / -120	+347 / -109	+370 / -96	+394 / -88	+429 / -80
G	444	439	435	425	415
g1	+14 / -149	+17 / -147	+19 / -145	+22 / -138	+26 / -132
g2	+42 / -99	+44 / -96	+47 / -95	+54 / -92	+62 / -90

For the names of nodes and branches, see Figure S4A. The estimated numbers of gene gains and losses for each branch are shown with plus and minus signs, respectively.
