# Supplementary material for: Extensive Gains and Losses of Olfactory Receptor Genes in Mammalian Evolution
Source: PLoS One. 2007 Aug 8;2(8):e708. doi: 10.1371/journal.pone.0000708 (PMC1933591; doi:10.1371/journal.pone.0000708)
Supplement: Table S2 — Estimated numbers of genes in the ancestral species and those of gene gains and losses for the mouse-outside tree and various bootstrap condensed trees. (0.03 MB PDF) [file pone.0000708.s003.pdf]

Table S2. Estimated numbers of genes in the ancestral species and those of gene gains and losses for the mouse-outside tree and various bootstrap condensed trees

Node/branch	Bootstrap value (%)				
	50	60	70	80	90
B	561	520	492	466	433
b1	+737 / -110	+751 / -83	+759 / -63	+775 / -53	+797 / -42
b2	+321 / -64	+285 / -53	+250 / -45	+221 / -34	+192 / -25
C	818	752	697	653	600
c1	+463 / -281	+443 / -226	+416 / -180	+392 / -144	+367 / -109
c2	+78 / -45	+68 / -31	+56 / -21	+42 / -17	+35 / -11
D	851	789	732	678	624
d1	+37 / -88	+32 / -65	+33 / -48	+31 / -38	+25 / -30
d2	+40 / -447	+37 / -387	+36 / -333	+33 / -286	+29 / -238

For the names of nodes and branches, see Figure S4B. The numbers identical to those in Table S1 are omitted.
