# Supplementary figures and images for: Extensive Gains and Losses of Olfactory Receptor Genes in Mammalian Evolution
Source: PLoS One. 2007 Aug 8;2(8):e708. doi: 10.1371/journal.pone.0000708 (PMC1933591; doi:10.1371/journal.pone.0000708)

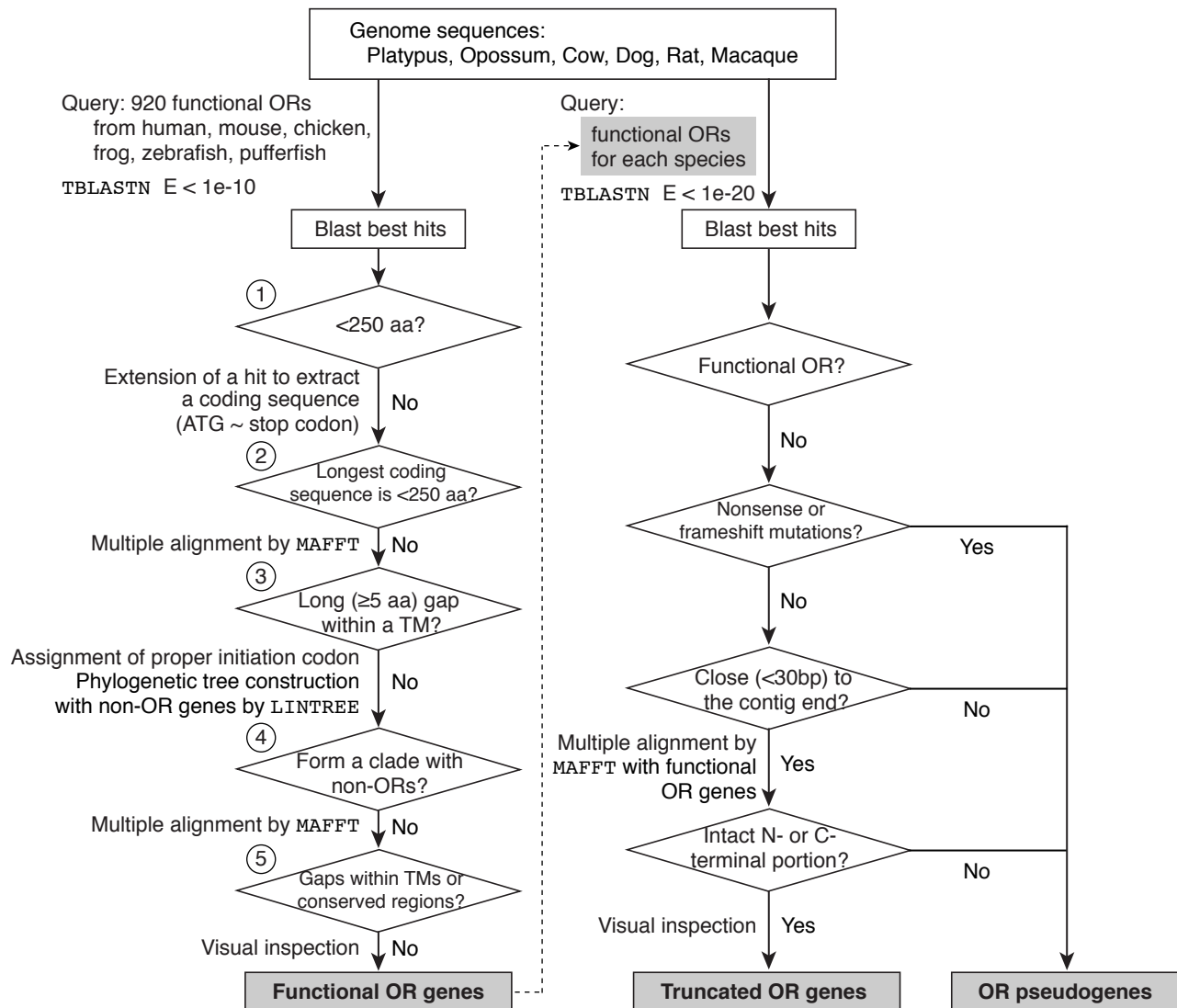

Figure S2

Supplement: Figure S2 — Flowchart for the identification of functional OR genes and OR pseudogenes. See Materials and Methods and Protocol S1 for details. (0.30 MB PDF) [file pone.0000708.s005.pdf]

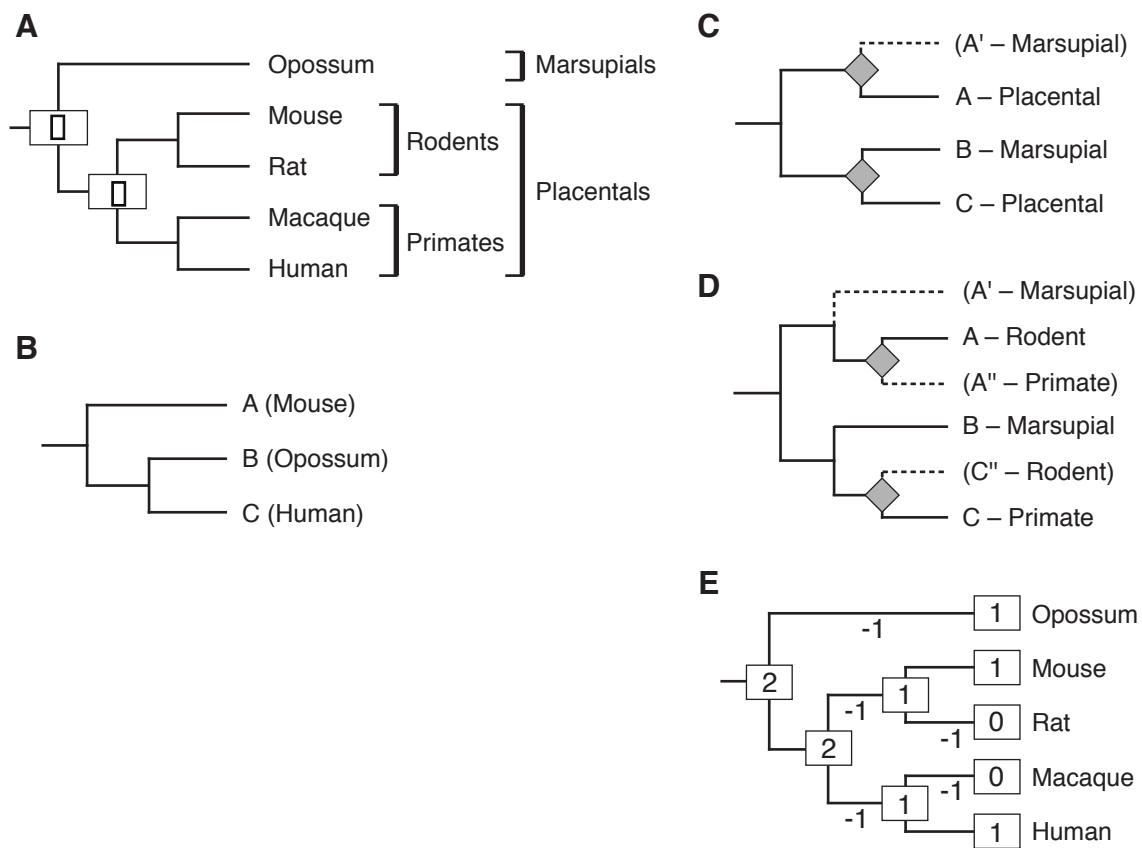

Figure S3

Supplement: Figure S3 — Estimation of the numbers of genes in the ancestral species and those of gene gains and losses by the reconciled tree method. See Protocol S1. (A) A species tree. (B) A gene tree. (C) A gene tree for estimating the number of genes α in (A). A diamond represents the divergence between marsupials and placentals. A dashed line indicates a gene loss. (D) A gene tree for estimating the number of genes β in (A). A diamond represents the divergence between rodents and primates. (E) Evolutionary changes of the number of genes inferred from (B). “-1” indicates a gene loss. There are no gene gains in this case. (0.24 MB PDF) [file pone.0000708.s006.pdf]

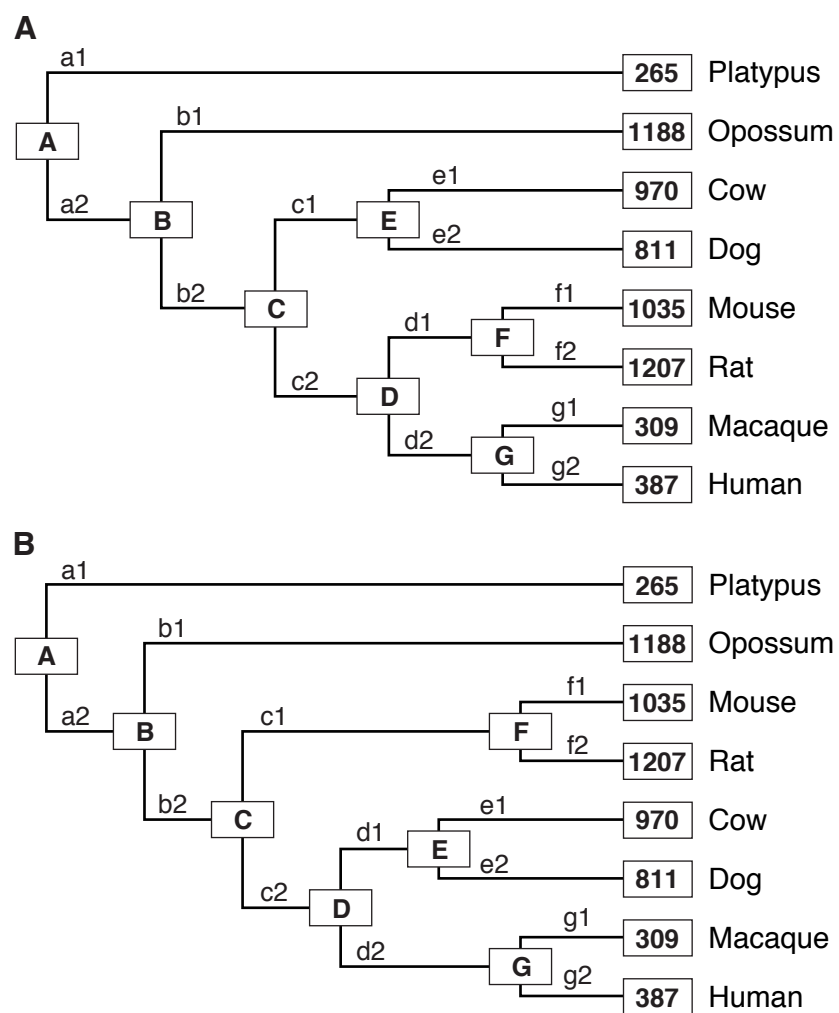

Figure S4

Supplement: Figure S4 — Names of nodes and branches for (A) Table S1 and (B) Table S2. (0.22 MB PDF) [file pone.0000708.s007.pdf]
