# Supplementary material for: Extensive Gains and Losses of Olfactory Receptor Genes in Mammalian Evolution
Source: PLoS One. 2007 Aug 8;2(8):e708. doi: 10.1371/journal.pone.0000708 (PMC1933591; doi:10.1371/journal.pone.0000708)
Supplement: Dataset S1 — Amino acid sequences of OR genes from six mammalian species. “Oran”, “Modo”, “Bota”, “Cafa”, “Rano”, and “Mamu” represent platypus, opossum, cow, dog, rat, and macaque OR genes, respectively. A gene name with “P” and “T” indicate a pseudogene and a truncated gene, respectively. An asterisk and a slash in an amino acid sequence represent a stop codon and a frameshift mutation, respectively. (6.30 MB DOC) [file pone.0000708.s008.doc]

>OranORc14.1P

VKEFSGTFIAGNLHSIIHVGFLFRLTFCRSNIISHFYCDALPLYRLSCTD

PYINVLMIFIFSGSIQVFTITTVLVSYHRILFTILTFKSKEGRGRAFSIC

ASHFLSVSLLYGSILFMYVWPGSIKEENKDILVAVFYTIIIPLLNPFIYS

IRNKELISVFRKIMKRR

>OranORc14.2P

MAEGNHTLKMEFVLSGFTDKSRLQLFLFVVLLVTYMITIVGNLGLVMLIW

VEPRLHTPMYFF/FFLGNLGLTDACCSCSIIPKVLMNIVTGNKAISLSEC

MVLFYFLCFAETADCFLLAAMAYDRYIVIFNPLLYPVMMSKKLCIHIHSI

VFI

>OranORc14.3P

MAERNNTSVAMFILSGLIEHPELQVLLSVLFSIVYLITMLGNFGLIMLIW

TDSQLHTPMYFFLGNLALVDACCSSDVTPNMIANFLFEKKKFLFLG/KKI

PFFGCVGQLGLFSACATTQCFLLASMAYDRYIAICNPMLYTVKMSQKLCI

RLIIGAYVGG/LHSSLHVGFLFRLSFCGPNVIHHFFCDIIPLFKVSCTDP

TLNELLVIIFSGSIQVFTILTIIISYSYILCTILRMKSVKGKHRTFSTCG

AHLVSVTLFFGSLLFMYVWPGSTEVEDQDMMDSLFYTIIIPLLNPFIYSL

RNKEVIGILRKVIRRQ

>OranORc588.1

MEKRNQTQVSEFILVGFSGQPDQQRLLFGLFLAVYSVSVLGNLLIILAVR

SDPRLHTPMYFFLVHLSFVDICLTSTTVPRMLENLWAGGKSIPFAGCLAQ

MYFFLSFVNTDGFLLAAMAYDRFLAICRPLRYAAAMGPRLCTLLVAGPWL

VTHSNALVQTLMMARLSFCVGTVIPHFFCEFAPLLQLACSDTHFNERLAL

AAAVLLGFTPAGCILASYARIVSVVLRVPSARGKRRAFSTCGSHLVVVTL

FYGTGIGVYLCPAAAYPSGNGPMASLVYAVVTPTLNPFLYSLRNGDMKGA

LRKVICGRFSHH*

>OranORc588.2

MEGVNRSRVDEFIFLGLTENPILEAAFFATFSVTYVLTLLGNLLIVVTVA

STPRLHTPMYFFLGNLSLIDVCHSSVTVPKMLEGFLSENKSISFEGCIAQ

LFFLHLFACSEIFLLTVMAYDRYVAICVPLHYPAVMTVEVGVQLVSALWL

GGVVHSLVQTLLTIRLPYCGPNVIDNYFCDVPQVIRLACTDTYLTWMLIV

SNSGAISLFCFLALSASYSVILASLRKRSAEGRRKALSTCTVHFIMVVFL

FGPCIFLYTRPHTTYSADKAVSVFYTVITPWLNPLIYTLRNEEVKKSMRQ

LRKKQVFVLRCF*

>OranORc685.1P

GFLVVCRPAHQDQQAPFYVEPKVINHYCCENTMLVQLACSDTHVI*MMEL

VLDVVTLILTLALVILS*AYIVWTI*RI/SVEQRRKAYSTCSCHLIVVAL

SHAHRYLSFCHSSAKGGTKMAAAPRPSQRGGGLALERTAAPALGP

>OranORc1021.1P

LSGLCPVSVTVPKSILSTLTGRGSISDSGCAAQVFFTLSFLTWEPFALPA

LSHDRYAAVCRPPNRAVTASGRARVRISGSVLGAVPAASASSLSCSSTAV

LPATSPRRRSCRAPNRPSPKKRPWPPGRRGCRPLYLHRCRLRSHLLRGPP

RVFSTCPPHLVAFVASGISAYLKPPSDSPSVPDPSASPSYVAVPLAPNPL

TSSPRNGAMRAAMGKVIAGR

>OranORc1714.1P

ISFPGCATQFFFLSLFGTTQAFLLATMAYDHFIFI*DPLHYSMIMSHGIC

HLLVGGLYLWGVANALTQTTMIF**MFCGPHLIYGFFCHLLAILTPSSSD

ILINQLFL

>OranORc1777.1P

NVSAVRGFLLLGFSEVRELRLVHAALFLLVYLAALTGNLLVVAVTVLDRR

LRAPMYFFLGHLSVLDLCLVSVTVPKSVTISLTNNSSISFPGCVAQLLLG

TLFAAAEYFVLTAMSYDRYVAICLPLRYGVLMDRGACEKMAAASWLSGGL

IGVLFSSFTFSLSF/FCGSNVIQQFFCDVSSLLKIACSEEHVAIDVSVAV

AVALGILCFVSIVVSYARIFRAVLRMPAAEGRARAFSTCLPHLTIVIVVI

LTAVTAYLKLPSDSSSTPDLLVSVFYIVVPPALNPLVYSLRNRDVKAAVG

RVI

>OranORc1777.2

MPNVSTVREFLLLRFSEVRELRLVQATLFLPVFLAALTGNLLVAAVTVLD

RRLRTPMYFFLGNLSLTDLCHVSVTVPKSVVISLTDRRSIRLLGCVAQVF

LVVVSATSEPVILTWMSDDRYAANCRPLSYELITTDTARGKMAVASWLGG

GLFGGLFSAWTFSLSFCGSNAVQRFFRDVPSLLKITCSEEHVAIDVSVAA

GVALGVVCFILIVVSYARIFGAVLRMPAAEGRAGAFSTCLPHLVVVTLFV

VTCFCAYLKPPSDSPSTPDLLVSVFYTVVPPALNPLIYSLRNRDVKAALG

SVLKGRFLLPPLRDRMSVSFS*

>OranORc1777.3P

MTNISAVREFLLLGFSEVRELQLVQAALFLLFYLAALTGNLLIVAVTLLD

RRLRTPMYFFLRNLSVLDFCLVTVTVPNSIHNSLTDRRSISLVGCTVQVF

SVAWFAGSELFVLTAMSYDRYAAICLPLRYEVVMEPRGLWEAGGRLLASA

GVCLQQCIQLGRSPCPSAGSNTVPQIFCDIPP

>OranORc1777.4

MTNISAVREFLLLGFSEVRELQLVQAALFLLFYLAALTGNLLIVAVTLLD

RRLRTPMYFFLRNLSVLDFCLVTVTVPNSIHNSLTDRRSISLVGCTVQVF

SVAWFAGSELFVLTAMSYDRYAAICLPLRYEVVMDRGACGKLAAASWLGG

GLFAAMYSAGTFSLSFCGSNTVPQFFCDIPPLLKITCSEKHIAIDATLIF

EAALGVVCFASIAVSYVRIFGAVLRMPAAEGRGRAFSTCLPHLVVVTVFF

SAGIFVYFKPPSDSSLIVDLLVSVFYTVVPPALNPLIYSLRNRDMKAAMG

RILKRSLTQPLLWYRMSPSLCR*

>OranORc1777.5

MPNVSTVRGFLLLGFSEVRELQPVQAALFLPVYPAAPTGNLLVVAVTVTV

LDRRLRAPMYFFLGNLSLIDLCLVSVTFPKSFYNSLTDLREISFLGCATQ

VCLVIFFAGAEYFLLTAMSYDRYAAIRLPLRYGVVMNRATCGKMAAASWL

GGGLFGVLFSASTFSLSFRGSNVVQQFFRDVPSLLKITCSEEHVAIDVSV

AAGVALGVVCFILIVVSYARIFRAVPRMPAAEGRAGAFSTCLPHLTVVTL

FVLTSFCAYLEPPSDSPSVLNPLMSLFYTVSPPALNPLIYSLRNRDVKAA

LGRGLSREWPPSCGREVATLCLLELTWPQ*

>OranORc1777.6

MSDDTANRPAARGFLLLGFSEVRGLRPVQAALFLPVFLAALTGNLLIVAV

TVLDRRLHTPLYFLLGNLALVDLRYVSVTVPKSIPDSLSNGNSVSPPACA

AQVFLVIFLIGSEMALPTVTSHDRLVAVCRPLRYDTVVHRGACASVLAAS

WLSSGLNAALYATGVFSLSFCAPNVVHQFLCDGPQSLRLACSSGQGAQKL

SLATTVGLPFFCSVLVVVSYARIFRAVLRMPVAEGRGKAFSTCPPHLAVV

TLFVTSGSSAYLKPISDSPSALELKLSVFYSVLPPTLNPLIYSLRNRDVK

MAMEKLLGQKPPPRGGLSFR*

>OranORc1777.7P

GGSMPTMLKLACVDVRANEIQLFVGTLVLILLPLSLITVSYGSIARAVLR

IKSPRVWRKALGTCGSHLLVVTLFYGSITAVYVWPGSSFSGTWDKFLTLF

YTVVTPTLNPLIYTLRNKDMKGAVRRILGKGQS

>OranORc1777.8P

KEMANRTAVTGFLLLGFSEVREVRLVQAALFLLVYLAALTGNLLVVAVTA

LDRRLRAPMYFFLGHLSVLDLCYISVTVPQSVHNSLTDNRSISFLGCVFQ

LFFLLLFAFSELYFLTAMSYDRYAAICRPLRYGVIMDRGACGKMAAASWL

GGTVSAATHAAATFSAGFGGSDVIRHFFCDTPQMMELSGPGGNLQEIGVT

VFSAILNVSCLVSIVVSYARIFGAVLRMPAAEGRARAFSTCLPHLAVVIL

FISTGTVAHLKPPSASSSTPDLLVSVFYTVVPPALNPLIYSLRNRDVKAA

LGRXXXXEG

>OranORc1777.9

MANCTAVREFLLLGFSEVREPQPVQAAPFLPVYPAAPTGNLLVVAVTALD

RRLRAPVYFFLGHLSVLDLCLVSVTVPKSIHNSLTDRREISVPGCISQVF

LVIALAGVEVAPLTAMSYDRYAAICLPLRYGAVVDRGACGKMAAASWLGG

GLNGGIHTAVTFSLLFCDSSAIRQFFCDVPSLIRLSSSRRHGEEMAVLGL

SVFLCLGCFASIVVSYARIFGAVLRMPAAEGRARAFSTCLPHLVVVILFL

STASFAHLKPPSASPSTSDPPVSAFCAVVPPALNPLIYSLRNRDVKAALG

RVLRWKFFT*

>OranORc1777.10P

MANLTAATGFLLLGSSEVREVRPVQAAPFLPVYPAAPTGNLLVVAVTVLD

RRLRAPVYFFLGHLSVLDLCLVSATVPQSVHNSLADRREISPLGCAFQVF

AFRLCACAETPLLTAMSFDRYAAICLPLRYGVITGRGACEKMAAASWLFG

ALSG/HTIATFSVPVWRSNVLPQFFCDIPQLIRLAGPEKMTNTTKVREFL

LLGSSEVRELRLAQAALFLP

>OranORc1777.11

MTNTTKVREFLLLGSSEVRELRLAQAALFLPVYLAALTGNLLVVAVTLLD

RRLRAPMYFFLGHLSVLDLCLVSVTVPQSVHNSLVNGRAISLRGCVAQVF

SVISIAASELSILTAMSHDRYAAVCRPLRYGAIVDRGACGKMAAASWLGG

GLFGAMYTAGTVTLSFCGSNLIQQFLCDVPSSLKISCSETHVVIAVSEAT

AFSLAFVAFVFIVVSYARIFGAVLRMPAAEGRAGAFSTCLPRLAGVTLFV

ANAAFVYLKPLSDPPSAQDLLVSVFYAAVPPALNPVIYSLKNRDMKTTLR

RMIAGKCL*

>OranORc1777.12P

SDHMANLTAMREFLLLGFSEVRELQPVQAALFLPVYPAAPTGNLLVVAVT

ALDRSLRAPMYFFLGNLSLVDLCYVSVTVPKSIHNSLMDNRSIYFRSCVA

QVFLLFPFGGSELFLLTVTSYDRYAAICLPLRYGAIVDRGACGKMASASW

LGGGLFGTMSSAGTFALSFCESNVIPRFF*DVPSLLKISRSERHVVVDDS

VAAGIGFGFVSSVSIVVSSVRTFSAVLRFPSTEGRAGAFSTCLPRLAVVT

VFLSTGAFAYLKPVSDSPSAPDLPVSAFYAVVPPALNPLIYGLRNRDIQA

ALGRLLGVRGWY

>OranORc1777.13P

NVSTVRGFLLLGSSEGREPRPVQAAPFLPVYPAAPTGNLLVVAVTALDRR

LRAPVYFFLGHLSVADLCYISVTVPKSILVSLTQGHSISSLGCGTQVCLV

ILFASSEYFVLTATSYDRYAAIRLPLRYEVVTDREACGKTAAASWLGGGA

DG/GGPMGVAFVASTFSLSFCDTKVIPQFFCATPALLKISSSEAHVAIDV

SVAVAVCLASLCSALIVVS*ARIFGAVLRMPAAEGRAGAFSTCLPRLAVV

AVFFRTAAIAHLKPVSDSPSTLDPPVSVFYVVVPPALNPLIYGLRNRDVK

AALG

>OranORc1777.14T

PFCASNVIRHFFCDIPQLLTLSCSPSVVPELVLIGVSVTLDFGCFLFMGV

SYFHIFSTVLRIPSAQNRTRAFSTCLPHLAVITIFLSTGFFSHLKPTSDG

PTAPDLPVSAFYTVVPPALNPLIYSLRNRDIKAALRKFLRGKL

>OranORc2061.1

MKGEMWRENETRMTGFILVGLFPEFQHRGFLVSVVLLVYLAAFTANAVLI

LLIWVDSRLHVPMYFLLSQLSLLDLALTSTTTPKIASDFFSGKRAISRVA

CGVQMFLYLTVGIAECLVLTLMSFDRYVAVCHPLRYPVLVSPGVCLLMVS

GSWIGGTLSSLVLTVYAATLPVCGSREIRQYFCELPSVVKLACKDTSAFE

MAVSVSGIAFLLIPVSLIVASYVLIFLSVLRVDSREGRRKALTTCSSHLT

VVTLYFGPGIFIYMTPGPSHSPDRNQGVSLFGTLLTPVLNPLIYSLRNRE

VSGALTKVLGKCVVLRWSGKSFLQ*

>OranORc2061.2P

RGEMWRQNETQGTDFTLLGLFPEFQHRGFLVSVVLLVYLAAFTANAVLIL

LIWVDSRLHVPMYFLLSQLSLLDLALTSTTTPKIASDFFSGKRAISRVAC

GVQMFLYLTVGIAECLVLTLMSFDRYVAVCHPLRYPVLVSPGVCLQMVSG

SRIGGTLSSLVLTVYAATLPVCGSREIRQYFCELPSVVKLAWQGHLGVRD

GGVLFRASPSLLI

>OranORc2061.3T

HLTVVTLYFGPGIFIYMTPGPSHSPDRNRGVSLFGTLLTPVLNPLIYSLR

NREVLGALTKIMKQSLVLQSSGKNFL

>OranORc2061.4P

RGGMAEGNETSSTDFILLGLFPEFRHRAFLISVNLLIFAVAVTGNAVLIL

PIWRDRRLHAPMYFLLSQLSLMDLALLNTTEPKMASDFFSGRRNISAFAC

GAQMFCYLTLGSSECLLLTLMSYDRFVAVCHPLRYPVPVSPGLCLGTVAG

TWVGCAFFSLSNVLYTMSIPICGARAIHRYSCELPTVLRLACRDASTYDT

VVLAFGFALILIPFSLITAS*ALIFLTVLRMESRVGRRKALTTCSSHLTV

VTLYYGPIFFIYMTPGISHSPGQNQACSIFGTLITPALNPLIYSLRNKEV

LRALAEVLGTC

>OranORc2061.5P

MGRRNESSTADFILLGLFPECRHRNLLIYIALLIYFVAIAGNAVLVLLIW

VDFRLHVPMYFLLSQLSLVDAILISTAVPKMVADFFSGRRNISRVACGAQ

MFLYLTLGGAERLLLTLTSYDRYVAVCHPLRYPVLVSPVSCVKMVVVTWS

GCVLGSLVHTTYTMNIPVRGAGEIHQYFCELLIVLKLARRDTSTYDRVVI

ASGLTLLLTPFSLIVASYALIFLSVLRMASREGRKKAPATRSSHLAVVAL

YFGPAVFIYVRPDSFRSPEQTSSLRV

>OranORc2061.6

MERGNETSTTDLILLGFSPEISPPSFLISLTLLIYIVAVTGNTVLIRLIW

ADLRLHTPMYFLLGQLSLIDLALISTTVPKMAADFFSGRRSISLPACGTQ

IFFFLTLAGAECLLLTLMSFDRSVAICHPLRYPVLLNRGVRHQMVTGCWV

GGAVNALIHTLYITRLPICNSREIHHFFCEVMTYLKLSCEETSSYQLGVL

GASVVFILFPFGLIVASYALIFFTVLRMDSREGRRKALTTCSSHLTVVTL

YFGPGIFVYVTPPSSHSPERDQGISLFSTILTPMLNPLIYSLRNQEVLGA

LRRVLGKCPSSE*

>OranORc2061.7

MGRSNATSAVTDFILLGLFAEFGYPGFLISIALLTYVVALVGNAVLILLI

LADFRLHTPMYFLLSQLSLMDATLISTTDPKMATNFSLRTRAISGVGCGA

QIFFSLTVGGAECLLLTLMSYDRYVAVCHPLRYLVLVSHQVCLRMILGSW

IGGIANALVLTVSTMQVPICQPRVLHHFFCEIPAILKLSCENTSAYQLTV

FVLGIVFMLTPFSLIVASYAFIFLSILRMDSREGRKKALATCSSHLTVVT

LYFGPTMFIYMRPDSFRRPEQNQALSVFPTILTPALNPLNYSLRNKEVLG

AMTKVFLRRSESG*

>OranORc2061.8

MERGNETSMDEFILLGLFPEFRHRTFLISVILLIYIVALAGNTVLILLIW

VDLGLHTPMYFLLSQLSLMDLAYISTTVPKMTADFFSGKRNISLIGCGAQ

IFFFFTLGGTECLLLTLMSYDRYVAVCHPLRYPVLMNHGVCLQMAAGTWL

GCVVNSLAHTIYTMIVPSCGPREIHHFFCEISAVLKLSCGNTSAYELAVF

AMGVVFLLVPFVLILVSYALVFLSVLRMDSREGRKKALATCSSHLTVVAL

YFAPNIFIYMTPGNAHSPEQDQAVSVFCTILTPLLNPLIYSLRNKEVLGA

LRRVLTRGLFFQRNGKTKGLDVLNSCEWLR*

>OranORc2061.9P

VPTLINSYPTLVXXXXRMDSREGRRKALATCSSHLTVVALYFGSAIFIYV

TPGLSQAPEQNQALSVFNTILTPTLNPLIYSLRNQEVWGALKKFLGTCLI

STWNPNSGCQTVLDARRLA

>OranORc2061.10

MERGNETSNSYLILLGLFPEIRQLGLLISLVLLIYTVAVVGNVVLVLLIW

AEPRLHVPMYILLSQLSLMDAILITTVVPKMAADFFSGRRNVSCVACGAQ

MFLYLTLGGSECLLLTVMSYDRYVAVCHPLRYPVLVSPRICLRMVGGSWT

GGAVNALVQTAYSMHFPICRSREIHHFFCEVMAVLTLSCRDTSAYEAGVT

ATAVGFFLIPVVLITTSYALIFLSVLRMDSREGRRKALATCSSHLTVVAL

YFGSAIFIYVTPGLSQAPEQNQALSVFNTILTPTLNPLIYSLRNQEVWGA

LKKFLGTCLISTWNPNSGCQTVLDARRLA*

>OranORc2575.1P

FLLLGISELVPATLFLLIYLVALTGNLLIITFTAVYHHLHTPTYFFLRHL

SILNFCYISIPVPKCIHSILTQSNFISF/SLFGMCNPTFPSYSFGSTELF

LLTMTS*DHYAAICSPLCYDVIVN*RACGKMAATFWLNKGLSGLSCTSAT

FSMPFCEPQGIHQFFCKIPHLLKLSYSRANLVEMGIAALTTLLDFVVFFY

IVSSYVHFFAAVLRMPATEGRSRAFSTSLSHLTVVSVFLSTEAFAYLKPT

SCSPIVMHLLVSLFYIVVTPP*TP/PTLNPLIYSLRNRDLKMVMG

>OranORc2575.2P

YIYTVVPTKTANFLFDRKFMSFFGCGVQSLFFLTTAGAEGLLLASMMYDL

YVAISSPLTYPVLMRQRRRGLMAVGSWLSGSINSIDHTTYALHTPYCSST

SATSSPCCSQPAWISGSITIWCL*APHHLL*LCLLYTVGRDCICLLWHCT

LPYAENCALHRVSSQ*TGLTE*PTNCHVFLTIYLMSSPMRRMKAFAMSSS

HLPVVIFYYTPFIYTY*GPGPSAFPGEDRKLAV

>OranORc3642.1

MGIPNQTWGAEFILLGLSRDWRTQASLFVLFLVTYMLTLMGNFLILVLIW

LDSRLHSPMYFFLGHLSLIDICYASSVIPQLLAHLLVVKKAIPYLRCAAQ

LYFSLALGGIEFLLLAVMAFDRYAAVCRPLRYPAIVTRGLCAWLAAACWG

GGSLNSLLQTAITFRLPVGHDDRIDHISCELLAVVRLAGVDTSANEAAIV

ASSVVLLMTPCVLVLLSYIRIAVAVVKIRSPEGRQKAFNTCAAHLTVVAL

CYGMAIFLYSKPRSGHGPSALREKLISLFYALLTPVLNPVIYSLRNEEMK

AAGWKLLGRCRELKSKMTLP*

>OranORc3642.2P

GPLNLLLQMAIIFWFFVGRSHSTDHITCELLAVVWFCHLSANEAAIVASS

IILLVMSCGLILLSYVWITTTVLKIRSPKG/PRARQKAFNSCATHLTVVT

LCYMMAIFLYGQSHSR*GPSVMSEKHISLFYVFMLPVLNPMTYSLRNKEL

>OranORc3888.1P

MANLTTLTGFLLMGFSEVRELQLVHAVLFLLVYLAALTGTLLIITTTALD

QCLHTPMYFFLRNLSFTDLCLISVTVSPS/KSIVTSLTDLHSIYYWGCVT

QVLCVIFFMGSEMCVLMVMSYDRYAAICRSLCYDIIMNRGACGKMVATSW

LSGGLFGVMYSAGTFTLSFCKSNTIQQFFCDIPSLLKISCSKTHLVIDVS

VAIGIGFGSFSIVAIITSYFRVFSILLMLPSTEDQAKAFSTCLPHLAVMN

VFVFTAGFAYL*PPSDSPSTLGLLVSVFYTVVPPNPEP/PPTLNPLIYSL

RNRDMKAAPRRI

>OranORc3903.1P

LSRANGSSLTDFILVGFTDQGTLQVLLFGGVLLLYLLNLMGNTAIILVAR

LDPRLHTPMYFFLCHLSFIDAALSTSIIPQLLWNLGGPEKTISYRGCVIQ

LYVALMLCSTECILLAAMAYDRYAAVCHPLHYTALMHPGLCWWLVGLAWL

CGLTNSLVLSTIALVLPRCGHQRVDHFICEVPALIKLACVDTRRSEATVF

AFGVLILVMPASLILASYYAITRAVLKIKSGTGRRKAFGTCGSHLTVASL

FFGTIIYMYMLPHTSSSQEQGKFLTLLYTTVTPVLNPRSTH*RTRTSRG

>OranORc3905.1

MAAGNHSLVTEFILVGLAERPELRLPLFLLFLGVFVATAMGNLGLITLIG

LSPPLHTPMYFFLTNLSFVDLCYSSVTTPKMLAGFVVGTNSVSYPACMAQ

LYFFLVFVIAEGFMLTAMAYDRYVAICHPLLYGTVMSPRACSLLVAGVYT

AGSLCAAVHTACMLRLSFCGENVINHYFCDIRPLLVLSCSRTDINELVVF

ASGGFNMVATSVGILGSYARILSCVLRVGSGRGRKKALGTCGSHLMAVVL

FFGSGAFTYLKPSSGESPGEGKVSSLFYTVVVPMLNPLIYSLRNKNFQDA

LRKILRRNISSGLDLH*

>OranORc3905.2

MLFLGGAEEENLTVATEFLLLGFSDLPELWLFLFLTFLLLYLAILAGNIL

LLSLLLADGALHSPMYFFLGNLSILDLCFTAVTVPNTLANLLTGNGAISA

TACAVQVYFLLAFGSAECYLLVAMAFDRFVAICSPLRYAAVMGRRTCVGL

TVGSWFVGAPVFLGQTLWVFGLPFCGRREVGQTFCDVPPLLQLACTDTAG

HELGTAVMAACFSALPFLLILGSYTRVLGALLRMPSAVGRQKAFSTCSAH

LLVVTLFYASCGAMYLRPKSTFSLRNDRLLALPYTFGTPLLNPLIYSLRN

RDVKGALRRALGRLPSSERV*

>OranORc4104.1P

MANLTTLTGFLLMEFSEVRELQLVHAVLFLLVYLAALTGTLLIITTTALD

QCLHTPMYFFLRNLSFIDLCLISVTVSPS/KSIVTSLTDLHSIYYWGCVT

QVLGVIFFMGSEMCVLMVMSYDRYAAICRSLCYDIIMNRGACGKMVATSW

LSGGLFGVMYSAGTFTLSFCKSNTIQQFFCDVPSLLKISCSKTHLVIDVS

VAIGIGFGSFSIVAIITSYFRIFSILLMLPSTEGQAKAFSTCLPHLAVMN

VFVFTAGFAYLKPPSDSPSTLGLLVSVFYTVVPP/PSTLNPLIYSLRNRD

MKAAPRRI

>OranORc4104.2P

MANGTEVTEFLLLGFLEVQELQLVHATLFLLIYLAALTGNLFIVAVTTLD

RHLHTPMFFFLRHLSVLDLCLISVTGPKSFFNSVTNNNAISFQGCILQVF

FFVSAVVSEVALLTVMSYDRYAAICHPLLYEIIMNRGVCGKMAATSWLSG

GLSGLMHTATTFSEPFSGPNVIHQFF*DVPQLLKLSGPQGNISEYSLTVL

SASSFAICFSYITVSYIRIFSAVLKMPSAEGRSKAFSTCLPHLIVVTFFI

STGVSEYLIPSSNCPTGLDLLLSVFYSMVHPALNPVIYSLRNQAVKAALR

RMLCLE

>OranORc4241.1

MFLIFYASSAETDGENDTISGFILMGLTDGPRHQIPLSILFLMIYGMTLV

GNLGIITLIGMDSRLQTPMYFFLSHLSFVDLCYSSVVAPKLLEILFTEKK

AISFLGCAAQMWFFGILVATECFLLAAMACDRYVAICSPLVYTIIMSPKT

CIQLATAPYVIGLVNATIHTTATFGLTFCGSRKINHFFCDIAPLLSLSCS

SIQVNEILTLLMASLIGVLSGAVILISYTRIVSAILRIRSVEGRYKVFST

CASHLTSVIIFYGTLFFTYVRPPSQDNADKVASVFYSLVIPMLNPLIYSL

RNKEVNNSLRRMIARRRLSTRY*

>OranORc4619.1P

MVVSIGASNDSNLQVTDFILMGFPGIHSWQHWLSIPLGLLYVLALMVNVM

ILLTIWNKTVMHQPMFYFLAVLALVDMGLSTNIMSRILAILWFNARTISL

PECFFQIYAIHIFLGLESGIFLCMALDRYVAICHPLRYLTVITEGFVLKA

TLFMVVRNGVLVIPVPVLAARRLYYSRNEID/HCLCSNLAVTSLACDLRK

ANSIFQLSVAWILMGSDMGLIILSYALILLAVLKLHSAKAASKALITCSS

HIILILFFYLVIVVLLITHSAKGKVPLILVLLNVLHNVIPPALNPIVYAL

RTHEIKVGFLKLVRLAEVRK

>OranORc4619.2P

PQIAKFILIHSWKHWLFLPMTLL/ALLMILLTIL*EMFLHHPVFYFLAVK

IGLSTTIMAR/WLSVRTISVP*CFIKIYAIHIFMGLESSIFLARQWSGKW

PSVTPSATPL*SQKALSSRPLYSWCQKWSPSCPLPVLTIKDIYRSRNEFD

HFLCSILSLTSLACDDRKANSIFQLSVAGILMSSDLGLISLFYALILRAV

LMLYSAKAASKAIRTCSSHIILILFFYTACIDIPITQSAKGKVPLILMLL

IVLHN

>OranORc4619.3

MGFPGIHSWQHWLSIPTAILYLLALVANTMILLTVWQETVLHQPMFYFLA

FLALVDMGLSSTIMPRILTLLWYNARTISLPECFFQIYSIHVFLGLESGI

FLCMALDRYVAICHPLHYPSVITEGFVLKAAMFMVFRYGLLAIPMPILAA

SHHYCSRSEIDHCLCSNLAVTSLACDGRKANSIFQLAVAWIILGSDMGFI

IVSYALILLAVQKLRSAEATSKALSTCSSHIILILFFYSALVLVVTHSAK

EKVPLILVLFNVRHNVIPPALNPIVYALRIQEIQVGILNLIKPCGLRK*

>OranORc4619.4

MFLSIGASNSSSPQVAEFILMGFPGIHSWQHWLSLPLALLYLSALVANVM

ILLTIWKETVLHQPMFYFLAVLALVDMGLSTTIMPRILAMLWFNARTISL

PECFIQIYAIHTFVGLESGIFLCMAIDRYVAICHPLCYPSIITEGFVLKI

TLFMVFRNGLLGVPVPVLASKRIYCSRNEIDHCLCSNLAVTSLACDDRKA

NSIFQLSVAWILMGSDVGLIILSYALILRAVLKLHSAEAASKALSTCSSH

IILILFFYTVIVVMSITHSAKGKVPLIPVLLNVLHNVIPPALNPIMYALR

TQEINVGILKLIRLTGERNSLWVGHGCKKEHERHSKQGSLRSSQLYIFTV

VIQFSETH*

>OranORc4674.1

MITPNQTTVTEFILQSFTENLRLQSLFFSLFLLLFVMAMAGNVLIIAAIH

VSTRLHLPMYFFLANLAILDIICTSSILPKVLENLISARKTISFGGCMNQ

MFFFTWSLSSELLLFTVMAYDRFLAICQPLHYSTMMSKTACVALAAFVWS

IGGLNSVILTGLVLRLSFCGPNLVPHFFCEIPPVLLLSCTPTYWNDILTI

TADIFLAGLNFLLTMVSYSFIISSVMKIRTREGKKRAFSTCSSHLTVVTL

YYSTVLYTYIRPVLGTSGFLDKLASVLYTVLTPTLNPLIYTLRNKDVKVA

LKKLFPFPTS*

>OranORc4816.1P

MANVSTVTEFVLLEFSDGSRELQLGHATLFLLVYMVTLMGNLLIIAATTL

DRHLHSPMYFFLRNLSFIDVCYISVTVPKSIHNSLTNNSLISLQG*ATQP

FQISFFTASELSVLTVMSYDRYVAICHPLHYEHIMDRAVCTQMIATSWFN

GDIFGVMYMAGTFSMPFCGFPIVQQFFCDAPSLLKASRFKRHVVLDVSFA

IGFIFALISFIAITLSYTRIFCTGGFQPLRSRQRLLHLPAPPCRCHIFFL

HGHLCLPYASFRIPLSVGPAGVRVLHCGAPALNPLIYSLRNRDIKAALGR

VLGG

>OranORc4816.2P

MNNHTSVTGFLLLDFSEVRELQLVHTALFLLVYLAALTGNLLVVTITTLD

RHLHTPMYFFLRNLSILDLCLIPITVPKSIL/HSMSQNRSISFLGCVFQV

LLVILFAASEMFVFKVMPYDRYMAICSPLRYEVFMGRGACVKMVVTSWFS

GGLLGVLFSTGVSSLPFCDSHEVQQLFCDIHSLLKISCSEKHVAEDISIA

MGATSGFFSFGFVVLSYIHIFLAVLRMPSSDARSKAFSTCLSRLVVFTLF

ITTGAFAYLTPPSASPSTLDLLVSVFYTVVPPNLKPLIYSL*NWDMKVTL

ARVLGS

>OranORc4909.1

MAENGTEGTEFLLTGFPGRPELQRVLFWVFLVLYLLTLGGNLGIVGLIQA

DAHLQTPMYFFLSHLSLLDACYTSVVVPQMLAALRSGGVSVTSGRCAAQF

FLFTLCASTECFLLAVMAYDRYVAVCHPLLYVAIVTPRARWALVSGAYGG

AMVNTAIRTGCTFSLSFCKSHRVDFFFCDLPPLLKLSCTETGARELVIYL

LAFSVITTSVTVILVSYLFIVKAILCIRSAGGRAKTFSTCGSHVTAVALF

FGTLAFMYLKGNMGQALEVDKVVSVFYTVVIPVLNPVIYSLRNKEVKEAL

RKILQRTKIAQGP*

>OranORc4909.2

MSAGKNQTSVAWFILLGLTDREELKGTLFGVFLLIYTVTLVGNLGIAALV

YADPRLHTPMYFFLGVLSFLDFSYSTVDTPKLLISFLTTDGSISFGACVT

QMALMTLHATGECLLLSVMGYDRFVAICHPLLYHAVMSRRWCGQLVAATF

AASVANAAVQTGNVFRLPYCGPNVIDHYFCDLPTVLHLACADTAEAEALL

SFFSSLVIFITVSVILVSYSCVLASVYKTRSPEARCKALSTCTSHLAAIS

LFYGTVIFMYVQPSSDGSGDRNKVISVFYTIAIPLLNPLIYSLRNKEVKA

ALRRRLFGKLNL*

>OranORc4942.1P

GFSDF*ELQLVHTTLFLLVYLEALKGIFLTNTTTVLKRHLHYP/TPMYFF

LKKLSFIDVCYIAITLPKYNISSLTRNSSIFFLGCLTQLLLIDLFAVSEL

FVLMAMFYDLYGSIYWPLHY

>OranORc4943.1P

YIYTVVPTKTANFLFDRKFMSFFGCGIQSLFFLTTAGAEGLLLASMMYDL

YVAISSPLTYPVLMRQRRRGLMAVGSWLSGSINSIDHTTYALHTPYCSST

SATSSPCCSQPAWISGSITIWCL*APHHLL*LCLL*TVGRDRICLLWHCT

LPYAENCALHRVSSQ*TGLTE*PTNCHVFLAIYLMSSPMRRMKAFAMSSS

HLPVVIFYYTPFIYTY*GPGPSAFPGEDRKLAV

>OranORc4971.1

MGSEGEANQSVETYFILVGFRVSPELQIFLFLAFLMAYCLVLVGNICMIA

VIQGDPQLHTPMYFFLQNLSFIDLSYTSAIAPKALAGFWEQGKSISFAGC

AAQFFLFTTFIVTEGFILAAMAYDRLVAICSPLLYPSRMSRSLCIWLVAG

SYACGCISSTLQCTMTFSMSFCASRVIEHFYCDSQPLRRITCSNTSVEEA

VSLVLASVIILPTVLVILGSYTSIGSAILKIHSSEGRKKAVSTCGSHLGV

VSLLYGTVAFVYLTPPSSPECRKVAAVCYTLITPMLNPMIYSLRNKDVKE

ALKRILGKKMASL*

>OranORc4971.2

MMRNQTAVTEFILLGLTDDPEFQALLFLFLFTTYLLSIAGNLTIITLTLL

DSRLCTPMYFFLRNFSFLEISFTSVFVPKMLVSIGTGNKTISFDACFAQF

FFAILLGATEFFLLAAMSYDRYVAICRPLHYKTIMSGSVCILLVLCSWVS

GLLTVLVPNIMISQLPFCGPNVINHYCCDYIILLQLACSDTLPIEVIVLV

LAVVTLLVTLALVTMSYTAIVRAILRLPSAQQRKKAFSTCSSHMVVVSIS

YGSCIFMYVNPFPKEDLDYSKRVAMLHTSVVPLLNPFIYTLRNQQVKQAF

RDVVGKVNIFHRK*

>OranORc4971.3P

MRNQTSVTEFILLGLTDNPDWKIVIFLFMLLSYLLSITGNLTILTLTLLD

SHLHTPMYFFLQNFSFLEISYTSVTIPKLLVAISTGDRTISYNACAIQLF

FYILLGAAEFFLLAAMSYDRYVAICRPLHYLTIMSQKVCTLLVLCSWL/M

LEISCTDTQLFEKMNFILAVGTLLVTLALVAVSYTAITRTILRLPSTQQR

KKAFSTCSSHMIVIFLSYGSCIFMYIKPSPKEGLDFNKRVAVLNTSVAPM

LNPFIYTLRNQQVKQAIKDVAKRMIFFKKK

>OranORc4971.4

MGNDTGVTDFILVGLTDDPHLQALLFFVLFLTYALSVTGNLTIVTLTLLG

SHLRTPMYLFLRSFSLLEIAFTSACIPRFLVTIVTGDRTISFSNCFTQLF

FFIFLGVTEFFLLAAMSYDRYVAICRPLHYTTVMSGDFCALLVFCSWLAG

FLVVFPPVIVLLQHDFCSSNIINHFLCDSSPMLQLSCSDTRFLELMAFLL

ALGTLLVTLAPVTVSYTAIILAILRLPSSKQRRKAFSTCSSHMVVVSISY

GSCIFMYVNPSPKSGVSFNKGVAVLNTSVAPMLNPFIYTLRNEQVIQALR

DLVFQVQCFLKKRGRAI*

>OranORc4971.5

MGNSTGVTEFILMGLTDDPHLQALLFFVLFLTYAVSITGNLTIITLTLLD

SHLHTPMYFFLRSFSLLEIAFTSACIPRFLVTIVTGDRTISFSNCFTQLF

FFIFLGVTEFFLLAAMSYDRYVAICRPLHYTTVMSGGFCALLVLCSFFSS

YLIVFPPVMMIAQLDFCASNILNHFICDSSPMMGLSCTDTRFLEFMAFLL

ALGTLLVTLALMTASYTAIVRTILRLPSTQQRRKAFSTCSSHMVVVSITY

GSCIFMYIKPSAKDRVELTKGIAVLNTSVAPMLNPFIYTLRNEQVKQAFR

VLVHRIGFSFRK*

>OranORc4984.1T

MSNISTVTEFLLLGFSEVRELQLVHAALFLLVYLATLMGNLLIVTVTALD

WRLHTPMYFFLSNLALIDLCLISVTVPKFVVNSLTNNRSISFLGCVLQVL

FFISLASTEMILLTVMSHDRYAAICHPLRYKVVMNRWACGKMAVASWLSG

GLSGLMHTAATFSKPFCGSNVIQHFFCDAPHLLGHADSTVILREVEVTTF

TTGLSLLCFISIIVSYVRIFSVVLKMPSVEGRSKAFSTCLPHLIVITLFL

SVGAFAFLNPKSNGPSGMDLFLSMFIPYSFQP

>OranORc4984.2P

CDCFLLLTGSDWIMAEVGIWALITSLALIRFICMIIS*VLRMRSSESRCK

AFSTCLPHLILMTFFLPKGSFDYPK/KPLFDSPSSQDLLVSMLCAILQP/

YSLRNSDMKVAL*KILGREMLT

>OranORc4984.3P

CDCFLLLTGSDWIMAEVGIWALITSLALIRFICMIIS*VLRMRSSESRCK

AFSTCLPHLIVMTFFLPKGSFDYPK/KPLFDSPSSQDLLVSMLCAILQP/

YSLRNSDMKVAL*KILGREMLT

>OranORc4984.4P

LLSPPSTWYLHTPMCFFLRSLSVCNLCFISVTVSKSILSVLSNHQSISFL

GCTSQVFSFVIFGVVGLAFFTAMSYDHCMTICHPLLYDIIMDRWLLRRWQ

LHPGSLVDFTP*CTWPPPSPPTSAVLVSSTSSLVTCDIPQLLKLTFLGEA

RAEVYVLAVSVTLVPACFVFLLVSYVHIFWVGLRMQFTEGWVKAFSTCLS

HFTVMTLFI

>OranORc5009.1P

SVTILKLLVAIPTGDRPISYNACASRSF/CLCQSLFYILLGVTEFFLLAT

MSYDQCVAICRRSHYLTIMSLSVCSLLVLCSCLVAYLIIFPDLILGLQLD

/NHFICDSAPKLEILCTDTQLFEQMNFVFAMGTLLVTLDLVTMSYMAITR

TNLRLSFTQQRKKAFSTCCSHMIVIFLSYCSCMFMHIKPSPKEGLDYNKR

VAVLNNSVAPMLNPY

>OranORc5009.2P

MRNHTQVTEFILLGLSDDPDWLTVILLYMSVTYALSIMGNLTIVTLALLD

YRLHTPMYFFLCCFSFLEICFTSACIPRFLATIITGDRMISYNCCA

>OranORc5009.3P

MRNHTQMTEFILLGLADDQDLQAVILLYMSVSYVLSVTSNLTIVIFTLLH

SCLHTPMYFFLLCFSFLEVCFSPCFLATIATKDRTISSSNCFTQLFFFFL

LGVTKFFLLSTMSYDRYIAICRPLHY/TVMSQKVCTLMVLSSCLNGFLVI

FPPVILGLQLDFCSSVATDHFFCDVSPLLLLSCLHTVFLELMAFILALGT

LLVTLMLVAVSYTAIAHTILRLPSDQQKRKAFSTCSSHMVMVSITYGSCI

FIYSKPSIKDRVDLTKGVAMLYTSVVPMLNPFIYTLWNEQVKQAIWDLMH

RFGFS

>OranORc5084.1P

FSLTVFSDIRGLQLFHAILFLLLYLAALTGTLLTTTITTLDLSPPYPHVL

LPEELVLPQSLLNLSQQSLDLSSTP*ATTLSLCGLCS*EFLEIVFGSSET

CVLR*CPMLLSAALHYEVIIKRGACEKMAATSWFTGILFGAMY*AGTLPL

SFCESNRIQELICDISTFTESLLF*NIHC

>OranORc5226.1P

MEKLTTVTEFILLGFLGGSWLQGTLFLTFLILYGMTVVGNLGMVAIISLD

PQLHTAMYSFLCSLSLLEVCYSSTIAPRALLNFLSERAAISFPGCATQFF

FLSLFGTTEAFLLASMAYDRFIVICDPLHYSMIMSHGICHLLVGGLYLWG

VVNAMTQTTMIF*LLFCGPNEIDGFFCDVLPILTPSCSDTLTNQLVLLGL

GGSIIVGTFSIISISYVLVLSTILRILSAEGRMRAFSTCVSHLVGVGLFS

GSVFFMYAQSGATSNMEQSKVVSIFYTVIIPVLNPIIYSLRNKDVKEALK

RIRKKLSL

>OranORc5226.2P

PLDSPSVMNLLLSMFFTVVPPTLKPLIYSLRNRDLKVTMGKFLKGKFCTR

EKSLHISNLA

>OranORc5226.3P

MSNHTTVTEFLLLGFLEVRELQLVHATLFLLNYLVALMGNLLIITVTALD

RHLHTPMYFFLRNLSILDLIIISATIPKSIINLLTDTKSISSVG

>OranORc5598.1

MVVSIGASNDSNLQVTDFILMGFPGIHSWQHWLSIPLGLLYLLALMANVM

ILLTIWKETVMHQPMFYFLAVLALVDMGLSSTIMPRILAILWFNARTINL

SECFFQIYAIHVFLGLESGIFLCMALDRYVAICHPLRYPTVITEGFVLKA

TLFMVVRNGVLVIPVPVLAARRLYCSRNEIDHCLCSNLAVTSLACDVRKA

NSIFQLSVAWILMGSDMGLIILSYALILLAVLKLHSAKAASKALSTCSSH

IILILFFYSVIVVLPITHSAKGKVSLIPVLLNVLHNVIPPALNPIVYALR

THEIKVGILKLVRLAEVRK*

>OranORc5598.2P

ANDSSHGVTEFILMGFPGIHSWQHWLSIPTAILYLLALVANTMILLTVWQ

ETVLHQPMFYFLAFLALVDMGLSSTIMPRILTLLWYNARTISLPECFFQI

YSIHVFLGLESGIFLCMALDRYVAICHPLHYPSVITEGFVLKAAKFMVFR

YGLLAIPMPILAASHHYCSRSEIDHCLCSNLAVTSLACDGRKANSI/IFQ

HAVAWIILGSDMGLIIVSYALILLAVQKLRSAEATSKALSTCSSHIILIL

FFYSAIIVLVITHSAKEMVPLILVLFNVLHNVIPPALNPIVYALRIQEIK

VGILNLIKPCGLRK

>OranORc5598.3

MFLSIGASNGSSPQVAEFILMGFPGIHSWQHWLSLPLALLYLSALVANVM

ILLTIWKETVLHQPMFYFLAVLALVDMGLSTTIMPRILAMLWFNAHTISL

PECFIQIYAIHIFVGLESGIFLCMAIDRYVAICHPLRYPSIITEGFVLKI

TLFMVFRNGLLVVPVPVLASDRIYCSRNEIDHCLCSNLGVTSLACDDRKA

NSIFQLSVAWILMGSDVGLIILSYALILRAVLKLHSAEAASKALSTCSSH

IILILFFYTVIVVMSITHSTKGKVPLIPVLLNVLHNVIPPALNPIVYALR

TQEINVGILKLIRLTGERN*

>OranORc5778.1

MSRVNHTTMSEFILLGFSHLGGSQRLLFGLFLPIYLVTLEANAVIVTTIV

LDRRLHVPMYFFLATLSCSETLYTLVIIPKALVDLLAQHRAITITACAFQ

MGSFLFLGCSHSFLLAAMGFDRYVAICRPLNYARLMTRGVCMVLVAAASS

CGSAVSLVVTFLIFGPPGPSSDRLQHFFCDIPPVLKATLPPGSPRRGVIL

ALGALVLVIPLSLIAASYAHIVATILRIPSSRGRVKPFSTCASHLIVVTI

HYTCASFIYLRPGAGDSSGRDALVSVTYTAVTPLLNPVIYSLRNRDFKAA

LNRVFERIFCPARL*

>OranORc5826.1P

MQRGNQTGVSEFLLLGLSSWAEQQQLLSVLFLWMYLLGVLGSLFIILAIG

SDPHLHTPMYFFLANLSLVDVCFLSTTVPKILANLQSHSKSISYAGCLAQ

LYFVILFVSLDSFLLT*MAYDRYVAICHPLHYATIMSPRLCTQTIVVSWS

VGSLDALLNILMVVRLSFCAGNEIHHFFCDLHEVLKYSCTDTFFNEVLAY

VLLVIFGVVPLTGLLFSYSHIISTVLKIPSVGGRYKVFSTCGSHLSVVSL

FYGTGLRIYFSPRSNQASRQGSTASVMYAVVTPMLNPFIYSLRNKDMKRA

LRNVFCKK/QKTLLLKPVTCPPGS

>OranORc5826.2P

MERGNQTGISEFLFLGLSDRAEQQQLLFVLFLWMYLLGVLGSLLI/ILAI

VSDPHLHTPMFFFLTNLSIADVCFLSTTVPKMLANIQTHDKSIIYAGCLA

QVYFFILFGGLDMFLLSRMACDRYVAICRPLHYTTIMSPQLCAWVYVVS*

SVDSLDALLHTIMLI*LLFCAENENHHFFCDLNQLLKLSCTDILFNQMLK

YVLFVALGILSLIGLLFSYSHIISTILRIPSTGGRFKTFSTFGSHLSVVA

LFYGIGFGVYFSPTCS/GSVASVMYFVVTPMLNPFIYSLRNKDLKDALRN

IFGKKT

>OranORc5858.1T

MFNITTGTEFLLLGFSEVRELQLVHGMMFLLVYLAALIGNLLIVTVTALD

ERLHTPMYFFLSNPALIDLCLISVTVPKSVVNSLTNNRSISFLGCVLQVL

FFISLASTEMILLTVMSHDRYTAICHPLRYEVVMNRGACGKM

>OranORc5858.2P

SGNLLIIAVTTLNWRLHTPKYFFLRHLSILDLCYISTTVPKSIVNSLTDR

REISFLGCVFQVFVFISLASTKMALLTVMSCNRYVAICLPLRYEFVISRG

ACGKMAASSWFSGSLSGLMQTAVTFSLPFRGSNEIHQFFCDIPQLLKLSG

SDWIMAEMDISALIACLAFICFVPMIISYVCIFSAVLRLRSSEGQNKAFS

ICLPHLVVVAFFFSIGSFAYLKPPSDSPSFQDLLVSVFYTGVPPTLNPLI

YSLRNKDMKAALKK

>OranORc5858.3P

CFVSILIPYVHIFWATLRMRSTEGQAKASSTCLPHLALCI/LYLFMGLFA

HLKLPLSSPSTLDLLVSVFYTVLPLTLNPLIYSLRNKDMK

>OranORc5858.4T

CYFCFGAALYPISRVLMSTFFLGGLKMPATEGRTKAFSTCLPHLIVVFLY

LCTGSFAHLKPPSSSPSQLDLLVSVFYTVLPPTLNPLIYSLRNKDMKATL

GKIFSVHFISRVN

>OranORc5858.5P

MANVSMVTEFLLLGFLEVWELQLVHAALFLLVYLVALTGNLLILAIPTLD

QRLHTPMYFFLRTCLS*ISATSPPPSPNPSHSLTNSRSSPSWGCTTQVLL

>OranORc5866.1

MERGNQTSISEFLFLGLSDRAEQQQLLFVLFLWMYLLGVLGSLLIVLAIG

SDPHLHNPMYFFLTNLSLVDIFLLSTTIPKMLANIQTQDKSISYPGCLAQ

MFFFLLFIALDHFLLTGMAYDRYVAICHPLHYTTIMNPRLCALVIAGSWI

VSSLHALTHTLLVVRLSFCSNHEILHFFCDLYQVLKLSCTDTLINESFMY

LLTVVFGVFPFICLMFSYSHIVSTILRIPSTRGRSKAFSTCGSHLSMVSL

FYGTGLGVYFSPTSNQASRQGTTASVMYTVVAPMLNPFIYSLRNKDMKWA

LRNMFIRKNPIF*

>OranORc5866.2P

GNQTSVSEFLLLGLSDRPEQQLLLFVVFLWMYLLGVLGSLLIVLAIGSDP

HLHTPMYFFLTNLSLADACFLSTTIPKMLVNIQTQSKSISYTGCLTQMYF

FILLGSLDHFLLTGMAFDRYVAICHPLHYTTIMSPPLLAILVSGSWIISG

LYALIHTLLVVRLSFCMDNEILHFFCELNQILKLSCTDTVINDVLLYVLI

VMLGVGPIAGLIFSYTHIVSTILKISSARGRSKAFSTCGSHLAVVSLFYG

SALGVYLSPMSIQATRQGSIASVVYTVVTPMLNPFIYSLRNKDMKWALRN

VFCRKSLFFQ

>OranORc5866.3

MKRANQTSVSEFLLLGLSDRAEQQQLLFVMFLCMYLLGVLGSLLIVLAID

SDPHLHTPTYFFLTNLSLADVCFLSTTVPKMLANIQTHSKSISYGGCLAQ

INFFMLFSGIDHFLLIWMAFDRYVAICHPLHYTIIMNPRLCAWMVAGSWI

VSAFNSLTHTSLLIQLSFYGDNKILHFFCEINQVLKLSCTDTLINVALLF

TLLVVLGVVPLTGLLFSYTRIISTILTIPSAVGRSKAFSTCGSHLSVVSL

FYGTGFGVYFSPTSTRASRQLSIASVMYTVVTPMLNPFIYSLRNKDMKWA

LRKLIRRKTLFSQGL*

>OranORc5866.4P

VEKGNQTNVSEFLLLGLFDWVEQWQLFFGLFLWMYLLGVLGNLLIILAIG

SDLHLHTPMYFFLTNLSLVYAYYL/VFFFLLFVGLDHFLLIGMAYDRYVA

ICYPLHYTTIMNPRLCTLMVSGSWSVSNLAALIHTLLVVQLSFCTDNEIL

HFFSELNRVLKLSCTDTLINDVVVYVLTVILAIFPFVGIIFSYARIITTI

LRVPSATGRYKAFNTCCSHLSGVSLFYGTAFGVYFSSVFTQASWKGSIAS

VVYTVVTPMLNPFIYSLRNKDMKWALRNVFRGKTL

>OranORc6057.1

MSLPGLQDPMEEANSTAVSEFIFLGLTQMQEMRIVLFLVFLLVYATTLLG

NVLIMVTVTCDPRLHTPMYFLLRNLSVMDLCFSSVSVPKMLVDLLSESMA

ISFGGCMTQMFFSHFFGGGTVFFLSVMAYDRYVAISRPLHYVAFMSPQMC

VGLIMAAWVGGFVHSIVQLGLVIRLPFCGPNILDNFYCDVPQVLRLACTD

ASVLEFLMISNSGLLTLLWFLLLLVSYTVILVMLRSHSGESRKKAISTCT

THLIVVTLMFGPCIYIYGRPFSPLSMDKAISLSFTVITPMLNPMIYTLRN

QEMKSAMKKLMKRHIRS*

>OranORc6057.2

MEEANFTAVSEFIFLGLTQMQEMRVVLFLVFLLVYTTTLLGNGLIVITVT

CELRLHTPMYFLLRNLSVMDLCFSSVSVPKMLVDLLSESMAISFGGCMAQ

MFFFHFFGGGTVFFLSVMAYDRYVAISRPLHYVALMSPQVCVGLIVATWV

SGFVHSIVQLGLVVRLPFCGPNILDNFYCDVPQVLRLACTDTSVLEFLMI

TNSGLLTLLWFLLLLVSYTVILVMLRSHSGEGRKKAISTCTTHLIVVTLM

FGPCIYIYGRPFSPLSMDKAVSLSFSVITPMLNPMIYTLRNQEVKSAMKK

LMGRHVYS*

>OranORc6057.3

MELENGTTTSEFVFLGLTQSRGLQGVLFVVVLLIYTTILLGNLLIMVTVT

CDPRLHSPMYFLLRNLSVMDIFCSSVTLPRMLVDFLSATKTISYTGCMIQ

IFFFHFLGGADLFFLSVMAYDRYVAISRPLHYVAAMNTQVCVGLIVASWV

GGFVHSIVQLALLLPLPFCGPNVLDNFYCDIPQVLRLACTDTSALEILII

SNSGLLVLIWFILLLASYVVILGMLRSHSKEGRRKAASTCTSHIMVVFVQ

FVPCIYIYARPFTPFPMDKAVSICDTVLPPMLNPMIYSLRNREVRSAVSR

MRKRYLLSKEHSESVRGRRKGLTGEGRSGKREDL*

>OranORc6071.1

MSASNLTQSRPAFFILKGIPGLEAMHVWISIPFSSLYCATILGNCTLLFV

IGTERSLHKPMYLLVAMLALTDLGMSTTTIPKVLCIFWFNWTRISFEGCL

TQLFFIHSISALQSAILTTMAFDRYIAICQPLRYTSILSNGRIGLIGLAS

LVRATLFILPMPLLLQKMSFCGHREIPYTYCEHMAVVKMACVDTRVNRMF

GLAIALVVVGLDLSAIGSSYALILRAVLRLSSHQAHHKAVHTCTAHVCVM

LTSYTPCLFSFLTHRFGHGIPPHVHTILGNLYFLFPPMLNPIIYGVKTKE

FWDKVAKYKCWRPAP*

>OranORc6280.1

MMEVTNGSSGGDFILMGFSDQPQLEMVLFVLILISYLLTLLGNTTIVVVF

HLDPHLHTPMYFFLSHLSFVDLCFTTSVIPQLLWNLWGPYKTITVIGCAV

QLSVALSLGSTECVLLTIMAFDRYAAVCRPLHYMTIMHPRLCQGLAAVAW

LSGMGNSAIQSTITLRLPRCGNRRIPNFGCEVPTMIKLACVDIRANEVVL

FIATLILVLLPIALIVVSYGFIARAALRIKSSQAWRKALGTCGSHLLVVT

LFFGMTSIIYIQPNSSFSKNSGKFLTLFYTVITPTLNPLIYTLRNKDMKG

ALRRLLGKDQS*

>OranORc6396.1P

MRNHTQVTEFILLGFTNNPDWLAVILLYMSVTYILSITGNLTIVTLTLLD

YRLHTPMYFFLRCFSFLEICFTSACIPRFLATIVTGDRAISYNCCATQLF

FLILLGVTEFFLLAAMSYDRYVAI*QPLHYTTIMSQKVCTLLVLSSCLTG

FLVSFPSVILGLQLDFCGSVAIDHFFCDVSPLLLLSCSDTVFLELMAFIL

ALGTLLVTLVLVAVSYTAITRTILRLPSAQQKRKAFSTCSSHMVVVSITY

GSCIFMYIKPSTKDRVDLSKGVAVLYTSVVPMLNPFIYTLRNQQVKQAIR

DLAHRVEFSTKG

>OranORc6396.2P

MRNHTQVTEFILLGLTDDPDLQSVILLYMSVSYVLSFTGNLTIVIFTLLA

SRLHTPMYFFLRCFSFLEICFTFACIPRSLATIATGDRIISFSNCFTQLF

FFIFLGATEFFLLVVMSYDRYVAICQPLHYTTIMSQKVCNLLVLSSCLIG

LLVIFPSVILGLQLDFFGSVAIDHFFCDVRSLCSYSPAQTPCSWS*WLSP

WPRGILLVTLVLVAMSYTAINRTILRLPSTQQKRKAFSICSSHVIVVSIT

VHHGSCIFTYIKPSIKDRVDLTKGVAVLNTSLAPMLNPFIYTLRNQQVKQ

VIRDLVHSFGFSSRR

>OranORc6396.3P

MRNYTPVTDFILLGLRDNPNLQTVILLYMSVIFVMSITGKLSIVTLTLLD

SYLCTPMYFFLCCFSSLEIYLTSTCIPRFLATILTGNRTTYYN*CATQLF

FF/YSFFQLGAT*CFLLAAMSSDHYDAICRPLNYMTVMSQRVCTHLGRCS

LLAGYLVTFPPIILFYRCD

>OranORc6396.4P

MRNHTEFIPLAPLDNPHLQVVILLYMSVTYVMSVTGILTIITLTLLDSCL

HKPMYFFLQSFSLLEMCFTSACIPRFLAHYRHRGQGHFLQLLYNTVPLLP

ARGDLIFLLANMSYDRYVAICRLLHYTTVMNQRVCTLLVF*SWLAVFLII

FPPITPTLQLDFCGSVAINHFFYDVSPLLLLSCSDTGPLELKTFSVAMRT

VLVTLALMVMSYTTIAYVILRLPSSQQKKKAFFTCSSHMVMVFITYGSCI

FMYIKPGQTPR/QADLTKGVAVLNTFVAPMLNSFIYTL*NQQVKQAIRDL

VH*GGSTSRK

>OranORc6396.5P

ISPLLLLSCLDTWFLELMALVLAVGALLLTLALMTASYMAIVRAILRLPS

TQQRRKAFSTCSSHIVVVTITNGSCIFMYIKPSAKERLELTKGVTVLYT/

THSAPMLNSFICTLQTQQVKQAFLGVVHWVGFSSRR

>OranORc6396.6P

MRNYSPVTDFILLGLTDDPDLWAVILLYKSVTYVLSVTSNLTIVTPTLMD

SYFCNSM*VFLHCFSFLETCFTSTCIPRFLATITTGDRTISYSCCVT/QL

FVFILLGATKFFLLAAMSYDHYVAIFPPLQYTTVMSQRVCILLVLCWTFL

VIFPLVILGLQLDFCDSVATDHFFCDISTRPLVSC

>OranORc6481.1P

MSMDNRNRNTTQSHPSSFLLLGVPDL*AAHNWLSFPFCSVYLIALMGNCT

ILFMIKTEQSLHQPMFYFLAMLATVDLGLSSATIPKMLGIFWLHLQEISF

GSCLTQMFFIHMFTGMESMMLVAMGYDRYVAICKPLRYCTILTNKSIRVI

LGLAVLRNFSLIIPMVFFLLRLPYCGHRIIPHTYCEHMGIARLACASIKL

DIIYGLCVICLILLDILLIALSYIRILHTIFRLPSQDARLKAINTCISHI

CVILAFFTPALFSFLTHRFGHDKIPGYVHILLANLYVVVPPALNPIIYGV

RTKQIRERVASIFTNKPSF

>OranORc6481.2

MQNVAGMSFCNSSSFAPTLFTLNGIPSLEALHAWLSLPFCSMYVIAMVGN

CGLLYLICREEALHQPMYYFLAMLSSTDLATCSGTVPRMLLLFWFNLREI

NFSICLVQMFFVHTFTGMESGVLMLMALDRYVAICYPLRYSTILTNPIIV

RAGLATFFRGAILVIPFTILTKRLPYCHGHIIPHTYCDHMSVAKVSCGNI

KVNAVYGLMVAILIGGFDIVCISASYTAILKAVVILSSAEAQHKAFSTCT

AHISAIIITYVPAFFSFFTHRFGGHTIPHHVHIIIANLYLVLPPMMMVNP

VVYEVKTKQIREAVVRMLSGKRGNDP*

>OranORc6611.1

MEKENDTMGTDFILLGLFNHTRAHQVLFALVMMTSITSLMGNTAIILLIH

RDLHLHTPMYFLLGQLSLMDVMLVFTTIPQMAGDFWYGRNSISLVGCAIQ

IFIFLTLEGGECFLLAAMAYDRFVAICCLLRYPVLMSPRLCLLLAVVSWL

LGATDGLVQAGITMSYHFCRSREVNHFFCEAPALVRLACDDTMVFESVMY

VCCVLMLLIPFSVILGSYGLILETILRMRSVEAKKKAFTTCSSHLSVVGL

FYGAAIYIYMRPSSYDSTDYDKVVSAFYTILTPVLNPLIYSLKNREVLGA

LKRGLTDCRFRNLRVEREAA*

>OranORc6621.1

MELENGTAVSEFIFLGLTQSPGLQRVLFVVFLLIYAMTLLGNLLIMVTVT

CDPRLHTPMYFLLRNLSILDVCYSSVTLPRMLVDFLSASKTISYSGCLTQ

IFFFHFLGGADLFFLSVMAYDRYVAISQPLHYTAAMNTQVCVGLIVAAWV

GGFLHSIIQLALLLPLPFCGPNVLDNFYCDIPQVLRLACMNTSVLEILII

SNSGLLVLIWFILLLASYTVLLVMLRSHSKEGKRKAASTCTSHLIVVFVH

FIPCIYIYARPFTPFPTDKAISIFDTVIPPMLNPMIYSLRNHEMKSAMKR

LKKRTSMKCGTFCQRRETYPGCSQTPQ*

>OranORc6621.2

MVLFILFLLVYIMTLLGNLLIMVTVTCDPRLHTPMYFLLRSLSVMDVCYS

SVTLPRMLVDFLSATKVISYGDCMIQIFFFHFLGGTQILFLAVMAYDRYV

AISRPLHYVAIMNTQVCVGLIVAAWVGGFVHSIVQLALLLPLPFCGPNVL

DNFYCDVPQVLRLACTDTSVLEILMISNSGMLVLVWFLLLLVSYTIILGM

LRSHSKEGRRKAASTCTSHLIVVFVQFVPCIYIYTRPFTPFPMDKAVSIC

DTVLPPMMNPMIYSLRNREVRSAVSRMRKRYLLSRRT*

>OranORc6621.3P

CLGMMLVTWLEDFVHSIFQLALLVRLPFCSPSILDNYSCDVPQVLKLSCA

NISLLEPLMISNSRLLILLWFIFLLVSYRRSQKVMDSNPGSYHLSAL*PW

ASHLTSLCLSYFICKMGNEIVSPIWKRDCV*PDLLVSTPALSSVWHTV

>OranORc6621.4

MRLFLDLMEVGNGSIVSEFVFLGLTQNRGLQVVLFMVFLFIYSTTLLGNL

LIMVTVTCDPWLHTPMYFLLRNLSVIDIFCSSVTLPRMLVDFLSPTNTIS

FDGCMAQIFFFHFFGGADMFFLGVMAYDRFVAISRPLHYTAVMNTPVCVG

LILAAWVVGFVHSSAQLALLYRLPFCGPNILDNFYCDVPQVLRLACTDTS

VLEILMISNSGLLVLIWFLLLLASYTVILRMLRSHSKEGRRKAASTCTSH

LIVVFVQFVPCIYIYARPFTPFPMDKAVSVCDTVLPTMLNPMIYSLRNRE

VKLAMKRLKKRLLQA*

>OranORc6657.1

MLISNTTNFHPSTFLLLGIPGMEHQHIWISIPFCSMYIMALLGNATILLV

VACDKTLHEPMYFFLCILSLTDLVLCSTTLPKMLAIFWFGAQHISYIGCL

TQMFFIHMVFATESAVLLAMAFDRFVAICRPLHYSSILSPGVIGKIVMAC

VVRGLFFVFPFVILIQRLPFCGHHVIPHTYCEHMGIAKLACANIKINIIY

GLTVALSVTGMDVVLIGFSYGFILHTVLHLPSQDAQLKAFSTCGAHVCVI

LVFYIPAFFSFFTHRFGHWMPPQVHIFVANLYLLVPPVLNPLVYGINTKH

IRQRILTLFLGYK*

>OranORc6657.2P

MPRFIQSSPVTFTLKGIPGLEDLYLWLSLPFSFMFATTLLG/NSTILFLL

VMESALDKPMYPLLAMLLLADLVSTLAMMPEILGLLWFGVQDISLDACLL

QMFIIHGTSVVHSAVLVAMAFDYVAICDPLHYTTALTRSLVCHLGLVVLV

KGMALISSIPLLLRQLTFCQTVIAHTYCDHMAELKMACGHTGPDRIYGLF

VAILVVGLDCLLLGASYALILQAVLRLSSRRARLRALSTCSNHLSVILIA

YGSVLFSALVHRFGHSLPIHIHILLANLYLFIPSLFIPVIFGVRTKEIRV

MVTKHLNY*PG

>OranORc6657.3

MPKFKEYSPMIFILKGIPSLEDLYLWLSLPFSFMFAAILLGNSAILFLLA

MESALDKLMYHLLALLLLADLVSTLAMMPQVLGLLLFGVQDISLDACLLQ

MLFIHGISLVHSAVVVAMVFDRYVAICDPLQYTTVLTGSLVCHLGLVALA

RGVGLVLPIPLLLRQLTFCRTVIAHTYCDHMAMVKMACGHTGPNRIYGLF

MVILVVGLDCLLMGASYALILRALLQLASCGARLRALSTCSAQLSVVLIT

WGPALFSAIMYRFGCSIPVYSHVLLANFYLLIPSLFNPVIFGVRTKEIRD

IVTKHLGHCPGT*

>OranORc6694.1P

MSNISTVMEFLLLGFSGIWKLQLVHAALFLLVSLAALTGNLLIVTITALD

LRLHTPMYFFVSYLALIDLCFICVTIPKSVINSLTNNRSMWLSG

>OranORc6763.1P

LSGASGSSLTDFILVGFTDQGTLQVLLFGVILLLCLLNLMGNKAIILVSS

LDPRLHTPMYFFLCHLSFIDAALSTYIIPQLLWNLGGPEKTISYRGCVIQ

LYVALMLSSTECILLATMAYDHYAAVCHPLHYTVLKHPGLCWWLVGVAWL

CRLTNSLVLSTIALVLPRCGHRQVGHFIYGVPALIKMAFVDTRWSGATVF

AFGVLILMMPASLLLASSCAITRAVLKIKSGTGRRKAFETCGSQLTVASL

FFGTIIYMYILPHTSSSQEQGKFLTLLYTTVTPVLNP/CAQPLTYTLRNK

DIKRALKRLLGQ

>OranORc6924.1P

IPTLLYSSKFVIIGLGTCSGLFCFVLIIVSYSHIFCTILKITTAEGCSKA

LSTCLPHLAVVTVFILSAMFTYLKPTSDSPYIMDFRVSRFNTVVPPAHNP

VVYCLRNMDIKATLGMMLG

>OranORc7292.1

MSPKGNYTRVTEFILVGFPGSVGLRLSLFLLFLLAYLLTLSENLVIITLV

QGHRPLRKPMYFFLSLLSFLEIWYVSVTVPKMLAALLTLRARHISFLACM

AQLYFFLGLACTECLLLAVMAYDRHVAVCHPLRYPAIMGPGLCLRLGAGS

WLGGFAVSLGKTFFVSRLVYCGPDVLNHFFCDVSPLLNLACTDMSLAELV

DFLLALVILLGPLLVSVFSYVSIVAAVLRIPSASGRKKAFSTCASHLAVV

VIFYSASLFIYARPRALDSFDYNKSVSVVYTVLTPLLNPIIYCLRNQEVK

DALKMMRKKATQKMCVAP*

>OranORc7446.1

MGESQGNTSGSLSIILTGFPGLEDSLYWMFILLGALYSVSILGNSLILVI

LKEEQSLHQPMYYFLAMLAISDLGVNCSTLPTVLGTFCFNVREVSFDACM

TQMFFIHSFSLTESGILLAMSFDRYVAICNPLRYSAILTGSSIVKMGLAI

LVRSFILILPLPFLLRRLPFCKVNILHHAYCLHPDLIHLPCGDTTFNSLF

GLFIVLSTFGLDSVLIFLSYVLILRSVLTIVSQSGQSKALNTCISHICAV

LIFYIPMLGVSMIHRYGKRAPPFIHTLMSIVYLFVPPMLNPIIYSIKTKD

IYKKMDKMLSGAFGVGRGVKPLSG*

>OranORc7663.1

MDNCTSVTEFFLLGFSNSRELQLVYATMFLLVYLASLMGNLLIITVITHD

RHLHTPMYFFLKNLSFIDLCYISVTVPKSILNGLTGSGSISLLGCVLQVF

WVTFLACAELLLLTAMSYDRYIAICCPLRYDSIMNRGASVKMVAVSWLGG

SFNGFLHTAASCTLPFCGSNVIHQFFCEIPQLLKLSSSRRNVPELAVTVV

SAVLVLFSFIAIIISYVRIFSAVMKISSVEGQSKAFSTCLPHLLVFTTFL

ITCVFEYLKPTSDSPSILDVLLSVFYTVVPPTLNPLIYSLRNMDMKAAIG

KFMGSCRIR*

>OranORc7663.2

MSNITTVTEFLLLGFSDIRELQLIHTALFLLVYLAALMGNLLIIAVTTLD

RHFHTPMYFFLKNLSILDLGYISVTVPKSILNSLTNVNSISLLGCAAQVF

LVFLFAGSEMALLTVMSHDRYVAICRPLHYEVIMPHGACVRMLAASWFGS

CLNAIMYTTSTFSLSFCGPNIVHQFFCDGPQLLRLACSTDRVTEDVSLAI

TVGLSFFCFVLIVGSYIRIFSAVLRMPSVAGRSKAFSTCLPHLVVVTLFL

MSGSFAYLKPITDSPSAPDLLVSVFYAMVPPTLNPLIYSLRNRDMKAAMG

TLLWPKHLPKEKTPIG*

>OranORc8006.1P

PNSILNALSNLQSISVWLHSQVFSS*YWYCGPGFLHGDVYDNYMTICRPL

LYDIITDR/CGKMAAASWLIVGLYTLVHMAATFSSHFCGPHVIHQFFCDV

PQLLKLMFPREARAEVCILVVNVTFVLRCFVSLLVPYVHIFCEPHVGQTD

YPVSTPALRTVLGT**ALNKYQHYYLLGMLRMQSTEGRAKAFSTCLPHFT

IVTSFIVNGA/AHLKPVSDSPSTLDRMVSMYYTLVPPTLNSLICSLRNGA

MKATLRRMIA

>OranORc8030.1

MSPMEGENQTRISEFVLLGLLGSPEQQQQQQQRLIFWLFLWMYLLGGAGN

LLMILAVSSDPLLHTPMYFFLSNLSFVDLCLITTTVPKMLLNIQTQKKTI

SYAGCLTQMYFFSLLLGMDNMILAVMAFDRNVAICHPLHYTSVMLPSLCG

LLVAVPWVLTNLISLSLTLLTARLSFCGNNEIPHFFCDINGLLKLSCSDT

RSAENLLLILTSVLGMPPLVGILASYSRIVATVLRIPSAKGKWKVFSTCG

SHLTVVALFYGSGLAVFFTPPSSHSKRKDTAASMMYTVVTPMFNPFIYSL

RNKDMKGVVKKWVC*

>OranORc8170.1P

HSPVLLLVQLLLLEIAFTPSCISRFLVTVVTGDRPISFSNCCFFQLFFFI

LLGVTEFFLLAATSYDRYV/VLRPLRPTTVMHWGVCTLLVLSSFLFSYLF

VFPPVVMSSRLDFCNSNILKHFICDSSSVMGLSCTDTRLWLSSCPWGYSS

LPWC**LHPTQPLSVSSAQQRRKAFSTCYSHMVMVSITYGSCIFAYIKLS

ATDRVELIKGVAVLNTSVAPMLNPFIYTLLNEQVKQAFRALVH*IGFSSR

K

>OranORc8256.1P

SRHLHTSMYFFLKHLSILNLCLISVTLPKSILSALSD/VSFLSCISQVLS

FVIFGAANRAFLTVMSYDYYVSTCRPQLYDIIINRRACGKMVTTSWLTSG

LYTLMEMAATFSSHFCCPCVIHQFFCDVPQLLKLMFPGEARPDVCTLVA*

VTLAPGYFISILLSYVHIFLDLLRM*SSEDRTKAFSICLLHLTVMTSFVA

IGTCYPQPISDSLDLLVSVFYPWCP

>OranORc8271.1P

LPICGSREIQQIFRDIPSLLKISCSEEHTTLDLTVAIGFCLTLFYFVSIV

VSYMHFFSTLLRIQSEEGRSKTFSTCLPHLTITSAFFTTGSFPYLAPTSE

SPSALDLLVYVFYTMVPPALNPLIYSLRNRDIKTSLGRVLQG

>OranORc8271.2P

MNNHTSVTGFLLLGFSEVRELQLVHTALFLLVYLVALTGNLLVVTITTLN

RHLHTPMYFFLRNLSILDLCLISITIPKSIL/HSMSQNRSISFLGCVFQV

LLVILFAASEMFVFTVMSYDRYMAICSPLRYEVVMGRGACVKMVVTSWFS

GGLLGVLFSTGVSSLPFCDSHEVQQLFCDIHSLLKISCSEKHVAEDISIA

MEATSGFFSFGFVVLSYIHIFLAVLRMPSSDARSKAFSTCLPRLVVFTLF

ITTGAFAYLTPPSASPSTLDLLVSTFYAVVPPNLKPLIYSL*NWDMKVTL

GRVLGS

>OranORc8314.1

MLTSNGTLLHQSTFILLGIPGLEAGHIWISIPFSLVYLMALLGNFALLII

IKMEPSLHEPMYLFLCMLAGADLVVCTTAVPKLLSLFWFNDGEISFEGCL

TQMFLIHSLSTMESGFFLAMAFDRYVAICYPLRHSAILTHLVVRRMGLAI

FLRGILLLSAHPFLLRWLPYCRTNIIAHTYCEFMALIKLACAETRVIRAY

SLIVAFFTGGLDFLLIIFSYVLILRAVFCLSSKDARLKTLGTCGSHICVI

LMFYTPAFLSFLTHRFGHHIAPHIHIFVANIYLLVPPMVNPIIYGVKTKR

ILEKVRRFLNLLKF*

>OranORc8398.1P

MSNHTTVTEFLLLGFLEVRELQLVHATLFLLDYLVALMGNLLIITVTALD

RHLHTPMYFFLRNLSVLHLIIISATIPKSIINLLTDTKSISSGG

>OranORc8398.2

MANHTFVMEFFLLGFSEVRELQLVHAALFLLVYLAALLGNLLIVAITVLD

RRLHTPMYFFLRHLSILDLCYISVTVPYSIHNSLTDRRSISFLSCAAQLY

FFACFASLEIALLTVMSYDRYVAICHPLRYDIIMDGGACGKMAATSWLSG

ALSGLVHTVNIFLLPFYDSNVIHQYFCDIPQLLTLSCSPSIVPELVLIGV

SIMLDFGCFLFMGVSYFHILSTVLRIPSAQARTKAFSTCLSHLAVIAIFF

SCGFIAHLKMPSDSPSLLDLLVSLFYPVLPPALNPLIYSLRNKDMKAALG

RVLKGKLP*

>OranORc8461.1

MGPANWTAVTEIVLVGIPTTPELGVFLFLLFLSAYMVTVLGNLLIIMLIC

LDYRLHSPMYFFLSHFSFSEILTTTCVVPRMLANFLSETKSVSFHECFTQ

LCFYFLFGSTEFIFFAIMSYDRYVAICQPLRYPTILTRSLCAQLVTIAWF

GSFFLILPSILLRTRLPYCGPNIIDHFFCDSAPFLHLACADVTFIELLDF

IVSLVLLISSLAMTIISYVCVISTILKIPSGKGRKKAFSTCASHFTVVSM

GYGISIFVYVQPTQKRSLQLNKILFVLSSVVTPLLNPFIFSLRNETMKRA

LSESLGRVQSFTKDLRVIPVMNSNPCRSRS*

>OranORc8461.2P

DPQIQLFLFLVFLLIYGIALVGNLLTMLVIQVDPHTST/PHLYTPMYFFL

SNPS/FLDVRFASSTVPRMLENFLSDQKSITFPECITQIVSLFILATVKI

YLLATMAYNCYRASGQLLCYPGSLTI*LCVQLVRGAWLVGDINVVVNALL

VLRLDFCRPNQNLHFSCEFELPPLLQISCSNIFASEMGILSLGVLLGLVS

FLLTLLSYIHVISILFQICSS*GHGKAFPTCSSHLITVLLFCGTVFFQYM

RPSSA/SHPLALDWVVSIQCSILTPMLNSMIYILKNWDMMRGLKKLLRK

>OranORc8764.1

MANVTTMMEFLLLGFSEVREQQRVHAVLFLLLYLAALMGNLLIVAVTTLD

QRLHTPMYFFLRHLSILDLCYISTTVPKFIVNSLTDRREMSFLGCVFQVF

FFISLASTEMALLTVMSFDRCMAICLPLHYEFVISRGACGKMVASSWFSG

SLSGLMQTAVTFSLPFRGSSEIHQFFCDIPQLLKLSASDWIMAEMGISAR

IVCLAFVCFVSMIISYVCIFSAVLRIRSSEGWNKGFSTCLPHLIVVAFFF

STGSFAYLKLPSDSPSFQDLLVSVFYTVVPPALNPLIYSLRNKDMKAALK

KILGREKFT*

>OranORc8817.1P

LFTLILLVFLVSLLGNATVILLIWLDPGLHSPMYFLLSQLFLMDLLNISI

FVHKMAANLLSGNSSSSFVGCGNQLFVFVLLVVVACLLLTTMAFDHCIAI

/CHPRWYLVLMCPKVCGLMVAGSWLASLLNVLIHCVYTLNLPFCAFWEI

>OranORc8830.1

MLEISLEKMANVTTMMEFLLLGFSEVREQQLVHAVLFLLLYLAALMGNLL

IVAVTTLDQRLHTPMYFFLRHLSILDLCYISTTVPKFIVNSLTDRREMSF

LGCVFQVFFFISLASTEMALLTVMSFDRCMAICLPLHYEFVISRGDCGKM

VASSWFSGSLSGLMQTAVTFSLPFRGSSEIHQFFCDIPQLLKLSGSDWIM

AEMGISALIACLAFVCFVSMIISYVCIFSAVLRIRSSEGWNKGFSTCLPH

LIVVAFFFSTGSFAYLKLPSDSPSFQDLLVSVFYTVVPPTLNPLIYSLRN

KDMKAALKKILGREKFT*

>OranORc8830.2P

MFNITTGKEFLLLGFSEVQELLRGPRHDVSPGLPGGPDGESPHCHCP**A

PPHPYVFLPQQLGPHRPLSHLRLICLISVTVPKSVSLTNNRSIPFLGCAL

QVLFFISLASTEMILFTVITHDRYAAICHPLRYEVVMNRGACGKIAATS*

LSGGLSTLIHTVTTFSEPFCGSHVIHHFFCSAPHLLAQAGSTVILREVKV

TIFTASLSFLCFISIIISYICIFSIVLKIPSVEGRSKAFSTCLPHLVIIA

LFLSTGAFSYLNSKSNGPSRMDLFLSMFYSIVALAMNPIIYSLRNRELKA

SLGRMLCGTVSNSLLFH

>OranORc9223.1P

MRNQATVTEFILLGLTDDTSLKVCLVSFLLLVYLLSVTSNLTIIALTFLV

PHLHTIMCFFLRNFSLMEASFTSVCIPNFLVSIFTGDKTISYSDCKTQIF

FYILLG/LGASEFFLLACMSFNHYAAICRPLSYTTIMNSRVCTLLVFCRC

LAGFFIIVPGVTFGLQQEF

>OranORc9228.1P

NESSFEGFILLGFSDQPHLEAAFFVFVLFFYLLTLLGNTAIMVVSRLDPR

LHTPMYFFLSHLSFLDLCFSTSLAPQTLVNLGEPQKTITFGGCVVQLYVS

LALGSTECILLALMALDRYAAVCQPLRYAMLMHPSLCRRLAAASWLGGVA

NSLVHTTLTLQMPRCGHRHLDHFICEVPALIKLACVDTTINELVLFTLSI

LVLLVPPAFILVSYGFIIRAVLRVGSSEGWRKAFSTCSSHLLVVSIFYGT

IIFMYLQPGSNYSQDQGKFVSLFYTMVTPTLNPIIYTLRNQEVKGALKKL

V

>OranORc9648.1P

DPQIQLFLFLVFLLIYGIALVGNLLTMLTPTPLHSHVLFPE*PVFLDVRF

TSSTDPKMLENFLSDQKSITFPECITQIVSLFILATVKIYLLATMAYNCY

RASGQLLCYPGSLTI*LCVQLVRGAWLVRDINVVVNALLVLRLDFCRPNQ

NLHFSCEFELPPLLQISCSNIFASEMGILSLGVLLGLVSFLLTLISYIHV

ISTLFQICFS*GHGKAFPTCSSHLITVLLFCGTVFFQYMRPSSA/LLGSH

PLALDWVVSIQCSILTPMLNSMIYILKNWDVMRGLKKLLRKFW

>OranORc9697.1

MSNTSTVRGFLLLGSSEVREPRPVHAAPFLPVYPAALTGNLLVVAVALLG

RRLRAPVYFFLGNLALVDLCFVSVTVPKSVAVCLTDCRSISFLSCAAQLF

LVVLFAAPEFFVLTAVSYDRYAAICLPLRYGVVVGRGVCGKMAAASWLGG

GLVGVLFSASTFSLSFCGSHVVRRFFCDVPSLLKITRSEDRSAIDASVTL

SLTVGAVGSVSIAVSYARVIGAVLRMPAAEGRAGAFSTCLPHLLVVTVFL

SSGVFGYLEPPSHSSSVFDLPMPVFYAVGPPAPNPLIYSLRNRDLKAALR

TILKRGFPQTLL*

>OranORc9697.2

MAKGPSQDVANTTTVRGFLLLGFSEVRELRLVQAALFLLVYLATLTGNLL

VVAVTALDRSLRAPMYFFLGNLSVLDLCFITVTVPNSVLDSLTDCNSISF

LGCATQVFLVVLFGGSEFFILTAMSYDRYAAICHPLRYEVLMDRGACVKM

ATASWLGGGLFGVLFSAPTFSLSFCGSHVVQQFFCDVPSLLKITCSEEHV

TIDLSVTAGVATGVVCFVSIVVSYARIFGAVLRMPAAEGRARAFSTCLPH

LAVVTLFVVTSFCAYLKPPSGSPSVLDLLVSVFYVVVPPAFNPLIYSLRN

RDVKAALGRVLGGNGFH*

>OranORc9697.3

MANLTAVRGFFPLGFSEVREVRLVQAAPFLPVYLAAPTGNLLVVAVAVLD

RRLRAPVYFFLGHLSVLDLCLVSVTVPKSILDALTDCSSVSFPACVSQLL

LVILFAASEMFILTAMSYDRYVAICHPLRYEVVMNRGACEKMAAASWLTG

GLFGVLFSASTFSLSFCGPRVVPQFFCDVPSLLTISCSENHVAVDVGVAI

GSCLGFVCLVSIVASYASIFSTVLRIPSAASRSKTFSTCLPHLLVVTVFF

TTSAFDHLKPPSDASPTPDLPVSVFYTVVPPALNPLIYSLRNRDVKAALG

KLLGDY*

>OranORc9741.1P

MTNVTTVTAFLLLGFSEVQELQLVHAVLFLLVYLVALVGNLLIVTITTLG

WCLHIPMYFFLRHLSVLDLCLISITVPKSIHNSLINDGSISFLGCVLQVF

FALSSVFTELVILTVMSYNRYVAISRPLHYEVVMNRGACVKMTTTSWLSW

VLSAAL

>OranORc9748.1

MANHTFVMEFFLLGFWEVRELQLVHAALFLLVYLAALLGNLLIVAITVLD

RRLHTPMYFFLRHLSILDFCYISVTVPHSIHNSLTDRRSISFLGCDAQLY

FFACFASLEIALLTVMSYDRYVAICHPLRYDIIMDGGACGKMAATSWLSG

ALSGLLHTVNIFLLPFCDSNVIHQYFCDIPQLLTLSCSPSIVPELVLIGF

SIMLDFGCFLFMGVSYFHILSTVLRIPSAQARTKGFSTCLPHLAVIAIFF

SCAFIAHLKMPSDSPSVLDLLVSVFYTVLPPALNPLIYSLRNRHEGRPGQ

GPQGETSLKSKVA*

>OranORc9748.2P

MSGHTTVTEFLLLGFLEVWELQLVHATLFLLDYLVALMGNLLIITVTALD

RHLHTPMYFFLRNLSVLHLIIISATIPKSIINLLTDTKSISSGG

>OranORc9748.3P

MTNVTTVTAFLLLGFSEVQELQLVHAVLFLLVYLVALVGNLLIITITTLG

WCLHTPLYFFLRHLSVLDLCLISITVPKSIHNSLINGRSVCFLGCVLQIF

FALSSVFTELVILTVMSYNPYVAISHPLHYEVVMNRGACVKMTTASWLSW

MLSAALHTASTFSFSFCGSNVVG

>OranORc9894.1

MPLTNSSSTTISDFLLNCFVESGSDHLGLSMVLALLFIVALAANAVLLLT

IWLEQSLHQPVYYLLSLLSLLDLVLCLAVIPKVLVIFWWDWRSISFAGCF

LQMFVMNSFLAMESCTLMVMAYDRYVAICHPLRYPSIVTDRFVAKAAFFV

VARNTLISFPIPVFSAWLHYCRTNVIEHCICANLHVSRLSCSDITLSRLY

QFVAGWTLLGSDLVLISLSYTFILRAVLRLKAEGAATKALSTCGSHFILI

LFFSTILLVLILTNVVKKRVHGNIPVLLNVLHNVIPAALNPIVYGVWTKE

IREGILRVLRKVRG*

>OranORc9919.1P

PPSSRHLHTWAASYFFLKHLSIVNLCLISVTLCKSILNALSD/ISFLGCI

SQVLSFVIFGAANRAFLTVMSYDCYLATCSPLLYDIIMNRGACGKMVATS

WLTSGLYTLMDMAATLSSHFCCPRVIHQFFCDVPQLLKPMFPGESRPEVC

GLAAWATLALGCFVCILLPNVHIFWAVLRM*STENRAKAFSTCLPSLTLM

TSFVANSTLPTSSP/LSLFYTLVPPTLNPL

>OranORc10127.1P

SGNLLIVAVTTLNWRLHTPKYFFLRHLSILDLCYISTTVPISIVNSLTDR

REISFLGCVFQVFVFISLASTKMALLTVMSFNRYVAICLPLRYEFVISRG

ACGKMTASSWFSGSLSGLMQTAVTFSLPFRGSNEIHQFFCDIPQLLKLSG

SDWIMAEMDISALIACLAFICFVPMIISYVCIFSAVLRLRSSEGQNKAFS

ICLPHLVVVAFFFSTVSFAYLKPPSDSPSFQDLLVSVFYTGVPPTLNPLI

YSLRNKDMKAALKK

>OranORc10127.2T

SHDRYTAICHPLRYEVVMNRGACGKMAAASWLSGGLSGLMHMATTFSEPF

CGSHVIHHFFCEAPHLLAQAGSTVILREVKVTIFTASLSFLCFISIIISY

IRIFSIVLKIPSVEGRSKAFSTCLPHLVVVILFLSAGAFAYLKSKSNGPS

GVDLFLSMFYSIVPPAMNPIIYSLRNRELKVGLRRMLCGTVSNSLLFH

>OranORc10127.3P

MFNITTGTEFLLLGFSEVRELQLVHGMMFLLVYLAALMGNLLIVTVTALD

ERLHNPMYFFLSNLALIDLCLISVTVPKSVVNSLTNNRSISFLGCVLQVL

FFISLASTEMILLTVMSHDRYTAICHPLRYEVVMNRGACGKMAAASWLSG

GLSGLHAHGHHLL*AFCGS

>OranORc10204.1P

KIHLAEATSKALTNWSSHIILIFFYTAIVVLPITHTAKEKVPLIPVLLNV

LHLSPPGSQP/PPALNPIVYALMTQEIRVGFLKLIR

>OranORc10204.2P

QVTEFILMGFPGIHSWQHWLSLPLTLLYLSAFFS/SALLANVMILLTI*Q

ETVLHQPMFYFLVVLALVDMGLSTTIMPRILAMFWFNASTISFPECFFQI

YAIHVFVGLEFYIFLCMAIDRYVAICHPLYYPSVITK/KGFVLKATLLMV

FRIGLLAIPGTVLAARCNYCSRNRIDHSLCSNLAVTSLACDDRKANSIFQ

LTMAWFLLGSDMGLII

>OranORc10204.3

MFVPIRASNGSSPQVTEFILMGFPGIHSWQHWLSLPLALLYLSALLANAM

ILLIIWQKTVLHHPMFYFLAVLALVDMGLSTTIMPRILAMLWFNARTISL

QECFFQIYAIHVFVGLESGIFLCMAIDRYVAICHPLRYPSVITEGFVLKA

TLFMVFRNVLLAIPLPILATKRHYCSKNEIGHCLCSNLAVTSLACDDWKI

NSIFQLSVAWVLMGSDVGLIIFSYTLILRAVLKLHSAEAASKALSTCSSH

IILILFFYTAIVVMSITHSANKEVPLIPVLLNVLHNVIPPALNPIVYALR

TQEIKVGILKLIRLAGER*

>OranORc10538.1P

MRNQTSVTEFILLGLTDNPDLKIVIFLFLLLSYLLSITGNLTILTLTLLD

SHLHTPSVF/PVYFFLQNFSFLEISFTPVTIPKLLVAISTGDRTISYNAC

VTQLFFYILLGAMEFFLLAAMSYDRYVAICQPLHYLTIMSQSVCTLLVLC

SWMISFLIIFPGLILSLQLDFCDLNVINHFTCDTVPMLEISCSDTQLFEQ

MNFILAVGTLLVTLALVTMSYTAITCTILKLPSTQQRKRAFSSCCSHMIV

ILLSYGSCIFMYIKPSPKEGLDFNKKVAVLNTSVAPMLNPFIYTLRNQQV

KQAIKDLTKRVIF

>OranORc10538.2P

GNGTVVTQFILVGLT/PQLHALLFFILFLTYALSVTGNLTIIHSHLAGLP

PPHPHVLLSAQFLLAGDSLHVRLHFQIPGHDHHRRQNQFLFHCFTQLFFF

ISLVVMESFLLAAMSYDRYIAICRQLHYTTVMSGAFCPLLVLCSWLAGIL

VVFPSVIVLLRHNLCPSNIINHFICDSSPMLSCSDT*FLELMAFLLAFGT

LLFTLALLTVSFTSITRTILRTSSSK*R*KTFYTCSSIVVMVSISYGSCV

>OranORc10538.3P

YQHYYYYYVNPSPKTGISFNKGVAVLNTSVAPMLNPFIYTLRNEQVKQAL

RNLVFQVWCFLKREG

>OranORc10886.1P

NLSTGIKFQLVEFSSNREVRLVQAALFLLVYLAALTGNLLIVAVTVLDRR

LRAPMYFFLGHLSVLDLCYVSATVPQSVHNALTDRRSISLPGCVMQVLSV

ITFACSELFVLTAMSYDRYVAICRPLSYESIMAPGACGKMASASWLVGVL

IGSLNSAGTFSLPFCGPTDLRRFFCDVPSLLKISCSESHLLEDVTLILGS

LLGFLCLGLIAASYARILGAVLRMPAAEGRAGAFSTCLPHLVVVALFVST

VIVAHLKPLSDPPSTPDLLVSVFYAVVPPALNPLIYSLRNRDVKAALEKL

V

>OranORc11057.1

MKKWNESSGTEFFLVGLFAHLSYPSLLVSTLSIVYLLALAGNSLMIFLIW

MDSRLHTPMYFFLSQLSLMDLLLTSVVVPKVLIDYLLNTSMITPVGCGIQ

MFLVMTLGGGEGLLLGFMSYDRYIAVCHPLRYPLLMPQDKCQRLGVGACM

GAALVSLFNTILTTCLPYCRPRKVHHFLCEPPALLKLACVDLSVYEPAVF

VISSIVILIPFLLILASYAHILLAILQMPSTGGWRKVAITCSSHLLVVHL

YYGAAMLMYIRPSSQHSPGEDNILAVFYTVLTPMLNPLIYSLRNKEVTGA

MRKLFREFCA*

>OranORc11057.2P

LFTLILLVFLVSLLGNATVILLIWLDPGLHTPMYFLLSQIFLMDLLNISI

FVHKMAANFLSGNSSSSFVGCGNQLFVFVMLVVVACLLLTTMAFDHCIAI

/CHPRWYLVLMCPKVCGLMVAGSWLASLLNVLIHCVYTLNLPFCAFWEIR

HFCEIPTLLELVCRDTTIHEKGLFLNGVLFLLPPNSIIMVLYRQIFQTNL

GKGSSQDHIK/KALITWSSHVIVVYLFYEAAIFKYHLPKSYHTPSRHQVV

SCSTPTHSHAQPSDLQS*N*EVVGVLKKVLGR

>OranORc11125.1P

MMEVTNDSSGGDFILMGFSDQPQLEMVLFVLILISYLLTLLGNATIVVVF

HLDPHLHTPMYFFLSHLSFVDLCFTTSVIPQLL*NLWGPYKTITVIGCAV

QLSIALSLGSTECVLLTIMAVDRYAAVCRPLHFMTIMHPPLCQGLAAVAW

LSGMGNSAIQSTITLRLPRCGNRRIPNFGCEVPTMIKLACVDIQANEVVL

FIATLILVLLPIVLIVVSYGFIARAVLRIKSSQAWGKALGTCGSQLLVVT

LFFGMISGIYIQSNRSFSKSSGKFLTLFYAVITPTLNRLIYTLRNKDMKE

ALRRLLGKDQS

>OranORc11394.1P

VANLTTLTRFLLAGFSEVRELQLVHAVLFLLVYLAALTGNLLIITTTALD

QRLHTPMYFFLRNLSFIDLCLISVTVSPSS/KSIVTSLTDLHSIYYWGCV

TQVLCVIFFMGSEMCVLMAMSYDRYAAICRSLCYDIIMNRGACGKMVATS

WLSGGLFGVMYSAGTFTLSFCKSNTIQQFFCDVPSLLKISCSKTHLVIDV

SVAIGIGFGSFSIVAIVTSYFRIFSILLMLPSTEGRAKAFSTCLPHLAVT

NVFVFTAGFAYLKPPSDSPSTLGLLVSVFYTVVPPTLNPLIYSLRNRDMK

AAPRRIFSVNLNSG*IDPLFQ

>OranORc11502.1P

XXXXLLIGLSEVQELQM/VHATLFLLIYLAALMGNLLIIAVTTLDRHLHT

PMFFLRHLSVLDLCLISVTVPKSFFNSVTNNNSISFQGCVLQVFFFVSAV

VSEVVLLTVMSYDRYAAICHPLHYEVIMNRGVCGKMAAASWLSGGLSGLM

HTATTFSEPFSGPNVIHQFFCDIPQLLKLSGPQGNISEYSLTVLSASSFA

ICFSYITVSYIRIFSAVLKMPSAEGRSKAFSTCLPHLIVVTFFISTGVSE

YLIPSSNCPTGLDLLLSVFYSMVPPALNPVIYSLRNQAVKAALGRMLCLE

>OranORc11514.1

MANLTTGTRFLLLGFSDVLREKLVHATLFFLVYLVALAGNLLIEAITTLN

RRLYTPMYFFLKHLSVLDLCYISVTVPKSIADSLMGNSSISFWECVFQVF

SFAILANAEMAFLTVMSFDRYVAICLPLRYEVLLRRRVCQRLAVAAWFSG

GLCAILHVANTFSLPFCGSRTIRQFFCDVPQLLKLACPEGILGEVVVSGI

LASLASLCFIAILLSYARIFSTVLRMASAEGRAKAFSTCLPHLGVATLFL

STSGFEYLKPTVDSASALDLGISVFYTVVPPTLNPVIYSLRNREMKAAMA

RVLGRKGSF*

>OranORc11569.1

MATPNGSDSSATFFILIGIPGLEAAQFWLAFPLCSLFLVAFLGNGILVYV

VGTEPGLREPMYLFLGMLSAVDALLATAAMPRMLGLFWFNSTAIAFDACL

VQMFAIHALSAVESSILLAMALDRYVAICHPLRHATLLTPSRVAKMGVAA

VVRGAALMAPLPVFLRHLPFCGSNVLSHSFCLHQDVMNLACADITTSVVY

GLVVIVSAIGLDALLVSLSYALILRAALRLSREARLKALATCVSHAGAVF

LFYAPFIGLSVVHRCGHGASSSLHVVLANVYLLVPPVLNPVVYGIGTTRI

RQRILRVFHSVSRDSST*

>OranORc11849.1

MSALHPSNFSTSSFCLLGFPGLESAYPWIALLLSTIYTIMLLGNSIILYV

LWIEWSLHKPMYYLLALLTTTDLGMGLSTVPTVLGVLWSLHQEISLNSCI

AQAYFIHGLSSIESGVLLAMSWDRFVAICNPLRYASLVTGSTIIKIGAIV

LTRSLLLILPAIIRLRFFFYCRPHVLSHSFCLHQDLLRLACSDIRFNSFY

ALGLVISTLILDSVFILASYVLILHTVLAVAARGERLKSLQTCTSHILAT

MVFYVPIIGLTMVHRFGKHLSPLVHVLMGHIYILFPPLMNPIIYSIKTQQ

IRKRIQGMFYMERG*

>OranORc11857.1

MEEFANQSSTTDFILPGLFTHSGASHFLFSLVCAIFTVALTANAVLILLI

GVDPRLRTPMYFLLSQLSVLDVSFISVTVPKMAVDFLSGRNVISFPGCGT

QMFFFLLVGAAECLLLGFMAYDRYLAVCHPLRYPVLMSRQVCLLMVAGSW

MGGILDGAILTPLTLGFPFCRSREIPHFFCEIPAVLQLSCSDVSRYETGV

FLSGIVFLLLPISFIVASYAFILASVLRMKSTEGRQKAFATCSSHLTVVG

LFYGIIIFMYMTPRSYHTPKQDKTMSVFYTILTPMMNPLIYSLRNKEVMG

ALGRMMRRCTFA*

>OranORc11867.1

MGEANQTCWVQLYFRPFSTDSDVQVLIFVAFLVMYLLGLTGNGAIGLTIW

TNYSLYTPMYFFLANLAILEIFYTTAIAPLAMTALLSTGRTPVPLAGCGT

QMFFFVFLGGADCVLLAIMAYDRFVAICHPLRYRLIMSWRLCVQLLIGAL

VLGFLSSLTLTALIFQLPFRGNIEISHFYCDVPAVMRLARADTRVHETAL

HVISFMVLAIPATLIFISYVPIGMVILRIRSAEGRRRTFSTCSSHITVVL

LRYGCTSFIYLSPSASFSPETGRGMSVVYTFITPILNPVIYSMRNELLKD

ALQKTLRRCLLPQRK*

>OranORc11908.1P

NQKSGNKFIITGLFHHTPVPGLLFILIMLVTLVALLANTTLPFLIWLDPR

LQIPM*FLFCSSS/LFLMDLLYVTIFIPKTAANFLSGDSSISFADCGIHL

FLFMTLMA/VAICHP*QYPVFMCLMVCGLMVATSLLGSLLNAPIQCVYTP

NISFCTSWEIHHFFCDISALLELVCHDTAI*ENGLFLSGILFPLPPISVI

MASYRESLQIVLEKGSSQGS*KVLTTCFSHIVVVCLIYGATIFKYLLPKS

YHTSSQDQVVSVFYTLLTPMSNLLIYSL*N*EALKTLGKVL

>OranORc11973.1P

NRTALTEFLLLSFSDFPDLRTPLFSGVLAAYLATLLGNALIIRVTLVDRA

LHSPMYFLLRHLSFLEILYTTDVVPKTLVDLLALRPAISFGGCAAQMFFF

IVLGITECCLLTAMAYDRYVAICRPLHYPRLLSRPACLRLVGTSWVLGVL

TASTHTSLIFSLPFRGTPTIHHFLCDILPVLRLA

>OranORc11973.2P

MRNYTPVTEFILLGLTDNPRLQTVILFYMSVTYVMSITGNLSIVTLTLLD

SHLRTPMYFFLRYFSFLEICFTSTCIPRFLATLATGNRTISYNCCATQLF

FFFLLGATEFFLLAAMSYDRYVAICRLLHYTTVMSQRVCTLLNAQFLLA/

MLSSC*PGFLVIFPPIILG

>OranORc11973.3T

FSFAGGFCFTSACIPRFLATIATGDRTISYNCCITQLFFFFLLGATEFFL

LAAMSYDRYVAICRPLHYTTIMNQRVCSLLVFCSWLAGFLVIFPPVILTL

QLEFCGSIAINHFFCDVSPLLLLSCSDTGPLELLTFAVAVGTVLITLALV

AVSYTAIAHTILRLPSSQQKRKAFSTCSSHMVVVSITYGSCIFMYIKPSA

KDRVDLTKGVAVLNTSVAPMLNPFIYTLRNQQVKQAIRDLVHRGGFSSR

>OranORc12131.1

MLPAGKVDRIPLLLGATSKETSAFFLEGLGKVTGRANASWGGGFILLGFS

DQPQLEKLLSMLISIFYVLTLMGNGTIILVACLDAQLHTPMYFFLSHLAI

LDLTLINCVIPQMLVNLWGPDRTIPFAGCAVQLFISLGLGGTQCVFLAFM

AYDRYIAICQPLHYVLVMHPGLCWKLLAISWGLGFGNSSLHTPLTLSLPR

CGHHQINHFFCENPPLVKLACVDTSSYEAEIFVLSVFFLLIPLLFILVSY

GFIARAVLEVKSAAGRRKASGTCGAHLAVVSIFYGSILFIYLQPKDQVSQ

DADKFVALFYNLITPALNPLIYTLRNEDVKGAIRRLVLERRGVQIIQVLS

QISLRSVTERLPRAACSVQG*

>OranORc12196.1

MCEPDFLPGWGSRRSREAAAGPCPRCSFLVYLVALTGNLLIVAVTALDRR

LHTPMYFFLRNLSFIDVCYISVTVPKSIFNSLAHRCDIRFPDCVLQVFFF

ISLASAEMGVLTVMSYDRYAAICRPLLYDAVMSRGACAKMAATSWLSGGF

AGLMHTAATFSRTFCGSNMIQQFFCDIPQLVRISHSKGNVAEVGVSLGTI

SLALACFVSMVVSYVYIISTVLKMPSAEGRSKAFSTCLPHLVVVIFFLST

SSFAYLKPTTESPSALDLLLSMFYAVVPPALNPLIYSLRNRE*

>OranORc12196.2

MANCTCVTRFLLLGFSEVRELQLVHAALFLLVYLVALTGNLLIVAVTALD

WRLHTPMYFFLRNLSFIDVCYISVTVPKSIFNSLAHRCDIRFPDCVLQVF

FFISLASAEMGVLTVMSYDRYAAICRPLLYDAVMSRGACAKMAATSWLSG

GFAGLMHTAATFFRTFCGSNMIQQFFCDIPQLVRISHSKGNVAEVGVSLG

TISLALACFVSMVVSYVYIISTVLKMPSAEGRSKVFSTCLPHLVVVISFL

STSSFAYLKPTTESPSALDLLLSMFYAVVPPALNPLIYSLRNRDMKSALE

KMLGRKLSL*

>OranORc12241.1P

VANLTTLTRFLLAGFSEIRELQLVHAVLFLLVYLAALTGNLLIITTTALD

QRLHTPMYFFLRNLSFIDLCLISVTLSPSS/PKSIVTSLTDLHSIYYWGC

VTQVLCVIFFMGSEMCVLMAMSYDRYAAICRSLCYDIIMNRGACGKMVAT

SWLSGGLFGVMYSAGTFTLSFCKSNTIQQFFCDVPSLLKISCSKTHLVSD

VSMAIGIGFGSFSIVAIVTSYFRIFSILLMLPSTEGRAKAFSTCLPHLAV

TNVFVFTAGFAYLKPPSDSPSTLGLLVSVFYTVVPPTLNPLIYSLRNRDM

KAAPRR

>OranORc12355.1P

PTLLLLLLFFLRNFSFLEFSFTSVFVPKMLVNIGTGDKTISFAGCFTQYF

FAILLGATEFYLLAAMSLDRYVAICRPLEYTTIMSRRICVLLVFCSWVSG

FLVVSGPHIMTSMLPFCGANVINHYCCDYTILLQLACSDTHVIEMMELVL

AVVTLILTLALVILSYAYILWTILRIPSVQQRKKAFSTCSSHLIVVALSY

GSCIFMYVNPSVKDAAAFNKGVAVLNTSVAPLLNPFIYALRNQQVRQAFK

NVCRKISVF

>OranORc12355.2P

MENQTTVKEFILLGLTSDPEFQDVLFLFLLLTYALSITGNLTIITLILLD

PHHHTPMYFFL

>OranORc12558.1P

SNESSRGDFFLVGFSDWPQLEPVLFGIVLTSYLLTMLGNTAIIVVSRLDP

RLHTPMYYFLGHLSFLDLCYATSITPGLLANLRGQAQTIGHLGCMVQLCV

SLALGCTECFLLAVMAYDRYAAVCRPLRYAALVPPALCRALAAVSWLGGA

FSSLVQAGLVGALPLCGLRRLDHFLCEMPALLS*LASGIRAGRRPRCS*P

GCLVLLVPVSLIL

>OranORc12714.1

MLAMRNDSLGEHFILMGFSDQPQLEVILFVVVLTSYLLTLVGNTIIIVVS

RLDPRLHTPLYFFLSHLSLVDLCFTTSIVPQLLWNLWGPSKAITVVGCAI

QLYVSLALGSAECVLLPIMAFDRYTAVCRPLHYRSIMHPRFCHALAAVAW

TSGLGNSVIQSTITLQLPRCGHRHLAHFICEVPALIKLACMDTKANEIQL

FVATLVLLLLPMTLIMLSYGFIARAVLRIKSSQAWQKALGTCGSHLLVVT

LFFGMGSVIYIQPNSSFSKTSGKFLTLFYTVVTPTLNPIIYTLRNKDMLG

AVRRLLWKDCCSMKT*

>OranORc12720.1

MLISNTTSFHPSAFLLLGIPGMEDQHLWISIPFCSVYIIALLGNATILLV

VASDKTLHKPMYLFLCILSLTDLVLCSATLPKMLVIFWFGAQHISYHGCL

TQMFFIHMIFGTESAVLLAMAFDRFVAICRPLHYSSILSPGVIGKIVMAC

IVRGLFFVFPFIILIQRLPFCGHHVIPHTYCDHMGIAKLACANIKINIIY

GLTVALLVTGMDVVLIGFSYGFILHTVLHLPSQDAQVKAFSTCGAHICVI

LVFYIPAFFSFFTHRFGHWMPPQVHIFVANLYLLVPPMLNPLVYGINSKH

IRQRILTLILGYK*

>OranORc12832.1

MGNGTAVTEFLLLGFSEVRELQLFHAALFLLLYLAALMGNLLIVAITVLD

RRLHTPMYFFLRNLSVLDLCLISAIVPKSILNSLTNNRSISFPGCILQVF

FFGFAASSEMALLTAMSHDRYVAICCPLSYDIIMHPKACGKMVAASWLSG

ALNGLLHTAASFSSPFCGSKVINQFFCDIPPLLRLSCSRGFHNELGVIAF

NMLIGSSCFASITLSYVRIFSAVLRMRSTSGRAKAFSTCLPHLAVVTLFV

SPALLEYSTFIAKPPSILDLVGSLFYTVVPPTLNPLIYSLRNRDVKTAVR

KLVCRSKII*

>OranORc12832.2T

MANCTCVTRFLLLGFSEVRELQLVHAALFLLVYLVALTGNLLIVAVTALD

RRLHTPMYFFLRNLSFIDVCYISVTVPKSIFNSLAHRCDIRFPDCVLQVF

FFISLASAEMGVLTVMSYDRYAAICRPLLYDAVMSRGACAKMAATSWLSG

GFAGLMHTAATFSRTFCG

>OranORc12885.1P

RRKAFSTCTSHLTVTVLHYGCAAFTYVRPKASYRPRRDKVVALVYTNLTP

LLYPLIYSLRNREVTGAIRKVLG

>OranORc12885.2T

FLLQGFSDLPRFRPLIFVLLLVGHLATLTGNLSILVALALAPRRPPMLLF

LGQLSAIELGYALVVGPRLLADLAGPAPPAGRPISFLGCAAQMQMFVALG

GAECFLLAAMAFDRYMAICLPLRYPAVVTPALCARLAVGCGLGALAVSLG

LTVAVVRLPFCRSRLLPHFFCDVTALLHVACTQSRADELPLLAASVLLLL

LPSLLILASYGAVLAAVLRV

>OranORc13013.1P

MEKGNQTSVSEFLLLGLFDQAEQRQLLFVLFLWMYLLGVLGSLLIILVIS

SNPHLHTPMYFFLTNLSLADVCLLSTIVPKMLVSIQTQDKSISYVGCLTQ

IYFFCLV/FFALLVCLDHFLLTGMAYDRYLAICHPLHYTTIMSPRLC

>OranORc13013.2

MERGNQTAVSEFLLLGLSDRVEQQQFLFVLFLWMHLLGVLGSLLIVLAIG

SNPHLHTPMYFFLTNLSLADICFLSTTVPKMLINVQTHSKSISYTGCLAQ

MYFFILFGGLDMFLLNGMAYDRYVAICHPLHYATIMSPRLCALVVAGSWS

VSTLQALIHTLLVVPLSFCADNEIHHFFCELNQVLKLSCTDTLINDTVVL

ILATVLGVVPLAGLLFSYSRIISTILTIPSARGRGKAFSTCGSHLSVVSL

FYGTGFGVYFSPTSTHSAQKGSIASVMYTVVTPVLNPFIYSLRNKDMKWA

LRKVFNRKTLFS*

>OranORc13094.1P

YDRYVAVGKPLLYVSAMTRGTRVALVAGAYAGGLAGATLRTALTFTLSVC

DANRIDFFFCDLPPLLRLTCGDPSTGETAIIFLGNFMILASGLVILVSYL

FVARTILRRRTAGGRAKTFSTRASHLTAVGLFFGTLALTYLRGGSGKSLE

GDKVVSVLYSVVIPAPNPFIYSLRNDQVKAALRRAFLRLRLALG

>OranORc13464.1P

LLMGFSDTRELQLVQAILFLLV*LAALAGNLLILTITTFD*HLQTPMYIF

LKHLSFLDLCYISVTIPKSFTNSLTCSGSISFLGCADQVFFFSVFVCTEM

GILTVMSCDCFVAICHPLCYEVVMSRGACVQMLASFWASGALAGLMHIIT

TFSLPFCGSNVIYQFFCDVPELLRLSSSNDTAGEVRVTALIAGLFIICFM

FIIVSYTYIFSTVLRIQSAEGKSKVFSTCLPHLLVVTLFLSTGSFEFLKP

ISQSASFVDVLIYVFYTVMPPTMNPLIQSLRNRDMKTTLGKVLGRELF

>OranORc13465.1

MANVTTMMEFLLLGFSEVREQQLVHTVLFLLLYLETLTGNLLIVAITTLN

WRLHTPKYFFLRHLSILDLCYISTTVPKFIVNSLTDRREISFLGCVFQVF

VFISLASTEMALLTLMSFNRYVAICLPLRYEFVISRGACGKMAASSWFSG

SLSGLMQTAVTFSLPFRGSNEIHQFFCDIPQLLKLSGSDWIMAEMDISAL

IACLAFICFVSMIISYVCIFSAVLRIRSSEGQNKAFSTCLPHLVVVAFFF

STSSFAYLKPPSDSPSFQDLLVSMFYTVVSPTVNPLIYSLRNKDMKAALK

KIIGREKFT*

>OranORc13557.1P

MSMDNRNNNNTQSHPSSFLLLGEPGLEAAHN*LGFPFCAMYLITLMSNCT

ILSVIKIKQSLHQPMFYFPAMLA/TIDLGLSSVTIPKMLGIFWLHLRGIS

FGGCLTQMFFIHMFTGMESTVLVAIGYDRYVAICKPLYYCTILTNKSIRV

FLGLVVLANCSLMIVFLLRLPCCGHRIIPHTYCEHMGLARLACASIKLNI

FYGLCVICLFLLDILLIFISYMRILHTVFRLSSQDARLKAINTCISHICV

MLAFFTPVLFSFFTHHFGHNKIHGYIHILLANLYVVIPPALNPII*GVRT

KQICECVASIFTKKPS

>OranORc13557.2P

NPSIFILVGIPELESFHVWFSLLFLLVYIVTILGNCTILLVIAREESLHA

PMYFFLCMLAITDLIGSTTVLPKTLSIFWCDDKEIHIDACLLQMFFIHSL

SVMESTILVAMAFDRYVAICDPLRYNSVLTNSVIVKIGLAIVARGSVLVA

P

>OranORc13788.1P

HTPMCFFLRSLSVCNLCFISVTVSKSILSVLSNHQSISFLGCTSQVFSFV

IFGVVGLAFFTAMSYDHCMTICHPLLYDIIMDRWPLRRWQLHPGSLVDFT

P*CTWPPPSPPTSAVLVSSTSSLVTCDIPQLLKLTFLGEARAEVYVLAVS

VTLVPACFVFLLVSYGHIFWVGLRMQFTEGWAKAFSTCLSHFMVMTLFIA

NGPSF*LPLGPGPAGVHLL

>OranORc13860.1

MGSGTAVTEFLLLGFSEVQELQLFHAALFLLLYLVALMGNLLIVAITALD

QRLHTPMYFFLRNLSVLDFCLISAIVPKSILNSLTNNRSISFPGCILQVF

FFGFAASSEMALLTAMFHHRYLAICCPLSYDIIMHPKACGKMVAASWLSG

ALNGPLHTAASFSSPFCGSKVINQFFCDIPPLLRLSCSRGFHSELGVIAF

NMLIGSSCFASITLSYVRIFSAVLRMRSTSGRAKAFSTCLPHLAVVTLFI

SPALLEYSTFISKPPSILDLVGSLFYTVVPPTLNPLIYSLRNRDVKTAVR

KLVCRSKTI*

>OranORc13860.2P

MANCTYVTRFLLLGFSEVRELQLVHAALFLLVYLVALTGNLLIVAVTALD

RRLHTPMYFFLRKLSFIDVCYISVTIPKSIFNSLAHRCDIRFPDCVLQVF

FFISLASAKMGILTVMSYNHYAAIFCPLLYDAVMSRGACA*MAATSWLSG

GFAGLMHTAATFSRTFCGSNMIHQFFCDIPQLV*ISHSKGNVAEVGVSLG

TINLALVCFVSMVVSYVYIISAVLKMPSTEGRSKAFSTCLPHLVVVISFL

LTSSFAYLKPMPESPSALDLSLSMFYAVVP/PALNSFIYSLRNREKSALE

KMLGTKLSL

>OranORc13925.1

MVTEFLLLGFSEVWELQLVHAALFLLVYLVALTGNLLILAIPTLNQRLHT

PMYFFLRNLSLLDLCYITTTVPKSILNSLTNSRSISFLGCTTQVLLMILF

GGSEFFILTAMSYDRYAAICCPLRYDTIMDQEVCRKMATASWLSGGLYSL

MHTAATFSSHFCGPRIIHQFFCDVPQLLKLMCPGEARAEICVLVLSVILC

LGCFVSILVSYVHIFLVVLKMPATEGRTKAFSTCLPHLIVVFLYLCTSSF

AHLKPRSSSPSQLDLLVSVFYTVLPPTLNPLIYSLRNKDMKATLGKIFSV

HFISRVN*

>OranORc13954.1

MGNKKNVTEFNLLGLSQDQNVQIFCFVLFSFCYIILLMGNLLILITVGCS

HLINQPMYYLLAHLSSVDMCYTSTVTPKLIGDLLTKKKVITFDNCMLQLF

SMHFFTCIEVFILTAMAFDRYVAICRPLHYVVIMSRKKCKLLILVSWVGG

AIHAFPQLFMVFQLPFCGPNEVDHYFCDIFPLLKIACVDTYLVGVLVVAN

SGMVALVTFVVLLVSYAVILFSLRTHSAEGRRKALSTCGSHIAVVFLFFT

PSIFTYLRPPSSYSEDKVFSLFYTIIAPMFNPLIYTLRNTEMKNAMRKVW

CRKVSSEGKLN*

>OranORc13954.2

MAEDNHTVVREFILLGFTESPMSKRILFVLFLGIYFTTLVGNLGMTGLVR

VEPHLHTPMYFFLSNLSFVDACYSSTIAPKMLVNFLAEKQTISFGGCATQ

LCFYIAFATTDILLLAVMAYDRYVAICIPLLYPVMMSRKMCFRLVAGSFL

GGSLSSLIHTSFTFQLSFCHSNIIDHYFCDIPPLLALSCSDTRINEILIF

ALGSLEAAVSLSTIFFSYLFILLTVLRICSAEGRRKTFSTCTSHLTAVVL

LYGTLTFIYLRPSSTSVLDQEPAVSVFYTTVIPMLNPLIYSLRNREVKSA

LRRVLERKLVFWSRCRSDSSVMPR*

>OranORc14135.1

MELINNSKVSEFVFLGLSSVRELEGFLFLVFLLAYLAALLGNLLIIVTLT

CEPRLHTPMYFLLLNLASLDICFSTITTPKMLADLLSETKIISYGGCMAQ

IFFFHLLGGADVFFLSVMAYDRYVAISRPLHYMAVMTWRVCKGLVVTSWI

IGLVHSVLQLALLLPLPFCGPNVLDNFYCDIPQVLRLACTDTSILELLMI

TNSGMLSLIWFILLLASYMIILRMLRSYSKEGRRKATSTCTSHIIVVSLH

FVPCVYTYARPFDSFSPDKVVSVALTVLSPVLNPVIYTLRNKEVRSAMRR

LRGRIMLPGRG*

>OranORc14230.1P

MANHTSVIEFLLMGFSDEHDLQLVHATMFLLMYLMALVGNFIITVITLDR

CFHTPMYFFLNNLSFLDLCVISTTVPKSTFNNLTHRKAISFLECCFQVLR

SFLAVQK*PSSQ*CPMTAMWPSAIPCTMREVIMSRGAYVKMASPSWLSGG

L/GGFATILHVSNTFSLSFCGPSVIYQFFCYIPQVLRLTCSNEISEEVGI

ICLISSLAILCFLLIIYSYTHSFSTVLRIPSAKGQSKAFSPCLSHLAVVT

LFLSTGTFEYLKSPSGSHSLLDLFVSRFYTMVPLTLNPVIYGLRNKDMKA

ALGRIWR

>OranORc14249.1

MADNTTGPPLSSFFLVGIPGLEEFHHWLSIPVFLLYSLSLMGNCLIILII

RLESSLHQPMYFFLCMLALNDLALSSSPAPKMLSIFWLDDHDTGFDTCLV

QMYFIHTFSITESGLLVVMAFDRYVAICQPLHYTTILTNKMVGLMGLVAS

LRAAIMVFPCIVLIKRLTFCTKNIIHHTYCEHMAMVKLACSNVIINRIYG

ICVALSVAVLDTGLITMSYVRILQAVFRLSSNKAQSKALGTCAAHLCAIL

TFYVPSLFSLLTHRIGTKVSPSIHIIFASIYLLVPPAVNPLVYGAKTKLI

RDWVIFTLFSNKETVSKQ*

>OranORc14249.2P

MANNNTGQTLSSFFLIGIPGLEEFHHWLGIPVLLLYSLSLLGNCLIILII

RLEPSLHQPMYFFLCMLALNDLALSSSPAPKMLSIFWLDDHDIGFDACLV

QLCLIHTFSIIESALLVAMAFDRYVAICQPLHYTTILTNKVVVLMGLVAS

LRAAIVVFPCIILIKRLTFCTKNIIHHTYCEHMAMVKLACSNAIINRIYG

ICVALSVVVLDMGLITMSYARILQAVFRLSSNKAQSKALGTCAAHVCAIL

TTYVPALFSFLTHRIGTKVSPS/IHIIFASIYLLVPPAMNPLVYWAKTKL

IRDWVIFTL

>OranORc14308.1P

LHALTHTLLVVRLSFCSIHEIRHFYCDLYQVLGFPVPASQSMKWWCIC*T

WCWS*FPSPVSVLLHSHCLHHIESPISRGRHKAFRSCG*HLSVVSLFYSI

SLGVYLCPPSAFTSQKGSIVSVV*AVVTPLLNPFIFSLRHE

>OranORc14308.2P

PLYFFLTNLSQVDTCFLSTTVPKILVNIQTQDKFISYAGCLEQMHFSILF

VSLDDFLLAGMAYDHYLAISHPLFSTTIRSPWFCALVLSGSWIISSFIHS

IVFIEGLLCAEH

>OranORc14308.3T

HPLHYTTIMSPRFCILVVTGSWFVSSLHALTHTLMVLRLSFCSHHEILHF

FCELYQLLKLSCTDTVINVALLLILLVVLGVVPLTGLLFSYTRIVSTILR

LPSAGGRKKAFSTCGSHLSVISLFYGTGLVVYSSPTSTYASRQGSIASVV

YTVVTPMLNPFIYSLRNRDMKRALSKLVNREKVFFQ

>OranORc14336.1P

LILTMFLVNLAGNSLIIMVIWTDPKLQTPMYFLLSQLSCVDMAFASITVP

ELLVHTFSKRKAITYGSCFVQMTFSWLWATWRATYWPPWPMTAMWPSAGP

CTQELCVRMVAGS*VLVTLNGAQHGIRMSLASYCNNRILHFLCDITFLLP

LACPKPFLNDLVLF/KAMST*GSHVLVVMLFYGRGIYLYFQSPGHFSLEQ

DWHVAIFYMVASPMLNPLIYSLWNRDVQGAMCRLLRK

>OranORc14336.2P

MVPGNETPISEFILLGLSERPEWQLAIFGVILAMYLLTVTSNTLIITVTL

IDPKLQTPMYFLLGRLSFVDMAFASITVPQMLAHLLSTSKSIPFTNCLAQ

LFFVLLVGPMEGYLLAAMAYDRYVAVCKP/NPLRYTTLVSRGLCLRIIVG

SGLVVLGNALLNTVLMMRLSFCSRHILQFFCDITPMLQLSCSRPFLNEML

IFTEGTAIILGPFFFILASYARIGAAVVHLHSAVGLRKAVSTCGSHVLVV

TLFYGPGMRMYLHPAASASEGQERQVAIFYTLVTPMLNPLIYSLRNHEVQ

GALRR

>OranORc14392.1P

MANGTEVTEFLLLGFSEVQELQLVHATLFLLIYLAALTGNLLIIAVTTLD

RHLHTPMFFFLRHLSVLDLCLISITVPKS/FNSVTNNNSISFQGCVLQVF

FFVSAVVSEVALLTVMSYDLYAAICHPLRYEVIMNRGVCGKMAAASWLSG

GLSGLMHTATTFSEPFSGPNVIHQFFCDVPQLLKLSGPQGNISEYSLTVL

SASSFAICFSYITVSYIRIFSAVLKMPSAEGRSKAFSTCLPHLIVVTFFI

STGVSEYLIPSSNCPTGLDLLLSIFYSMIPPALNPVIYSLRNQAVKAALR

RMLCLE

>OranORc14605.1P

MANRTVVMEFLLLGFSEVRELQLIHATLFLLVYLAALAGNLFIVTITALD

RHLHTPMYFFLRHLSVLDLCYISVTIPKSIFNSLTNLNRGACEKMAATSW

LSGALSAILHTSATFSVPLGGSNVIHQFFCEIPQVIRLSDSSGKIWELIA

TTFSASLTLICFVSIVVSYVHIFVAVLRMPSGESRSKAFCTCLPHLIVVT

*FVSTGDAAYLKPVSDSPSVLDLLVSMFYTVVLPTLNPLIYSLRNRDMKA

AMLRML

>OranORc14659.1T

SISLVGCAIQIFIFLTLEGGECFLLAAMAYDRFVAICCPLRYPVLMSPRL

CLLLAVVSWLLGATDGLVQAGITMSYHFCRSREVNHFFCEAPALVLLACD

DTMVFESVMYVCCVLMLLIPFSVILGSYGLILETILRMRSVEAKKKAFTT

CSSHLSVVGLFYGAAIYIYMRPSSYDSTDYDKVVSAFYTILTPVLNPLIY

SLKNREVLGALKRGLTDCRFRNLRVEREAA

>OranORc14699.1

MENLTTVSEFILLGFRGGLWLQGTLFLTFLVLYGVTVVGNLGMVAIISLD

SQLHTAMYSFLCSLSLLEVCYSSTIAPRALLNFLSERAAISFPGCATQFF

FLSLFGTTEAFLLATMAYDSFIVICDPLHYSMIMSHGICHLLVGGLYLWG

VVNAVTQTTMSFRLLFCGPNEIDGFFCNVLPILTPSCSDTLTNQLVLLGL

GGSIIVGTFSILSISYVLVLSTILRILSAEGRMRAFSTCVSHLVGEGLFY

GSVFFMYAQSGATSNMEQSKVVSIFYTVIIPMLNPIIYSLRNKDVKEALK

RIRKKLSL*

>OranORc14771.1

MQHLLFVLFSVFYMAIVLGNSLIVLTVVADPRLHSPMYFLLANLSFFDIC

VSSTVTPKMIYDTLRDSKIISFGGCFAQMIFIHIVGGSEMVLLVAMAYDR

YVAICRPLHYVAIVTLRMCIWIVVAAWIIGLIHAMSQAAFVVNLPFCGPN

EVDSFFCDFPRIIQLACTDTYWLELMVTANSGFISMGTFLLLIFSYVFIL

VSVRKRSSVGLSKAFSTLSAHISVVVLFFGPCIFVYIWPFPKLPVDKTLA

VFDAVITPFLNPIIYTFRNKDMKLAMMRLSVHVGRPAKSARTG*

>OranORc14796.1T

FLWASWNSVVIAVMAYDRFVAICHPVSLHSIMNARLCVQLVALPWIFSGL

ISLAHTILMSRLNFCAANELPHFFCDLTPLLRLSCSDTTANETLVFIVGA

AVIATPFVCILASYARIVLAILKVPSAGGRKRAFSTCSSHLSVVFLFYGT

TIGVYLSPSSGHSALKDKASAVMYTVVTPTLNPFIYSLRNKDMKRALRKV

FYKKV

>OranORc15098.1T

PSSFLLLGVPGLEAAHIWIGFPFCAVYLIALVGNCTILFMIKTEQSLHQP

MFYFLAMLATIDLGLSTATIPKMLGIFWFGLQEISFGGCLTQMFSIHTFT

GMESAVLVAMGYDRYVAICKPLHYHTILVHATIGMIAGIAVVRSFCMMTP

LLFLVLWLPFCGHSIIPHTYCEHMGIAWLACASIRVNVIFGLVLICIFFV

DVILIVFSYIRILYAVFRLPSRDARLRAMSTCGSHVCVILAFFTPALFSF

LAHRFGHNEIPGYVHILLANLYVVVPPALNPIVYGVRTKQIRQQLGKIFN

KKDEF

>OranORc15235.1

MSMDNRNHSGTQSHPSSFLLLGVPGLEATHNWLGFPFCAVYLIALMGNCT

ILFMIKTEQSLHQPMFYFLAMLATVDLGLSSATIPKMLGIFWLRLQEISF

GGCLTQMFFIHMFTGMESTMLVAMGYDRYVAICKPLRYCTILTNKSIRVI

LGLAVLRNFSLIIPMVFFLLRLPYCGHQIIPHTYCEHMGIARLACASIKL

DIIYGLCVICLILLDILLIAISYIRILYIVFRVPSRDARLRALSTCGSHI

CVILALYAPALFSVLTHRFGHNKIPGYIHIFLANLYVVVPPALNPIIYGV

KTKQFRERVASVFTKKPSF*

>OranORc15426.1P

MANRTAVRGFLLLGFSEVREVRPAQAAPFLPVYPAGNLLVVAVLDRRLRA

PVYFFLGHLALVDLCLVSVTVPKSVLDSLTDKRSISFPGCVAQVFLVVFC

AGTDVAPLTVTSHDRYAAIRLPLRYGVIVDRGACGKTAAASWL/SAATFS

LPFCGSDVIHQFFCDVPQLVKLSGVNVRELGAVIFTASSGLGC

>OranORc15426.2

MADRTAAAGFLLLGSSEVREPRPVQAAPFLPVYPAAPAGNLLVVAVAVLD

RRLRAPVYFFLGRLSVVDLCHVSVAVPRSVHNALTDNRSVSLPGRVLQVF

SLLPFARAELYFLTAGSHDRSVAIRLPLRYEVIVNGGACGKMAAASWLGG

GLSALTHPAATFSVPSGGSSVIHRFFCQSPQIGELAHSSGKIQEDAVTSF

SALLASARFVSIAVSYARVFRAVLRMPAAQGRAGAFSTCLPRLAVVTVSV

SLPQARVGLPLDAGPAGVPVLRRGAPGPRPPHLQPEEPGPEGRSGDLKLI

SGR*

>OranORc15646.1

MANCTCVTRFLLLGFSEVRELQLVHAVLFLLVYLVALTGNLLIVAVIALD

RRLHTPMYFFLRNLSFIDVCYISVTIPESIFNSLAHRCDIRFPDCVLQVF

FFISLASAEMGILTVMSYDHYAAIFRPLLYDAVMSRGACAKMAATSWLSG

GFAGLMHMAATFSRTFCGSNMIQQFFCDIPQLVRISHSKGNVAEVGVSLG

TINLALVCFVSMVVSYVYIISAVLKMLSAEGRSKAFSTCLPHLVVVISFL

LTSSFAYLKPMTESPSALDLSLSMFYAVVPPALNPFIYSLRNRDMKSALE

KMLGTKLSL*

>OranORc15646.2T

FLLLGFSEVQELQLFHAALFLLLYLVALMGNLLIVAITALDQRLHTPMYF

FLRNLSVLDLCLISAIVPKSILNSLTNNRSISFPGCILQVFFFGFAASSE

MALLTAMFHHRYVAICCPLSYDIIMHPKACGKMVAASWLSGALNGLLHTA

ASFSSPFCGSKVINQFFCDIPPLLRLSCSRGFHSELGVIAFNVLIGSSCF

ASITLSYVRIFSAVLRMRSTSGRAKAFSTCLPHLAVVTLFVSPALLEYST

FISKPPSILDLVGSLFYTVVPPTLNPLIYSLRNRDVKTAVRKLVCRSKTI

>OranORc15656.1P

MSNHTTVTEFLLLGFLEVRELQLVQATLFLLNYLVALMGNLLIITVTALD

RHLHTPMYFFLRNLSVLDLIIISATIPKSIINLLTDTKSISSVG

>OranORc15656.2

MANVTTVMALLLLGFPQELQLVHAVLFLLVYLAALVGNLLIVTITNLGWC

LHTPMYFFLRHLSVLDLCLISITVPKSIHNSLINDRSISFLGCVLQVFFA

LSSVFTELVILTVMSYNRYVAISHPLHYEVVMNRGACVKMTTASWLSWVL

SAALYTTSTFSFSFCGSNVVGQFFCDIPQLLTISRSPDLLSEVVPICVNV

AFDFCCFIGIIVSYIHIMATVLRMPATEGWNKAFSTCLPHLIIITISLST

GFFAYIKAPLDSPSVMDLLLSMFYTVVPPTLKPLIYSLRNRDLKVTMGKF

LKGKFCTREKSLHISHLA*

>OranORc15656.3P

MANVTTVTAFLLLGFSEVQELQLVHAMLFLLVYLAALVGNLLIVTITNLG

WRLHTPMYFFLRHLSILDLCLISITVPKSIHNSLINDRSIFFLGCVLQVF

FALSSVFTELVILTVMSYNRYVAISHPLHYEVVMNRGACVKMTTASWLSW

VLSAALHTASTFSFSFCGSNVVGQFFCDIPQLLTISHSPDLLNEVVPICV

KVAFDFCCFIGIAVSYIHIMAAGLRMPAT*G*TKAFSTCLPHLIITISLS

TGFFAYLKAFFAYLKAPLDSPLSYGPSAVYVLHSGAPPP*APLIYSLRNR

DLKVTMGKFFKGEILHKEK

>OranORc15780.1

MARGNHTTVTEFILVGLTDRPELQVPLFMLFLAIYFVTLVGNIGMIVLIQ

VDSRLQTTMYFFLSNLSFLDLCYATVFAPKMLANYFSEKKTISFAGCFTQ

CFLFIMYVSSEGILLAVMAFDRYVAICNPLLYNIIVTKKLCINLVVGSYL

GGIINSLSHTGSLLRLPFCGPNVVNHYFCDVPPLLKLPCADTRINEILLF

TLSGILAIITFLFVFISYIFIVATILRIPSTEGRQKAFSTCASHLTAVTL

FYSSVSFSYVQPTSLHSLDQEKVVAVFYTLVIPMLNPLIYSLRNKDVKEA

LKKAMT*

>OranORc15790.1

MKVIGFLTFFMIYLVTLIGNLGLITVIKADAQLHTPMYYFIGHLAFLDLC

YSSAVLPKILDNLLAQNETISFNGCAAQMYFLIVPASSECYLLAAMAYDR

YVAICNPLLYSFIMSQGVCYLLVAGSYLVGFISATTQTYLTFRLSFCGSS

VINHFVCDVPPLLALSCSATYVNEFVLFIFAIFLGVVTSSEILVSYICIL

CAILRIQSAKGRQKAFSTCATHLCSVVLFYGTSTFIYTRSASDYSLGQDK

VISVFYTVVIPMLNPMIYSLRNQEVKRALKRIISIKKPYSW*

>OranORc15790.2

MAQENITIITEFILLGLTDRAELKVIFFLVILVNYALSLLGNIGMIVLIR

TESKLHMPMYFFLSSLSFVDACYSSVFAPQMLAHFFVERGTISRPACIVQ

YFFFVLLLTTEGCLLAAMAYDRYAAIVIPLLYTGVMTKRLCVILVVASYI

GGLINSLTHTIGLAGLAFCGPNVICHFFCDLPPLLKLACSDTSRNELLLL

IFSGVIALFTFLSILFSYIFIVAAILKIRSTSGRYRAFSTCASHLTAVTL

FYGSISFSYIQPHSQYSLEQEKVVSVFYTLVIPMLNPLIYSLRNKEVKDA

VKRRCFR*

>OranORc15795.1P

GFSEVRELQLVHGMMFLVYLVALMGNLLIVTVTALDERFHTPMYFSLSNL

ALIDLCLISVTVLKSVVNSLTNNRSISFLGCVLQVLFFISLASTEMILLT

VMSHDRYTAICHPLRYEVVMNRGACGKMAATSWLSGGLSALMYMATTFSE

PFCSSQVIHHFFCETPHLLAQAGSTVILREVKVTIFTASLSFLCFISIII

SYIRIFSTVLKIPSVEGRSKAFSTCLPHLVVVVLFLSAGAFAYLKSKSNG

PSGVDLFLSMFYSIVPPAMNPIIYSLRNRELKVGLRRMLCGTVSNSLLFH

>OranORc15795.2

MANVTTMMEFLLLGFSEVREQQLVHTVLFLLLYLETLTGNLLIVAITTLN

WRLHTPKYFFLRHLSILDLCYISTTVPKFIVNSLTDRREISFLGCVFQVF

VFISLASTEMALLTLMSFNRYVAICLPLRYEFVISQGACGKMAASSWFSG

SLSGLMQTAVTFSLPFRGSNEIHQFFCDIPQLLKLSGSDWIMAEMDISAL

IACLAFICFVSMIISYVCIFSAVLRIRSSEGQNKAFSTCLSHLVIVAFFF

STGSFAYLKPPSDSPSFQDLLVSMFYTVVSPTVNPLIYSLRNKDMKAALK

KIIGREKFT*

>OranORc15900.1P

KLM*PGEARAKVCDLAVSVTWVLGCFVSILISYVDIFWATLRMRSTEGQA

KASSTCLPHLAVISLYLFMGLFAHLKLPLSSQ*ALDLLVSVFYTVLPLTL

NLLIYSLRNKNMK

>OranORc15900.2

MANVSMVTEFLLLGFSEVWELQLVHAALFLLVYLVALTGNLLILAIPTLD

QHLHSPMYFFLRNLSLLDLCYITTTVPKSILNSLTNSRSISFLGCTTQVL

LMILFGGSEFFILTAMSYDRYAAICCPLRYDTIMDQEVCRKMATASWLSG

GLYSLMHRAATFSSHFCGPRIIHQLFCDVPQLLKLMCPGEARAEICALVL

SVILCLGCLVSILVSYVHIFLAVLKMPATKGRTKAFSTCLPHLIVVFLYL

CTGSFAHLKPPSSSPSKLGLLVSVFYTVLPPTLNPLIYSLRNKDMKAALG

KIFSVHFISRVN*

>OranORc16008.1

MANLSVRSEFFLLGFSEVRELQLVHALLFFLVCLAALMGNLLIIMIITLD

WHLCTPMYFFLKNLSILDLCLISTTVPKTITNSLTNHRSISFLGCAVQVF

SVVLFAGSELFILTAMSYDRYVAICCPLRYEMIMNRGVCVQMAAASWSTA

GLFGMMCSAGTFSLPFCGSHVVPQFFCDVPSVAKLSYSETHIALDVIIIT

GVSFAANFLICILVSYIHIFSAVLRMPFTEGRTKAFSTCLPRLAVTTVFF

TTVAFSHLKSTSNSPSALELLVSVFYNVVPPTLNPLIYSLRNRDMKAAMG

RILGKTLFTRV*

>OranORc16008.2P

IIITVTTLQLVTHYIPHVHLPRKSCRSSLSVSCPAMVPQALLSISMTENS

VSFLGCKCFSSFLQGILKWPCSRPCRRTAM*LSATPPHYEVVMSRATCVQ

VAAASCIFVSLSGLRYMTVTFSVPFCG/PSVDSKVIRQFFCDVPRFLKLT

GDSGNMVEFTIIIMSA/ICFMCIIVSYTHIFSRVLGIPPTEVRSKA*STC

LSHFIIFTSLLSVGIFKYLIPSSNTPSALDVCLSVAYVIMP

>OranORc16112.1

MERGNQTSISEFLLLGLSDRAEQQQPLFVLFLWMYLLGVLGSLLIILAIG

SDPHLHTPMYFFLTNLSLADICFLSTTVPKMLVNIQTHNKSISYARCLVQ

MYFFILLVSLDHFLLTGMAYDRYVAICHPLHYTTIMSPWLCALVVAGSWL

ISSLHALTHTLLVVRLSFCSSHEILHFFCDLYQVLKLSCSSILINEVVVF

VIAVVLAVVPLTGLLFSYTRIVSTISRIPSTSGKRKAISTCGSHLSVVSL

FYGTALGVYFSPTSPQTAKKGSIASVMYTVVTPMLNPFIYSLRNKDMKQA

LRNVFCRKIVFSQML*

>OranORc16112.2P

SLDHFLLNGMAYNHYVALCHPLHYTTIMSTRLCALVAAGSWIISSLCVLI

HTLLVVRSSFRADDKVYHFF/KLFCTDPLISEILLFVIGALLGLGAPTSL

VFSYSHNVSILRIPSTERRGEGFSTCGSHLSMIFLFDGSAFAVYFSPRST

YAPHQGSIASIMY/IYNV*IPMLNPFIYSLRNKDMREAMRKFIPR

>OranORc16112.3

MEKGNQTSVSEFLLLGLSDRAEQRQLLFVLFLWMYLLGVLGSLLIVLAIG

SDPHLHTPMYFFLINLSLADVCFLSTTVPKMLANVQTQDKSISYAGCLAQ

GYFFNLFGVMDNFLLTGMAYDRYMAICRPLHYTTVMNPRLCALMVIGSWL

LSGLDALIHTLLVLRLSFCKNNEILHFFCEINQVLKLSCTDTHINVVLLY

ALIMALGIGPLTGLLFSYTRIVFTILTIPSTRGRYKAFSTCGSHLSVVSL

FYGTGLGVYFSSTSTHASQKDSIASVMYTVVTPVLNPFIYSLRNKDMKGA

LRNLFSRQARLLKRLGPGRE*

>OranORc16179.1P

MFNITTGTEFLLLGFSEVRELQVVHGMMFLLVYLAALMGNLLIVTVTALD

ERLHTPMYFFLSNLALIDLCLISVTVPKSVVNSLTNNRSISFLGCVLQVL

FFISLASTEMILLTVMSHDRYTAICHPLRYEVVMNRGACGKMAAASWSQR

GPLRPHAHGHHLL*ALLWIPRDPPFLCELPHLLAQ

>OranORc16179.2

MFNITTGMEFLLLGFSEVRELQLVHGMMFLLVYLAALMGNLLIVTVTALD

ERLHTPMYFFLSNLALIDLCLISVTVPKSVVNSLTNNRSISFLGCVLQVL

FFISLASTEMILLTVMSHDRYTAICHPLRYEVVMNRGACGKMAAASWLSG

GLSGLMHMATTFSEPFCGSHVIHHFFCEAPHLLAQAGSTVILREVKVTIF

TASLSFLCFISIIISYIRIFSIVLKIPSVEGRSKAFSTCLPHLVVVILFL

SAGAFAYLKSKSNGPSGVDLFLSMFYSIVPPAMNPIIYSLRNRELKVGLR

RMLCGTVSNSLLFH*

>OranORc16409.1

MNNHTSVTRFLLLGFSEVRELQLVHTALVLLVYLAALTRNLLVVTITTLD

RHLHTPMYFFLRNLSILDLCLISVTVPKSILNSMSQNRSISFLGCVFQVL

LVILFAASEMFVFTVMPYDRYVAICSPLRNEVVMGRGACVKMVVTSWFSG

GLLGVVFSTGVSSLPFCDSHEVQQFFCDIHSLLKISCSEKHVAEDISIAM

RAASGFFSFGFVVLSYIHIFLAVLRMPSSDGRSKAFSTCLPRLVVFTLFI

TTGSFAYLTPPSASPSTLDLLVSVFYMVVPPNLKPLIYSLRNWDMKATLG

RVLGSA*

>OranORc16443.1P

ANFTPVTEFLPLGFSEVRELRPVQAAPFLPVYPAAPPGNLLVVAVPVLDR

RLHTPRYFFLGNLSVLDLCLVSVTLPNSVVDSLTDRRSISLLGCVTQVLL

LIFFGSAETAFLTTVSYDRYVAICLPLRYEVLTSRGACGKTAGAS/GGGL

SGLPHPAAAFSGPSCGSNAIRQFFRDIPQKLSGSNADLGEVGVTTFTASL

STVCFASTTVSYVGVFGAVLRTRSAEGRAKAFSTCLPRLAVVTFFISTSG

FAYLKPPSDSPSTLDLLVSAFYSAVPPTSNPLIYSLRNGDVKAAIWKTL

>OranORc16635.1P

MANRTVVTEFLLLGFSEVRELQLIHAALFLLVYLAALAGNLFIVTITALN

RRLHTPMYFFLRHLSVLDLCYISVTIPKSIFNSLTNLNRGACEKMAATSW

LSGALSAILHTSATFSVPLGGSNVIHQFFCEIP/FVRFPQVIRLSDSSGK

IWELISTTFSASLTLICFVSIVVSYVHIFVAVLRMPSGESRSKAFSTCLP

HLIVVT*FVSTGDAAYLKPLSDSPSALDLLVSMFYTVVLPALNPLIYSLR

NRDMKAAMLRMLGGK

>OranORc16699.1

MENQTTVKEFILLGLTSDLEFQDVLFLFLLRTYALSITGNLTIITLTLLD

PHLHTPIYFFLCNFSFLEISFTSVFVPKMLVNIGTGDKSISFAGCFTQYF

FAILLGVTEFYLLAAMSLDRYVAICRPLEYTTIMSRRICVLLVFCSWVSG

FLVVSGPHIMTSMLPFCGANVINHYCCDYTILLQLACLDTHVIEVMELVL

AVVTLILTLALVILSYAYILWTILRIPSIQQRKKAFSTCSSHLIVVSLSY

GSCIFMYFNPSVKDAAAFNKGVAVLNSSVAPLLNPFIYALRNQQVRQAFK

NVCRKISIF*

>OranORc16735.1

MSNHSSGTWFLLGEFSEVRELQLLQATLFLVAYLAALTGNLLLLAVITLH

RGLHTPMYFFLKNLSFLDLGYVSVTVPRAIFGSLTGDASISFWECVFQVF

SFAGFGNAEIALLTVMSYDRYVAICQPLRYEVIMKRTACAQMAGASWVSG

GLSAILHTAVTFSVPFSGRNVIRQFFCDIPQMIRLSGPRGNPGEVGLIFL

MAGLSLACLVSITVSYALIFSTVLKFPSAKGRSKAFSTCLPHLAVVSLFL

LTGSSEYLKPTSGDPLLLDQLLSVFYTVIPPALNPIIYSLRNKDIKAALG

KGLWGMK*

>OranORc16786.1P

MEKKNESAVSELVLLGLSGFSEAAAAGVVLLTVLWICLIGLLGNLLIILT

VRSDSHLRTPMY*FPSHLSFVDLCRS/VDLCLTTSTVPKMPLNIQTQKKT

ISYAGCKSRCTSSWTDG/LHGLMDGFLPTAMA*DRYEVICPLL*YSLIMN

PQLCVFLVAGAW/LHTVVTARLSFCGDSETPFCCCDLTLLIKISCSSTGV

NEVVIFIVAGLVGMTPPICIPVSYIQALPHV

>OranORc16915.1

MMEVTNSSSGRDFILMGFSDQPQLEMVLFVLILISYLLTLLGNTIIVVVS

HLDPHLHTPMYFFLSHLSFVDLCFTTNAIPQLLWNLWGPYKTITVIGCAV

QLSVGLALGSTECVLLTIMAFDRYAAVCRPLHYMTLMHPRLCQGLAAMAW

LSGMGNSAIQSTITLRLPRCGNRHIPNFGCEVPTMIKLACVDIRANEVVL

FIATLILVLLPIALILVSYGFIARAALRIKSSQAWRKALGTCGSHLLVVT

LFFGMISGIYIQSNRSFSMSSGKFLTLSYAVITPTLNPLIYTLRNKDMKK

ALRRLLGKDQS*

>OranORc16982.1

MFVSIGASNGSSPQVTEFILMGFPGIHSWQHWLSLPLALLYLSALLANAM

ILLTIWQEMVLHQPMFYFLAVLALVDMGLSTTIMPRILAMLWFNARTISL

QECFFQIYAIHTFMGLESGIFLCMAIDRYVAICHPLRYPSVITEGFVLKA

TMFMVFRIGLLAIPVPILAARRNYCSRNEIDHCLCSNLAVTSLACDDRKA

NSIFQLTIAWFLLGIDVGLIIVSYALILRAVLKLHSAEAASKALSTCSSH

IILILFFYTAIVVLPITHTAKRMVPFIPVLLNVLHSVIPPALNPIVYALR

TREIKLGILNLIGLAGKRK*

>OranORc17022.1P

LGFSEVRELQQVHATLFLMFSGGSDRESPHL*I*ILDRQLHTPMYCFLRN

LSILDLCLISVTVSNSILNSLTDCRPISFLGCVLQVFFFISFASSKITLL

TAMSYDRYVAICCPLHYEVIMNPRACGKMATT/GLVGRWPPPSWLRGGLS

GIMHSATVSSLPFCRVNVVHQFFCDIPQFMRLSGSEGMLQEVGVSTFLIL

LALICFAFIGVSYVHIFWAVLKMPSVEDWAKAFSTCLPHLVAVTV/YLKP

PSDFPSMPDLLVSVFYTVVPPALNPLFYSLRNRDMKSALGRVAWGKL

>OranORc17297.1P

MVSANHTNLDPSVFFLLGIPGLESVPFWFSLPVCSICLVTVLGNCTTLLA

ILSEPALRKPMYLFFCMLSLIDLVACASILPKMLSIFWFNAG*IDTNACL

VQMFFIHSFCMMESTVLLAMAFDRYVAICHPLRYAAIFTNAIMVKIGLAA

IARGSLLMFPCPFLIKRLSFCGTNHLPH/IIFHTYCEHMAVVKLACGDTT

ANWVYGLAAALLVIGVDLLFIGLSYFLIVRAVLRLSSQKARAKTFSTCGS

HICIILISYMPALFSFYSH*FGVRVAPHIHILLANLYLLFPPMLNSMIYG

VKIKEVCNKVVRVFL

>OranORc17617.1

MATPVMDGTNTSLVTEFVLLGLTSSHELQLTFFVIFSAFYLAIVLGNLLV

ILAVASDPHLHSPMYFLLANLSFIDVCQASFATPKMIVDFLSEPKTISYN

GCITQIFFVHLFTGSEQVFLVAMAYDRYVAICQPLHYMAIMSWRKCVGLS

VTSWVIGFIHSSSQLAFTVNLPFCGPNVVDSFFCDLPRVTQLACVDTYVL

GLFIIADAGLISTSCFVLLLASYTVILATVWRRQSSTSMAKARATLSAHI

TVVILFFGPCVFIYLWPFTTYPVDKVLAVFYTVATPILNPIIYTLRNKDM

KAALKKLSSHHLSSKGSQGSLGRKTFPL*

>OranORc17617.2P

LKTKPMVGDNRSAVSEFVLLGLADSHELQLGFFMVFFTLYVAIVLGNLLV

ILAVTTDLRLHSPMYFLLANLSVFDLCQASFATPKMIADFLSEHKTISYN

GCITQIFFIHLFTGGEMVLLVAMAYDRYVAICRPLRYVTIMSRRFCTILV

LTSWAAGFVHTLSQLSFTVHLPFCGPNVVDSFFCDLPRVTQLA*VDTYVT

ELLIMANSGLLSLTSFLFLLTSYAVILSTLWLKSSAAMAKALSTLTAHIT

VVILFFGPCVFIYMWPFTTYPVDKVLAVFYTVANPILNPIIYTLRNKDMK

AALRHL

>OranORc17617.3P

VTEFVLLGLPSSRAPRLLLSCLFSAAFAAIVLGNLLIVVSVRADRRLLQA

PMYLFLAHLAFLDLGLGCVTVPKVLEDLLRGRNTISFPACLAQVCALHLL

GASEMLLLTAMAFDRYVAICRPLRYLAVMDGRLRRRLVTACWAGGFVHSA

TQAALVLRLPFCGPNRLDNFYCDVPQLTKLACADTAVVEALMVANSGLLS

LACFVVLLASYTVILATLRSRLRHGRGLATCASHLTVVSLIFGPCVFIYL

RPFRSFPVDKVISVFYTVVTPGLNPLVYTLRNAEVKAATQRL

>OranORc18856.1P

NDLPRLEEMALFGILLTLFGNISIIFLLTMDSLLGTSMHVFLPTSS/TYL

LHQQLHITWSSDKTILDLGHATQLYFVLAFVGVECVMLAIMSCNCYAIMC

QPLCYPAIMLL*L*GQLEYLSLIEWADHCISPCGHC*SGLFLCEMPVLIG

IARVNIMSLVELAFVLVILIILS

>OranORc19003.1P

MFNITTGKEFLLLGFSEVQELQLVHGMMFLLVYLAALMGNLLIVTVLDER

LHTPIYFLLSNLALIDLCLISTTVPKSVVNSLTNNRSIPFLGCALQVLFF

ISLASTEMILFTVITHDRYAAICHPLRYEVVMNRGACGKIAAAS*LSGGL

STLIHTATTFSEPFCGSHVIHHFLCEAPHLLAQAGSTVILREVKVTIFTA

SLSFLCFISIIISYICIFSIVLKIPSVEGRSTAFSTCLPHLVIIALFLST

GAFAYLNSKSNGPSRMDLFLSMFYSIVALAMNPIIYSLRNRELKASLGRM

LCGTVSNSLLFH

>OranORc19003.2

MANVTTMMEFLLLGFSEVREQQLVHAVLFLLLYLAALMGNLLIVAVTTLD

QRLHTPMYFFLRHLSILDLCYISTTVPKFIVNSLTDRREMSFLGCVFQVF

FFISLASTEMALLTVMSFDRCMAICLPLHYEFIISRGACGKMVASSWFSG

SLSGLMQTAVTFSLPFRGSSEIHQFFCDIPQLLKLSGSDWIMAEMGISAR

IACLAFVCFVSMIISYVCIFSAMLRIRSSEGWNKGFSTCLPHLIVVAFFF

STGSFAYLKLPSDSPSFQDLLVSVFYTVVPPALNPLIYSLRNKDMKAALK

KILGREKFT*

>OranORc19093.1P

MEKLTTVTEVILLGFLEGSWLQGTLFLTFLVLYGMTVVGNLGMVAIISLD

PQLHTAMYSFLCSLSLLEVCYSSTITPRALLNFLSERAAISFPGCATQFF

FLSLFGTTEAFLLASMAYDRFIVICDPLHYSMIMSHGICHLLVGGLYLWG

VVNALTQTTMIF*LLFCGPNEIDGFFCDVLPILTPSCSDTLTNQLVLLGL

GGSIIVGTFSILSISYVLVLSTILRILSAEGRMRAFSTCVSHLVGVGLFY

GSVFFMFAQSGATSNMEQSKVVSIFYTIIIPGLNPIIYSLRNKDVKEALK

RIRKKLSL

>OranORc19460.1T

NHTQFHPSSFFLLGLPGLEAAHIWIGLPICAAYLISFVGNCTILFVIKTE

QSLHQPMFYFLAMLATVDLGLSTTTIPKMLGIFWLGLREITFGGCLTQ

>OranORc19460.2P

VRAAILGEPHYPAHYCEHMGIARLACASIRTNIIYGLVALSMGFFDTSVI

GFSYIQILRAVFHLSSWNARFKALNTCGSHICVILAFYIPAFFSFLTHRF

GHQKIPLYIHILLANLYVIVPPALNPVIYGVRTRQILEQV

>OranORc19475.1

MERGNQTGISEFLLLGLSDRMEQQQLLFVLFLCLYLLGVLGSLLIILAVI

SDPHLHTPMYFFLTNLSLADVCFLSTTVPKMLVNIQTHNKSISFAVCLVQ

MYFFILFGSLDHFLLTGMAYDRYVAICHPLHYTTMMSPRLCALMAGGSWL

ISSLHSLTHTLLVVRLSFCSKREILHFFCELYQILKLSCSSIIINKAMVF

VFATVIALPPFIGLLFSYIRIISTILRIPSAGGRWKAFSTCGSHLSVVSL

FYSTTFGTYLFPASTQAFSKGLIASVLYTVVIPTVNPFIYSLRNKNMKEA

LRNTFKRKSVFP*

>OranORc19475.2P

MERGNQTSISEFLLLGMSNQVEQRQLLFLLFLWMYLLGVLGSLLIIILVI

VSDPHLHTPMYFFLTNLSLADVCFLSTTVPKMLVNIQTPSKSITYAACLV

QMYFFILFISLDHFLLTGMAYDRYVAICHPLHYTTIMSPRLCGLVLAGSW

LISSLHAPTHTLLVVRLSFCSNREVLHFFCELYHILKLSCSNILINEVAV

FVAAVVIGLAPLTGVLFSYTCIIFTILRIPSKGGRWKTFSTCDSHLSMAS

LFYSHEFKSQL

>OranORc19728.1

MANVTTVTAFLLLGFSEVQELQLVHAMLFLLVYLAALVGNLLIVTITNLG

WCLHTPMYFFLRHLSVLDLCLISITVPKSIHNSLINNRSISFLGCVLQVF

FALSSVFTELVILTVMSYNRYVAISHPLHYEVVMNLGACVKMTTASWLSW

VLSAELHTASTFSFSFFGSNVVGQFFCDIPQLLTISCSPDLLSEVVPICV

NVAFDFCCFIGIIVSYIHIMTTVLRMPATEGWNKAFSTCLPHLIIITISL

STGFFAYLKAPLDSPSVMDLLLSMFYTVVPPTLKPLIYSLRNRDLKVTMG

KFLKGKFCTREKSLHISHLA*

>OranORc19728.2P

MSNHTTVTEFLLLGFLEVRELQLVHATLFLLNYLVALMGNLLIITVTALD

QHLHTPMYFFLRNLSVLDLIIIHSFIQ*YLLSAYYVQSTV

>OranORc19728.3T

MSNHTTVTEFLLLGFLEVRELQLVHATLFLLNYLVALMGNLLIITVTAID

QHLHTPMYFFLRNLSVLDL

>OranORc20000.1P

LLYFLRCSEVGILMAMSYNRCVAICWPPCYDVIMAPGACGKMALASWLSG

QLMGILHVSNIISLPFCGPAMIHHFFCDVPQVLKLVCPGDTMGEVGVICL

IVTVAFFSLILIIYSYVSIFWAVLRMPSTEGWPKAFSTCLPHLVVVTLFL

SSGGFEYLNPVPDSPSLPDFLLPVFYAIVPPSLNPVIYSLRNRDIKSALG

>OranORc20012.1

MRNHTPVTDFILLGLTDDPNLQAVILLYMSVTYILSVTGNFTIVTLTLMD

SSLRTPMYFFLRCFSFLEICFTSTCIPRFLATIVTGDRTISYNCCATQLF

FFILLGATEFFLLAAMSYDRYVAICRPLHYTAVMSQRVCTLLVLCSCLTG

FVVIFPPVILGLQLDFCGSVAIDHFFCDVSPLLLLSCSDTWFLELMALVL

AMGTLLLTLALVTASYVAIVRAILRLPSTQQKRKAFSTCSSHIVVVSITY

GSCIFMYIKPSAKERVELTKGVAVLNTSVAPMLNPFIYTLRNQQVKQAFR

VMVHRIGFSSRR*

>OranORc20085.1

MEPGNETGVSGFILLGLSREPGTQRLLFVVFLGLYVVTALGNLLIVLAVG

SDPRLHSPMYFFLANLSFADVGLTSATVPKMLADLRTQSPTIPYAGCLAQ

LYFFMTFGALDDFLLATMAYDRFVAICHPLRYATAMSPWRCGLLLTVCWG

LTSLAALVHTLLLSRLSFCADNPAIPHFFCDLAPLLPLSCSDPTLNRVML

LIVGTMVLVAPLGLILGSYARIVAAVLRVPSAGGKRRAFSTCGSHLAVVS

LFYGTAVAVYLCPPSSRSAGKDRIAAVLYTAVTPLLNPFIYSLRNRDLHR

ALRRALWTQDRHCVLHRALRNRHLHQSLHWALQRAP*

>OranORc20159.1P

LHYTVLKHPGLCWWLVGVAWLCRLTNSLVLSTIALVLSRCGHRQVDHFIY

GVPALIKMAFVDTRWSEVTVFDFGVLILMMPASLLLSSSYAITRAVLKIK

S/VKRQKAFETCGSQLTVASLFFGTIIYMYILPHTSSSQEQGKFLTLLYT

TVTPVLNP/CAQPLTYTFRNKDIKRALKRFLGQ

>OranORc20159.2P

MLSGASGSSLRDFILVGFTDQGTLQVLLFGVILLLYLLNLLGNKAIILVS

RLDP*LHTPMYFFLCHLSFIDAALSTYIIPHLLWNLGGPEKTISYRGCVI

QLYVALMLSSTECILLATMAYDHYAAVCHPLN

>OranORc20341.1P

MENQTTVKEFILLGLTSDPEFQDVLFLFL*RTYALSITGNLTIITLTLLD

PHLHTPMYFFLCNFSFLEISFTSVFVPKMLVNIGTGDKSISFAGCFTQYF

FRHSLG/FFAILLGVTEFYLLAAMSLDRYVAICRPLEYTTIMSRRICVLL

VFCSWVSSFLVVSGPHIMTSMLPFCGGNVINHYCCDYTILLQLACSDTHV

IKVMELVLAVVTLILTLALVILSYAYILWTILRIPSVQQRKKAFSTCSFH

LIVVSLSFMYLNPSVKDAAAFNKGVAVLNSSVAPLLNPFIYALRNQQVRQ

AFKNVCRKISIF

>OranORc20348.1P

ACSEVGILMAMSYNRYVAICWP/PPCYDVIMAPGACGKMALASWLSGQLM

GILHVSNIFSLPFCGPAMIHHFFCDVPQVLKLVCPGDTMGVVGVICLIVT

VAFFSLILIIYSYVSIFWALLRMPSTEGRPKAFSTCLPHLVVVTLFLSSG

GFEYLNPVPDSPSLPDFLLPVFYAIVPPSLNHVIYSLRNRDIKSALG

>OranORc20348.2P

MANVTTVREYLLLGFSEVRELQLVHLLVYLLALTGNLLIFVVTAFNQRLH

TPMYFFLRHLSVLDLCLISVTVPKSISNAVINSRSISSARCGLQVLFFTF

CACSEVGILMAMSYNRYVAICWPPCYDVIMAPGACGKMALASWLSGQLMG

ILHVSNIFSLPFCGPTMIHHFFCDVPQVLKLVCPGDTMGVVGVICLIVTM

AFFSLILIIYSYVSIFWALLRMPSTEGRPKAFSTCLPHLVVVTLFLSSGG

FEYLNPVPDSPSLPDFLLPVFYAIVPPSLNPVIYSLRNRDIKSALG

>OranORc20425.1P

LLTEPMGGDNRSVVTEFVLLGLSDYWAMQRLLFVLFSVFYVAIVLG/VGN

TLIVLTVVSDPRLHSPMYFLLANLSFFDICVSSTVTPKMIDDTLRESKII

SFGGCFAQMIFIHIVGGSEMVLLVAVAYDRYVTICRLLHYVAVVTF/VVA

WVVGLIHALSQAAFVVNLPFCIPNEEDSFFSDSPRVIQLACTDTYRLEFV

VTANGGLISVGTFLLLIFYIFILFSVRHRSSFGLSKAFSTLSAHISVVVL

FFGPCISVYVCPFPTLTFDKFL/FDAVVTPVLNPLIYTFRNKDMKMAMKR

LGSH

>OranORc20433.1P

AAVSEFILLGLTDGKMPQSILFVAVPTDPPGHYLGGGGNLSIILAILLEP

QLHNPMYFFLGNLSLTDISCITVTVPHRLGNLLSQKKTISLAGCLAQLFF

FHLLTGANCFLLTAMAYDR*LAICHPLDYRARMKWSVQVALVGASW/NAL

THTVAMSTLYFCGPNVINHFFCDIPPLCQLSCSSNHHNQLLLFVTVIFMA

LIPIGFLVLSYTHIVVAILRLRSAEGLRQTFSTCASHLIMVCIFYGTGIF

SYMRLGSGDASEKDKVIGILNSIVSPTLNPLIYSLRNAEVKGALRKVFTR

R

>OranORc20592.1P

SIFILIGIPKLESFHVWFSLLFLLSSCS/LLAQFLLLGNCTILLVITLEE

SLHAPMYFLLCMLAITDLIGSTTVLPKTLSIFWCDDKEIHIDACLLQMFL

VHSLSVMESTILVAMAFDRYVAICDPLRYNLVLTNSVIMKIGLAIVARGL

VLIAPLCCLAKRLPFCRTNIIANTYCKHMELVKLACSGFLVSKVYGLTVA

LSVVGLDSIFIALSYFRIIQTVLRLSSPEARVKAFGTCRAHITVILMGYI

PALFSFLSHRISHNVAPYIHVIIGNPYILVPPAFNPIMYGMKTKEIRN

>OranORc20679.1

MANHTFVMEFFLLGFSEVRELQLVHAALFLLVYLAALLGNLLIVAITVLD

RCLHTPMYFFLRHLSILDLCYISVTVPHSIHNSLTDRRSISFLGCDAQLY

FFACFASLEIALLTVMSYDRYVAICHPLRYDIIMNRGACGKMAATSWLSG

ALSGLVHTVNIFLLPFCDSNVIHQYFCDIPQLLTLSRSPSIVPELVLIGI

SIMLDFGCFLFMGVSYFHILSTVLRIPSAQARTKGFSTCLPHLAVIAIFF

SCGFIAHLKMPSDSPSVLDLLVSVFYTVLPPALNPLIYSLRNRHEGRPGQ

GPQGETSLKSKVA*

>OranORc20679.2P

MSNHTTVMEFLLLGFLEVWELQLVHATLFLLDYLVALMGNLLIITVTALD

RHLHTPMYFFLRNLSVLHLIIISATIPKSIINLLTDTKAIS

>OranORc20732.1

MWQTLSFCNSSSFTPMLFTLNGIPNLEALHAWLSLPFCSIYVIAMVGNCG

LLYLICHEEALHQPMYYFLAMLSSTDLAMCSSTIPRMLLLFWFNLREIDF

STCLVQMFFVHTFTGMESGVLMLMALDRYVAICYPLRYSTILTNPVIAKA

GLATFLRGAFLIIPFTILTKRLPFCQSHVIPHTYCDHMSVAKLSSGNIKV

NTIYGLMVAILIGGFDIFCISASYTIILKAVVSLSSVEAQRKAFSTCTAH

ISAIIISYVPAFFTFFTHRFGGHTIPHHVHIIIANLYLLLPPTMNPIIYG

VKTKQIREAVVRLLGWKTALTPKRPD*

>OranORc20732.2P

VENVASMSFPNSSSFVPMLFTLNGIPNLEALHALLSLPLCSMHVITMVGN

CGLLYLICHEEALHQPIYYFLAMLSSTDLAMCSNTVSRMLLLFWFNLREI

NCSTCLIQMFFVHTFMGMESGVLMLMALDHYVAICYPLRHSTILTNAIIV

RAGLATYLRVAILSIPFTFFNKRLPFCHGHVIPHTYCDHMSVAKVSCGNI

KVSAVYGLTVAILIGGFDIICIPHPTL*WI*HHLHFRIQHCDP*GCGEPV

FSRGEHKAFSTCTAHISVIIITYVPAFFSFFTHRFGGTPSP/GHTIPHHK

HIIIANLYLVLPPMMNPIVYGMKTKQIQEAVVRMLGGKRGNDP

>OranORc20808.1

MANHTFVMEFFLLGFSEVRELQLVHAALFLLVYLVALLGNLLIIAITVLD

RRLHTPMYFFLRHLSILDLCYISVTVPHSIHNSLTDRRSISFLGCAAQLY

FFACFASLEIALLTVMSYDRYVAICHPLRYDIIMDGGACGKMAATSWLSG

ALSGLVHTVNIFLLPFCDSNVIHQYFCDIPQLLTLSCSPSIVPELVLIGV

SIMLDFGCFLFMGVSYFHILSTVLRIPSVQARTKAFSTCLPHLAVIAIFF

SCGFIAHLKMPSDSPSVLDLLVSVFYTVLPPALNPLIYSLRNKDMKAALG

RVLKGKLP*

>OranORc20808.2P

MSNHMTVTEFLLLGFLEVWELQLVHATLFLLD*LVALMGNLLIITVTALD

RHLHTPMYFFLRNLSVLHLIIISATIPKSIINLLTGTKSIS

>OranORc20809.1T

MSLSNHTHVNPTSFFLMGIPGLESAHFWLAFPFCSMYLLAVLGNVTVLLV

VATEASLQQPMFLFLSMLATLDLLLCSATVPKILAIFWFDASWISLGACA

AQMFFIHGFSAVESGVLLAMALDRHGAICRPLHYATILTRANVAKMGAAA

FLRGLGLMTPLTCLVQ

>OranORc20809.2P

PSSFLLTGIPGLETQHIWLGIPIFSLFVVSMLGNTAILWVVRSERSLHQP

MFLFLAMLAVTDLVLSTSTVPRMLAVFWLGAQEIGLDACLAQMFLIHCFA

TVESGIFVAMAYDRYVAICTPLRHCAVLPFAVVARIGLAALLRGDTVHRP

PP/GTLYIGPLPLLLRWRLPSYRHRVIAHSYCEHMAVVALACGDQRPSNL

YGMAIGFLVLILDSDGHHCLLCSDLRRRAGAGLPPKPGR

>OranORc21098.1P

LLLLTGSDWIMAKVGVWALITSLALICFVCMIIS*VCIFSAVLRMRSSE/

KAFSTCLPHLIVMTFFLPKGSFDYPK/SDSPSSQDLLVSVLCAILQPEDS

DMKVAL*KILGREMLT

>OranORc21238.1P

MEKLTTVTEVILLGFLEGSWLQGTLFLTFLVLYGMTVVGNLGMVAIISLD

PQLHTAMYSFLCSLSLLEVCYSSTIAPRALLNFLSERAAISFPGCATQFF

FLSLFGTTEAFLLASMAYDRFIVICDPLHYSMIMSHGICHLLVGGLYLWG

VVNALTQTTMIF*LLFCGPNEIDGFFCDVLPILTPSCSDTLTNQLVLLGL

GGSIIVGTFSILSISYVLVLSTILRILSAEGRMRAFSTCVSHLVGVGLFS

GSVFFMYAQSGATSNMEQSKVVSIFYTVIIPVLNPIIYSLRNKDVKEALK

RIRKKLSL

>OranORc21307.1

MGPTNQTGDSVFILLGVSSDPGQQRILFVLFLTLYLVTVGGNLLIVGAIG

TDSNLHSPMYFFLSNLSLVDICFSSTTVPKMLANMQTGSHTISYVGCLSQ

VYFSFLFGDLDEFLLAVMAYDRFMAICQPLGYTTAMSPRRCVLLVAACWV

MAQFNSLLHTILLAQLTFCADRTVPHFFCDLAPVLLLSCSSTSINELALI

SVGGTVIVLPLMCILGSYAHIMSAILRMPSAGSKHKAFSTCGSHLAVVSL

FYGTVIGEYLCPSPPGSTDESSLAAVLYAVVTPMLNPFIYSLRNHKLQRA

LHRFLGRKKPSNL*

>OranORc21307.2T

MGSANKTGDSTFILLGLSHDPGQQRLFFVLFLVLYLVTLGGNLLIILAIG

TNSHLHSPMYFFLSNLSLVDICFSSTTVPKMLVDLQTGRPAISYTSCLTQ

LYFLVSLVALDNLLLTAMAYDRYMAICHPLRYTTVMSPGRCALLLTMCWT

LSVLYGLTHTALMDTLSFCASRGVGNIFCEMYALLRLSCSDTRLNQAMLL

ATGSLLFIAPFLLVLLSYTHIAMAIARVSSAQAVPKPSPPAASHLHVVSL

FLWDTVWX

>OranORc21405.1T

NENSFEGFILLGFSDEPHLEAAFFVFVLFFYLLTLLGNTAIMVVSRLDPQ

LHTPMYFFLSHLSFLDLCFSTSLAPQTLVNLGGPQKTITFGGCVVQLYIS

LALGSTECILALMALDRYAAICQPLRYAMLMHPSLCQRLAAASWLGGVDN

SLVHTTLTLQMPRCGHRHLDHFICEAPALIKLACVDTTINELVLFTLSIL

VLLV

>OranORc21446.1

MGNGTEVTEFLLLGFSEVQELQLVHATLFLLIYPVALTGNLLIVTVTTLD

RHLHTPRFFFLRHLSVLDLCLISVTVPKSFFNSVTNNSISFQGCVLQVFF

FVLAVVSEVALLTVMSYDCYAAICHPLRYEVIMNRGVCGKMAAASWLSRG

LSGLMHMATTFSEPFSGPNVIHQFFCDVPQLLKLSGPQGNISEYSLIVLS

ASSFAICFAYIIVSYIRIFSAVLKMPSAEGRSKAFSTCLPHLIVVTFFIS

TGVSEYLIPSFNCPTGLDLLLSIFYSMVPPALNPVIYSLRNQAVKAALRR

MLCLE*

>OranORc21476.1

MANHTFVMEFFLLGFWEVRELQLVHAALFLLVYLAALLGNLLIVVITVLD

RRLHTPMYFFLRHLSILDLCYISVTVPHSIRNSLTDRRSISFLGCDAQLY

FFACFASLEIALLTVMSYDRYVAICHPLRYDIIMDRGAYGKMAATSWLSG

ALSGLVHTVNIFLLPFCDSNVIHQYFCDIPQLLTLSCSPFIVPELVLIGV

SIMLDFGCFLFMGVSYFHILSTVLRIPSVQARTKAFSTCLPHLTVIAIFF

SCGFIAHLKMPSDSPSVLDLLVSVFYTVLPPALNPLIYSLRNRHEGRPGQ

GPQGETSLKSKVA*

>OranORc21616.1

MSPQEMANVTDSLLAGFILVGFSDRPRLEMVLFGVVFVFYLLTLFGNMTI

ILLAALDLRLHTPMYFFLTNLSFLDLCYTTSSIPQMLYNLRGPDKTISYL

GCAIQLYFVLAFGGVECVLLAVMSYDRYAAICQPLRYSVIMSPRLCGWLA

SLAWLSGMGNSLVMTPQTLLLPRCGHRRVDHFLCEMPALIGLACVDTLSL

EGLAFTLGILIVLAPLMLILASYIFITRAVLRIKSVVGRRKAFNTCGSHL

TVVCLFYGTIIYVYLQPADSPSQDQGKFLTLFYTIITPCANPLIYSLRNK

DVKGALKKVFGRGGEPGGA*

>OranORc21616.2T

MVNATVEGGFYLMGFSQVQGILIIHAMLFLGIYLAALMGNLLIITVTTLD

HQLHTPMYFFLRNLSFIDLCLISVTIPRSIVNSLAGNTSISYLACVFQVF

>OranORc21846.1P

MNNHTSVTRFLLLGFSEVRELQLVHTALFLLVYLAALTRNLLVVTITTLD

RHLHTPMYFFLRNLSILDLCLISVTVPKSILNSMSQNRSISFLGCVFQVL

LVILFAASEMF/FVFTVMPYDRYVAICSPLRNEVVMGRGACVKMVVTSWF

SGGLLGVVFSTGVSSLPFCDSHEVQQFFCDIHSLLKISCSEKHVAEDISI

AMRAASGFFSFGFVVLSYIHIFLAVLRMPSSDGRSKAFSTCLPRLVVFTL

FITTGAFAYLTPPSASPSTLDLLVSVFYMVVPPNLKPLVYSLRNWDMKAA

LGRVLGSA

>OranORc22019.1T

GNPPEMVNATKVTGFLLLGSSEVREPRPVQAAPFLPVYPAAPTGNLLVVA

VAVLDRRLRAPVYFFLGNLSLIDLCYVSVTVPKSIHDSPTDRREISFLGC

AAQVFSVVLFGGSEVFLLTAMSYDRYAAICLPLRYEVVMDRGACGKLAAA

SRLFGGLFAVMSSSGTFSLPFCGSFLIPQFFCDIVSVVRLSCSETHLVID

AVVVTGTLLGVLCFVSIAVSYARVFRAVLRMAAAEGRARALSTCLPRLAV

>OranORc22019.2P

MTNVTLKTEFLPLGFSEVREPRPVQAAPFLPVYPAAPTGNLLVVAVTALD

RRLRAPVYFFLGNPSVLDLCLVSVIVPKSVHDSLTDNNSISFLGCIVQLF

LWILFAGSEYFILTAMSYDRYAAVCLPLSYGVVMDRAACGKMAAASWLGG

GLIGVLFSASIF

>OranORc22019.3T

LKITCSEDHVATDVSMATGVALAVVCFVLIVVSYVHIFWAVLRMPAAEGR

AGAFSTCLPRLVVVIAFFSMGTFAYLKPPSDSPSTPDPLASAFYTVVPPA

LNPLIYSLRNKDVKSALGRILKGTLSRLPLWRKISL

>OranORc22223.1

METLGKNNDSESMSGFVLLGFPCRREIRPLLFSFFSASYALTLLGNGAIV

CAVRRDRRLHTPMYLLLGSFSFLEICYVTSTVPNMLANILSETKAISLAG

CFLQFYCFFSLGTTETFHLSVMAFDRFLAICRPLRYPSIMTPQLCTLLVV

TCWVGDFLCFVLPVYLVARLPFCSPRVIDHFLCDPGPLLALSCTPAPGVE

LTCAGLSSLLLLSTLVFILGSYALVLRAVLRVPSAAGRRKAFSTCGSHLA

VVALFYGSVMGTYVSPGAGSPGGGQKVVTLFYSVLTPLCNPLIYSLRNQE

MKAALWKGLGARGAGPRRGRGCRDPTSSWEPQK*

>OranORc22223.2T

RLHTPMYLLLGGFAFLEICYVNSDVPNMLANIFSETKAISLAGCFLQFYC

FFSLGTTECLFLSVMAFDRFLAICRPLRYPSVMTKRSCAHLVISCWAGGF

LWFLLPVYLVARLPFCGPNVIDHFLCDLGPLLALAAACSPIPGTVLICGL

MSSLLIFATLLFILGSYALVLRAVLRVPSAAGRRKAFSTCGSHLAVVALF

YGSVMVTYVSPGAGQTEGMEKFTTLFYSVLTPLCNPLIYSLRNQEMKNAL

RAGLG

>OranORc22231.1P

ANHTMVLQFLLMGFSIIRELQLGYASLFHLVYQLALMGNLLIITTIILNW

QLLTLMYFFLKNLSFLSLCYISITVPKFILNSLPNHSSISFLRCLF*V*G

VAYLAVTELALLTIMCYDCYAVICQPLCYAIAMNRQSCLQMAAASWFTGT

LTRILHVYSTFSLPYCGSNMVHQFFCEIPQILKLSCSGEILGELKVIVFI

SCLIFISFLFIIYFYAHIFSVVLKIPSAEG*AKAFSTYLSHLVVVFLFVF

SGC*GHLLKPCRL*IR*YLWCPQPEPFIYSLRNRDMKAALGKLIG

>OranORc22265.1P

LAQYPINLAGKRLIITLSWNDTKLWTPMYFLLSQFSFVDMAFASIT*ILV

HTFSKRKAIPYGSCFVQVTFILAVGNMEGYLLGTMAYDCYVAICRPLHYA

IIMTQDLCACTVLGSWILVTLHDALHGIWLSLASYCNNRILHFFCDIPFL

VSPACSKPFLNDLVVFTEGISVVVSPFLLILASYAHIGAALLRLHSAEGL

HKAVSTCSSHVLVVTLFYGMIIRLYFHLPSHSNLEQDTVASPMLNLFIYS

LRNHDVQGAMHRL

>OranORc22309.1P

MSNCTTVMEFLLLGFSEVRELQQVHATLFLLVYLAALMGNLLIFAVTILD

RHSTPP/LHTPMYFFLRNLSILDLCLISVTVPTSILNSLTDRRPISFLGC

VLQVFFFISFASSKITLLTSMSYDRYLANCCPLHYEVIMNPRACGKMA/Q

GLVGRWPPPSWLRGGLSGIMHSATVSSLPFCRVNVVHQFFCDIPQLMRLS

GSEGMLQEVGVCASLTLLALICFAFIGVSYVHIFWAVLKMPSVEDWAKAF

STCLPHLVVVTVNKQL*VSEASLRLPLDAGPADVRVLHSGAPCPEPPLL

>OranORc22385.1P

MFNITNGTEFLLLGFSEVRELQLVHGMMFLLVYLAALMGNLLIVTVTALD

ERLHTPMYFFLSNLALIDLCLISVTVPKSVVNSLTNNRAISFLGCVLQVL

FFISLASTEMILLTVMSHDRYTAICHPLRYEVVMNRGACGKMAATSWLSG

GLSGLMHMATTFSEPFCGSHVIHHFFCEAPHLLAQAGSTVILREVKVTIF

TASLSFLCFISIIISYIRIFSIVLNIPSVEGRSKAFSTCL/HLVIVVLFL

SAGAFAYLKSKSNGPSGVDLFLSMFYSIVPPAMNPIIYSLRNRELKVGLR

RMLCGTVSNSLLF

>OranORc22442.1P

MVNHTTVMEFLLLGFSEVRELQLVHAVLFLLLYLVALTGNVLIVAITTLD

WHLHTPMYFFLRHLSILDLISVTLPKSILVSLTTCHSISFLDCCLQVLLV

LLFATSELFILTAMSYDRYTTICHPLR*EVIMAQGACGKMAATSWLSRCL

LGASLSAGALSLPICGPREIQQIFRDIPSLLKISCSEEHTTLDLTVAIGF

CLTLFCFVSIVVFYMHIFSTLLSIQSAEERSKTFFPCLPHLTITSAFFTT

GSFPYLAPTSESPSALDLLVSVFYIMVPPALNPLIYILRNRDIRTSLGRV

LQG

>OranORc22442.2P

MVNHTTVMEFLLLGFSEVRELQLVHAVLFLLLYLVALTGNVLIVAITTLD

WHLHTPTYLFLRHLSILDLISVTLPKSILVSLTTCHSISFLDCCLQVLLV

LLFATSERFILTAMSYDRYTAICHPLR*ERDHQSSDERSSQGACGKMAA

>OranORc22621.1P

VIEFLILRFSEVWELQLVQATLFLL/YLVALTGNLLIVTVTALDWRLHTY

MYFLFRHLSILDLCYINVTTPKSILNSLTDSHSISFLGCAFQVFVFIRCS

CALTPLLTAMSFDHFAAICHPLHYEVIMDNGAWVKMSAASWFFGTLSTMT

HNVTTFSTLIWWSNDLPRFFCDIPQLIRLTGPDRNLQEFVAKTSSAGLTF

GYFLAIVVTYVCIFWAVLKMLTAEGQAKAFSTCLPYFAVVTLFIA/HLKP

VTDSPSAVDLLMPVFYTVVPPTLNLLIYSLRNQDMKATLRRMI

>OranORc22753.1P

FLEVWELQLVHAALFLLVYLVSLVGNLLIVTVTALNQHHHTPMYFFLRNL

SFVDLCLISITVPKSIHNSLTHTRAISFLDCIAQIFLVILLVAVELFILT

VMSYECYVVICHP/PPCYDVTMNRETCVKLATASWLGGGIFGMMYTAGSF

SLLFCGSPIFLQFLCDASSLLMVSCSNTHVVFAFSLVSGFVCALFSFVST

VISYICIFWAVLRMPATEGWAKVFSTCLPHLAIITVFLSTASIAYFKPPQ

IPP/DSPSAVELVVSVFYTTVPPALNPLIYSLRNRDVKAALEKVLE

>OranORc22951.1

MHFGDLAAVNHTTVTEFILVGLIDNPDRKVIGFLTFFMIYLVTLIGNLGL

ITVIKADAQLHTPMYYFIGHLAFLDLCYSSTVLPKILENLLAQNETISFN

GCATQMYFLIVPASSECYLLAAMAYDRYVAICKPLLYSFIMSQGVCYLLV

AGSYLVGFINATTQTYLTFRLSFCSSNVINHFVCDVPPLLALSCSATYIN

EFVLFVFAIFLGVFTSSEILVSYICILCAILRIQSAKGRQKAFSTCATHL

CSVVLFYGTSTFIYTRSASDYSLGRDKVISVFYTVVIPMLNPMIYSLRNQ

EVKRALKRIISIKKPYSW*

>OranORc22951.2

MAQENITIITEFILLGLTDRAELKVIFFLVILVNYALSLLGNIGMIVLIR

TESKLHMPMYFFLSSLSFVDACYSSVFAPQMLAHFFVERGTISRPACIVQ

YFFFVLLLTTEGCLLAAMAYDRYAAIVIQQWLYTGVMTKRLCIILVVASY

IGGLINSLTHTIGLAGLVFCGPNVIIHFFCDLPPLLKLACSDTSRNELLL

LIFSGVIALFTFLSILFSYIFIVAAILKIRSTSGRYRAFSTCTSHLTAVT

LFYGSISFSYIQPHSQYSLEQEKVVSVFYTLVIPMLNPLIYSLRNKEVKD

AVKRRCSR*

>OranORc22953.1P

MSNHTTVTEFLLLGFLEVRELQLVHATLFLLNYLVALMGNLLIITVTALD

QHLHTPMYFFLRNLSVLDLIIIHSFI

>OranORc22953.2

MANVTTVTAFLLLGFSEVQELQLVHAMLFLLVYLAALVGNLLIVTITNLG

WRLHTPMYFFLRHLSVLDLCLISITVPKSIHNSLINNRSISFLGCVLQVF

FALSSVFTELVILTVMSYNRYVAISHPLHYEVVMNRGACVKMTTASWLSW

VLSAELHTASTFSFSFFGSNVVGQFFCDIPQLLTISCSPDLLSEVVPICV

NVAFDFCCFIGIIVSYIHIMATMLRMPATEGWNKTFSTCLPHLIIITISL

STGFFAYLKAPLDSPSVMDLLLSMFYTVVPPTLKPLIYSLRNRDLKVTMG

KFLKGKFCTREKSLHISHLA*

>OranORc22999.1P

MSRVNHTTMSEFILLGFSHLGGSQRLLFGLFLPIYLVTLEANAVIVTTIV

LDRRLHVPMYFFLATLSCSETLYTLVIIPKALVDLLAQHRAITITACAFQ

MGSFLFLGCSHSFLLAAMGFDRYVAICRPLNYARLMTRGVCMVLVAAASS

CGSAVSLVVTFLIFGSPGPSSGRLQHFFCDIPPVLKGDLASWLP/KATLP

PGSPRRGVILALGALVLVIPLFLIAASYAHIVATILRIPSSRGRVKPFST

CASHLIVVTIHYTCASFIYLRPGAGDSSGRDALVSVTYTIVTPLLNPVIY

SLRNRDFKAALNRVFERIFCPARL

>OranORc22999.2

MSRENCTTMSEFILLGFSHLGGSQRPLFGLFLLLYLVTLGTNMVIVTTIV

LDHRLHMPMYFFLATLSCSETLYTLVIIPKALVDLLAQHRAITITACAFQ

MGSFLFLGCSHSFLLAAMGFDRYMAICRPLSYTRLMTRGVCVALVVAAFF

CGSAVSLVVTSLVFGSSSPSSSHLQHFFCDIPPVLRATSPPGSQRRVVIL

ALCSLVLVIPLFLIAASYACIVATILHIPSSGGRVKPFSTCASHLIVVTV

HYACASFIYLRPGAGDSSGRDALVSVTYTVVTPLLNPVIYSLRNREFRVA

LYRVFERICCPALC*

>OranORc23007.1P

YNRHTAICWPLH*SGTMTRGTCLGLVAASYALGGFN*ANQKGNVSALPF*

GPNRVGHFFCDTPPLLWLACAKTATTQRVLYLISCLVTLVPAGVILISYG

LVPVAVGWMCSLAGQEKALSTWASHILAIAIFYGTMAFTFVQPHSVGELT

HARVVSVVFIIVTSMLNPFIYSLRNKEVKNA

>OranORc23045.1P

KLM*PGEARAEVCALAVSVTWVLGCFVSILISYVDIFWATLRMRSTEGQA

KASSTCLPHLAVISLYLFMGLFAHLKLPLSSP*ALDLLVSVFYTVLPLTL

NPLIYSLRNKNMK

>OranORc23097.1P

GLTDNPNLQTVILLYMSVTYVISITGRLSIVTLTLL/MYFFLHCFSSLES

CLASTCIPRFLATIATGNRTIPYKHCATQLFFFSAGDN*VIPPASMSYDC

CVTICRPLHYTTIMSKRVCILLVLCFLLAG/LLAGRFLVIFPPIILGLHL

DFWGSVVTDHFS*VASPMLLLSCLDTWLPELMVLALVVGRVLITLALVVI

TYTSIVCAILRLPFSQQRRKAFSTCSSHMVVVSITSSSCIFMCIKPSTKD

RVDLTKGLSLLYTSMAPMLNPFIYTLWTQQVKQAIRDLVHGV*FFFKG

>OranORc23097.2P

TKFIPLGLTDNLHLLVVILLYVSVT*AKRVSSNLDHQSDLTLLASRPQRP

/TPMYFFLRSFSLLEIRFRSACIPRFLATIATRDRTISYNCCITHLFFFF

LLRATEFFLLAAI

>OranORc23097.3

MRNHTPVTDFILLGFTDDPDLQAVILLYMSINYVLSVTGNMTIVILTLLE

SRLRTPMYFFLRCFSFLEICFTSTCIPRFLATIITGDRTISYNCCITQLF

FFILLGATEFFLLAAMSYDRYVAICWPLHYTTVMSQRVCILLVLCSCLTG

FLVIFPPVILGLQLDFCGSVAIDHFFCYVSPLLLLSCSDTWFLELMALVL

AVGTLLLTLALVTTSYMAIVCAILRLPSTQQRRKAFSTCSSHIVVVSITY

GSCIFMYIKLTAKERVELTKGVAVLYTSVAPMLNPFIYTLRNQQVKQAFW

GIVHQVGFPSRR*

>OranORc23397.1

MENLTTVTEFILLGFRGGPWLQGTLFLTFLVLYGVTVVGNLGMVAIITLD

SQLHTAMYSFLCSLSLLEVCYSSTIAPRALLNFLSERAAISFSGCATQFF

FLSLFGTTEAFLLASMAYDHFVAICDPLHYSMIMSHGICHLLVGGLYLWG

VVNTVTQTTMSFRLLFCGPNEIDGFFCDVLPILTPSCSDMLTNQLVLLSL

GGSIIVGTFSILSISYVLVLSTILRILSAEGRMRAFSTCVSHLVGVGLFY

GSVFFMYAQSGATSNMEQSKVVSIFYTVIIPMLNPIIYSLRNKDVKEALK

RIRKKLSL*

>OranORc23442.1

MSSLSNNSFTQPSEFHLMCLPGPRSHQLGLSVVLALLFIVALGANALLLL

TVRLEESLHQPVYYLLSLLSLQDLVLCLTVIPKVLAIFWLGWWSISFAGC

FLQMLIVNTFLTMESSTFLVMAYDRYVAICHPLQYPSIITDCFVAKAIVF

IVTRNFLLLAPVPILSARLHYCGNTIIRNCLCGNLSVSQLSCGNITLNQL

YQFVVGWTLLGSDLVLITLSYAFILRAVLRLKAEGAAAKALSTCGSHFIL

ILFFSTILLVLVLTNVVKKRVHADIPILLNVLHYIIPAALNPIVYGVRTK

EIKEGIHRVLRKVRG*

>OranORc23520.1

MKTSNISLASSSTFILVGIPNLEAVRAWLSIPFCLMYLTILVGNSTILYV

IQKDPVLHQPMSHFLAMLAFTELGVSLSTMPTVLGIFLAGATEIDFNACL

AQMYFIHSFSIMESGVLLAMAFDRFVAIYSPLRYSLILTHRRIRATAVGL

SLKSAVLMAPLAVLLKRLPFCHRENVLSHSYCLHSNLISLPCTDTTLNNI

YGLFVVLSTFGLDSVLIALSYALILKTVLGIVSGDGWLKALNTCVSHMCA

VLVYYVPMIGLAMMHRFGQHPSPLLQVMMANAYLFIPPVVNPIVYSLKTK

EIRKGIFRNLIQKRGRP*

>OranORc23696.1

MTQSNQTIVTEFILLGFSHLPELKPLLFVLFLGMFLITLVGNSLIIFVTM

TSSALRSPMYFFLRNLSLLEICYSLDIVPRLLIDLLADRRRISLPACALQ

LLLILSCVTSECILLTVMAFDRYVAICQPLRYGALMSPRLCLWLAVGTWM

AGVPVSLAFTLWLFSFPFCGRQEIHHFVCDISPLLRLVCADTGVFETHVL

AATILVVLVPFALIAVSYGRILSAVLGVRLVSGRSRALSTCTSHLLVVAL

FYGTAGVIHLQPRASYSPESKELVSLSYTVVTPMLNPIIYSLRNEEVRVA

LWRMWGKKKRSRTS*

>OranORc23928.1

MDCEQEMKNQTTDASPGFILSGFSTHPAAHLGLFFLSLLLFLLILLGNLT

IVLLAWTDSVLLSAPMYFFLRYFSLLEMGFTSVTVPKLLADSFSSCHLIS

FAGCATQTFFFIALGSTECALLAVMAYDRYVAVCRPLRYLQEMRPELCAQ

LVAGAWLSGFFNSTVHTAAVFQLSFCGSRVVSQFFCDLPPLLRLACGDRV

ASEAVVLTFGSLYGLTAFLVTLASYARVLFTVLGMGSTPGQYRAFSTCSS

HLAVVGLFYGSAFSTYVQPVSARPTSQALLLPFFYALVTPTLNPFIYSLR

NREVKQALIRALGRKLF*

>OranORc23966.1

MMQDSKWGNQSVVSEFILMGFATTSTTAPLLFFFFLVIYLLILLGNGLLI

TLVQLDQRLHTPMYFFLSFLSMLDISYTTTTVPQMLAHLLSQKRSISFAS

CVAQMYIFLLFGVTESWLFSIMSVDRYFAICHPLRYKVIMSRWTCLLLAG

ICGTYGVVGSLVYTYFAMRLPYCGPNGINHFFCEVPAVLKLACADTTLND

WVDFIVGFNVIVVPQSLILVIYVNIFVAILRIRSAQGRLKAFSTCASHIT

VVTMFGLPCILMYMRPSSQSNPEDDKKMAIFYNIITAFLNPIIYSLRNKD

VKRAFLKAIGRGRTSE*

>OranORc24105.1P

MENLTSVTEFILLGFRGGPWLQGTLFLTFLVLYGVTVVGNLGMVAIITLD

SQLHTAMYSFLCSLSLLEVCYSSTIAPRALLNFVSERAAISFPGCATQFF

FLSLFGTTEAFLLATMAYDHFIVICDPLHYSMIMSHGICHLLVGGLYLWG

VVNALTQTTMIF*LLFCGPNKIDGFFCDVLPILTPSCSDTLSNQLVLLGL

GGSIIMGTFSIVSISYVLVLSTILRILSAEGQMRAFSTCVSHLVGVGLFY

GSVFFMYAQSGATSNMEQSKVVSIFYTVIIPGLNPIIYSLRNKDVKEALK

RIRKKLSL

>OranORc24170.1P

MANHTVVTEFLLLGFSEVRELQLIHATLFLLVYLAALEGNLFIVTITALD

RRLHTPMYFFLRHLSVLDLCYISVTVPKSIFNSPTNLN*GACEKMAATSW

LSGALSAILHTSATFSVPLGGSNVIHQFFCEIPQVIRLSDSSGKIWELIA

TTFSASLTLICFVSIVVSYIHIFVAVLRMPSGESRSK/QTFSTCLPHLIV

VT*FVSTGDAAYLKPVSDSPSVLDLLVSMFYTVVLPTLNPLIYSLRNRDM

KAAMLRMLGGK

>OranORc24401.1

MENQTTVKEFILLGLTIDPEFQDVLFLFLLLTYALSITGNLTIITLTLLD

PHLHTPMYFFLRNFSFLEISFTSVFVPKMLVNIGTGDKTISFAGCFTQYF

FAILLGATEFYLLAAMSLDRYVAICRPLEYTTIMSRRICVLLVFCSWVSG

FLVVSGPHIMTSMLPFCGANVINHYCCDYTILLQLACSDTHVIEMMELVL

AVVTLILTLALVILSYAYILWTILRIPSVQQRKKAFSTCSSHLIVVGLSY

GSCIFMYVNPSVKDAAAFNKGVAVLNTSVAPLLNPFIYALRNQQVRQTFK

NVCRKISVF*

>OranORc24658.1P

MSVSTRASNGSILQVTEFILMGFPGIHSWQHWLSLLLALLYFSALLDNVM

ILLTIWQETVLHQPMFHFLAVLAQVDMGLSATIMSRILPILWFNARTISL

PECFFQIYAIHVFVGLESGIFLCMAIDRYVAICHSLRYPSVITESFVLKA

TLFMVFRNVLLAIPLPILASKRIYCPRNEIDHCLCSNLGVTSLACDDRKA

NSIFQLSVAWILTGSNVGLIILFYALILWTVLKLHSAEAASKALSTCSSH

IILILFFYTAIIVLSITHIAKKKVPLIPVLLNVLHNVILP/PALNPIVYA

LRTQGIKVGILKLIGLAGER

>OranORc24780.1P

MAEDNHTVVREFILLGFTESPMSKRILFVLFLGIYFTTLVGNLGMTGLVR

VEPHLHTPMYFFLSNLSFVDACYSSTIAPKMLVNFLAEKQTISFGGCATQ

LCFYIVF/FATTDILLLAVMAYDRYVAICIPLLYPVMMSRK

>OranORc24780.2T

FTFQLSFCHSNIIDHYFCDIPPLLALSCSDTRINEILIFALGGLEVAVSL

STIFFSYLFILLTVLRILSAEGRRKTFSTCTSHLTAVVLLYGTLTFMYLR

PSSTSALDQEPAVSVFYTTVIPMLNPLIYSLRNREVKSALRRVLERKLVF

WSRCRSDSSVMPR

>OranORc24927.1

MSSANQTGDSAFVLLGLSSDTGQQQLLFLLFLTLYLVTVGGNLLIILAIG

ADSHLHSPMYFFLANLSLVDICFSSTTVPKMLADMQTGSHTISYVGCLSQ

VYFSFLFGDLDDFLLAVMAFDRFMAICQPLGYTTAMRPRCCVRLVATCWI

IAQLNSLLHTILLAQLAFCANHTVPHFFCDLTPLLALSCSDASINKMMLM

SVGGVVILIPLICILGSYAHIISAVLKMPSAGSKRKAFSTCGSHLVVVML

FYGTVIGVYLCPSHSGSADESSLAAVLYAVVTPLLNPFIYSLRNHDLHRA

LHKLFFQRMPFILLESFQGPK*

>OranORc24927.2P

NQSSVSAFVLLDLSTDPGQQQLLSMIFLALYLVTIVGNLLIILVIGTDSH

LHSPMYFFLINLSVVDVCFSSTTVPKMLADMQTGSHTISQADCLSQVYFS

ILFGDLDDFLLAVMSFD*YMAICRPLCYATAMSSQCCVLLVATCWVIAQL

NSLLHTVLLAQLTFCADHTIPHFFCDLALLLPLSCSDTSINELVLMSMGG

AGILIPLMCILGSYAQIISAILRMPSAGSKRIAFSTSSSHLAVVSLFYGT

VISEYLCPSPSGSSDESSLAAVLYAVVTLLLNPFIYSLRNHDLHRALCTF

FCRKTPFILLQTFQAPK

>OranORc24946.1P

KLM*PGEARAEVCALAVSVTWVLGCFVSILISYVDIFWATLRMRSTEGQA

KASSTCLPHLAVISLYLFMGLFAHLKLPLSSP*TLDLLVSVFYTVLPLTL

NPLIYSLRNKNMK

>OranORc24946.2P

LPHLIVVFLYLCTGSFAHLKPPSSSPSQLDLLVSVFYTVLPPTPEP/PPP

LNPLIYSLRNKDMKATLGKIFSVHFISRVN

>OranORc25205.1P

TSENSFEGFILLGFSDQPHLEALLFTCVLFFYLLSLLGNMAIMVVSHLDP

CLHMPLHFFLCHLSFLDLCFSTSLAPQTLVNLWGPIETITFGGCVVQLYV

SLALDSTECILLALMALDLYAAICQPLCYAMLMHHSLFRQLAAVSELCGV

ANSLVHTKLIMCLPQFGHRHLDHFIYEAPALL

>OranORc25289.1

MPIPNTTSFHPSMFLLLGIPGMEDQHIWISIPFCSMYIMALLGNGTILLV

VASDKTLHEPMYHFLCILSLTDLVLCSTTLPKMLAIFWFGAQPISFHGCL

TQMFFIHAAFATESGVLLAMAFDRFVAICRPLHYSSILSPGVIGKITVAC

ISRSLIVDLPFIILIRRLPFCGHQVISHTYCEHMGIAKLACVNIKINIIY

GLTVALLVTGVDVVLIGFSYGFILHAVLHLPTQDAQRKAFSTCGAHVCVI

LVFYIPAFFSFFTHRFGHWVPPQVHIFVANLYLLVPPVLNPLVYGINTKH

IRRRILTVILGNR*

>OranORc25804.1P

NTSTFNSFILTGFWGLEDSLHWIFILLGVLYCVAILGNSLILVILKEKQS

LHQPMYYFLAMLSVNDLGVSCSTLPTVLGTFCFNVREVAFNACMAQMFFI

HLLSFMESGILLAMSFDRYVAICNPLRYSLILTNARITKMGLAIFSRTFG

AVFPLPFLLKRLSFCKGNILSHAYCLHPDLIRLPCGDITINNIFGLFIVL

STFGMDSVLIFLSYVLILRSVLTIASQ/RGGRSKALNTCVSHNCAVLIFY

IPMVGVSM

>OranORc26072.1

MWLSPSRRPNPILLGLNRTSFHPASFLLQGIPGLEGLHLWLSIPTCLMYI

LALLGNSALLFLILLDRNLHTPMFLFLAMLAGADLALSTSTVPKTLSVLW

ALSQEISFHACLAQMFFVHVTFIAESTILLAMAFDRYMAICRPLHYPAIL

TTAVTGRIGLAALGRALCVMVPTIFLLERLPYCGHRRMPHTYCEHMGIAR

LACAPIGVNIWYGLTTTLLSPGLDVMLISTSYGLILHAVFQLPSRDAKLK

ALGTCGAHFCVILLFYLPALFSFFAHRFGQGIPLHVHVLLANLYVLLPPM

LNPIIYGVKTKPIWERMVRLLSCVSAAVRGS*

>OranORc26198.1P

NSPTVTEVILLGFLEGSWLQGTLFLTFLVLYGMTVVGNLGMVAIISLDPQ

LHTAMYSFLCSLSLLEVCYSSTITPRALLNFLSERAAISFPGCATQFFFL

SLFGTTEAFLLASMAYDRFIVICDPLHYSMIMSHGICHLLVGGLYLWGVV

NALTQTTMIF*LLFCGPNEIDGFFCDVLPILTPSCSDTLTNQLVLLGLGG

SIIVGTCSILSISYVLVLSTILRILSAEGRMRAFSTCVSHLVGMGLFYGS

VFFMFAQSVATSNMEQSKVVSIFYTVIIPVLNPIIYSLRNKDVKEALKRI

RKKLSL

>OranORc26287.1P

FLEVWELQLVHAALFLLVYLVSLVGNLLIVTVTALNQHHHTSMYFFLRNL

SFVDLCLISITVPKSIHNSLTHMRAISFLDCIAQIFLVILLVSVELFVLM

VMSYECYVVICHPLRYDVTMNRETCVKLATAS*LGGGIFGMMYTAGSFSL

LFCGSPIFLQFLCDASSLLMVSCSNTHVVFVFSLVSGFVCALFSFVSTVI

SYICIFWAVLRMPATEGWAKVFSTCLPHLVIITVFLSTASIAYLKPPPDS

PSAVELVVSVFYTTVPPALSPLIYSLRNRDVKAALEKVL

>OranORc26300.1P

EDMSNCTTVMEFLLLGFSEVRELQQVHATLFLLVYLAALMGNLLIFAVTI

LDRHSTPP/ALHTPMYFFLRNLSILDLCLISVTVPNSILNSLTERRPISF

LGCVLQVFFFISFASSKITLLTSMSYDRYVAICCPLHYEVIMNPRACGKM

AT/SWLRGGLSGIMHSATVSSLPFCRVNVVHQFFCGIPQLMRLSGSEGML

QEVGVCTSLTLLALICFAFIGVSYVHIFWAVLKMPSVEDRAKTFSTCLPH

LVVVTV/LTSSFEYLKPPSDFPSMPDLLVSVFYTVVPPALNPLFYSLRNR

DMKSALGRVAWG

>OranORc26480.1P

MANLTTLTGFLLMGFSEVRELQLVHAVLFLLVYLAALTGTLLIITTTALD

QCLHTPMYFFLRNLSFIDLCLISVTVSPS/KSIVTSLTDLHSIYYWGCVT

QVLCVIFFMGSEMCVLMVMSYDRYAAICRSLCYDIIMNRGACGKMVATSW

LSGGLFGVMYSAGTFTLSFCKSNTIQQFFCDIPSLLKISCSKTHLVIDVS

VAIGIGFGSFSIVAIITSYFRVFSILLMLPSTEGQAKAFSTCLPHLAVMN

VFVFTAGFAYL*PPSDSPSTLGLLVSVFYTVVPPNPEP/PPTLNPLIYSL

RNRDMKAAPRRI

>OranORc27258.1P

MSNCTTVMEFLLLGFSEVRELQQVHATLFLLVYLAALMGNLLIFAITILD

/LHTPMYFFLRNLSILDLCLISVTVPNSILNSLTDRRPISFLGCVLQVFF

FISFASSKITLLTSMSYDRYLAICCPLHYEVIMNPRACGKMATT/GLVGR

WPPPSWLRGGLSGIMHSATASSLPFCRVNVVHQFFCDIPQLMRLSGSEGM

LQEGGVCASLTLLALICFAFIGVSYVHIFWAVLKMPSVEDWAKAFSTCLP

HLVVVTV/YLKPPSDFPSMLDLLVSVFYTVVPPALNPLFYSLRNRDMKTA

LGRVAWGKL

>OranORc27658.1P

TPKPHVLFPSKFVLCRNLFDTVTVPKILAVQDFSTACMYRYLAICNPLCY

PLLMNRRVLIQLTGGLWLLGLFVAIVQTTWIFSFPFCGPNGIDYFFCDTP

PVLELVCADTSVLELYTLMGTIFLNVVPFLLILLSYICIISTILKMPSTE

GRKKAFFTCSSHLTVVTVFFGAAILTYLQPKASSSAESKKLLTLSYILLT

PVLNPLIYSLCNSEVIRTLQRTLCRET

>OranORc27899.1T

FVSTVISYIRIFWAVLRMPATEGWAKVFSTCLPHLVIITVFLSTASIAYL

KPPPDSPSAVELVVSVFYTTVPPALNPLIYSLRNRDVKAALEKVLE

>OranORc27952.1

MRNYTPVTEFILLGLTDNPRLQTVILFYMSVTYVMSITGNLSIVTLTLLD

SHLRTPMYFFLRYFSFLEICFTSTCIPRFLATLATGNRTISYNCCATQLF

FFFLLGATEFFLLAAMSYDRYVAICRPLHYTTVMSQRVCTLLMLSSLLAG

FLVIFPPIILGLQLDFCGSVAIDHFFCDVSPMLLLSCSDTWLLELMALVL

AVGTVLITLALVVMSYTAIARAILRLPSTQQRKKAFSTCSSHMVVVSITY

GSCIFMYIKPSAKDRVDLTKGVSLLYTSVAPMLNPFIYTLRNQQVKQAIR

DLVHRVQFFFRG*

>OranORc28039.1

MEKENDTMGTDFILLGLFNHTRAHQVLFALVMMTSITSLMGNTAIILLIH

RDLHLHTPMYFLLGQLSIMDVMLVFTTIPQMAGDFWYGRNSISLVGCAIQ

IFIFLTLEGGECFLLAAMAYDRFVAICCPLRYPVLMSPRLCLLLAVVSWL

LGATDGLVQAGITMSYRFCRSREVNHFFCEAPALVRLACDDTMVFESVMY

VCCVLMLLIPFSVILGSYGLILETILRMKSVEAKKKAFTTCSSHLSVVGL

FYGAAIYIYIRPSSYDSTDYDKVVSAFYTILTPVLNPLIYSLKNREVLGA

LKRGLTDCRFRNLRVEREAA*

>OranORc28077.1P

MEKLTTVTEFILLGFLGGSWLQGTLFLTFLVLYGMTVVGNLGMVAIISLD

PQLHTAMYSFLCSLSLLEVCYSSTIAPRALLNFLSERAAISFPGCATQFF

FLSLFGTTEAFLLASMAYDRFIVICDPLHYSMIMSHGICHLLVGGLYLWG

VVNALTQTTMIF*LLFCGPNKIDGFFCDVLPILTPSCSDTLTNQLVLLGL

GGSIIVGTFSIVSISYVLVLSTILRILSAEGQMRAFSTCVSHLVGMGLFY

GSVFFMYAQSGATSNMEQSKVVSIFYTVIIPGLNPIIYSLRNKDVKEALK

RIRKKLSL

>OranORc28149.1

MANNTIGQTLSSFFLIGIPGLEEFHHWLSIPVFLLYSLSLMGNCLIILII

RLESSLHQPMYFFLCMLALNDLALSSSPAPKMLSIFWLDDHDTGFDTCLV

QMYFIHTFSITESGLLVVMAFDRYVAICQPLRYTTILTNKLVVLMGLVAS

LRAAIMMFPCIVLIKHLTFCTKNIIQHTYCEHMAVVKLACSNAIINRIYG

ICVALSVVVLDTGLITMSYVRILQAVFRLSSSKARSKALGTCAAHVCAIL

TFYVPSLFSFLTHRIDTKVSPSIHIIFANIYLLVPPAVNPLVYGAKTKLI

RDWVIFTLFSNKETVSK*

>OranORc28222.1P

MGTRNQTSGSTFTLQSITNAEQQRLLFVLFLGLYLVTVVGNLLIVLTIRT

DSRLHSPMYFFLANLSLVDICFSCTTVPSLLGTLFTGHQDISYGGCLAQM

YFFIAFGITESCLLAAMAYDRYLAICNPLLYSVVMSPQRCVLLVVTCWLV

SHLHSLLHTLLMTQMTFCASREIPLFFCDVFPLLKISCSDPHINFLVVYT

EGAVIVNSALLMVLASYAHIVPAVLRVPSAHGKRKAFSTCSSHLAAVGLF

YGTVIWVYFQPSSSFSSERDTIATVIYTTITPLLNPFIYSLRNRDLHRAL

H

>OranORc28222.2T

LSPRLSALMVVTCWVLTAVHALVHTLLVARLSFCTNRVVPHFFCDLSTLL

HLSCSDASVNKLVIITVGGVVVVGPFLAVLISYAHIFCAVLRVPSVRGLR

RAISTCGSHLAVVSLFYGTVIGLYLSPSPSHSAERGSVVAVLYTVVTPLL

NPFIYSLRNRDLLQAL

>OranORc28366.1P

MANLTTLTGFLLAGFSEVRELQLVHAVLFLLVYLAALTGNLLIITTTALD

QRLHTPMYFFLRNLSFIDLCLISVTVSPSS/KSIVTSLTDLHSIYYWGCV

TQVLCVIFFMGSEMCVLMAMSYDRYAAICRSLCYDIIMNRGACGKMVATS

WLSGGLFGVMYSAGTFTLNFCKSNTIQQFFCDVPSLLKISCSKTHLVIDV

SVAIGIGFGSFSIVAIVTSYFRVFSILLMLPSTEGRAKVFSTCLPHLAVT

NVFVFTAGFAYLKPPSDSPSMLGLLVSVFYTVVPPTLNPLIYSLRNRDMK

AAPRR

>OranORc28379.1P

DPQIQLFLFLVFLLIYGIALVGNLLTMLVIQVDPHTST/PHLYTPMYFFL

SNPS/FLDVRFASSTVPRMLENFLSDQKSITFPECITQIVSLFILATVKI

YLLATMAYNCYRVSGQLLCYPGSLTI*LCVQLVRGAWLVGDINVVVNALL

VLRLDFCRPNQNLHFSCEFELPPLLQISCSNIFASEMGILSLGVLLGLVS

FLLTLISYIHVISILFQICSS*GHGKAFPTCSSHLITVLLFCGTVFFQYM

RPSSA/SHPLALDWVVSIQCSILTPMLNSMIYILKNWDMMRGLKKLLRK

>OranORc28452.1

MTNISTVTRFLLLGFSDVRELQLVHAILFLLVYLAALLGNLLIISITTLD

QHLHTPMYFFLKYLSFNDLCFISITVPKSIAISLTNNNSISFLGCASQVF

LVILFGSSEFFVLIAMSYDRYAAICLPLRYELIMDRLACVQMLAASWLSG

SLFGVLFSVSTFSLPFCGSNTVQQFFCDVPSLLKISCSEDHVVTDMSLVV

AAVVVVVGFICIIISYIRIFSAMLRIPSSEGRKKAFSTCLPHLTVVTFFL

STGLFASLKPPSVTSSGLDLIVSIFYIVIPPTLNPLIYSLRNRDMKAALR

RTLKGENTHWLLWDNLCVSLC*

>OranORc28507.1P

MEKLTTVTEVILLGFLEGSWLQGTLFLTFLVLYGMTVVGNLGMVAIISLD

PQLHTAMYSFLCSLSLLEVCYSSTIAPRALLNFLSERAAISFPGCATQFF

FLSLFGMTEAFLLASMAYDRFIVICDPLHYSMIMSHGICHLLVGGLYLWG

VVNALTQTTMIF*LLFCGPNEIDGFFCDVLPILTPSCSDTLTNQLVLLGL

GGSIIVGTFSILSISYVLVLSTILRILSAEGRMRAFSTCVSHLVGVGLFS

GSVFFMYAQSGATSNMEQSKVVSIFYTIIIPGLNPIIYSLRNKDVKEALK

RIRKKLSL

>OranORc28739.1P

MFNITTGMEFLLLGFSEVRELQLVHGMMFLLVYLVALMGNLLIVTVTALD

ERFHTPMYFSLSNLALIDLCLISVTVLKSVVNSLTNNRSVSFLGCILQVL

FFISLASTEMILLTVMSHDHYTAICHPLRYEVVMNRGACGKMAATSWLSG

GLSALMYMATTFSEPFCRSQVIHHFFCETPHLLAQAGSTVILREVKVTIF

TASLSFLCFISIIISYIRNLLHCAEDTIGRGPIQSLLNLPAH*SLSFFLL

CRAFAYL

>OranORc28745.1P

NDQPRLEEMALFGILLTLFGNISIIFLLTMDSLLGTSMHVFLPTSS/TYL

LHQQLHIA*SSDKTILNLGHATQLYFVLAFVGVECVMLAIMSCNCYAVMC

QPLCYPAIMLL*L*GHLEYLSLIEWADHCTSLCGHC*SGLFLCEMPVLIG

IARVNILSLVELAFMLVILIILS

>OranORc28859.1

MIMSLSNSSDALLDTFFLLGIPGLEAVHIWISIPFCSMYFFALLGNSALI

VIITTERSLHKPMYIFLSVLSATDLALSSTTVPKMLQILWFGARSISFSG

CLTQMFFVHSVFALESFILLAMAFDRYMAICHPLRYASVLTPQAIGKTGI

MGIVRSMAVVSPFIFLLKRLPYCGHRLIPHTYCEHMGIARLACSDITVNV

VYGLTVALLAMGLDAVLIAASYVLILCTVFKLPSHKARRKALNTFGSHLC

VILIFYIPAFFSFLTHRFGRHIPHHVHIFLANLYVLVPPMLNPAVYGVNT

KEIRKQVLKIFLLRRGEF*

>OranORc28987.1T

QVLFFISLASTEMILLTVMSHDRYTAICHPLRYEVVMNRGACGKMVAASW

LSGGLSTLMHTATTFSKPFCGSHVIHHFFCEAPHLLAQAGSTVILREVKV

TIFTASLSFLCFISIILSYIRIFSIVLKIPSVEGRSKAFSTCLPHLVVVI

LFLSAGAFAYLKSKSNGPSGVDLFLSMFYSIVPPAMNPIIYSLRNRELKV

GLRRMLCGTVSNSLLFH

>OranORc28987.2P

SGNLLIVAVTTLNWRLHTPKYFFLRHLSNLDLCYISTTFPKSIVNSLTDR

REISFLGCVFQVFVFISLASTEMALLTVMSFNRYVAICLPLRYEFVISRG

ACGKMAASSWFSGSLSGLMQTAVTFSLPFRGSNEIHQFFCDIPQLLKLSG

SDWIMAEMDISALIACLAFICFVPMIISYVCIFSAVLRLRSSEGQNKAFS

ICLPHLVVVAFFFSTGSFAYLKPPSDSPSFQDLLVSVFYTGVPPTLNPLI

YSLRNKDMKAALKK

>OranORc29124.1P

ANSMAMIEFLILRFSEVWELQLVQATLFLL/YPVALTGNLLIVTVTALDW

RLHTYMYFFFRHLSILDLCYIYVTTPKSILNSLTDNRSISFLGCAFQVFA

FILCSCAETPLLTAMSFDHFAAICHPLHYEVIMDDGTWVKMAAASWFFGT

LSTMVHTVATFSALIWWSNDLPRFFCDIPQLIRLTGSDRNLQEFMAKTSS

AGLTFGYFLAIVVTYMCIFWAVLKMLTAEVQAKAFSTCLPYFTVMTLFI/

HLKPVTDSPSAVDLLMSVFYTVVPPTLNLVIYSLRNQDMKATLRRMI

>OranORc29148.1

MPPTNSSSTTISDFLLNCFVESGSDHLGFSMVLALLFIVALAANAVLLLT

IWLEQSLHQPVYYLLSLLSLLDLVLCLTVIPKVLAIFWWGWRSISFVGCF

LQMFVMNSFLAMESCTFMVMAYDRYVAICHPLRYPSIVTDRFVAKAAFFI

VARNTLISFPVPILSAWLHYCRTNVIEHCICANLHVSRLSCSDITLTRLY

QFIAAWTLLGLDLVLITLSYAFILRVVLRLKAEGAAAKTLSTCGSHLILI

LFFSTILLVLVLTNVVKKRVHANIPVLLNVLHNVIPAALNPIVYGVRTKE

IKEGILRVLRKMRG*

>OranORc29349.1

MANGMEVTEFLLLGFSEVQELQLVHATLFLLIYLVALTGNLLIVTVTTLN

RHLHTPRFFFLRHLSVLDLCLISVTVPKSFFNSVTNNSISFQGCVLQVFF

FVSAVVSEVALLTVMSYDCYAAICHPLHYEVIMNRGVCGKMAAASWLSGG

LSGLMHMATTFSEPFSGPNVIHQFFCDVPQLLKLSGPQGNISEYSLIVLS

ASSFAICFAYITVSYIRIFLAVLKMPSAEGRSKAFSICLPHLIVMTFFIS

TGVSEYLIPSFNCPTGLDLLLSVFYSMVPPALNPVIYSLRNQAVKAALRR

MLCLE*

>OranORc29644.1P

QPLAYPAIMSKRFCLL/LMVVDSYLGSLLTAFVFTSNLFRLSFCGPNVIN

HFFCDFPPLLKLSCSDTTLAKIIPSSFATLLILSTLAIIVVSYLYIMLAI

LRIKSSAGRGKAFSTCASHLTAVSLFYGSSAFIYMLPKSKYSMDQKKVVS

MFYMVVIPMLNPLIYTLRNKEVKEALKRTLGIKGIYYGQF

>OranORc29644.2T

MKGNQSRVTEFILLGLTDDPEVQTVLFVVFLMIYSFTLLGNLAITLVIKI

NSHLHTPMYLLLSHLSLSDIGFPLFFHALTPKMLVNLL

>OranORc29665.1P

VIEFLILRFSEVWELQLVQATLFLL/YPVALTGNLLIVTITALDWRLHTY

MYFFFRHLSILDLCYINVTTP/PKSILNSLNDSRSISFLGCAFQVFAFML

CSCALTPLLTAMSFDHFAAICHPLHYEVIMDDGAWVKMVAAFCFFGTLST

MMHTVATFSALIWWSNDLPRFFCDIPQLIRLTGPDRNLQEFVAKTSSAGL

TFGYFLAIVVTYMCIFWAVLKMLTAEGQAKAFSTCLPYFAVVTLFIANSP

FPTSSP/FPHLKPVTDSPSAVDLLMSVFYTVVPPTLNLLIYSLRNQDMKA

TLRRMIA

>OranORc30093.1P

MFNITTGKEFLLLGFSEVQELQLVHGMMFLLVYLAALMGNLLIVTVLDER

LHTPIYFFLSNLALIDLCLISVTVPKSVVNSLTNNRSIPFLGCALQVLFF

ISLASTEMILFTVITHDRYAAICHPLRYEVVMNRGACGKIAAAS*LSGGL

STLIHTATTFSEPFCGSHVIHHFFCEAPHLLA*AGSTVILREVKVTIFTA

SLSFLCFISIIISYICIFSIVLKIPSVEGRSTAFSTCLPHLVIIALFLST

GTFAYLNSKSNGPSRMDLFLSMFYSIVALAMNPIIYSLRNRELKASLERM

LCGTVSNSLLFH

>OranORc30118.1

MEKGNQTSISEFLLLGLSDRAEQWQLLFVLFLWMYLLGVLGSLLIVVAIG

SDPHLHTPMYFFLTNLSLADICFLSTTVPKMLINIQARDKSISYVGCLAQ

MYCFMLFSCLDNFLLTGMAFDRYVAICHPLHYTTIMSPRLCAWMIVVSWS

IGNLNALLHTLMVVRLSFCAGNKIHHFFCDLSQLLKLSCTDTLFNEVLVY

VLLVALGVVPLTGLLFSYSCIISTILRIPSPGGRYKVFSTCSSHLSVVSL

FYGTGLGVYFSQTSSTQTSGKGSVASVMYTVVSPMLNPFIYSLRNKDMKE

ALRNVFRRKSRPKKL*

>OranORc30455.1P

MANVTTVREYLLLGFSEVWELQLIHLLVYLVALTGNLLIFVVTAFNRRLH

TPMYFFLRHLSVLDRSLISVTVPKSISNAVINSRSISSARCGLQVLFFTF

CACSEVGILMAMSYNRCVAICWPPCYDDIMALGACGKMALASWLSGQLMG

ILHVSNIFSLPFCGPAIIHHFFCDVPQVLKLVCPGDTMGVVGVICLIVTV

AFFSLILIIYSYVSIFWALLRMPSTEGRPKAFSTCLPHLVVITLFLSSGG

FEYLNPVPDSPSLPDFLLPVFYAIVPPSLNPVIYSLRNRDIKSALG

>OranORc30914.1P

ISFCHQEVFVLKATLFMVFRICLLAIPLPLLAARLPYCSRSEIDHCQLFN

LAIASLACDDRKANNIFQFNMV*IIMGSDVGLIILYYALILRAVLKLHSA

KATSKALNTCSSHSILILFYMVIVVMSITHTAKEEVTRIPILLKLLHDVI

PLALNPIVYSLRTQDIKVGILKPIRLAGERK*CWVLCGCK

>OranORc31044.1P

MANRTVVMEFLLLGFSEVRELQLIHATLFLLVYLAALVGNLFIVTITAFD

RRLHTPMYFFLRHLSVLDLCYISVTIPKSIFNSLTNLNRGACETMAATSW

LSGALSAILHTSATFSVPLGGSNVIHQFFCEIPQVIRLSDSSGKIWELIA

TTFSASLTLICFVSIVVSYVHIFVAVLRMPSGESRSKAFSTCLPHLIVVT

*FVSTGDAAYLKPVTDSPSVLDLPVSMFYTVVLPTLNPLIYSLRNRDMKA

AMLRMLGGK

>OranORc31170.1T

DRYEAICHPLHYSLIMSPQLCVFLVAGAWFITFLHALLHTVLTAQLSFCG

DNKIPHFYCDLTALLELSCSDTTTNEVVIFTVAGLLGIAPPMCILASYAC

IVSALLKLPSARSKRRAFSTCSSHFTVVTLFYGAGLGVHFHPTSSHSKYE

DLVASVMYTVVTPMLNPLIYSLRNEDMKGALRKL

>OranORc31325.1P

MSSLSNNSFTQPSEFHLMCLPGAQSHQLGLSLVLALLFIVALGANTLLLL

TVRLEESLHQPVYYLLSLLSLLDLVLCLTVIPKVLAIFWLGWWSISFAGC

FLQMLIVNSFLAMESCTFLVMAYDRYVAICHPLRYPSIVTDHFVAKAAIF

IVARNFLLLLPIPILSARLHYCGNTTIRNCLCGNLSVSQLSL*KYYPQPT

PTSFVVGLGPLLGLRPXXXXSVSQLSCENITLNQLYQFVVGWTLLGSDLI

LIALSYAFILRAVLRLKAEGAAAKALSTCGSHLILILFFSTVLLVLVLTN

VVKKRVHADIPVLLNVLHYVIPAALNPIVYGVRTKDIQEGIHRVLRKARG

>OranORc31659.1P

VIEFLILRFSEVWELQLVQATLFLL/YPVALTGNLLIVTVTALDWRLHTY

MYFFFRHLSILDLCYINVTTPKSILNSLTDSHSISFLGCAFQVFAFILCS

CALTPLLTAMSFDHFAAICHPLHYEVILDDGAWVKMAAASWFFGTLSTMT

HTVTTFSTLIWWSNDLPRFFCDIPQLIRLTGPYRNLQEFVAKTSSAGLTF

GYFLAIVVTYMCIFWAVLKMLTAEGQAKAFSTCLPYFAVVTLFIA/HLKP

VTDSPSAVDLLMPVFYTVVPPTLNLLIYSLRNQDMKATLRRMI

>OranORc31659.2P

VIEFLILRFSEVWELQLVQATLFLL/YPVALTGNLLIVTVTALDWCLHTY

MYFLFRHLSILDLCYINVTTPKSILNSLTDSHSISFLGCAFQVFAFIRCS

CALTPLLTAMSFDHFAAICHPLHYEVIMDDGAWVKMSAASWFFGTLSTMT

HTVATFSTLIWWSNDLPRFFCDIPQLIRLTGPDRNLQEFVARTSSAGLTF

GYFLAIVVTYMCIFWAVLKMLTAEGQTKAFSTCLPYFAVVTLFIA/HLKP

VTDSPSAVDLLMPVFYTVVPPTLNLLIYSLRNQDMKATLRRMI

>OranORc31797.1P

KLM*PGEARAKVCDLAVTVTWVLGCFVSILISYVDIFWATLRMRSTEGQA

KASSTCLPHLAVISLYLFMGLFAHLKLPLSSQ*ALDLLVSVFYTVLPLTL

NLLIYSLRNKNMK

>OranORc31797.2P

MANVSTVTEFLLLGFSEVWELQLVHAALFLLVYLVALTGNLLILAIPTLD

QRLHTPMYFFLRNLSLLDLCYITTTVPKSILNSLTNSRSISFLGCTTQVL

LMILFGGSEFFILTAMSYDCYAAICCPLCYDTIMDQEVCRKMATASWLSG

CLYSLMHRAATFSSHFCGPRIIHQFFCDVPQLLKLMCPGEARAEICALVL

SVILCLGCFVSILVSYVHIFLAVLKMPAT*GRTKAFSTCLPHLIVVFLYL

CTGSFAHLKPPSSSPSKLGLLVSVFYTVLPPTLNPLIYSLRNKDMKAALG

KIFSVHFISRVN

>OranORc32114.1

MRNHTQVTEFILLGLSDDPDWLTVILLYMSVTYALSITGNLTIVTLALLD

YCLHTPMYFFLCCFSFLEICFTSACIPRFLATIITGDRMISYNCCATQLF

FLILLGVSEFFLLAAMSCDRYIAIYRPLHYTTIMSQKVCTLLVLSSCLTG

FLVSFPSVILGQQLDFCGSVAIDHFFCDVSPPLLLSCSDTMFLELMAFIL

AVGTLLVTLALVAVSYTAITCTILRLPSAQQKRKAFSTCSSHMVVVSITY

GSCIFMYINPSTKDRVDLNKGVAVLYTFVVPMLNPFIYTLWNQQVKQAIR

DLVHRVEFSTKG*

>OranORc32123.1P

LDFIVSVVLLISSLAMTIIS/YVCVISTILKIPQARGERR/PSGKGRKKA

FSTCASHFTVVSMGYGISIFVYVQPTQKRSLQLNKILFVLSSVVTPLLNP

FIFSLRNETMKRALSESLGRVQSFTKDLRVIPVMNSNPCRSWS

>OranORc32198.1

MWVLTSLTVMHNRTIDSEFFLLGLTDVQGLQTFLFALLLLVYLINLAGNG

TIMMIVASEPRLHSPMYFFLGNLSCLDICFSTVILPKLLANLLSARKAIS

FLGCIAQLHFFHFLGSAEAALLAVMALDRFVAICRPLRYPVIMSRRVCVL

LAGATWTAGFFHALLHSVLTSRLSFCGSRRVPHFFCDVKPLLALACDSIE

LHLWLLNTVTGSLVLGSFFLTLLSYLYLIGFLLLQSRSWGVLSKALSTCA

AHLSVVILFYVPVTVAYTSTTLDGSTVQDRAITIMYSIATPLFNPLIYTL

RNRDVKESLKMVILRKLCPVRF*

>OranORc32198.2T

ENQTAPTEFILLGFSNLLGLQVLFFVIFLTTYFYTLVGNGFILWVTLLDA

RLHAPMYFFLRNLAFLDLCYTTTIVPQMLVHLLAERKNISFARCATQQFA

FIFFVGVECLLLAGMAFDRYLAICQPLRYSVVMERGLSIQLAAASWAGGL

LNSMVHTALTFRLPFCGNNLIDYFFCDILPLLLLSCGDTSVNEVVLLSVG

VLIGWTPFM

>OranORc32282.1

MLAINWTSLHPFTFIMLGIPGLEAAHIWISIPFSLVYLMALLGNCIFLCL

IRTDPKLHQPMYLFLSMLSAVDLMLSTSALPKLLSLFWFNDREISFEACL

TQMYFIHSLSIMESGFFLAMAFDRYVAICHPLRHSAILTHSVVRGLGLAV

TFRGVLLLSPYPFLLRWLPYCRTNVIAHTYCEFMVIVKLACAETRGIRAY

GLMVAFFTGGLDFLLIVCSYVLILRAVFRLPSKGARLKTLGTCSSHICVV

LLFYFPCFFSFLTHRFGHQIPHHIHVFVANIYLLIPAMMNPIIYGARTKC

IRERVIKVLIQ*

>OranORc32298.1

MLFASFPTAWQLVPESLPGGNLTAVTEFVLLGFSVRPGLRIPLFLLLLAI

YLATLTGNSLIILVTAADAALRSPMYFFLRNLSFLEIGFNLVIVPKMLQT

LLAEDEPISFRGCAAQLFFFFFFGTAECFLLAAMALDRYVAICDPLRYPA

VMAREPCARLAAVSWLAGLPVATVQTSWIFSFPFCGPNRVDHFFCDSPPV

LKLACADTSLFELEAYVATVLVVLAPCLLVLGSYARIAATVIRMSSTKGK

RKAFSTCSSHLLVVSLYYGSAGVTYFRIKAGTSPASRKLLSLSYTVVTPM

LNPIIYSLRNVQVKAAVRRTLLRA*

>OranORc32298.2P

PCATQPSWRGGLRPAGGRLLAGRAPRGHRADSWIFSFPFCGPNRVDHFFC

DSPPVLKLACADTSLFELEALTATVLFILFPFVLILGSYIRIIAAVVRSR

SERHRAVSTCSSHLLVVSLFYSTAILTYSRPRADASPGSRKLLSLSYTVV

TPVLKPIIYSLRNGEVKAAVFRALRRA

>OranORc32320.1P

MANVTTVREYLLLGFSEVRELQLVHLLVYLVALTGNLLIFVVTVFNRRLH

TPMYFFLRHLSILDLCLISVTVPKSISNSVINSR

>OranORc32651.1

MANVTIVTAFLLLGFSEVQELQLVHAMLFLLVYLAALVGNLLIVTITNLG

WRLHTPMYFFLRHLSVLDLCLISITVPKSIHNSLINNRSISFLGCVLQVF

FALSSVFTELVILTVMPYNRYVAISHPLHYEVVMNRGACVKMTTASWLSW

VLSAELHTASTFSFSFFGSNVVGQFFCDIPQLLTISYSPDLLSEVVPICV

NVAFDFCCFIGIIVSYIHIMATVLRMPATEGWNKTFSTCLPHLIIITISL

STGFFAYLKAPLDSPSAMDLLLSMFYTVVPPTLKPLIYSLRNRDLKVTMG

KFLKGKFCTREKSLHISHLA*

>OranORc32688.1P

MFNITTGTEFLLLGFSEVRELQLVHGMMFLLVYLAALMGNLLIVTVTALD

ERLHTPMYFFLSNPALIDLCLISVTVPKSVVNSLTNNRSISFLGCVLQVL

FFISLASTEMILLTVMSHDRYTAICHPLRYEVVMNRGACGKMVAACWRSG

GVCTLMHAAATVSKPFCGSHVIRHVFCEASHRVSQAGSTAVLREGKSTIC

TASLSF/LCFVSIMLSYMRIFSIVLKIPSVEGPIRSLLHLLP

>OranORc32751.1P

SFFNSVTNNNSISFQGCILQVFFFVSAVVSEVALLTVMSYDRYAAICYPL

RYEVIMNRGVCGKMAAASWLSGGLSGLMHTATTFSEPFSGPNVIHQFFCD

VPQLLKLSGPQGNISEYSLIVLSASSFAICFAYITVSYIRIFSAVLKMPS

AEGRSKAFSTCLPHLIVVTFFISTGVSEYLIPSFNCPTGLDLLLSIFYSM

VPPALNPVIYSLRNQAVKAALRRMLCLE

>OranORc32889.1

MANVTTVTAFLLLGFSEVQELQLVHAMLFLLVYLAALVGNLLIVTITNLG

WRLHTPMYFFLRHLSVLDLCLISITVPKSIHNSLINDRSIFFLGCVLQVF

FALSSVFTELVILTVMSYNRYVAISHPLHYEVVMNRGACVKMTTTSWLSW

VLSAALHTASTFSFSFCGSNVVGQFFCDIPQLLTISCSPDLLSEVVPICV

NVAFDFCCFIGIIVSYIHIMATVLRMPATEGWNKAFSTCLPHLIIITISL

STGFFAYLKAPLDSPSVMDLLLSMFYTVVPPTLKPLIYSLRNRDLKVTMG

KFLKGKFCTREKSLHISNLA*

>OranORc33027.1T

NQSYVTEFILLGFSNLHEFQVILFMVFLVIYLIALIGNSLLVLVSSMDPA

LQTPMYFFLKSLSLMDIGYTTVIIPKMLTNFLSKNQNISFGGCAAQMCFS

FFFGPAECLILTTMAYDRHAAICDPLHYSLIMNRRFCLQLALASWLSGIP

VATVQTTMMFTLPFCGPNLINHFFCDGPPLLELVCTETFAFEVYSLTATV

IVLMFPFGVIIVSYVHILITILKMSSAEGRRKAFSTCSSHLIVVSLFFGA

ASLTYFRVKSSYSPESKKLLSLSYTVFTPMLNPLIYSLRNQEVKGALKKI

LGKK

>OranORc33196.1P

LRN*TSVTEFILPGLTDDPSF*VYFFSFSLLAYLLTITGNLTIITLILL/

LHTAM*FFLCNFSLLEISVCIATSFVSILTGNKIISYNCCTNQIFFYILL

GVSEFWLACPVNVTWPFADLYTTQPS*PPESVLCSSSAATC*ASCPPSPS

RPLCPAVEFL*HQ*HQSLQV*HCPLAADRLLEHTIPGGADILFCHDQAAI

TLSLVILSYVYIIKTILKTPSTQQRKKAFPTCSSHMVVVTLSCGSCIFMY

INPSNREVTTTQMVAVLNTFVASMLNPFIYTMRNQKVKQAFTNMA*KVFS

SQKR

>OranORc33392.1P

MVGRRNSTNVIKFILLGFSEPPQLQGLLFGVFLLIYLMTLSWNLGLITLI

RTNFHLHTPMYFFLSHLSFVDVCYSSSVVPKMLADFFKERKTISLMGCTI

QCFVFVGMGGTECCLLAAMAYDRYVAICDPLRYQAAMTQTLCARMVVAAH

LGGFLTSLAETSSIFQLRFCGPNVIHHFFCDLPLLLDLSCSNSFISKVVN

WLMVFITGVTSGLIVLISYLYIITTVVKIRPVKGRSKAFSTCASHLTVVT

LFYGSGLFAY/LHPGASHSANQGEAASLFYGAVIPMLNX

>OranORc33543.1P

GFSKKMANLTVVRGFLLLGFSEVRELRLVQAALFLLVYLAALTGNLLVVA

VTVLDRRLRTPMYFFLGNLSVLDLCLVSVTVPQSVHNSLTDQRSINYWGC

VAQVFLVVLFAASELFLLTAMSYDRFAAICHPLRYGVIVDSGACVKMVAA

SWLGGGLFGAMYSAGTFSLLYCVLREVRHVFCHVRSPLTIS

>OranORc33543.2P

ICYWGCVAQVFLVVPFAGSELLVLTAMSYDRYAAICRPLRYGVLVDRGAC

VKMAAASWLGAGPFGAMYTAGTFSLGFCGLHEVRQFSCDVRSLPKASRSG

NHVVVDVGEAAGAVLGFVSFVSIVVSYARIFGAVLRMPAAEGRAGAFSTC

LPRLAVVTLFVVTFFCAYLKPPSGSPSVPDLPVSVLYAVVPPALNPLIYS

LRNRDVKAALGRVLGGPPF

>OranORc33543.3T

MANLTVVTGFLLLGFSEVRELRPVQAAPFLPVYPAAPTGNLLVVAVTALD

RRLRAPVYFFLGHLSFLDLCLVSVTVPKSVLDALTDQX

>OranORc33543.4T

GFVSFVSIVVSYARIFGAVLRMPAAEGRAGAFSTCLPRLAVVTLFVVTFF

CAYLKPPSGSPSVPDLPVSVLYAVVPPALNPLIYSLRNRDVKAALGRVLG

GLPF

>OranORc33556.1T

CGLQVLFFTFCACLEVGILMAMSYDRCVTICWPPCYDVIMAPGACGKMAL

ASWLSGQLMGILHVSNIFSLPFCGPAMIHHFFCDVPHVLKLVCPGDTTGE

VGVICLIDTVAFFSLILIIYSYVSIFWALLRMPSTEGWPKAFSTCLPHLV

VVTLFLSSGGFEYLNPVPDSPSLPDFLLPVFYAIVPPSLNPVIYSLRNRD

IKSALG

>OranORc33754.1

MGDPNQSSLYPASFTLLGIPGLEEAHTWIAFLICLMYVTALAGNSIMLLV

IGREPGLHSPMFFFICFLSTTDIFLSSATIPKMLAIMWLQAREISFDSCI

AQMVFIHLFTGVESGILVAMAFDRYMAICRPLRYRVILTDRLVVKLGLTA

LLRPLLIVLPFILLIKRLHFCKATILPHSYCEHMGIAKLACSDIRVNVVY

GLTGALLVFSVDLVLIGLSYSLILRAVLRLPTKEARVKAFSTCGSHVSVM

MVFYTPALFSFLTHRFGHHVPRFVHILLANFYVVLPPMLNPIIYGVKTKQ

IRESILRTFQKKRSN*

>OranORc33762.1P

MGRGHQTSVSEFLLLGLSNWVE**QLLFMLVLRCTCSGSCGA/VYLLWVL

WSLLIVLAVNSDPHLHTSSYFFLTNLSLADACFLSTTVPIMLVNIQTRIN

S/QDKLISYSGCLAQMIFFFL

>OranORc33824.1T

FPNSSSFTPTSFTLNGIPSLEALHAWLSFPFCSMYVIAMVGNCGLLYLIC

REEALHQPMYYFLAMLSSTDLAMCSSTVPRMLLLFWFNLREIDFSTCLVQ

MFFVHTFTGMESGVLMLMALDRYVAICYPLRYSTILTNPVIAKAGLATFL

RGAFLIIPFTILTKRLPFCQNHIIPHTYCDHMSVAKLSCGDIKVNTVYGL

MVAILIGGFDIFCISASYT

>OranORc33908.1P

MNNHTSVTGFLLLGFSEVRELQLVHTALFLLVYLAVLTGNLLVVTITTLD

RHLHTPMYFFLRNLSILDLCLIPITVPKSILTLCPKTDPSPSWAVSSRSC

**FCLLPQRCLSSR*CPMTATWPSAAPCATRSSWVEGPV*RWWSPHGFSG

GLLGVLFSTGVSSLPFCDSHEVQQLFCDIHSLLKISCSEKHVGPKT*ALA

MGAHLRFSS

>OranORc34719.1

MGNGTGVTEFILMGLTDDPHLQALLFLVLFLTYAVSITGNLTIVTLTLLD

SHLRTPMYFFLRSFSLLEIAFTSACIPRFLVTIATGDRTISFSNCFTQLF

FIIFLAVTEFFLLAAMSYDRYVAICRPLHYTTVMSGGVCSLLVLCSFFSS

YLIVFPPVVMIAQLDFCASNILNHFICDSSPMMGLSCADTRFLELMAFLL

SLGTLLITLVLMTASYAAIVRAILRLPSAQQRRKAFSTCSSHMVVVSITY

GSCIFMYIKPSAKDRVELTKGVAVLNTSVAPMLNPFIYTLRNEQVKQAFR

VLVHRIRFSSRK*

>OranORc34806.1P

MEKLTTVTEFILLGFLGG/GVPGGSWLQGTLFLTFLVLYGMTVVGNLGMV

AIISLDPQLHTAMYSFLCSLSLLEVCYSSTIAPRALLNFLSERAAISFPG

CATQFFFLSLFRTTEAFLLASMAYDRFIVICDPLHYSMIMSHGICHLLVG

GLYLWGVVNALTQTTMIF*LLFCGPNKIDGFFCDVLPILTPSCSDTLTNQ

LVLLGLGGSIIVGTFSIVSISYVLVLSTILRILSAEGQMRAFSTCVSHLV

GVGLFYGSVFFMYAQSGATSNMEQSKVVSIFYTVIIPGLNPIIYSLRNKD

VKEALKRIRKKLSL

>OranORc34827.1

MQRGNQTGVSEFLLLGLSSWAEQQQLLSVLFLWMYLLGILGSLFVILAIS

SDPQLHTPMYFFLANLSLVDVCFLSTTVPKILANLQSHSKSISYAGCLVQ

LYFFILFVCLDSFLLTGMAYDRYVAICHPLHYATIMSPRLCAQTIAVSWS

VGSLDALLNILMVVRLSFCAENGIHHFFCDLHQVLKLSCTDTFFNEVLAY

VLLVAFGVVPLTGLLFSYSHIISTILKIPSVGGRYKVFSTCGSHLSVVSL

FYGTGLGIYFSPRSNQASQQGATASVLYAVVTPMLNPFIYSLRNKDMQQA

LRNVFCKKTLLLKPVTCPPGS*

>OranORc34946.1P

MSNHTTVTEFLLLGFLEVWELQLVHATLFLLDYLVALMGNLLIITVTALD

RHLHTPMYFFLRNLSVLHLIIISATIPKSIINLLTDTKSISSGG

>OranORc35041.1

MENLTTVTEFILLGFRGGPWLQGTLFLTFLVLYGVTVAGNLGMVAIITLD

SQLHTAMYSFLCSLSLLEVCYSSTIAPRALLNFLSEQAAISFPGCATQFF

FLSLFGTTEAFLLVTMAYDRFIVICDPLHYSMIMSHGICHMLVGGLYLWG

VVNAMTQTTMSFRLLFCGPNEIDGFFCDVLPILTPSCSDTLTNQLVLLSL

GGSIIVGTFSILSISYVLVLSTILRILSAEGRMRAFSTCVSHLVGVGLFY

GSVFFMYAQSGATSNMEQSKVVSIFYTVIIPMLNPIIYSLRNKDVKEALK

RIRKKLSL*

>OranORc35500.1P

LDDFLPGL*WPYDRFMAICQPLGYVTAMSPRRCVLLVAACWVIAQFNSLL

HTVLLVQLSFCADHTIPHFFCDLAPLLLLSCSDTSINELMLMSVSGAVIL

IPLMCILVSYAHIISAILKMPSSGSKHKAFSTCGSHLAVVSLFYGTVIGV

YLCPSPPSSSDESSLAAVSYAVVTPLLNPFIYSLRNHDLHQALHTFFCKR

TPSIL

>OranORc35757.1

MANGTEVTEFLLLGFSEVQELQLVHATLFLLIYLAALTGNLLIIAVTTLD

RHLHTPMFFFLRHLSVLDLCLISVTVPKSFFNSVTNNNSISFQGCVLQVF

FFVSAVVSEVALLTVMSYDHYAAICHPLRYEVIMNRGVCGKMAAASWLSG

GLSGLMHTATTFSEPFSGPNVIHQFFCDVPQLLKLSGPQGNISEYSLTVL

SASSFAICFSYITVSYIRIFSVVLKMPSAEGRSKAFSTCLPHLIVVTFFI

STGVSEYLIPSSNCPTGLDLLLSVFYSMVPPALNPVIYSLRNQAVKAALR

RMLCLE*

>OranORc35889.1P

MLAMRNDSLGEHFTLMGFSDQPQLEVILFVVVLTSYLLTLVGNTIIIVVS

RLEPRVHTPLYFLLSHLSLVDL*FTTSIVPQLLWNLWGPSKAITVVGCAI

QLYVSLALGSAECVLLPIMAFDHYTAVCRPLHYRSIMHPRFCHALAAVAW

TSGLGNSVIQSTITLWLPHCGDRYLAHFIYEVPVLIKLACVDTKANEIQL

FMATLVLLLLPMSLIMLSYGFIARAVLRIKSSQAGRKPWALVG/WQKALG

TCGSHLLVVTLFFGMGLIIYIQPNSSFSKTSSKFLTLFYMVVTLTLNLII

YTLRNKDMLGAVRRLLWKDCRSTKT

>OranORc36098.1

MANGTEVTEFLLLGFSEVQELQLVHATLFLLIYLAALTGNLLIIAVTTLD

RHLHTPMFFFLRHLSVLDLCLISVTVPKSFFNSVTNNNSISFQGCVLQVF

FFVSAVVSEVVLLTVMSYDRHAAICHPLRYEVIMNRGVCGKMAAASWLSG

GLSGLMHTATTFSEPFSGPNVIHQFFCDVPQLLKLSGPQGNISEYSLTVL

SASSFAICFSYITVSYIRIFSAVLKMPSAEGRSKAFSTCLPHLIVVTFFI

STGVSEYLIPSFNCPTGLDLLLSVFYSMVPPALNPVIYSLRNQAVKAALG

RMLCLE*

>OranORc36534.1T

TFSILSISYVLVLSTILRILSAEGRMRAFSTCVSHLVGVGLFYGSVFFMF

AQSGATSNMEQSKVVSIFYTVIIPGLNPIIYSLRNKDVKEALKRIRKKLS

L

>OranORc36740.1P

F*GIPEFQGLVFGVFLVICWTLGLISLIRTDSHFHTPKYFFLGHLSFVDI

CCSSSVVPKTLSDFFKKAKTTSFKG

>OranORc38031.1

MAAFNSSNFRPFLLTGFLGLESTHHWISALFFMLYLIAILGNSTILVAIK

KERSLQQPMHLFLSLLAISDLGLCTTTLPTLLKLSWFDDREIGFDACLVQ

MFFIHVFSLIESGILLTMAFDRFVAISHPLRYRAILNQMTIAKIGIGIIL

RAVAVILPGPILIKRLKFCEANVLSHAYCLHPDIIKLACSDHRISSIYGL

MVVLVTFGVDSLLILLSYLKILATVFRLASAQEQRRALDTCVSHILAVLL

LYVPMLGVSIIHRFAKHIPPVIHTIMGYIYLLVPPVLNPIVYCIKTREIR

AHLLKLFSQK*

>OranORc38057.1T

MENQTTVKEFILLGLTIDPEFQDVLFLFLLLTYALSITGNLTIITLTLLD

PHLHTPMYFFLRNFSFLEISFTSVYVPKML

>OranORc38253.1P

IANVSMVTEFLLLGFSEVWELQLVHAALFLLVYLVALTGNLLILAIPTLN

QRLHTPMYFFLRNLSLLDLCYITTTVPKSILNSLTNSRSISFLGCTTQVL

/GVLHRSFLMILFGGSEF

>OranORc38524.1T

FFFSTSSFAYLKPPSDSPSFQDLLVSMFYTVVSPTVNPLIYSLRNKDMKA

ALKKIIGREKFT

>OranORc39241.1P

FLEVWELQLVHAVLFLLVYLVSLVGNLLIVTVTALNQHHHTPMYFFLRNL

SFVDLCLISITVPKSIHNSLTHMRAISFLDCIAQIFLVILLVGVELFILM

VMSYECYVVICHPLRYDVTMNRETCVKLATAS*LGGGIFGMMYTARSFSL

LFCGSPIFLQFLCDASSLLMVSCSNTHVVFAFSLVSGFVCALFSFVSTVI

SYIRIFWAVLRMPATEGWAKVFSTCLPHLVIITVFLSTASIAYLKPPPDS

PSAVELVVSVFYTTVPPALNPLIYSLRNRDVKAALEKVLEWDIHSL*KAL

WP

>OranORc39485.1P

MDGRNQTGVSGFILLGLSSDPGMQLLLFVVFLGLYLVTVLGNLLIVLAVG

SDPRLHSPMYFFLANLSLVDVGATSATIPRMLADLWTRSPTISYAGCLAQ

LYFFLLFTDLENFLLTVMAYDRFVAICRPLHYWTVMSLRRCGLLLATCWV

TTHLYALAHTVLVDRLPFCASRRIAHFYCDLNPILRLSCARPWANQAVL/

GVLISLPPLLILGSNSRYPGPRHEGTPSAGGKRMSFN

>OranORc39585.1P

MTNVTTVTAFLLLGFSEVQELQLVHAVLFLLVYLAALVGNLLIVTITILA

WCLHTPLYFFLRHLSVLDLCLISITVPKSIHNSLINGRSISFLGRVLQIF

FALSSVFTELVILTVMSYNPYVAISHPLHYEVVMNRGACVKMTTASWLSW

VLSAALHTVSTFSFSFCGSNVVGQFFCDIPQLLTISHSPDLLNEVVPICV

KVAFNFCCFIGIVVSYIHIMATVLRMPATEG*TKAFSTCLPHLIITISLS

TGFFAYLKAFFAYLKAPLDSPSVMDLLLSTFYTVVPPTLSPLIYNLRNRD

LKVTMGKFLKGKFCTREKSLHISHLA

>OranORc39721.1

MATPNGTYNNPLSFILLGIPGLESLQHWIGIPFCGMFTVAVLGNCTVLIV

IKRNPELHQPMYLLLAMLALNDLGICPTIVPKTLGIFWFNLREIQFNSCL

AQMFFVHVLAAFETGILVAMALDRYVAICHPLRYSSILTPNVLLGMGLLV

VLRGVFMILPFLLLIKARVNSFRSTVVPHTYCEHMAVLKLATDDTRVNRI

YGLAVVFIIFWFDSSFITTSYTLIFKAVFRLPGKEARLKAFNTCTAHIII

IMLTYTLALFSFLGHRYGHHLTPYVHILLANFYLLVPTVVNPIIYGAKTK

EIRVRVITMFSLRGTLSKM*

>OranORc40365.1

MENQTTVKEFILLGLTSDPEFQDVLFLFLLLTYALSITGNLTIITLTLLD

PHLHTPMYFFLRNFSFLEISFTSVFVPKMLVNIGTGDKTISFAGCFTQYF

FAILLGATEFYLLAAMSLDRYVAICRPLEYTTIMSRRICVLLVFCSWVSG

FLVVSGPHIMTSMLPFCGANVINHYCCDYTILLQLACSDTHVIEMMELVL

AVVTLILTLALVILSYAYILWTILRIPSVQQRKKAFSTCSSHLIVVALSY

GSCIFMYVNPSVKDAAAFNKGVAVLNTSVAPLLNPFIYALRNQQVRQAF*

>OranORc40891.1

MESENQTLVTEFLILGLTDNPQLQPFLFLLFLTIYFLTVLGNVTILVVTC

FDSRLRMPMYFFLSNLSFLDVCYTSVTVPRMLLNLLSQFKAISYTACISQ

LYFFIIFAGTECFLLTAMAYDRYLAVCKPLRYPALMDNRRLLLLATAAWG

MGIFNATSHISFTVRLVFCGPNEIDHFLCDIRELLKLACSSTRNNQLEIF

LVGGTLRITSFCLTLGSYVPIIITVLKIRSEESRKKTFSTCASHLTVVVL

YYGTLNFVYGRQTSGNTLYMSKMMSVMFSVEIPMLNPLIYTLTDQQGEGA

IGNFLVH*

>OranORc41147.1

MERGNQTSISEFLLLGLSDRAEQRQLLFVLFLWMYLLGVLGSLLIILAIG

SNLHLHTPMYFFLTNLSLADVFFLSTTVPKMLANIQTHNKSITYAGCLAQ

VYFFIFFGGLDMFLLTGMAYDRYVAICHPLHYTTIMSPRLCALMVSGSWS

VSSLEALLNTLLMLHLSFCADNKILHYFCELNQILKLSCTDTVINDVLLY

ILIVVLGVGPLAGLLFSYVHIISTILKISSAGGRWKAFSTCGSHLTVVSL

FYGSALGVYLSPMSTQVSRKGSIASVVYTVVTPMLNPFIYSLRNKDMKRG

LRNVFCRKNLFLQMS*

>OranORc42203.1T

FCDVQPLMKLACSDTHLANLLFVSNSGAICTSSFVVLMASYVVILYSLRS

KGAEGRRKALSTCSSHILVVTLFFGPCIFIYTRPQTTFSVDKSVSVFYTI

VTPLVNPLIYTLRTTEVKYAMGKLWNRKVIPGKLLGRF

>OranORc42508.1T

MDGANGTPLTEFILTGLPHPPKLRVFLLLFFLATYILTQLGNLLILLVVS

GTPQLRARPMYIFLGVLSVVDMGISTVVVPRILTTLTATGRAISYAGCAA

QLYGYHFLGSTQCFLYTIMAYDRYLAICHPLRYPAVMTRRACSGLAAGAW

AAGSFHGAIQASLTFRLPYCGPNEVDHFFCDIPAVLKLACADTAANELVT

LVDVGAVASGCFLLILLSYARIVRAVLAMRTASGRRRALS

>OranORc42667.1P

ANSMTMIEFLILRFSEVWELQLVQATLFLL/YLVALTGNLLIVTVTALDW

RLHTYMYFFFRHLSILDLCYIYVTTPKSILNSLTDNRSISFLGCAFQVFA

FILCSCAETPLLTAMSFDHFAAICHPLHYEVIMDDGTWVKMAAASWFFGT

LSIMAHTVATFSALIWWSNDLPRFFCDIPQLIRLTGSDRNLQEFMAKTSS

AGLTFGYFLAIVVTYLCIFWAVLKMLTAEVQAKAFSTCLPYFTVMTLFI/

HLKPVTDSPSAVDLLMSVFYTVVPPTLNLVIYSLRNQDMKATLRRMI

>OranORc42888.1P

LICFAFIGVSYVHIFWAVLKMPSVEDRAKTFSTCLPHLVVVTV/YLKPPS

DFPSMPDLLVSVFYTVVPPALNPLFYSLRNRDMKSALGRVAWGK

>OranORc42888.2P

MSNCTTVMEFLLLGFSEVRELQQVHATLFLLVYLAALMGNLLIFAVTILD

RHSTPP/LHTPMYFFLRNLSILDLCLISVTVPNSILNSLTDRRPISFLGC

VLQVFFFISFASSKITLLTSMSYDHYLANCCPLHYEVIMSPRLVG/PEAC

GRWPPPSWLRGGLSG

>OranORc43121.1P

VNEFLLMGFSDIWALQLIHALLLLATCLTALTGNLLIIMVLTFSQHLHTP

MYLLPEEPVFPRSLLCLHHCSQITFQL*PVINLFPSWSVLLKCFFWICLL

ALKSP*VMSYDRYVAICSPLCYEVIMH*TVYVQMVALSWFSGSLAAILHV

SSTFSLPFCGPNVIYQFFCDIPQVIRLACMDEILGETGVITLLAWVAFSA

FILIIYTYIHIFSIVLRMPSAESGSKAFFTFLSHLIVVTLFLSTSTFENV

KPHSNSPSGLDLLVSVFYTLNLCSTPTLNPLI*SLRNKDMKAALAKALRG

KFSLRIN

>OranORc43151.1P

MVENISDFGFPSLFLVGIPNLGRFHVWFGFPVCFIYILTLLGNCLLVLMI

KTESSLHQPMYFFLCMLALNDMAISSSTAPKMLSIFWWNVHGIDFDSCLT

QMYFIHTFSIIESALLLSMAFDRYIAICYPLRYSTI

>OranORc43682.1T

HAQGLVGRWPCSCSVGNSWGSFMSLISSRCPFCGPAMIHHFFCDVPQVLK

LVCPGDTMGVVGVICLIVTVAFFSFILIIYSYVSIFWALLRMPSTEGRPK

AFSTCLPHLVVVTLFLSSGGFEYLNPVPDSPSLPDFLLPVFYAIVPPSLN

RVIYSLRNRDIKFALG

>OranORc44480.1T

MSHGICHMLVGGLYLWGVVNTMTQTTMSFRLLFCGPNEIDGFFCDVLPIL

TPSCSDTLTNQLVLLSLGGSIIVGTFSILSISYVLVLSTILRILSAEGRM

RAFSTCVSHLVGVGLFYGSVFFMYTQSGATSNMEQSKVVSIFYTVIIPML

NPIIYSLRNKDVKEALKRIRKKLSL

>OranORc44528.1P

GFSDF*ELQLVHTTLFLLVYLEALKGIFLTNTTTVLKRHLHYP/TPMYFF

LKKLSFIDVCYIAITLPKYNISSLTRNSSIFFLGCLTQLLLIDLFAVSEL

FVLMAMFYDLYGSIYWPLHY

>OranORc44614.1P

MANLPGGTGFLLLGSSEVREVRPVQAAPFLPVYPAAPTGNLPAVAALDRR

LRAPVYFFLGHLSVLDLCLVTVAVPRSVHNSLTDRRSVSLPGRAAQVFSV

VRFAGSELLVLTATSYDRYAAICRPLSYGVVVGRGACGKMAAASWLGGGL

FGVLFSAGPFSLSFRSSAALHQFFCAIPSLVKASCSDEHAALDAIMTTGI

SLGVACLASVVVSYARIFGAVLRMPAAEGRAGAFSARLPRLAAPAVFFAA

ASSSHLKPPSLSPSTPDPPVSAFYAAVPPALNPLIYGLRNGDVQAALGRV

LR

>OranORc45874.1P

MANGTEVTEFLLLGFSEVQELQLVHATLFLLIYLAALTGNLLIIAVTTLD

RHLHTPMFFFLRHLSVLDLCLISVTVPKS/FNSVTNNNSISFQGCVLQVF

FFVSAVVSEVALLTVMSYDLYTAICHPLPYEVIMNRGVCGKMAAASWLSG

GLSGLMHTATTFSEPFSGPNVIHQFFCDVPQLLKLSGPQGNISEYSLTVL

SASSFAICFSYITISYIRIFSAVLKMPSAEGRSKAFSTCLPHLIVVTFFI

STDVSEYLIPSSNCPTGLDLLLSVFYSMVPPALNPVIYSLRNQAVKAALR

RMLCLE

>OranORc46090.1P

MSNCTTVMEFLLLGFSEVWELQQVHATLFLLVYLAALMGNLLIFAVTILD

RHSTPP/LHTPMYFFLRNLSILDLCLISVTVPNSILNSLTDRRPISFLGC

VLQVFFFISFASSKITLLTSMSYDRYVAICCPLHYEVIMNPRACGKMA/Q

GLVGRWPPPSWLRGGLSGIMHSATVSSLPFCRVNVVHQFFCDIPQLMRLS

GSEGMLQEVGVCASLTLLALICFAFISVSYVHIFWAVLKMPSVEDRAKTF

STCLPHLVVVTINKQL*VSEASLRLPLDAGPAGVRVLHSGAPCPEPPLL

>OranORc46148.1P

VSIANTTSFRLSTFLLLGIPGMEEQHIWISIPLCSTYITALLGSGTILLV

VTSGETLHEPMYLFLCILSFTDLVLCSTTLPKMLAIFWFWAQPISFHGCL

TQMFFIRAVFATESGVPLAVAFDRFVAICRPLHYSSILSPGVTGKITVAC

ISRSLVVVIPFIILIHRLPFCGHHVISRTHCEHTGLAKLARANIKINIIY

GLTVALWGMGVDTVLTDFSYGFIPHTVLHLPSQDAQLKAFSTCGSHVCVI

VVFYIRA/FSFLTHCFGHWVPPQVHVFVAILYLLLPPVLNPLVFGVNTQH

IRRRILTLILGKS

>OranORc46221.1

MSIPNTTSFHPSTFLLLGIPGMEDQHIWISIPFCSMYITALLGNGTILFV

VTSDKTLHEPMYLFLCILSLTDLVLCSTTLPKMLAIFWFGAQPISFHGCL

TQMFFIHAVFATESGVLLAMAFDRFVAICRPLHYSSILSPGVIGKITVAC

ISRSLTVVIPFIILIHKLPFCGHHVIPHTYCEHMGIAKLACANIKINIIY

GLTVALWVMGVDIVLIGFSYGFILHTVLHLPSQDAQFKAFSTCGSHVCVI

MVFYIPAFFSFFTHRFGHWVPPQVHIFVAILYLLLPPVLNPLVYGINTKH

IRRRILTLILGKS*

>OranORc46729.1P

MANFTTVTEFLLI*FSEIWELQLVHATLFLLVYLLALVGNLLIITATTFD

RCLHTPMYFFLKNLSFLDIGY

>OranORc47828.1P

MNPTNKSTPDGFILLGFSNQP/IYMLILTCYIFTLFDNPTIILFSCFEPP

LHSPIYFFLSNLSVLDHSFITNIVPQMLWNLHRPEKTIANQGCLVQLYIS

LGLDYTEHVLLTHLKITKITFLTNPKPLLHPNPLRPLRCLQ

>OranORc47955.1P

SVPIPKLLVGILGRDKTISYKACAIQLFFYILLGAMEFFLLASMSYDHYV

AICQPLHYLTIMSHSVCTLLVLCSWLVGFLIIFPGLILGLQLNIC/IELF

TCDTAPKLEILCIDMQLFEQMNFTFTVGTLLVTLALDTMSYTTIIHSIV

>OranORc49068.1T

MFNITTGTEFLLLGFSEVRELQLVHGMMFLLVYLAALMGNLLIVTVTALD

ERLHTPMYFFLSNLALIDLCLISVTVPKSVVNSLTNNRSISFLGCVLPSF

IF

>OranORc49242.1T

PKMLVNCLVGNKSISFSGCATQMLFFITFGTTECFLLAAMAYDRYVAISI

PLLYTAFMSPRIYVSLIIGSYIGGLLHGLLHTTATFSLSFCRFNEIRHFF

CDIPPLLTLSCSDTHINELLLFNFVSAIEVVTVLIVLVSYGYILAAILRI

RSAQGRRKAFSTCASHLAGVTIYHSTILAAYVRPSSSYALEHGMVVSLFY

TVLIPMMNPVIYSLRNKDVKDAFKKVLEKHFQNETYKKQ

>OranORc50168.1P

MSNHTTVMEFLLLGFLEVWELQLVHATLFLLDYLVALMGNLLIITVTALD

RHLHTPMYFFLRNLSVLHLIIISATIPKSIINLLTDTKAIS

>OranORc50461.1P

SIFILIGIPELESFHVWFSLLFLLSSCS/LLAQFLLLGNCTILLVITLEE

SLHAPMYFLLCMLAITDLIGSTTVLPKTLSIFWCDDKEIHIDACLLQMFL

VHSLSVMESTILVAMAFDRYVAICDPLRYNLVLTNSVIMKIGLAIVARGL

VLIAPLCCLAKRLPFCRINIIANTYCKHMELVKLACSGFLVSKVYGLIVA

LSVVGLDSIFIALSYFRIIQTVLRLSSPEARVKAFGTCRAHIAVILMGYI

PALFSFLSHRISHNVAPYIHVIIGNLYILVPPAFNPIMYGMKTKEI

>OranORc52129.1P

MSSLSNISFTQPSEFHLMCLPGP/PWAWSHQLGLSLVLALLFIMALGAST

LLLLTVRLEESLHQPVYYLLSLLSFLDLVLFLTVIPKVLAIFWLGWWSIS

FSGCF*QMLIINSFLAMESCTFLVMAYDRYVAICHPLRYPSIVSDRFVAR

AAVFIVARNFLLLLPIPILSARLH*CGNTTIRNCLCGNLSVSQLSCENIT

LNQLYQFVVGWTLLGSDLILIALSYAFVLRAVLRLKAEGAAAKALSTCGS

HLILILFFSTVLLVLVLTNVVKKRVHADIPFLFNVLHHIIPAALNPIVYG

VRTKDIQEGIHRVLRKVRG

>OranORc52379.1P

WFNLRGDKTLALAWSRCYLSTRSREMEKGGPHANGLVSFI*TINNRLREC

ARKTNPIIVRAGLATFFRGAILVIPFTILTKRLPYCHGHIIPHTYCDHMS

VAKVSCGNIKVNAVYGLMVAILIGGFDIVCISASYTAILKAVVILSSAEA

QHKAFSTCTAHISAIIITYVPAFFSFFTHRFGGHTIPHHVHIIIANLYLV

LPPMMMVNPVVYEVKTKQIREAVVRMLGGKRGNDP

>OranORc54963.1T

NHTQFHPSSFLLLGLPGLEAAHIWIGFPICAAYLISLVGNCTILLVIKTE

QSLHQPMFYFLAMLAAIDLGLSTATIPKMLGIFWLGLREISFGGCLTQMF

F

>OranORc55172.1

MENQTTVKEFILLGLTSDPEFQDVLFLFLLLTYALSITGNLTIITLTLLD

PHLHTPMYFFLRNLSFLEISFTSVFVPKMLVNISTGDKTISFAGCFTQYF

FAILLGATEFYLLAAMSLDRYVAICRTLEYTTIMSRRICVLLVFCSWVSG

FLVVSGPHIMTSMLPFCGANVINHYCCDYTILLQLACSDTHVIEMMELVL

AVVTLILTLALVILSYAYILWTILRIPSVQQRKKAFSTCSSHLIVVALSY

GSCIFMYVNPSVKDAAAFNKGVAVLNTSVAPLLNPFIYALRNQQVRQAFK

NVCRKISVF*

>OranORc56184.1P

NRNNVTEFVLLGLSSDRNLQIFCFGLFLSCYIAILLGNFLILITVRGSPL

FKQPMYFFLFHLSLMDVCYTSTVTPKLVRDLLSEKKTISFGDCMMQLFDM

HFFGSIEVFILVGMAYDRYVAICKPLCYMIVMNEQRCNTMVVICWGAGLL

HSMIQLLLAISLPFCGPNKIDHYFCDVYPLLELACTDTHLIGFLILANSG

IVVLISFIVLVFSYVTILVCLRNLSSEGRRK

>OranORc57520.1T

MEEANFTAVSEFIFLGLTQMQEMRVVLFLVFLLVYTTTLLGNGLIMITVT

CELRLHTPMYFLLRNLSVMDLCFSSVSVPKMLVDLLSESMAISFGGCMAQ

MFFFHFFGGGTVFFLSVMAYDRYVAISRPLHYVALMSPQVCVGLIVATWV

SGFVHSIVQLGLVVRLPFCGPNILDNFYCDVPPVLRLACTNTFVLEFLMI

>OranORc57534.1

MEGENQTRVSEFVLLGLWDSPEQQQQRLFFWLFLWMYLLGGAGNLLMILA

TGSDPLLHTPMYFFLSNLSFVDLCLTTTTVPKMLLNIQTQKKTISYAGCL

TQMYFFLLLLDLDNMILAVMAFDRYMAICHPLHYTSVMLPSLCGLLMAVL

WVVANLFSLLFTLLMAQLSFCGNNTIPHFFCDLSVLLKLSCSDTHIVENL

LLIVSGLLGVTPLMCILVSYSRIVATVMRIPSAKGKRKTFSTCGSHLTVV

ALFYCAGFGVFFTPPSSHSGGKDTAASVMYTVVTPMFNPFIYSLRNKDMK

GALQKLVCKNTALPHKP*

>OranORc57554.1P

MANRTVVTEFLLLGFSEVRELQLIHATLFLLVYLAAMAGNLFIVTITALD

RRLHTPMYFFLRHLSVLDLCYISVTVPKSIFNSLTNLN*GACEKMAATSW

LSGALSAILHTSATFSVPLGGSNVIHQFFCEIPQVIRLSDSSGKIWELIA

TTFSASLTLICFVSIVVSYVHIFVAVLRMPSRESRSKAFSTCLPHLIVVT

*FVSTGDAAYLKPVSDSPSVLDLPVSMFYTVVLPTLNPLIYSLRNRDMKA

AMLRMLGGK

>OranORc58797.1P

LALICFAFIGVSYVHIFWAVLKMPSVEDRAKTFSTCLPHLVVVTI/FEYL

KPPSDFPSMPDLLVSVFYTVVPPALNPLFYSLRNRDMKSALGRV

>OranORc59818.1P

MMVETSENSSEGFILLGFSDQPHLEAVLFMCVLFFYLLSLLGNMAIMVVS

QLDPRLHMPMYFFLCHLSFLDLCFSTSLVSQNPVNLRGPKETISFGDGVV

QLYVSLALGSTECILLALIQLYVSLALGSTECILLALMAPDLYGAVCQPL

CYAMLMHHSLFWQLAAVS*LCGVANSLVHTKIILCLPQFGHHHLDHFIYE

APALL/MEATISELVLFTLSMLEDIVPLAFILISYGFIARSILRVGSSEG

WRKAFSTCSSHLLVVSIFKGTIIFMHLHPGGNYSQDPGKFVSLFYT

>OranORc59881.1P

MFNITTGKEFLLLGFSEVQELQLVHGMMFLLVYLAALMGNLLIVTVLDER

LHTPIYFLLSNLALIDLCLISTTVPKSVVNSLTNNRSIPFLGCALQVLFF

ISLASTEMILFTVITHDRYAAICHPLRYEVVMNRGACGKIAAAS*LSGGL

STLIHTATTFSEPFCGSHVIHHFLCEAPHLLAQAGSTVIL*RSQSHDLHC

KPSPFFAFISIIIS

>OranORc59935.1

MQMEYMAEKNLSYVTEFVLSGISNHPEWQIPLFLVFLIIYLITAIGNLGI

ITITNIDSRLKAPMYFFLRHLAFINLGNSTAIAPKMLVNFLAGNKTISYY

ECATQIGCFSTLIIAEIFMLATMAYDRYMAICKPLLYRMIISRGTCTLLV

ILIYTYSFIEGVIVSTHVFSVSYCSSNTINHFYCDHFPLLALSCSDTHIP

ETIIFAFSGSNLIFTLTVVLVSYFFIILAIVRIRSSEGRWKAFSTCTSHL

TAVTVFYGTLFFMYLQPQGSHSLDTDKMASVFYTLVIPMLNPLIYSLKNK

EVKGALKRLLTNSYQSFKIRM*

>OranORc61046.1P

ENSFERVILLGFSDQSHLEAVLFMCVLFFYLLSLLGNMAIMVVSHLDPCL

YMPWHFFLCHLSFLDLC/STSLAPQTLVNLWGPKETITFGGCVVQLYVSL

ALGSTECILLALMALDPYAAVCQHLCYAMLMHHSLFRQLAAVS*LCGVAN

SLVHTKLIMCLPQFGHRHLDHFIYEAPALL/EVTINELVIFTLSILENIV

PPAFILVSYGFIAQSVLRVGSSEGWRKAFSTFSSHLLVVSIFDGTIIFMH

LHPGGNYSQDQGKFVSFFYTMVMPTLNP

>OranORc61662.1P

SGSDWIMAENGHFQPFIACLAFICFV/MIISYVCIFSAVLRLRSSEGQNK

AFSICLPHLVVVAFFFSTGSFAYLKPPSDSPSFQDLLVSVFYTGVSPTLN

PLIYSLRNKDMKAALKK

>OranORc62900.1T

HPLRYPVLMSPRACGRLVVFSFTSGFAVSAVKVYFIANATFCGSNVLNHF

FCDISPILKLACTDFSTAERVDFVLALAVLVLPLAATVLSYGSIASAVVR

IPSASGRRKSFSTCASHLSVVTVFYTAIIFMYVRPRAIDSRDSNKLVSAV

YTILTPVVNPLIYCLRNKEFKRAVK

>OranORc62950.1P

GGMMQTFGVHFLVARDLPLTAMATT*RGHLQISPLHEHHEQVCVQ*NARG

HVGWGFHPLHHSGGVGCPAAFCGPSVIDHYFCDVHPVLKLACANTHLAGV

VVTANSGTIALGSFLILLFSYAVILWSLRQQSEEGRRKALSTCGSHIAVV

IIFFGPCTFMYMRPDITFSEDKMVAIFYTIITPMLNPLIYTLRNAVVKNA

MKRLWSRRVALQE

>OranORc65882.1T

LTPSCSDTLTNQLVLLGLGGSIIVGTFSILSISYVLVLSTILRILSAEGR

MRAFSTCVSHLVGEGLFYGSVFFMYAQSGATSNMEQSKVVSIFYTVIIPM

LNPIIYSLRNKDVKEALKRIRKKLSL

>OranORc66517.1P

MAEGNFTSVSEFLILGLTDDPILRLIFFLIFLLVYSVTIMGNLCIIMLIR

VSSQLHTPMYLFLSHLAFIDIWYSTSVTPEMLKGFLVGRDTISYAGCTAQ

LCSAFAFGTSEGILLAMMAYDRYVAICNPLFYAISMSNRVCVQLITVSYV

GGCLNALVFTTFVFI*SSVEPDEIESIF

>OranORc67251.1P

NWRLHTPKYFFLRHLSILDLCYISTTVPKSIVNSLTDRREISFLGCVFQV

FVFISLASTKMALLTVMSFNRYVAICLPLRYEFVISRGACGKMAASSWFS

GSLSGLMQTAVTFSLPFRGSNEIHQFFCDIPQLLKLSGSDWIMAEMDISA

LIACLAFICFVPMIISYVCIFSAVLRLRSSEGQNKAFSICLPHLVVVAFF

FSTGSFAYLKPPSDSPSFQDLLVSVFYTGVSPTLNPLIYSLRNKDMKAAL

KK

>OranORc69505.1T

YLAALMGNLLIVTVTALDERLHTPMYFFLSNLALIDLCLISVTVPKSVVN

SLTNNRSISFLGCVLQVLFFISLASTEMILLTVMSHDRYTAICHPLRYEV

VMNRGACGKMAAASWLSGGLSGLMHMATTFSEPFCGSHVIHHFFCEAPHL

LAQAGSTVILREVKVTIFTASLSFLCFISIIISYIRIFSIVLKIPSVEGR

SKAFSTCLPHLVVVILFLSAGAFAYLKSKSNGPSGVDLFLSMFYSIVPPA

MNPIIYSLRNRELKVGLQRMLCGTVSNSLLFH

>OranORc70131.1P

LYFFLRPLSVLNLCLISVSVPKSILNALSNLQSISCLGCTSQVFSFMIFG

TADLAFLTAMSYDNYMTICRPLLYDIITDR/CGKMAAASWLIVGLYTLVH

MAATFSSHFCGPHVIHQFFCDVPQLLKLMFPREARAEVCILVVNVTFVLR

CFVSLLVPYVHIF

>OranORc70758.1T

TRFLLLGISELVPATLFLLIYLVALTGNLLIITFTAVYHHLHTPTYFFLR

HLSILNFCYISIPVPKCIYSTLTQSNFISFLDVQPKFTIVILLAAQSCF

>OranORc70800.1P

MANFTTVTEFLLM*FSEIWELQLVHATLFLLVYLLALAGNLLIITATTFD

RRLHTPMYFFLKNLSFLDIGIITIIIPKSIINSITHSNSISLLGCAAQVF

FFLCFAGSEVFLLMVMSYDRYITICHPLHYEVVMSRGACVQMVARAWITS

TVHATMHTASIFSLPFCGPKPLHQFFCDGPQLVKLACSDSYVAEKVSVAI

TICLVSTCFIFIIVSYVHIFSDVLRMPS/EGRSKAFSTCLPHLAVVTFFL

GAGCFDIAQPSQDAPSVTDLLVPMLYTVVPPSLNPIIYSLRKRDMKVALR

MVLR

>OranORc73112.1P

MFVPIRASNGSSPQVTEFILMGFPGIHSWQHWLSLPLALLYLSALLANAV

ILLIIWQKTVLHHPMFYFLAVLALVDMGLSTTIMPRILAMLWFNARTISL

QECFFQIYAIHVFVGLESGIFLCMAIDRYVTICHPLRYPSVITETLSSTP

P*FMV

>OranORc73751.1T

LGSNAQIISAILRMPSAGSKRIAFSTNSSHLAVVSLFYGTVISEYLCPSL

SGSSDESSLAAVLYAVVTLLLNPFIYSLRNHDLHRALCTFFCRKTPFILL

QTFQAPK

>OranORc74544.1P

VSFLSCISQVLSFVIFGAANRAFLTVMSYDYYVSTCRPQLYDIIINRRAC

GKMVTTSWLTSGLYTLMEMAATFSSHFCCPCVIHQFFCDVPQLLKLMFPG

EARPDVCTLVA*VTLAPGYFISILLSYVHIFLALLRM*SSEDRTKAFSIC

LLHLTVMTSFVAIGTCYPQPISDSLDLLVSVFYPWCP

>OranORc75414.1P

YDIIMDPWPYRKMAGASWLNGGLYTLMHMAITFSSHFCGPRVIHQFFSDM

*HPPVAETHVSGRSPGRGLCSGSVTLVPACFVFLLVSYGHIFWVGLRMQS

TEDWAKAFSTCLPHFTVMTLFI/SFAYLKLVSDFHSALDLLVSIPYTLRN

*DIKSTLRRM

>OranORc79239.1P

LYFFLRPLSVLNLCLISVSVPKSILNALSNLQSISCLGCTSQVFSFMIFG

TADLAFLTAMSYDNYMTICRPLLYDIITDR/CGKMAAASWLIVGLYTLVH

MAATFSSHFCGPHVIHQFFCDVPQLLKLMFPGEARAEVCILVVNVTFVLR

CFVSLLVPYVHIFCEPHVGQTDYPVSTPALRTVLGT**ALNKYQHYYLLG

MLRMQSTEGRAKAFSTCLPHFTIVTSFIVNGA/AHLKPVSDSPSTLDRMV

SMYYTLVPPTLNSLICSLRNGAMKATLRRMIA

>OranORc84976.1

MKKWNESSGTEFFLVGLFAHLSYPSLLVSALSIVYLLALAGNSLMIFLIW

MDSRLHTPMYFFLSQLSLMDLLLTSVVVPKVLIDYLLNTSMITPVGCGIQ

MFLVMTLGGGEGLLLGFMSYDRYVAVCHPLRYPLLMPQDKCQRLGVGACM

GAALVSLFNTILTMCLPYCRPRKVHHFLCEPPALLKLACVDLSVYEPAVF

VISSIVILIPFLLILASYAHILLAILQMPSTGGWRKVAITCSSHLLVVHL

YYGAAMLMYIRPSSQHSPGEDNILAVFYTVLTPMLNPLIYSLRNKEVTGA

MRKLFREFCA*

>OranORc85870.1T

MEEGNLTMWNGFILSGLTDRVDLQATLFLFLLLAYIVTVIGNLGMTMLIK

IEPRLHTPMYFFLSNLSFVDFCYSSCVTPRMLVTFLSETKAISFTACASQ

AYFFGAFGTTEIFLLAVMAYDRYVAICHPLLYTVIVSPRVCVFLA

>OranORc86056.1P

MFNITNGTEFLLLGFSEVRELQLVHGMMFLLVYLAALMGNLLIVTVTALD

ERLHTPMYFFLSNLALIDLCLISVTVPKSVVNSLTNNRSISFLGCVLQVL

FFISLAATEMILLTVMSHDRYTAICHPLRYEVVMNRGACGKMAAASWLSG

GLSGLMHMATTFSEPFCGSHVIHHFFCEAPHLLAQAGSTVILREVKVTIF

TASLSFLCFISIIISYIRIFSIVLKIPSVEGRSKAFSTCL/HLVIVVLFL

SAGAFAYLKSKSNGPSGVDLFLSMFYSIVPPAMNPIIYSLRNRELKVGLR

RMLCGTVSNSLLFH

>OranORc86859.1T

PNEINHFYCDFPPLLELPVRTPGVAQIWNSITSILIILVTILIIAISYLC

ILSAILRIPSTQGRHKAFSTCASHLTAVTLFYGTLTFIYTQPNSRYSMDQ

KKMVSVFYMVVIPMLNPLIYSLRNKDVKEALRRIMIFKRCPD

>OranORc89394.1P

FLEVWELQLVHAALFLLVYVVSLVGNLLIVTVTALNQHHHTPMYFFLRNL

SFVDLCLISITVPKSIHNSLTHMRAISFLDCIAQIFLVILLVGVELFILM

VMSYECYVVICHPLRYDVTMNRETCVKLATAS*LGGGIFGMMYTVGSFSL

LFCGSPIFLQFLCDASSLLMVSCSNTHVVFAFSLVSGFVCALFSFVSTVI

SYIRIFWAVLRMPATEGWAKVFSTCLPHLVIITVFLSTASIAYLKPPPDS

PSAVELVVSVFYTTVPPALNPLIYSLRQDVKLRWKSPGVGY

>OranORc89426.1P

MMRNQKTVIEFILLGLTDNPELQALLFLFLFATYVLSVAG/VSLDFTSFF

VPIMLVSIGTGNKTVSFDACFAQFFYTILLSVTEFFLL

>OranORc89470.1P

RIPTIESRGKDFSTCLPHLAVVTSFFFTVTFAYLMPPSGSPSVLDLLVSV

FYTAVP/PALNPLFYSLRNRDIKAALGRVLGG

>OranORc90912.1T

YGSKMSRGVCVGLVMATYVYGFLNGLIQTALILRLSFCGSNEVNHFYCAD

PPLLVLSCSDTFVKETAMFVVAGFNLICSLLIILISYGFIFAAILRIRSA

EGRLKAFSTCGSHLTVVTIFYGTLLCMHLTPPTERSVEQGKIIAVFYIFL

CPMLNPFIYIMRNNDVRDVMNSVLKFQLLTK

>OranORc91120.1P

LYFFLRPLSVLNLCLISVSVPKSILNALSNLQSISCLGCTSQVFSFMIFG

TADLAFLTAMSYDNYMTICRPLLYDIITDR/CGKMAAASWLIVGLYTLVH

MAATFSSHFCSLMSSQFFCDVPQLLKLM

>OranORc91569.1P

ICHILR*EVVMNRRARGKIAAASWLSGGLSSLTHMAATFSKPFCGSNVIQ

HFFCDAPHLLGHANSSVILREAEVTTFTTGLSLLCFISTIVSYVHIFSVV

LTMPSVEGRSKAFSTCLPQLVVVTLFLSTGAFAFLNLKSNGPSGMDLFLS

MFYSIVPPAMNPIIYSLRNRELKASLGRMLYGTGSN

>OranORc92205.1P

MANVSMVTEFLLLGFLE/VWELQLVHTALFLLVYLVALTGNLLILAIPTL

DQHLHTPMYFFLRNLSLLDLCYITTTVPKSILNSLTNSRSISFLGCTTQV

LLMILFGGSEFFILTAMSCDRYAAICCPLRYDTIMDQEVCRKMATASWLS

GGFYSLMHMAATFSSHFCGPRIIHQFFCDVPQLLKLMCPGEARAEICVLV

LSVILCLGCFVSILGLMSTSSWRS*RCQPPRAGPKPSPPVCLTSLGLFIS

LHQFICPSEHPRHLHRSWTCWVWFLYRVAPPPLNPL

>OranORc92471.1P

MANRTVVTEFLLLGFSEVRELQLIHAALFLLVYLAALAGNLFIVTITALD

RRLHTPMYFFLRHLSVLDLCYISVTIPKSIFNSLTNLNRGACEKMAATSW

LSGALSAILHTSATFSVPLGKDPM*STNSFCEIP

>OranORc92491.1P

MANRTVVMEFLLLGFSEVRELQLIHATLFLLVYLAALAGNLFIVTITALD

RHLHTPMYFFLRHLSVLDLCYISVTIPKSIFNSLTNLNRGACEKMAATSW

LSGALSAILHTSATFL

>OranORc92625.1T

TFGTNECLLLAVMAYDRFTAIRHPLLYTAMMTKKICTLLVASSYTGAGIN

ALVETTSVFSLSFCGPNVMNHFLCDFHPLLQLSCSDISLAKVLNSFVSGI

IVAITIPTVLISYLYILLAILKIPSTQGRRKAFSTCGAHLIAVALFYGTT

TFVYVLPNSRDTLEQNKVFSVFYIIVIPMLNPFIYSLRNREVKGTLRRTI

DQRRITSGSYFI

>OranORc93235.1P

MANNNSSKVTEFILLGLTENWELQVIFFVLFLIIYIITVLGNLVIILLIR

VHSQLHTPM*FF/PCDFFLCHLSFSDTCYS

>OranORc94068.1P

SSRPQSFFLIGIPGIEDIQCWVAVPFCIMYILALLGNNIILFIIKIDPTL

HQPMYLFLAMLSINDLVISTSTVPKMLAILWTHTQEMEYHACLIQMFFIH

TFSSLDS

>OranORc94356.1P

VANLTTLTRFLLAGFSEVRELQLVHAVLFLLVYLAALTGNLLIITTTALD

QRLHTPMYFFLRNLSFIDLCLISVTVSPS/SIYYWGCVTQVLCVIFFMGS

EMCVLMAMSYDRYAAICRSLCYDIIMNRGACGKMVATSWLSGGLFGVMYS

AGTFTLNFCKSNTIQQFFCDVPSLLKISCSKTHLVIDVSVAIGIGFGSFS

IVAIVTSYFRVFSILLMLPSTEGRAKVFSTCLPHLAVTNVFVFTAGFAYL

KPPSDSPSTLGLLVSVFYTVVPPTLNPLIYSLRNRDMKAA

>OranORc94358.1P

MSYDNYMTICRPLLYDIITDR/CGKMAAASWLIVGLYTLVHMAATFSSHF

CGPHVIHQFFCDVPQLLKLMFPREARAEVCILVVNVTFVLRCFVSLLVPY

VHIF

>OranORc94612.1P

MEKGNCTMVTEFILLGFSDHPELQLFFFLLFLVMYGITVVWNLGIIVLIK

ISSQLHTPMYFFLSHLSFVDLCDSTTIVPKMLTNIVTQSHTISYPGCLVQ

FYLFCTCVVTE/LLALMAYDRFVAICY

>OranORc94813.1P

LCPVSVTVPKSILSTLTGRGSISDSGCAAQVFFTLSFLTWEPFALPALSH

DRYAAVCRPPNRAVTASGRARVRISGSVLGAVPAASASSLSCSSTAVLPA

TSPRRRSCRAPNRPSPKKRPWPPGRRGCRPLYLHRCRLRSHLLRGPPRVF

STCPPHLVAFVASGISAYLKPPSDSPSVPDPSASPSYVAVPLAPNPLTSS

PRNGAMRAAMGKGDSGEASCW

>OranORc94995.1P

KSNMIQQSL*RPILLKISCSKTHLVIDVSVAIGIGFGSFSIVAIVTSYFR

VFSILLMLPSTEGRAKVFSTCLPHLAVTNVFVFTAGFAYLKPPLDSPSML

GLLVSVFYTVVPPTLNPLIYSLRNRDMKAA

>OranORc95390.1T

MANVSMVTEFLLLGFSEVWELQLVHAALFLLVYLVALTGNLLILAIPTLD

QRLHTPMYFFRQEHVSLDLCYITTTVPQ

>OranORc95685.1T

MEKGNCTMVTEFILLGFSDHPELQLFFFLLFLVMYGITVVWNLGIIVLIK

ISSQLHTPMYFFLSHLSFVDLCYSTTIVPKMLTNIVTQSHTISYPGCLVQ

FYLFCTCVVTESSGCTDAYDRFVAICY

>OranORc95829.1P

MNNHTSVTGFLLLGFSEVRELQLVHTALFLLVYLAALTGNLLVVTITTLN

RHLHTPMYFFLRNLSILDLCLISITIPKSIL/HSMSQNRSISFLGCVFQV

LLVILFAASEMFVFTVMSYDRYMAICSPLRYEVVMGRGACVKMVVTSWFS

GGLLGVLFSTGVSSLPFCDSHEVQQLFCDIHSLLKISCSEKHVAEDISIA

MEATSGFFSFGIRSPFLTFTSSWLC*RMPSSDARSKAFST/CLPRLVVFT

LF

>OranORc96865.1

MTQSNQTIVTEFILLGFSHLPELKPLLFVLFLGMFLITLVGNSLIIFVTM

TSSALRSPMYFFLRNLSLLEICYSLDIVPRLLIDLLADRRRISLPACALQ

LLLILSCVTSECILLTVMAFDRYVAICQPLRYGALMSPRLCLRLAVGTWM

AGVPVSLAFTLWLFSFPFCGRQEIHHFVCDISPLLRLVCADTGVFETHVL

AATILVVLVPFALIAVSYGRILSAVLGVRLVSGRSRALSTCTSHLLVVAL

FYGTAGVIHLQPRASYSPESKELVSLSYTVVTPMLNPIIYSLRNEEVRVA

LWRMWGKKKRSRTS*

>OranORc97855.1P

MDLWKNHTSATEFILSGFSSYSDVQGTLFMAFLVVYVFTLLGNLGMIILI

RKDSQLHLPMYFFISNLSFIDLCCSSNITPNMLVNCLAERKVISYSGCVT

QLCLFAAFVTIECYLLAIMAYDRYLAVCNPLLYPTIMSQHLCLELIIGSY

VAGILNSALQTFSVFRLSFCSSNVIQHFFL*YSKLLKLS

>OranORc99660.1T

ILVSYVHIFLAVLNMPATKGRTKSFSTCLPHLIVVFLYLCTGSFAHLKPP

SSSPSKLGLLVSVFYTVLPPTLNPLIYSLRNKDMKAALGKIFSVHFISRV

N

>OranORc100601.1T

MANRTAVRGFLLLGFSEVREVRPVQAAPFLPVYPAAPTGNLLVVAVTALD

RRLRTPVYLFLGHLCVLDLCLVSVTVPKSVLDALPDQRWICYWGCVAQVF

LVVPFAGSELLVLTAMSYDRYAAICRPLRYGVLVDRGACVKMAAASWLGA

GPFGAMYTAGT

>OranORc104336.1P

VIEFLILRFSEVWELQLVQATLFLL/YPVALTGNLLIVTVTALDWCLHTY

MYFLFRHLSILDLCYINVTTPKSILNSLTDSHSISFLGCAFQVFAFIRCS

CALTPLLTAMSFDHFAAICHPLHYEVIMDDGAWVKMSAASWFFGTLSTMT

HTVTTFSTLIWWSNDLPRFFCDIPQLIRLTGPDRNLQEFVAKTSSAGLTF

GYFLAIVVTYMCIFWAVLKMLTAEGQAKAFSTCLPYFAVVTLFIA/HLKP

VTDSPSAVDLLMLVFYTVVPPTLNLLIYSLRNQDMKATLRRMI

>OranORc107319.1P

IFTVMSYDRYVAICHPVHYEHIMDRAVCTQMIATSWFNGDTFGVMYMVGT

FSMPFCGFPMVQQFFCDAPSLLKASRFKRQVVLDVSFANGFIFALISFIA

ITLSYTRIFCT

>OranORc108860.1T

MANVSMVTEFLLLGFSEVWELQLVHAALFLPVYLVALTGNLLILAIPTLD

QRLHTPMYFFLRNLSLLDLCYITTTVPKSILNSLTNSRS

>OranORc110180.1P

MRMKEGNQSHVTEFILLGFASLPEFQVILFMIFLMIYLIAVIGNSL*VLV

STVDPALQTPMYFFLRSLSLMDISYTTVIIPKMLTNFLLKDKSISYGGCA

AQMCFSFSFGPSECWILMTMALTDRLPSVTRSTIH*L*TGGSACSSPWLP

G*HGFLWL*YRLQ*CLHCPFCGPNVI

>OranORc110968.1P

SGNLLIVAVTTLNWRLHTPKYFFLRHLSILDLCYISTTFPKSIVNSLTDR

REISFLGCVFQCFVFISLASTE

>OranORc112983.1

MDCEQEMKNRTTATSPGFILLGFSTHPAAQLGLFFLSLLLFLLILLGNLT

IVLLAWTDSVLLSAPMYFFLGHFSLLEMGFTSVTVPKLLADSFSSCHFIS

FAGCATQTFFFIALGSTECALLAVMAYDRYVAVCRPLRYLQEMRPEMCAQ

LVAGAWLSGFFNSTAHTAAVFQLSFCGSRVVSQFFCDLPPLLRLACGDRM

ASEAVVLMFGSLYGLTAFLVTLASYARVLFTVLGMGSTPGQYRAFSTCSS

HLAVVGLFYGSAFSTYLQPVSARPASQALLLPFFYALVTPTLNPFIYSLR

NREVKQALIRALGRKLF*

>OranORc113134.1P

MTNLTAATGFLLLGSSEVREVRPVQAAPFLPVYPAAPTGNLLVVAVTVLD

RRLRAPVYFFLGHLSVLDLCLVSATVPQSVHNSLADRREISPLGCAFQVF

AFRLCACAETPLLTAMSFDRYAAICLPLRYGVITGRGACEKMAAASWLFG

ALSG/HTIATFSVPVWRSNVLPQFFCDIPQLIRLAGPSGNLRESVTKTVS

VGLTFGYFLAIVVSYVRIFGAVLRMPAAEGRAGAFSACLPRLAVVTLFVA

NGASAYLRPVSDSPSALDPPVSVFYTVVPPALNPLIYGLRNRDVKAALRR

RIAGK

>OranORc114141.1P

MANGSAGTWFLLGGFSEVRELQLLQATLFLVVYLAALMGNLLLLTVITLH

RGLHTPMYFFLKNLSFLDICYISVTVPRSIFGSLTGDGSISFLGCVTQVC

LFVWLADTEVALLTAMALDRYVAICHPLRYEVIVTGTACIQTAAASWLSG

GLSAIAHVTTTFSVPLCGPHVIHQFFCDIPSLLHLSCSRFNLGELVLVAS

SCILGLGCFSLVVASYVRIFSAVLRAPSAQGRSKAFSTCLPHL

>OranORc117801.1P

KLM*PGEARAEVCALAVSVTWVLGCFVSILISYVDIFWATLRMRSTEGQA

KASSTCLPHLALCIFSWVC/LYLFMGLFAHLKLPLSSPSTLDLLVSVFYT

VLPLTLNPLIYSLRNKDMK

>OranORc119031.1P

MTNVTTVTAFLLLGFSEVQELQLVHAVLFLLVYLVALVGNLLIVTITTLG

WCLHIPMYFFLRHLSVLDLCLISITVPKSIHNSLINDGSISFLGCVLQVF

FALSSVFTELVILTVMSYNRYVAISHPLHYEVVMNRGACVKMTTTSWLSW

VLSAALHTASTFSFSFCGSNVVGQFFCDIPQLLTISHSPDLLNEVVPICV

KVAFDFCCFIGIVVSYIHIMATVLRMPATEG*TKAFSTCLPHLIITISLS

TGFFAYLKAFFAYLNAPLDSPSVMDLLLSMFYTVVPPTLNPLIYSLRNRD

LKVTMGKFLKGKFCTREKSLHISHLA

>OranORc119912.1T

MANVTTVMAFLLLGFSEVQELQLVHAMLFLLVYLAALVGNLLIITITNLG

WHLHTPMYFFLRHLSVLDLCLISITVPKSIHNSLINDRSISFLGCVLQVF

FALSSVFTELVILTVMSYSRYVAISHPLHYEVVMNRGACVKMTTASWLSW

V

>OranORc121776.1P

KLM*SGEARAEVCALAVSVTWVLGCFVSILISYVDIFLATLRMRSTEGQA

KASSTCLPHLAVISLYLFMGLFAHLKLPLSSP*ALDLLVSVFYTVLPLTL

NPLIYSLRNKNMKIFPNWGKIFSSNFIS

>OranORc122227.1T

MFNITNGTEFLLLGFSEVRELQLVHGMMFLLVYLAALMGNLLIVTVTALD

ERLHTPMYFFLSNLALIDLCLISVTVPKSVVNSLTNNRSISFLGCVLQVL

FFISLAATEMILLTVMSHDRYTAICHPLRYEVVMNRGACGKMAAASWLSG

GLSGLMHMATTFSEPFCGSHVIHHFFCEAPHLLAQAGSTVILRRSQSHDL

LLQAPLLFFCF

>OranORc126184.1P

ICDPLR*SLIM/NRRFCLQLSLASWLAGIPVATVKTTLMFTLTFCGPNVI

NHFFCDSPPVLDLVCTDTFAVEIYSVTATVMVLMLPFGVIVVSYIRILVT

ILKMSSAEGRRKAFSTCSSHLVVVSLFFGAAGSTYFRVKASYSPETKKLL

SLSYSVFTPMLNPLIYSLRNQEVKGALKRILGRKISSQDL

>OranORc127143.1P

LSCGPSLGQPGRAAVLGWGLISLPPLLILGSYAGIVAAV*GSPRPGG/LR

VPSAGGKRRAFSTCGSHLAVVSLFYGTVIAVYLFPSGPDSVEKARIAAAA

LYTAVTPLLNPFIYSLRNSDLHRALR

>OranORc131934.1P

SGNLLIVAVTTLNWRLHTPKYFFLKHLSILDLCYISTTVPKSIVNSLTDR

REISFLGCVFQVFVFISLASTKMALLTVMSFNRYVAICLPLRYEFVISRG

ACGKMAASSWFSGSLSGLMQTAVTFSLPFRGSNEIHQFFCDIPQLLKLSG

SDWIMAKM

>OranORc132713.1P

DPQIQLFLFLVFLLIYGIALVGNLLTMLVIQVDPHTST/PHLYTPMYFFL

SNPS/FLDVRFASSTVPRMLENFLSDQKSITFPECITQIVSLFILATVKI

YLLATMAYNCYRVSGQLLCYPGSLTI*LCVQLVRGAWLVGDINVVVNALL

VLRLDFCRPNQNLHFSCEFELPPLLQISCSNIFASEMGILSLGVLLGLVS

FLLTLISYIHVISILFQICSS*GHGKAFPTCSSHLITVLLFCGTVFFQYM

RPSSA/SHPLALDWVVSIQCSILTPMLNSMIYILKNWDMMRGLKKLLRK

>OranORc133507.1P

QSPMYFSHEVCRTDIGLYDCHHPQNAHQFTVQG*KYFLWWLCCPDVSRFF

FGPSECWILTTMAYDRQAAICDPLRYSLIMNRRFCLQLSLASWLAGIPVA

TVKTTLMFTLTFCGPNVINHFFCDSPPVLDLVCTDTFAVEIYSVTATVMV

LMLPFGVIVVSYIRILVTILKMSSAEGRGKAFSTCSSHLIVVSLFFGPAG

STYFRVKASYSPETKKLLSLSYSVFTPMLNPLIYSLRNQEVKGALKRILG

RKISSQDL

>OranORc136267.1P

NLTTLTRFLLAGFSEVRELQLVHAVLFLLVYLAALTGNLLIITITALDQR

LHTPMYFFLRNLSFIDLCLISVTVSPS/KSIVTSLTDLHSIYYWGCVTQV

LCVIFFMGSEMCVLMAMSYDRYAAICRSLCYDIIMNRGACGKMVATSWLS

GGLFGVMYSAGTFTLNFCKSNMIQQFFCDVPSLLKISCSKTHLVIDVSVA

IGIGFGSFSIVAIVTSYFRVFSILMMLP

>OranORc137159.1P

DPCLHMPLHFFLCYLSFLDLCFSTSLAPQTLVNLWGPIETITFGGCVVQL

YVSLALDSTECILLALMALDLYAAVCQPLCYAMLMHHSLFRQLAAVSELC

GVANSLVHTKLIMCLPQFGHRHLDHFIYEAPALL/MEVTINELVIFTLSM

LEDIVPPAFILVSYGFIAQSVLRVGSSKG*RKVFSTFSSHLLVVSILDGT

IIFMHLHPGGNYSQD*GKFVSLFYTMVMPTLNP/PIVYTLRNQEVKGAMK

KLV

>OranORc137183.1P

GFFHGELLTSF*VHITVRGPWEILHFYCDGHLLVELSCSDIHLIQIISSV

SAAIIILSTVLTVLISYVYILITIMRIQASDGRQKAFSTCTSHLTAVILY

YGTLAFTYMQPSSSHSLERNKVVSLFYTVVIPMLNPLIYTLRNKDVKGVV

IKIMGKNVCSL

>OranORc137399.1P

YDRYTAICHPLR*EVIMAQGA/SCGKMVVTSWLSRCLLGASLSAGALSLP

ICGPREIQQIFRDIPSLLKISCSEEHTTLDLTVAIGFCLTLFCFVSIVVS

YMHIFSTLLSIQSAEERSKTFSTCLPHLTITSAFFTTGSFPYLAPTSESP

SALDLLVSVFYMMVPPALNPLIYILRNRDIRTSLGRVL

>OranORc137672.1P

MVNHTTVMEFLLLGFSEVRELQLVHAVLFFLLYLVALTGNLLIAAITTLD

WHLHNPMYFFLRHLSILDLCLISVTLPKSNLVSLTTCYSISFLDCCLQVL

LVLLFATSELFILTAMSYNHNTATCHPLR*EVIMAQGACGKMAATSWLSK

YLLGASSQLVHYPCPSVGP

>OranORc138167.1T

NFLSKDKNISYGGCAAQLYFSFFFGPSECWILMTMAYDRQAAICDPLRYS

LIMNRKFCLQLSLASWVAGIPVATVQTAMMFTLPFCGPNVINHFFCDSPP

LLELVCTDTFANEVYGVIGTVIFLMLPFGVIIASYIHILITILKMSSAKG

RRKAFSTCSSHLIVVSLFFGAASMTYFRVKSSYTSESKKLLSLSYTVFTP

MLNPLIYSLKNQEVKVALKRILGKKDIFPGSGRL

>OranORc139701.1P

NWTVVTEFILVGFPGSLEVRALVFPLFLATYLLTITENVVIILLVQKDRT

LHKPMYLFLAHLSFLEIWYISVTVPKLL

>OranORc139895.1T

EDAVTSFSALLASARFVSIAVSYARVFRAVLRMPAAQGRAGAFSTCLPRL

AVVTVSVSLPQARVGLPLDAGPAGVPVLRRGAPSPRPPHLQPEEPGPEGR

SGDLKLISGR

>OranORc141228.1T

TQSHPSSFLLLGVPDLEAAHNWLSFPFCSVYLIALMGNCTILFVIKTEQS

LHQPMFYFLAMLATVDLGLSSATIPKMLGIFWLHLQEISFGSCLTQMFFI

HMFTGMESTMLVAMGYDRYVAICKPLRYCTILTNKSIRVILGLAVLRNFS

LIIPMVFFLLRLPYCGHRIIPHTYCEHMGIARLACASIKIDIIYGLCVIC

LILLDILLIALSYIRILHTIFRLPSQDARLKAINTCISHICVILAFITPA

LFSLLTQIFGHYKIPSYIHILLANLFVVFTPAINPILYGVKTNQIRERVS

HIFTTKPTF

>OranORc145043.1T

MGNQTRVTEFILLGLTDSPKWQAVIFFFLFVTYALSITGNLTIFNLTLLD

SRLHTPMYFFLRNFSFLELSFTSACIPRFLVTIATGDRTVSFAGCMTQFF

FVILLGATEFFLLTAMSYDRYVAICRPLHY

>OranORc147270.1P

VIEFLILRFSEVWELQLVQATLFLL/YPVALTGNLLIVTVTTLDWRLHTY

MYFFFRHLSILDLCYINVTTPKSILNSLTDSHSISFLGCAFQVFAFIRCS

CALTPLLTAMSFDHFAAICHPLHYEVIMDDGAWVKMAAASWFFGTLSTMT

HTVATFSTLIWWSNDLPRFFCDIPQLIRLTGPDRNLQEFVAKTSSAGLTF

GYFLAIVVTYMCIFWAVLKMLTAEGQAKAFSTCLPYFAVVTLFIA/HLKP

VTDSSSAVDLLMPVFYTV

>OranORc147564.1P

FAICPLLTLCSTTTAVPPALVNCMRDCPTITYNDCFAQMTISLFWGITEC

CLLAVMAYDRFVAISNPLRYTLIMTMRACFQTAATMWISNFLLALIPVVT

IPVRFCAGHNVVNHFVCELEAVLKLVCSDTTVSEILMLINSIFIFPLPFL

FILLSYIRIVVAILKIPSTAGRRKAFSTCGSHLIVVTIYYGTLISIYLKP

QNKDSKDQDKIISIFYGTVIPMMNPLIYMLRNKDMIGALRKAVGKTKQVK

LSI

>OranORc154878.1P

ILEISLEKMANVTTMMEFLLLGFSEVREQQLVHAVLFLLLYLAALMGNLL

IVAVTTLDQRLHTPMYFFLRHLSILDLCYISTTVPKFIVNSLTDRREMSF

LGCVFQVFFFISLASTEMALLTVMSFDRCMAICLPLHYEFVIS*GACGKM

VASSWFSGSLS

>OranORc157995.1T

AFIAAAILRIQSAEGRRKAFSTCAGHLVVSLLHNGCAIFIYIRPKSAYAP

DQDKVVSLVYTNVTPMLYPMIYSLRNQEVQGALRRVLGRKIISWVS

>OranORc160196.1P

NHLLVAVPTLNWILLTPKYFFLRHLSIGDLY*ISSPVPLSIGNSLTEQRE

VSFLSCVF*VFVFISLASTKMALLTVMSSHR*IAICLSLRYNFVISAWAC

VDDDSFVLVLWESFRDLCKQRMTFYPPFPGSNEIINSSVIYPQLESSPGS

DW

>OranORc161765.1T

MANCTYVTRFLLLGFSEVRELQLVHAALFLLVYLVALTGNLLIVAVTALD

RRLHTPMYFFLRKLSFIDVCYISECIPKSIFNSLAHRCD

>OranORc162663.1P

MVVISTGDRSISNNACAIQSLFYILLGATEFFFLATMSYDQCVAICWRSH

YLTIMSLSVCSLLVLCSWLVAYLIIFPDLILGLQLDIRDGTI/NHFTCDS

ALKLEILCTDTQLFERMNFTFAMGTLLVTLDLVTMSYMAITCTNLRLSFT

QQRKKAFSTCCSHMIVIFLSYGSCMFMHIKPSPKEGLDFNKRVAVLNNSV

APMLNPY

>OranORc164943.1P

MGNENNVTKFILLGLTRDPKMQKIISVVFLIYH/F*FIITVVGNLIIVLT

VASSQTLDSPVYFFLAHLSFIDACYSSVNKSKLIGESLSENKIIDFKGCM

TQVFGEHFFGGTEIILLTVMAYDRYVVICQPLHYTTIVSRCPCSSLVAVT

WAGSFLQATVQVLFMVHLPFCDPNVTDHFMCDLHPLLKLVCSDTHTLRLL

VAVNSG/CVLIFVLLMSSSVFILHSLK

>OranORc165408.1P

LFVAIFIFYLFTLVGNVAIILVSHLDPWLHTLMYCFLIKLSFLDLCFTKS

ISPQLLTTTRSRLKSMSYCRCAAQLFQATALGSTECFLLALMAY*CYVAL

CRSPHYVVIMYPQLCQML

>OranORc166633.1P

MANVSMVTEFLLLGFSEVWELQLVHAALFLLVYLVALTGNLLILAIPTLD

QHLHTPMYFFLRNLSLLDLCYITTTVPKSILNSLTNSRSISFLGCTTQVL

LMILFGGSEFFILTAMSYDRYVAICCPLRYDTIMDQEVCRKMATASWLSG

GLYSLMHRAATFSSHFCGPRIIHQFFCDVPQLLKLMCP

>OranORc167478.1P

EDMLNCTTVMEFLLLGFSEVRELQQVHATLFLLVYLATLMGNLLIFAVTI

LDRHSTPP/ALHTPMYFFLRNLSILDLCLISVTVPNSILNSLTDRRPISF

LGCVLQVFFFISFASSKITLLTSMSYDRYLAICCPLHYEVIMNPRACGKM

AT/SWLRGGLSGIMHSATVSSLPFCRVNVVHQFFCDIPQLMRLSGSE

>OranORc167844.1P

MGNGTAVTEFLLLGFSEVRELQLFHAALFLLLYLAALMGNLLIVAITVLD

RHLHTPMYFFLRNLSVLDLCLISAIVPKSILNSLTNNRSISFPGCILQVF

FFGFAASSEMALLTAMSHDRYVAICCPLSYDIIMHPKACGKMVAASWLSG

ALNGLLHTAASFSSPFCGSKVINQFFCDIPPLLRLSCSRGFHNELGVIAF

NVLIGSSCFASITLSYVRIFSAVLRMRSTSAGAKAFSTCLPHLAVVTLFV

SPAL*KYSTFIAKPPN

>OranORc170735.1P

MSMDNRNRNTTQSHPSSFLLLGVPDL*AAHNWLSFPFCSVYLIALMGNCT

ILFMIKTEQSLHQPMFYFLAMLATVDLGLSSATIPKMLGIFWLHLQEISF

GSCLTQMFFIHMFTGMESMMLVAMGYDRYVAICKPLRYCTILTNKSIRVI

LGLAVLRNFSLIIPMVFFLLRLPYCGHRIIPHTYCEHMGIARLACASIKL

D

>OranORc176864.1P

MEKLTTVTEFILLGFLGGSWLQGTLFLTFLVLYGMTVVGNLGMVAIISLD

PQLHTAMYSFLCSLSLLEVCYSSTIAPRALLNFLSERAAISFPGCATQFF

FLSLFGTTEAFLLASMAYDRFIVICDPLHYSMIMSHGICHLLVGGLYLWG

VVNALTQTTMIF*LLFCGPNKIDGFFCDVLPILTPSCSDTLSNQLVLLGL

GGSIIMGTFSIVSISYVLVLSTILRILSAEGQMRAFSTCVSHLVGVGLFY

GSVFFMYAQSGATSNMEQSKVVSIFYTVIIPGLNPIIYSLRNKDVKEGIE

/RRALKRIRKKLSL

>OranORc181103.1P

MANVSMVTEFLLLGFSEVWELQLVHAALFLLVYLVALTGNLLILAIPTLD

QHLHSPMYFFLRNLSLLDLCYITTTVPKSILNSLTNSRSISFLGCTTQVL

LMILFGGS*FFILTAMSYDRYAAICCPLHYDT/PCTMTPIMDV*VCMKMA

TASWLGGGLYSLMHTAATFSSHFCGPRIIHQFYCDVSHLLKLMCHAEAQA

*ICA

>OranORc183637.1P

HYTTIMSPRLCALVVAGSWIVSSLHALTHALLVVRLSFCSNHEILHFFCE

LYQVLRLSCSNIYTNEVVMFGSAVGLGIIPLTGLLFSYTRIVSTILSVPS

AKGRYKAFSTCGSHLSVVSLFYSTSLGTYLCPTSNQTSQLGSIASVMYTV

VTPMLNPFIYSLRNKDMKGALKKLVSRKLIFT*IL

>OranORc192576.1P

MENENNVTEFILLGLTQNPGMQKIIFVVFLAVYSVTVIGNLLIMATINSS

KTLGCPMYFF

>OranORc196512.1T

MENLTTVSEFILLGFRGGLWLQGTLFLTFLVLYGVTVVGNLGMVAIISLD

SQLHTAMYSFLCSLSLLEVCYSSTIAPRALLNFLSERAAISFPGCATQFF

FLSLFGTTEAFLLATMAYDGFIVICDPLHYSMIMSHGICHLLVGGLYLWG

VVNAVTQTTMSFRLLFCGPNEIDGLLLQRPPDTDPVLLRHADQPAGLARP

GGSIIVGTILHPLDLYVLVLSTIL

>OranORc197259.1P

IAWFLLGMDVGLIIVSYAQILRAVLKLHSAEAASKALSTCSSHIILILFF

YTAIVVLPITHTAKRMVPFIPVLLNVLHSVIPPSSQP/PPALNPIVYALR

TREIKLGILNLIGLAGKRK

>OranORc198220.1T

VRSAVLVAMAFDRYVAICDPLRYTTVLTGSLVCRLGLMALAKGMVLILPI

PLLLRQLTFCRTVIAHTYCDHMAVVKMACGHTGPNRIYGLFLVVLVVGLD

CPLIGASYALILWAVLHLSSRGARLRALSTCSAQLSVVLVTYGPALISAI

IHRFGRSIPVHSHILLANLYLLIPSLFNPVIFGVRTKEIRDMVTKHLGHC

PGT

>OranORc203517.1T

NKAFSTCLPHLIIITISLSTGFFAYIKAPLDSPSVMDLLLSMFYTVVPPT

LKPLIYSLRNRDLKVTMGKFLKGKFCTREKSLHISHLA

>OranORc203642.1P

FYIAMGTLLVTLDLVTMSYMAITRTNLRLSFTQQRKKAFSTCCSHMIVIF

LSYGSCMFMHIKPSPKEGLDFNKRVAVLNNSVAPMLNPY

>OranORc206054.1T

LNGFLVIFPPVILGLQLDFCSSVATDHFFCDVSPLLLLSCLHTVFLELMA

FILALGTLLVTLMLVAVSYTAIAHTILRLPSDQQKRKAFSTCSSHMVMVS

ITYGSCIFIYIKPSIKDRVDLTKGVAMLYTSVVPMLNPFIYTLWNEQVKQ

AIWDLMHRFGFS

>OranORc215889.1P

PNVLPGDGASGGAEGVCS*FLPFLLLIKARVNSFRSTVVPHTYCEHMAVL

KLATDDTRVNRIYGLAVVFIIFWFDSSFITTSYTLIFKAVFRLPGKEARL

KAFNTCTAHIIIIMLTYTLALFSFLGHRYGHHLTPYVHILLANFYLLVPT

VVNPIIYGAKTKEIRVRVITMFSLRGTLSKM

>OranORc216494.1T

IITLDSQLHTAMYSFLCSLSLLEVCYSSTIAPRALLNFLSERAAISFPGC

ATQFFFLSLFGMTEAGLLASMAHDHVVPICDPLFFLIKKSHGICHLLVGG

LFVGGVVNTVTQTTVTFQLL

>OranORc217050.1P

FPAGHLSILDLISVTLPKSILVSLTTATPISSWIVVCKVVLVLLFATSKL

FILTAMSYDRYTTICHPLR*EVIMAQGACGKMAATSWLSRCLLGASLSAG

ALSLPICGPREIQQIFRDIPSLLKISCLEEHTTLDLTVAIGFCLTLFCFV

SIVVSYMHIFSTLLSIQSAEGQSKTFSTCLPHLTITSAFFTTGSFPYLAP

TSESPSALDLLVSVFYMMVPPALNPLIYILRNRDIKTSLGRVL

>OranORc225178.1T

MASANHTNLDPSVFFLLGIPGLESVHFWFSLPVCSICLVTVLGNCTTLLA

ILSEPALHKPMYLFFCMLSLIDLVACASTLPKMLSIFWFNAGWIDANACL

VQMFFIHSFCMMESTVLLAMAFDRYVAICHPLRYAAIFTNAITVKIGLAA

IARGSLLMFPCPFLIRRLSFCRTNVIPHTYCEHMAVVKLACGDTTVNRVY

GLAAALLVIGVDLLFIGLSYFLIVRAVLRLSSQKARAKAFSTCGSHLCII

LISYTPALFSFFSHRFGHRVAPHIH

>OranORc232875.1P

PPALKLSGSDWIMAEMDISALIACLAFICFVSMIISYVCIFSAVLRIRSS

EGQNKAFSTCLPHLVIVAFFFSTSSFAYLKPPSDSPSFQDLLVSMFYTVV

SPTVNPLIYSLRNK

>OranORc234921.1P

REMSFLGCVFQVFFFISLASTEMALLTVMSFDRCMAICLPLHYEFVIS*G

ACGKMVASSWFSGSLSGLMQTAVTFSLPFRGSSEIHQFFCDIPQLLKLSA

SDWIMAEMGISARIACLAFVCFVSMIISYVCIFSAVLRIRSSEGWNKGFS

TCLPHLIVVAFFFSTGSFAYLKLPSDSPSFQDLLVSVFYTVVPPALNPLI

YSLRNKDMKAALKKILGREKFT

>OranORc235525.1P

SVPKSILNALSNLQSISCLGCTSQVFSFMIFGTADLAFLTAMSYDNYMTI

CRPLLYDIITDR/CGKMAAASWLIVGFYTLVHMAATFSSHFCGPHVIHQF

FCDVPQLLKLMFPGEARAEVCILVVNVTFILRCFVSLLVPYVHIFCEPHV

GQTDYPVSTPALRTVLGT**ALNKYQHYYLLGMLRMQSTEGRAKAFSTCL

PHFTIVTSFIVNGA/AHLKPVSDSPSTLDRMVSMYYTLVPPTLNSLICSL

RNGAMKATLRRMIA

>OranORc241400.1P

LFKLFFFISVASTEMALLSVMSFKNCLAICLSL/EFVISRGACGKMVASS

WFSGSLSGLMLTPMTFSLTFRGSSEIQ*IFCDILPLLNLSDSEWIMADMG

ISTAIVCLAFVCFVAMIISYVCIFSAVLRIRSSEGWNKGFSTCLPHLIVV

AFFFSTGSFAYLKLPSDSPSFQDLLVSVFYTVVPPALNPLIYSLRNKDMK

AALKKILGREKFT

>OranORc248933.1P

STLTQSNFISSFFICNPNFPSYWLGGKNKILLFMTS*DQYAAICSPLCYD

VIVN*RACGKMAATFWLNGGLSGLLCTSATFSMPFCEPQGIHQFFCKIPH

LLKLSYSRANLAEMGIAALTTLLDFVVFFYIVSSYVHFFAAVLRMPATEG

RSRAFSTSLPHLTVFSVFLSTEAFAYLKPTSCSPIVMHLLVSLFYIVVTP

P*TP/PTLNPLIYSLRNRDLKMVMG

>OranORc249081.1T

VSITVPNSVHNSLINGRAISLWGCVAQVVLVISFAFSELTVLTAMSYDRY

AAICLPLRYEVIVDRGACGKLAAASWLGGGLFGAMYTAGTVRLSFCGSNL

IQQFFCDIPSLLKISCSETHVVIDVSVATAFSLAFVSFVFIVVSYVRIFG

AVLRMPASEGRARAFSTCLPHLVVVTVFLSTGAVAYLKPVSDSPSSLDLL

VSVFYTTVPPALNPLIYS

>OranORc250352.1T

MGSENKTGASGFILLGLSSDPEQQRLLLVLFLTLYLVTVGGNLLIVLAIG

TDSHLQSPMYFFLANLSLVDVCFSSTT

>OranORc252511.1P

MANVSTVTEFVLLEFSDGSRELQLGHATLFLLVYMVTLMGNLLIIAATAL

DRHLHSPMYFFLRNLSFIDVCYISVTVPKSIHNSLTNNSLISLQG*ATQP

FLISFFTASELSVLTVMSYDCYVAICHPLHYEHIMDRAVCTQMIATSWFN

GDIFGVMYMAGTFSMPFCGFPIVQQFFCDAPSLLKASRFKRHVVLDVSFA

IGFI

>OranORc254385.1P

ILTDNSIVKMRLAVFIHSFIMILPLLFFLRRMPFCKANVLHHAYCLHPDL

ILLPFVDTTFSSSFGLYIIVS/LTFLSYILIFRCVLTITSQAGQSKAANT

CLPHLCSAHLLIIMLVFVKRL

>OranORc265035.1P

MANGTEVTEFLLLGFSEVQELQLVHATLFLLIYLAALTGNLLIIAVTSLD

RHLHPPSVCVEQETVCFSLPPHLRHCPFYI*KKMTNNNSISFPG

>OranORc268998.1P

APLRYEVIMAQGAVGRWRPLPGSANTFLGASLSAGALSLPICGSREIQQI

FCDILSLLKISCSEEHTTLDLTVAIGFCLTLFCFVSIVVSYMHIFSTLLR

IQSAEGRSKTFSTCLPHLTITSAFFTTGSFPYLAPTSESPSALDLLLYVF

YTLVPPALNPLIYSLRNRDIKSSLGRVLQG

>OranORc269244.1P

MGNGTAVTEFLLLGFSEVRELQLFHAALFLLLYLAALMGNLLIVAITVLD

RRLHTPMYFFLRNLSVLDLCLISAIVPKSILNSLTNNRSISFPGCILQVF

FFGFAASSEMALLTAMSHDRYVAICCPLSYDIIMHPKAVG/EMVAASWLS

GALNG

>OranORc270987.1T

ADPRLGAPMYFFLSHLSVVDLLFASVTVPQLLADLLTAGPRAVLPRACFT

QMYFFVALGITESYLLAGMAYDRAVAVGRPLLYGAVMTRRRCGLLVGGAW

VVAHLHALLHTRLIAGLAYPRPARLRHFFC

>OranORc275903.1P

MEERNCTIASQFIFLARIEARCVSDVSHNLRDHPCGNLTMVTLIEISPQL

HSPMYFFLRNLSLLDVVYSSDIVPKVLINYLLETKVISFGECVVQFFFLS

LCVNTELFLLATMAYDRFIAICHPLLYTVMMSKEVCILLVASSYLLSCVS

AIVH

>OranORc276778.1P

MGNGTAVTEFLLLGFSEVRELQLFHAALFLLLYLAALMGNLLIVAITVLD

RRLHTPMYFFLRNLSVLDLCLISAIVPKSILNSLTNNRSISFPGCILQVF

FFGFAASSEMALLTAMSHDR

>OranORc276968.1P

MNNHTSVTGFLLLGFSEVRELQLVHTALFLLVYLVALTGNLLVVTITTLN

RHLHTPMYFFLRNLSILDLCLISITIPKSIL/HSMSQNRSISFLGCVFQV

LLVILFAASEMFVFTVMSYDRYMAICSPLRYEVVMGRGACVKMVVTSWFS

GGLLGVLFSTGVSSLPFCDSHEVQQLFCDIHSLLKISCSEKHVAEDISIA

MEATSGFFSFGFVVLSYIHIFLAVLRMPSSDARSKAFLHLPAPPCHFSLF

LS

>OranORc278876.1P

LLGFSEVRELQLVHAALFLLVYLATLMGNLLIVTVTALDWHLHTPMYFFL

SNLALIDLCLISVTVPKFVVNSLTNNRSISFLGCVLQVLFFISLASTEMI

LLTVMSYDRYAAICHPLRYEVVMNRWACGKMAVAWWLSGGLSGLMHTAAT

FSKPFCGSNVIQHFFCDAPHLLGHADSTVILREI*VTTFTTGLSLFCFIS

IIISYVRIFSVVLKMPSVEGRSKAFNTCLPHLIVITLFL

>OranORc281117.1P

LGVLLVTLVLMTASYTATVCVI/LSVSSAQQRRKAFSTCYSHMVMVSITY

GSCIFAYIKLSATDRVELIKRVAVLNTSVAPMLNPFIYTLLNEQVKQAFR

ALVH*IGFSSRK

>OranORc283511.1P

GNGTIGLIIWIDHSLHSPMYFFLANLAALEICYSSTIAPLTLVNVRSMGN

SFISLLGCGTQMFFFIFLGSSDCIVLAVMAYDRFVAICQPLRYTLIMRWR

LCVQLAAGSLALGFTLAVPLTVLIFQLPFRGNSEIGFFYCDVLPVILLAH

ADTRVHEAIVFAVSVVVLTIPFLLITASYVFITAAILRIRSAEGRRKAFS

TCSSHLTVVLLQYGCGSLIYLCPSSA

>OranORc283516.1P

SWETHHFFCDISTLLELVCHDTAI*ENGLFLSGILFPLPPISVIMASYRE

SLQIVLEKGSSQGS*KVPTTCFSHIVVVCLIYGATIFKYLLPKSYHTSSQ

DQVVSVFYTLLTPMSNLLIYSL*N*EALKTLGKVL

>OranORc284585.1P

NQTMISEFILLGFSDDPQQQPFIFGLFFFMYLIAVLGNLLIILAIGFDSH

LHTPMYFFLSKLSF/ANYLFVDLCLTTTTLPKMLLNIQTRKENHLLHWAA

LTQAYFLIFFL

>OranORc287206.1P

MGNGTGVTEFILVGLTNDPHLQALLFLVLFLTYALSVTGNLTIVTLTLLD

SRLHTPMYFFLRSFSLLEIAFTSACIPRFLVTIVTGDRTISFSNCFTQLF

FFIFLGVTEFFLLAAMSYDRYVAICRPLHYTTVMSRGVCSLLVLCSFLSS

YLIVFPPVVMIARLDYCASNDLQPLHLRLFPKDGAFLLIHPLFGADGFPF

NLGNNAHYLVLMSWSSAPIIRAIPKMLSCQKTEK

>OranORc290265.1P

MVNHTTVMEFLLLGFSEVRELQLVHAVLFLLLYLVALTGNVLIVAITTLD

WHLHTPMYFFLRHLSILDLISVTLPKSILVSLT

>OranORc290716.1P

MTNSLTVKEFTLLGFLEV*ELQLVHTALFLLV*LVAQMGNPLVVSVITLD

RSLHYSLYFFLKHLSVLDLSYIFIIAP/HHCPNSLTKGRNFYLLGCVLQL

FFLLLFACIELFFLTVLSYN

>OranORc291693.1P

LGFSDVWELQQVHATLFLPAYLAALMGNLLIFAITILDRQLHTPMYFFLR

NLSILDLCLISITVSNSILNSLTDCRPISFLGCVLQVFFFISFALSKITL

LTAMSYDRYMAICCPLHYEVIMNPRACGKMATT/GLVGRWPPPSWLRGGL

SGIMHSATVSSLPFCKVNVVHQFFCDIPQLMRLSGSEGMLQEVEVCTFLT

LLALICFAFIGVSYVHIFWAVLKMPSVEDWAKAFSTCLPHLVAITV/YLK

PPSDFPSMLDLLVSVFYTVVPPALNPLFYSLRNRNMKTALGRVAWGKL

>OranORc292930.1P

MGFSGTREFQLVKVILFLLVYLAALAGNLLVIAITTFDWHLQTPMYIFLK

HLSFLDLCYISVTTPKSFTNSLTRTGSLF/LSFLGCADQVFLFSVFVCTE

MGILTVMSYDHFVAICHALHYEAVMSRGAYVQMLVSLWARGSLAGLMNIT

IFALPFCGSNVIYQFFCNVPQLLRVSSSNDTAGEVRVTTLIAMLFFICFM

FIIVPYTPSLIAHTPSPCKS*LLPHRVLRSLDPIRLSKSISPHLASLSSL

PTLDDQVSALNSTLSTHLN

>OranORc293563.1P

LLIITFFKTTLLGNGVILGPCLLGMDGRLHTPMYFFLCHLAVVDISYACN

TVPRMLVNLLRPAEPISLAGCSAQTFLFLTLAHTECFLLVVMSYDRFVAI

CRPLRYPLVVSRRACRILVGLCWALGSLLALLHLLLLLRLPFCGRREIDH

FFCEILALLRAACGDARINEALVLAGAVSVLVGPFFSILTSYLSILWTIL

RLRSREGRRKAFSTCASHLGIVGLFYGTAIITYVGPGTANPGEGKKYLLL

FHSLLNPMLNPMIYSLKNGEVKKALREVM

>OranORc294350.1P

TILKMSSAEGRRKAFSTCLSHLIVV/FFGAAGSTYFRVRSSYSPESRKLL

SLSYSVFTPMLSPLMYSLRNQEVKDAPKRILGKRIVSQDLRGSD

>OranORuc153.1P

VFP*FI*LISFLSFISIGLGHQVISHTYCEHMGIAKLACVNIKINIIYGL

TVALLVTGVDVVLIGFSYGFILHAVLHLPTQDAQRKALHHLWGPCMCYLG

LLYPSF/FSFFTHRFGHWVPPQVHIFVANLYLLMTPVAQPAGCMAFTTKH

IPPEI

>OranORuc193.1P

MLAMRNNSLGEHFILMGFSDQPQLELILFVVVLTSYLLTLVGNTIIIVVS

RLDPRLQTPLYFFLSHLSLVDLCFTTSIVPQLLWNLWGPSKAITVVGCAI

QLYVSLALGSAECILLPIMAFDRYTAVCRPLHYRSIMHPRFCHALAAVAW

TIGLGNSVIQSTITLRLPRCGHRHLAHFICEVPALIKLACVD/KANEILL

FMATLVLLLLPMTLIM*SYGFIARAMLRIKSSQAWQKALGTCGSHLLVVT

LFFGMSSIIYIQPNSSFSKTSGKFLTLFYTVVTPTLNPIIYTLRNKVMLG

AVRRLLWKD

>OranORuc201.1

MESENQTLVTEFLILGLTDNPQLQPFLFLLFLTIYFLTVLGNVTILVVTC

FDSRLRMPMYFFLSNLSFLDVCYTSVTVPRMLLNLLSQFKAISYTACISQ

LYFFIVFAGTECFLLTAMAYDRYLAVCKPLRYPALMDNRRLLLLATAAWG

TGIFNATSHISFTVRLVFCGPNEIDHFLCDIRELLKLACSSTRNNQLEIF

LVGGTLRITSFCLTLGSYVPIIITVLKIRSEESRKKTFSTCASHLTVVVL

YYGTLNFVYGRQTSGNTLYMSKMMSVMFSVVIPMLNPLIYTLRNQQVKGA

IGNFWYTRCCLVKPPLSGG*

>OranORuc201.2

MNGALGNLTTASEFVFLGFADLPEGQVLLFGLFLLMHVITLSGHLTITLI

TLIDPCLQTPMYFFLRNLSSIEICYTLAIVPNMLANLISVTREISFAGCA

VQMYFFVALGTSECFLLSVMSYDRYTAICNPLRYSVIMSRSSCQRLLLAV

GTSGFVLALGLTVLIFRLPFCGSHAINHFFCDIPALLFLACSDTRVNELV

VFALCFLALLIPFLLILLSYAFIAAAILRIRSAEGRRKAFSTCAGHLVVS

LLHNGCAIFIYIRPKSAYAPDQDKVVSLVYTNVTPMLYPMIYSLRNQEVQ

GALRRVLKRKVFSEKM*

>OranORuc201.3P

IYILPKSAYAPDQDKVVSLVYTNVTPMLYPMIYSLRNQEVQGALRRVLGR

KIISWVS

>OranORuc201.4

MDPVQENSTSPSEFIILGFGNHPELQLFFFGLFFIIHLITLTGHLTIVLV

TSVSHSLRIPMYFFLRNLSAIEILYILVIVPNMLANFLSKNKTISFVGCA

LQMHCFIALGGAECFLLAFMSYDRYVAICQPLRYAVIINRTLCFQMLAIA

GLSGFALSLSLTVLIFRLPFCGSHAINHFFCDIPALLFLACTDSPVNEVA

VFIVCVLILLIPFFLILLSYAFIAAAILRIRSAEGRRKAFSTCAGHLVVS

LLHNGCAIFIYIRPKSAYAPDQDKVVSLVYTNVTPMLYPMIYSLRNQEVQ

GALRRVLGRKIISWVS*

>OranORuc201.5P

MNELRGVNYTEVTEFILVGLSNQPKSQIIFFCILLHLYLGTIVGNILIIL

VVWKEPRLHTPMYFFLCNLSFVDLCSTTTAVPPALVNCLRDCPTITYNDC

FAQMTISLFWGITECCLLAVMAYDRFVAISNPLRYTLIMTMRACFQTAAT

MWISNFLLALIPVVTIPVRFCAGHNVVNHFVCELEAVLKLVCSDTTVSEI

LMLINSIFIFPLPFLFILLSYIRIVVAILKIPSTAGRRKAFSTCGSHLIV

VTIYYGTLISIYLKPQNKDSKDQDKIISIFYGTVIPMMNPLI/HMLRNKD

MIGALRKAVGKTKQVKLSI

>OranORuc201.6

MNELRGVNYTEVTEFILVGLSNQPKSQIIFFCILLHLYLGTIVGNMLIIL

VVWKEPRLHTPMYFFLCNLSFVDLCSTTTAVPLTLVNCLRDCPIITYNDC

FAQMTISIFWGITECCLLAVMAYDRFVAISYPLRYPLIMTMRACFYIAAT

MWISNFLLALIPVITIPVRFCAGNNVVNHFVCEVEAVLKLVCSDTTVNEI

LMLINSIFIFPLPFLFILLSYIRIVVAILKIPSTAGRRKAFSTCGSHLTV

VTIYYGTLISIYLKPQNKDSKDQDKIISIFYGIVIPMMNPLIYTLRNKDM

IGALRKAVGKTKQVRLSVCEF*

>OranORuc201.7

MVNANESRVNEFLLLGITRDPLEQKFLFGLFLLVYMVTLVGNLGLISLIR

MMPHLHTPMYFFLSNLSFLDFCYSSVTLPKMLADLLSERKTISFSGCVAQ

LSLFIIFATAELYLLASMAYDRYVAIRSPLLYPVLMSTRVCVSLVALCYV

AGIVNSLTVVSSIFQLSFCTSKTVNSFFCDIPPLLALSCSDTRTCEILVF

AFGGFIQLSSLLIVLISYLFIIITILKIRSADGKLKSFSTCASHLAVVSL

FYGTLIFMYLRPTSSYSLDHDRVVSVFYTTVIPMLNPIIYSLRNREVKEA

LKRAVYKGF*

>OranORuc253.1

MEEMNDTTVAEFFLVGFSQFPVLQRFVCWLCLLMYLIVLLGNSFLLVISI

VDSRLHTPMYFFLRNLSFLDICYTSTSIPQMLINFISKRKSISFIGCALQ

MFVSLGLGSTECILLAMMAYDRFMAIYSPLQYPIIMKKVVYVQMASWSWI

IGCLNALVQTILVMILPFCGNHTIDHLTCEILAVLKLVCADISLNVKIMM

LACVIFLLIPVLLIFTSYVFILSTILRMKSAEGRFKAFSTCSAHLTVVIL

FFGSALFAYMKPKSKDTKTLDELIGLSYGVVNPMLNPIIYSLRNKEVKEA

VRNVLSRNFLICVLLRGNRRSF*

>OranORuc285.1

MADGKDTSMKEFIILGLTDDPVLRIFLFTLFLVVYVVTVVGNLSIIILIR

ASSQLHMPMYHFLSHLAFIDFWYSSTVTPKMLKGFLVERDTISFSGCVAQ

LCSVFVFGTSEGILLAVMAYDRFVAICNPLLYTISMSNKVCAQLTATSYV

GGCLNSMLFTICVLGLAFCRSNEINHFFCDFPPLVELACSDVYLVQIISS

ISAAIIILSTVLTIVISYVHILCAIVRIQATGGRQKAFSTCTSHLTAVIL

YYGTIAFTYVQPSSSHSLEQNKVVSLFYTVVIPMLNPLIYTLRNKDVKGA

ALRIIDKYE*

>OranORuc285.2

MAQGNRTGITEFIFLGLTDQLQWQIILFMLFLLYYLVTLVGNLGLIAPIG

VDSRLRSPMYFFLSHLAFVDTCSSSTVVPKMLTDFFKEKKTISFLGCAAQ

MWFFGLFVAAECFLFSAMAFDRYVAIRNPLLYRSFVSPEVRVRLAVAPYV

LGFLSTTTHTTSTFRLPFCGPDVINHYFCDIAPLLALVCADVQASRWLLF

ISAGTVGVSSGLAILVSYLYILVAVSRIRSATGRRKAFSTCSSHLTAVAV

LYGTLFFIYVRPGASLSLHVNKVVSVFYTAIIPMLNPLIYSLRNKEVKDA

LCWTLQRKMVLYR*

>OranORuc285.3

MKKENFTYITEFIFLGLSVQPRVQLMLFIIFLFFYLLTLAGNVVIITVIC

MEPQLQTPMYFFLANLSFLDICYTSTNVPQMLSNLVGERKTISFTRCATQ

MYFSLSFGMIECILLGVMAYDRYVAICHPLHYTVIMNRAMCVQLGMTSWI

SSFLSSLVINALTSSLPYCGPNVVNHFFCEVPTVLRLACTDTTLPEMVVF

VFSIIIVFIPFLLIVVSYAHILLSVLRRQSTTGRRRAFSTCVSHLTVVAL

FYGTAIFVYMRPQSGASQASGKVIALVYTVITPMLNPLIYSLRNQEVKGA

LRKVIDRQKTPEC*

>OranORuc285.4

MLPQNHTTGLEFVLLGLSSQPELQVILFVVFLLIYLVTLVGNLGLILLIK

TDSLLHTPMYFFVSHLSLVDLCYSTNLSPQMLVHFLSEKKTISAAGCFIQ

CLVFIGLVITEFYILAAMAIDRFVAICYPLQYGRKMSKNACLFLVAVPYT

FGLLNGLSQTVLTFHLSFCSSRTINHFYCADPPLIVLSCSDTSIKEMAMF

IVAGFTLSSSLLIILTSYIFILAAILRIRSAEGRRRAFSTCGSHVTAVVV

FYGTLFGMYLRRPSKQSLEESKITAVFYSFVSPMLNPLIYSLRNKDVISA

LKKVTRRKLLNHMAGSMSAIF*

>OranORuc285.5

MSKMSSRNHTEVTEFILLGLKVQAELRPILFIMFLAIYLITVVGNVGMIV

LIQITPRLHNPMYFFLSHLSFVDLCYATNISPQMLVHFLSEKKTISFTGC

FIQFTIFISLAITEYYLLTVMAYDRYVAICSPLLYGSKMSRGVCVGLVMA

TYVYGFLNGLIQTALILRLSFCGSNEVNHFYCADPPLLVLSCSDTFVKET

AMFVVAGFNLICSLLIILISYGFIFAAILRIRSAEGRLKAFSTCGSHLTV

VTIFYGTLLCMHLTPPTERSVEQGKIIAVFYIFLCPMLNPFIYIMRNNDV

RDVMNSVLKFQLLTK*

>OranORuc285.6

MSRDNCTSVTDFILLGLTDRPELQAFLFVLFLIIYFFTLLGNLALIVLIR

LNSSLHTPMYFFLTNLAFVDLWYSSNATPKMLANFLSAKKTISFAGCFIQ

CYIFVALLLTEFYMLAVMAYDHYMAICYPLHYSIKMSRRVCTCLIAFPYV

YGFSEGLFQTISTFRLSFCGSNVMNHFYCADPPLIKLSCSDTHFKENAML

LSAGFNLTNSLTIIILSYIFILTAILKIQSSEGRSKAISTCGSHMMGVTL

FYGSLFCMYVRPLKDQSMEQSKMIAVFYTFVSPMLNPLIYSLRNKDVKAA

LRKMVNKRAILSFYNKS*

>OranORuc285.7

MPTPKEMSQRNDTLVTEFILMGLTDRPEIQPVLFVLFLFIYLITVVGNLG

LLMLIWLDSRLHTPMYFFLSSLSFLDLCYSTNVTPKMLMDFFSEKKTISL

ASCLVQCYFFIAMVITEYYTLAVMAYDRYVAICRPLFYHSRMTRAVCLRL

VVAPYIYGFLSGLMETMWTYRLTFCDSNTINHFYCADPPLIRLACSDTFI

KEMSMFIVAGFNLSNSLLIILISYVFILIAVLRTRSAEGRRKAFSTCGSH

LIVVTIFYGTLFCMYVRPPTDKSVEQSKIIAVFYTFVSPMLNPLIYSLRN

KNVKAAFWKIVRRNPLSK*

>OranORuc285.8

MGRGNYSGVTEFILVGLTDSIQMRAVLFVLFLPIYVLSVTGNLGLIGLIG

LSPRLHTPMYFFLSHLAFVDFCYTSSITPNMLVDLVVELKSISFLACAIQ

VCCFITFINVEIFLLALMAYDRYVAICHPLLYTTVMSWKLCTQLVAGTYL

YCFLVGFFHTFLTFRFSYCDSNVINHFYCDEVPLLALSCSDTRIKERLIF

AFAGFNTLGSLLIVLISYIFILLAILRIRSSEGRYKAFSTCASHLTSVTI

FYGTIIFMYLQPKSNHSLDTDKIASVFYTIVIPMLNPLIYSLRNQEVKGA

LKKKIGKISCMTPRVQFIK*

>OranORuc285.9

MAKGNHSQLSEFILTGLTTLPELQIPLFIVLLLIYCTTVLGNLGLIFLTR

TDSRLQTPMYFFLGHLALVDVGYSTAVGPQMLVNLFEERKSISFCLCTVQ

LCCFITFIITELFLLSAMAYDRYVAICNPLLYKLIISKKVCILLVTIPYI

YSLTVALFTVVFTFRSSFCGNNVIDHFYCDSLPILALSCSDTRVVEYFIL

GDSTFNLIFSLLVVLVSYIFIISNILKIRSAQGRCKVFSTCGSHLMGVTV

FYGTLIFMYMRPQASHSFETDKMASLFYTQVIPMLNPLIYSLRNREVKEA

LERTVTACWLHFKLLKS*

>OranORuc285.10P

DAHLQTPMNFFLSNLSFLDLCYSTVTMSKMLTNLLVEKVFSFAGCAAQMY

FFIALGTTEFFLLAAMLRDHL*SINISNYHIPQSVCPLDHWFLLGGFLHS

ALCMDFTFSLTFCGTNEINHFFCDITTLLSLSCSDPSLNELRLFNFVLFI

EIITVMAILTSYAAILLTVFRINSTRGKCQAFVLTCS/AAYFAAVSIYHG

MILFIYPRPCSSYSLETDELIAVFYTAMIPMLNPLIYSLRIQDVQGSLRK

ILMRN

>OranORuc285.11

MQMEYMAEKNLSYVTEFVLSGISNHPEWQIPLFLVFLIIYLITAIGNLGI

ITITNIDSRLKAPMYFFLRHLAFINLGNSTAIAPKMLVNFLAGNKTISYY

ECATQIGCFSTLIIAEIFMLATMAYDRYMAICKPLLYRVIISRGTCTLLV

ILIYTYSFTEGVIVSTHVFSVSYCSSNTINHFYCDHFPLLALSCSDTHIP

DTIIFAFSGSNLIFTLTVVLVSYFFIILAIVRIHSSEGRWKAFSTCTSHL

TAVTVFYGTLFFMYLQPQGSHSLDTDKMASVFYTLVIPMLNPLIYSLKNK

EVKGALKRLLTNSYQSFKIRM*

>OranORuc285.12

MHNDSTIIEFVLMGFPVPEELEIILFLLFLAIYLFTLVGNLGIVGLIRMD

TRLHNPMYYFLSVLSFLDACYSSVVTPKMLVNCLVGNKSISFSGCATQML

FFITFGTTECFLLAAMAYDRYVAISIPLLYTAFMSPRIYVSLIIGSYIGG

LLHGLLHTTATFSLSFCRFNEIRHFFCDIPPLLTLSCSDTHINELLLFSF

VSAIEVVTVLIVLVSYGYILAAILRIRSAQGRRKAFSTCASHLAGVTIYH

STILAAYVRPSSSYALEHDMVVSLFHTVLIPMMNPVIYSLRNKDVKDAFK

KVLEKHFQNETYKKQ*

>OranORuc285.13

MQMTRTTHNHSTIAEFVLMGFLVPEELQVMLFLLFLAIYLFTLVGNLGMV

LLIRMDSRLHNPMYYFLSVLSSLDACCSSVVTPKILLNCLVGNKSISFSG

CAAQLFFLVTFGTTECFLLAAMAYDRYVAVYSPLLYMAAMSPRIYVPLII

GSHIGGLLHSILHTAATFSLSFCGSNEIRHFFCDIPPLLSLSCSDTHINE

LLLFNFVSAIEVVTVLIVLVSYGYILAAILRIRSAQGRRKAFSTCASHLA

GVTIYHSTILATYVRPSSSYTLEHGMVVSLFYSIVIPMLNPLIYSLRNKD

VKEAFKKALKTVWFQ*

>OranORuc285.14

MDEGNYSAIAEFIFLGFSSDPDLQIILFVVFLMIYMITLLGNLGIIVLIK

IDAQLHTSMYFFLSNLASLDACFSCVIAPRTLINFLVERKTISYAGCVAQ

MGFFAALGTTECFLLAAMAYDRFIAICHPLHYSVMMSRRISVSLVVGSYM

YGFLNSVIHTVFTFSLSFCGHRTINHYFCDVPPLLSISCSNTSANQKLLF

VQISLNGLSTTGIIVVSYAYILFTILKIRSSEGKHKAFSTCASHIMVVAI

FYGTTCFMYLRPMSSYSLERDKRTSVFYTFVIPMLNPLIYSVRNKEMKDA

MRKVIERKPCSRW*

>OranORuc285.15P

QFIFLGIVDSEELQLILFILFLAVYVVTVLGNLGMMVLIWVDSRLHTPMY

FFLLSLSFADVCYSSTITPKMLVDLVSERKTISFVGCFLQMYVFIALATT

ECILFGLMAYDRYAAICNPLLYTVIMSRAICLKMIIGAYIAGFLNSVIHT

SYISSLSFCDSNVIQHFFCDSPPLLKLSCSDTHVNENIIFTCAGLNMLGT

LLIVLTSYSYILFSILGMHSAEGRQKAFSTCASHLTAVTIFYGTLIFTYL

RPSSSYSLNEDRVVSVFYTVVIPMLNPLIYSLRNKEVKEALQKLILRKRI

PVIV

>OranORuc285.16

MPDQNRTTVTEFIFLGIVDSEELQLILFILFLAVYVVTVLGNLGMMVLIW

VDSRLHTPMYFFLLSLSFADVCYSSTITPKMLVDLVSERKTISFVGCFLQ

MYVFIALATTECILFGLMAYDRYAAICNPLLYTVIMSRAICLKMIIGAYV

AGFLNSVIHTSYISSLSFCGSNVIQHFFCDSPPLLKLSCSDTHVNENIIF

TCAGLNMLGTLLIVLTSYSYILFSILGMHSAEGRQKAFSTCASHLTAVTI

FYGTLIFTYLRPSSSYSLNEDRVVSVFYTVVIPMLNPLIYSLRNKEVKEA

LQKLILRKRIPVIV*

>OranORuc285.17

MMAERNFTWMTEFIFVGFTDYQPLRVTLFLVFLGVYVLTLAGNIGIMILV

NTSSSLQTPMYYFLSNLSFLDISYSTAIAPKMLVNLLAAKRSISLYGCAL

QMYFFSCFADAECLILAAMAYDRYVAISNPLLYSTLMSRRVCVGFIVGAY

ASGSITSIVHVCLTFRLPFCGSNLINHFFCDIPPLLALSCADTHLNELLL

FLLCGFIQTSTFMIIFISYVSILITALNIKSSGARSKTLSTCVSHLVAIT

LFYGSLLFMYLRPITSYSLDTDKVVAVFYTVVFPMFNPIIYSLRNKDVKS

AVKKLLQRKCFSK*

>OranORuc285.18P

MAGSNHTSVTEFLLVGLTDRPELQMPLFVLFLIIYLVTMVGNVCMILLIQ

INTTLHTPMYFFLCNLALCDICYSTVLSPKMLINFFLESKSSSFIGCVFQ

SFFFAIYITTEGFLLAIMAYDRYTAIVNPLLYTAIMIPKICIQLVLASYL

GGLVNSLTHTVGLLRLNFCGPNVVNHFFCDIPPLLKLSCSDSHTNELLLL

TFSGVIATITLIIIVISYVQIVIAILRIGSVKGRYKTFSTCTSHLTAVIL

FYGSLSF/FSYIQPSSQYSLEQEKVSAVVYTLVVPMLNPLIYSLKNKGC*

KEGL*K

>OranORuc285.19P

LLRTHSRRG*SVKRALLCFPADCCFHFSMGPLIIMYFQPHSNSSRIQNRL

VSVFYTVFIPMLNPLIYSLRNKEVKSAFRKVIETKMLSLFFAD

>OranORuc285.20

MEEGNLTMWNGFILSGLTDQVDLQATLFLFLLLAYIVTVIGNLGMTMLIK

IEPRLHTPMYFFLSNLSFVDFCYSSCVTPRMLVTFLSETKAISFTACASQ

AYFFGAFGTTEIFLLAVMAYDRYVAICHPLLYTVIVSPRVCVFLASVTYF

GGFANSLIHTYLTFRLSFCLSNVISHFLCEIPLLLQLSCSDTSINFLVMF

ICVSFNILSTLWTILISYIYILAAILRTHSREGKWKAFSTCASHLTAVFI

FYGTLIIMYLQPHSNSSLNQNRLVSVFYTVFIPMLNPLIYSLRNKEVKSA

FRKVIETKMLSLFFAD*

>OranORuc285.21

MKEKNCTRVTEFILLGFTDQPDLEFIIFLLFWAIYLITMVGNLSIVLLIK

MSSQLHTPMYFFLSNLSLLDVSYSSVIAPKMMVNYLLAKNAISFAEYVTQ

FFLFSLAASAELYLLAVMAYDRFVAICKPLHYTFLMSKKSCILLVADSYL

CASVNAIAHTSALFSLSFCGSNVINHFFCDIPPLHTISSSATHVAASVHF

IFASIIIISTILAILLSYAFITAAILRIRSAEGRCKTFSTCASHLTAVSI

LYGTLIFMYLGPVSSLSAYQIKVMSMLYSLVIPMLNPLIYSLRNKEVKEA

LKKTLGWKLSVF*

>OranORuc285.22P

MEERNCTIASQFIFLGLTDWPELKLVVFLMFLTIYVTTLVG/GNLTMVTL

IEISPQLHSPMYFFLRNLSLLDVVYSSDIVPKVLINYLLETKVISFGECV

VQFFFLSLCVNTELFLLATMAYDRFIAICHPLLYTVMMSKEVCILLVASS

YLLSCVSAIVHTSSLFSLSFGSSNITDHFFCDIPPLHTISCSDTYIADLV

H

>OranORuc285.23

MANNNSSKVTEFILLGLTENWELQVIFFVLFLIIYIITVLGNLVIILLIR

VHSQLHTPMYFFLCHLSFSDTCYSSSVTLNMLVSFLSVKKSISYLGCAAQ

FCAIFTFGTNECLLLAVMAYDRFTAIRHPLLYTAMMTKKICTLLVASSYT

GAGINALVETTSVFSLSFCGPNVMNHFLCDFHPLLQLSCSDISLAKVLNS

FVSGIIVAITIPTVLISYLYILLAILKIPSTQGRRKAFSTCGAHLTAVAL

FYGTTTFVYVLPNSRDTLEQNKVFSVFYIIVIPMLNPFIYSLRNREVKGT

LRRTIDQRRITSGSYFI*

>OranORuc285.24

MKGETLMAAGNGTMVTEFILFGFTDDQNLQTTLFVLFLVIYTITLMGNLG

IIMLIRASPQLHTPMYYFLSHLSLSDMCYSSTVTPKMLENLRQGKRTISF

GGCALQFAVAAIFGTNECFLLAVMAFDRYSAICRPLLYPLIMSNKVRVQL

VLASYIGSCVNAVIFGSSVFSLSFCGPNEINHFCCDFPPLMELACSDTRV

AQILNSITSILIILVTILIIAISYLCILSAILRIPSTEGKHKAFSTCASH

LTAVTLFYGTLTFIYTQPNSRFSMDQKKVVSVFYMVVIPMLNPLIYSLRN

KDVKEALRRIFSSLKMSGLIYAMKKKLF*

>OranORuc285.25

MKGKTLIPTGNGSLVTEFILFGFTDDQKLQAILFGLFLVTYMITLMGNTG

IIMLIRASPQLHTPMYYFLSHLSLSDMCYSSSVTPKMLENLLQGKRTISF

GGCALQFAVAATFGTNECFLLAVMAFDRYSAICRPLLYPLIMSNKVRVQL

VMASYVGSCVNAAIFESSVFSLSFCGPNEINHFYCDFPPLLELACSDPRV

AQILNSITSVLISLVTILIIAISYLCILSAILRIPSTEGRHKAFSTCASH

LTAVTLFYGTATFIYTQPNSRYSMDQKKVVSVFYMVVIPMLNPLIYSLRN

KDVKEALRRVLIFDRCSD*

>OranORuc285.26

MATGNGSLVTEFILLGFTEDQKLQAILFGLFLVIYMITLMGNLGIIMLIW

ASPQLHTPMYYFLSHLSLSDMCYSSTVTPKMLENLLQGKRTISFGGCALQ

FAVAATFGTNECFLLAVMAFDRYSAICRPLLYPLIMSNKVRVQLVVASYV

GSCVNGVIFESSVFSLSFCGPNEINHFYCDFPPLLELACSDPRVAQILNS

ITSILIILVTILIIAISYLCILSAILRIPSTQGRHKAFSTCASHLTAVTL

FYGTLTFIYTQPNSRYSMDQKKMVSVFYMVVIPMLNPLIYSLRNKDVKEA

LRRIMIFKRCPD*

>OranORuc285.27

MANGNHTALSEFILSGLTSDPQLQIPLFMLFVLIYAVTLLGNLLIILIIR

LSSQLHTPMYFFLTHLSFSDSCYSSSVTPKMLANFLVDKKAIPYPGCVAQ

LCAAATFGVVEYCLLAAMAYDRYVAICKPLLYVVCMPQRLCALLVTASYL

ASCLNAVVCVASVFSLSFCGSHEINHFYCDVPPLLKLSCSDPRLAQVLPA

ASTAVIMLVTVLTTLISYVYILLAIFKIHSPGGRQKAFFTCASHLMTVTL

FYGTTMFIYLLPSSSFSMDQKKMVSVCYMILIPMLNPMIYSLRNKEVKDA

LWKVIALKKTVLFTSRIAH*

>OranORuc285.28

MANGNHTVLSEFILSGLTSDPQLRIPLFMLFLLIYMVTLLGNLLIVLIIR

VSSQLHTPMYFFLSHLSFSDSCYSSSVTPKMLANFLLDKKTISYPECVAQ

FCVAATFGVVEYCLLAVMAYDRYVAICKPLLYIVCMPQRLCASLVTASYL

ASCLNTVVYTANVFSLTFCGPPEINHFYCDVPPLLKLSCSDPGLAQVLPA

ATMAVIMLVTVLTTLISYVYILPAILKIQSSGGRQKAFFTCSSHLMTVTL

FYGTTMFIYLLPSSSFSMDQKKLVSVCYMILIPMLNPMIYSLRNKEVKDA

LWKVLVRERIG*

>OranORuc285.29

MANGNRTALSEFILSGLTSDPQLQIPLFILFLLIYMVTLLGNLLIVLIIR

VSSQLHTPMYFFLSHLSFSDSCYSSSVTPKMLANFLLDKKTISYPECVAQ

FCVAATFGVVEYCLLAVMAYDRYVAICKPLLYIVCMPQRLCASLVTASYL

ASCLNTVVYTVNVFSLTFCGPLEINHFYCDVPPLLKLSCSDPRLAQVFPA

ASTAIIMLVTVLTTLISYVYILLAILKIQSSGGRQKAFFTCTSHLMTVTL

FYGTTMFIYLLPSSSFSMDQKKLVSICYMILIPMLNPVIYSLRNKEVKDA

LWKVMAREWIG*

>OranORuc285.30

MANRNHTALSEFILSGLTSDPQLQIPLFMLFLLIYAVTLLGNLLIVLIIR

VSSQLHTPMYFFLSHLSFSDSCYSSSVTPKMLANFLADKKTISYPGCVAQ

LCAAATFGVVEYCLLAAMAYDRYVAICKPLLYVVCMPQRLCALLVIASYL

TSCLNTVVYTANVFSLTFCGSLEINHFYCDVPPLLKLSCTEPRLAQILPA

ASMAIIMLVTVLTTLISYVYILLAILKIQSSGGRQKAFFTCASHLMTVTL

FYGTTMFIYLLPSSSFSMDQKKMVSVCYMILIPMLNPMIYSLRNKEVKDA

VLKVMARKWIS*

>OranORuc285.31

MMANGNNTAVTEFILLGLTGDPNLQVIFFLLFLVMYSVTLLGNLVIIILI

RISSQLHTPMYFFLSHLSFSDTCFSSSVTPKMLVNFLAEKKTISYPACAT

QFWLAATFGFSECFLLAVMAYDRYVAICSPLIYTLIMSEKTCASLVAASY

VASFMNSLLCTYGVFSLFFCGPDQINHFYCDTPALLKLSCSDIQLAKIFQ

SSSAGITVIITSGTIITSYVYILISILRMPTKGGRRKAFSTCASHLLVVT

LFYGTVTFIYVMPNSDNSVDLNKMVSVFYMVMIPMLNPVIYCLRNKDVIE

ALKRMVLIKGLPNVKGESGQ*

>OranORuc285.32

MKGNQSRVTEFILLGLTDDPEVQTVLFVVFLMIYSFTLLGNLAITLVIKI

NSHLHTPMYLLLSHLSLSDIGFSSSVTPKMLVNLLAERKTISFPACAAQL

FIGALFGGTECFLLAAMAYDRYAAICKPLIYLALMSEKKCLGLVALSYLA

SVINALMCTNWVFSLWFCGPNEINHFFCDSPALLKLSCSDTHLAKIFPSI

SSGTVVTITIVIIVISYLYIVTAILRIHTTEGRHKAFLTCASHLMTVTLY

YGTVTFIYVRHSSNYSMDQNKVVSVFYLVVIPMLNPLIYSLRNKKIMGTL

RRMVSKRSLS*

>OranORuc285.33

MAGGNQTAVVEFILLGLTSDPVAQVILFGLFLVTYTVTLLGNCSIILLIR

IHSHLHTPMYFLLSHLSFTDMCISSTATPKLLENSLMGKTSISYMECAVQ

FFIASIFGPAECLLLAAMAYDRYVAICKPLAYPAIMSKRFCLLMVVDSYL

GSLLTAFVFTSNLFRLSFCGPNVINHFFCDFPPLLKLSCSDTTLAKIIPS

SFATLLILSTLAIIVVSYLYIMLAILRIKSSAGRGKAFSTCASHLTAVSL

FYGSSAFIYMLPKSKYSMDQKKVVSMFYMVVIPMLNPLIYTLRNKEVKEA

LKRTLGIKGIYYGQF*

>OranORuc285.34

MAGGNHSTTTEFILLGLTGDATLQAVLFVTFLGIYTVTLMGNLSIIILIR

VSSKLYTPMYFFLSHLAAVDIGYSSCVTPVMLESFLTDLYTMTLAGCVVQ

VFYLVTFGTTECFLLAAMAYDRYIAVCSPLLYSSKMSDKFCVLLVVASYI

GGFVNAWTFTSCLLNINFCGPNQINHFFCDLSPLLKLSCSDISVITIIPS

VSSGSIIGFTMLVIVISYIYIVLEILRMNSKEGRYRAFSTCSSHLTAVTL

YYGTITFIHVMPKSSYSIEQDKVVSVFYPIVIPMLNPLIYSLRNKDVQEA

LKKMIDRKRISLTIMMH*

>OranORuc285.35P

VVGENQTAVLEFILFGLTNDPLAQITLFGLF*SPAWSLCRGTLFFS*PLV

LHRCV*DPTTTSKLLENKFLEKKSISYMECAV*FFIINISGPAKCPLLTV

TACNQYIVVCPPLAYPVTMSKRLHVLRLVGSYLGNLSINRSVIFIKCLLC

TEHHIWGLYSFILIFSRPNAIIYFCDFLTLLTKIIPSAIEALIILPTLGT

QLLFLIDLFGQSQNKIICGS*KSVNSCASRLNTVTPSYGSSA

>OranORuc285.36

MTGRNQSTVTEFILLGLTGDATFRAVLFVTFLGIYIVTLMGNLSIILLIR

VSSKLYTPMYFFLSHLAAVDIGYSSCVTPVMLGGFLADLYTVPLVSCIAQ

LCSVVTFGTTECFLLAAMAYDRYVAICSPLLYSVKMSNKVCILLVVISYI

GGCMNSWIFTSCLLNVDFCGPNRINHFFCDFSPLLKLSCSDISVLKVIPS

ISSGSIIVVTLLVIVISYVSIVVKIMRMNSRDGKHKAFSTCTSHLTAVTL

FYGTITFIYVMPKSIYSINQNKVASLFYTIMIPMLNPLIYSLRNKDVKEA

LRKVINRNRLS*

>OranORuc285.37P

MAGGNQTAAMEFILLGLTIDPVVQIILFGLFLVTYMVIMLGN/LFFPQQA

YESSQPPLLSYLRTKLLEKKSISQMDCAV*FFITKISGQAKCSLFNCDGM

*SIPSHLSLLPYPVTMSKRP*W**API*EIL*SINQSYLLSTYCTWNTVF

GNFDLFILTFSGPNVIIIHFCDFPTLLQLPGSPKSFTKIIPSILGVLIIK

PTLV/FL*IWLTSLKIKSHEG*RKFFNTCASHLNMVTLFYGS

>OranORuc285.38

MAGRNQSAVTEFILLGLTGDAILQSVLSVTFLGIYVVTLMGNLSIIVLIR

VSSKLYTPMYFFLSHLAAVDIGYSSCVTPVMLGNLLADLQAIPLAGCIAQ

LCSVVTFGTTECFLLAAMAYDRYVAVCCPLLYSGKMSNKVCTLLVVASYI

GGCVNAWTFTSCLLNINFCGPNQINHFFCDFAPLLKLSCSDILVIKIIPS

VSSGSIIAVTVLVIVISYICIVLTILRMNSREGRHRAFSTCTSHLTAVTL

YYGTITFIYVFPKSSYSINQNKVVSVIYTILIPMLNPLIYSLRNKDVKEA

LRKAIDRERFS*

>OranORuc285.39

MADGNDTAVSEFIILGLTDNFILRIFLFIFFLVVYVVTVLGNFSIITLIG

VSSQLHTSMYHFLSHLAFIDFWYSSTVTPKMLQGFLVERNTISFSGCAAQ

LCSVFVFGTSEGILLAMMAYDRYVAICNPLLYAISMSNKVCIQLIAASYL

GGCLNGVLFTSFVFSLAFCGPNEINHFFCDVPVLMELSCSDIHLIQIISS

ISAAIIILSTVLTVLISYVYILITIMRIQASDGRQKAFSTCTSHLTAVIL

YYGTLAFTYMQPSSSHSLERNKVVSLFYTVVIPMLNPLIYTLRNKDVKGV

VIKIMGKNVCSL*

>OranORuc285.40P

TLFSHLAAIIILSTVPTIIISYVYILCAIMRIQASDGRQKAFSTCASHLT

AVILYYGTITFTYVQPSSSRSLEQNKIVSLFYTVVIPMLNPLIYTLRNKD

VKRALMKIMGKNASSW

>OranORuc285.41

MAEGNFTSVSEFLILGLTDDPILRLIFFLIFLLVYSVTIMGNLCIIMLIR

VSSQLHTPMYLFLSHLAFIDIWYSTSVTPEMLKGFLVGRDTISYAGCAAQ

LCSAFAFGTSEGILLAMMAYDRYVAICNPLFYAISMSNRVCVQLITVSYV

GGCLNALVFTTFVFNLVFCGPDEIDHFFCDFPPLMEISCSDIHLIQTLSS

ISAAIIILSTVPTIIISYVYILCAIMRIQASDGRQKAFSTCASHLTAVIL

YYGTITFTYVQPSSSRSLEQNKIVSLFYTVVIPMLNPLIYTLRNKDVKRA

LMKIMGKNASSW*

>OranORuc285.42

MDLWKNHTSATEFILSGFSSYSDVQGTLFMAFLVVYVFTLLGNLGMIILI

RKDSQLHLPMYFFISNLSFIDLCCSSNITPNMLVNCLAERKVISYSGCVT

QLCLFAAFVTIECYLLAIMAYDRYLAVCNPLLYPTIMSQHLCLELIIGSY

VAGILNSALQTFSVFRLSFCSSNVIHHFFCDIPKLLKLSCSSTFLSEILS

SFFSVVVTMSTIVPILASYILILSSILKIQSLDGRFKAFSTCASHLTAVS

LFYGTGIFVYVQPMLNSAVDTDQGISIFYTVVIPMLNPMIYSLRNKVVRS

AWQKMFVTEKQYRERQL*

>OranORuc285.43

MDGNNGTMVTEFILVGFRDHPELQIPLFLVFLLIYATTVLGNLGMIVLIQ

MDSHLHLTMYFFLTGLSFVDICYSSSIAPRALVNFFEWGETISVAGCAGQ

LFFFAGFASTESFLLAAMAYDRYVAICNPLLYETVISRRVCAGLVAGSYL

AGFANATLLTSCTFQLTFCGPNVIHHYFCNVLPLLQLSCSDTRINEILLS

TGAGLIQISTVMTLLSSYLRILIAILKIRSPEGRRKAFSTCSSHLVAVAI

FYGTTFFIYLRPDSSSSLDEDRALSIFYTLVIPMLNPLIYSLRNREVKDA

LRNAMERRVACRRPCNSV*

>OranORuc285.44

MARRNLTMLTEFILLGFTDQLDLQILLFVIFLVIYLITLIGNLGMMGLIR

ISSRLHTPMYFFLSHLSFVDICYASSIAPKLLANVFVKIKTISFSGCFTQ

MYFSSALVTTECFLLAAMAFDRFVAICNPLLYAVIMSNRVCSELALAAYI

YGFLNSLIQTAMTLQASFCNSNAINHFYCAHPPLLSLSCSDIHVKEKQIF

FISTLNLVSSLLVVLLSYCCIFVVVLKFPSREERCKAFSTCVTHLTVIAL

FYGTLFSMYFQEPYTNHALPYDKAASVFYSLIVPMLNPLIYSLRNRDVKE

ALRKVIERKVWG*

>OranORuc285.45P

SRGIQNTMCEICLQRCKDLPELQESLPWLFLIFCTIIIRENLEMFMFIKL

DRHLHPITNCFLGYLLPLDLCHSSSLTPMLLKMLLMKKINTSPSIYFPQL

HWPYALAVSECYLLATVVSDRGLAIYTPLVHSATVFRSLCHGLMVGISIC

DFLTSVVQTFQFFIFDFNTNHIQIIDLQVDALTGLVVYTNEDRLLVILTA

HFITSVLTVLIPYDLIFATLLKILSLKGRHKFCSTFSFHLSVAF*FCETY

FFGYVNEHNSSHSLNYDKKASLFYRLIIPILNPLYTLDIIDSMRRSSV

>OranORuc285.46

MARGNYSTVTEFVLMGFSDHPELQLPLFLVFLVIFLATLLGNLMMVLLIQ

VSSPLHTPMYYFLSHLSFIDLCYSSSIAPKMLQDFLRKKKTISFAGCFAQ

MYFSSAFSTTECFLLATMAYDRYMAICKPLIYTTIMTKRVCRELMIGVYT

YGFLYSVIQTVLTFQLSFCDSNIIHHFYCADPPLLALSCSDILAKRNQLL

IFSALNLSSSLLTILVSYVCILVSILKIPSSEGRCKAFSTCASHLTVVTI

FYSTLFFMYLRQPRIGDSWNYNKEVSVFYSLVIPMLNPLIYSLRNAEVKA

TLTKLLDGKALR*

>OranORuc285.47

MLKPLAAGNDTGMANFILMGLTDSPRAQLVLFVVFLSIYMISVVGNLGLI

LIIQKDTQLHSPMYFFLCGLSFVDLSYSTAITPKTLVNLVTSRKKISFIG

CFAQMYFFISLAGTECFLLSAMAYDRYVAICNPLLYSSVMSSRLCMWLMV

GSYGVAFTNSLISLLFIARLPFCNSNVIHHFFCDTSPILVLSCTDTHNTE

IMIFILAGSTLAMSLITIAVSYMAIVSAVLKIDSTVGRKKAFSTCASHLT

GVTIFYGTLIFTYLKPRKSYSLGKDQVASVFYTIVVPMLNPLIYSLRNKE

VKNAISRMIHLIQPEVGQQAEVTRWRRKGKSFGRRPGRRALVQDFKPRLS

VWPPPSLWSSPLAFPPSVPFGAPAGVVRVPPPLPPPVATPGLSRVAQTGL

KPP*

>OranORuc285.48

MTQIKEMDQGNISAVTEFILMGLTNLPEFQLLLFSVFLAIYLISLLGNLG

LIVLIAVDSRLHTPMYFFLGNLSALDLSYSSAIAPKMLAGFFLEARTISL

IECAIQMYLFVACVTTECYLLAVMAYDRFMAICNPLLYMVIMSPRVCIQL

MLGSYLIGFLQGLVQVLLIFRLPFCKSNIINHFFCDLPPILKLSCSNTFP

NEVLLFSLGFFNGTVTSLEIMVSYVYILITILKIRSAAGRSKAFSTCASH

LTAVGLLYGTAIFIYVRPSSQYAPENDKVVSVFYTLVIPMLNPLIYSLRN

KDVKDALWKITHRKALSWRM*

>OranORuc285.49

MSPGGETFGGNHSSVAEFILLGLTDNTRIKIACFMTFLGMYLVTLVGNLG

MILLIWIDPRLHSPMYFFISHLSLVDLGYSSAVTPHMLSDFLRKGKSISF

SGCAAQMYFFVISASTECYLLAAMAYDRYVAICNPLLYVVTMSPKICAQL

VIGCYFMGFVNATTQTVLTFRLPFCASNVINHFFCDLPPLLELSCSDTHM

NETVLLLFAIFLGVFTSLEILLSYIFILATILRIGSAEGRRKAFSTCTSH

LAAVTIFYGTTVFMYMRPSSSYSLDSDKVTSVFYTAVIPMLNPVIYSLRN

KEVKNAMSRLIYQGRGH*

>OranORuc285.50P

EMMRGNRSTVTDFIFLSFSNFPKLQFLLLVVFLIIYLITLVGNTVILFII

TLERSLYIPMYLFMSILSILETCYSGMIISKMLMILSMDHKTISFAGCAV

QMCFILFLGVTDCFLLSAWPMITT*PSAIPCYPILMNKADVTKLTGGSVI

SGSVIAIIQTPWVFSFPFCGCNKTDLLLRETPSVLELVLGDTFLFEIYSY

IGTISVALLPFLMILLSYTRILYTILKMSSTAGRQNAFSTCASVTLFCGP

VNLSFLQPKSSYTAESKELLS/KNCSLAYDLLTSLLNPLIYSL*SSETKD

ALKKILRRK

>OranORuc285.51

MEKGNCTMVTEFILLGFSDHPELQLFFFLLFLVMYGITVVWNLGIIVLIK

ISSQLHTPMYFFLSHLSFVDLCYSTTIVPKMLTNIVTQSHTISYPGCLVQ

FYLFCTCVVTEVILLALMAYDRFVAICYPLQYMVTMSPNLCIQLVTGSYL

CGMVCSTIHTCLALKVSSFQSKNVNHFFCDLPTLLSLSCSEVSLNKLLLL

TVASFNEIGTILIILISYGFILITILRIHSTEGRRRAFSTCASHFTAIAV

FHGTILFIYCQPGTGKNMDIDKVAAVFYTVVIPMLNPLIYSLRNKDVKHA

LRKWIGPKILF*

>OranORuc285.52

MLYPKPAAEMGRGNHSSVTEFLLLGFSKLPELHFLLFMLFFIIYLIILMG

NVLIILVTTVDHILQTPMYFFLRNLSFLEICFNSVTVPKVLVILSTEDKS

ISFAGCFAQMFFFLVFGAGECFLLGAMSYDRYLAICNPLCYPILMNRHMF

ISLAGGSWCLGLMVAIIQTVWIFNFPFCGPNGIDHFFCDTPPVLELVCAD

TYLFELYALTGTILVVMVPFLLILLSYVHIISTILKMSSAEGRKKAFSTC

SSHLTVVTLFYGAIILTYLRPKTGYSAESKKLLTLSYILLTPLLNPLIYS

LRNSEVKEALRRTLCQKTYLRD*

>OranORuc285.53P

ATEMSSINETSVTEFFLLGFSNFPELQFLLFVSFFIIYLMILM*NLLIIL

IITVDCTLHSPMYFFLRSLSFLEICFDTVTVPKIQTFQPAHFQFLIPAST

FPVLFFCYV

>OranORuc285.54

MEKGNCTMVTEFILLGFSDRPELQLFFFLLFLVMYGITVVWNLGIIVLIK

ISSQLHTPMYFFLSHLSFVDLCYSTTIVPKMLTNIVTQSHTISYPGCLVQ

FYLFCTCVVTEVILLALMAYDRFVAICYPLQYMVTMSPNLCIQLVTGSYI

CGMVCSTIHTCLALKVSSFQSKNVNHFFCDLPPLLSLSCSEVSLNKLLLL

TVASFNEIGTILIIFISYGFILITILRIHSAEGRRRAFSTCASHFTAIAV

FHGTILFIYCQPGTGKNMDIDKVAAVFYTVVIPMLNPLIYSLRNKDVKHA

LRKWIGPKILSE*

>OranORuc285.55

MLYPKPTAEMGRGNHSSVTEFLLLGFSKLPELQFLLFLLFLIIYLIILMG

NVLIILVTTVDHILQTPMYFFLRNLSFLEICFNTVTVPKILVILSTENKS

ISFAGCFVQMFFFLVFGAGECFLLGAMSYDRYLAICNPLRYPILMNRHMF

ISLAGGSWCLGLMVAIIQTIWIFNFPFCGPNGIDHFFCDTPPVLELVCAD

TYLFELYALMGTILVIMVPFFLILLSYVHIISTILKMSSAEGRKKAFSTC

SSHLTVVTLFYGAAILTYLRPKTGYSAESKKLLTLSYILLTPLLNPLIYS

LRNSEVKEALRRTLCQKTLTSSSKEILS*

>OranORuc285.56

MEQGNLTVEEFVLLGFLDLPKLREFLFGIFLFSYMIILAGNGLIIIITAT

DQTLHTPMYFFLRNLSFLEICYTSVTLPRMLMSLQTQDGTISFLSCAAQM

CFLLMLGATECFLLAVMAYDRYVAICNPLRYPIIMSHKACVLLAAGSWIS

DIPVQIGQTSQIFSLPFCNSNKLNHFFCDIPPVLELACGDTSMNQLSVYA

AALLFVTAPFLLILVSYVKIISTILKLPSATGRRKAFSTCSSHLIVVTSF

FGSAIITYLWPKSSHSVDVDKFLSLFYTTVIPLFNPMVYSLRNKEVTVAL

KKFLSQRFVS*

>OranORuc285.57

MQVKETNQSHVTEFILLGFSNLHEFQVILFMIFLVIYLIAVIGNSLLILV

STVDPTLQTPMYFFLRSLSLIDISYTTVIIPKMLINLLSKDKSISFDGCA

AQMYFSFFFGSSECWILTTMAYDRQAAICDPLHYSLIMNRRFCRQLALAF

WLAAIPVATVQTTMMFTLPFCGPNVINHFFCDSPPLLELVCMDTFAIEVY

SVTGTVIVLLLPFGVITASYVRILVTILKMSSAEGRRKAFSTCSSHLIVV

SLFFGAAGSTYFRVKSSFSPESKKLLSLSYSVFTPMLNPLIYSLRNQEVK

GALKRILGKKIVSQDLRGSDLSK*

>OranORuc285.58P

MRMKEGTQSHVTEFILLGFSHIQEFQIILFMIFLVIYLITLIGNTLLVLV

STVDPTLHTPMYFFLRNLSLIDIGYTTVIIPKMLNNFLSKNPSISFGGCA

AQMYFSFFLDP/FLLFFGSSECWILTTMAYDRQVAICDPLRYLLVMNQGR

CLHLAMASWLSGIPLATVQTTMMFTLPFCGPNVINHFFCDGPPLLELVCT

DTFAFEVYGVTVTVTFLMFPFGVIIVSYVHILVTILKMSSAEGRHKAFST

CSSLLIVVSLFFRAASLTYFRVKSFYSPETKKLLSLSYTVFTPLLNPLIY

SLRNQEVKGALKRIL

>OranORuc285.59P

SLMDICYTTVIILKMLTNFLSKSPSISFGGCAAQMYFSFFFGPSECWILT

TMAYDRHAAICDPLCYSLIMNQRPC

>OranORuc285.60

MKEGNQSHVTEFILLGFSNLHEFQVYLFIIFLVIYLIALIGNSLLVLVSS

VDPALQTPMYFFLRSLSVMDICYTTVIIPKMLTNFLSKSPSISFGGCAAQ

MYFSFFFGPSECWILTTMAYDRHAAICDPLRYSLIMNRRLCLQLALASWL

SGIPVATVQTTMMFTLPFCGPNRIDHFFCDSPPLLELVCMDTFAFEVYGV

TATVTFLMFPFGVIIASYIHILITILKMSSAKGRRKAFSTCSSHLIVVSL

FFGAASMTYFRVKSSYTSESKKLLSLSYTVFTPMLNPLIYSLKNQEVKVA

LKRILGKKDIFPGSGRL*

>OranORuc285.61P

SLMDICYTTVIILKMLTNFLSDTPSISFGGCAAQMYFSFFFRLSECWILT

TMAYDLHAVIRDSLCNSLIMNQR

>OranORuc285.62

MRMKEGNQSHVTEFILLGFASLPEFQVILFMMFLMIYLIAVIGNSLLVLV

STVDPALQTPMYFFLRSLSLMDIGYTTVIIPKMLTNFLSKDKNISYGGCA

AQLYFSFFFGPSECWILMTMAYDRQAAICDPLRYSLIMNRKFCLQLSLAS

WVAGIPVATVQTAMMFTLPFCGPNVINHFFCDSPPLLELVCTDTFANEVY

SVIGTVIILILPFGVIIASYIRILVTILKMSSTESSRKAFSTCSSHLIVV

SLFFGAVGSTYFRVKSSYSPESRKLLSLSYSVFTPMLNPLIYSLRNQEVK

NALKKILCRWIFSKGLRGSDL*

>OranORuc285.63

MKLTRQNHTTVTEFVLMGFTNAPKLQTFLFATFLVIYMMILMGNSLIILI

TKTDRALHTPMYFFLRNLSFLEVGYTSVTLPRMLANLWTQTRTIPLVACA

AQMYFFLMFGATECFLLAVMAYDRYVAICSPRQYSIIMDDKACLKMAAGS

WISGIPVQLGQTYQIFSLPFCDSNKLNHFFCDIPPVLELACGDTAMNELS

VYAVALLLVTAPFLLILVSYVKIISTILKLPSAMGKRRVFSTCSSHLIVV

TSFFGSAIITYLRPKSSHSVHVDKFLSLFYTTVTPMFNPMIYSLRNKEVT

VALKKFLSQLF*

>OranORuc285.64

MRMREGNQSLVTEFILLGFSNLHEFKVILFMLFLVVYLITLIGNSLLVLV

STVDPTLQTPMYFFLRSLSLMDMGYTTVIIPKMLTNFLSKDKSISFGGCA

TQMYFAFFFGSSECWILTTMANDRQAAICDPLRYSLIMNRRFCLQLSLAS

WLAGIPVATVQTTLMFTLPFCGPNVINHFFCDSPPVLDLVCTDTFALEIH

SVTATVIILMLPFGVIVVSYIRILVTILKMSSAEGRRKAFSTCSSHLVVV

SLFFGAAGSTYFRLKSTYSPETKKLLSLSYSVFTPMLNPLIYSLRNQEVK

GALKRILGKKISSQDL*

>OranORuc285.65P

MQMREGNQSLVTEFILLGFSNLHEFQVILFMLFLVVYLVTLIGNSLLVLV

STVDPSLQSPMYLFLRSLSLMDIGYTTVIIPKMLTNLLSKDKSISFGGCA

AQMFFAFFFGPSECWILTTMAYDRQAAICDPLRYSLIMNRRFCLQLSLAS

WLAGIPVATVKTTLMFTLTFCGPNVINHFFCDSPPVLDLVCTDTFAVEIY

SVTATVMVLMLPFGVIVVSYIRILVTILKMSSAEGRRKAFSTCSSH/ARL

IVVSLFFGPAGSTYFRVKASYSPETKKLLSLSYSVFTPMLNPLIYSLRNQ

EVKGALKRILGRKISSQDL

>OranORuc285.66P

WGLCCPDVFRLLFSGPRSVGSLTTMAYDRQAAICDPLRYSLIMNRRFCLQ

LSLASWLAGIPVATVKTTLMFTLTFCGPNVINHFFCDSPPVLDLVCTDTF

AVEIYSVTATVMVLMLPFGVIVVSYIRILVTILKMSSAEGRRKAFSTCSS

HLVVVSLFFGAAGSTYFRVKASYSPETKKLLSLSYSVFTPMLNPLIYSLR

NQEVKGALKRILGRKISSQDL

>OranORuc285.67

MQMREGNQSLVTEFILLGFSNLHEFQVILFMLFLVVYLITLIGNSLLVLV

STVDPSLQSPMYFFLRSLSLMDIGYTTVIIPKMLTNLLSKDKSISFGGCA

AQMFFAFFFGPSECWILTTMAYDRQAAICDPLRYSLIMNRRFCLQLSLAS

WLAGIPVATVKTTLMFTLTFCGPNVINHFFCDSPPVLDLVCTDTFAVEIY

SVTATVMVLMLPFGVIVVSYIRILVTILKMSSAEGRRKAFSTCSSHLVVV

SLFFGAAGSTYFRVKASYSPETKKLLSLSYSVFTPMLNPLIYSLRNQEVK

GALKRILGRKISSQDL*

>OranORuc285.68

MKLTRQNHTTVTEFVLMGFSNAPKLQTFLFATFLVIYMTILMGNSLIILI

TTTNRALHTPMYFFLRNLSFLEVGYTSVTLPRMLANLWTQTRTIPLAACA

AQMYFFLTFGATECFLLAVMAYDRYVAIYNPLQYSIIMDDKTCLLMTAGS

WIGGIPVQIGQTYQIFSLPFCDSNKLNHFFCDIAPVLKLACGDTAMNELS

VYAVALLFGTAPLVLILISYIKIISTILKLPSATGRHKAFSTCSSHLIVV

TLFFGSGIVTYLWPQSSHSADVDKFLALFYTTVIPMFNPIIYSLRNKEVT

VALKKFLLQQF*

>OranORuc285.69P

YVTIRNP*SYPLIMSYRVCVQLGVAMWLSLLPVQKGQIY*LFTQPF/HKI

NYFFCDLLPVLKLTYGEIFPSKLLA*DVILLTVMVPFLLILVSYVKIISL

PRNCPQAMRR*KVYSTCPSHLIVIPKVLTVIPWVFGSPIITYSLAKSSHS

GDLDKLLSLFPTTTFNRLIFSLKKQEGTTTFK

>OranORuc285.70

MPEKNMPMREGNQSYVTEFILLGFSNLREFQVILFMTFLGIYLVALVGNS

LLVLVSMVDPSLQTPMYFFLRSLSLMDIGYTTVVNPKMLINLLSKDKSIS

FGGCAAQMYFAFLFGPSECWILTTMAYDRQAAICDPLRYSLIMNRRRCLH

LALASWLAGIPVATVKTTLMFTLTFCGPNVINHFFCDSPPVLDLVCTDTF

ALEVYSVTGTVIVLMLPFGLIIVSYIRILVTILKMSSAEGRRKAFSTCSS

HLLVVSLFFGAAGSTYFRVKSTYSPETKKLLSLSYTVFTPMLNPLIYSLR

NQEVKCALKRILGKKISS*

>OranORuc285.71

MENRNNVTEFVLLGLSSDRYLQIFCFGLFLSCYVAILLGNLLILITVRGS

SLIKQPMYFFLCHLSLMDVCYTSTVIPKLVRDLLSEKKTISFDDCMMQLF

TMHLFGGVEVFILVGMAYDRYVAICKPLQYMVIMNKQRCATVVGVCWGGG

FLHSIVQWLLVIFLPFCGPNKIDHYFCDVYPLLELACSDTDVTGYLVLAN

SGTIVLISFVVLVFSYVTILASLRTRSLEGRRKALSTCASHITVVALLFL

PCIFIYLRPAQTFPEDKVFALFYTVIAPMFNPLIYTLRNRAMKTAMRKVW

CCKLRLERKSCR*

>OranORuc285.72P

LLELACTGY*RDRIF*SSSIQGTIALISFLVWVFSYVTILASLRTRSSEG

RRKALSTCASHITVVALLFLPCIFIYLRAGPDLP/PAQTFPEDKVFALFY

TIIAPMFNPLIYTLRNREMKTAMRKVWCWKLTLEGKSAR

>OranORuc285.73T

MENRNNVTEFVLLGLSSDRNLQLFCFGLFLSCYFAILLGNLLILITVRSS

SLIKQPMYFFLCHLSLMDLCYTSTVIPKLVRDLLSEKKTVTFRACMTQLF

TMHLFGGVEIFILVGMAYDRYVAICKPLHYVVIMNKQRCATVVAVCWGGG

FLHSMVQWLLIIFLPFCGPNKIDHYFCDVYPLLELAC

>OranORuc285.74

MENRNNVTEFVLLGLSSDRNLQIFCFGLFLSCYVTILLGNLIILITVRGS

SLIKQPMYFFLSHLSLMDVCYTSTVTPKLVRDLLSEKKTISFGHCMMQLF

TMHLFGGVEIFILVGMAYDRYVAICKPLHYMVIMNKQRCATVVGVCWGGG

ILHSMVQWLLIIFLPFCGPNKIDHYFCDVYPLLELACSDTDVTGFLVLAN

SGTIVLVSFAVLVFSYITILASLRTRSLEGRRKALSTCASHITVVALFFL

PCIFIYLRPAQTFPEDKVFALFYTIIAPMFNPLIYTLRNREMKTAMKKVW

CCNLCLERKSCR*

>OranORuc285.75P

MENRNNVTEFVLLGLSSDRNLQIFCFGLFLSCYVTILLGNLVILITVRGS

SLIKQPMYFFLSHLSLMDVCYTSTVTPKLVRDLLSEKKTISFGHCMMQLF

TMHLFGGVEIFILVGMAYDRYVAICKPLHYMVIMNKQRCATVVWSLAGEE

DFSISMVQWL

>OranORuc285.76

MENSNNITEFVFRGLFPNEEGRVTCFVLFLLCYLAILLGNLLILITIRNS

HLSQQPMYIFLSSLSVMDLCFTSTVAPKMIADLLVERKTISYNSCMVQLF

GAHFFGGAEIFILVAMAYDRYVAICKPLHYLIVMNRQTCRSLVLASFVGA

FLHSISQIFIMVRLPFCGPNQIDHYFCDVFPLLKLVCTETYFANVSIIVS

TGVLSLLTFFALIVSYLIILSVLRKHSIEGRQKALSTCGSHITVVLMFFL

PLIFTYVPVADSVSEDKVFALFYTIIAPMFNPLIYSLRNTEMKNAMRKVW

YRKVLSERK*

>OranORuc285.77

MENQNNISEFILLGLSPNPSVQVICFGLFLLLYVGIVMGNLLIFLTIQFS

HLISQPMYYFLRSLSLVDLCYASTISPKLITDLMVERRTISFPNCMTQLF

ASHFFGGVEIFILVWMAYDRFVAICKPLHYTVIMNRRACNGMLLLACVVA

FVHSMAQLLLTLQLPFCGPNQIDHFFCDVNPLLKLACTDTYVVGLLVMAN

TGMIAMLTFVVLLISYIVILVSLRTHSPHSRRKALATCGSHITVVVLFFG

PCIFIYVRPATTFMADKVLAVFYTIVVPMFNPLIYTLRNTEMKSTIRKMW

CRRVFSRGK*

>OranORuc285.78

MEKINNVTEFELLGFSQNVEVQKACFVIFFLFYAIILLGNILIMLTVCLG

NLYRSPMYFFLNYLSLVDLCYSSVTAPKMIRDLISERKTISFEGCMMQLF

GVHFFGCTEIFLLTAMAYDRYVAICKPLRYMNIMNRSVCNKMLVGTWAGG

FIHSIIQVALVVQLPFCGPSVIDHYFCDVHPVLKLACANTHLAGVVVTAN

SGTIALGSFLILLFSYAVILWSLRQQSEEGRRKALSTCGSHIAVVIIFFG

PCTFMYMRPDTTFSEDKMVAIFYTIITPMLNPLIYTLRNAVVKNAMKRLW

SRRVALQE*

>OranORuc285.79

MAISNNVTEFILLGLTQDPEKQKVIFAVFLIFYLATLFGNFLIVVTIKTS

PTLESPMYYFLTYLSFSDACFSSTTAPKLIVDSLSDKKTITFGGCMTQLF

SAHFFGCMEILVLILMAYDRYVAICKPLLYTTIMNQHLCGVLVGLAWVGS

FLHSTAQLFLTLSLPFCGPNVIDHYFCDVQPLMKLACSDTHLANLLFVSN

SGAICTSSFVVLMASYVVILYSLRSKGAEGRRKALSTCSSHILVVTLFFG

PCIFIYTRPQTTFSVDKSVSVFYTIVTPLVNPLIYTLRTTEVKYAMGKLW

NRKVIPGKLLGRF*

>OranORuc285.80

MVIKNNVTEFILLGLTDDPAEQKIIFAIFIILYVATVLGNLLIIVTIKTS

QTLESPMYFFLTYLSFSDACFSTTTTPKLLVITLSEKKTISFNDCMTQIF

ALHFFGCLETLILIMMSYDRYVAICKPLHYTAIMTRRVCSILMGIAWFGS

FLHSSTQLLLTLSLPFCGPDMIDHFFCDLQPLMKLACVDTYYVNLLFVSN

SGAICTLSFVMLMASYMVILYSLRSKGAEGRLKALSTCSSHILVVTLFYG

PCIFIYTRPQTTFSVDKLVSVFYTIVTPLLNPLIYTLRNTEVKNAMKKLG

SHRVTPGANDHFSVE*

>OranORuc285.81

MAIKNNVTEFLLLGLTQDLKGQKIIFAIFLVFYIATLLGNLLIIITIKTS

QTLGSPMYYFLTYLSLSDSCFSTTTAPKLIVDSLSDKKMISFSECMIQIF

VFHLFGCMEILVLILMAYDRYVAICKPLLYAAIMNRRLCGVLVGLAWVGS

LLHSSVQLLLTLSLPFCGPNVIDHYFCDVQPLMNLACTDTHFVSLLFISN

GGVICTLSFVVLMASYVVILYSLRSKGAEGRRKALSTCSSHILVVTLFFG

PCIFIYTRPHATFSVDKSVSVFYTIITPLLNPMIYTLRNVEVKNAMKKLW

SRKVTPIDK*

>OranORuc285.82

MGSRNNVTEFILLGLSQKPEMQKILFVVFLFVYLVTVGGNMLIVTTITTS

HSLLGSPMYYFLAFLSFLDTCFSSVIAPKMIVDSLQERKTISYEGCMTQL

FAEHFFAGVEVFVLSVMAYDRYVAICKPLHYVTIMSRRTCGQLVGLAWAG

GFLHSMIQFFFMFRLPFCGPNVIDHFMCDLYPLLKLACTETRIPGLLVIA

NSGMICIMNFSVLITSYGVILCSLKSHSSEGRRKALATCGSHITVVIFFF

VPCIFIYTRPPATFSFDKMVAVFYTILTPLLNPLIYTFRNLEIKDAIRKL

WRRTVVFAEREMGLK*

>OranORuc285.83

MHDPEIKYNVTEFILLGLTQDPHLQKIFFVVFLVFFIFTVGGNLLIVTTI

TFSHTLSAPMYFFLTYLAFIDACYTCVTTPKMITDLLYRRRIISFNDCMA

QLYLAHFLGGSEIILLIVMAYDRYVAICKPLHYVITMSRRVCRLLVGVVW

MGGLLHATVQILFMFQLPFCGPNVIDHFMCDLFPLLKLACADTYVLGLVV

AANSGVMCMLILAMLLVSYIVILRSLKTQSSEGRRKALSTCGSHFTVVIL

FFVPCIFTYTRPVSTLAVDKLVTVFFAIITPMLNPIIYTVRNTEVKKAIR

NLWNRRVVMAAR*

>OranORuc285.84

MEQMENHTVTEFILLGLTQNSEMRQIFFVTFLIIYIVTLLGNMLIVVTIG

SSKALSTPMYFFLSHLSFIDASYSSSSAPKMLIDLSSGEKTITFNDCMTQ

VFTEHLFAGAEIVLLMAMAYDRYVAICKPLHYTAIMSQRLCSLLVGVAWT

GGFLHATIQVLFMVQVPFCGRNTMDHFMCDLFPLLTLSCSETQVLGLLVV

ANSGAMCMLSFFLLVVSYIVILHSLKAHVSSGRRKALSTCVSHITVVILF

FVPAIFVYVRPVATHPMDKYVAVFYTLITPMLNPFIYTVRNSEVKNAMMK

LWNRKVK*

>OranORuc285.85P

KMENRNLTEFILLGLTQNLESQKILFITFLSIYIATVVGNLLIVVT/IMA

SPTLDSPMYFFLAILALIDASYSSSIAPKMLVDFLHDRKTISFNDCITQL

FVVHFLGCSEVIILIATAYDCYVAICKPLHYLTIMNRHLCCLLVVLAWTS

GFLHSILQIVVTFHLPFCGPNVMDHFMYDMIPLIKLTCSETYIFGILVVS

NGGLIAMLSFVIFVSSYVVILHSMRNHSSAGKRKALSTCTSHITVVIFFF

VLCIFMYARPVATYPMDKAVSVFYTSTTPMLNPLIYTVRNAEVKNAMGKL

*GRRV

>OranORuc285.86

MENRKNVTEFILVGLTQSPDMQKLFFVVFFSVYIATLVGNLLVVVTIISS

STLGSPMYFFLAYLSLIDTCYSTSITPKMLVDLLSMRKTISFNRCMAQLF

IEHLCAGAEIVLLLAMAYDRYVAICNPLHYITIMRQTVCSLLVGVAWGGG

FLHATLQILFMSPLPYCGPNVIDHFMCDLFPLLKLACTDTHIFGLLAVAN

SGAMCTLSFFLLVISYVVIFRSLKNQSSPGRRKALSTCVSHVIVVIFFFF

PCIFTYMRPVSTFPIDKSVAVFFTIITPILNPIVYTVRNAEVKSAMRKLW

SPKATSGCK*

>OranORuc285.87

MFFTENMEKKNNVTEFVLLGFTQSPEIQRTFFVIFLLSYLVTVMGNLLIV

IAIKASKTLGTPMYFFLAYLSFIDTCYTSCVAPKMITDLLQERKTISFNG

CMSQLFAEHLFAGAEIILLMVMAYDRYVAICKPLHYMAIMSQRMCGLLVG

TAWMGGFFHASIQVLFTVQLPFCGPNVIDHFICDLFPLLRLACTDTQVRG

LLVVANSGAMCTLSFLLLVFSYVIIWSSLRTHSSAGRRKALSTCVSHIVV

VILFFFPCIFMYMRPVYTLPMDKSVAIFYTIVTPMLNPVIYTVRNAEVKN

AMKKLWSRKVK*

>OranORuc285.88

MDYKNNVTEFILLGLSQNPEVQKVLFVVFFIIYMVTIVGNLLIVVTVIAS

QNLRSPMYFFLAYLSLMDAFYSTATAPKMIVDLLYEKKTISFKGCMTQLF

VEHLFGGAEVFVLVVMAYDRYVAICKPLYYMTTMNRRACGLMVGLAWVGG

FLHSMAQLLFMFRLPFCGPNVIDHFICDTYPLLELACTDTYLLGLSVAAN

GGAICVIIFLLLLLSYVVILRSLKSLSLEKRHKALSTCSSHLTVVVLFFV

PCIFMYMRPVCTFPIDKSVTVFFTIITPMLNPLIYTLRNAEVKNAMKKLW

RKKAILN*

>OranORuc285.89

MFTGMFITSTEVMEKRSNVTEFILLGLAQNPESQKLLFVLFLIIYLVTLL

GNLLVAITIEASQNLSSPMYYFLSYLSFIDAFYSSSTTPKMIVDSLSARK

VISYDGCMVQLFVSHFFGGTEVILLIVMAYDRYVAICKPLHYLIIMNRKV

CALLVGVAWVVGFFHSVIQVLFLFRLPFCGPNVINHFLCDMYPLLELACT

DTYIVGLSVAANGGLICTICFLMLVVSYMVILNSLKSYTLKIRQKALSTC

GSHVTVVLLFFIPCIFIYMRPASTFSIDKAVAVFYVFITPMLNPLIYTVR

NAEVKNAMRKLWSQIMTSGEK*

>OranORuc285.90

MGNENNVTEFILLGLTPNPKMQKVIFVVFLFIYIITVVGNLIIVVTITAS

QTLDSPMYFFLAYLSFIDACYSSVNTPKLIVDSLREKKIIYFKGCMTQVF

GEHFFGGAEGILLTVMAYDRYVAICKPLHYSTIMNRRVCGVLVGVVWMGG

LVHATIQILFMVDLPFCGPNVIDHFMCDLNPLLNLACTDTHTLGLFVAAN

SGLICLLNFVMLMISYIVILRSLKAHSKEGRRKALSTCVSHITVVVLFFV

PCIFVYMRPASTFPLDKAIAVFYTIITPMLNPLIYTLRNVQMKNAIRKLW

SRKVASEDK*

>OranORuc285.91P

MGNENSVTKFILLGLTRDPKMQKIISVVFLIYH/F*FIITVVGNLVIVLT

VASSQTLDSPVYFFLAHLSFIDACYSSVNKSKLIGESLSENKIIDFKGCM

TQVFGEHFFGGTEIILLTVMAYDRYVVICQPLHYTTIVSRCPCSLLVAVT

WAGSFLQATVQVLFMVHLPFCDPNVTDHFMCDLHPLLKLVCSDTHTLRLL

VAVNSGGMCV/CVLIFVLLMSSSVFILHSLKTHSAMGRLRALSTCASHFT

VRCHLLLYPMYF*ICAALNYLIIDKALAVFYTMITPMLNPFIYTLRNAEM

RNAIRKAVSK

>OranORuc285.92

MENENNVTEFILLGLTQNPGMQKIIFVVFLAVYSVTVIGNLLIMATINSS

KTLGCPMYFFLNHLSFIDMIYSSSSAPKLIADSIREKNTISFEGCMTQVY

AEHIFGGAEILLLTVMAYDRYVAICKPLHYPTIVNRRLCRLLVGVIWMGG

ILHATIQILLTVRLPFCGPNVIDHFMCDLYPLLKLACSDTRNLGLFVAAN

SGLICLFNFFLLTLSYVIILRSLKTHSLEGRRRALSTCISHITVVVLSFI

PCIFVYMRPMTTLPVDKTVAVFYTVVAPMLNPLIYTLRNAEVKNAMRKLC

RRKVSSHKINGPK*

>OranORuc285.93

MEKRNNVTEFIFLGISQNQVLQRIGFALFVFLYTVTLLGNLLIMITINVS

PRLTSPMYFFLGYLSFVDVCYSTTTTPKMMADLLAERKTISFGGCMTQLF

AVHFFGCTEIFLLTGMAYDRCVAICRPLRYPTIMSRRVCQIMVTTLWGAA

FLHSIVQTLLTVQLPFCGPNVLDHYFCDVHPLLKLACADTSVVGLMVVAN

SGMISLVAFFILVISYAIILFTLRTQSPEGRRKALSTCGAHIIAVFLFFG

PCLFMYIRPSRTLAADKMVTLFNTVMPPMLNPLIYTLRNAEVKSSMRKLW

R*

>OranORuc285.94

MEFMAATNNVTEFVFLGLSQNKEVQKVCFLVFLLLYTATVLGNLLIVVTV

NVSKNLGSPMYYFLSYLSFVEICYSSTTAPKLILDLGVEKKGISLNDCMT

QLFFIHFFGGTEIFLLTMMAYDRYVAICKPLHYTSFMSRRVCGLLVGMAW

MGGFLHSMAQVILVFRLPFCGSNVIDHYFCDLLPLLKLACKDTFVIGLLI

VANGGVLSAVSFLVLVVSYVIILLHLKTRGSRGRRRALSTCGSHIMVVIL

FFGPCIFIYMRPSATFSIDKMVAVFYTVITPVLNPIIYSLRNTEMKNALK

RLWVIKMKGGENERQNGKRIGDL*

>OranORuc285.95

MAHTNNVTEFMFLGLFQDWEVQKITFMVFLFVYTTTMLGNGLIVVTIKTS

KSLSSPMFFFLSYLSSVEICYSSTIVPRLITDSLAERKVISLEGCITQLF

FFHFLGVTEILVLTAMAYDRYIAICKPLLYAVIMNRRTCGILMGSSWAGG

FLHSMVQISVVIRLPFCGPNVIDHYFCDLHPLFKLACTDTSVEGVVVLVN

SGLISVISFLLLVSSYVVILRNLRSRSAEGRRKALSTCTSHITVVILFFG

PAIFLYLRPSSTYTEDKMVAVFYTVITPMLNPIIYTLRNAEVKNAMRRMW

SRK*

>OranORuc285.96

MENNVTEFVLMGLSRNAMEQQVYFVLFLLFYVIIIIGNLLIIVTIKGSSN

LNSPMYFFLSYLSFIDVCYSSVTAPKLIVDFQAKVKVISFIGCLAQLFGV

HFLGCTEIFLLTAMAYDRYVAICKPLHYTVIMNHRMCSILVVSSWLGGLV

HSVVQTLLIAWLPFCGPNEIDHYFCDVHPLLKLACTDTYIVGIIVVANSG

MISLSCFVILVVSYTVILLSLTTRSSEGRRKALSTCASHILVVILFFGPC

IFIYLRPSTTFSEDKMVALFYTIITPMLNPLIYTLRNAEVKNAIKRLWNR

RVMRKEN*

>OranORuc381.1

MTDFSPYRLKMMNRSFSQSFILLGFSNWPYLELVLFVIILIFYLLTLLGN

TSIILVSHLDPQLHTPMYFFLSHLSALDLCFTTSVIPQLLVHLSRADKSI

TFGGCAVQLYVSLALGSTECLLLAAMALDRFAAICHPLRYAVFMNPRLCR

QLAAGSWGCGFTASLLQTVLTMKLPFCGQAQVDGFLCEVPALLKISCVDT

TFIEMELFAATLLYLGLPVVLIFISYGCIARAVLRVGSGEGQRKAFGTCG

SHLLVVSLFYGTILAVYIVPQNRYAEARGKLLSLFYTVVTPTLNPLIYTL

RNQDVKGAMRRLLAQKKTVKLGMGPGWACWETSLG*

>OranORuc381.2

MVKNSTQSSLEGFVLLGFSDKPWLETPLFVIFLLAYVLALFGNISIILVS

RLDPQLNTPMYFFLSNLSFLDLCYTTSTTPQMLVNLWGPEKTISYGGCVT

QLYIFLALGSTECILLAIMAFDRYAAICRPLRYPVIMNPQRCMHMAAGTW

FSGFANSLVQSTLTVVVPRCGHQVLDHFFCEVPALLKLACTDIRINEAEL

NVLGSLLLLVPLTLIVGTYGFIARTVQRIRSAESRHKAFNTCASHLVVVS

MFYFSAISMYVQPPSSYSQERGKVMALFYGLLTPTFNPFIYTLRNKDVKA

ALRRALTKEFWVRGT*

>OranORuc381.3

MELSEANQSSGGDFILLGFSDHPGLEKVLFVVISIFYFLTLMGNTAVILV

SYLDSQLHTPMYFFLSNLSFLDLCFTTTTIPQLLINLWAPGKTISYQGCL

AQLYIALALGSTECILLAVMSYDRYVAVCQPLRYAVLMPLCLCRQLAALA

WVSGFSNSIVGTAVTLRMPLCGRRRLDHFQCEVPALIRIACGDTAPNEAE

MFAASVIFLLAPVSLIVTSYGHITRAVLRLQSAAGRVKAFQTGSSHLMVV

VIFFGTIAFMYLQPAKSTSKDRGRFVSLFYVVVTPCLNPLIYTLRNREVK

DAVRRLGRR*

>OranORuc381.4

MNRANESTPQGFILLGFSDRPQLELPLFVVFLVSYILTILGNATIILLCR

LDPRLHTPMYIFLTNLSLLDLCYTTSIVPQMLANLHRAQKMISYGGCVAQ

LFIFLALGSTECLLLAVMSFDRFMAICRPLHYPAIMHHRLCLQLAASSWL

SGFGNSALQSTWTLKMPRCGRRRVDHFFCEVPALLKLSCANTVPNEAELF

FISVLFLLIPVTLILISYGFIARAVLRISSVEGRRRAFGTCGSHLLVVSL

FYGTAIYMYLQPPSPTSLDRGKMVSIFYGIVAPMLNPVIYTLRNKDVHHA

FSGLVSRLSSLK*

>OranORuc381.5

MRSNGSSLGAFVLLGFSDQPKLELVLFVVVLIFYILTLVGNAIIILVSRL

DPQLRTPMYFFLTNLSFLDICFTSSSVPQLLFNLRGPDKSISYLGCAIQL

FMFLGLGGTECILLGVMAYDRFTAICKPLRYKVAMHPHLCLQLVVGAWAS

GIANSLAMSPVTLSLPRCGRYHVDHFLCEMPAMIRLACVDTTVVESIVFV

LAGCFTLLPLSLILVSYGYIARAVLRIKSTMGRHKALNTCGSHLTVVTLF

YGNIIYMYMQPGTSSSQDQGKFLTLFYNLLTPTLNPLIYTLRNKEVKGAL

KRLVRSSMA*

>OranORuc381.6T

LQSTWTLKMPRCGRRRVNHFFCEVPALLKLSCVNTVANEAELFFISILFL

LIPMTLILISYCFIVRSVLRIPLVKGRRRAFETCGSHLLVVLLFYGTAIY

VYLKPPSPISMEHGKMVSLFYGIITPMLNPIIYTLRNKDVHRAFLGLVSK

VPSL

>OranORuc381.7

MDHKNVSSHWVFILLGFSDRPWLEMPLFLMVLVAYLFTLVGNVAIILVSR

LDPKLDSPMYFFLSNLSFLDLCFTTTTVPQMLKNLWGPDKTISYGGCVAQ

FYIFHFLGATECILLAMMSFDRYVAICKPLHYLAIMHQRLSILLVATAWV

SGLANSLLQSSLTVQLPLCGNHVVDNFFCEVPVVIEMACVDTSFNVAMLS

VVGTFYALVPLSLILVSYSFIAVAVLRIRSSAGRLKAFNTCGSHVTVVSL

FYGPVIGMYVQPSADNSRDKAKLMTLFYSVVTPMFNPFIYTLRNKDVKGA

LKRLLGKSSQSRGRRGEA*

>OranORuc381.8

MVNVTTVTNFLLMGFSDIWELQLIHAALFLLVYLAALTGNLLIVAITTLD

RRFYSPMYFFLKHLSILDLCYISVTVPKSIANSLTNSCSIYYWGCVAQLF

LTIMLAISELFVLTVMSYDRYVAICRPLHYEVIMNRGACVQMAAATWFSG

GLLGAMYSAGTFTLSFCRTNMIQQFFCDVPSLLNISCSERHIVVDVSVAI

GMSFGFISFVSIIISYIYIFSTVLTFPSTESRSKAFSTCLPHLAVIMFFL

STGACAYLKPPSDFPSTLDLMVSVFYTVVPPTLNPLIYSLRNRDMKAALW

EVWG*

>OranORuc435.1

MVGRRNSTNVIKFILLGFSEPPQLQGLLFGVFLLIYLMTLSWNLGLITLI

RTNFHLHTPMYFFLSHLSFVDVCYSSSVVPKMLADFFKERKTISLMGCTI

QCFVFVGMGGTECCLLAAMAYDRYVAICDPLRYQAAMTQTLCVRMVVAAH

LGGFLTSLAETSSIFQLRFCGPNVIHHFFCDLPLLLDLSCSNSFISKVVN

WLMVFITGVTSGLIVLISYLYIITTVVKIRPVKGRSKAFSTCASHLTVVT

LFYGSGLFAYLHPGASHSANQGEAASLFYGAVIPMLNPIAYSLRNKEIKD

ALTKLKEAIDQWYLSNVHYVQSTVLRIWESPVNRIESRNVFPMCH*

>OranORuc435.2P

F*GIPEFQGLVFGVFLVICWTLGLISLIRTDSHFHTPKYFFLGHLSFVDI

CCSSSVVPKTLSDFFKKAKTTSFKG

>OranORuc435.3

MSCGRNTTVVTEFILLGFSDHPELKIFLFVLFLAIYLVTLTWNLGLTSLI

KVSPNLHIPMYFFLRHLSFIDMCYASSVIPKMLSDFFKEQKTISFLGCAT

QYFVFSGLGLTECCLLAAMAYDRYAAICTPLLYTTVMSPSLCRKMVAAAY

AGGFLSALTATDLLYRQDYCGPNVIHHFFCDLLPLLALSCSDTSTTQVVV

FVVGVVIGMMSVLVVFLSYGYITVAVLLIRSARGRYKTFTTCASHLITVI

LFYGSGFFVYMRLNSSYSESRDKVVSLFYTVVIPMLNPLIYSLRNREILD

TLKRVMEKKVVLFQCLSPF*

>OranORuc435.4P

LLALSCSDTSTTQVVVFVVGVVIGMMSVLVVFLSYGYITVAVLLIRSARG

RYKTFTTYTSHLITVILFYGSGFFVYMRLNSSYSENRDKVVSLFYTVVIP

MLNPLIYSLRNREILDTLKRVMEKKVVLFQCLALF

>OranORuc435.5

MSWGRNTTVVTEFILLGFSDHPELKIFLFVLFLAIYLVTLTWNLGLTSLI

KVSPSLHIPMYFFLRHLSFIDMCYSSSVIPKMLSDFFKEQKTISVLGCAT

QYFVFSGLGLTECCLLAAMAYDRYAAICTPLLYTTVMSPSLCRKMVAAAY

AGGFLSALTETGLIYRQDFCGPNVIHHFFCDLLPLLALSCSDTSTTQVVV

FVVGVVIGMMSVLVVFLSYGYITVAVLLIRSARGRYKTFTTCASHLITVI

LFYGSGFFVYMRLNSSYSESRDKVVSLFYTVVIPMLNPLIYSLRNREILD

TLKRVMEKKVVLFQCLALF*

>OranORuc435.6

MAGEGNGTTTIRFILLGFLSVPELQVFFFVMFLGIYLIAIAGNLGLFTLI

RSGDPHLHTPMYFFLSNLAFTDICYSSTITPKMLSDFFRKEKTISFLGCA

TQFFVFASLGGGECLLLSAMAYDRYAAICNPLLYTAVMSPRLCGQMVAGA

FMGGFLASSIQTYFVFQLHFCGSNIINHFFCDLPPLLALSCSDTFYCQVA

NISLTTTIGVTSVLVILVSYGYIADAILKIRSAQGRSKAFNTCASHLTAV

TLLYGSILFTYLRPSSSYSLNQDKVVSVLYSLVNPMLNPLIYSLRNKEIK

DALRRVREKTRSFSLPYLS*

>OranORuc435.7

MNEGWNTTSVTTFTFLGFTGLPQLQSLLFGLLLMIYLLTLSWNLGLIILI

RTDSRLHTPMYFFLGHLSFIDVCYSSSVVPKTLSDFFKEQKTISFMGCAA

QFFVFVGMGLTECCLLDAMAYDRYAAICAPLRYQAAVTRTLCQKMVAGAY

VAGFASSLIQTVSIFQLHFCGPNVIDHFFCDLPPVLALSCSDTLDSQVVN

FLVVVTVGGTSFLILLISYGAIGAAVLKVRSTEGRCRAFYTCASHLTVVS

LLYVPALFTYLRPSSSYSLGQDKVVALFYSVMTPMLNPLIYSLRNKEIRG

TLHKMAERKRGLSWN*

>OranORuc435.8

MENHTRLKEFSFLAVTEFRELEIFLFVAFLTVYVTAVLGNLLIVVTVTKE

FLLHTPMYFLLRNKAVLDIIFPSIMFPKFLVDLLSERKTISFNGCMTQIF

FFHFIGGADIFFLSVMALDRYLAISRPLRYVTLMNQETWMGLIMASWVGG

GLHSIVQIILMLSLPFCGPNVLQAFYCDVPQVLKLACTDTSTLELLMISN

NGLVTSLWFLLLLGSYAAILVMLRSRPGEGRSKMLSTCSSHIMVVTLHFV

PSIYIYCRPFTALPMDTAVSLTNTGVTPMLNPMIYALRNQEMRTAMRRLK

RRRDF*

>OranORuc435.9

MELGNHTRVSEFIFRGLTQSRELSFVLFVFLFVLYIITILANLLIIVTVT

WESRLHLPMYFLLRNLSILDISLSCITIPKVLVDLLSKRKAISFNGCLTQ

IFFFHLLGGTDIFSLSVMAYDRYVAISKPLHYVTIMSKGTCFGLVAASWV

GGFVHSVVQILLLLPLPFCGPNVLDGFYCDVPQILKLSCTSAFSTELLMI

SNNGMISSLWFVLLIGSYSGTLVMLRSHSGEGKKKGISTCTSHMTVVTLH

FVPCIYIYARPFTALPTDTAVSITLNGIIPVLNPLIYTLRNHEMMLAMKR

LKKRVTQSGRK*

>OranORuc435.10P

MDLGNHTRVSEFIFRRLTQSRELSFALFVLLFVLFVINMLANLLIIVTVT

WESRLHVPMYFLLRNLSILDISLSCITIPKVLVDLLSKRKAISFNGCFTQ

LFFFHFLGGADIFSLSVMAYDRYIAISKPLHYVTIMSKETCFGLVAASWV

GGFVHSMVQILLFLPLAFCGPNVLDGFFCDVPQILKLSCTNAVTTELLTL

SNNGSILSLWFVLLIGSYVVTLAMLRSYSGEGKKKASATCTSHMTVVTLH

FVPYIYIFDRPF/VYLHLRPALPALPMDTAVSIX

>OranORuc435.11

MELGNHTRVSEFIFRGLTQSRELSFVLFVLLFVLYVITVSANLLIIVTVT

WESRLHLPMYFLLRNLSILDISFSCITIPKVLVDLLSKRKAISFNGCLTQ

IFFFHLLGGADTFSLSVMAYDRYVAISKPLHYVTIMTKGTCFGLVAASWV

GGFVHSVVQIFLLLLLPFCGPNVLDGFYCDVPQLLKLSCSNAVTTELLMI

SNNGMITSLWFVLLIGSYTVTLLMLRSHSGEGKKKAIATCTSHMMVVTLH

FVPCIYIYARPFTALPTDTAVSITLAGIIPVLNPIIYTLRNREMMLAMKR

LKKRDIQSGRE*

>OranORuc453.1T

HTPMYFFLSKLSFVDLCLTTTTLPKMLLNIQTRKKTISYTGCLTQAYFLI

FFAFMDSFLLTAMAFDRYVAICHPLYYTTTVNPQLCVFLVAGSWFITFLH

ALLHTVMTARLSFCGDNEIPHFYCDLTPLLKLSCSDISTNEVLIFTVTVL

LGSAPAMCIVVSYIRIVSAVLKIPSAEGKQKVFSTCGSHLTVVTLFYGAG

MGVHFQPSSIQSKHEDLLASVLYTVVTPMLNPFIYSLRNKDMKGALRKL

>OranORuc453.2

MARENQSSLSEFLLLGLSDRAEQRQLLFVSFLCMYLLGVLGNLLIVLAIG

SDPHLHTPMYFLLSNLSAVDACFLSTTVPKMLCNIQTHSRSVSFSGCLAQ

MYFFMLFVILDNFLLTGMAYDRFVAICHPLHYSAIMNPRVCALLVGVPWI

VVSLISLLHTLLVFHLSFCSNNEIFHFFCEISHILKLSCSDTLPNEILLY

FFVVVLAAVPFIGILVSYSRIISTILKIPSVGGRWKAFSTCGSHLSVVFL

FYGTGLGVYFSPGSSQGSRRGSTASVMYTVVTPMLNPFIYSLRNKDMKRA

LMKLCHRKTLASQKL*

>OranORuc453.3

MHELMEGKNQTLTSEFFLLGLSNQLEEQQSLFVLFLSMYLTTGLGNLLII

LAIAFDPHLHSPMYFLLSNLSFVDLCFTTTTVPKMLVNHISGNKTIPYAG

CLTQMFFFIWFASIDSFLLVAMAYDRYIAICHPLRYASLMIPRLCALLVA

TSWSFACINALTHTVLLTQLSFCSHNEIPHFFCDLSPLLKLSCSDTFIND

VLVYTVGALPILMPFVGILVSYTRIFAAVLRIPSARGKRKAFSTCGSHLS

VVSLFYGAVIGVYLSPMSYHTVEKDTAAAVMYTVVTPMLNPFIYSLRNRD

MKGALWKLFSRIVASPGDCDPASGVRSLNFK*

>OranORuc453.4

MVSEFILIGFSTFPRLQGFFFVLFLLMYLSTLLGTLLIIVTVQNERKLHT

PMYCYLCTLSLSEIFYTFAIIPKMLSDLLSTQRTISFLGCANQMFFSFTF

GFTHSFLLTTMGYDRYVAICYPLRYSVLMKPQICIWLLVSSCLGGLVMGL

VVTVAVFHLSFCGPNEIHHFACHMLPIVKLACGDVSLTALAVGVLCIIVL

MSCFLLILLSYAFIVATIMKIPSAEGRLRTFSTCAAHLTVVVVHYGFASI

IFFKPKTHVLEGDSLMAVTYTVFTPFLSPIIFSLRNKELKNALKKTFKNG

LFPHTKK*

>OranORuc453.5T

VLILLSHAFILATIVKISSAEGLHRALSICESHLTVVVVHYGFASVIYLK

PKALPCPEGDTVMGVTYKRLTPFLSPIIFSLRNKELKNILKKTIKDCLFP

>OranORuc453.6T

LVSEFILVGFSTLPSRLQPVLFVLILLMYLSTLLGTLLIVITVRNERKLQ

TPMYHYLCTLSLSEIFYTFAIIPKMLSDLLSTQRTISFLGCANQMFFSFT

FGFTHSFLLTTMGYDRYIAICHPLR

>OranORuc581.1P

SRHLHTFMHFFLKHLSILNLCLISVTLPKSILNALSD/VSFLGCISQVLS

FVILGAANRAFLTVMFYDYYVATCRPQLYDIIMNRGACEKKVSTSWLTSG

LYTLMEMAATFSSHFCCPRVIQQFLCDVPQLLKLMFPGGARPEVCTLVAW

ITLAPGCFVPSFCLMSTSSWSC*GCDPARTGPKPSPPVCPTSLSWLHSLL

TALAYLQPISDSLDLLVSVFYSLVPPTLTPL

>OranORuc581.2P

RNRATATEFILLGLTDDPPLEGL/PPLKVCLVSFLLLAYLLSVIGNLTII

ALTLLVPHLHTPMCFFLRHFSLLEASFTSVCIPNFLVNISTGDKTTYYSG

CMTQIFYILLGASELFLLACMSFNHYATICSTLSHTTILNSRVCTLLVLC

RCLAEFLIIVPGVTFGLQLEF

>OranORuc581.3P

MRNQATVTEFILLGLTDDTSLKVCLVSFLLLVYLLSVTSNLTIIALTFLV

PHLHTIMCFFLRNFSLMEASFTSVSIPNFLVSIFTGDKTISYSGCKTQIF

FYILLG/LGASEFFLLACMSFNHYAAICRPLSYTTIMNSRVCTLLVFCRC

LAGFFII

>OranORuc659.1

MANLTFKPEFILLGFSDVRELQLVHAALFLLVYLVALMGNLLIIAIITLD

MRLHTPMYFFLKNLSILDLCLISVTVPKSIINSLTDRRSISFLGCGVQVF

SVILFAGSELFILTAMSYDRYVSICCPLRYEITMNREACKQMVAASWLTA

GLFAVTSSAEMFSLPFCGFPVILQFFCDIPSVAKLSCSAPHIALDVIVVT

GVSLGASSFICILISYICIFSAVLRMPSTEGRTKAFSTCLPHLVVVTLFL

FTGSVEHLKPPTASPSLLDLLVSMFYVVMPPTLNPLIYSLRNRDIKVAMR

RVIGGTLFSKDRKFHLML*

>OranORuc659.2P

MVNVTRMTGFLLMGFSDIWELQLVHATLFLLVYLVALTGNLLIIAITTFD

RRLHTPMYFFLKNLSFIDLCLISTTIPKSIGNSLTNHRSISFPGCVIQLF

SVVLFAGSELFVLTAMSYDRYVAICRPLHYEAVMSRRTCVQLVAASWISA

GLVGVMSAAEIFSLPFCGSYEIPQFFCDITSVVKGSCSKSHIGLDVIIIT

SACFAVFCLICILVSYVCIFSAVLRMLSAAGRFKTFSTCLPHLAVTTVFF

TTAAFANLNFSSDSSSILDLLVSVFYIAVPPALNPLIYSLRNKDMRMVMG

RVLSVHLYTK

>OranORuc659.3

MTNVSLVTNFLLMGFSDIRKLQIVHATLFLLVYLAALTGNLLIITVTTLD

RHLHTPMYFFLKNLSFIDICYISVTVPKSIAISLTDNSSISFLFCATQLL

LVVWLAASEFFVLTAMSYDRYAAICRPLHYEVTMCRGACVKMMASSWLSG

SLFGFLFSTSTFSLSFCGSNAVQQFFCDVPSLLKISCSEDHIAIDASVSA

GVTLGVICFIYIILSYIHIFSAVLRMPSTESRAKAFSTCLPHLIVIIVFF

FTGAFAYLKPLSDSPSVQDLLVSVLYAVVPPTLNPLIYSLKNKDVKSALG

RALKGTFPCLPLSNKLSVSLC*

>OranORuc659.4

MTNVSTVAEFLLLGFSDIRELQLVYATLFFFVYQVALLGNLLIVAVTTLN

QSLHTPMYFFLKNLSFIDLCYISTTVPKSIVNSLIGDSSISFLGCVSQLF

LLVLFAASEFFVLTAMSFDRCAAICSPLSYELIMNKTACMIMTAVSWLRG

CLFGVLFSASTFSLTFCGSNAVQQFFCDVPPLLKISCSEDHVAIDVSVAT

AIVLDAICFFYIVFSYVRIFSAVLRMPSSEGRAKAFSTCLPHLAVITLFV

STGVFAYLKPPSYSPSSLDLLVSVFYTVVPPTLNPIIYSLRNRDLKAAMG

RILKGTIPLHSLWNKMFVSLCE*

>OranORuc659.5

MTNGSTVAEFLLLGFSDIRELQLVHATLFLLVYLVALLGNLLIVAVTTLD

QHLHSPMYFFLKNLSIIDLCYISTTVPKSIVNSLTSDSSISFLGCVLQLF

LVILFAASEFFLLTAMSYDRYAAICSPLHYELIMNKTACIRMAAASWFGG

CLFGVLLSASTFSLTFCGSNTVQQFFCDAPPLLKISCSEIHIAIDVSVAA

GFVLDAICFIYIILSYVFIFSAVLRMPSSEGRTKAFSTCLPHLTVIIIFL

FTGAFAYLKPPSDSPSALDLLVSMFYTVVPPTLNPLIYSLRNRDLKAALG

RILKGTFPLHPLCDKMSVSMYQ*

>OranORuc659.6

MTNVSTVAEFLLLGFSDIWELQLVHATLFLLIYLLALLGNLLIVAVTTLD

QRLHTPMYFFLKNLSIIDLCYISTTVPKSVVNSLTSDSSISFLGCVSQLF

LVVLFAVSELFVLTAMSYDRYAAICCPLRYELIMNKTACMRMAVASWLNG

GLFGILLSASTFSLTFCGSNTVQQFFCDIPPLLKISCSKVHVAIDVSVAT

GLVLDAVCFIYIILSYVFIFSAVLRMPSSEGRTKAFSTCLPHLTVIIIFL

FTGAFAYLKPPSDSPSALDLLVSMFYTVVPPTVNPLIYSLRNRDLKAALG

RILRGHSPSIHFGTKCLYPCTNNALPPLTRGIVLRLIHIYH*

>OranORuc659.7

MTNVSMVAEFLLLGFSDIRELQLVHATLFLLIYLLALLGNLLIVAVTTLD

QHLHTPMYFFLKNLSFIDLCYISTTVPKSIFNSLTGDRSISFLGCVSQLF

LVVLFAASEFFVLTTMSYDRYAAICSPLRYELIMNKTASVRMAAASWLIG

CVFGVLFSASTFSLTFCGSNAVQKFFCDAPPLLKISCSEDHIGIDVSVAT

AIVLDAICFFYIIFSYVHILSAVLRMPSSEGRTKAFSTCMPHLTVIIIFL

FTGAFAYLKPPSDSPSALDLLVSMFYTVMPPTVNPLIYSLRNRDLKAALG

RILKGTFPQHSLWDKMSVSMYQ*

>OranORuc659.8

MTNFSMVTEFLLWGFSDVWELQLVHTVLFLLVYLATLTGNLLILTVTTLD

RHLHTPMYFFLKNLSILDICLISTIAPKSIHNSLTSNRSISFLGCVAQLL

LWVLFAGSEIFVLTAMSYDRYAAICYPLRYEVLMSRGVCVKMMAASWLIG

SVFGFLLSASTFSLPFYGSNAVPQLFCDVPSLLQISCSVDHSAIDVSVTA

GVALAVGCFIYIILSYVRIFSTVLRMPFTAGRAKAFSTCLPHLMVVTLFI

MNGAFAYLKPPSDTPSVLDLLVSMLYTMVPPALNPLIYSLRNRDMKNALR

RTLKGRLLRPSFQVKISLSLIRGNHQWHVHL*

>OranORuc659.9P

VASITRIIKFLLLEFSDIQELQLVHTALLLLIYLVALSGNLLIVAITSLH

QRFHTPMYIFLKNLKNMSVLDLCYISVTIPKSILISLTQYSSLSFLSCAA

QDWLVIFFLDAE*FVLMVMS*NCYVTICCPLCYEVTAKKAYMQMGAASWL

RRILFVILISALTFSLSFYGSNTVQQYFCGALSILNIPCSEDHVAIDVSV

IFVVALVVFCFISIITSYMSIFSAVRRILAQQRR/TEGWTKVVSTFLPHL

MVVTLFVMIFFCAYMKLSSNPPSVLDLLVLYTAVHSAPNPLIYNLRKRGM

KTSLGKIL

>OranORuc659.10P

GLLFGVIHTTRTFTLTFYVFHEIQQFFYDGPSLLKISCTETYVVPDAGMA

FGTGFTLISFLSIIISYVHIFFAAPKMSFSEGWTEAVSTCLPHLTLITGF

LSTATIAYLKPPETPP/PRDSASMLDQLVSMLYNVVHFYTLIPIIYSLRN

R

>OranORuc664.1T

MANCTCVTRFLLLGFSEVRELQLVHAALFLLVYLVALTGNLLIRAVTALD

RRLHTPMYFFLRNLSFIDVCYISVTVPKSIFNSPAHRCDIRFPDCVLQVF

FFISLASAEMGVLTVMSYDRYAAICRPLLYDAVMSRGACAKMAATSWLSG

GFAGLMHTAATFSRTFCGSNMIQQFFCDIPQ

>OranORuc704.1P

FLLAYVLALFGNISIILVSRLDPQLNTPMYFFLSNLSFLDLCYTTSTTPQ

MLVNLWGPEKTISYGGCVTQLYIFLALGSTECILLAIMAFDRYAAICRPL

RYPVIMNPQRCMHMAAGTWFSGFANSLVQSTLTVVVPRCGHQVLDHFFCE

VPALLKLACTDIRLNEAELNVLGSLLLLVPLTLIVGTYSFIARTVQRIAR

QRADTSLQHLCPPHL

>OranORuc724.1P

VAICHPQQYPVLMCPKVCGLMVATSLLGSLRNAPIHCVYTLNISFCTSWE

IHPFFCEISTLLELFCHDTAI*ENGLFLTGIL/PISVIMASYRERLQIIL

EKGSSRES*KVLTTCFSHIVVVCLIYGATTFKYLLPKSYHTSSQDQVVSV

FYTLLTPMSNPLNYSL*N*EVLKTLGKVL

>ModoOR1.1.1

MEKKNLSNLEEFILLGFPGTQGLQIFLFLLFFVMYVLTVMGNMAIITLVW

IHQRLQTPMYFFLCNLSFLEIWFTTACVPKTLANFASQSKVISFISCATQ

MYFVFSLGCTEYFLLAVMAYDRYLAICYPLRYSTIMTHSVSARLALVSWV

CGFSAITVPTALITRLSFCASNVINHFFCDIAPWIVLSCTDTRVVELVSF

GIAFCVILGSCIITLVSYIYIISTIVRIPSAQGRHKAFSTCSSHLTVVLI

WYGSTIFLHVRTSVESSLDLTKAITVLNTIVTPVLNPFIYTLRNKDVKEA

LRKTVSGK*

>ModoOR1.1.2

MKYTNGTSSVKEFILLGFPSLNHLQALLFLLFCIIYVMTLIENLVIIVIV

QVNRQLHTPMYFFLANLSVLEALYTSVTIPKMLANFLTEKKTISFAGCFT

QLFFFLSLASSECFLLAAMAYDRYLAICLPLRYPTLMNPTVCLVLALSAW

LSGFLASFVSIVLISQLSFCGPNVLNHFFCDISPLLKLSCSNTEAIETLD

FVAALAVLMTSLCVTAFSYICILATVIQIPSGTGRRKAFSTCASHLVVVT

VFYTTTIFMYARPRAISTFDFNKLVSIIYSVVTPLLNPIIYCLRNREVWE

ALAKLTRMLRFFVAAQFSSSVREKFI*

>ModoOR1.1.3

MFSWDNYTQVTEFILLGFPGSLGLHLSLFMLFLFVYSLTVIENIIIITLI

RVNPSLHKPMYLFLSNLSFLEVWYISVTVPKMLLTFMAPEFGRISFMGCM

AQLYFFLGLACTECALLGVMAYDRYVAICNPLRYPAIMGPGVCLRLAASS

WLSGFTISLGKVFFISRLGYCGPNVMNHFFCDVSPLLNLACTDMSMAELI

DFLLALLILIVPLLVTIFSYVCIISTVLRIPSASGQQKAFSTCASHLAVV

VIFYSASLFIYARPRAIYSFDYNKLVSVVYTVLTPLLNPIIYCLRNTEVK

VALKKTVQRMTKKMDAVA*

>ModoOR1.1.4

MALSNQTLVAEFVLQGFSEIPQLQPFLFTAFLLLYLMALTGNLVIVMAIC

LDSNLHTPMYFFLANLAIIDIGCTSTVLPKLLENLVVEKKSISFNGCMTQ

LFFFSWFLGTELLLFTAMAYDRYVAICHPLHYSAMMNKMVCTFLVGSVWT

TGTLSSSLQTGLMVRLTFCGPNQINHFLCEVPTLLLLSCTSTYLNDIMIV

IADIYFGVVSFVLTMVSYGFIISNILKIRTTEGKKKAFSTCSSHLIVVTI

YYTTITYTYMLPGSSSSMDNGKVVALLYTTVSPTLNPLIYTLRNKDFKTA

LKKLFPSVL*

>ModoOR1.1.5

MASNNQTLVTEFILQGFSETPQLQILLFISFLFLYLTALTGNILIVMTTS

LDSNLHTPMYFFLANLAILDIGCTSTVLPKLLENLVVEKKSISYEGCMTQ

LYFLSWFLGTELLLFTVMAYDRYVAICHPLHYNTMLSKMICVLLVSSVWV

TSALVSLLHTGLMVRLTFCGPNEIQHFLCEIPSLLLLSCSSTYLNNIAII

MADVYFGVINFVLTMVSYGFIISNILKIRTTEGKKKAFSTCSSHLMVVSI

YYTTIIYTYFFPGSGSSMDNGKVVALLYTAFCPTVNPLIYTLRNKDFKTA

LKKMFLFIQ*

>ModoOR1.1.6P

MAFNNQPFVTEFTPQGFSENSHLEILFLCFFCFLYSGSYLKFPYCFNWGH

SRHSHTHVFFFINIALLDIICMIAVVPKLLKNLMKEKKVISF/MTQVYVF

SWTLGSELLIFITIAYDHYVAICRPLHYITLMSKKTCGVEWIVVGLCSGA

HTMMLIQLSFCGPNVIDHLF*EIP

>ModoOR1.1.7

MAVSNQSIVTEFFLQGFSETPPLQLALFFIFFFLYIMALIGNALIVLAIS

IDSGLHTPMYFFLANLAILDIGCTSTVLPKLLEILVDKKSISYDGCMTQL

YFFTWFLGAELLLFTAMAYDRYVAICHPLHYTTMMSRMVCIFLAGSVWAI

SVINSSIHTGLMVQLTFCGPNKIKHFLCEIPTLLLLSCTSTYLNNMMIVI

ADVYFGVINFGFTMVSYGFIISSILKIRTKEGKKKAFSTCSSHLIVVTIY

YTTITYTYFLPGSGSSMDNGKIVAVLYTTISPTLNPLIYTLRNKDFKTAV

KKMFPFIQ*

>ModoOR1.1.8P

MALHNQTLVTEFILQGFSETPQVQILLFISLLFLYLIALTGNILIVMAIG

LNSGLHTPMYFFLANLAILDIGCTCTVLPKLLENLYVEKKSISYEGCMTQ

VFFLTFFFG/FSLRFFLDIELLLFTAMAYDRYVAICHPLRYSTMMSRMVC

MLLVSSVWATGVFSSLINTGLMVRLTFCGPNEIQHFLCEIPSLLVLSCSS

TYLNNIMVVIADVYFGVINFVLTMVSYGFIISNILKIRTTEGKKKAFSTC

SSHLMVVSMYYTTIIYAYIFPGSDSSVDNSKVVTLLYTAVDPALNPFIYT

LRNKDFKTALKNCFQSLN

>ModoOR1.1.9

MALHNQTLVTEFILQGFSETPQVQILLFISLLFLYLIALTGNILIVMAIG

LNSGLRTPMYFFLANLAILDIGCTCTVLPKLLENLVVEKKSISYEGCMTQ

VFFLTCFLGSELLLFTAMAYDRYVAICHPLRYSTMMSRMVCMLLVSSVWV

TGVFSSLINTGLMVRLTFCGPNEIQHFLCEIPSLLVLSCSSTYLNNIMVV

IADVYFGVINFVLTMVSYGFIISNILKIRTTEGKKKAFSTCSSHLMVVSM

YYTTIIYAYIFPGSDSSVDNSKVVTLLYTAVDPALNPFIYTLRNKDFKTA

LKKLFSFIH*

>ModoOR1.1.10

MASNNQTLVTEFILQVFSETPQLQLFLFISFLFLYLTAFTGNILIVMAIS

LDSGLHTPMYFFLANLAILDIGCTCTVLPKLLENLVVEKKSISYKGCMTQ

LYFLTCFLGTELLLFTAMAYDRYVAICHPLHYSTMMNRMVCMLLVSSVWA

TGSLSSLLHTGLMVRLTFCGPNEIQHFLCEIPSLLLLSCSPTYLNNIMIV

IADVYFGVINFVLTMVSYGFIISNILKIRTTEGKKKAFSTCSSHLMVVSM

YYTTIIYTYIFPGSGSSMDNGKVVALLYTTVGPTLNPLIYTLRNKDFKTA

LKKMFLFIY*

>ModoOR1.1.11

MAWMNQTLVTEFFVQGFSEAPHLRFLFFFLFLSLYTVALSGNILIFVTIS

FNSTLHTPMYFFLVNLAVVDVLCTSTILPKLLENMMGRKTISFAGCMAQL

YFFTWSLGAELLLFTAMAYDRYVAICHPLHYSTMMSKKVCLLLSTGVWSI

SILNTSINSSLMFRLSFCHSNVVNHFFCEIPPLLKLSCTPTYLNETMAFT

ADVFLAVGNFIITMLSYACIISSILKIRTTEGKKRAFSTCSSHLIVVSMY

YSTVIYTYIRPSSSYSLDKDKIVSVIYTSVAPTLNPLIYTLRNKEVKAGL

KKVFSFFKR*

>ModoOR1.1.12

MAVSNQSIVTEFFLQGFSETPPLQLALFFIFFFLYIMALIGNALIVLAIS

IDSGLHTPMYFFLANLAILDIGCTSTVLPKLLENLVDKKFISYDGCMTQL

YFLTWFLGAELLLFTAMAYDRYVAICHPLHYTTMMSRMVCIFLAGSVWTI

SAISSAVHTGLMVQLTFCGPNKIKHFLCEIPTLLLLSCTSTYLNNIMIVI

ADVYFGVINFGFTMVSYGFIISSILKIRTKEGKKKAFSTCSSHLIVVTIY

YTTIIYTYILPGSGSSMDNGKIVAVLYTTISPTLNPLIYTLRNKDFKTAV

KKMFPFIQ*

>ModoOR1.1.13P

NNQPFVTEFTPQGFSENSRLEILFLCLFSFLYSGSCLKFPYCFSWGHSRH

SHTHVFFFLINIALLDIICMIAVVPKLLKNLMKEKQVISFPG/MTQVYVF

SWTLGSELLLFIAIAYDHYVAICRPLHYITLMSKNTCGVEWIVGALYSGA

HTMMLIQLSFCGPNVIDHLF*EIP*FCHFLAP**K*MSL*HL*QNSSLVV

PIFFWPLYSLTLSLPIS**SRLKRRRRGHFSLLLPLQGGNHILFFWGGEV

YLYKSK*SFCPKKWKAGAILYTTLTPSVNPSIYTMKNKDVKAGLKK

>ModoOR1.1.14

MNNQTFVTEFILKGLTEIPQFQVLFFILFLFLYTIALIGNSLIVAAISLS

LGLHTPMYFFLVNLALLDVICTCTSVPKLLQILGGEDKTISFGGCMTQLY

FLTWSVAAELLLFTAMAYDRYVAICQPLHYSKMMSKQVCVSLAVAVWGIS

AFGATTNTSLLLRLSFCGPNVIDHFLCEIPSLLPLSCSSTYVNDIMTIVA

DIFFAVLNFLLTMLSYGFIIFSIMAIQTTEGKKRAFSTCSSHLIVVTMYY

STVIYVYLSPGSSYSPDKGKIMAVLYSTVSPTLNPLIYTLRNKDVKRALK

KLFIFLKEK*

>ModoOR1.1.15P

CTTCFLIKILVNLVSEKKTIFYGVFMAQMYFLMWLIFGELLLFTLMAYNR

SVAICYPFHYSMMMSKICVFLTDVELSISELSTTINTGLII*LSSYKPT*

SILL*NLPPC*FTLSPQRM*IS*QSLLMSYFQF*SSCSPLSYYIISRFMR

IQTKEGKKRAFSTSFSLLVVDTM*YCTILCLC*SHLYLFS*K*EICGCVI

HNSPILIHLINTFRNKDITETLR

>ModoOR1.1.16

MLLRNQTLVTEFILQGFSDSIPIRILLFGTFFFLYTIALTGNILIIVLVI

RSSSLHTPMYFFLVNLAMLDMMCTTSVLPKVLENLVTEKKTISYGGCMAQ

VYFLIWSVSGELLLFTAMAYDRYVAICHPLHYSTMMSKKICVLLAVGVWS

ICGLNTTINTGLMIRLSFCGPNIINQFFCEIPPVLLLSCTSTYVNNIMTV

LADVFFSVLNFLLTIVSYCFIISSIMRIQTTEGKKRAFSTCSSHLIVVTM

YYCTVFYAYVSPISSYSPENGKVVAVLYTAVSPTLNPLIYTLRNKDVREA

LRKGFRFVSEGL*

>ModoOR1.1.17

MLLRNQTLVTEFILQGFSDSIPIRILLFGTFFFLYTIALTGNILIIVLVI

RSSSLHTPMYFFLVNLAMLDMICTTSVLPKVLENLVTEKKTISYGGCMAQ

VYFLTWSVSGELLLFTAMAYDRYVAICHPLHYSTMMSKKICVLLAVGVWS

ICGLNTTINTGLMIRLLFCGPNIINQFFCEIPPVLLLSCTSTYVNNIMTV

LADVFFSVLNFLLTIVSYCFIISSIMRIQTTEGKKRAFSTCSSHLIVVTM

YYCTVFYAYVSPISSYSPENGKVVAVLYTAVSPTLNPLIYTLRNKDVREA

LRKGFRFVSEGL*

>ModoOR1.1.18P

TLVPGAILQSFSDSIPIRSLHLFSLNATAFTGNTIIIVLVVWSANF*LLC

TFFLVY*AKLDIICTMSVLPKFLKNLVSEKTISCGECKTQFYFFLFWVTV

EILPFTAMAYDR

>ModoOR1.1.19

MAISNHSQVTEFILQGFSEHPSLRIPLFGCFLSLYLVALTGNVLIITAIT

LNSSLHSPMYFFLFNLATMDIICTSSVLPKVLQGLLSEENTISYGGCMTQ

LYFLIWSASSELLLFTVMAYDRYVAICHPLHYSTRMSKMLCNVLAASVWL

ICAFNSSIHTGLMTQLSFCGPNVITHYFCEIPPLLLLSCTSTYINRVMTV

LADAFYGFINFVLTLVSYGFIISSILKIQTTEGKKRAFSTCSSHLIVVSM

YYTAVFYAYISPICGYNPERSKLAGVLYTMLSPTLNPLIYTLRNKEVKMA

LRKLFPCFRN*

>ModoOR1.1.20

MASNNQTSVTEFILKSFSENPQFQIFLFSLFLGLFIVAFTGNSLIIVVIS

LNPGLHTPMYFFLINLALMDILSTFTVVLKLLQNLMTENTISYGGCIAQI

YFLTLCIGAELLLFTAMAYDRYAAICQPLHYNKMMNKTVCCLLASVVWSV

SGINTTIHTIITVRLSFCGPNAIDHFFCEIPPLLPLSCSSTYINNIMTFV

SDMFFAIINFLFILLSYGFIISSILKIQTTEGKKKAFSTCSSHLIVVTMY

YCTIIYIYILPALGQSLKEGKIASVFYAIVSPAVNPLIYSLRNKDVKVAL

NKLCPFFQK*

>ModoOR1.1.21

MAISNHSQVTEFILQGFSEHPSLRIPLFGCFLSLYLVALTGNVLIITAIT

LNSSLQSPMYFFLFNLAIMDIICTSSVLPKVLQGLLSEENTISYGGCMTQ

LYFLIWSGSSELLLFTVMAYDRYVAICHPLHYSTRMTKMLCNVLAASVWL

ICAFNSSVNTGLMTQLSFCGPNVITHFFCEIPPLLLLSCTSTYINRVMTV

LADAFYGFINFVLTLVSYGFIISSILKIQTAEGKKRAFSTCSSHLIVVSM

YYTAVFYAYISPICGYNPERSKLAGVLYTMLSPTLNPLIYTLRNKEVKMA

LRKLFPCFRN*

>ModoOR1.1.22

MASNNQTSVTEFILKSFSENPQFQIFLFSLFLGLFIVAFTGNSLIIVVIS

LNPGLHTPMYFFLINLALLDILSTCTVVLKLLQNLMTENTISYGGCIAQI

YFLTWFLGSELLLFTAMAYDRYAAICQPLHYNTMMNKTVCCLLASVVWSV

SGINTTIHTIITVRLSFCGPNAIDHFFCEIPPLLPLSCSSTYINNIMVFV

ADMFFAIINFLFILISYGFIISSILKIQTTEGKKKAFSTCSSHLIVVTMY

YCTIIYIYILPALGQSLNEGKIASVFYAIVSPAANPLIYSLRNKDVKVAL

NKLCPFFRK*

>ModoOR1.1.23

MASNNQTSVTEFILKSFSENPQFQIFLFSLFLGLFIVAFTGNSLIIVVIS

LNPGLHTPMYFFLRNLALMDILSTCTVVPKLLQNLMTENTISYGGCIAQI

YFLSWCLGAELLLFTAMAYDRYAAICQPLHYNTMMNKTVCCLLASAVWAF

SGTNTTINTLMTVRLSFCGPNAIDHFFCEIPPLLPLSCSPTYINNIMTFV

ADMFFAIINLLFILLSYGFIISSILKIQTTEGKKKAFSTCSSHLIVVTMY

YCTIIYIYILPAFGQSLKEGKIASVFYAIVSPAVNPLIYSLRNKDVKVAF

KKLCPFFRK*

>ModoOR1.1.24P

GFSEHPSLRIPLFGCFLSLYLVVLTGNVLIITAIALNSGLQAPYTLF/SP

IYFVLFNLATMEIICTSSLLPKVLEGLLSEENTLSYGDCMTQLYFLIWSA

SSELFLFTVMAYDHYVAICHPLSTRMIKMLCNVFTVSVWLICAFNSSINT

GLMT*LSFCGPNVITQFFCEIPPLLLLSCISTYINRVMTVLADAFYGFIN

FVLTLVSYGFIISSILKNQTTEGKKRAFSTCSSRLIVVSMYYTAVFYAYI

SPICGYNPERSKLAGALYTMLSPTLNPLIYTLRNKEVKMALRKLFPCFRN

>ModoOR1.1.25

MASNNQTSVTEFILKSFSENPQFQIFLFSLFLGLFIVAFTGNSLIIVVIS

LNPGLHTPMYFFLINLALLDILSTCTVVPKLLQNLMTENTISYGGCIAQI

YFLTWFLGSELLLFTAMAYDRYAAICQPLHYNTMMNKTVCCLLASAVWAF

SGTNSMIQTIVTVRLSFCGPNAIDHFFCEIPPLLPLSCSPTYINNIMAFV

ADMFFAIINLLFILISYGFIISSILKIQTTEGKKKAFSTCSSHLIVVTMY

YCTIIYIYILPAFSQSLKEGKIASVFYAIVSPAVNPLIYSLRNKDVKIAL

NKLCPFFKK*

>ModoOR1.1.26

MAISNHSQVTEFILQGFSEHPNLRIPLFGCFLSLYLVALTGNVLIITAIT

LNSSLHSPMYFFLFNLAIMDIICTSSILPKVLQGLLSEENTISYRGCMTQ

LYFLIWSGSSELLLFTVMAYDRYVAICHPLHYSTRMTKMLCNVLAASVWL

ICAFNSSINTGLMTQLSFCGPNVITHFFCEIPPLLLLSCTSTYINMVMTV

SSDAFYGFINFVLTLVSYGFIISSILKIQTSEGKKRAFSTCSSHLIVVSM

YYTAVFYAYISPICGYNPERSKLAGVLYTMLSPTLNPLIYTLRNKEVKMA

LRKLFPCFRN*

>ModoOR1.1.27P

MAISNHSQVTEFIFQGFSEDPSLRIPLFDCFLSLYLVALTGNVLIITAIT

LNSSLQSPMYFFLYNLAKMDII*TSSVLPKVLEGLLSEENTISYRGCMTQ

LYLVWIIRITSFHCHGI*LLCGHLPPLALQHQDDQDALKCVDSYCVADMC

LQFLHKYWSDDTTVLLWTQYDYSFLL*DPSSVVALLYFLPPTLNMVMTVS

SDAFYGFINFVLTLVSYGFIISSILKIQTSEGKKRAFSTCSSHLIVVSMY

YTAVFYAYISPICGYNPERSKLAGVLYTMLSPTLNPLIYTLRNKEVKMAL

RKLFPCFR

>ModoOR1.1.28

MASNNQTSVTEFILKSFSENPQFQMFLFSLFLGLFIVAFTGNSLIIVVIS

LNPGLHTPMYFFLINLALLDILSTCTVVPKLLQNLMTENTISYGGCIAQI

YFLTWCLGAELLLFTAMAYDRYAAICQPLHYNTMMNKTVCCLLASAVWAF

SGTNITINIIMTVRLSFCGPNAIDHFFCEIPPLLPLSCSSTYINNIMTFV

ADMFFAIINLLFILLSYGFIISSILKIQTTEGKKKAFSTCSSHLIVVTMY

YCTIIYIYILPALGQSLKEGKIASMFYAIVSPAVNPLIYSLRNKDVKIAL

KKLCPFFRK*

>ModoOR1.1.29

MQISTALMATMNQTLVTEFFIKGFSEAPHLQSLFFVLFLSLYTVALSGNV

LIFVTISFNPALHTPMYFFLINLAMVDVLCTSTILPKLLENMVSVKTISY

EGCMAQLFFFTWSLGAELLLFTAMAYDRYVAICHPLHYSTMMSKGVCMLL

ATGVWIISIINTSVHTCLMMKLTFCHSNVVNHFFCEIPPLLKLSCTPTYL

NETMAFTADVFLAVGNFMITMLSYACIISSILKIRTTEGKKRAFSTCSSH

LIVVSMYYSTVIYTYIRPSSSYSLDKDKVVSVIYTSVAPTLNPLIYTLRN

KEVKVGLKKVFLFFRR*

>ModoOR1.1.30

MAKRNQTQVTEFILQGFSEHPELQIPLFSCFLSLYVVALTGNVLIIIAIV

LNTNLHSPMYFFLFNLAIMDIICTSSILPKVLDGLLSEKNIISYVSCLAQ

LYFLTWALSSELLLFTAMAYDRYVAICHPLHYSTMMSKTLCSMLAAGVWG

ICAFNSSIHTGLMTRLSFCGPNVITHFFCEIPPLLLLSCTSTYINSVMTV

LADAFYGVFNFLLTLVSYGFIISSILKIRSVEGKKKAFSTCSSHLIVASM

YYTAVFYAYISPVASYNPEKSKLAGVLYSVLSPTLNPLIYTLRNKEVKQA

LRKLFPFYRN*

>ModoOR1.1.31

MINETQTFQFILQGFSEHPELRILLFICFFFLYMVALAGNVLIITAIVFN

SSLHSPMYFFLFNLATMDIICTSSILPKVLEGLVSEENAISFGGCMAQLY

FLTWSASSELLLLTVMGYDRYVAICHPLHYNTMMSKTLCSVLASGVWGLC

AFNTAIHTGLMIRLDFCGPNVVTHFFCEVPPLLLLSCSPTYVNTIMIVLA

DAFYGILNFAMTLVSYSLIISSILKIRTTEGKKKAFSTCSSHLIVVSMYY

TAVFYAYISPVSSYSPEKSKLAGVLYTMLSPTLNPLIYTLRNKEVKQALR

KILPFTKK*

>ModoOR1.1.32

MEKMGNETSVKEFILEGFPAVQHLGKLLFGVLLLLYLVSIVGNTVIVMIM

WMDHRLQIPMYLFLSGFSFLECCFTTSVIPKLLAIFLSGMQTISFAACLT

QTFVFLTLGVTGFFLMAVMSIDRYMAICNPLHYHSIMTMRVSFLLILFCC

SIGPIVTTSLIIKVSQLSFCGSNIIKHFVCDLGPLTQLSCSDTSSIESLA

FFLALFIILSSLAVTIICYINIALTITHLPSAKERQKAFSTCSSHLIVLF

LMYGSCIFIYMKPNQANRLDTNKEAALINTVVTPVLNPFIYTLRNKQFRQ

ALRDVIFKIKLLR*

>ModoOR1.1.33

MLTIDNLTTVQEFILEGFPAVQHLGKPLFMVHLLLYLVSITGSIVIVTIT

WTDHCLQTPMYFFLSNYSFLESCFITTVIPKLLAIFLSGRQTISFAACLT

QTFFFLFLGSTCFFLLAVMSLDRYVAICNPLHYHSIMNIKICFFLVLSCY

VLGFMLIISLVLMVSQLSFCGFNVINHFFCDLGPLVHLSCSDTSSVESLS

FLLSIVIILSSLIVTVISYFNIINTIVNLPLAKERWRAFSTCSSHLIVLF

LTYGSVVFIYVKPKQTDRVDVNKEAALVNTVITPLLNPFIYTLRNKQVKK

ALRDTLYRMRYMKSSWNFR*

>ModoOR1.1.34P

MLKIDNLTTVQEFILEGFPAVQHLGKPLFMVHLILY/IFIVTITWTDHCL

QTLMYFFLSNYSFLESCFITVIPKLLAIFLSGRQKISFAVYLTQTFFFLF

FGDNNMLLSFGCDVSG*VCGNL*PSALSQHHEHQDLFLLSLILLFLGFM*

IINLVLMVS

>ModoOR1.1.35P

QLSFCDFNIIDQFFCDLGPPRVSFMF*HH*LLNRYPFILSFLIVTVISYF

SIINTIVNLPSAKERWRGFSTCSSHVIVLFLTYGSIVFIYVKPKQTDRVE

VNKEAALVNTVITPLLNPFIYTLRNKQVEKA*TDALYEM

>ModoOR1.2.1

MDGSNESVVYEFVLLGLASSQEIQVLLFLFFSVFYVASMLGNLLIVLTVV

SDARLHSPMYFLLANLSFIDTCVCSIATPKMISDLFRKRKVISWNGCITQ

MFFIHTVGGTEMVLLIVMAFDRFIAICKPLHYLTIMRLRRCILLLMVSWV

IGFIHSMLQLAFVVNLPFCGPNKVDSFYCDFPRFIKLACMDTYRLEFLVT

ANSGFISIGTFFILIISYIFILVTVRKHSSGGSSKALSTLSAHITVVIFF

FGPCIIVYVWPFPTLPIDKFLAIFDALITPFMNPVIYTFRNKEMKVAMKK

LFSNIISFKKKI*

>ModoOR1.2.2

MDGSNESVVYEFVLLGLASSQEIQVLLFLFFSVFYVASMLGNLLIVLTVI

SDARLHSPMYFLLANLSFIDTCVCSIATPKMISDLFRKRKVISWNGCITQ

MFFIHTVGGTEMVLLIVMAFDRFIAICKPLHYLTIMRLRRCILLLMVSWV

IGFIHSMLQLAFVVNLPFCGPNKVDSFYCDFPRFIKLACMDTYRLEFLVT

ANSGFISIGTFFILIISYIFILVTVRKHSSGGSSKALSTLSAHITVVIFF

FGPCIIVYVWPFPTLPIDKFLAIFDVLITPFMNPVIYTFRNREMKVAMKK

LFSNIISFKKKF*

>ModoOR1.2.3

MDEMNHSVVTEFVLLGLSDSWEIQLLLFLFSSVVYVASILGNMLILITVI

FDPYLHSPMYFLLANLSFIDLGFCSIAAPKMICDLFRKHKVISFGGCVAQ

IFFSHAFGGVEMVLLIAMSFDRYVAICKPLHYITIMNSRMCILLLILAWI

IGITHSGVQLAFVVDLPFCGPNILDSFFCDLPQFIKLACTDTYRLQFMVT

ANSGFISLACFCVLIISYIFILVTVQKHASGVSSKAFSTLAAHITVVVLF

FGPLIFFYTWPFHISNLDKFLAIFDAVIIPFLNPVIYTFRNKEMKMGMKR

MFRRLVNFRKFHKGTGSKDCVFSWFLIVCRKQKLSLNFQR*

>ModoOR1.2.4

MDEVNHSVVAEFVFLGLSDSWEIQLLLFLFSSVVYVASILGNLLILITVI

FDSHLHSPMYFLLANLSFIDMGGSSIAAPKMICDLFRNHKVISFGGCVAQ

IFFSHALGGVEMVLLIAMAFDRYVAICKPLHYLTIMNPKMCILLLILAWI

TGITHSGVQLAFVVHLPFCGPNILDSFFCDLPQFIQLACMDTYRLQFMVN

ANSGFISLICFCVLIISYIFILVTVQKQSSGVSSKALSTLAAHITVVFLF

FCPLIFFYTWPHPVSHLDKFLAIFDAVVTPFLNPVIYTFRNKEMKLGMRR

MYSRLALLKKFS*

>ModoOR1.2.5

MDEVNHSVVAEFVFLGLSDSWEIQLLLFLFSSVVYVASILGNLLILITVI

FDSHLHSPMYFLLANLSFIDMGGSSIAAPKMICDLFRNHKVISFGGCVAQ

IFFTHALGGVEMVLLIAMAFDRYVAICKPLHYLTIMNSRMCILLLILAWI

TGITHSGVQLAFVVHLPFCGPNVLDNFFCDLSQIIQLACMDTYRLQFMVN

ANSGFISLICFCVLIISYIFILVTVQKQSSGVSSKALSTLAAHITVVFLF

FCPLIFFYTWPHPISHLDKFLAIFDAVITPFLNPVIYTFRNKEMKLGMRR

MYSRLVLLKKFS*

>ModoOR1.2.6

MDGMNHSIVSQFVFLGLSDSWEIQCFLFVSSSVLYVVSILGNMLILLTVS

VDSHLHSPMYILLANLSLIDLGFGSVTAPKMICDLFRKQKLISFGGCIAQ

IFLIHAFGGTEMVLLIAMAFDRYVAICKPLHYLTIMNPRMCIFLLIVSWI

IGLIHSLIQLAFVVNLPFCGPNELDSFFCDLPRFIKLACAETYRLQFMVT

ANSGFISLGSFCVLIVSYIFILVTVLKHSSAGSSKALSTLAAHITVVILF

FGPLIFFYTWPFSTSNLDKFLAIFDAVLTPFLNPVIYTFRNKEMKLAMGR

VYSQLWKKVF*

>ModoOR1.2.7

MSRVNHTLVSEFVFLGLSNSWYIQLFLFVLSSIFYVASMLGNSLIVLTVT

SDPHLHSPMYFLLANLSFIDLGVSSVTSPKMIYDLFRKRKVISFAGCITQ

MFFIHLIGGVEMVLLIAMAFDRYVAICKPLHYLTIMSPRMCTVFLVTAWV

IGFIHSVVQLVFVVNLPFCGPNELDSFYCDLPRFIKLACTDTYKLEFMVT

ANSGFISLGSFFILVISYIFILVTVQKHSSSGSSKALSTLSAHITVVVLF

FGPLIFVYTWPHPTSHLDKFLAIFDAVLTPFMNPVIYTLRNKEMKMAMRR

LCSQIGNFRKIS*

>ModoOR1.2.8

MDRKNHTVVSEFVFLGLTNSWDIQILLFLFSSLFYVSSILGNLLIVFTVI

SDPCLHSPMYFLLANLSFIDLGVSSVASPKMICDLFRKRKVISFFGCITQ

IFFIHLIGGVEMVLLIAMAFDRYIAICKPLHYLTIMSPRMCTLLLVAAWS

IGLIHSLVQLAFVVNLPLCGPNELDSFYCDLPRFIKLACTDTYRLEFMVT

ANSGFISLGAFFMLVISYIFILVTVQKHSSGGSSKALSTLSAHITVVVLF

FGPLIFFYTWPFPTSHLDKFLAFFDAILTPFLNPLIYTLRNKEMKLAMKK

VCRQLANFKEIS*

>ModoOR1.2.9

MDRGNRTVVSEFVFLGLTNSWDIQLLLFVFSSVFYLASMLGNSLIVLTVT

SDSHLHSPMYFLLANLSFIDLGISSVTSPKMICDLFRKHKVISFSGCITQ

MFFIHLIGGVEMVLLIAMAFDRYVAICKPLHYVIIMGPRMCTLLLVAAWI

IGLIHSVVQLAFVINLPFCGPNELDSFYCDLPRFIKLACTDTYRLQFMVT

ANSGFMSLGVFLILIISYIFILITVQKHSSSGSSKALSTLSAHIIVVILF

FGPIIFFYASPFNTSHLDKFLAIFDAILTPFLNPVIYTLRNKEMKLAMRR

VCSQLVSFRKIS*

>ModoOR1.2.10

MERVNHTVVSEFVFLGLTNSWDIQLLLFVFSSVFYLASMLGNSIIMLTVT

SDPHLHSPMYFLLANLSFIDLGISSVKSPKMICDLFRKHKVISFSGCITQ

MFFIHLIGGVEMVLLIAMAFDRYVAICKPLHYVIIMGPRTCILFLAASWV

IGFIHSVVQMAFVINLPFCGPNELDSFYCDLPKFIKLACTDTYLLQFMII

ANSGFMSLGVFLILIISYIFLLATVQKYSSTGSSKAFSTLSAHIIVVILF

FFPIFFFYAWPFNTSYLDKFLAIFDVVLTPFLNPLIYTLRNKEMKLAMRK

VYSQLVRFRKIS*

>ModoOR1.2.11

MDRKNHSVVSEFVFLGLSNSWDVQLFLFVFSFVFYLASMLGNSLIVLTVT

IDTHLHSPMYFLLANLSFIDLGMSSVISPKMMSDLFRKHKVISFYGCITQ

MFFIHLIGGVEMVLLIAMAFDRYVAICKPLHYLIIMCPRMCILLLAAAWI

IGLIHSVAQLAFVVHLPFCGPNELDSFYCDFPRFIKLACIDTYRLQFIVT

ANSGFMSLCVFFILIFSYIFILVTVQKHSSGGVSKALSTLSAHITVVVLF

FGPLIFVYTRPHATSRLDNFLAVFDIVLTPSLNPVIYTLRNKEMKVAMRR

VCSQLVSFRKIS*

>ModoOR1.2.12

MDVRNQSIVSEFVLMGFTNFWAMQLFLFVFSSVLFLAIILGNSLIVLMVA

FDSHLQSPMYFFLAQLSFIDMIDSCMVTPKMLADIFRKHKLISFGGCVSQ

IFFIHAVGATEMVLLLAMAFDRYIAICKPLHYLTIMNLRVCISIVVTSWT

IGISHSFIQLAFVIKLPFCGPNKIDSFYCDLPRFIRLACTDTYKLQFMVT

ANSGFISLGAFFILIISYFVILISVRQHSSGGSAKALSTLSAHIAVVILF

FGPCIFDYVWPFPTWPVDKYLALFDSIITPFLNPIIYTLRNNEMKVAMKK

VCNLMRLRFMRLTRSQIIS*

>ModoOR1.2.13

MDVRNQSIVSEFVLMGLTNFWVMQLFLFVFSSVLCLASILGNSLIVLTVA

SDPHLQSPMYFFLAHLSFIDIIDSCIATPRMLTDLFKKHKLISFGGCVSQ

IFFIHAVGATEMVLLLAMAFDRYIAICKPLHYLTIMNLRVCISIVVTSWT

IGISHSFIQLAFVIKLPFCGPNKIDSFYCDLPRFIRLACTDTYRLQFLIT

ANSGFISLGAFFILIISYFVILISVRQHSSGGSAKAISTLSAHIAVVILF

FGPCIFVYVWPFPTWPVDKYLALFDSIITPFLNPTIYTLRNKEMKVAMKK

AWDLMRLRFMRLTRSQIIS*

>ModoOR1.2.14

MDVRNQSIVSEFVLMGFTNFWVIRLFLFVFSSVLFLAIILGNSLIVLMVA

FDSHLQSPMYFFLAQLSFIDMIDSCMVTPKMLADIFRKHKLISFGGCVSQ

IFFIHAVGATEMVLLLAMAFDRYIAICKPLHYLTIMNLRLCISIVVTSWM

IGISHSFIQLAFVIKLPFCGPNKIDSFYCDLPRFIRLACTDTYKLQFVVT

ANSGFISLGAFFILIISYIVILISVRQHSSGGSAKALSTLSAHIAVVILF

FGPCIFKYVWPFPTWPVDKYLALFDSIITPFLNPIIYTLRNKDMKVVMKK

VWNLMRLRLMTLTRSQIIS*

>ModoOR1.2.15

MLIPMDGTNHSMVSEFVLIGLTNSWVLQFFLFVFSSLFCLASMLGNSLIV

LTVVSDSHLQSPMWFLLAHLSLIDMIDSCMVTPKMLADIFRKHKLISFGG

CVSQIFFIHAVGATEMVLLLAMAFDRYIAICKPLHYLTIMNLRVCISIVV

TSWMIGISHSFIQLAFVIKLPFCGPNKIDSFYCDLPRFIRLACTDTYRLQ

FLVTANSGFISLGAFFILIISYIVILISVRQHSSGGSAKALSTLSAHIAV

VILFFGPCIFVYVWPFPTLPVDRYLALFDSVITPFLNPTIYTLRNKDMKV

SMKRLWSQLMRSPNIS*

>ModoOR1.2.16

MDGVNRSVVYEFVLMGLTNSWGLQLFLFLFSSIFYTASMLGNFLIVLTVI

SEPRLHSPMYFLLANLSFIDMIDSCIVTPKMICDLFKKHKSISFGGCVSQ

IFFIHCIGGVEMVLLIAMAFDRYIAICKPLHYLTIMNLRICIGILISGWI

IGLTHSLIQLGFVIQLPFCGPNKVDSFYCDLPRFIKLACTDTYKLQFLVT

ANSGFISLGAFFILIISYVIILATVRQHSSSGGSSKALSTLSAHITVVIL

FFGPCIFVYVWPFPTLPLDKFLAIFDSVITPFLNPTIYTFRNKEMKVAMK

RLWSQLLKSQTFS*

>ModoOR1.2.17

MEGANNSVVSEFVLLGMSTSWEMKILLFLFFFSFYVGIMLGNFFIVFTVI

FDSHLHSPMYFLLANLSLIDLGLSSTTVPRMIIDIFSEHRVISFPGCMVQ

IFFIHVMGGTEMVLLIAMAYDRYTAICKPFHYLTIMNYKTCTCFVVAAWV

IGVIHAVSQFVFVVNLPFCGPNKVDSFYCDIPKVAILACTDTYWLEYVVT

ANSGLISMGTFFLLILSYIFILVTVRHHSSAGLSKAFSTLSAHITVVFLF

FVPCFFVYVWQVPTLSLDKFLLILVFLVTPLLNPAIYTLRNKDMKLAMKR

LTEKIVRSR*

>ModoOR1.2.18P

FLLVNLSLTDCGLSSTIVSRMVIDIFRKYKVISFPQCMIQIF*IVKLGIP

VLFVWAISKPLH*LIIMNYERCIHFAVIARRVGTFYSLSWFVFIIDLPFC

SPNKLENFTVNLPRL/HIIILVTDRHCSIVGLSKAFSTLSVHLSVIFLFF

CSIFL*LYVARFLNKILIIFIFLIMPVFNPIIYTMRNKDMKMAVK*LTKK

IVG

>ModoOR1.2.19

MIMTGEMDGENHSVVTEFVMLELSASWEMNILLFLFFFSFYVGIVLGNLF

IVFTVTFDNNLHSPMYFLLANLSLTDFGLSTTTVPRMIIDIFSEHKVISF

LECMIQMFFIHVMGGTELVLLIAMAYDRYTAICKPLHYLIIMNRKACIRF

AVVAWVIGILHSISQFVFVIDLPFCGPNKLESFYCDFPQVAKLACRDTSR

VVFTMTLNNGLVTMGTFFLLIISYIIILITVRHHSSVGLSKAFSTLSAHI

SVVFLFFAPCFFVYVWQVPTLSLDKFLLIFIFLITPLLNPAIYTLRNKDM

KMAVKRLINKIAGSRELS*

>ModoOR1.2.20P

FSFSYSFTIRQENIFIVFIMVYDSHMHSLLYF*PASLSLIDVGLSPTVVP

QMITGVFSEYKVVSFPSIMEMFFYPCCGRN*VGTPCIMAYDRYMVIYKLL

YFLTIMNHKPILFPISFFIIFLHFFGPNHVYSFYCDIPQMVVLICIDTS*

IEYMVSANNTLISRSTFS*SSLSSLSWLLLGTILQLIYRKLSL*S*FISL

*CFTLLYLYVTISQPLIGHILFVIVFLITLILDTANYISKNEDMELTMNG

LVNQILNSR

>ModoOR1.2.21

MTVMPTVSMDGANDSVVTEFVLLGLSASWEMKVLLFLFFFSFYVGVVLGN

LFIVFTVVCDSHLHSPMYFLLANLSLIDLGLSSTTAPRMITDIFSEHKVI

SFPGCMIQMFFVHVMGGTEMVLLIAMAYDRYTAICKPLHYLTIMNHKTCI

SFVVVAWVIGVIHAVSQFVCIVNLPFCGPNKVDSFYCDFPRVAELACTDT

DWVEDVVTANSGLISMGTFFLLIISYIFILVTVRHHSSAGLSKAFSTLSA

HITVVFLFFGPCFFMYVWEFPTLSLDKFLAILDFVVTPLLNPAIYTFRNK

DMKVAMKRLSRKIVSSKDSS*

>ModoOR1.2.22

MSIMPTISMNGANDTVVTEFVLLGLSASWGMKVLLFLFFFSFYVGIVLGN

LFIVFTVVCDPHLHSPMYFLLANLSLIDLGLSSTTAPRMITDIFSEHKVI

SFPGCMIQMFFVHVMGGTEMVLLIAMAYDRYTAICKPLHYLTIMNHKTCI

SFVVVAWVIGVIHAVSQFVCIVNLPFCGPNKVDSFYCDFPRVAELACTDT

DWVEDVVTANSGLISMGTFFLLIISYIFILVSVRHHSSAGLSKAFSTLSA

HITVVFLFFAPCFFVYVWEFPTLSLDKFLAILDFVVTPLLNPAIYTFRNK

DMKVAMKRLSRKVLSSKETS*

>ModoOR1.2.23

MDGANDSVVTEFVLVGLSVSWEMNIFLFFFFSLFFVGIVLGNLFIVFTVI

FDPHLHSPMYFLLANLSLLDLGLSATTVPRMIADVLSKYRIISFPSCMTQ

IFFIHVMGGTEMVLLIAMAFDRYTAICKPLHYLTIMSPKTCVCFVVAAWV

IEVIHAVSQFIFVINLPFCGPNKVDSFYCDFPRVTKLACADTHTVDYVVT

ANSGLISMGTFFLLIISYIFILITVSHHSSAGLSKAFSTLSAHITVVFLF

FTPCFFVYVWPFPTLSLDKFLIILDFVVTPVLNPAIYTFRNKDMKVAMKR

MSRMIIGSKESS*

>ModoOR1.2.24

MDGVNNSVVNEFVLLGLSASWEMKILLFFFFSVFFVGIMFGNLFIVFTVI

FDPHLHSPMYFLLANLSLIDLGLSATTVPRMIVDLFSECNIVISFPGCMT

QMFFIHVMGGTEMVLLIVMAYDRYTAICKPLHYLTIMNPKMCICFVVTAW

IVGVIHAISQFVFVINLPFCGPNKVESFYCDFPRVIKLACIDTYSLEFVV

IANSGFISIGTFFLLIISYIFILASVRQHSVGDLSKAFFTLSAHITVVIL

FFGPCMFLYVWPFPTLSLDKFFAIVDFVVTPVLNPAIYTLRNKDMKMAMR

RLSSQIVSSRETL*

>ModoOR1.2.25

MNELNHSMVSEFVFLGISSSWAIQLGLLLFSSFFYIVIVLGNIFIVLIVS

TDGHLHSPMYFLLANLSFIDLCLSTVVVPKMISDLLYEHKVISFQGCITQ

IFFIHLMGGTEMVLLIIMAFDRYVAICKPLHYLTIMNPKMCIFLQVSSWV

IGLIHSLTQLAFVANLPFCGPNEIDSFYCDLPRFIKLACIDSYKMEFIVT

ANSGFISIGTICLLFISYIYILVTVHKHSSGLSKALSTLSAHISVVILFF

GPCIFVYVWPFPTLPVDKFLAIIDFIITPVLNPSIYTFRKQDMKAVMKRL

SSRLVNFRKIL*

>ModoOR1.2.26P

MNELNHSMVSEFVFLGISSSWAIQLGLLLFSSFFYIVIVLGNIFIVLIVS

TDGHLHSPMYFLLANLSFIDLCLSTVVVPKMISDLLYEHKVISFQGCITQ

IFFIHLMGGTEMVLLISMAFDRYVAIYKPLHYLTIMNPKMCIFLQVSSWV

IGLIQSLTQLAFVANLPFCGPYGN*QFLL*SSSESSNWPA*IHIRWKFIV

TANSGFISIGTICLLFISYIYIFVTIQK

>ModoOR1.2.27

MNELNHSIVSEFVFLGISSSWAIQLGLLLFSSFFYMVIVLGNVFIVLTVS

TDGHLHSPMYFLLANLSFIDLCLSTVAVPKMIVDLLYEHKVISFQGCITQ

IFFIHLMGGTEMVLLISMAFDRYVAICKPLHYLTIMNPKMCIFLQVSSWV

IGLIHSLTQLAFVANLPFCGPNEIDSFYCDLPRFIKLACIDTYKLEFIVT

ANSGFISIGTICLLFISYIYILVTVHKHSSSGLSKALSTLSAHISVVILF

FGPCIFFYVWPFPTLPVDKFLAIIDFIITPVLNPSIYTFRNKDMKAAMKR

LSSRLVSFRKIL*

>ModoOR1.2.28

MDRGNQSVVSEFVLLGLTDSWELQICLFLLFSVIYLATVLGNILIMTTVI

ADSHLHSPMYFLLANLSFVDLWLSSVTIPKAIIDFFRESKTISFGTCMWQ

VVFVHFAGGGEMVLLVTMAYDRYVAICKPLHYTTIMSQQRCIWLVVISWA

IGLVHSISQLAMIVDLPFCGPREIDSFFCDLPLVIELACMDTYILGSLVN

ADSGFLATACFILLLISYVYILLTVRHHSTDGVYKALSTCTAHIMVVLIF

FVPCIFIYLCPLNIPSVDKFLAVFYAVFTPLLNPSIYTLRNKEMKEAMKR

LSM*

>ModoOR1.2.29

MDEANNSVVSEFVLLGLCNSRQLQIFLFLLFSVLYLTIILGNILIILSVI

NDPNLHSPMYFLLANLSFIDLWLSSVTTPKMIIDFLRESKTISFGGCLCQ

IFFGHFVGGGEMVLLVAMAYDRYVAICKPLYYTTIMNQKRCIGLVVISWA

VGFVHSVSQLAIIIDLTFCGSKKMDSFFCDLPLVIELACMDAHVLRLIVN

IDSGILALICFFLLLISYAHILLTVCHHSMDGSTKALSTCTAHIMVIVIF

FGPCIFIYLWPLKIASVDKFLAVFYTVFTPLLNPAIYTLRNKEMKVALKK

WGSQYVNFKRNS*

>ModoOR1.2.30P

MTGMNRSTVSEFVLLGLCNSRELQIFLFILILVLYLVIVLGNIFIVILVI

IDLHLHSPMYFLLANLSFVDLWLSSVTTPKMITDLLKENRS/YSRKTEAI

SFEGCMCQILFVHFVGGGEMVLLVVMAYDRYIAICKPLYYTVIMTKGRCI

CLVMMSWTIGFVHSISQLVVIVKLPFCGPRVVNSFFCDIPLVIELACIDS

YVLGILMNADSGVLAMTCFILLLISYTYILLTVCHHSKDGAYRALSTCTA

HIMVVVLFFGPCIFIYVWPLNITSVDKFLAVFYSVITPLLNPAIYTFRNK

EMKVALRRFKSHY

>ModoOR1.2.31

MDLTNDSVISEFILLGLTNSWSLEILFFAIFFLAYALIMTGNILIIIIVI

FDSHLHSTPMYFLLANLSFLDMTISTITVPKMIRDCLSEHKTISKWGCMA

QMFFLHFLGGSEMTLLIFMAVDRYVAICKPLHYTSIMNYRTLVAFVLLSW

VVGFVHTMSQMAFTVNLPFCGPNLVDNIFCDLPLVIKLACADTYVLDLLV

IADSGLLSLICFILLLVSYTVILVTVHRRSSGGLTKAMSTLSAHITVVTL

FFGPCIFIYASPFSSFSVDKFLSVFYSVITPLLNPIIYTLRNQDMKAAMI

RLRSRHVSSRQTL*

>ModoOR1.2.32

MKNNSVLNEFILLGLTNSWELEILFFVIFFLAYTSIMAGNSLIILMVIFD

SHLRSTPMYFLLANLSFLDMTLSTVTVPKMITDFLRDRKTISLWGCMAQM

FLVHLLGGSEMSLLVVMAVDRYVAICKPLHYTTIMNHRILLGSVFISWVI

GFVHTMSQMAFVVTLPFCGPNVIDDVFCDLPLVMNLACTDTYILDLLVIA

DSGLLSLICFLLLLISYVVILLTVHRRSSGGLSKALSTLSAHITVVTLFF

GPLILIYAWPVSSYTLDKFLSVFFSVVTPLLNPIIYSLRNREMKAAMVRL

RSRHISSGPPL*

>ModoOR1.2.33

MELKNHSFINEFILLGLTNSWELEIFFFVIFFLAYTSILAGNSLIILMVT

FDSRLNSSPMYFLLANLSFLDMTLSTVTVPKMITDFFRERKTISLWGCMA

QIFLVHLLGGSEMVLLIVMAIDRYIAICKPLHYTTIMNWRILVGSVLLSW

VVGFVHTMSQMAFAVTLPFCGPNVIDDVFCDLPLVINLACTDTYVLDLLV

IADSGLLSLISFVLLLISYIIILVTVQRRSSSGLSKALSTLSAHITVVFL

FFGPSILIYAWPVSSYALDKFLSVFFSAVTPLLNPIIYSLRNQEMKTAMV

RLRSRHISSRPTL*

>ModoOR1.2.34P

AICKLFHYIVVMNHQILIGSMLLSWNIAFVHTMSQRAFMMSLSFWGPNVI

DDVFCDLLILKLVCPDTYVLEQLVALMGLLSLISFIILLISYIILATV*H

HLSSGLSKALS

>ModoOR1.2.35

METKNNSMINEFILLGLTNSWELEIFFFVIFFFAYMSILAGNCLIILMVI

FDSHLHSTPMYFLLANLSFLDIVLSTVTVPKMIIDFFKKKKTISFWGCMA

QIFLAHLFGGTEMTLLIVMAIDRYVAICHPLHYTTIMNYRILVGGVLLSW

TVGFVHTMSQMAFMVSLPFCGPNVIDDVFCDLPLVLKLACTDTSVLELLL

VIFSELLSLIAFITLLMSYIIILVTVWHRSANGLSKALSTLSAHITVVIL

FFGSLILIYGLPISSYTIDKFLSVFYSVITPLLNPVIYSLRNQEMKAAMT

RLRSWSINFKPTF*

>ModoOR1.2.36

MEMNNSMVNEFILLGLTNSWELEIFFFVVFFLAYTLILTGNFLIILMVTL

DSHLHSTPMYFLLANLSLFDMSLSTVTVPKMIIDFFRERKTISLWGCMAQ

MFLAHLFGGSEMTLLIVMAVDRYVAICKPLHYTTIMNRRILVGSVLLSWI

VGFVHTMSQMAFMVNLPFCGPNVIDDVFCDLPLVLKLACTDTYILEMLVI

ADSGLLSLISFILLLVSYTIILVTVWHRSASGLSKALSTLAAHITVVILF

FGPITLIYAWPVDSYTLDKFLSVFYSVITPLLNPIIYTLRNQEMKAAMIR

LRSRHISSKPIF*

>ModoOR1.2.37P

FSFLVILVYMSISAGNGFIVLLVTLDSCLHSTPMYFLLVNFYFLDIVLST

VTVFKMIINFFKRKKTISF*GFMAQIFLAHLFGGTEMTLLIVMAIDRYVA

ICHPLHYTTIMNHRILVGSILLSWTVGFVNTMSQVAFMVSLPFCGPNVID

DVFCDLPLVLKLACTDTSVLELLLVVFSELLSLIAFITLLMSYIIILVTV

WHRSANGLSKALSTLSAHITVVILFFGSLILIYGLPISSYTIDKFLSVFY

SVITPLLNPVIYSLRNQEMKAAMTRLRSWSINFKPTF

>ModoOR1.2.38

MEMNNSVVNEFILLGLTNSWELEIFFFVVFFLAYTLILAGNFLIILMVTL

DSHLHSTPMYFLLANLSLFDMSLSTVTVPKMIIDFFRERKTISLWGCMTQ

MFLAHLFGGSEMTLLIVMAVDRYVAICKPLHYTTIMNRRILVGSVLLSWI

VGFVHTMSQMAFMVNLPFCGPNVIDDVFCDLPLVLKLACTDTYILEMLVI

ADSGLLSLISFILLLVSYTIILVTVWHRSASGLSKALSTMAAHITVVTLL

FGPVILIYAWPVGSYTLDKFLSVFYSVITPFLNPIIYSLRNQEMKAAMIR

LRSRHINSKTIF*

>ModoOR1.2.39

MDLMNKSIVSEFVLLGLSGTWTLQIFYFLIFFMLYGATVVGNLLIMVTVT

FSSHLHSPMYFLLGNLSFFDMCLSTVTTPKMIADLLRKQRTISLWGCVTQ

MFFMHLFGGAEMTLLIAMAFDRYVAICKPLHYTSIMSRRLLYGFVLFSWI

IGFIHTMSQIVLTVNLPFCGPNVVDNLFCDLPLVIKLACKDTYILELFVI

ADSGLLSLICFILLLVSYTVILVTVHNHSSGGLSKALSTLSAHITVVTLF

FGPCIFIYAWPFSNFSGTKILSVFYTVITPLLNPIIYTLRNQEMKVAMKK

LRIQRISSR*

>ModoOR1.2.40

MSDQTLRVESMEQRNFSMVAEFILLGLTESRELQIFFFVFFSIIYTVTVF

GNLFIIFTVIFNSHLHSPMYFLLANLSFIDMSLASFATPKMIHNLASKHK

AISYQGCMAQMFFLHLFGGSEMMLLVAMAIDRYIAICKPLHYSTIMNHRA

CFGLVLLSWTTGFVHTMSQMVFTVTLPFCGPNIVDSFFCDLPRVIKLACL

DTYILELLVIADSGLLSLICFILLLISYSIILVTIRLHSSSGSSKALSTL

SAHITVVVLFFGPCIFIYVWPFSSISTDKTLSVFYTIFTPLLNPIIYTFR

NKDMKRAIRRLKHQHVNCIRTS*

>ModoOR1.2.41

MEPKNNSVVSEFILLGLTKSQNLQIFFFLGFSLVYIGIVLGNLLILITVA

FDSHLHTPMYFLLANLSLIDMILGSFATPKMIVDFLREQKTISWWGCFSQ

MFFMHILGGSEMMLLVAMAIDRYVAICKPLHYMTIMNHRVLLGLILTSYA

VGFVHSSSQMAFMLNLPFCGPNTVDSFFCDLPLVIKLACRDTYVLQLLVI

ADSGLLSLICFILLLFSYTIIIYSVRHRASGGSSKALSTLSAHITVVTLF

FAPCVFIYVWPFSRYSVDKILSVFYTIFTPLLNPIIYTLRNQEVKAAIKK

IRNQHINSKHNL*

>ModoOR1.2.42

MDHQNYSVVSEFVLRGLSNSLQLQLFFFVFFSLVYIATVLGNLLIVVTVI

SEPRLYSSPMYFLLGNLSFLDMWLASFATPKLIKDFLSEMKLISFGGCMA

QIFFLHFIGGAEMVLLVTMAYDRYVAICKPLHYLTIMSWRTCIGLVMISW

TIGFIHSISQIAFTVNLPYCGPNIVDSFFCDLPRVIELACTDTYVLGILM

IAHSGLLSISCFLLLMISYTIILVTVQQHSSRSASKALSTCSAHIMVVTI

FFGPCIFIYLWPFSRFSVDKVLSVFYTIFTPLLNPIIYTLRNEDMKTAMK

KLRIQHKFFH*

>ModoOR1.2.43

MNGTNHSRVSEFILLGLSDSPELQPLFFVVFSVLYVAIVMGNFLIILTVT

SDPRLHSPMYFLLANLSFIDVCVASFATPKMIADFLVEQKTISFEACLAQ

IFFVHLFTGSEMVLLVSMAYDRYVAICKPLHYMTIMSRRVCIILVIISWC

VGFIHTTSQLAFTVNLPFCGPNKVDSFFCDLPLVTKLACLDTYVVSLLIV

ADSGFLSLSSFLLLVISYTVILITVQSRSSASMAKARSTLTAHITVVTLF

FGPCIFIYVWPFSSYSVDKVLAVFYTIFTPILNPTIYTLRNKEVKAAMSK

LKSRYLNTNKVSVVIRSVFSLEPK*

>ModoOR1.2.44

MEWSNKSVVTEFILLGLSSSWGLQLSLFFVFSLFYGAAVLGNLLIILTVI

TDSRLHSPMYFLLSNLSFIDVCQATFATPKMIADFLNEHKTITFEGCMSQ

IFFLHVFGGSEMVLLVAMAYDRYIAICKPLHYMTIMSRRVCTVLVSVSWA

IGILHSASHLVFTVNLPFCGPNKVDNFFCDLPLVIKLACLDTYFLEILVL

TNSGLLSLICFILLLISYTIILVTVRSRSSTGTSKALSTLSAHVTVVVLF

FGPLIFIYIWPFDSFPIDKFISVFFTVFTPFLNPIIYTLRNEEVKAAMRK

LRSRHIGSKQIS*

>ModoOR1.2.45

MAGGNQSVVSEFVLLGLSNSRELQFFFFVIFTIVYVASVLGNIIIILTIV

SDSHLNSPMYFLLSNLSFIDICQSNFATPKMIADFLVEKKVISFEGCMAQ

IFLLHSFVGSEMMLLVAMAYDRFIAICKPLHYSTIMNRRLCIIFVAISWA

VGILHSVSHLAFTIDLPFCGPNEVDSFFCDLPLVIELACMNTYEMEIMTL

TNSGLISLSCFLALIISYTVILVIVRRRSSSGSSKALSTLTAHVTVVILF

FGPCIYFYIWPFSRLSIDKFLSVFYTVCTPLLNPIIYSFRNEDVKSAMRK

LKNRYLNSWK*

>ModoOR1.2.46

MEGANSTVVSEFVLLGLSSSQELQLFFFIFFSLLYGLIILGNLLIIITVT

FDAHLHSPMYFLLGNLSFVDICQSSFATPKMIADFLNEHKTISFNGCIAQ

IFFIHLFTGGEMVLLVSMAYDRYVAICKPLHYVTIMNQNVCIALVLISWA

VGFVHTMSQLSFTVNLPFCGPNVVDSFFCDLPRVTKLACLDSYTIEILIV

VNSGILSLSTFFLLLISYIVILLTVRYKSSAAMAKALSTLSAHIMVVILF

FGPCIFIYVWPFTTYPVDKVLAIFYTIFTPILNPIIYTLRNKDMKAAMRK

LMTRNLKPKKISEMSLMMRSSLY*

>ModoOR1.2.47

MDKSNYSGVTEFILLGLSGSTELQIFYFLFFTMFYVAILLGNFFIVLTVI

SEPVLHTPMYFLLSNLSFIDVCLSTFATPKMITDFLLEHKSISFNGCMTQ

IFFLHVFAGGEMLLLVAMAYDRYVAICKPLHYATIMSLHKCAGLVVASWV

IGLLHSLSQLAFTVNLPFCGPNEVDSFFCDLPLVIKLACVDTYILEVMML

SDSGLMAMVSFILLLISYTVILVTMKYRSSASMAKARATLTAHITVVTLF

FGPCIFIYAWPFSNFPVDKVLSVFYTVFTPLLNPIIYTLRNKEMKSAMQK

LRGQLLNSRGLSQFLARRALHN*

>ModoOR1.2.48

MDQGNHSRVTKFVLVGLSTSWELEILFFVLFTLLYMAIVLGNLLIVLTVM

LEPVLHTPMYMMLSNLSVLDICLATYATPKMIIDFLAELKTISFEGCMAQ

IFFLHVFAGGEMVLLVAMAYDRYVAICKPLHYAAIMSFNKCIGLLVASWV

IGILHSLSQLAFTMNLPFCGPNIVDSYYCDLTLVIRLACTDTYVPEVLML

LDSGLMGVTSFLLVLISYTVILVTVQHRSSASMAKARATLTAHITVVTLF

FGPCIFIYVWPFSSFPVDKIFSVFSTVFAPILNPIIYTLRNKEVKAAMQK

LKSQHINVRQIFLPTPVSTDMMP*

>ModoOR1.2.49

MDIGNKSTVSEFVLLGLSSSWDLQLFFFMVFSLFYVATMVGNCLIVITVI

ADSHLHSPMYFLLTNLSLIDMSLASFATPKMITDYLTGHKTISFDGCISQ

IFFLHLFTGTEIILLMAMSFDRYIAICKPLRYATIINPRVCLMLVVTSWV

VGVMHSMSQVIFALHLPFCGPNEVDSFFCDLPVVFQLACVDTYVLGLFMI

STSGIIALSCFILLFNSYVIVLVTIKHHSSGGSSKALSTCTAHFIVVFMF

FGPCIFIYMWPLSSFLVDKVLSVFYTIFTPILNPIIYTLRNQEVKTAMRK

LKNRYLNSDKISQSPHYS*

>ModoOR1.2.50

MSATLMEAANYSGVSEFVLLGLTDSPELQAFFFVVFSVLYVVTILGNCLI

LLTVISIPQLHSPMYFLLGNLSFIDMCLSSFATPKMIMDFFFHRKTISFE

GCISQIFFLHLFTGTEIVLLISMSFDRYIAICKPLRYSNIMSQRVCLGLV

VVSWTVGFLHTMSQLAFTLYLPFCGPNVVDSFFCDLPLVIQLSCIDIYVL

GLFMISTSGMIALVSFLLLLTSYIIVLVTMKKHSSGGSSKALSTCTAHFI

VVFMFFGPCIFIYVWPFTNFLVDKVLSVFYTIFTPFLNPLIYTLRNQEVK

AAMRKMSNQYINFRKTAQFPRYPVQ*

>ModoOR1.2.51

MEQENSTVTEFTLLGLTESREIQLFVFVLIFSFYMIVLPGNLLIIITIRS

DPALTAPLYFFLGNLAFLDASYSFIVAPKMLVDFLYEKKTITYQGCITQL

FFLHFLGAGEMFLLVVMAFDRYIAICQPLHYANIMNRQVCYALLLALWIG

GFTHSIVQVALIVRLPFCGPNQLDNFFCDVPQVIKLACTDTFVVELLMVS

NSGLLTLLCFLGLLTSYAVILFRVRGSSSEGKSKALSTCTTHVIIVFLMF

GPAIFIYTRPFRTFPADKVVALFHTVIFPLLNPVIYTLRNQEVKSSMKKL

VNRQVLCGVE*

>ModoOR1.2.52

MELENSTVTEFILLGLTQSQEIQLLVFVLIFSFYMVILPGNLLIIITIRS

DPALTAPLYFFLGNLAFLDASYSFIVAPKMLVDFLYEKKTISYQGCITQL

FFLHFLGVGEMFLLVVMAFDRYIAICQPLRYAVIMNRQVCYALLLALWIG

GFTHSIVQVALIVRLPFCGPNQLDNFFCDVPQIIKLACTDTFVVELLMVS

NSGLLTLLCFLGLLTSYAVILFRVRGSSSEGKSKALSTCTTHVIIVFLMF

GPAIFIYTRPFRTFPADKVVALFHTVIFPLLNPVIYTLRNQEVKSSMKKL

VNRQVLCGVE*

>ModoOR1.2.53

MESENYTRVTEFVLTGLSQTREVQLILFVIFLSFYLIILPGNVLIIFTIR

YDAHLTSPMYFLLANLAFLDIWYSSITAPKMLVDFFAERKIISFGGCIAQ

LFFLHFVGASEMFLLTVMAFDRYAAICRPLHYATIMNKRLCCVLVACSWF

GGFIHSIIQVALIIRLPFCGPNELDSYFCDITQVVRIACANTFPEELVMI

FSSGLISVVCFIALLMSYAFLLVMLKKHSGSGESTSRAMSTCYSHMTIVV

LMFGPSIYIYARPFDAFSLDKIVSVFHTVIFPLLNPIIYTLRNKEVKTAM

RKMINKYILCKGK*

>ModoOR1.2.54

MEVANNITEFLFLGLSQDPKLQLMFFFLFLLFYTVIIVGNLLILFTVCFE

SKLHSPMYFFLSNLSFVDIAYSSATAPKMIADFISEDKTISYWGCVTQMF

TFHFFGCAEIFVLTVMAFDRYAAICQPLRYSTIMSANTCAMLAFLSWAGA

LGHSFVQTLLTFQLPYCSARVIDHYFCDVHPVLKLACADTSLVNLLVVAN

SGLISLGCFLILLASYIVILLSLRKRSSESRKKALSTCGSHLTVVTFFFV

PCIFIYLRPSTTFPLDKAVSVFYTTITPMLNPLIYTLRNEDVKNAVKHFW

RHRVLGEEKKG*

>ModoOR1.2.55

MLSSLKGGNDSRVMEFVLLGLSSTWELQLLLFLIFSMFYMAIVLGNILII

VTVKADGHLIQSPMYFFLAHLSFIDLCLGCVTVPKMLGDFLHQRKIISFP

GCLAQIYFLHFLGASEMFLLTVMAYDRYVAICNPLHYLTVMHRQLCFNLV

AASWFGGFLHSITQVLLVIQLPFCGPNELDNFYCDVPQVIKLACMDTYVV

EVLMVSNSGLLSLICFLILLLSYAVILATLRTRFRQGQSKALSTCASHLT

VVSLIFVPCVFIYLRPFCSFPVDKVFSVFYTVITPLLNPLIYTLRNSEMK

TAMRKMRKKYAVSCFLAKG*

>ModoOR1.2.56

MKNSERSNHSGSVSEFVLLGFPGPWEIQIFLFSFFSGTYILTLIGNLSII

CAVNLDQKLHTPMYIFLANFSFLEICYVTSTVPNMLANFFSENKIISFTG

CFLQFYFFFSMGTTETFFLSAMAYDRYLAICQPLHYPTVMTLLKCKKMVA

CCWVCGFSSYLLPVYLISQLPFCDHNTIDHFICDPGPLIGLSCVPAPATE

IIFSVFNSVLIFSTFLFITSSYTLVIRVVLKIPSAEGRRKAFSTCGSHLA

VVSLFYGSIMVMYVSPTSGNPAGIQKIVTLFYSVLTPLFNPLIYSLRNKE

MKASLKKVFGSMKFGQHV*

>ModoOR1.2.57

MASEERNTSDIENMVNSVSHFILLGFPSSREIQMVYFVVFSTTYILTLMG

NAAIICAVHWDRHLHTPMYILLGNFSFLEICYVTTTVPNMLANFLSETNT

ITFVGCFVQFYFFFSFGCDEGFYLCIMAFDRYLAICRPLHYPNIMTTHLC

TDLVIFGWSSGFILFVIPVVLISQLYYCGPNIINHFICDPVPLMALSCSK

AHTTKLIYSTFNTIFMVGTFIFVLISYALVIFSVLRIPSAAGKRKAFSTC

ASHLAIVILFFGSVMTMYVGPGSEQPVEFQKVMTLFYSVITPLFNPLIYS

LRNKDMKAALRKVLVATRTSHKT*

>ModoOR1.2.58P

GNTSIICAVWSSRQLHTPMYFLLANFSFLEMCYVSSDVPKLLANILSQKK

TISFAGCILQFYFFFSMCASEGYFLSAMAFDRYLAICRPLHYPTIMTQSL

CAKLVFFCWLGGFLSILVPAILISKVPFCGPNIIDHFFCDLGPLLALSCV

PVPKTTLTCATVSSLIIFITFLYILASYTRVLKAVLKVPAGTGRNKGF/F

VVVSLFYGSVMVMYVSPGSKNQPGMRKFVTLFYCMATPFFNPLIYSLRNK

DMKDALKKVLSGLSQDFPKGHEK

>ModoOR1.2.59P

NISGTVSEFILLGFTCQKETQIILLMLFSIIYILTIMGNTCIIYAVWSSQ

QLHTPMYTLLANFSFLEICYVNSDVPKMLVNMLSKTKTISFTGCLLQFYF

FFSMCASECLFLSVMAFDRYLAICQPLHYSTIMTTLLC/RFCANLAFFCW

VGGFLWLLTPVILISQVPFCGPNIIDHFLCDLGPLLALSCAPVPNTTLAC

GAISSLIIFITFLYILGSYVQVLRAVLRVPSGSGRNKAFSTCASHFTVVS

LFYGSVMVMYVSPGSGNQFGMEKFVTLFYSMATPFFNPLIYSLRNKDMKK

ALKKLL

>ModoOR1.2.60

MRILGVTNISGTVSEFILLGFTCQKETQIILLVLFSIIYILTIIGNTFII

YAVWSSQQLHTPMYTLLANFSFLEICYVNSDVPKMLVNILSKTKTISFTG

CLLQFYFFFSMCASEGLFLSVMAFDRYLAICQPLHYSTIMTQRLCANLAL

FCWVGGFLWLLTPVILISQVPFCGPNIIDHFLCDLGPLLALSCAPVPNTT

LACGAISSLIIFITFLYILGSYVQVLRAVLRVPSGSGRNKAFSTCASHFT

VVSLFYGSVMVMYVSPGSGNQSGMEKFVTLFYSMATPFFNPLIYSLRNKD

MKKALKKLLSGFSHRFPKRHQK*

>ModoOR1.2.61P

LVNFLSKTKAISFTRCLLQLYFFFSLGITECLFLSIMDYDHFLAICLPLH

YPTIMTTKFCSHLVIFCWVYGFLWFLVPVVLVIQLPFCGPNVIDDFLCDL

GPLLDLASACVPIPGTVLICGTMSSLLIFAIFYITGSYTLMLRAMLRVSS

APGLLKAFSTCSSHLAVVLLFYGSVMMTYVSPGSGLAEGMQKFTTLFYSV

MTPLFNPLIYSLQNKEMKGALKKVLGSA

>ModoOR1.2.62P

MNIARGNRVTEFILLSFHCPREMQILLFGVLFLTYILTLMGNESIICAVK

VDHQLHTPMYILLANFSFLK/FLKNCYINTTVHNMLASFLSETKTISFTA

CFIQTYFFCSTGITETFFLSLTAFEGYLVICGHLHYPTIMTGHFHHSSAE

KMASSTYFIT*SSLCGLNSNDCVFFFVTQEHY*FFSCSLPPRLSSFYSIL

SFLIIFLIFPFILGS*TLMLRTILKVPSAASCNKAFSKCGS

>ModoOR1.2.63

MKNSERSNHSGSVSEFVLLGFPGPWETQIFLFSFFSGTYILTLIGNLSII

CAVNLDQKLHTPMYIFLANFSFLEICYVTSTVPNMLANFFSENKIISFTG

CFLQFYFFFSMGTTETCFLSAMAYDRYLAICQPLHYPTVMTLLKCKKMVA

CCWVCGFSSYLLPVYLISQLPFCDHNTIDHFICDPGPLIGLSCVPAPATE

IIFSVFNSVLIFSTFLFTTSSYTLVIRIVLKIPSAEGRRKAFSTCGSHLA

VVSLFYGSIMVMYVSPTSGNPAGIQKIVTLFYSVLTPLFNPLIYSLRNKE

MKASLKKVFGSMKFGQHV*

>ModoOR1.2.64P

IIYILTIMGNTCII*AVWSNQQLHTPMYTLLANFSFLEIYVNSDVPKMLV

NILSKIKTISFTGCLLQFYFFFSMCASEGLFLSVMAFDQYLAICQPLHCS

TIMTQHLCAN/CSVGGFLWLLTPVILISQVPFCGPNIIDHFLCDLGPLLA

LSCAPVPSTTLACGAFSSLIIFITFLYILGSYVQVLRAVLHVPSGSGRNK

AFSTCPSHFIVVSLFYGSAMVMNVSLGSGNQSGMKFVTLFYSMATPFFNP

LIYSLRNKDMKKALKKLLSEL

>ModoOR1.2.65P

MTILGIHNISWTVSEFILLGFPYSQEIEILLFVLFSIMYTLTLMGNGSIV

FAVWWDQQLHTPMYILLANFSFLEICYISSSVPIMLTNLLSRAKTISYNG

CMLQFYIFSSLCATELFFLALMAFDRYIAICYPLHYPTIMTGQLCATLVS

ICWLGGFIWLLTPVTLLSQVPFCGPNIIDHYLCDLGAMLALSCTPVSKTT

LTCGAVSTLI/IYILGSYTQVLRAVLRVPKGSGRQKAFSTCASHLGVVSL

FYGSIMMMYVSPGSASQPEMQKFITLFYSVATPFFNPMIYSFRNKEIKAA

LRKVL

>ModoOR1.2.66

MPSEARNISYTVTEFILLGFPSRWEIQVLLFCLFSVTYILTILGNMAIVC

AVYWDLRLHTPMYLLLANFSFLEICYVNSDVPNMLANFLSKTKAVSFTRC

LLQLYFFFSLGTTECLFLSIMAYDRFLAICRPLHYPTIMTTKFCSHLVIF

CWVYGFLWFLVPVVLVTQLPFCGPNVIDDFLCDLGPLLDLASACVPIPGT

VLICGTMSSLLIFATFFYIIGSYTLVLRAVLWVPSASGRRKAFSTCSSHL

AVVLLFYGSVMMTYVSPGSGQAEGMQKFTTLFYSVMTPLFNPLIYSLRNK

EMKEALKKVLGRA*

>ModoOR1.2.67

MNIYSIYNISSNMAGFILLGFPCHRENQLLLFSLFFIIYILTLLWNGAII

CAVVWDQKLQTPMYILLANFSFLEIWYVTTIIPNMLVNILSKTKVISFSG

CFIQFYFFFSFGVTECFFLAIMAFDRYLAICRPLHYPTIMTGRLCTNLII

SCWVLGFLWFLVPIIIISQMSFCDPKIIDHFVCDPGPLLALMCTRAPMME

LTCSTLSCLNLFAPFGFIMGSYALVLRAVLRVPSVAGRRKAFSTCGSHLA

VVSLFYGSVMVMYVKPASGNDSGTQKIVTLFYSVVTPFLNPMIYSLRNRE

MKEALRKVLGKRELES*

>ModoOR1.2.68P

ISGRNRVTEFILLSFPCPREIQILLFGVFFLTYILTLVGNGSIIYAVKLD

CRLHTPMYIVLSNFSFL/FLSNTKTITFTACFLQFYFFFSIGTNEAFFLT

LMSVYRYFAICQPLYYPTLMTGRLCIKLMILCWVSAFLCYLVPIYFITQL

PFCGPNIIDHFVYNPGPLLALSCTPAPGIGL

>ModoOR1.2.69P

LQLSYSILSSLIIFITFFLILGSCTLVLKGVVQLPSTVGKLKVFSTCGSH

LVVVSLFYGKLMVMYMSPISGNPAGTQKIVTL

>ModoOR1.2.70

MNISGGNSVTEFILLSFPCPRDIQILLFGIFSLAYILTLMGNGSIICAVR

LDHHLHTPMYFLLANFSFLEICYINTTVPNMLANFLSETKTISFIACFVQ

FYFFFSTGATETFFLSLMAFDRYLAICQPLHYPTIMTGHFCIKLVFLCWV

SGFLYFPVPICFMTQLPFCGPNIIDHFVCDPGPLLALSCAPTPGIELSYS

ILNCLLIFVTFPFILGSYTLVIRAVLNVPSAAGRKKAFSTCGSHLIVVSL

FYGTLMVMYISPNSGNSSGSQKIFTLFYSVVTPLVNPLIYSLRNKEMKTA

LRKVQMSIKTIHRR*

>ModoOR1.2.71

MSGGNRVTEFILLSFPCPREMQILLFGVFSLTYVLTLMGNGSIIWAVRLD

HQLHTPMYILLAHFSFLEICYVNTTVPNMLANFLSETKTISFTACFLQFY

FFFSTGITEPFFLSLMAFDRYLAICQPLHYPTIMTGHFCIKLVLFCWVTG

FLCFPLPIYFMTQLPFCGPNVIDHFICDPGPLLALTCVHSPKLELSFSII

PSLAIFLTFPFILGSYALVFRAVLQVPSAAGRKKAFSTCGSHLTVVSLFF

GTVLVMYITPTSGHITTTQKIVTLFYSVFTPLVNPLIYSLRNKEMKTALK

KVQLAMKILPNKWDSVSKPALKS*

>ModoOR1.2.72

MSGGNRVTEFILLSFPCPREMQILLFGVFSLTYVLTLMGNGSIIWAVRLD

HQLHTPMYILLAHFSFLEICYVNTTVPNMLANFLSETKTISFTACFLQFY

FFFSTGITEPFFLSLMAFDRYLAICQPLHYPTIMTGRFCIKLVLFCWVTG

FLCFPLPIYFMTQLPFCGPNVIDHFICDPGPLLALTCVHSPKLELSFSII

SSLAIFLTFPFILGSYTLVFRAVLRVPSAAGRKKAFSTCGSHLTVVSLFF

GTVLVMYITPTSGHITTTQKIVTLFYSVFTPLVNPLIYSLRNKEMKTALK

KVQLAMKILPNK*

>ModoOR1.2.73

MSVGNRVTDFILLSFRCSREMQILLFGVFSLTYVLTLMGNVSIICAVRLD

HHLHTPMYIILAHFSFLEICYVNTTVPNMLANFLSETKTISFTACFLQFY

FFFSTGTTEPFFLSLMAFDRYLAICQPLHYPTIMTGHFCIKLVLFCWVMG

FLCFPLPIYFMTQLPFCGPNVIDHFICDPGPLLALTCVHSPKLELSFSII

PALVIFLTFPFILGSYALVFRAVLRVPSAAGRKKAFSTCGSHLTVVSLFF

GTILFMYITPTSGHITSTQKIVTLFYSVFTPLVNPLIYSLRNKEMKTALK

KVQLAMKILPNE*

>ModoOR1.2.74

MTFSNTYNGSKSITGFILLGFTYTGEIQKLLFMLLFTIYILTLLGNGSII

CAVYCDERIHTPMYILLANFSFLEICYASTTVPNMLVNFFSETKVISFSG

CFLQFYFFFSLGAVECFFLAIMAFDRYLAICRPLHYHTIMTRHLRTSLVV

SCWIFGFLWFPIPIIFISKLSFCGPKIIDHILCDPSPILVLTCTPAPMME

FACSFLSSMILVIPFFFIIGTYALVLKAVLKIPSGRGQRKAFSTCGSHLV

VVSLFYGSAMVIYMRPRSGQKTDTQKVVTLFYSMVTPFLNPFIYSLRNKE

MKDALKKVLGYENESLCKRYLLRSS*

>ModoOR1.2.75

MDIFNTYNNSNNTAGFILLGFPCSREIQILLFILFFIIYILTLIGNGSII

CAVYWDERLHNPMYILLANFSFLEIWYVTSTVPNMLVNFLSKNKTISFSG

CFLQFYFFFSLGTTECFFLAVMAFDRYLAICRPLHYPTIMTGHLCTCLVV

NCWIFGFLWFLVPIIFISQMSFCGSKIIDHFLCDPGPLLALTCTRAPLIE

FTCSILSSLLLFAPFLYIMGTYALVLRAVLKVPSAAGRRKAFSTCGSHLA

VVSLFYGSVMVMYVKPTSGHEAGTQKTVTLFYSVVTPFLNPLIYSLRNKE

MKDALNKFLGI*

>ModoOR1.2.76

MKISYMENNSNGTAGFILLGFPCSKEVQILLFIFFLFIYILTLIGNGSII

CVVHGNKRLHTPMYILLANFSFLEIWYVTSTVPNMLANFLSKTKTISFSG

CFLQFYFFFSLGGTECFFLAIMAFDRYLAICWPLRYPTIMTGRLCINLVV

TCWVSGFLWFLVPIILISQLSFCGSKIIDHFLCDPGPLLALTCTRAPLIE

FACSIVNSLPLFIPFLFIMGTYALVLKAVLRVPSAAGRRKAFSTCGSHLA

VVSLFYGSVMVMYVKPTSGHEAGTQKIVTLFYSVVTPFLNPLIYSLRNKD

MKDALKKILSI*

>ModoOR1.2.77P

MNNSGVHTVTQFILLGFPGDWEIQILLFFLFLIVYILTMMGNGAIICAVK

WDQRLHTPIYVLLGNFAFLEIWYITSTVPSMLENFLSETKAISFAGCFLQ

FYFFSSLGTTEAYFLCIMAYDRYLAICHPLHYPIKMTLQRCYILISVCWV

LGFLSYFLSTVQLSQLTFCVPNIIDHFICDIDPLMALSCAPALTTEILFY

IISSLIIILTIIYLFGSYILLLRAVLQVPSAAGRRKAFSTCGSHLAVVCL

FFGSVMVMYVSPTSENSGEVQKIITLFYSVVTPF*NPLIYSL*NKEMKTA

LRKVIGISSE

>ModoOR1.2.78

MNKSRTHIVTEFILLGFPGHWEIQILLFVLFFIVYILTMVGNGAIICAVR

WDQRLHTPMYILLGNFAFLEFWYINSTLPSMLENFLSETKTISFAGCFLQ

LYFFSSLGTTEIYFLCIMAYDRYLAICHPLHYPTIMTLKYCNTLMSVCWV

LGFLTYSLSTIQLSQLPFCGPNIIDHIICDMDPLISLSCVPAPTTEILFY

IISSLIIFLPILYILGSYFLLLRTVLQIPSAAGRRKAFSTCGSHLAVVCL

FFGSLMIMYVSPTSENSGDVQKIISLFYLVVTPFLNPLIYSLRNKEMKAA

LRKVIGIASE*

>ModoOR1.2.79

MNKSEAHTVTEFILLGFPGDWEIQILLFSLFLIVYILTMIGNGAIICAVK

WDQRLHTPMYILLGNFAFLEIWYITSTVPSMLENFLSETKTISFAGCFLQ

FYFFTSLGTTEAYFLCIMAYDRYLAICHPLHYPTKMTLQHCYTLISVCWV

LGFLSYFLSTVQISQLTFCGPNIIDHFICDLDPLMALSCAPDPTTEILSY

IISSLIIILTAIYILGSYILLLRAVLQVPSAAGRRKAFSTCGSHLAVVCL

FFGSLMIMYVSPTSDNSGEAQKIITLFYSVVTPFLNPLIYSLRNKEMKTA

LRKVIGISSE*

>ModoOR1.2.80

MQNLFFFLILVVYILTLLGNGAIVCAVKWDKQLHTPMYILLGNFAFLEIW

YVSSTVPNLLANFLSETKTISFSGCFCQFYFFFSLGTTECFFLSVMAYDR

YLAICRPLHYPTIMTGRFCANLISACWVSGFLCYPVPIVLISQLPFCGPN

IIDHFVCDPGPLFALICVPAPVIKLICYTFNSMIIFGPFLSILGSYTLVL

RAVLRVPSGAGRHKAFSTCGSHLVVVSLFYGTLMVMYVSPTSGNPAGMQK

IVTLVYSVVTPLLNPLIYSLRNKDMKNALRKVLGGLKISQST*

>ModoOR1.2.81

MNKSGVHTVTEFILLGFPGDWEIQILLFSLFLIVYILTMMGNGAIICAVK

WDQRLHTPMYILLGNFAFLEIWYITSTVPSMLENFLSETKAISFAGCFLQ

FYFFSSLGTTEAYFLCIMAYDRYLAICHPLHYPTKMTLQHCYTLMSVCWV

LGFLSYFLSTVQLSQLTFCGPNIIDHFICDIDPLMALSCAPDPTTEILFY

IISSLIIFLTIIYLFGSYILLLRAMLRVPSAAGRRKAFSTCGSHLAVVCL

FFGSLMIMYVSPTSDNSGEVQKIITLFYTVVTPFLNPLIYSLRNKEMKAA

LRKVIGISSE*

>ModoOR1.2.82P

MNKSRTHIVTEFILLGFPGHWEIQILLFVLFFIVYILTMVGNGAIICAVR

WDQRLHTPMYILLGNFAFLEFWYILHSSQHVGKLPLRDENYLFCWLLPPV

IFLLLTWYN*NLFPVYHGLRSLSCHLPPTALPNHHDPKVL*HLDVCVLGP

WLPHLFSI*YPALSAAILWSQHH*PNIICDMDPLNALSCVPAPITEILFY

IISSLTIFLPILHILDSYFLLLRTMLQIPSAAGQRKAFSKCGSHLTMVCL

FLGSLMIMYMSPTSENLGDVQKIISLFYLVVTPFLNPLIYSL*NKEMKAA

LRKVMGIASE

>ModoOR1.2.83P

RVFVAMYILVANFSFLEIW*ITSTIPDMLANFLSKTKTISLSGYFLQYYF

SLERTEFF/FFLAVMAFD**LAIY*LMCYPTFTTGCLFINLVVTCWVSGF

**FLILIILMSQLSFCGSKVIDQLLCDPGPLLAFTCTRPLLIEFACFLVN

SLQLFIPFLFIMGTYAMALKVVLRVSSAASPHKTFSTCESHLAIVSLFYG

SVIVMYVKPTSGHEAGTQKIVTLFYSMVLHSLIYSLRNKDMKDVLKKILS

I

>ModoOR1.2.84

MNKSEAHTVTEFVLLGFPGDWEIQILLFSLFLIVYILTMIGNGAIICAVK

WDQRLHTPMYILLGNFAFLEIWYITSTVPSMLENFLSETKTISFAGCFLQ

FYFFTSLGTTEAYFLCIMAYDRYLAICHPLHYPTKMTLQHCYTLISVCWM

LGFLSYFLSTVQISQLTFCGPNIIDHFICDLDPLMALSCAPDPTTEILFY

VISSLIIILTAIYLLGSYILLLRAVLRVPSAAGRRKAFSTCGSHLAVVCL

FFGSLMIMYVSPTSDNSGEAQKIITLFYSVVTPFLNPLIYSLRNKEMKAA

LRKVIGISSE*

>ModoOR1.2.85

MNKSEAHTVTEFVLLGFPGDWEIQILLFSLFLIVYILTMIGNGAIICAVK

WDQRLHTPMYILLGNFAFLEIWYITSTVPSMLENFLSETKAISFTGCFLQ

FYFFTSLAATEVYFLCIMAYDRYLAICHPLHYPIKMTLQRCYTLISVCWV

LGFLSYSLTTVQLSQLTFCGPNIIDHFICDLDPLMALSCAPAPTTEILFY

IINSLIIILTAIYLLGSYILLLRAVLRVPSAAGRRKAFSTCGSHLAVVCL

FFGSLMIMYVSPTSENSGEAQKIITLFYTVVTPFLNPLIYSLRNKEMKAA

LRKLIRISSE*

>ModoOR1.2.86

MNKSGAHTVIEFVLLGFPGDWEIQILLFSLFLIVYILTLIGNGAIICAVK

WDQRLHTPMYILLGNFAFLEIWYITSTVPRMLENFLSETKAISFAGCFLQ

FYFFTSLAANEIYFLCIMAYDRYLAICHPLHYPIKMTLQLCYSLISVCWV

LGFLSYSLTTVQLSQLTFCGPNIIDHFVCDIDPLMALSCAPAPTTENLFY

IISSLILFLSIIYLLGSYILLLRAVLQVPSAAGRRKAFSTCGSHLAVVCL

FFGSLMIMYVSPTSENSGEVQKIITLFYSIVTPFLNPLIYSLRNKEMKAA

LRKFIGISSE*

>ModoOR1.2.87P

GEWAIICAVRWDQRLYTPMYVLLGN*AFVEFWYINATVPSMLENFLSETK

TISFAGCILLFYFSSFGTIESFFLCIMTYGHYLAICHPLHYPTIMTIKSY

SILMSVSWSLG

>ModoOR1.2.88P

LLGNGAIVCAVKWDKQLHTPMYILLGNLAFLEIWYISSTVPNLLANFLSE

TKTISFSGCFCQFYFFFSLGTTECFFLSVMAYDRYLAIC*PLHYPTIMTG

RFCANLISACWVSGFLCYPVPIVLISQLPFCGPNIIDHFVCDPGPLFALI

CVPAPVIKLICYTFNSMIIFGPFLSILGSYTLVLRAVLRVPSGAGRHKAF

STCGSHLVVVSLFYGTLMVMYVSPTSGNPAGMQKIVTLVYSVVTPLLKLL

I

>ModoOR1.2.89

MNKSRTTVTEFVLLGFPGSWETQVVLFSLLLVIYILTLLGNGAIICAVTW

DQRLHTPMYILLGNFSFLEIWYVSSTVPNMLASFLSKTKTISFSGCFLQF

YFFFSLGTTECLFLAIMAYDRYLAICCPLHYPTIMTGRHCATLVSLCWLA

GFLGYPVLIFLISQLPYCSSNIIDHFLCDMDPLMALSCAPAPITELVFYT

QSSLVLFLSITYILTSYALVLRAVFQVPSAAGRRKAFSTCGSHLTVVSLF

YGTVMVMYVSPTYGIPTLMQKIVTLVYSVMTPFVNPLIYSLRNKDMRCAL

KAVLYGMRSSQHS*

>ModoOR1.3.1

MGRNQSSGVTEFILAGFPTLHSMQAELGSIFLLVYLLTLMGNMVIISVVG

VDPRLQTPMYFFLGNLSCLEILITSVIIPKMLTNFFSGRHTISFNACIVQ

FYFYFCLGASEFLLLAIMSVDRYLAVCHPLRYPILMRGAICIRLALICWA

GGLLPVLGPTVVVALLPFCGQDVVIQHFFCDSGPLLRLACTNTKQLEEVD

FALASVVIMISLLLTATSYGHIVLAVFRIPSASGRQKAFSTCTSHLMAVT

LFYGSAIFLYVRPSQSSSVGTNWAVTVVTTFVTPLMNPVIYTLRNERVKE

ALRDMLKKGLAACSGHH*

>ModoOR1.4.1P

LHTPMYIFLKSLSFLNFWCSSMISPQTLVNFLVEKKVISYLGCMAQMFVW

VFFMVAL**QSATS*LLWPMTTMLPYITQYSIMSPTPNLCVSLWLLDPLL

LAFSIPSFTQAVSSA*IFVGHM*PLPFSVMHPQFCQFCEDTFLCEILLFI

FAGVTLLSTTSTILIYYLFIFLTIIRVNSREGRFKALSTCAFLLLAVYLF

FGTSLFVYLRPPSSYSLNQDGLVALIYTVVIPV*NPLIYSLRNKDVKEVL

RKILRRKQ

>ModoOR1.4.2P

LEKTNSTPLAEFILTRIPYPPKLKIFLFLFFLVIYILTQMASPFIFLIVY

SDPQFHAHPMYIFLSILSIIDIG/ILFSSCVAQLYFFHFLKSTQCFLCTL

KACDRYLAICRPLH**L*RWVAPWTEPAIHLPNCGPSQVDYFFCDILAVL

RLTCTHTTINELVAFLVIGVIVGSYFSLILLSYIHIVRAILRICRTDLCI

CHLWSLCDCNYNLLCACCLYLLRPEVNIP

>ModoOR1.4.3

MERMNSTQLTEFILTGIPYPPKLRIFLFLFFLVIYILTQMGNLLIFLTVC

IDSQLHARPMYIFLGVLSIMDMGTSSIIVPRLIMNFTPGIKPIPFGGCVA

QLYFYHFLGSTQCFFYTLMAYDRYLAICRPLHYPVLMTSKVCFLMVAGAW

VAGSIHGAIQATLTFCLPYCGPNQVDYFFCDIPAVLRLACADTTINELVT

FVDIGVVVASCFSLILISYVHIIQAILRIRTADGRRRAFSTCGAHVTVVT

IYYVPAAFIYLRPEANSPLDGAAALFPTTITPFLNPLIYTLRNKEVKLAL

KRMIGDRRAMSKN*

>ModoOR1.4.4P

PTQAKVIPFLFFLVIYTLTQISNLLIFLTVCIDS*LHVQLMYIFFGILSI

IDMRISSIIVPQLIMNFTPGIKPNHLVGM*LSSISIISWAAPSASFTL*W

PMINTWPYANPCTTLS**PQRCAA*W*QEHVAGSIHGAIQAILIFHLPYH

GPNQIDYFFCDIPAVLRLAWDDTTINELLTFVDIGMVVANCFSLILLSYI

HII*AILRILTADGRRHAFSTCGAHVTLVTICYEPAAFNYLRLEANSPLD

GAATLFPTTITLFLNPLIYTSRNQEVKLALKLMVGDQNAMSKN

>ModoOR1.4.5

MERTNSTPVTEFILTGIPYPPKLRTFLFLFFLVIYTLTQMGNLIIFLTVC

IDPQLHARPMYIFLGALSVIDMGISSIIVPRLIMNFTPGIKPISFGGCVA

QLYFYHFLGSTQCFLYTLMAYDRYLAICRPLHYPVLMTSKMCCLMVTGAW

VAGSIHGAIQAILTFRLPYCGPNQVDYFFCDIPAVLRLACADTTVNEQVT

FVDIGVVVATCFSLILLSYIYIIRAILRIRTADGRQRAFSTCGAHVTVVT

IYYVPCAFVYLRPEANSPLDGAAALFPTAITPFLNPLIYTLRNQEVKLAL

KRMVGDRTTMSKN*

>ModoOR1.4.6

MEKTKNNSMESKVTEFILLGLSHPPNLRTFLCLVFLVIYILTQLGNMLIL

LTVWADPQLHARPMYILLGVLSFLDMWLSSVIVPRIILNFTHASKAMSFA

GCVAQLYSFHFLGSTQCFLYTLMAYDRYLAICRPLHYPVLMNGRLCTMLV

AGAWVAGSIHGSIQATLTFRLPYCGPNQVDYFFCDIPAVLRLACADTTIN

ELVTFVDIGVVAASCFLLILLSYANIVYAILQIRTADGRRKAFSTCGSHL

TVVTVYYVPCIFIYLRPGSKSPLDGAVAVFYTVVTPLLNPLIYTLRNKEV

KSALRKLAGSGGTTK*

>ModoOR1.4.7

MEKVKNNSVETTVTDFILLGLSHPPNLRTFLCLVFLVIYTLTQLGNILIL

LTVWVDPQLNARPMYILLGVLSFLDMWLSSVIVPLIILSFTPASKAIPFA

GCVAQLYFFHFLGSTQCFLYTLMAYDRYLAICRPLHYPVLMNGRLCTMLV

AGAWVAGSIHGSIQATLTFRLPYCGPNLVDYFICDIPAVLRLACADTTIN

ELVTFVDIGVVAASCFLLILLSYANIVYAILQIRTADGRRKAFSTCGSHL

TVVTVYYVPCIFIYLRAGSKSPLDGAVAVFYTVVTPLLNPLIYTLRNQEV

KAALKRLTRARGTHNEKK*

>ModoOR1.4.8

MEGFNQTRVTEFVFRGLTDNSMLEMLFLVTFSATYMLTLFGNLLIVVTIM

FTPRLHTPMYFFLSNLSFIDICLSSVTVPKMLEGFLLERKVTSFDACIAQ

LFFLHLFACAEIFLLTIMAYDRYVAICTPLHYSTVMNMKVCAQLVFALWL

GGTIHSLVQTVLTTRLPYCGPNIIDSYFCDVPSVIKLACTDTYLTGVLIV

SNSGTISTVSFLALVTSYTVILVSLRKQSAEGRRKALSTCTAHFTVVVLL

FGPCIFIYTRPDTSFSIDKVVAVFYTVITPLLNPLIYTLRNEEVKNSMKQ

LRQHQVFTKVFT*

>ModoOR1.4.9

MEEAILPNQTSVSYFRLRGLSTNQKVQMTVFAIFLIFYILTLMGNILIVI

TIIYDRRLHTPMYFFLSNLSFIDVCHSTVTVPKMLVDTWSEKKLISFDAC

VVQMFFLHLFACTEIFLLTVMAYDRYVAICNPLHYMTVMNWKVCVLLAAA

LWTGGTIHSVALTSLTIKLPYCGPHEIDNFFCDVPQVIKLACTDTRIIEI

LIVSNSGLISVVCFVVLVVSYAVILVSLRKRISAGRRKALSTCAAHLTVV

TLFLGHCIFIYSRPSTSLPEDKVVSVFFTAVTPLLNPIIYTLRNEDMKNA

LSKLTGRKEGKEEK*

>ModoOR1.5.1

MKNQTTTVAEFILLGFPISREVELLFFILLLPTYLLTLLGNILIICTVVS

YSRLYTPMYFFLCNLSILDILFTSVISPRVLTNLATGNKTISFAGCITQC

YFYFFLGTVEFLLLTSMSYDRYAAICNPLRYSTIMSPSVCIGLVLFSWVG

GFLSVLFPTILISRLPFCGSNIINHFFCDSGPLLALACTDTTAIELMDFL

LSSTVILLSLILIGYSYTYIIMTILRIPSASGRKKAFNTCASHLTVVGIA

GGITVFIYVTPSQKETLEINKIPSVLSSVVAPFLNPFIYTLRNDTVHGIL

REVWVGLRVALVERRRSFLRMLYHKGH*

>ModoOR1.5.2

MGNNSTTVTEIILLGLTDACEFQMPIFVGLLLTYLIILLGNLLIIVVTLM

DHRLYTPMYYFLRNFAVLEIWFTSVIFPKMLNNILTGNKTISLVGCFLQG

FLYFFLGTTEFLLLAVMSFDRYVAICNPLRYATIMSKRVCVQLVLSSWLG

GFLLIVIPSGITFQQPFCGPNVINHFFCDNFPLLELVCADTSLVELMGFV

VANISLLGTLSVTAPCYGHILYTILRIPSAKERQKAFSTCSSHIIVVSLF

YGSCIFMYVRAGKGSEGENLNKVVALLITVVTPMLNPFIYTLRNKQVKLV

IREKVGKWISQA*

>ModoOR1.5.3

MWNSTTITEIILLGLTDACEFQMLIFVGLLLTYLITLLGNLLIIVVTLMD

HRLYTPMYYFLRNFAVLEIWFTSVIFPKMLNNILTGNKTISLVGCFLQFF

FYFFLGSTEFLLLAVMSFDRYVAICNPLRYATIMSKRVCVQLVLSSWLGG

FLLIVIPSGITFQQPFCGPNVINHFFCDNFPLLELVCADTSLVELIGFGV

ANISLLGTLSVTASCYGHILYTILRIPSAKERQKAFSTCSSHIIVVSLFY

GSCIFMYVRAGKGSEGENLNKVVAILNTVVTPMLNPFIYTLRNKQVKLVI

REKVGNWISQT*

>ModoOR1.5.4

MGNNSTTVTEFVLQGLTDACEFQMLIFVGLLLTYLITLLGNLLIIVVTLM

DHRLYTPMYYFLRNFAILEIWFTSAIFPKMLNNILTGNKTISLVGCFLQG

FLYFFLGTTEFLLLAVMSFDRYVAICNPLRYATIMSKRVCVQLVLSSWLG

SFLLIIVPSYITLQQPFCGPNIINHFFCDNFPLLELICADTSILELIGFI

GANVSLLGTLSVTASCYGHILYTILRIPSAKERQKAFSTCSSHIIVVSLF

YGSCIFMYVRTGKGNEGEELNKVVAILNTVVTPMLNPFIYTLRNKQVKLV

IKEKVGKWISQA*

>ModoOR1.5.5

MGNSTTITEIILQGLTDACEFQMLIFVGLLLTYIITLLGNFLIIVVTLMD

HRLHTPMYYFLRNFAVLEIWFTSVIFPKMLNNILTGNKTISLLGCFLQCF

LYFFLGTTEFLLLAVMSFDRYVAICNPLRYATIMSKRVCVQLVLSSWLGG

FLLIIVPSCITFQQPFCGPNVINHFFCDNFPLLELICADTSLVELIGFLG

ANVSLLGTLSVTASCYGHILYTILRIPSAKERQKAFSTCSSHIIVVSLFY

GSCIFMYVRAGKGSEGEDLNKVVAILNTVVTPMLNPFIYTLRNKQVKLVI

SEKVGKWISQV*

>ModoOR1.5.6

MENQTTVTEFILLGFPISREVELLFFLLLLPTYLVTLLGNILIICIVVSH

SCLYTPMYFFLCNLSILDILFTSVISPRVLANLATGYKTISFAGCITQCY

FYFFLGTVEFILLTSMSYDRYAAICNPLRYSTIMSPSMCIGLVLFSWVGG

FLSVLFPTILTSMLPFCGSNIINHFFCDSGPLLALACTDTTFIELMDFLL

SSTVIIFSIILTGYSYVCIIMTILRIPSATGRKKAFNTCASHLTIVVMAG

GITAFIYVTPSQKESLEINKIPSVLSSVVAPFLNPFIYTLRNDTVLGVLR

EVWVGVRAVLVGRGRELLSRVSKKDH*

>ModoOR1.6.1P

SNVIDYFGCDASPILKTSCSDTKFIEKMVIICAVLTLIIT*ILVILSYVY

IIKTILIFSSVQQRKKAFFT*TSNMIVVSMT*GT*IFSYIRSLGKELIA*

NK

>ModoOR1.7.1P

SCVLILQVVFHLPSQDAWLRALHSHDSHICVILAFYTPAFFFMTRHFDQN

VPPYIHIFLVNLYVIVPPMLNPDIFGVGTQQNQERMLKMLLK

>ModoOR1.8.1T

MGGTNMSSFKGFILKGVSDHPQLEMIFFVAILFSYLLTLMGNLTIILISR

LDARLHTPMYFFLSNLSSLDLAFTTSSVPQML

>ModoOR1.9.1P

MAKGNWTTVNEFVLLSFSSLQPELQVLLFLLFLSIELVTLLGNTIIIVVT

SADSALNSPMYFFLRNLSVLEVGFNMVIVPKMLATLLAHDTTISFLSCTT

QMYFFFFFGVSECCLLTTMAYDRYVAIL*PPALP/CDPLRYPVIMSHRAC

VHLAAASWFSGLPVATVQATWLFSFSFCHSNKVNHFFCDSPPVLSLVCGD

TTLFEIYAIEG

>ModoOR1.9.2

MDWENWITVDEFFLVSFPALHPELQILLFLLFLFIYFVTFMGNVLIILVT

TADSALHSPMYFFLRNLFFLEISFNMVIVPKMLSTLLPKNTTISFVGFAT

QMYFFFFFGAAECCLLATMAYDHYVAICNPLRYPVIMDQQACVQLAAASW

FSGFPVATVQTSWIFSFPFCGPNKVNHFFCDSPPVIALVCADTSLFELEA

LTATILFILFPFLLILGSYVRILSTIFRMPSAEGKQKAFSTCSSNLVVVS

LFYSTAILTYFRPRSNTSPETKKLLSLFYTVVTPMLNPIIYSLRNSEVKA

ALHRTLQKALGAQKL*

>ModoOR1.9.3

MNQTNHSRVTEFLLLGLSDDPHMQLLLFILVLGVYLVTVLGNLLLIFLVL

TDTQLHTPMYFFLCNLSTADLFFSTNIVPQALVHMLSKRKVISFIGCAAQ

LLLFLIFGCTQSALLAVMSYDRYLAICDPMHYSLIMTGRLCCLLSLGCWT

SGFLISIVDTTFTLHLPYQGDNKISHFFCEAPAILAIASGDTHSSKMAIF

LMGMMILLAPVSLILVSYGSIIVTVIRMKTASGRLKAFSTCGSHLLVVIL

FYVTGIMTYMTPKSSKEQGKLVSVFYAVINPMLNPLIYSLRNKDVKRAFR

NATKRGKVCKL*

>ModoOR1.9.4

MAAENWTTVTEFVLLSFSTLQPKVQALLFLLFLTIELITLMGNALIILVT

TVDSGLHSPMYFFLRNLSFLEIGFNLVIVPKMLGTLLAHDTNISFLNCAI

QMYFFFFFGASECFLLATMAYDRYVAICDPLRYPVIMSHKACVQLAAASW

LSGLPVATVQTTWLFSFPFCGSNQVNHFFCDSPPVLKLVCADTALFEIYA

IVGTILVVMAPCLLILGSYVCIGSTIIRMPSVEGKRKAFSTCSSHLLVVS

LFYGSASLTYFRPKSNNSPESKKLLSLSYTVVTPMLNPIIYSLRNKEVKT

TLYHILHRVLGCLKI*

>ModoOR1.10.1

MERTNRSSEGAPFILLGLSTSPGQLRPLFALFLVLYILGVMGNGLIVTAI

RASPALHAPMYFLLAHLSFADLCFTSVTVPKMLANLLAQDRSISRAGCLT

QMYFFFALGVTDSCLLAAMAYDRYVAIRHPLHYAARMSRAVCTGLVGAAW

AVSHSHSLLHILLMARLSFCASRSVPHFFCDHQPLLRLACSDTRHIQLLI

FTEGAAVVVTPFLLILASYGAIAVAVLRLPSASGRLRAVSTCGSHLAVVG

LFYGTVIAVYFQPTARYEAERGRVATVMYTVVTPMLNPVIYSLRNRDVQG

ALRALFTGRRISTEPI*

>ModoOR1.10.2

MTLKNITWIDGAPDEFILLGITDRWDLRVALFLVFLPIYLLSLLGNMGMV

LLINVDTRLHTPMYFFLANLSLLDACYSSAIGPKMLIDLLLSHATIPYAA

CAIQMFVFAGLADAECCLLAAMAYDRYVAIGNPLLYTTAMSRRLCLLLLA

ASGLGGALSAFVHTTFTFRLHFCHSREVNSFFCDIPPLLAISCDDTSLNE

LLLFAVCGFIQTATVLAIAVSYAFILVAVIRMNSAEGRRRAASTCGSHLT

AVAMLYGTLIFMYLRPSSSYALDTDKMASVFYTLVIPALNPLIYSLRNKE

VKEALQRTWNRFCFPHQRHQ*

>ModoOR1.10.3

MGRGNTTSISEFLLLGLSEQPDYQLILFGLFLIMYLVTMLGNLFIILAIA

SNSHLHSPMYFFLANLSFIDNCFTSTTVPKMLMNIWTHHQSISYAGCLTQ

MYFFMTFALLDDFLLAAMAYDRYVAICLPLHYTTVMSPTRCVLLVTISWL

CSHLIALSLTLFMTQFSFCGSHVIPHFFCDLLPLLKLSCSDTDTFQVVMF

IDAALAGLVPFTCILFSYVHIISTIFRVPSADGKRKVFSTCGSHLSVVIL

FYGTVILVYLRPSSSYSAETGTIASVMYTVVTPMLNPFIYSLRNRDIKGT

LRRLLSKGMISFW*

>ModoOR1.10.4

MQKGNTTGISEFLLLGLSEQPNHQLILFGIFLTMYLVTILGNLLIILAIA

SNSHLHSPMYFFLANLSLVDTCFTSTTVPKMLLNIWTHRQNISYAGCLTQ

MYFFMAFALLDDFLLAAMAYDRYVAICLPLHYTTVMSPTRCVLLVTISWL

CSHLIALSLTLFMAQFSFCGSYVIPHFFCDLLPLLKLSCSDTSIFQVVMF

IDASLAGFIPFACILFSYVHIILTIFRVPSDDGKHKVFSTCGSHLSMVIL

FYGTLILVYLQPSSSYSAETGIIASVMYTVVTPMMNPFIYSLRNKDMKRA

LRKLLSKGMTPSW*

>ModoOR1.10.5P

FFCDIQSLLTLSCSDTSINEVTAFTEGSLVIMSPFLFIVISYVRITHIVL

RVPLGRGRYKAFSNCSSHIAVVVLFYGTIISVYIHPSSTYSVTKDRVVTI

MYTVVTPVLKPFIYSLRNKDMQGALRKVMRRAE*P

>ModoOR1.10.6P

SSTSGFILLGLSSNPQLQKPLFITFLSMYIVILLGNMLIILVIRSDSRLH

SPMYFFLSILSFIDICFTSVIVPKMLVNFLSETKAISYIGCLVQMYFFMA

FGNTDSYLLASMAIDRLVAICNPLQYAMVMSDGRCIILAVVSCVISHLHA

LLRVLLMSRLSFCASHVIKHFFCDTQPVLKLSCSDTSSSQVVVMTETLAV

IVTPFLCIIFSYLRIIVAILKIPSASGKWKTFSTCGSHLTIVVLFYGSII

YVYFRPLSSYSVVKDRVATVMYTVVTPMLNPFIYSLRNKDMKQGLGKLRD

RIKF

>ModoOR1.10.7

MATKNWTEVTEFLLLGLSDRPEMQPVIFGVILSMYLVAVTGNTMLIVVAS

TDSKLQTPMYFLLRQLSFIDVLLTTIVVPQMLVHTGTGFKTIPFENCIVQ

LFFFMAIGSMEGHLLAAMAYDRYVAICKPLQYYAIVTRSLCLRITLSSWV

VVSLNSLLYSVLAARLTFCGNRVTHFFCDITPLLKLSCTRPLLNEMLIFT

EGAAIILSPFFFILASYVRIGVAIGRLHSAAALKKALSTCSSHILVVLLL

YGSVIRMYQRPSSSYDLDQDRQVAVFYTVVTPMLNPLIYSLRNQEVKDAL

WRLLNKIQNVYASGDFQTDHPSRRPQHHIS*

>ModoOR1.10.8

MDTRNWTEVTEFLLLGLSDRPEMQPVIFGVILSMYLVAVTGNIMLIVVAS

TDSKLQTPMYFLLRQLSFIDVLLTTVVVPQMLVHTVTGFKTIPFENCIVQ

LFFFMAIGSMEGHLLAAMAYDRYVAICKPLQYYAIVTRSLCLRITLSSWV

VVSLNSLLYSVLAARLTFCGNRVTHFFCDITPLLKLSCTRPLLNEMLIFT

EGAAIVISPFFFILASYVRIGVAIGRLHSAAALKKALSTCSSHILVVLLL

YGSVIRMYLRPSSSYDLDQDRQVAVFYTVVTPMLNPLIYSLRNQEVKNAL

WRLLKKIQNVYASGDFQPNSPSRGTQDHIS*

>ModoOR1.10.9

MQEMERTNWTSVSYFILLGISTQSEEQIPLFVLFLFMYIINVSGNLVIIL

LTISTTRLHTPMYFLLSSLSLADIGFTSTIVPNMLFNIFSDMKTISYIGC

LTQVYFFISFAAMENFLLAVMAYDRYVAICHPFHYPVVLTRRLCAQMVVS

CFMLSHVHSLLHTLLMTRLSFCSDNKIHHFFCDLYPLMKISCSSAYINTL

VIQTEGVVIINGALTFIIISYAYIISAILKVPSAIGKRKAFSTCGSHLTV

VAIFYGTLTWVYFRPLSSYSVAMGRIMTVMYTVLTPMLNPFIYSLRNGDM

KAAFRKCLTRIWYSSLK*

>ModoOR1.10.10

MERENKTSISEFLLLGLPIRPQQQVLFYILFLSMYLTTVLGNLLIIFLIR

LDSHLHTPMYFFLSHLAFTDVCFSSVTTPKMLMDMQTGSQTIPYGGCISQ

MYFFILFTDLDSFLLTAMAYDRYIAICYPLHYTTIMNQSLCSLLVVGSWI

LSCASSFTHTLLLVQLSFCAENVVSHFFCDLDALLKLSCSDTSLNKLVMF

TVGTTVIMLPLICVLVSYIHIVATILRMPSSKGTCKALSTCGSHLSVVSL

YYGTIFGQYLFPSSRNSNIKDIVSAMMYTVVTPMLNPFIYCLRNRDMKDA

LRRILNKTIFSQRR*

>ModoOR1.10.11

MGTDNNKTSVSEFLLLGLPIRPQQQVLFYILFLSMYLTTVLGNLLIIFLI

RLDSHLHTPMYFFLSHLAFTDVCFSSVTTPKMLMDMQTGSQTIPYGGCIS

QMYFFILFTDLDSFLLTAMAYDRYIAICHPLHYTTIMSQSLCSLLVVGSW

ILSCASSLTHTLLLARLSFCEEHTIAHYFCDLAALLKLSCSDTFLNELVI

FTVGVAIITLPLICILVSYIHIIATILKIPSTKGICKALSTCGSHLSVVS

LYYGTIIGQYFFPSSSNSNFKDIISAMMYTVVTPMLNPFIYSLRNRDIHG

ALKKVFHKRMFFFSSQSDP*

>ModoOR1.10.12

MGQENRTSISEFILLGLSEQPDQQRLLFGLFLTMYLITVVGNLLIILAIG

SDSHLQSPMYFFLANLSFADICFTSASIPKMLVNIETQHQTISYVGCITQ

LYFLLALGGLDNLLLAAMAYDRYVAICRPLHYATTMSPQNCVLIMSMCWA

LTNIPALTHSILLAHLFFCTQHAIPHFYCDISALLKLACSDTHLNELMVI

ILGAIFLTVPLALIILSYACITSAVLGFSSPEGRWKAFSTCGSHLTVVLL

FYGSLMGVYLFPSSSDSVRRDSATSVLYIVVTPMLNPFIYSLRNRDMKGA

LRRLLGNGKTLSSS*

>ModoOR1.10.13

MDRGNQMRVSEFLLLGLSHPLEQQLILSGLFLIIYLVTAVGNLLIMLAIC

SDSHLHTPMYFFLANFSFTDVCVSSTTVLKMFNIQTHSQTICYGGCLTQI

FFFLSFIGLDVFLLGIMAYDQLVAICHPLHYTILMSPQHCYLLVAVSLVM

AHLFSLTHTNLLAQLPFCAANTIPHFFCELLPLMKLSCSEPYTNQHVLIY

WGGALTILIPLMILISYVPIMNPILCIPSVKGKCKAFSTYGSHLSAVCLF

YVAAIGVYFIPFEADSAGKDRLAAVMYAVVTPMLNPFIYTLRNTDMKAAL

GRILNRRSLLFL*

>ModoOR1.10.14

MEVKNQTTVSEFLLLGLSEQPEQQLILFGLFLVMYLVTVVGNLFIILAIG

SDLHLHSPMYFFVANLSFADMSFISTTVPKMLANIWTQSQTISFEGCLTQ

MHFFMTFGALDDFLLGVMAYDRYVAICQPLHYAIIMSSGICILLLAVCWF

LTNSAAFLHTFLMASLTFCAENRIPHFFCDLTPLLSLSCSDTSINQLMLF

IVGSIVLTAPLTLIVISYVHIISAILRIPSASGRWKAFSTCGSHLTAVSL

FYGAAIGVYLCPPSTHSAGKDRIAAVLYTVVTPMLNPFIYSLRNRDMKKA

LVKLLKRKALSS*

>ModoOR1.10.15P

FGLFLGMYLVTLVGNLTTILAIIFDSHLYIPLYFFVANLSFVDISFPSTT

VPMML/KNVYYGSCLTYHYFFGLFADLDNFLLAIMAYGCYIAISCPL*YA

TTMCLQHCIVMVGASWVVIVIHALLHHPPTGLSGLLWSSCYPLLLL*PDS

TLKTSLFRHPYK*TNAISNVLYFTCQTFLCILVSYIYILLTILKMHSPQG

KYKAFSNCPSHLSVVSLFYGSGIGVYLCPSSITSNGKSCAITCMVVTLML

NPFIYSLRNKDMKRAIEKLPNKGT

>ModoOR1.10.16

MGCASNASHTPIFLLVGLWRGGPPNHLLFPLFLIAYVAAVVGNLMLVLLI

SRDSQLGTPMYYLLRGLSVVDAGQATVTLPQLLVHLVSLQPAIPAAHCLA

QFFFFYLFAVTDTLVVAVMALDRYVAICDPLHYSALMNRHVCDCFMASCL

FLSLLHSLLHLGLLLPLQWNGNSGDAVYIPHFFCDHWPLVRASCSNIQLN

VLAIFLEGSLFVAGPCALILFSYAHIAVAILRLPSAAGRWKAVSTCSSHL

TMVSFLYGTVILVYFQPPSHNSPEQDMAAAVMYTAITPVANPFVYSFRNK

NVKNALHRLLSPNRVASQLEWS*

>ModoOR1.10.17

MACASNASHTPIFLLVGLWKGGPPNSLLFLLFLTAYMGAMVGNLTLVLLI

SWDSRLSTPMYYLLRGLSVIDTGLATVTLPQVLAHLASAQPAIPAIRCLL

QFFFFYVFGVTDTLVVAVMAVDRYVAICNPLHYPAVMNQQVCGRLLASCW

TVSFIHSLLHVGLLFPLQWTGDNEGVVYLPHFFCDHRPLLKASCSDTKPN

ELAIFLEGGLLMMGPCILILLSYARIAITISRLPSAAGRKKAFSTCGSHL

TMVTFLYGTIIWVYFQPPSQNSQQQDMAAAVMYTAITPLANPFVYSLRNK

DVKGAFQRLLVPNRVTS*

>ModoOR1.10.18

MSLSLMETYNYTSVTEFVLLGLTSQLDIQPVIFGVIFAMYLITIVGNSMI

ITVTWIDPKLKTPMYFLLSQLSIIDICFTTITVPQLLIHTFSRSKTISFT

QCMTQVFFFVAVGNMEGYLLATMAYDRYVAICNPLRYGAIVTQKFCVSIV

LVSWLLMFLNSLLHTILVSRLNFCSNHILHFFCDLPPLMQLSCSRPFINE

IIILTEGVAATLSPFVFILASYVCIAVTVLGLRSTAGLRKAVSTCGSHIV

VVTLFYGTIIRLYFQPVSSYTLDRDRQVAVFYTVVTPMLNPIIYSLRNQE

VKGALQRTLRKIF*

>ModoOR1.11.1

MSDINKTWVTEFFFLGLSDDLQIQLFLFILFLIVYLVTLLGNLLLISLVL

IDSQLHTSMYFFLCNLSLADLGFSTCIVPQVLAHMLSRRKVISFIGCAVQ

LLFCVILGGTECALLAVMSYDRYLAICDPMHYPIIMTSRLCGHLVLGCWI

SGILVALVHTTFTLRLPYQGDNRIAHFFCESPALLALASADTHSSKMAIF

LMGSLFLLAPVSLILASYGSIIVTVIKMKTTSGRRKAFSTCGSHLLVVII

FYGSAIINYMRLKPSKEEAKVASVFYAVINPMLNPLIYSLRNKDVTKALR

NIAKKRKCFIS*

>ModoOR1.11.2

MGQTNQTWVTEFFLLGLSDDPQTQLLLFALFLAVYLCTILGNLLLTSLVL

LDSQLHTPMYFFLCNLSLADLCFSTTIVPQTLIHMLSRRKFISFTGCAVQ

LLLILFFGCTQCSLLAVMSYDRYLAICNPMHYPLIMTWRVCVLMALACWS

SGVVVSMVDTTFTLRLPYQGENRIAHFICEPPALLVVASADTHNSEMAVF

LMGVVILLAPVSLIMISYVCIIASVVRMKTASGRLKAFSTCGSHLIVVII

FYGSAIINYMTPKSAKELGKMVSVFYAVINPMLNPLIYSLRNKDVKRAFK

NVANRRLFCTV*

>ModoOR1.11.3P

MSQTNQNCETEFFLLRVSNDPQIQLLFFTLFLAICLGTILRTWSSHPWFC

LTHDSTHPYIFSYVIISCLSLLLNYYTTIPQTLIHILPRRKLISFRG*TV

QLLSSLFFIST*SLLLDIMSYDSYLKIYDQMHYSFILTWRVCGFLALEY*

SSGFVLSFVDSTFILLLPYKGDGRIAHFIYALLALLVVASADT/THSSEM

AFFLMGVMTLLVLLSVISVSYMCVIVYMIRMKTALGKLKAFSTCVSHLIM

VIFYGSAIINVMTPRSAKELGNIVYVFFVVISPIFHPLIYNLRNKDVVTS

Y

>ModoOR1.11.4P

MNQTNQTWVTEFFLLGLSDDPQTQVLLFALFLVVYLSTILGNLLLTSLVL

LDSQLHTPMYFFLCNLSLTDLCFSTTTVPQSLIHMLSRRKLISFTGCAGQ

VLFFLFFGSTQCVLLGVMSYDRYLAICDPNALSPHHDMEGMWPLRP*GAG

PLALLHPWWTAFSY*VYLTKETIRLLISSVRLQPFWFWHLQILTAQIWSF

SSMGSVIIVTPMFLILISYVCIIISVIRMKTASGRLKAFSTCGSHLIVVI

IFYGSAIINYMTPKSSKELGKMVSVFYAVINPMLNPLIYSLRNKDVKRAF

RNVANSRPFCRI

>ModoOR1.11.5P

MNQSNQN*VTEFFLLGLSDDPQTQRLLFSLFLAVYLSTILGNLFLMILVL

LDSQLFIPMYFFLGNLSLADLCLLITIVSQS/LIQMLTRKKLISFTGYAG

QVLLFLFFGSLQCVLLDVMSYNRYLAISDLLHYLLIMIWRVCGLLALCCW

SSGLVTSLV

>ModoOR1.11.6P

HNPIHTCIFFLCNLFLADFCFSTTIVPQTLIHMLSRRKFISFRGCAVQLL

SILSFGSTQRSLLGMMSYDRYLAICDPMHYPLIMTWKVCGLLAFGCWSSG

LVASVVDTEYTLHLPYQGDNRISHYFCE

>ModoOR1.11.7

MNQTWITEFLLLGLSDDSQNQLLLFALFLVVYLVTLLGNLLLITLVLTDS

QLHTPMYFFLCNLSTADLFFSTSIVPQALFNLVSRRKVISFIGCAAQLLL

FLLFGCTECALLAVMSYDRYLAICDPMHYPLIMTWRVCGYMALGCWASGI

LVSLVDTTFTLRLPYQGDNRIAHFFCEPPAILALASADIHSAETAIFLMG

VVILLAPLSLILVSYGSILMTVVRMKTTSGRLKAFSTCGSHLLVVILFYG

SGIISYMTPKSAKLLGKLVSMFYSVITPMLNPLIYSLKNKDVKKALRDVA

IKRLLLKL*

>ModoOR1.11.8

MDWGNWSIVNEFVLVSFTALHPELQILLFLLFLFIYFVTFMGNVLIILVT

TADSALHSPMYFFLRNLSFLEISFNMVIVPKMLSTLLTKNTTISFVGCAT

QMYFFFFFGAAECCLLATMAYDRYVAICNPLRYPVIMDQQACVQLAAASW

FSGFPVATVQTSWIFSFPFCGPNKVNHFFCDSPPVIALVCADTSLFELEA

LTATILFILFPFLLILGSYVRILSTIFRMPSAEGKQKAFSTCSSHLVVVS

LFYSTAILTYFRHRSNTSPETKKLLSLFYTVVTPMLNPIIYSLRNSEVKA

ALHRTLRKALGAQKL*

>ModoOR1.11.9

MAKGNWTTVNEFVLLSFSSLQPELQVLLFLLFLSIELVTLLGNTIIIVVT

SADSALNSPMYFFLRNLSVLEVGFNMVIVPKMLATLLAHDTTISFLSCAT

QMYFFFFFGVSECCLLTTMAYDRYVAICDPLRYPVIMSHRACVHLAAASW

FSGLPVATVQATWLFSFSFCHSNKVNHFFCDSPPVLSLVCGDTTLFEIYA

IIGTIVFVMIPCLFILSSYARIVAAILRMPSAEGKRKAFSTCSSHLVVVS

LFYGSLSLLYFRPKSNNSPESKKVLSLSYTVVTPMLNPIIYSLRNNEVKA

ALSRTFLRAISFCKL*

>ModoOR1.11.10P

HTLIFFPM*LSLAYLCFSTTTPQTLIHILPRRKLISFRG*TVQLLSSMFF

IST*SLLLDIMSYDCYLKIYDQMHYPFILTWRVCGFLALEY*SSGFVLSF

VDSTFILLLPYKGDGRIAHFIYALLALLVVASADT/THSSEMAFFLMGVM

TILVLLSVINVSYMCVIVYVIRMKTASGKLKAFSTCVSHLIMVIFYGSAI

INVMTPRSAKELGNIVYVFFVVISPIFHPLIYNLRNKDVVTSY

>ModoOR1.11.11

MNQTNQTWVTEFFLLGLSDDPQTQVLLFALFLVVYLSTMLGNLLLTSLVL

LDSQLHTPMYFFLCNLSLTDLCFSTTTVPQSLIHMLSRRKLISFTGCAGQ

VLFFLFFGSTQCVLLGVMSYDRYLAICDPMHYPLIMTWKVCGLLALGCWS

AGFVASMVDSIFILSLPYQGDNQIAHFFCEAPALLVLASADTHSTNMVIF

LMGSVIIVTPMFLILISYVCIIISVIRMKTASGRLKAFSTCGSHLTVVII

FYGSAIIKYMTPKSSKDIGKVISVFYAVISPMLNPLIYSLRNNDVKRAFR

NVANSRPFCRI*

>ModoOR1.11.12

MNQTLVTEFFLLGLSDDPQTQLLLFALFLVVYLGTILGNLLLTSLVLLDS

QLHTPMYFFLCNLSLADFCFSTTIVPQTLIHMLSRRKFISFRGCAVQLLS

VLFFGSTQCSLLGVMSYDRYLAICDPMHYPLIMTQKVCGLLAFGCWSSGL

VASVVDTAYTLHLPYQGDNRISHYFCEPPALLVVASADTYSTEIAIFLMG

VVILLFPVSMIMVSYTCIIVSVVRMKTASGRLKAFSTCGSHLIVVIIFYG

SAIINYMTPKSAKELGKITSVFYAVINPMLNPLIYSLRNKDVKRAFRNVA

NRRPVFRS*

>ModoOR1.11.13

MSQMNQTWITEFLLLGLSDDSQTQLLLFVLFLVIYLVTVLGNLLLITLVL

TDSQLHTPMYFFLCNLSTADLFFSTTIVPQALFNLSRRKVISFIGCAAQI

LLFLLFGCTECALLAVMSYDRYLAICDPMHYPLIMTWRVCGYMALGCWTT

GILVSLVDTTFTLRLPYQGDNRIAHFFCEPPAMLALASADIHSAETALFL

MGVVILLAPLSLILVSYGSILMTVIRMKTTSGRLKAFSTCGSHLLVVILF

YGSGIISYMTPKSAKLLGKLVSMFYSVITPMLNPLIYSLKNKDVKKALRV

VSIKRLFVKL*

>ModoOR1.11.14

MSDTNKTWVTEFFFLGLSDDLQIQQLLFILFLIVYLVTVLGNLLLISLVL

IDSQLHTSMYFFLCNLSLADLCFSTCIVPQVLAHMLSKRKVISFTGCAAQ

LLFSIFLGGTECALLAVMSYDRYLAICDPMHYPIIMTSRLCGHLALGCWV

SGILVALVDTTFTLRLPYQGDNRIAHFFCESPALLALASADTHSSKMAIF

LMGSVILLAPVSLILASYGSIIVTVIKTQTTSGRRKAFSTCGSHLLVVIV

FYGSAIINYVTLKPSKEQAKVASVFYAVINPMLNPLIYSLRNKDVNKALR

KVVKKRKLFIS*

>ModoOR1.11.15P

MSQTNKTWVTEFLLLGLTDDPQTQLLLFVLFLVVYLITVLGNLLLISLVL

YDSQLHIPMYFFLCNLSTADLSFSSTIVPQALVHMISRRKFISFMGCVAQ

LLFILLFGSTECALLAVMSY

>ModoOR1.11.16P

VLKFTVDTTFTLQPPYQGENRIPHFFCEPPALLALASADTHSSQMAIFLM

GVVILLAPVSLILVSYGCIIITVVRMKTTSGRLKAFSTCGSHLLVVVIFY

GSAIISYMTPRTTKEQGKLVSVFYAVITPMLNPLI

>ModoOR1.11.17

MSDINKTLVTEFFFLGLSDDLQIQLLLFILFLIVYLVTVLGNLLLISLVL

IDSQLHTSMYFFLCNLSLADLCFSTCIVPQVLAHMLYRRKVISFTGCVAQ

LLFSLFLGCTECALLAVMSYDRYLAICDPMHYPIIMTSRLCGHLALGCWV

SGILVALVDTTFTLRLPYQGDNRIAHFFCESPALLALASADTHSSKMAIF

LMGVVILLAPVSLILASYGSIIVTVIRMKTTSGRRKAFSTCGSHLLVVII

FYGSAIINYMTLKPSKEQAKVVSVFYTVINPMLNPLIYSLRNKDVNKALR

NIAKKRKCFIS*

>ModoOR1.11.18

MSQTNKTWVTEFLLLGLTDDSQTQLLLFVLFLVVYLITVLGNLLLISLVL

YDSQLHTPMYFFLCNLSTADFCFSTTIVPQALVHMISRRKFISFMGCAAQ

LLFILLFGCTECALLAVMSYDRYLAICDPMHYPLIMTWKVCIKLALGCWV

SGILVSMVDTTFTLQPPYQGENRIPHFFCEPPALLALASADTHSSQMAIF

LMGVVILLAPVSLILVSYGCIIITVVRMKTTSGRLKAFSTCGSHLLVVVI

FYGSAIISYMTPRTAKEQGKLVSVFYAVITPMLNPLIYSLKNKDVKKAFR

NAVIQGPLQRF*

>ModoOR1.11.19

MNLSNQTWVTEFFFLGFSDDPQTQLLLFISFLIIYFVTALGNLLLIILVL

ADSKLHTPMYFFLCNLSVADLCFSTSIVPQALVHMLTRRKIISFMGCATQ

LFLFLIFGCTGCALLSVMSYDRYLAICDPMHYPLIMTKKVCVQLALGCWV

SGVVVSIVDTTFTLQVPYRGENKISHFFCEAPALLALGSADTYRAEKVIF

IMGVVILLAPVSLILVSYGSIIVTVVRIKTTSGRLKAFSTCGSHLLVVTI

FYVSAIMTYMTPKSSKEQGKLVSVFYAVIAPMLNPLIYSLRNKEVKRALR

NTITKGQICKL*

>ModoOR1.11.20

MNQTNQIWVTEFLLLGLSDDPQTQLLLFVLFMGVYLVTMLGNLLLISLVL

IDSQLHTPMYFFLCNLSFADLFFSTNIVPQALVHLLSKRKVISFIGCAAQ

LLLFLIFGCTQCALLSVMSYDRYLAICDPMHYPLIMTWKVCSQFSLGCWV

SGILVSLVDTTFTLQLPYQGDNKIAHFFCEAPALLVVASADIHKAETAIF

FMRVMILLAPVSLILVSYGAIIVTVVKMKTTSGRLKAFSTCGTHLLVVIL

FYGSAIITYMTPKSSKKQGKLVSVFYAVINPMLNPLIYSLRNKDVKRAFR

NVINRGKLFRFILSL*

>ModoOR1.11.21

MNQTTHFRVTEFLLLGLSDDPKIQLLLFILVLGVYLVTVLGNLLLIFLVL

TDTQLHTPMYFFLCNLSTADLFFSTNIVPQALVHMLSKRKVISFIGCAAQ

LLLFLIFGCTQCALLAVMSYDRYLAICDPMHYSLIMTGRLCCLLSLGCWI

SGFLVSIVDTTFTLHLPYQGDNKISHFFCEAPAILAIASGDTHSSKMAIF

LMGVVILLAPVSLILVSYGSIIVTVIRMKTASGRLKAFSTCGSHLLVVIL

FYGTGIMTYMTPKSSKEQGKLVSVFYAVINPMLNPLIYSLRNKDVKRAFR

NATNRGKICKL*

>ModoOR1.11.22

MNLNNQTWVTEFLLLGLSDDPQTQLLLIILFLGVYLGTVLGSLLLIYLVL

TDSHLHTPMYFFLFHLSLADLGVSTTIVPQALFHMLTRKKAISFMGCAAQ

LFFTLHFGSTQCSLLAVMSYDRYLAICDPMHYSLIMTGRLCGQLALVCWV

CGIVVSSVDTVFTLSLPYQGDNRIAHFFCDAPVILTLASGDTHKAEVAIF

FMGVVILLTPVSLILVSYGFIIVTVIRMKTTSGRLKAFSTCGSHLLVVIL

FYGTLISSFMTPKTLREQMKQVAVFFVVINPMLNPLIYSLRNKEVKRAFK

TAVNRK*

>ModoOR1.11.23

MNHNNQTWVTEFLLLGLSEDPQTQLFLIILFLGVYLGTVFGSFLLIYLVL

TDSHLHTPMYFFLFHLSLADLGVSTTIVPQAVIHMLTRRNAISFFGCAVQ

IFLSIVSGATQCLLLAIMSYDRYLAVCDPMHDSLIMTGRVCGQLALVCWV

CGTVASSVDTIFILCLPYQGNNRIEHFFCDAPVLLTLASGDTHKAELAIF

FMGVVILLAPVSLILVSYGLILVTVIRMKTSSGRLKAFSTCGSHLLVVIL

FYGTLIIGYMIPKSSREQIKQVAVFYGVINPMLNPLIYSLRNEEVKRAFR

NAVNKIFLFHSRSLTTFGHISYPQSLSFIPLAKNDHTTMTGIRECMKILS

FYFTYTYIYIYVCIKICGSKHITVYNYND*

>ModoOR1.11.24

MNQNNRTWVTEFFLLGLSGDPQTQLLLTVLFLGVYLGTVLGSLLLIYLVL

TDSQLHTPMYFFLCNLSLADLGLSTVIVPQAIIHMLTRRNAISFMGCAAQ

IFFSIISGATQCSLLAVMSYDRYVAICDPMHYSLIMTGKVCGQLALVCWV

SGIVASSVDTIFTLCLPYQGDNRIAHFFCEAPVLLTLASGDTHKAEMAIF

FVGVWILIAPMSLILVSYGLIIVTVIRMNTMSKRLKAFSTCGSHLLVVIL

FYGTLIIGYMTPKSSREQFKQVAVFYGVINPMLNPLIYSLRNKEVKRAFK

NAIKRK*

>ModoOR1.11.25

MNQTNQTWVTEFLLLGLSNDPQTQVLLFVLFLGVYLVTVLGSLLLIYLVL

TDSQLHIPMYFFLCNLSVADLCFSTNIVPQAMNHMLTRKKVISFMGCVAQ

LFLFLIFGATQCALLAVMSYDRYLAICDPMHYPIIMTRRMCSLLALGCWI

SGILISLVDTTFTLHLPFKGDNKIAHFFCEAPALLDVASADTHNSQMVIF

LMGVVILLAPVSLILVSYGSIIVTVVRMKTTSGRLKAFSTCGSHLLVVIL

FYGSAIISYMTPKSSKEQAKLVSVFYAVINPMLNPLIYSLRNKDVKRAFR

NATNRGKPCRP*

>ModoOR1.11.26P

MNQNNQTWVIEFLLLGLSDDPQIQLLFIVLFLGVFLGTVLGSLLIIYLVL

TDSQFHTPMYFFLCNLSLADIGVTITTVPQALVQMLSRKKTISFLGCGAQ

IFFSTTFGATQCSLLAIMSYD*YLAICDPMHYSLIMTGKVCGQLALVCWV

SGIVASYVNATFTLCLPYQGDNRIAHFICEAPVPLTLASGNTHKAEMALF

FIGVCILLAPMSLILVSYGLIIVTVIRMKTMSKRLKAFSTCGSHLLVDIH

FYGMLIIGYMMPKSSRKQFKWVAIFSGVINPMLNPLIYSLRNKEVKRAFR

NTVSRK

>ModoOR1.11.27

MNQTNQTWVTEFLLLGLSNDPQTQVLLFVLFLGVYLVTVLGSLLLIYLVL

TDSQLHIPMYFFLCNLSVADLCFSTNIVLQAMNHMLTRKKVISFMGCVAQ

LFLYLIFGATQCALLAVMSYDRYLAICDPMHYPIIMTRRMCSLLALGCWI

SGILISLVDTTFTIHLPFKGDNRIANFFCEAPALLDVASADTHNSQMVIF

LMGVVILLAPVSLILVSYGSIIVTVVRMKTTSGRLKAFSTCGSHLLVVIL

FYGSAIISYMTPKSSKEQAKLVSVFYAVINPMLNPLIYSLRNKDVKRAFR

NATNRGKPCRP*

>ModoOR1.11.28P

VMTTGLCVHLLTLSYASNFTDSRIKVYFSHDIFCVSNVMNNFFCYISPIL

KLVYMDFSIVKLVNSDLAFIIFIL*YSFINIYVSTHSMLSCGYIS*TIIY

ILSSPSPWKAFST

>ModoOR1.11.29

MRGKNISNIREFILLGFPTALWLQYLLFILFLFVYLFVLVENFIIIFTIW

ANSSLHKPMYYFLGNMSFLEVWYVSDIIPKMLSGFLFQQKSISFVGCMTQ

LYFFISLICTECVLLASMAYDRYVAICFPLRYGVIMTTGLCVQLVAFSYV

IAFAISVIKVYFISHATFCGSSIINHFFCDVSPILKLACTDFSTAELVDF

VLGFIILVFPLISTMLSYGYISSTVIRIPSSIGRWKAFSTCASHLIVVII

FYTALIFMYVRPQAIDQRSSNKLISVIYTVVTPIVNPLIYCLRNNEFKNS

LKKTMAVAHTPEK*

>ModoOR1.12.1

MEWRNQSGPVTEFVLLGFPAPAPVRALLFSLSLLAYLLVLTENSLIIIAV

KSHSTLHKPMYFFLANMSFLENWYVTVTIPKMLAGFATGESGAGQRISFS

GCMTQLYFFLGLGCTECVLLAVMAYDRYVAICHPLRYPVIVSGRLCVHLA

AGSWAGGFGISMVKVFLISRLSYCGPNLINHFLCDVSPLLNLSCTDMSTA

ELTDFVLAIFILLGPLSVTGASYVAIAGAVMRIPSATGRQKAFSTCASHL

TVVIIFYAASIFIYARPKALSAFDTNKLVSVLYAVIVPLLNPIIYCLRNR

EVKQALSQTLHLHHNWGESSRKSGR*

>ModoOR1.12.2

MWEENITNVTEFILVGFPTTFWLQILLFFLFLGTYLFVLMENLVIIFTVW

INGILHKPMYYFLGIMSFLEIWYITVTVPKMLTGFLLHPNTISFLGCMTQ

LYFFLSLACTECVLLAAMAYDRYVAICHPLHYPVIMTIELCIQLTASSWV

CGFSIGIVKVYFISRVFFCGNNVLNHFFCDVSPILKLACKNFSMAEVVDF

ALAIVILLFPFSVTVLSYGFIINTVLNIPSAAGQRKAFSTCASHLTVVII

FYTAVIFMYVRPRAIASFNSNKLISVIYAVFTPMLNPIIYCLRNQEVKDA

IRKTLSSGWALFLRDSSF*

>ModoOR1.12.3P

MLEENITNVTEFILVGFPTTFWLQILLFILFLRTYLLVLVENLVIILTVL

TNVSLHKPMYYFLG/SFLEIWYITVTVPKMLAGFLLHPNTISFLSCMTQF

YFFLSLACTECVLLAAIAYDHYVAICQPPHYPVIMTVDFCIQLTATSSWI

CGFSIAVIKVYFISCVTFCGNNVLNHFFCDVSPILKLACKNISMAEMVDF

ILAIVILVFHFLAAVLSYGFIISTVLHIPSASGQQKAFSTCGSHLTVVII

FYMAVIFVYVQPRAIASFNSNKPISAIYVVFTPMLNPIIYCLRNQKAKSA

IRKTLSSGW

>ModoOR1.12.4

MREENITNLTEFILVGFPTTVWVQILLFILFLGTYLFVLIENLVIILTVW

VNVSLHKPMYYFLATMSFLEIWYISVTVPKMLAGFLLSPNTISFLGCMTQ

LYFFISLVCTECVLLASMAYDRYVAICHPLHYPVIMTTELCIQLTSGSWV

CGFSIAVAKVYFISRVSFCSNNVLNHFFCDVSPVLKLACKNFSLAETVDF

SLAIVILVFPFSATVLSYGFIISTVLHIPSAAGQRKAFSTCASHLTVVVI

YYTAVIFMYVRPRAIASFNSNKLISAIYAVFTPMLNPIIYCLRNQEVKDA

IRKTIAGGWGLFLRDSSF*

>ModoOR1.12.5

MEFWNTTLVNGFFLIGIIQDSRSPQLVCVIIIFFYLVAMATNSLLLLLII

MDNRLHVPMYFLLSQLSLMDLLFTSVVAPKTLVNYLCGQNTISFTGCGLQ

MFLALTLGGAEDILLAFMAYDRYVAIRHPLTYMVIMRPKICWSMVATSWL

LASLNALVHTIYTMHFPFCKSREIHHLLCEIPPLLKLACAETSQYKFILY

TTGVTFLLMPLSAIFASYALVLTAVFHMPPTQGKEKALLTCSSHLTVVGL

YYGAAMFMYVLPTAYHSPHQDNILSVFYTIVTPALNPLIYSLRNKDVLGA

LKKVLGKGPSVQRF*

>ModoOR1.12.6

MELWNTTLVNSFFLIGILQDSKSPEVVCAVITLLYLIAMASNGLLLLLIT

LDNRLHMPMYFLLSQLSLMDLLFTSVVAPKTLVDYLCGQNTISFIGCGLQ

MFLALTLGGAEDILLAFMAYDRYVAIRYPLNYMVLMRPAVCWSMVAISWL

LASLSALVYTIYTMHFPFCRTQEIHHLLCEILPLLKLACADTSQYELMVY

TMGVTFLLIPLSAIVASYTLVLSAVFHMPSAQGRQKALLTCSSHLTVVGL

YYGAAIFMYVLPSAYHSPRQDNILSVFYTIITPALNPLIYSLRNKDVVGA

LKKVLGKNSSEQRW*

>ModoOR1.12.7

MELWNTTLVNSFFLIGILQDSRSPEVVCAVITLLYLVAMASNGLLLLLIT

LDNRLHMPMYFLLSQLSLMDLLLTSVVAPKTLADYLCGQNTISFIGCGLQ

MFSELTLGGAEDILLAFMAYDRYVAIRYPLNYMVLMRPAVCWSMVAISWL

LASLSALVYTIYTMHFPFCKTQEIYHLFCEILPLLKLACADTSQYELMVY

TMGVTFLLIPLSAIVASYTLVLSAVFHMPSAQGRQKALLTCSSHLTVVGL

YYGPATFMYVLPSAYHSPRQDNILSVFYTIVTPALNPLIYSLRNKDVVGA

LKKVLGKNSSEQRW*

>ModoOR1.13.1P

GNQTGVSEFFLLAFSSHQDFLFLLFLVVYLASLLGNLLILMAVSSDPQLQ

TPMYIFLANLSLGDLCFTSTTVPRLLYSLFTRDQTISHTACLTQVFFFHM

AGNLSSYVLAAMAWDRYVAICRPLHYATVVTLPRCLMLLGGLWVGTSLHS

LLNTVLTARLTFCSHHDILHFHCDIYPLLQLACSDTSLNYEVILIEGSID

IILPAACIITSYGFIGVAVIGVRATGGLHKALATCGSHLAVVMLFYGTLI

IVYLLPLSQAEAQVRGGNLVASVMFSVVAPTLNPYIYSLQNHKIKLSLAR

LL

>ModoOR1.13.2

MGPGNQSTVSEFLLRGISHRPEHQQVLFGLFLFMYLVTIVGNLLISLAII

SEPHLHTPMYFFLACLSLADIGLSSTTVLKMLVNMYTQCHTISYIGCLSQ

LYFFLTFGDLDSFLLAVMAYDRYVAICRPLHYTTAMRPRICVFLVSLCWV

LTNLVALIHTLLMAQLSFCVPGEVSQFFCDIGPLLKLSCSDTHINELMVF

AVGGPVMTIPFLCIVASYILIVSAILRIPAGSGGRLKAFSTCGSHLSVVC

LFYGTVLSAYFCPSSVYSTEETASSVVYTVVTPMLNPFLYSLRNQDMKAG

LVSLLRGRLFISQAQ*

>ModoOR1.13.3P

RNQTSSSDFLLLAFSTHQELWFPLFLALYLAALLGNLMILIAVGSDPHLH

TPMFVFLVNLSLADLCFTSTTVPRLLHALLTGDQTITHSACLSQVFFFLM

AGNVDSYVLAAMAWDRYVAICRPLHYATVVTLPRCLVLLGAVWVGTSLHS

LLHTVLIAHLTFCSNRALPYFFCDLHPLLHLACSDTSLNYMMIIVEGGLV

IIMPAVCIMASYVFIVVAVCKIRAPGGFQKAFSTCGSHLTVVLLFYGTVL

CVYLQPPSQAEGQERYRDLVATVMFSVVAPTLNPYIYSLQNREIKASLVK

MF

>ModoOR1.13.4P

RNQTSSSDFLLLAFSTHQELWFPLFLALYLAALLGNLMILIAVGSDPHLH

TPMFVLLVNLSLADLCFTSTTVPRLLHALLTGDQTITHSACLSQVFFFQM

AGNVDSYVLAAMAWDRYVAICRPLHYATVVTLPRCLVLLGAVWVGTALHS

LLHTALTAHLTFCSNRALPYFYCDLHPLLHLACSDTSLNYMMIIVEGGLD

IIMPAVCIIVSYAFIVAAVCKIRAPGGFQKAFSTCGSHLTVVLLFYGTIL

CVYMQPPSQAEGQERYRDLVATVLFSVVAPTLNPYIYSLQNREIKASLVK

MF

>ModoOR1.13.5

MGHGNQTSVSEFLLRGLSQWPEHQHLFFGLFLSMYLVTMAGNVLIFLAIV

SEARLHTPMYFFLASLSLADIGLSSSIVLKMLHNLYTQHHTISYTGCLAQ

LYFFLSFGDMDSFLLAVMAYDRYMAICHPLHYSTAMSARVCVLLVSVCWL

VTNLHALLHTLLMAKLSFCVLGEISHFFCDISPLLKVSCSDTRVNELMVF

AAGGPVMTVPFLCTLVSYICIVYAILRMPSSGGGRSKAFSTCGSHLSVVC

MFYGTIFSVYLCPSSTFPTKDIVASVVYTVVTPMLNPFIYSLRNQDMKVA

LGSLFRGKIFASPIC*

>ModoOR1.13.6P

RNQTSSSEFLLLAFSTHQELWFPLFLALYLAALLGNLMILVAVGSDPHLH

TPMFVLLVNLSLADLCFTSTTVPRLLHALLTGDQTITHSACLSQVFFFLM

AGNVDSYVLAAMAWDRYVAICRPLHYATVVTLPRCLVLLGAVWVGTALHS

LLHTALLARLTFCSNRALPYFYCDVHPLLHLACSDTSLNYLVAVFEGGLV

ILMPAVCIVVSYIFIVAAVCKIRAPGGFQKAFSTCGSHLTVVLLFYGTVL

GVYLQPPSQAKGQKEGRNLATTVMFSVVAPTLNPYIYSLQNREIKAALVK

AF

>ModoOR1.13.7P

NQTSTSDFLLLAFSSHQEVLLPLFLTLYLAALLGNLLILVAIGSDPHLHT

PMYTLLVNLSLADLFFTSTTMPRLLHALLTGDRAITHSTCLAQVFFFLLA

GNVDSYVLAAMAWDRYVAICRPLHYATVVTLPRCVVLLGTVWLGAALHSL

LHTLLTARLSFCSNHALPHFYCDVYPLLRLACSDTSLNFLVALIEGGSTI

LLPAACIVTSYAYIGAAVYRVRATGGVRKALATCGSHLTVVGLFYGTLVG

VYLQPPGQHDTEGRDRDLVATVMFSVVAPTLNPYIYSLQNREIKVSLARI

>ModoOR1.13.8P

IEMSNHTSVTEFILLGFSRHFFLSFPVILLGFLLIVLLILRDPFLHMPCA

LLSY*FIHSRSLLYILYVFTDPGTLVGREKVYLLFLLYLHIPVLWHCRVL

YPLSDGL*PLCCHSRPLKVLSQDELGVFMRLVTGSWICGFGESIWHPWWQ

Q*LHSIISHFLCEISVLVHFSCANTSKVEIISQIFCVLMLLFLVMFITFS

NVHVINIVLWIRSVQGHRKTFSTWSKILCL/LYMRPKSLYSPEKHKIVIM

FYVAFPPNLNLLISSLRNKVKMALRKLLGKTGD

>ModoOR1.13.9

MESSNLTSVTEFILLGLSSRPELQVYLFFLFLMVYLMILLANLLIVLLIL

VDSCLHTPMYFFLTNLSILEVSYTSCVIPQMLGHFLAERRSLSFSRCAIQ

FYMFLFFGIAECYILSVMAYDRYVAIRDPLRYTVIMSWKVCGGLAAGCWV

GGFIASTVDTAATFQLLFCRENVINHFMCEMPALLHLSCTDTSHVEMVMN

ILCIFTLLCPVTFITFSYIHIIHVVLHIRSAQGRRKAFSTCSSHLLVVMV

LYGTVLSLYIRPRSLSSPKYDKIIAIFYVAFTPIFNPLIYSLRNNEVKMA

LRKLLGKIRNT*

>ModoOR1.13.10

MESLNLTSVTEFILLGLSSRPELQVHLFLLFFMIYLMILLGNLLIVLLTL

RDSRLHTPMYFFLTNLSIIEVCFTSCAFPQMLVHFLAKKKSISFSCCVIQ

FYTFLSFGIAECYILSVMAYDRYVAIRDPLRYSVIMNWGVCGRLAAASWL

GGFVFSAVDTVTTFQLSFCRDNIINHFVCEMPALLHLSCSDTTQAELVMH

VLCIFTLFCPITLVIISYAQIIIAVLRVRSAQGRRKAFSTCSSHLLVVTL

FYGTIISLYTKPRSLSSPEYNKIVSVFYAAFTPTLNPLIYSLRNKEVKMA

FRRLLGKPESA*

>ModoOR1.13.11P

MESSNLTSVTEFILLGLSSRPELQVYLSFFFLMAYLMILLANLLIVLLIL

VNSCLHNPMYFFLTNLSILEVSYTSCVIPQMLGNFLAERSTTFSHCSIHF

YTFLFCGIAEGWPLA/CRGLATGCWVGDFIASTVDTAATFQLLFCQEHVI

NHFMCEMPALLHLSCTDTSHVEMVMNILCIFTRLCPVTFITFSYIHIIHV

VLHIRSAQGHRKAFSTFSSHLLVVMIFYGTVLSLY/YIRP*SLTSPKYGK

IIAIFYVVFTPIFNFLIYNLRNNEANMAPKILGKSRNT

>ModoOR1.13.12

MERENQTSISEFLLLGLSSQPEQQNLLFWLFLCVYLVTVTGNLLIMLAIG

LDTRLHSPMYFFLANLSFVDICFSSVTIPKMLVNHIIGSNSISYVECMMQ

MYFFITFVNMDGFLLVVMAYDRYMAICRPLHYTTVMRPGLCILLVAISWV

ITNLHALLHTLLMAQLSFCADNTIPHFFCDPYPVRKLSCSDTYISDLMVF

TVGGVVFVTPFTCIAISYVFIFSNVMKIPSTQGMQKALSTCGSHLTVVSL

FYGAIMGIYLRPSSSYSAVDTVATVIFTVVTPMLNPFIYSLRNQDMKVAL

RRLILTKAILQMS*

>ModoOR1.13.13

MTFIERMTRENLTSISEFLLMGISKWPEHQQLLFSLFLGMYLVTVSGNLI

IILAIYSSIHLHSPMYFFLTNLSFTDICFISTTVPKMLVNICTQDHTISY

VKCLTQMYFFTLFVGLDDFLLGVMAYDRYIAICRPLHYSKAMSHRFCFLL

VSGSWIITNSCSLLHTLLVNKLSFCAENRIPHFFCDLSALLKLSCSDTHI

NDLEIVIVGGILVGAPFVLIMVSYIHIILAILRIPSTGGKYKAFSTCGSH

LCSVSIFYGTLMGVYFLPSSTHLDNLDMLAAIFYTIVTPMLNPFIYSLRN

RDMQNALRLLHRKTTLPFR*

>ModoOR1.13.14

MGWKNQTTVSEFFLLGLFEGSEHQQLLFWLFLLMYLITVIGNLLIIVAVG

SNTHLHTPMYFFLANLSFADINFISTIVPKVLMNSQTQNNAISYSDCLAQ

LYFFIMFGTIDEFLLATMAYDRYVAICHPLHYTIVMTHKLCLLLISIIWV

VNVLHALLHTLLMNELIFCAKNEIPHFFCDLNPLLKLSCSDIFINELMIL

TIGGLAGLIPFLCVIISYVYIVLAILRIPSAKGKQKAFSTCSSHLSVVSL

FFGTVFGVYFSPSSNRSAHRNIIASIMYTVVTPMLNPFIYSLRNREMKAA

LRKLF*

>ModoOR1.13.15

MNKENQTSISGFLLLGFSELPEEQIIISWLFLCVYLVTIVGNVLILLAIG

LDAHLHTPMYFFLANLSFVDICFSSVTIPKVLVNHILESKSISYKECMTQ

IYFFITFINMDGFLLGVMAYDRYVAICQPLHYTTIMCPRLCIILVMVSWV

ITHLHALLHTLLMVQLSFCANNIIPHFFCDPYPILKLSCSDTYLNDLMVF

TVGGIVFMTPFTCITISYAYIFSKVLKIPSTRGIQKALSTCGSHLTVVSL

FYGAIMGIYLRPSSSYSAQDMVATVIFTIVTPMLNPFIYSLRNRDIKTAL

QKIKRGLFKGSRIPQF*

>ModoOR1.13.16P

IQPELKTHFYFFFLIMYLTKFLGNLLFILFILVDSNLQTPMYLLLSNLSF

SDMCFSSMTIPKLLVNMKSPKPDIPYTDCLAQLFFFLLFANVESFLLEPI

AYDHYVTVMISPKLCRS

>ModoOR1.13.17

MESPNLTSVTEFILLGLSSRPELQVHLFLLFLMIYLMILLGNLLIVLLIL

RDSRLHTPMYFFLINLSVLEVCYTSCVFPQMLVHFFAERKSISYSCCVIQ

VYTFLSFGIAECYILSVMAYDRYVAIRDPLRYTVIMNWGVCGRLAAASWL

GGFMASTVATVTTFQLSFCWDNVINHFLCEMPALLHLSCSDTNHAELIMQ

VLCIFTLLSPVTFIILSYTQIIIAVLRIRSAQGRRKAFSTCSSHLLIVTL

FFGTVISLYMNPHSLSTPEYNKIVSVFYIAFTPTLNPLIYSLRNKDVKMA

LRRLLGKPEDA*

>ModoOR1.13.18

MESPNLTSVTEFILLGLSSRPELQVHLFLLFLMIYLMILLGNLLIVLLIL

RDSRLHTPMYFFLINLSVLEVCYTSCVFPQMLVHFFAERKSISYSCCVIQ

VYTFLSFGIAECYILSVMAYDRYVAIRDPLRYTVIMNWGVCGRLAAASWL

GGFMASTVDTVTTFQLSFCRDNVINHFLCEMPALLHLSCSDTNHAELIMQ

VLCMFTLLSPVTFIILSYTQIIIAVLRIRSAQGRRKAFSTCSSHLLIVTL

FFGTVMSLYMKPHSLSTPEYNKIVSVFYIAFTPTLNPLIYSLRNKDVKMA

LRRLLGKPEDA*

>ModoOR1.13.19P

MDRENQSSISEFHFLGLSNQPEQQKLLFWLFLCVYLVIVTGNLLIILAIG

LDARLHSPMYFFLTNLSITDICTSSVTIPKMLVSHIIGTSPISFVECMM*

IYFLITFVNMDGFLLMVMAYDGYMSIYRLLHYTTVM*PGLCILLVAISWI

ITNLHALLHTLLMT*LSCADNGIPNFYCDPYAIQKISCSETYISDLIVFT

KGGVVFFTPFTCIAISYVFIFINVMKIPSTQGIQKALSTC

>ModoOR1.13.20

MERENQSSISEFHFLGLSNQPEQQKFLFWLFLCVYLITVTGNLLIMLAIG

LDARLHSPMYFFLANLSFTDICTSSVTIPKMLVSHIIGSSSISFVECMMQ

MYFFITFVNMDGFLLMVMAYDRYMAICRPLHYTTVMRPGLCILLVAISWV

ITNLHALLHTLLMAGLSFCADNAIPNFYCDPYAVRKVSCSDTFISDLMVF

TVGGVVFVTPFTCIAISYVFIFSNVMKIPSTQGIQKALSTCGSHLTVVSL

FYGAILGVYLRPSSTYSAMDTVATVIFTVMAPVLNPFIYSLRNQDIKIAL

RRLILIKAIL*

>ModoOR1.13.21

MERENQSSISEFLLLGLSSQPEEQQIIFWLFLFVYLVTVIGNLLIILAIG

LDARLHSPMYFFLANLSFTDICTSSVTIPKMLVSHIIGSSSISFVECMMQ

MYFFITFVNMDGFLLMVMAYDRYMAICRPLHYTTVMRPGLCILLVAISWV

ITNLHALLHTLLMAGLSFCADNAIPNFYCDPYAVRKLSCSDTYISDLVVF

TEGGVVFVTPFTCIAISYFFIFSNVLKIPSTQGIQKALSTCGSHLTVVFL

FYGAVMGVYLRPSSTYSAVDTVASVIFTVVTPMLNPFIYSLRNQDMKVAL

RRLILTKAIL*

>ModoOR1.13.22

MERENQSSISEFLLLGLSSQPEEQQIIFWLFLFVYLVTVIGNLLIILAIG

LDARLHSPMYFFLANLSFADICFPSITIPKMLVNHMIGRNSISYVECMMQ

MYFFISFGNMDGFLLMVMAYDRYMAICRPLHYTTVMRPGICILLVAISWV

VPNLHALLHTLLMAQLSFCDDNTIPHFLCDPYAVRKLSCSDTYISDLVVF

TEGGVVLVTPFTCIAISYVFIFSNVMKIPSTQGIQKALSTCGSHLTVVFL

YYGALMGIYLRPSSTYTGVDTVASVIFTVMTPMFNPFIYSLRNQDMKVAL

QRLIISKTIL*

>ModoOR1.13.23

MERENQSSISEFLLLGLSSQPEEQQIIFWLFLFVYLVTVIGNLLIILAIG

LDARLHSPMYFFLANLSFADICFSSVTIPKMLVNHIIGRNSISYVECMMQ

MYFFISFGNMDGFLLMVMAYDRYMAICHPLHYTMVMHPGLCILLVAISWV

ITNLHALLHTLLMAQLSFCADNTIPHFFCDPYPVRKLSCSDTYISDLVVF

TEGGVVLVTPFTCIAISYVFIFSNVMKIPSTQGIQKALSTCGSHLTVVFL

FYGAIMGVYLRPSSTYSGVDTVASVIFTVMTPMFNPFIYSLRNQDMKVAL

WRLILSKAIL*

>ModoOR1.13.24P

MKRKNQSSISEFLLLGLSSQPEEQQIIF*LILFVYMVTVIGNLLIILAIA

LDARLHSPMFFFLANLSFTDICFSSVTIPKMLVNHIIGRNSISYVECMMQ

MYFFIGFGIMDGFLLMVMGYDRYMAICRPLHYTMVMHPGLCILLVAISWV

ITSLHALLHTLITTQLFFCADNTILHFFCDPYPVRKLSCSDTYISDLVVF

TEGAVVLVTPFTCIAISYVFIFSNVMKIPST*GIQKALSTCGSHLTVVFL

FYEAIMGVYLHPSSSYSGVDTVASVIFTVVIPTLNPFIYSLRNQDMKIAL

RRLILRKAIL

>ModoOR1.13.25

MGLKNQTRSSEFLLLGLFEGSEYQQLFFWLFLLMYLVTMIGNLLIIVAVG

SSTHLHTPMYFFLANLSFADISFISTTVPKMLVNIKTQNNAISYSDCLTQ

LYFFIMFGMVDEFLLATMAYDRYVAICCPLHYTLVMSPQLCFLFISITWA

FNVLYSLLHTLLMNELDFCAKNEIPHFFCDLKPLLKLSCSDIFINELMIL

TIGSLVGLIPFLCVIISYVYIVLAILRIPSAKGKQKAFSTCSSHLSVVSL

FFGTVFGVYFSPSSNHSAQRDLIASVMYTVVTPMLNPFIYSLRNRDMKAA

LVRLF*

>ModoOR1.13.26

MGFQNQTTVSEFLLLGLTQDPEHEKFLFGPFLAMYLVTMAGNLLIILAIV

SDVCLHTPMYFFLANLSFTDVCFVSNTIPKMLVNLYTQKKTIPYTGCLIQ

LYFLVSLVALDNLLLAVMAYDRFVAICRPLHYTMIMSSRLCILFLVACWA

LSILYGLTHTILMATLTFCGPREISHIFCEMYVLLRLACSDTRVNQAVLI

ITGSLLFIIPFLLMLISYVQIVKAILRIPSASGKYKAFSTCASHLAVVSL

FYGTLFGVYLQPLKTYSMKATVMYAVVTPMLNPFIYSLMNKDMKGSLKKL

LRGKTAS*

>ModoOR1.13.27P

MGFQNQTTVSEFLLLGLTQDPEVQRYLFGLFLAMYLITLVGNLLIILAIV

TDVHLHTPMYFFLANLSFTDVFFVSNTVPKMLVNLYTQKKTIPYTGCLIQ

LYFLVSLVALDNLLLAVMAYDRFVAICRPLHYTMIMSPRLCILLLVACWA

LSILYGLTHTILMATLTFCGHREISHIFCEMYVLL*LACSDTWANQAVLI

VTGCLLFIIPLSLMIFSYIQIVKAILRIPSASGKYKAFSTCASHLIVVSL

FYGTLSMVYLQPLKTYSVKDSVATVMYAVVTPMLNPFIYSLKNQDMKAAV

RKLFRGKNVS

>ModoOR1.13.28P

MKQIHDEKGNQTIISEFLLLGLPIQPEMKVPLYTVFLAMYLITVLGNLII

IFLILVDSHLHTPMYLFLSNLSFSDMCFSSVTIPKMLINMQNKKPAISYT

GCLTQMYFFFFFGDLESFLLVAMAFDRYVAICDPLHYTVIMNPKLCSALV

LLSWVLQHF/TFHALLHTLLMSQVTFSSNNIISHFFCDLSALLKLASSDV

HINELMIFIFGGLVLVVPFLLIVASYARILSSILRVPSVRGIRKAFSTCG

SHLSVVSLFYGTIIGLYLCPSSNNSTVKEIVMSIMYTVVTPMLNPFIYSL

RNQDIKGAIKKLLTGRKLPFP

>ModoOR1.13.29P

MKQIHDEKGNQTIISEFLLLGLPIQPEMKVPLYTLFLAMYLITVLGNLII

IFLILVDSHLHTPMYLFLSNLSFSDMCFSSVTIPKMLINMQNKKPAISYT

GCLTQMYFFFFFGDLESFLLVAMAFDRYVAICDPLHYTVIMNPKLCSALV

LLSWVLQHF/TFHALLHTLLMSQVTFSSNNIIPHFFCDLSALLKLASSDV

HINELMIFIFGGLVLVVPFLLIVASYARILSSILRVPSVRGIRKAFSTCG

SHLSVVSLFYGTIIGLYLCPSSNNSTVKEIVMSIMYTVVTPMLNPFIYSL

RNQDIKGAIKKLLTGRKLPFP

>ModoOR1.13.30

MKQVHGEKGNQTIFSEFLLLGLPIQPEMKVPFYAFFLTMYLTTVLGNLLI

ILLILVDSRLHTPMYLFLSNLSFSDMCFSSVTIPKLLVNMQSSKPAITYT

DCLAQLYFFLLFADVESFLLVAMAYDRYVAICYPLHYTVMMSPKLCNSLL

ALSWVLTFSISLLHTILVSRVAFCASNIIPHFFCDMSALLKLACSDIHIN

ELMIFIFGGLVLVVPFLLIVASYTRILSSILRVPSVRGIRKAFSTCGSHL

SVVSLFYGTVIGLYLCPSTNNSTVKETIMSIMYTMVTPMLNPFIYSLRNK

DMKEAIKKLLSGRKLPFSLGL*

>ModoOR1.13.31

MQQVHNEKGNQTIVSEFLLLGLPIQPELKAPCYVLFLAMYLTTVLGNMLI

ILLIHVDSHLHTPMYLFLSNLSFSDICFSSVTIPKLLVNMQSEKPAIPYP

GCLAQMYFFLFFADLESFLLVAMAYDRYVAICYPLHYTVIMSPKLCSFLV

VLSWVLTTFHALLHTLLMSRLSFCANNIIPHFFCDMSALLKLACSDTRPN

ELVIFVMGGLILVVPFLLIITSYAHIISSILKVPSVRGIRKAFSTCGSHL

SVVSLFYGTVIGLYLCPSTNNSTVKETVMSIMYTVVTPMLNPFIYSLRNQ

DMKGAFRKLLAGRKLPFP*

>ModoOR1.14.1P

ENYLLASMAYDCHAAVCMSLHYTTNMTSRVCAGVVVGTYIY/GGYLYLRY

LASSIQIVNTFSLSFCCSNVVYHFFCDIPTFLPLSCSDIHKNLSRIQCFF

DLLLILISYLSIFITVLRICSVEDCQNAFSTCGSHLSVVSIYYGTISFMY

LQPSSHHTIHTDKFASVFHTMIIPMLNFLVYSLNNKEVHNAFKKVTG

>ModoOR1.15.1

MRGENVTIISEFILVGFPTAPWLQALLFVLFLLAYLFVLAENSSIILVVW

ASRALHKPMYYFLASLSFLEVWYVSDILPKMLDGLLLGRKRISFAGCMTQ

LYFFSSLVCTECVLLASMAYDRYVAISQPLRYSILMTTGLCVRLVAFSYA

SGFTVSMIKVYFISSATFCGSNVMNHFFCDISPILKLACTDFSRAELVDF

ILAFIILVFPLISTVLSYGHICSAVLRIPSSTGRWKAFSTCASHLTVVVI

FYMAMIFMYVRPQAIDSRSSNKLISAVYTVLTPIVNPLIYCLRNKEFKGS

LRRSLGLGQAPGEEPTVLS*

>ModoOR2.1.1

MQKKNHTMVTMFTFQGFSSFHEHQITLFAVFLTLYIITLASNVIIVAVIC

IDRYLHTPMYFFLSILSTSETLYTLVIIPRMLSSLIGWNQPISLEGCATQ

MFFFVTLAINNCFLLTAMGYDRYVAICNPLRYMIVMNKKVCAHLVGGACV

IGLTMASVQVTSIFSLTFCEAEVAHFYCDILPMMKLACIDTTINEIINFV

VSLGVILVPMGLIFISYILIISAILKIASAEGRKKTFATCASHLTVVIVH

YGCASIAYLKPKSENSMEQDRLLSITYTLITPLLNPVVYSLRNKEVKDAL

RRVIGKKFS*

>ModoOR2.1.2

MQRKNYTQVTEFTLQGFSSFHEHQITLFAIFLTLYILTLVSNVIIVTIIC

IDRHLHTPMYFFLSILSTSETVYTLVIIPRMLSSLVGQNESISLEGCATQ

MFFFATLAINNCFLLTAMGYDRYVAICNPLRYMIIMNKKMCVLLVGGACA

IGLIMASIHVTSIFNLPFCGTEVAHFYCDIRPVMKLACIDTTINEIISFV

ISSLVILVPMGLVFISYILIISSILKIASAEGRKKTFATCASHLTVVIVH

YGCASIAYLKPKSENSMEQDRLLSITYTLITPLLNPVVYSLRNKEVKDAL

RRAMGKKLP*

>ModoOR2.1.3

MKIQNHTEVNEFYFQGFSSFQEHQITLFLIFLALYILTLAGNVIIVIIIQ

IDRHLHTPMYFFLSMLSTSETLYSLVIIPRMLSSLVCRDKSISLAGCATQ

MFFFVTFGINNCFLLTAMGYDRYVAICNPLRYTVIMNKKVCALLVWGAFS

IGLVVAMTQVTSVFRLPFCDIEVAHFFCDIRPVMKLSCTDTTINEILTLI

ISILVLLVPMGLVFISYVLIISTILKIASAEGRQKAFATCASHLTVVIVH

YGCAAIAYLKPKSENTKDQDQLISVTYTVITPLLNPVVYSLRNKEVKDAL

LRAIGKKHL*

>ModoOR2.1.4

MPKANITTVSEFTLEGFSSFGWNHRLILFVIFLVLYLLTLASNALILTLI

QLNRQLHTPMYFFLSVLSISETCYTVAIIPRMLTGLLHPYRPITIPGCAT

QLFFYLTFGVNNCFLLAAMGYDRYVAICNPLRYSVLMGKTTCAQLACGSW

SMGLGTAVIQVPSVFSLPFCGVSIVSHFFCDIRPLLKLACADTTIKELVT

LLISLGVIVLPMVLVFISYVLIFSTILKIASAEGRRKAFATCASHLTVVI

VHYGCTSFVYLKPKSQNSLEDRLISVTYTVITPLLNPVVYSLRNKEIKEA

LRRSLGWKSLT*

>ModoOR2.1.5

MKTKNHSEVSAFVFQGFSNFQEHQITLFVVFLALYILTLAGNVIIVTIIR

IDHHLHTPMYFFLSILSTSETFYSLVIIPRMLSSLVGLSQSISFIGCATQ

MFFFLGFAITNCLLLAVMGYDRYVAICNPLRYSVIMNWRVCTLMVSSVGI

TGFSLSLVQAVAIFRLPFCDPLIEHFFCDVRPILDLACASTFINDILTLI

ISLLAISAPATFLLITYILIISTILHIASAEGQRKAFATCASHLTVVIIH

YGCASIAYFKPKSENTKDQDQLISVTYTVITPLLNPVVYSLRNKEVKDAL

SRALRRKSLS*

>ModoOR2.1.6P

MTRSNFTAISEFTFEGFSSFKWQHRIILFVIFLGLYLLTLAVNGIIVTII

HLNRHLHIPMYFFLSVLSISETCYTLAIIPCTLFGLLSLYQPIAIPECAT

HLFFYLTFGINNCFLLMVMGYDRYVAICKPLKYTVIMGKKTCIQAACGSW

GIGLGMAIIQVTSVFSLLFCKASVISHFFCDVGPLLKLICANTVVNEIIN

FIIGMCVSLSCPWD*SSSPMFSTFLLSLGLPHLRGGGRPLLHVVIIHYGC

ASIIYLKPKSQSSLEQDRLISSAYTVLTPLLNPIVYTLRNKEVKDALRRA

LSKKPLSS

>ModoOR2.1.7

MPRPNYTVVNEFTFEGFSSFRWQNRLILFVVFLGLYLMTLTGNGIIVTII

RLDRHLHIPMYFFLSVLSISETCYTLAIIPRMLADLLSPQQPIAIPECAT

QLFFYLTFGINNCFVLTAMGYDRYVAICNPLRYSIIMRKSVCAQLACGSW

GIGLGMAIIQVTSVFGLPFCDATVISHFFCDVRPLLKLACVDTTINEIIN

FVVSVCVLVLPMGLVFISYVLIISTILKIASAEGRKKAFATCASHLTVVI

IHYGCASIIYLKPKSQSSLEQDRLISVTYTVITPLLNPVVYSLRNKEVKD

ALHRALGKKTLSS*

>ModoOR2.1.8

MKRENYSEVNEFIFQGFSSFQEHQIVLFVVFLALYILTLAGNTIIVTIIS

MDRHLHTPMYFFLSMLSISETLYTLVIVPRMLASLTGLNQPISKAGCATQ

MFFFITLAINNCFLLTAMGYDRYVAICNPLRYTVIMDKKMCAKLLCGACS

IGLTVATVQIASVFRLSFCETKVPHYFCDILQVMKLSCADTTIHDIINLI

ISSLVIVVPMGLVFISYVLIIATILKIASAEGRQKAFATCASHITVVIVH

YGCASIAYLKPKSENTRDQDQLISVTYTVFTPLLNPVVYTLRNKEVKDAL

CRAVGRKPLS*

>ModoOR2.1.9

MDCANQTWIQNFVLVGFTPTPFLQPFAFLGVLFIYLLTLAGNMFIIVLVQ

ADSGLATPMYFFISILSFLELWYISTTVPTLLCTVLHGPSPVSPTVCFTQ

LYVFHSLGMTECYLLGVMALDRYFAICRPLHYHSLMGGQVRVWLATASWV

AGFSAALVPTCLTASLSFCQVEIAHYFCDLAPLMHLACVDTAWHARVHGA

VIGVATGCNFILILLLYGGILRAVLKLPTATSRAKAFSTCSSHMMVVTLF

YGSAFAVYVGSPGGREEGSDKLIALIYALLTPFLNPIIYTLRNKEVKEAV

KRVAHRFGAVLKG*

>ModoOR2.1.10P

MEQYNYSTVTEFVLLGFPNVDDIQVWLFTLLLLAYLFTIFGNLLIFLVIR

LDSALHTPMYHFVSVLSFLELWYTATTIPK/LVRERQFPLQGCLLQTYFF

HSLGASECYLLTAMAYDRYLAICRPLHYPAIMTPALCAKMAASCWTCGFL

CPISEVILVSRLPFCGYNEIPHIFCDFPPLLSLACKDTSTNVLVDFAINT

FIIIITFLFIMASYGRIIGAVLKIKTASGRKKAFSTCASHLLVVLIFFGS

IIFMYVRLKKSYSLTLDRTLAVVYSVLTPLFNPIIYSLRNKEIMKAIKRT

ILR

>ModoOR2.1.11

MDTGNWSKVTEFIILGFPHLQGVQTYLFLLLLLIYLVTVLGNLLIFLVVR

LDAKLHTPMYQFVSVLSLLELGYTAATIPKMLANLLSKKKTISFSGCLLQ

IYFFHSLGASECYLLTAMAYDRYLAICRPLHYPTLMTPTLCTKMAAGCCL

GGLAGPIVEISLVSRLPFCGPNHIQHIFCDFPPVLSLACTDTSINVMVDF

IINSCKILATFLLILTSYVQIIRTVLRIPSAEGKRKAFSTCASHLTVVLI

FFGSILFMYVRLKKSYSLDYDRALAVVYSILTPFLNPFIYSLRNKEIKDA

VRRLLQRTRMLHI*

>ModoOR2.1.12

MTQGPEIVNQTAVTEFFFSEFPNLEEGGLLFFFPLLLIYVFIITGNLVIF

IVIQLDVALHTPMYFFISVLSFLEIWYTTTTIPKMLTNLVSVQKTISVAG

CLLQMYFFHSLGISEGCLLTAMAIDRYVAICKPLHYPTIVTPRLCSQLTA

GSCLCGFLLVLPEIVWISTLPFCGPNQIHQIFCDFTPVLNLACTDTSLIV

IVDAIHAVEILASFLVIALSYIRIILVILRIPSVEGRHKAFSTCAAHIAV

FLLFFGSVALMYLRFSATYSMFWDTAIAITFVVLAPFLNPIIYSLRNKDM

KEAIKRFLCPQKWDNRAEG*

>ModoOR2.1.13

METKNQTGISEFFFSGFPRFQEGGFLFFIPLLLIYMFVVIGNLMIFIAIR

LDVRLHNPMYNFIGIFSFLEIWYTTTTIPKMLSNLINDQKTISFTGCLLQ

MYFFHSLGNSEGILLTTMAIDRYIAICNPLRYPTIMTPRLCDQLIMGSCV

FGFLVLLPEIIWISTLPFCGSNQIHQLFCDFAPVLSLACTDTSMILVEDV

VHAIAILIAVLIIILSYIQIIAVILGIPSVEGRQKAFSTCASHLTVFIMF

YGSVSLMYLRFSATFPPFLDRTIALMFAVLAPFCNPFIYSLRNKDMKEAI

RKILSSQKVFSMSEN*

>ModoOR2.1.14P

KEEDFLIFIPLLLIYLFIVTGNLMIFIAVRLDIQLHNPMYNFISIFSFLE

IWYTTATIPRMLSNLVSDQKTISYTGCLLQMYFFHSLRNTKDILLITMTY

MLSVISSDTKP*LSDSVPR*ALTLLLFFFLVLFPEISWISTLPFCGSNQI

HQDFCDFVPVLNLACKDTPMLLV

>ModoOR2.1.15

MENKNQSMVTEFFFSVFPQFQEGGFLFFIPLLLIYLFIVTGNLMIFIAVR

LDTRLHNPMYNFISIFSFLEIWYTTATIPKMLSNLVSDQKTISFTGCLLQ

MYFFHSLGNTEGILLMTMAIDRYIAICNPLRYPTIMTPRLCAQLNIVSCV

FGFLILLPEIAWISTLPFCGSNQIHQIFCDFAPVLNLACKDTSMLLVQDI

FHAIAILSTALIITLSYIRIIIVILRIPSKEGRQKAFSTCASHLAVFLLF

FGSVGLMYLRFSATYPPIWDTTIAVMFAVLAPFFNPIIYSLRNKDMKEAI

KKLLCPKETQ*

>ModoOR2.1.16

MSFFDVESRNWSTAQEFIFSAFPHDWGDSVICFVPLLFIYAFIIVGNLVI

VIVVQTDTHLHTPMYFFISSLSFLELWYTTSTIPLMLSNLLSEKKSISLN

GCMVQLYFFHSTGISEVCLLTAMAFDRYLAICKPLHYPTIMTPKLCVQLT

LVCCICGFITPLPEVVWISTLPFCGSNHLEHVFCDFLPLLRLACTDTRAI

VMIQVVDVVHAAEIVTAVLLIFLSYAGIVAVILRIRSAEGRRKAFSTCAS

HLIVFLLFFGSVALMYLRFSATYSLFWDTVIALSFTVLSPFFNPIIYSLR

NKEIKEAVKKTYKSAKNLFL*

>ModoOR2.1.17

MDTSNWTTAQEFIFSVFPVDWGNAVTCFVPLLFIYAFILIGNLIIIMVVQ

LNVHLHTPMYFFISILSFLEIWYTTSTIPIMLSSLLSERRSISLNVCMVQ

LYFFHSTGISEMCLLVAMAFDRYLAICKPLHYPTIMTPRLCVQLTLICCV

CGFITPLPEVVWISTLPFCGSNHLEHVFCDFLPLLRLACTDTRAIVLIQV

VDVVHAMEIVADIFLILLSYAGIVAVVLRIHSAEGRRKAFSTCASHLTVV

SLFFSTVGLTYLRFSDTHSLIWDTAIALSFTVLCPFFNPIIYSLRNKEIK

EAIKKYMSQSKIFSHKAR*

>ModoOR2.1.18

MENENQTMIIEFLFTAFPHFQDGGCPFFILLLFIYLFIIIGNSMVFVAVV

LDVRLHTPMYFFIGVLAFLEIWYTTTTIPKMLINLVSDQKTISIAGCLLQ

MYFFHSIGITEAYLLTTMAIDRYLAICYPLRYPTIMTPQLYTSLTLGCCF

FGFLTPLPEITWIATLPFCGPNQIHNIFCDFDPILNLACVDTSSIILIKV

VDIIHAMEIISAITLVILAYIQIIGVILRIRSSEGRHKAFSTCASHLAIF

LIFFGSVALMYLRFNAKYSFIWDTTISLMFAVLSPFFNPIIYSLRNKEIK

GAIKKYLCQLKIFTCYST*

>ModoOR2.1.19

MGLCNETTWRDFVFLGFSSFGNLQFLLFAIFLSLYLITLASNVFIIIIIR

LDSHLHTPMYLFLSVLSFSETCYTLGIIPKMLSNLAVGSQAISYVGCAVQ

MDFSASWACTNCFLLAVMGFDRYVAICAPLHYASRMHPTLCTQLVGTSFL

SGYLIGMGMTLVIFQLPFCGSHEIHHFFCDTPPVLSLACGDTSMSELEIL

ILSLLVLLVSFSLITISYANILVTILRIPSAKGKQKAFSTCASHLTVVIV

HYGCASFMYLRPKASYSLERDQLIAVTYTVVTPLLNPIVYSLRNRAVQAA

FKNVFQGKLLGKR*

>ModoOR2.1.20

MVTEFLLLGFSNLNKMQFVLFGVILCLYLVILSGNITVITTICLEHSLHT

PMYFFLSVLSVSETSYTLVVLPKMLLNLLSVLRTISFTSCVAQMFFLLGF

GTTNCLLLGVMGYDRYTAICHPLRYPVLMSWWICKLMATTCGISGFLVSI

FGTFLVFSLPFCNSNRINHYYCDIAPVIRLACGDPYINEAIILIAGIVVL

VVPWMFICITYGFIIRTILRIPTTEGKKKAFSTCASHLTVVVFHYGCASF

VYLRPSSKHTTDKDKLVTVTYTIVTPLLNPMVYTLRNREFQVAIQKVMNW

RRFSPKII*

>ModoOR2.1.21

MVTEFLLLGFSNLNKMQFVLFGVILCLYLVILSGNITVITTICLEHSLHT

PMYFFLSVLSVSETSYTLVVLPKMLLNLLSVLRTISFTSCVAQMFFLLGF

GTTNCLLLGVMGYDRYTAICHPLRYPVLMSWWICKLMATTCGISGFLVSI

FGTFLAFSLPFCNSNRINHYFCDIAPVIRLACGDPYINEAIILIAGIVVL

VVPWMFICISYGFIIRTILRIPTTEGKKKAFSTCASHLTVVVFHYGCASF

VYLRPSSKHTTDKDKLVTVTYTIVTPLLNPMVYTLRNREVRVAIQKVMNW

RGFSPKII*

>ModoOR2.1.22

MYARPQREDNWTATTQFILLGFPTQPEIQLLLFSLFLIAYLLTLLENFMI

IVAIHSDGQLHKPMYFFLSHLSFLEMWYVTVISPKMLVDFLSHDKSISFA

GCMTQLYFFVTFVCTEYILLAVMAFDRYVAICNPLRYPAIMSNQLCSILA

GGCWICGLMTAMIKMVFIARLHYCGMPHINHYFCDISPLLNVSCEDSSQA

ELVDFFLALMVIAVPLCVVVASYAAIIATILRIPSAQGRYKAFSTCASHL

TVVTLFYSTTLFTYARPKLMYAYNSNKVVSVLYTVIVPLLNPVIYCLRNR

EVKVALKKTILCRAGGNGEDRASHA*

>ModoOR2.1.23

MGNWSGIHVENFILVGFPTSQPLQLFLFVLFLIFYLMTLLENALIISTVW

LTPALHRPMYFFLSHLSFLELWYINVTVPRLLGAFLTQNLWISFVGCMTQ

LYFFVALACTECVLLAVMAYDRYLAICEPLRYPSLMSPSLATRLATASWV

SGFFSSMMKLLFIGRLSYCGPNVINHFFCDISPLLNLTCSDKEQAELVDF

LLALVMILLPLLAVVSSYVAIIVAILRIPTSQGRQKAFSTCASHLAVVVI

FYSSILFIYARPRAMYAFNYNKVISVLYTVIVPLLNPAIYCLRNKEVKDA

LRKTVLDRCHHCGDSQD*

>ModoOR2.1.24

MSINKTLLEEFILVGFSIYPDWQVVLFVTFIFLYLLTLTGNLAIMAITWM

DHTLHTPMYLFLSALSFSETCYTLTIIPKMLVDLLAKDRSISIPGCGLQM

CFFLGLGGTNCVILTVMGLDRFLAICNPLRYPIFMTNKVCGQLVASAWIG

GFFISLTETTLIFWGSFCGPNFIKHFFCHMRAVIKLSCQNRDITEVVITI

IPVSGLLGTFLFIVLTYVFILSTVLKIPSAEGRKKAFSTCASHLTVVIIH

FGFASIVYLKPEAEEGDDTLMSVPYTVVTPFLSPLIFTLRNKDMKNALKK

VLDKKFSLTK*

>ModoOR2.1.25

MGNWSGIHVESFILVGFPTSQPLQLFLFVLFLIFYLMTLLENALIISTVW

LTPALHRPMYFFLSHLSFLELWYINVTVPRLLGAFLTQNLWISFVGCMTQ

LYFFVALACTECVLLAVMAYDRYLAICEPLRYPSLMSPSLATRLATASWV

SGFFSSMMKLLFIGRLSYCGPNVINHFFCDISPLLNLTCSDKEQAELVDF

LLALVMILLPLLAVVSSYAAIIVAILRIPTSQGRQKAFSTCASHLAVVVI

FYSSILFIYARPRAMYAFNYNKVISVLYTVIVPLLNPAIYCLRNKEVKDA

LRKTVLDRCHHCGDSQD*

>ModoOR2.1.26

MSINKTLLEEFILVGFSVYPDWQVVLFVTFLFLYLLTLTGNLAIMALTWV

DHTLHTPMYLFLSALSFSETCYTLTIIPKMLVDLLAKDRSISIPGCGLQM

CFFLGLGGTNCIILTVMGLDRFLAICNPLRYPIFMTNKVCGQLVASAWIG

GFFISLTETTLIFWGSFCGPNFIKHFFCHMRAVVKLSCRNRDITEVVVTI

ISVSGLLGTFLFIVLTYVFILSTVLKIPSAEGRKKAFSTCASHLTVVIIH

FGFASIVYLKPEAEEGDDTLTAVPYTVVTPFLSPLIFTLRNKDMKNALKK

VLDKKFSLTK*

>ModoOR2.1.27

MVSWEELPLKNLTTVTEFLLLGFSNLQDLQLVLFAIFLCLYIIILCGNIT

IVTVILLEHSLHIPMYFFLGVLSVSETCYTFVILPKMLLNLLSVLRTISF

TSCAIQMFFFLSFAINNCLLLGVMGYDRYAAICHPLRYPILMNWRACRLL

AATCGLSGFLISMVGTTLVFILPFCNSNKIRHYFCDISPVIHLACGNTYI

NEMIIFICGVLVLVVPLIFICFSYVFIVSTILRIPSTEGKRKAFSTCASH

LTVVIVHYGCASFVYLRPSSKVSSGKDQLVTVTYTIVTPLLNPMVYSLRN

RDVQMAIRKVIGFGKFSQKTT*

>ModoOR2.1.28

MVTEFFLLGFSNLQEMQFLLFVIFLCLYLAILSGNVTIVSVIILEHSLHT

PMYFFLGVLSVSETCYTFVILPKMLTNLLSVLRTISFTGCAVQMFFFLGF

AANNCLLLVVMGYDRYAAICRPLRYPILMNWRTCRILVATCGVSGFLISM

VITDLVFSLPFCKSNKINHYFCDISPVMNLACGVTYVNELFIFISSAFVL

MVPFVFICISYGFIVSTIVKIPSAEGKLKAFSTCISHLTVVVVHYGCASF

VYLRPSSRVTSVKDQLLTVTYTIITPLLNPMVYSLRNRAVQVAIQKMFNW

GEFSHKAI*

>ModoOR2.1.29P

IIPWERFSSENLTTVNEFLLLGFSSLQEKQLVLFPIFLCLYLLILCGNII

IVTVICLEHSLHIPMYFFLGVLSVSETCYTFVVLPKMLINLFSVLRTISF

TSCAIQMFFFLGFGVTNCLLLGVMGYDRYAAICHPLRYPILMNWRVCRLL

AASCGVSGFLISLIGTTLVFILPFCNSNKVEHYFCDISPVIHLACGDAHI

NELIIFICGILVLVFPLSFICISYGFIISTILKIPSAEGKRKAFSTCASH

LTVVVVHYGCASFVYLRPTSRITSSKDQLVTVTYTIVTPLLNPMVYSLRN

RDVQLAIRKVISWEGFSLKT

>ModoOR2.1.30

MIPSANLTMVTEFLLLGFSNLQEKQLMLFPVFLCLYLLILSGNITTVTAI

RLEHRLHTPMYYFLSVLSLSETCYTFDILPKMLLNLLSVLRTISFIGCAV

QMFFFVGFAVNNCLLLMVMGYDRYVAICHPLRYPILMSWRVCGQLTVSCG

IAGVIIALIMINMVFSLPFCNSNKVNHYFCDISPVIRLACTDSETREFVN

FICGVLVLVVPFSFICISYLYILRTILKIPSTEGKKKAFSTCASHLTVVL

IHYGCASFIYLRPTASYISDKDLLVTLTYTILTPLLNPIVYSLRNKDVQL

AIRKVLSRKFC*

>ModoOR2.1.31

MIPSANLTMVTEFLLLGFSNLQEKQLILFPVFLCLYLLILSGNITTVTAI

RLEHRLHTPMYYFLTVLSVSETFYTIVILPKMLLNLLSVLRTISFTNCAV

QMFFFLGFAVTNCLLLGVMGYDRYAAICHPLRYPVLMSWRVCGQLSVFCG

IAGFLLSLIMVNLVFSLPFCNSNKINHYFCDISPVIRLACTDSDTHEFVI

FILGALVLVVPFSFICISYISILRTILKIPSMEGKKKAFSTCASHLTVVI

IHYGCASFIYLRPTASYISNKDRLVTVTYTIVTPLLNPIVYSLRNKDVQL

AIRKVLVQKFCLN*

>ModoOR2.1.32P

MLPSANLMVVTEFLLLGFSNLQEKQLVLFPVFLCLYLLILTGNITIVTVI

HLEWSLHTPMYYFLSVLSVSETFYNFVILPKMLINLFSVLRTIAFTSCAV

QMFFFLGFAVNNCLLLGVMGYDRYVAICHPLRYPILMSWRVCGQLTVSCG

IGGLLVSLTTVNLVFSLPFCNSNKVNHYFCDISPVIRLACTDSDSHEFVI

FICGVLVLVVPFSFICISYLYILRTILKIPSTEGKKKAFSTCASHLTVVL

IHYGCTSF

>ModoOR2.1.33

MERSNETSVSEFIFLGFSSLAEFQWLLFVVFLLLYLFTLGTNAVILSTIL

LERALHTPMYFFLGILSSFETCYTFVIVPKMLVDLLAQEKTISFVGCAIQ

MLIFLFLGCSHSFLLAAMGYDRYVAICNPLRYTVLMGYGTCLGLVAAACA

CGFTVALVTTSLVFHLPFHSSNQLHHFFCDISPVLKLASHHTRLSQMVIF

MLGVFVLVIPLLLILASYIRIISAILKIPSTVGRYKAFSTCASHLIVVTV

HYGCASFIYLRPKTNYTSSQDALISVSYTILTPLFNPMIYSLRNKEFKAA

LWRAMGQNMCPKHSRTCSST*

>ModoOR2.1.34

MERTNETAVNEFIFLGFSSLAGFQWLLFVLFLLLYLFTLGTNAVILSTIL

LERALHTPMYFFLGILSCFETCYTFVIVPKMLVDLLAQKKTISFWGCVVQ

MLTFLFLGCSHSFLLAAMGYDRYVAICNPLRYTVLMSHRTCLGLVASACV

CGFTVALIITTLVFHLPFHSSNQLHHFFCDISPVLKLASHHTSLSQMIIF

LLSVLVLVIPLLLILISYIYIISAILKIPSTVGRYKAFSTCASHLIVVTV

HYGCASFIYLRPKTNYTSSQDALISVSYTILTPLFNPMIYSLRNKEFKTA

LWRAMGQNLCPKLAGTTSFP*

>ModoOR2.1.35

MKRQNQSRITEFILVGFSSLGDLQILLFFIFLLVYLTTLMANVTIMTVIR

LDRTLHTPMYFFLFVLSCSETCYTLVIIPKMLTNLLSTDKTISFSGCAAQ

LYFFVGLACTNCFLIAVMGYDRYVAICNPLNYMLIVSRATCIQLVLASAL

CGFMISVIVNILVFSLPFCASNRVNHFFCDISPVIKLGCTDTNMKEMIIF

FLSILVLLVPFVLIFISYVFIVSTILKISSAEGQRKAFATCISHLTVVIV

HYGCASFIYLRPTSLYSSDKDRLVAVTYTVITPMLNPLVYTLRNKEVKTA

LRRVLSRYSLPKSI*

>ModoOR2.1.36

MERANETAVSEFVFLGFSSLVGLQRLLFVIFLLLYLFTLGTNAVILSTIL

LDRALHTPMYFFLAVLSCSETSYTFVIVPKMLVDLLSQKRTISFMGCAIQ

MFTFLFLGCSHSFLLAAMGYDRYVAICNPLRYAMLMSHGVCLGLVAMACA

CGFTVSIIITSLVFHLPFHSSNQLLHFFCDISPVLKLASYHSHLVQMIIF

MLCALVLVIPLLLILVSYIHIIFAILQFPSSVGRYKAFSTCASHLIVVTV

HYGCASFIYLRPKTNYTSSQDTLISVSYTILTPLFNPMIYSLRNKEFKSA

FRRVMGRTFFSTQ*

>ModoOR2.1.37

MKRINQTTRVTKFILVGFSSLGELQMLLFVIFLLLYMTILLANATIMTVI

RCSRSLHTPMYGFLFILSFSETCYTFVIIPQLLAHLLSITKTISFVACAT

QLFFFLGFACTNCFLIAVMGYDRYVAICYPLRYMIIMNKRLGLGLISVSG

ATGFFIALVATNLICEMPFCGPNRVNHYFCDMAPVIKLACTDTHVKELAL

FSLSILVIMVPFLLILISYGFIVKTILKIPSAEGKRKAFATCASHLTVVI

VHYGCASIIYLRPKSKSASDKDQLVAVTYTVVTPLLNPLVYSLRNQEVKE

ALKKVFWRPVSAKLI*

>ModoOR2.1.38

MVTEFLLLGFSNLQEMQFVLFAVFLCLYLVILGGNITIITTVHLEHSLHT

PMYFFLSVLSVSETCYTFAILPKMLLNLLSVLRTISFTRCVTQMFFFLGF

AITNCLLLGVMGYDRYTAICHPLRYPVLMSWWICKLMVAACGVSGFLVSM

LGTFLVFSLPFCNSNRVNHYFCDVAPVIHLACGDTYMNELVIFIGGVVVL

MVPFAFICISYGFIVRTILRIPSTEGKKKAFSTCASHLTVVIVHYGCASS

VYLRPSSRHISDKDKLVTVTYTIVTPLLNPMVYSLRNRDVQLAIRKVMSW

KRFSPKNI*

>ModoOR2.2.1

MEAGNLTSVTEFVLLGLSQAQELQGFLFLVFLIVYITTVMGNLLIIITVT

SDTRLHTPMYFLLRNLAVIDLCYSSVTAPKMLMDFLSEAKTISYQGCMTQ

IFFFHLLGGGTVFFLSVMAFDRYVAISRPLHYVTIMNSQMCLGLVVAAWA

GGFVHSIVQLALLLPLPFCGPNVLDNFYCDVPQVLRLACTDTSLLELLMI

TNSGMLVLIWFLLLLASYTVILVMLRSHSGEGRRKASSTCTTHIIVVSLI

FVPCIYIYARPFTSFPLDKAVSISYTVITPMLNPMIYTLRNQEMQSAMKR

LGIRLLVCRRE*

>ModoOR2.2.2

MEAGNFTRVTNFIFLGLSETPELQDFLFLVFLIVYITTILGNLLIMVIVT

FDSRLHTPMYFLLRNLAVVDICFSSVTTPKMLIDFLSEPKTISYQGCMTQ

IFFFHFLGGATVFFLSVMAFDRYVAISRPLHYVTIMNSQMCLGLVVAAWA

GGFVHSIVQLALLLPLPFCGPNILDNFYCDVPQVLRLACIDTSFLELLMI

TNSGLLDLIWFLILLASYTVILVMLRSLSGEGRRKASLTCTTHIIVVSLI

FVPCIYIYARPFISFPIDKAVSISYTVITPMLNPMIYTLRNQEMRAAIKR

FGRHPATCRMSEIHAS*

>ModoOR2.3.1

MRRNNETSMEDFILLGLFPEFRHSGILIYILLMIYIIAFLGNSLLILLIW

GDSRLHTPMYILLSQLSLIDLTLTSTIVPKMVTNFCSGTKTISWIGCGTQ

SFFFLTLGMSECLLLTLMAYDRYVAVCNPLHYPVIMNFTVCLQMAIGCWI

GGALSSLVHTVYPMNFPICGSREIHHFFCEVPVILKLSCEDTSTYELVVM

VTSIVLLVVPFSLITASYIAIFLTVLQMNSVKGRRKALAICSSHLTVVSL

FFGPNIFIYMTLSSSHSPEQDQALSVFSNILTPMLNPVIYSLRNKEVVAA

LKKVLGKCTLSL*

>ModoOR2.3.2

MDKSNETSTTDFILLGLFPEFKYSGFLVSIIISFYIIAFTGNSILILLIW

VDSRLHTPMYFLLSQLSIIDVAYISSSVPKMALNYYLGKRNISRVGCGTQ

MFFCLTLGGSECLLLTFMSYDRYVAICKPLHYPIIMCPRICLHMAVVSWV

GGALNSLIQTIYTMHFPVCGSREIHHFFCEMPAILKLSCEDTTDYEMGIF

VVSIVFILLPFSLIVTSYTLIFLTILHMNSPEGRNKALATCSSHLTVVSL

YLGPGIVVYMTPGSSHTPELTQGLSVFYTILTPMLNPLIYSLRNKDVLAS

LRKTMTKNLISK*

>ModoOR2.3.3T

MINKSFQDHFILLGFSDRPKLEHILFVIVLIFYLITLVGNITIILVSLLD

TRLHTPMYYFLTNLSFLDLCFTTSSIPQLLFNLKGPNKTISYLGCAIQLF

MF

>ModoOR2.3.4

MRHHNYSSPDGFILLGFSDHPELEMVLSGIVTVFYLITLIGNTAVILVSL

LYPQLHTPMYFFLRNLSFLDLCFTTSIIPQMLVNLWGQDKAISYVGCAIQ

LYGYMWFGSVECLLLAVMSYDRFTAVCKPLHYLVIMNPQICLQMVSAAWG

ISLANSVVLCTMTLKLPRCGNHILDHFLCELPALVKIACIDTTAIEMTVF

ALGIIIILIPLSFILISYALIAKAVIRMKSAVGRKKAINTCGSHLTVVSI

FYGTIIYMYMQPGNSASKDQGKFLTLFYTIITPSLNPIIYTLRNKDMKGA

MKKLVKGDKGSLEIGGH*

>ModoOR2.3.5

MRAMEKINDSSEYGFILVGFSDRPKLELILFTINLTLYSVAVLGNTTIIL

VSILDPRLHTPMYFFLANLSFLDLCFSTSCIPQMLVNLWGPDKTISYAGC

AVQLFSFLSVGGIECILLAVMAYDRYAAVCKPLHYMVIMHPRLCLQLVAV

AWGSGLVNAIVMSPLTMTLSRCGRRHVNHFLCEMPALIKMACVDARAVEM

LAFTFAIPIVLMPLFFILVSYGYIAAAVIRIKSAAGRKKAFNTCSSHLTV

VSLFYGSIIYMYMQPGNSSSQDKGKFLTLFYNLVTPMLNPLIYTLRNKEV

KGALKKVFGRQKEARER*

>ModoOR2.3.6P

NYSYFYGFILLGFTDHCQLDVTISGVVFFLLYDCFDQ*HCHHPGVTPRYP

FPNTFSLETCPSWIFAISLILSLKC*STSGVRTKE*LQLFIDMVLCSVEC

ILLAVMAYDNYNAVCKPLHYMVVINPKLCQSLLTMAWLVCIINCMILSPY

AMSLPRCGNHHLDHFFCEMSATIKIDCVDTTAMEATTFGMCLVIVLVPLL

ILVSYCFIVVAVLRIKSATGRKKAFGTCSSHLIVVSIFYGTVIYMYIQPG

NSPSQDEGKLLRIFYSIITPSINPLIYTLRNKEFKEAVKRLVRE*KGT*Y

TIGH

>ModoOR2.3.7T

YLLTLLGNMAIIFLSTLDSRLHTPMYFFLSNLSFLDMCFTTGSIPQMLYN

LWGPDKSITYLGCAIQLYFVLALGGVECVLLAVMSFDRYAAVCKPLHYTV

IMHPRLCGQLASVAWLSGFGNALIMVPQTLMLPRCGHRRVDHFLCEMPAL

IGMACVDTMELEALAFSLAIFIILAPLILILISYGYIARAVLRIKSAAGR

RKAFNTCSSHLIVVSLFYGTIIYMYLQPANTYSQDQGKFLTLFYTIVTPS

VNPLIYTLRNKDVKEAVKKVLG

>ModoOR2.3.8

MSGSNESLPDMFVLLGFSDHSWIELPLFFILLISYLLAMLGNFSIILICR

LDHHLHSPMYFFLTNLSFLDICFTTSIVPQILFNLGSSKTITYIGCAIQL

YFFHFLGSTECLLLAVMSLDRYVAICKPLRYTLIMNQNICFLLVSIVWLS

GLIYSLSEVTLTLQLPLCGHNELDHVVCEMPVLIKAACGDKSSNEFGLSL

VCVFFLAIPLVLVIASYVCIGKAVMNIQSSEGRKKAFGTCSSHLIVVLLF

YGPAISMYLQPPSSISRDQPKFMALFYGVITPTLNPFIYTLRNKDVKGAI

GNVLRTMFKSKRVSVSNIK*

>ModoOR2.3.9

MMDWANETFPKEFILLGFSDWPWLEMPLFIILLVSYTMSIFGNMAIILVS

YLDPKLHTPMYFFLTNLSLLDLCYTTSTVPQMLINICSKVKTISYGGCVA

QLIIFLALGSTECLLLAIMSFDRFVAICRPLHYQVIMHQRLCLKLAAACW

LSGFSNSVLQSTWTLFMPRCGHYKVDHFFCEVPALLKLSCVDITANEAEL

FFICVLFLLIPLSLILISYGFIAQAVLRMRSAEGRRKAFGTCGSHLVVVS

LFYGAAIYMYLQPPSSTSEIWGKIVSLFYGIITPMLNPLIYTLRNKDMKG

AFKGLLKRLLFLKK*

>ModoOR2.3.10

MNNASSRGYFILIGFSDQPQLENILFWIILIFYCLTIVGNMTIILISCLE

PRLHTPMYFFLSNLSLLELCFTTSCIPQMLVNLWGPEKTISYIGCAIQLY

VFLWLGATECILLVIMAVDRSVAICRPLHYVSIMHPHLCLQLVILAWGSG

LIQSMIQVPSTLHLPFCLHHRVDDFVCEVPAMIRLSCGDTAYNEIQLSIS

SIILLVVPLVFILISYGAITKAILKVKSTAGQRKAFGICGSHLLVVSLFY

CTVTAVYLQPKGQYSHKQGKVFTLFYTVVTPTMNPLIYTLRNKEVKVALK

RLGERNWVSWENCRK*

>ModoOR2.3.11

MRRHNDTLPHPTGFILVGFSEWPRLEMVLFVAISIFYIMTLLGNTAIIIL

SYLDPRLHTPMYFFLANLSFLDLCYTTSTVPQMLVNIQSKERSITFVGCV

AQLLIFLGLGSTECVLLSVMAFDRYVAICRPLRYTVIMHSRLCHQLAIVA

WVTGFSNSLIQTVLTLHLPRCGHYRLENFFCEVPAMLLLSCADTWVNEVE

MYAAVVVIKVIPVGLILFSYANIAWAVLKIQSSEGRKKAFNTCGSHLLVV

IMFYGSAISGYAYMAPKSKSAKLKGKLLALFYGLITPMLNPLIYTLRNKD

VKAATKRLLGREQEHGWSMT*

>ModoOR2.3.12

MERTNESSPVGFILLGFSDQPQLEMVLLFVISIFYILILVGNTTIILVSY

LDPKLHTPMYFFLSNLSFLDLCFTTSIIPQMLWNLRGPDKTITYIGCVIQ

LYVALGLGSTECVLLTVMAYDRYIAICRPLHYAIIMHPRLLKQLAAVIWA

SGFIESLVQSILTFQLPLCSHHRLDDFMCEEPAVIKIAAVDTTFMENELC

IAIILYVVIPLGLILISYGCIARTTLRIKSAEGRKKVFGTCGSHLVVVAL

FFGTIIAVYIQPKNKYTENQGKFLTLFYTVVTPTLNPMIYTLRNKDVKGA

LRRLLGQNQGSGET*

>ModoOR2.3.13

MNSRYRVSLDNHTYISQFIFSNFSDVPELCYLLFAIVLLMFLTSLTGNTL

IGIVIQSTPALHTPMYFFLVNLSFLEIGYTCTVVPKMLQSLVSENREISR

LGCATQMFFFTLFGISECCLLASMAFDRYVAICTPLRYATRMSWSMCTQL

AGVSWSIGTLVALGQTNYIFSLNFCGPTKINHLFCDILPVIALACGDTSH

NEMAVYAVAFLFIASPFLLIIASYGFILADVLAMSSPEGQRKALSTCSSH

LIVVTLFYGSGSITYLRPKASHSPILDKLLALFYTVVTSMLNPIIYSLRN

KEVKAALQKTLGKIMCSVQDKN*

>ModoOR2.3.14

MERSNKSSSVGFILLGFSDQPQLEIVLLYVVSIFYFLTLVGNTTIILVSY

LDPKLHTPMYFFLSNLSFLDLCFTTSIIPQMLWNFKGPDKSITYVGCIIQ

FFVALGLGCTECILLTVMAYDRFVAICQPLHYAIIMHPRLLKQLAAVSWI

SGFLESLVQSNLTFHLPLYSHHRLDDFMCEEPALIKIAAVDTTFIETEIC

IASILHVMIPLGLILVSYGFIARTTLRIKSSGGWRKVLGTCGSHLVVVTL

FFGTILVAYIQPKKKYTQSQTKFFTLFYTVITPTLNPIIYTLRNKDVKGA

LTRLLG*

>ModoOR2.3.15

MERTNKSSPVGFILLGFSDQPQLEMILLYVISVFYILTFVGNTTIILISY

LDPKLHTPMYFFLSNLSFLDLCFTTSIIPQMLWNFRGPDKNITYIGCMIQ

FFVALGLGSAECVLLTVMAYDRYIAICRPLHYTLIMHPRLLKQLAVVIWI

SGFLESLVQSILTFQLPFCSHHKVDDFMCEEPALIKIAAVDTTFIETELC

IASILYVMMPLGLILVSYGCIARTTLKIKSTEGRRKVFGTCGSHLMVVTL

FFGTVLIVYIQPKNKYTQNQSKFLTLFYTVVTPTLNPMIYTLRNKDVKGA

LGRLLG*

>ModoOR2.3.16

MGMINVSKGGDFILVGFSEQPQVEKILFVVVLISYLLTLVGNTAIILVSC

LDPKLRTPMYYFLTNLSLVDLCFTTSIVPQLLWNLRGPAKTITPVGCAIQ

LYVSLALGSTECVLLAVMAFDRYAAICQPLHYATVMHPRLCQALAGVAWV

SGLGNTLIQSTITLNLPLCGHRKLYHFMCEVPAMIKLACVDIHANEIQLF

MASLILLLLPLGLILISYALIAQAVIKIRSAKAWKKALRTCGSHILVVTL

FYGTITAVYIQTNSSYAHRQGKFITLLYTVVTPTLNPLIYTLRNQDMKGA

LKRLLRKDLN*

>ModoOR2.3.17

MMKVMNDSTGGDFILVGFSDQPKLEKILFVVVLISYLLTLLGNTAIILVS

RLDSKLHTPMYYFLTNLSLIDLCFTTSIVPQLLWNLRGPAKTITPIGCAI

QLYVSLALGSTECVLLAVMAFDRYAAVCRPLHYATFMHPRLCQTLAGVAW

VSGLGNTLIQGTITLQLPLCGHRRLYHFICEVPAMIKLACVDIHANEIQL

FVASLVLLLLPLGLILISYALIAQQVLRIRSAQAWQKAMRTCGSHLLVVT

LFYGTITAVYIQPNSSYAHRQGKFITLLYTVVTPTLNPLIYTLRNKDMKG

ALKRLLGKEPR*

>ModoOR2.3.18

MERNNKSSPMGFILLGFSDQPQMERVLLFIIAIFYVLTLMGNTTIILVSF

LDPKLHSPMYFFLSNLSFLDICFTTSIIPQMLWNLSGTDKTITYIGCIIQ

LYIFLGVGSTECILLTVMAYDRYIAICRPLHYNVIMHPRCLKQLAAVIWV

SGFVGPLIQSILTFQLPLCSHHRVEHFMCEAPALIKIAAVDTTFIETELS

IASILYVVIPLVLILVSYGCIARTTLRMNSAEGRWKLFGTCGSHLMVVAL

YFGPIIAVYIQPKNEYTQKRTKFLALFYTVVTPTLNPMIYTLRNKDVKGS

LRRLLGSNKGSEHT*

>ModoOR2.3.19

MVGMMNDSTGGDFILVGFSDQPKLEKILFVVILISYLLTLLGNTAIILVS

HLDPKLHTPMYYFLTNLSLVDLCLTTSIVPQLLWNLRGPAKTITPIGCAI

QLCVSLALGSTECVLLAVMAFDRYAAVCRPLHYATFMHPRLCQTLVGVAW

VSGLGNTLIQGTITLQLPLCGHRRLYHFMCEVPAMIKLACVDIHANEIQL

FVASLVLLLLPLGLILISYAVIAQQVLRIRSSQAWQKAMRTCGSHILVVT

LFYSTITAVYIQPNNSYAHSQGKFITLLYTVVTPTLNPLIYTLRNKDMKG

ALKRLLGKDPR*

>ModoOR2.3.20

MMRMINDSTGGDFILVGFSDQPQIEKILFIVVLISYLLTLVGNTAIILVS

RLDPKLHTPMYYFLTNLSLIDLGFTTSIVPQLLWNLRGPAKTITPIGCAI

QLYVSLALGSTECVLLAVMAFDRYAAVCRPLHYATFMHPRLCQTLVGVAW

VSGLGNTLIQGTITLKLPLCGHRRLYHFICEVPAMIKLACVDIHANEIQL

FIASLVLLLLPLGMILISYALIAQEVMRIRSAQAWQKAIKTCGSHLLVVS

LFYGPSTAVYIQPSSSYAHRQGKFITLLYTVVTPTLNPIIYTLRNKDMKE

ALIKLLGKDTR*

>ModoOR2.3.21

MWTSNQSSLEGFVLLGFSDRPWLEKPLFVIFLVAYIFALFGNVSIILVSR

LDPQLDSPMYFFVSNLSLLDLCYTTSTVPQMLVNLWGPEKTISYGGCVAQ

LYIFLALGSTECILLAIMAFDRYAAICKPLHYPVIMNQRRCIHMAAGTWF

SGFANSLVQSTLTMVAPRCGRKIVDHFFCEVPALLKLACIDTHVNEAELN

VLGALLLLVPLTLILGTYGFIAQAVMKIRSAESRWKAFNTCASHLLVVSM

FYFTAISMYVQPPSSYSRDRGKVMALFYGIVTPTLNPFIYTLRNKDVKAA

LRRALTKEFWIKAR*

>ModoOR2.3.22P

NETTVTEFLLLGLTNIQELQFVLFVVFLILYLISLAGNCAIMMIIICEPR

LHSPMYFFLGNLSCLDICYSTVTMPKVLENFFSTHKVISFLACITQLHFF

HFLGSTEAILLAIMAFDRFVAICNPLRYLILMKHQVCLQLATAAWVISFF

YALMHSIMTSQLNFCQSHQLNHFFCDVKPLLELACGNTVLNQWLLSVVTG

SISMGAFCLTLLSYFYIIGFLFFKSRSWNRLQKALSTCASHFMVVCLFYG

PVGFTYIRPTSDSSMAQDRIVAIMYSAVTPVLNPLIYTLRNEEVKFAFKK

ALRGKFLF

>ModoOR2.3.23

MSNHTSVNELLLIGITETQELQPFLFAVFLIIYILILIGNGAILVIVISD

PRLHSPMYFFLGNLSCLDICYSTVTLPKLLDNFLSTHKTISFVGCITQLH

FFHFFGSTEAILLGVMAFDRFMAICNPLRYTILMNHEVCLCLAAAAWITG

FFHALLHSIMTSHLNFCGSNLIHHFFCDIKPLLELACGNIELNQWMLEIV

TGTLGMSPFFFILLSYFYIISFLLFKSRSCSTLQKALSTCASHFMVVMLF

YVPALFTYIRPSSGSSMDQDRIVAIIYSVVTPVLNPVIYTLRNKDVKGAL

SKAIRKKLWFE*

>ModoOR2.3.24

MPNHTSVNEFLLLGITDIRELEPFLFAVFLIIYILILIGNGAILVIVISD

PRLHSPMYFFLGNLSCLDICYSTVTLPKMLDNFLSTHKTISFVGCIIQLH

FFHFLGSTEAILLGVMAFDRFMAICNPLRYTILMNHEVCLCLAAATWITG

FFHALLHSIMTSRLTFCGSNHIHHFFCDIKPLLDLACGNTELNQWMLETV

TGTIAMSPFFFILLSYFYIISFLLFKSRSCSMLQKALSTCASHFIVVILL

YVPVVFTYIRPSSGSSMNHDRIVAIMYSVVTPVLNPLIYTLRNKDVKGAL

NKAMRKKLWSKQL*

>ModoOR2.3.25P

NHTLVYYLLFFGITETQELQSFLFAVFLIIYILILIGNGAILVIVITDPQ

LHSPMYFFLGNLSCLDICYSTVTLPKLLDNFLSTHKTISFVGCIIQLHFF

HFLGSTEAILLGVMAFDRFMAICNPLCYTILMNHEVCLCLAAATWITGFF

HALLHSIMTSHLKFCGSNHIHHFFL*HQATTGAGMWEN*AQLKGA*NSNW

HHWHEPFLFLYSFSTSILSVFSTSKVSPVFMLQKAQSICASHFMVVMLFY

VPALFTYICPSSGSSMDQDRIVAIIYIVVTPVLNLVIYTLRNKDAKRALS

KAIRKKLWFE

>ModoOR2.3.26

MPNHTSVNEFLLLGITDIRELEPFLFAVFLIIYILILIGNGAILVIVISD

SRLHSPMYFFLGNLSCLDICYSTVTLPKMLDNFLSTHKTISFVGCIIQLH

FFHFLGSTEAILLGVMAFDRFMAICNPLRYTILMNHEVCLCLAAATWITG

FFHALLHSIMTSRLTFCGSNHIHHFFCDIKPLLDLACGNTELNQWMLETV

TGTIAMSPFFFILLSYFYIISFLLFKSRSCSMLQKALPTCASHFIVVILF

YAPVVFIYIRPTSDSSMDQDRIAAIMYSVVTPVLNPLIYTLRNKDVKGAL

SKVIRKKLSVT*

>ModoOR2.3.27P

KVLASFLSTNKTISFVG*VTHLHFFNVLGSTKAILSGVMVSDHFLAICNP

L*YMLLISHQKYLCL/LFVATWITGFSDALLHSIMTFFSDIKPLLELFCG

NTEIN*WMLEIITETIAMSP

>ModoOR2.3.28

MFNHTSVNEFLLLGITDIRELEPFLFAVFLIIYILILIGNGAILVIVISD

SRLHSPMYFFLGNLSCLDICYSTVTLPKMLDNFLSTHKTISFVGCIIQLH

FFHFLGSTEAILLGVMAFDRFMAICNPLRYTILMNHEVCLCLAAATWITG

FFHALLHSIMTSRLTFCGSNHIHHFFCDIKPLLELACGNTELNQWMLETV

TGTIAMSSSFFILLSYFYIISFLLFKSRSCSMLQKALSTCASHFIVVILF

YAPVVFIYIRPTSDSSMDQDRIAAIMYSVVTPVLNPLIYTLRNKDVKGAL

SKVIRKKLSVT*

>ModoOR2.3.29

MTNYTSMNEFLLLGITDCQELEPFLFAVFLTIYILILIGNGAILAIVISE

PRLHSPMYFFLGNLSCLDICYSTVTLPKVLDDLFSNQKTISFMGCIIQLH

FFHFLGSTEAILMDVMAFDRFVAICNPLRYTILMNHQVCLCLAATSWITG

FSYALLHSIMTSRLTFCGSNHIHHFFCDIKPLLELACGNTELNQWMVETV

TGTIAMGSFFFILLSYFYIISFLLFKSRSCSMLQKALSTCASHFMVVILL

YVPVVFTYIRPSSGSSMNQDRIVAIMYSVVTPVLNPLIYTLRNKDVKGAL

NKAMRKKLCSKQF*

>ModoOR2.3.30

MALMEITFTGNQSTITEFILLGFSELPDLHLLFFIFSTIIYITIILGNML

IIVAVVSSSGLHTPMYFFLVNLSFLEILYTSTVVPKMLAGFLRKKEAISL

AGCLLQFFIFGSLATSECFLLAIMAYDRYLAICYPLHYTLLMGSKQCVCL

VVTAWLTGFVVDGLVVVLMAQLKFCGSNHINHFYCDFMPIVGLACSDPQV

AQATTFILSVICLTIPFGLILISYARILMAVLKVPTGASRKKAFSTCSSH

LAVVSTFYGTLMVMYIAPSAIHSQLLSKVFALLYTVVTPIFNPVIYTLRN

KEVHQALKKLLCNKQQGNSA*

>ModoOR2.3.31

MEKANHTLVNEFVFLKFSNSPQIQMLFFILFLLIYLLSLAANTLILLLVV

LDERLHTPMYFFIGNLSLVEIWYTTVTVPKMLANFISTRGVISVPSCIAQ

YYFFFSLAATELFILTTMAFDRYVAICRPLHYTLLLSPKTCKMLAWICWS

LGFLCPMFPSFLLTQISFCTPNQINHFFCDADQIFRLSCTDTYAIQAVGY

AFSTVIILGALLFTLLSYAQILATILAMASATARGKAFSTCTAHLSVVTI

YFGTLIFMYVRPAVKYESSINKVVAIFYSVITPLLNPLIYTLRNKDVKDA

LRIIAAQIQGHHPISLET*

>ModoOR2.3.32

MPANTSGVVTDFILVGFSRLVGFQGLLFTLFLTIYLLTVVGNLLIVTLVS

VDAKLQSPMYFFLRLLSALEIGYTSVTVPLVLHHLRTGQRRIPRVGCAVQ

MFFFLFFGATECCFLAAMAYDRYAAICTPLRYPILLNRRVCLQLAAGAWT

SGALVGLGHTSFIFSLNFCGPNAIPHFFCEIQPVLQLVCGDTYLNELQII

LAAALIILCPFGLILVSYAKILATIIRIPSATGRRKAFSTCSSHLVVVSL

FYSTAIFIYIRPKDSYDSATDPLLSLFYSVVTPILNPVIYSLRNADVKAA

LKRTLQKMSSKEI*

>ModoOR2.3.33P

YFLLGFSDWPSLQPLLFALILFCYLFTLVGNWALVFLVVRDPRLHAPMYY

FLCHLALVDVGFTTSVVPLLLANLWGWARALSRARCMTQLYVSLALGSTE

CVLLAVMALDRSAAVCRPLHYTALASPHLCRVLAAVSWFGGFTNSLAQTI

LLATRQLCGPRRLDHFICELPALLKLVCGGGGAVERQMFAARVVILLVPS

AVILASYGAVGRAVWKMKSQTGRRKAMGTCGSHLTAVCLFYGSAIYTYLQ

PTHSYSQGQGKFVSLFYTVVTPALNPIIYTLRNKEVKGAAKRLLGR

>ModoOR2.3.34P

MNNESSPGGFVLLGFSDHPQIEKVLFMIVLMSYFITLVGNTLIILLSILD

SKLHTPMYFFISNLSFLDLCFTTSCVPQLLSNLWGPEKTISFNGCAI/FL

ALGTTECILLAVMAFDRYVAICQPLRYAIIIHPRLSQQLAAVAWIIGLLE

SVIQTPPTLLLPFCSHRRVDDFVCEVPALIRLSCEDTTYNEVQMAVASVL

ILVVPLTLILVSYGAIVQAVLKINSAVGRKKAFGTCSSHLLVVSFFYSSV

ITVYLQPKNPYAQDQGKFFGLFYAVGTPALNPLIYTLRNKEVKGAFRRL

>ModoOR2.4.1

MERNFSDVTEFILLGFRVSPELQIILFLVFLLIYVVILVANIGMTVLIKM

DPRLHTPMYFFLRNLSYLDLCYSTVIAPQTLTNFLSSSKTISYNSCAAQF

FFFAFFVTTEGFLLAVMAYDRFTAICSPLLYPVRMSQSLCIRLVAGSYFG

GCINSMVQTGFTFSLQFCGENRIDHFFCDISALIKISCVDTFLNEIVLFI

LSAVIIISTSLVILVSYSYILTAVLKIPSTRGRRRTFSTCGSHIAVVSLF

YGTVFFMYAQPGALSSPEKSKVVAVFYTLVIPMVNPLIYSLRNKDVKESV

KRILGKKRSSQ*

>ModoOR2.4.2

MLMMGNQTAVTEFFLVGLTDDPEIQVILFIFLLLTYLLSFTGNMTIIILT

LLDSQLQTPMYFFLRNFSFLEISFTTVFVPKMLVNIGTGNKTISFAGCFT

QFFFAILLGATEFYLLAAMSYDRYVAICKPLHYTMMMNKKLCLQLILSSW

FSGFMVVIGPHIMTTMLPFCASNVINHYCCDYTILLQLACADTHIIEIIT

FVLAVVTLLFTLVLVIISYVYIMGTIMRIPSAQKRKKAFSTCSSHMIVVS

LSYGSCIFMYVNPSMKEAATFNKGVAVLNTSVAPLLNPFIYTLRNQQVKR

AFKDMVRRIKFFSIK*

>ModoOR2.4.3P

MINKSFQDHFILLGFSDRPKLEHILFVIVLIFYLITLVGNITIILVSLLD

TRL/TPMYYFLTNLSFLDLCFTTSSIPQLLFNLKGPNKTISYLGCAIQLF

MFLGLGGTECILLSVMAYDRFIAICKPLHYTVIMHPQLCVQLVAVSWGIG

LLNSLVMSPITMKLPRCGRCQVKHFLCEMPALIKMACVDTVVMESTVFIL

SVIIVLVPLSLILISYGYIAQTVL

>ModoOR2.4.4

MIIMQGNESSLQGFILLGFSDRPRLELVLFVMVLIFYLMTLVGNAIIILV

SRLDPHLQTPMYFFLTNLSFLDICFTTSSIPQLLFNLGGSDKSISYTGCA

IQLFMFLALGGTECILLAVMAYDRFTAICKPLHYTVIMHPQLCLQLVAVA

WLSGIANSLAMSPVTLTLPRCGRHQVDHFLCEMPAMIKMACVDTTLIEST

VFVLASCFTLLPLSLILVSYGYITRTVLRIKSAAGRRKAFNTCGSHITVV

SLFYGNIIYMYMQPGNNSSQDQGKFLTLFYNLLTPSLNPLIYTLRNKEVK

GALRRLVKKERG*

>ModoOR2.4.5

MKQNNESSFTGFILLGFSDQPQLELLLFILLLIFYLFTIFGNTTIILLSQ

LDPRLHTPMYFFLSNLSFLDLCYTTSIVPQLLVNLWGPDKSISYVGCASQ

LYISLALGSTECILLGMMAYDRYAAVCRPLHYMIIMHPRICSQMAAISWL

SGFFNSLIQTTLTLHVPLCGKNRIDHFLCEVPPLLKLACVDTTINEAELF

VVSVLFLLVPISFILVSYGHIVWTVLHMKSESGQRKAFWTCGSHLVVVSL

FYGTAIYAYLQPTNSYSQDQGKFISLFYTVITPMINPLIYTLRNKDVKGA

IKKALHMES*

>ModoOR2.4.6

MKLNNESSFTGFILLGFSDQPQLELLLFMLLPIFYLFTIVGNTTIIFLSQ

LDPRLHTPMYFFLSNLSFLDLCYTTSIVPQLLVNLWGPDKSISYVGCASQ

LYISLALGSTECILLGMMAYDRYAAVCQPLHYMIIMHPRICNQMAAISWL

SGFFNSLIQTTLTLHVPLCGKNRIDHFLCEVPPLLKLACVDTTINEAELF

VVSVLFLLVPISFILVSYGRIVWTVLHMKSESGQRKVFWTCGSHLVVVSL

FYGTAIYAYLQPTNSYSQDQGKFISLFYTVITPMINPLIYTLRNKDVKGA

IKKALHMES*

>ModoOR2.4.7P

SALSSRLQAPHSHVLLPQKPFLSRPLLHPSIVPQMLVNIWGGSKKISYSG

CMVQYWVALALGSTECVLLAVMAVDRYVAVCWPLRYAMIMHPRLCHLLGA

TSWSSGFANSLLQSSLAMVLPLCGNRRVDHFFCELLVIVKLSCVDTGPTE

SKMFIAHLIILAMPVSIILTSYGCIAWAVMKIHSAEGRKKAFGTCASHLL

VVSLFYGTIMFVYLQPKNNYSQDQGKILAVLYTILAPTLNPLIYTLRNKD

VKKAVKN*WGRSR

>ModoOR2.4.8

MKNEQSMEVNDSSGGYFILMGFSDRPQLELVLSLFFFVLYTITLMGNMTI

IILSFLDSRLHTPMYFFLRNLSFLDIGFITSVVPQMLINIWGHNKRISYI

GCLVQYSVALALGSTECVLLAVMAVDRYFAVCWPLRYATIMHQRLCHLLA

ATSWFSGFANSVLQSSLAMVLPLCGHRQVDHFVCELLVIVRLSCVDTRSM

ESKMFIARLIILAIPVSIILTSYAHIAWAVAKIHSAEGQRKAFKTCASHL

MVVSLYFGTIMFIYLQPRNNYSQVQGKVLALIYTIIAPSLNPLIYTLRNK

DINRAMKKVMGKQDT*

>ModoOR2.4.9P

EMPVFLKLACKDTKDTEAKMFVARVIIMVLPASLLLVSYVYLVQTVLNIK

SAEGQRKAFETCGSHLMVVFLFYGSAIYTYLQPTHSYSTSHGKFAALFYT

IITPMLNPFIYTLRNTDMKAALRKVIKRKRALG

>ModoOR2.4.10P

MRQFNTSSQDFILLGFSDWPLWNISSL*SS*SFIS*PWLATQPSLLCPIC

FLSSIPPCFFFLCHLSFLDLCYTTSIVPQLLVNLHGFDRTITKGGCVAQL

FISLALGSTECVLLVVMAFACYAAVCRPFHYTTIMQPWLCLLLASISWFG

GFVNSVAQRGLMMAMPLCGLCYLDHFFCEMPVF

>ModoOR2.4.11

MEIANDSSDDFILVGFSERPELELILSLFVSLFYIITLTGNTAIILLSLL

DSRLHTPMYFFLRNLSFLDLCFTTSIVPQMLVNIWGGSKKISYSGCMVQY

WVALALGSTECVLLAVMAVDRYVAVCWPLRYAMIMHPRLCHLLGAASWSS

GFANSVLQSSLAMVLPLCGNRRVDHFFCELLIIVKLSCVDTGPTESKMFI

ARLIILAMPVSIILTSYGCIAWAVMKIRSAEGRKKAFGTCASHLLVVSLF

YGTIMFVYLQPKNNYSQDQGKILAVLYTILAPTLNPLIYTLRNKDVKKAV

KKLIGKEQA*

>ModoOR2.4.12

MKNEQSMEVNDSSGGYFILMGFSDRPQLELVLSLFFFVLYTITLMGNMTI

IILSFLDSRLHTPMYFFLRNLSFLDIGFTTSVVPQMLINIWGHNKRISYV

GCLVQYSVALALGSTECVLLAVMAVDRYFAVCWPLRYATIMHQRLCHLLA

ATSWFSGFANSVLQSSLAMVLPLCGHRQVDHFICELLVIISLSCVDTRSM

ESKMFIARLIILAIPVSIILTSYAHIAWAVAKIRSAEGQRKAFGTCASHL

MVVSLYFGTIMFMYLQPKNNYSQDQGKVLALIYTIIAPTLNPLIYTLRNK

DINRAMKKVMGNT*

>ModoOR2.4.13

MRQFNTSSQDFILLGFSDWPHLEHLLFVIILIFYLLTLVGNTAIIVVSNL

FPQLHTPMYFFLCHLSFLDLCYTTSIVPQLLVNLHGFDRTITKGGCVAQL

FISLALGSTESVLLVVMAFDRYAAVCRPLHYTTIMQPWLCLLLASISWFG

GFVNSVAETGLMMAMPLCGLRYLDHFFCEMPVFLKLACKDTKDTEAKMFV

ARVIILVLPASLLLVSYVYIIQAVLNIKSAEGQRKAFETCGSHLMVVFLF

YGSAIYTYLQPTHNYSTSHGKFIALFYTIITPMLNPLIYTLRNKDMKAAL

RKVIKRKRALG*

>ModoOR2.4.14

MRQFNTSSQDFILLGFSDWPHLEHLLFVVILIFYLLTLVGNTAIIVVSNL

FPQLHTPMYFFLCHLSFLDLCYTTSIVPQLLVNLHGFDRTITKGGCVAQL

FISLALGSTESVLLVVMAFDRYAAVCRPLHYTTIMQPWLCLLLASISWFG

GFVNSVAQTGLMMAMPLCGLRYLDHFFCEMPVFLKLACKDTKDTEAKMFV

ARVIILVLPASLLLVSYVYIIQAVLNIKSAEGQRKAFETCGSHLMVVFLF

YGSAIYTYLQPTHSYSTSHGKFAALFYTIITPMLNPLIYTLRNKDMKAAL

RKVIKRKRALG*

>ModoOR2.4.15

MEWSGNQTFITHFVLLGLFSHTPLHHFLFSLIMIMFLVALTGNGLMILLI

NIDSRLHSPMYFFLSWLSCMDLMLISTIVPRMAVDFLSDGGYISFTGCGL

QILFFLTLLGDECFLLAFMAYDRYVAISNPLRYSLVMNQRICWLMVAFSW

LFGLIDGLIQAVFTLHFPYCGSKEIDHFFCEVPAILKLACADTSLYESMI

YVCCVLMLLLPFSVISASYLRILVAVLQMRSTEGRKKAFATCSSHMTVVT

LFYGAAMVTYIRPQAYHSSKQDKVVSAFYTMITPMLNPMIYSLRNKEVTG

ALKKLLGRCSCGQREDSQ*

>ModoOR2.4.16

MLNRRNMGPWLNHSSVGDFILLGIFSHSQTDLVLFSAVLVVFTVALAGNI

LLLFLIYSDAQLHTPMYFFLSHLSFMDLTLICTIVPKMADNFIHERKSIS

FIGCGLQIGLFVTLVGSEGLLLGLMAYDRYVAINHPLRYHVLMSQRVCLQ

IVGSSWAFGILDGLIQVVAAMTFPYCGSREVDHFFCELLSLLKLACADTS

FFDTLIFSCCVFMLFLPFSIIVASYAKILRAVISMNSAQAQQKALTTCSS

HLAAVSLFYGAAMFIYLRPKHYRAPGHDKIVSLFYTVLTPMLNPLIYSLR

NREVMGALRKKLNRWVTDCQK*

>ModoOR2.4.17

MLNRRNMGPWLNHSLVGDFILLGIFSHSQTDLVLFSAVLVVFTVALAGNI

LLLFLIYSDAQLHTPMYFFLSHLSFMDLTLICTIVPKMADNFIHERKSIS

FIGCGLQIGLFVTLVGSEGLLLGLMAYDRYVAINHPLRYHVLMSQRVCLQ

IVGSSWAFGTLDGLIQVVAAMTFPYCGSREVDHFFCEVPALLKLACADTS

LFDTLLFSCCVFMLLLPFSIIVASYAKILRAVISMNSAQARQKALTTCSS

HLAAVSLFYGAAMFIYLRPKRYRAPGHDKIVSLFYTVLTPMLNPLIYSLR

NREVMGALRKKLNRWVTDCQK*

>ModoOR2.4.18

MNLGNQTSKTDFFLLGLFNTSELHYFLFSLAFISFLLTLLSNGLMVFLIY

VEVQLHTPMYFFLGQLSFMDMLLACTTVPKMATNFLSGRKSISFVGCGFQ

IFFFLTLGGGECFLLAFMAYDRYVAITKPLRYPTIMTNCVCWLMAATSWL

LGVLDGLIQGLVTLTFPFCGLRVIDHFFCEVPAVLALACADTTTFETVMY

VCCVGMLLIPFLIILASYAQILVAVLQMTSTKGKKKAFSTCSSHLAVVIL

FYGTVISIYMVPHSHRSPGVDKTVAAFYTFFIPTLNPFIYSLRNKDVMGA

LGKLLGKIRLCRNETVQVV*

>ModoOR2.4.19

MEKRNYTTVTEFILLGLSSQPEKQELIFALFLVMYLIGAAGNLLIVLAIS

LDSHLHTPMYFFLSNLSLVDFCFTSATVPKMLLNIHTQTKSISREGCLTQ

IYFCILLANMDNFLLTTMAYDRYVAICYPLQYTTMMSLQLCFLMLAGSWL

IANFHSLLHTLLMARLDFCAKNVIPYFFCDLVPLLQLSCSDTQINQLMIL

LVGGLIVLIPFLCILVSYIHIVSAVLKVPSARGKQKAFSTCGSHLTVVIL

FYGTITGVYLNPSSSHSTEKESVASVMYMVVTPMLNPFIYCLRNNEIKRA

LKKMFSRQNSSHSP*

>ModoOR2.5.1

MGRGNDSSLGGFILVGFSDRPWLEMILFAFVLVFYILTLLGNTTIIVLSI

ADSRLHTPMYFFLGNLSFLDLCFTTSIVPQLLWNLWGPKKTISYNGCVAQ

LYIYMVLGSTECVLLCVMSYDRYVAVCRPLHYTVVMNPRLCLQLTIVSWF

CGFLNSFVMCPQTMQLARCGHHQVDHFLCEMPALIAMACEDTTLVEAFAF

IFGVILLLVPLSLILTSYGLIAVAVLRIKSAAGRKKAFNTCSSHLAVVSL

FYGTIIYMYLQPANTYSQDQGKFLTLFYTVLTPSINPLIYTLRNKDVKGA

MKKLLGWEQRTREA*

>ModoOR2.5.2

MEKINMSISEGFLLLGFSDRPLLEMILFIFVSIFYMLTLLGNATIMLVSH

LDARLHTPMYFFLTNLSFLDIGFTTSIVPQLLVNLRGPEKTISYAGCVIQ

FYISHWLGATECVLLAVMSYDRYVAICRPLHYTIIMNSRVCWGLASTAWV

GGLSTSLLGSTLTVQLPLCGYNRIDHFFCEMPLIMQLSCVDTSLNELEMY

VASFIFVVLPLGLILVSYSQIARAVLRIKSTEGRAKAFNTCSSHLMIVFL

FYGSIIFMYLQPAKSSSHEQGKFVSLFYTVVTPMLNPLIYTLRNKDVKGA

LKQLVIGSGCGPVKKPGAIS*

>ModoOR2.5.3P

LLFLTGNALIIIIVFVEPMLHTPMYIFLGNFSFLEILYTTVTVPKLLATC

VSRLIIIPVAECVVQYYFFFIGATECILLTVMAYDR*SPGLCNLNKSSSL

PLVPS*LLGMWFYCSIAAHNSHFPSLLLWPSEDNHFFCESDPIFKLSYSE

TFLVEAL/YTCSSVVILSSFLLTMSSYDHIIITIINMPSWKAKKEAFPIC

ASHLTVVTTYHGTIIFAYICPPAKYNFTIGKFISVFYCVITPLLNPIIYT

LRNKDMEKPLK

>ModoOR2.5.4

MNHSTVTEFVVMGLSGQPELQGIFFIIFFFIYLVALLGNVIIVIAIIYNT

TLHSPMYLFLLALGVVDVICTSTIIPKMLENMLVLDKTISYEGCMSQLFF

FTWSLGAEMVLFTVMAYDRYVAICFPLRYSTIMNRNMCMALLCTVMAIAI

ANSWVHTGLILRLTFCGPNTINHFFCEIPPLLALSCSPVRTNEVMVFAAD

IALAMGDFILTCISYGFIIAAILRIRTTEGKRKAFSTCSSHLLVVSLYYS

PVIYTYIRPASTYNFERDKVVAALYTLVTPTLNPLVYSFRNKEIQGGIKK

VFSFLKCH*

>ModoOR2.5.5

MRSNNQSLLGTFTGNFFLLGVSDRPWLELPLFVILLISYVLAVLGNIAII

LVSRLDPLLSSPMYIFLSHLSFLDLCYTTTTVPQMLFNMGSSKKTISYSG

CTVQYAIFHWLGCTECVVLAAMALDRYVAICKPLRYAVIMNRPLCQQLVG

TAWISGFGNSLVQVVLTVQLPFCGRRVLNNFFCEVPAMIKLSCANTSIND

ATLAVLVAFFVLVPLVLILISYGFIAQAVMRIKSSKGRSKAFGTCSSHLI

VVSLFYLPAIYMYLQPPSSYSQDQGKFISLFYSIITPTLNPFIYTLRNKD

VKGALRRLWERSWRLFQR*

>ModoOR2.5.6

MEKRNWTMVTEIILVGIPTTHAVLGGLLFLLFLLAYLVTVLGNFLIIILI

WVDYRLHSPMYFFLSHLSFSEILTTTCAVPKMLVDFLSEKKTISFAGCFS

QSYFYFLSGCTEFILFAIMSYDRYIAICNPLQYPTIMTCPLCIRLVVLSW

LGGFLLIFPSTVLKGGLPYCGPNVIDHFFCDSAPLLHLACTDIRAIELLD

FLSSLVLLISSLTLTVVSYVYIISTILKIPSGQGQRKTFATCASHFTVVS

LGYGISIFVYVRPSQKSNLHFNKILFILSSIVTPLLNPFIFSLRNETMKE

ALKDISAKIQTFLKNLRFN*

>ModoOR2.5.7P

MYFFLSHLSFSEILTTTCTVPKMLVDFLSEKK/IREKINISFAGWFSHFY

FYFLSGCTEFILFSVMSYDHYIVICNPFNIP/LQYPTIMTCPLCVRLAIL

SWLGSFLLIFPSTVLKGVLPYCESNVIDHFFCDSAPLLYLACADIRAIEL

LDFLSSLVLLIGSLTLTVVSYVYIISTILKIPSGQGHCKAFATCASQFTM

LSLSYGISIFVYVPPSQRSSLHFNKILFVLSSIVTPLLNPFIFSLRNETM

KEALK

>ModoOR2.5.8

MEFWNATTIMKFQLLGFQNISEWQNFLFAIFLFFYILTIIGNILIIVVVS

QDQRLHLPMYSFLKHLSFLEIWYTTTIVPLLLTNLLSQGLSISFSACMIQ

LYFFVFFGATECFLLAMMAYDRYLAVCNPLHYTTLMSSEVCIKLIFGSWI

TGIGTGFLPALMISELNFCGPNQINHFFCDLPPLMQLSCSSTFITEITIF

ILSIAVLCICFLLTLVSYVFIVSSVLRIPSASGRIKTFSTCGSHLAVVTI

YYGTMISMYVRPNAHKSPELNKIISIFYTVVTPLLNPVIYSLRNKDFKEA

VRKVIKRKCSMYSV*

>ModoOR2.5.9

MMDGTNESIQDDFILLGFSDRPQLEVVLFVVILIAYLLTVVGNTTIILLC

HIDPRLSSPMYFFLTHLSFLDLCFTTSSIPQLLYNLNGPDKTISYTGCAF

QLFLFLALGGVECLLLAVMAYDRFVAVCKPLHYMVIMYPKLCLGLVSIAW

FGGVLNSLTMSPITLKLPRCGHNKVDHFLCEMPALIRLACVNTAIIEGTV

FVLAVIIVLTPLVLILVSYGYITQAVVQIKSAVGRRKAINTCSSHLIVVS

LFYGNIIYMYMQPGNNASQDQGKFLTLFYNIVTPLFNPLIYTLRNKEVKG

ALRRLLGEKKGRREK*

>ModoOR2.5.10

MKPENQTSSSGFILLGLLVDDKISGIVFAIILSIFVVALTANVVMIFLIQ

VDSRLHTPMYFLLSQLSVMDTLFICTTVPKLLIDMISEEKTISFVGCGIQ

IFLYLTMIGSEFFLLGLMAYDRYVAVCNPLRYPVLMNRRMCLLLAAGSWF

GGSLDGFLLTPITMNVPYCGSRTINHFFCEIPAVLKLACADTTLYETLMY

ICCVLMLLIPISIISTSYSLILLTVHRMRSADGRKKAFTTCSSHLTVVSI

FYGAAFYTYVLPRSYHTPEKDKVVSAFYTIVTPMLNPLIYSLRNKDVMGA

LKKVLFKCSPTQKLTASAA*

>ModoOR2.5.11

MEWGNYSMYADFVLLGFFSNTKFPWLFFSLIFLVFVISVVSNVIMIILIH

IDPRLHTPMYFLLSQLSIMDILYISTIVPKMLVDQLMGQKTISFAGCTAQ

HFLYLTLAGAEFFLLGLMSYDRYVAICNPLRYPVLMNRKVCLLIVTAAWL

GGSVDGFLLTPVTMQFPFCASREINHFFCEVPALLKLSCTDTSTYETAMY

VCCIMMLLIPFSVISASYTRILITVYRMSEAEGRRKAVTTCSSHMVVVSL

FYGAAMYTYVLPHSYHTPEQDKAVSAFYTILTPMLNPLIYSLRNKDVTGA

LKKALGQCFSRRIRVF*

>ModoOR2.5.12

MDGYNTTSSTDFTLVGLFSNRETSGLLFAIICAIFFTALLANGIMIFLIY

TDLHLHTPMYFLLSHLSFIDMMYISTIVPKMLVDYLLGQRTISFVGCTAQ

HFLYLTLVGAEFFLLGLMAYDRYVAICNPLRYPILMNRRVCLMIIAGSWF

GGSLDGFLLTPITMSFPFCDSRKINHFFCEAPAVLKLACADTALYETVMY

VCCVLMLLIPFSVVIASYARILTTVHQMSSVEGRKKAFATCSSHMTVVTL

FYGAAMYTYMLPHSYHTPAQDKILSVFYTILTPMLNPLIYSLRNKDVAGA

MKRVLGKCKASHRVAGTF*

>ModoOR2.5.13P

MDGDNRTNPINFFLMGFFSHNVASGFIYIIIFIIFFIALIANGAMIFLIN

TDTQLQTPMYFLLSHLSFIDIIYISTIVPKMLVSYFSAQETISFVACTAQ

YFLYQTFVGAEFFLLGLMAYDRYVAICHPLHYSVLMSRQICLIILASSWF

G/FLLTPITMSLPFCASNKINHFFCEAPTMLRLACGDKAIYEMVMYICCV

MMLLIPFSVVIISYTKILITVHQMNSPEGGKKALATCSSHIIVVTLFYGA

ALYTYMLPQLYHTPVKDKIFSAFYTILTPMINPLIYSLRNKDVTNAMKRV

LGRCKRIPRVTG

>ModoOR2.5.14

MKQTIWLENHTLGTDFILIGLLSQSKYPTVSSSVIFIVFIVAVTGNATLI

LLIYCEPHLHTPMYFFISQLSLMDMMYSFIIVPKMLLDQVTGIHNILIPS

CGMQMFLYLTLVGAEFFLLAAMSYDRFVAICHPLRYPILMNHRVCKFLII

GCWFLGSIDGFMITSITMTFPFCKSREIQHFFCETPALTKLSCSDTSFYK

IAMYLCCFLMILIPVIVILGSYSLILLTVYRMNSVTGYKKAFSTCSSHMI

VVILLYGAAAYNYMLPPSYHTAKKDMMVSVFYTIITPALNPFIYSFRNKD

ITGSLRKLLAVKRVTGTYGE*

>ModoOR2.5.15

MKQTMWLENHTLGTDFILMGLFSQSKHPTLLCSVIFIIFMVALIGNATLI

LLIQRDSHLHTPMYFFISQLSLMDMMYISVTVPKMLLDQVTGTHSISTPS

CGIQMFLYVTLVGTEFFLLAAMSYDRFVAICHPLRYPILMNHRVCILLVT

VCWFLGSIDGFMLTPITMNFPFCKSREIQHFFCEVPALMKLSCSDTSVYE

TVMYLCCVLMLLIPITVISGSYSLILLTVHRMNSATGRRKAFATCSSHMA

VVILFYGAAIYTYMLPSSYHTTEKDMIVSVVYTILTPVLNPLIYSLRNKD

VTAALKKVLWEELVLANLQK*

>ModoOR2.5.16

METLYQNSTDFIFLGLFANSKIPGLIFTILLSIFVVAIMANVVMILLIHV

DSKLHTPMYFLLSQLSVMDTVYICITVPKMLADILSEEKNISFLGCAVQI

FLYLTLFGSESFFLGLMAYDRYVAVCNPLHYPLLMNHRVCLLMVIGSWIG

GSLDGFMLTPVTMSFPYCGSRKINHFFCEIPAVLKLSCADTSLYETLMYA

CCVLMLVVPISLISFSYIRILITVTQMKSAEGRHKALTTCSSHIMVVSIF

YGAAFYSNVLPRSFHTPEKDKIVSAFYTILTPMLNPLIYSLRNKDVSAAL

KKALRKCASSQEIQVRD*

>ModoOR2.5.17P

MKETMWLENETLGTDFILLGLFNQSKCPTLLYSVILVVFIVALTGNATLI

LLIYSDLHLHTPMYFFISQLSLMVMMYISVTVPKMLLIQVTGIHASSFPS

CGIEMFLYVLLGGAEFFLLAAMSYDRYMVIYHPLQYPILMNHQVCMLFEA

VCWLLGSINGFMLTFITMTFPFCKSREI*HFFCEVPDLIKLSCSDTSLYK

IAMYRCCVFMILITVTVISSSYSLILLTVYRMTLATGRKKAFVTCSSHMS

VVILFYGSAVYTYMLPTSYHSVDKDMMISVFYSILTPVLNPLIDSL*NKD

VTAALKKVLGKLTLVNIGK

>ModoOR2.5.18

MHLFLFSIVVLIYVLAVTGNLTMVLLIWADSRLHSPMYFLLSQLSFLDIF

FTSVTVPKMIVGFLWGWTSISFGGCGAQMFFFMFLGAAECLLLALMAYDR

YVAICNPLRYPLLMSRRTCCLMVIASWLGGSLNASIQTILTLQFPYCGSR

KIAHFFCEVPSLLQLACADTAAYEKVLFVTGVVVLLVPIAFITASYALIL

AAVLRMKSAEGRHKALATCSSHLTVVNLFYGPLVYTYMLPASYHSPGQDD

IVSVFYTVFTPMLNPIIYSLRNKEVSGAMKKVIVKCIVTRNG*

>ModoOR2.5.19P

STDFILLELFLDMKHISFLVLIILLIYIISLTANTGLIFLDPQLHIPMYF

LLSQLAIMKVTLISSTVPKMVDDFFSRKTKISHVTCVTQIFFFLTLGIAE

SILFTLMAYDHYVAICKPLKYPVFMSHRTCIKMTCVS*LGGAFISLVHTA

YVMHFPICGSRKIPDFLCEVMAILKITCEDTRAYEKAVVVTSIVVLLISL

SLILSSYILIFLNVLYMNSLKGRNKALTTCSSHLTIVSLYYGLASIVYMR

PSYYHRPKIDQILFVLGVILTPMMN

>ModoOR2.5.20P

MGRVNQTTDFILLGLFPEIKHLAVLISIIFLIYMIAVTGNTLLIFLILGD

SQLHTPMYFLLSQLSLIDLALISTVVPNMMTSFFSGRRNISQTGCGTQIF

FTLTLGISECLLLTFMSYDRYVAICNPLRYSVIMSHTVCMQMVIGSWLGG

TLTSLVHTAYAMHFPICGSREILHFFCEVMALLKLTCEDISAYEKSVVVS

SFLVVLIPLSLILLSYAFIFLTVLRMNSPEGRNKALATCSSHLSVVSLYF

GPAILIYMRPGSSQTPKLNQALF/FLFDSILTPMLNPIIYSLRNRDVLAA

VNKILRNMFTSRKIQRHLGCIT

>ModoOR2.5.21

MGRVNQTTDFILLGLFPEIKHLAVLISIIFLIYMIAITGNAVLIFLILGD

SQLHTPMYLLLSQLSLIDLALISTVVPNMVTSFFTGRKNISQTGCGTQIF

FILTLGISEFLLLTFMSYDRYVAICNPLRYSVIMSPTVCMQMVIGSWLGG

TLTSLVHTTYVMHFSICGSREILHFFCEVMALLKLTCENTSAYEKSVVIS

SFLVVFIPLSLILLSYTFIFLTILRMNSPEGRSKALTTCSSHLSVVSLYF

GPAILIYMRPGSSQTPKLNQALFLFDSILTPMLNPIIYGLRNKDVLAAIR

KILRTIFSRGKIQRHLGCIT*

>ModoOR2.5.22P

SADLYVL*PLCGHLQSPSLLSHHESHCRMQMVIGSWLGVTLTSLVHTAYA

MHFPICGSREILHFFCEVMALLKLTCENTSAYEKSVVISSFLVVFIPLSL

VSLSYTFIFLTILCMNYPEGKNKALVTCSSRLSVVKSLFCSSYINLHEVR

LYLTPKLNQALFSFDSILTPMLNLIIYGFRNNDVLSAVRKIFRNIFPPGR

IQRQLG*IT

>ModoOR2.5.23P

RVN*TTDFILLGLFPEIKRLAVLIPIIFLIYMIAVTGNAVLVFLILGDSW

LLMPMYFLLSQLSLNDLALISTVVPNMVTSFFTGKKNILQTG

>ModoOR2.5.24P

MERGNATSTTDFILLGLFPGMKHIDFIISVIFLIYIFAITGNVILIFLIW

ADSHLHTPMYILLSQLSVVDMAFISSVVPKMVIDFFSGKKDISHLGCGSQ

MFFSLMLGGAEGTLITLMAYDRYVAVCNPLHYVVIMNHNIYVQMVVGSWI

GGTLNSLVQTIYSMYYRFCNSKEINYFLCEVMAVLKLSCEDTSACEMG/L

IPLGFIMTSYILIFLSVLHMNFPEGRNKALVTCSFHLTVISLYFGPGIFI

YMMPRSAQILKINQVLSILYATLTPMLNPLIYSLRNKDMVGSLRKVLERF

LFSK

>ModoOR2.5.25P

MFMMGNQTTVIEFFLVGLRNDPEIQVILFIFLLLIYLLSVTGNITIIILT

LLDSQLQMSIYFFLQNF

>ModoOR2.5.26

MLIMGNQTAVTEFFLVGLTDDPEIQVILFIFLLLTYLLSFTGNMTIIILT

LLDSQLQTPMYFFLRNFSFLEISFTTVFVPKMLVNIGTGNKTISFPGCFT

QYFFAILLGATEFYLLAAMSYDRYVAICKPLHYTMMMNKKLCLQLVLSSW

FSGFMVVIGPHIMTTMLPFCASNVINHYCCDYTILLQLACADTHIIEMIE

FVLAVVTLLFTLVLVIISYVYIMGTIMRIPSAQKRKKAFSTCSSHMIVVS

LSYGSCIFMYVNPSMKEAATFNKGVAVLNTSVAPLLNPFIYTLRNQQVKR

AFKDMVRRIKFFSVK*

>ModoOR2.6.1

MGNLTVVTGFLLMGFSNTSDLQILHAMFFLLIYLVALIGNLLIFTLISLD

GCLHTPMYFFLKNLSFLDLCLISVTVPKSIANSLSHSCSISYLGCVLQLF

LVILFAGSEISILTVMSYDRYIAICQPLHYETIMNKESCVKMAVASWFSG

GVVGTMYSASTFSLPFCGSREIHQFFCDVPSLLRISCSEKHMVVYVSVFI

GLGLVIFCCISIIISYGHIFSTVLKIPTTQGRSKAFSTCLPHLIVFIVFV

ITGSMAYLKPHLDSDSVLDLLLSMFYTVVPPTLNPVIYGLRNKDMKTSIT

KFIAWKHSSERLMKLSFP*

>ModoOR2.6.2

MSNLTSMTEFLLMGFSNTWELQVVHATFFLMIYLMALIGNLIIFTIISLD

VHLHTPMYFFLKNLSFLDLCLISVTVPKSIVNSLSHNSSISYLGCVLQLF

LVILFAGSEISILTVMSYDRYVAICQPLHYETIMIKGSCVKMAVASWFSG

GVVGTMYSASTFSLPFCGSREIHQFFCDVPSLLRISCSEKHIVVYVSVAI

GLGLGIFCCIYIITSYVHIFSTVLKIPTTQGRSKAFSTCLPHLIVFIVFI

ITGAMAYLKPPFDSNTVLDLLLSMFYTVVPPTLNPVIYSLRNKDMKTSIN

KLIAWKHSSEGLMKFFP*

>ModoOR2.6.3

MNNLTMVTEFLLMSFSKTWELQILQAILFLLIYLVALMGNLLIFTLISLD

GHLHTPMYFFLKNLSFLDLCLISTTVPKSIANSMSHSHSISLLECILQLF

LVIFFASSELFLLTVMSYDRYVAICRPLNYEVVMNKGTCVKMAAFSWLTG

GLFGVLYTISTFSLSFCNSNEIHQFFCDVPSLLRISCSDTHIALDVTIAM

GFSLGILCCISITISYGPIFSTVLKIPTTEGRSKAFSTCLPHLFVLLIFI

TTAALSYLSPPLDTNSVLDLLLSMFYMVVPPTLNPIIYSLRNKDVKTSLK

KLIA*

>ModoOR2.6.4

MANLTLVTGFLLLGFAKSWELQVLHAILFLLIYLMSLMGNLVIFTLISLD

EHLHSPMYFFLKNLSFLDLCLISTTLPKSITNSLSNSRSISFMGCVLQFF

LVILFSGSELFLLSVMSYDRYVAICQPLHYEVIMTRGLCVKMAAVSWFFG

IVFGTLYSATTFTLPFCHSKEIHQFFCDVPSLLRLSCSDVHIGVDVTMAI

AAAIGILCFISITISYGPIFSTVLKIPTTEGRSKAFSTCIPHLTVLMVFI

TTAVIVYLKPPQQSDSILDLLLSMFYAVVPPTLNPVIYSLRNKDMKTSLR

KLIAGKHIS*

>ModoOR2.6.5

MANLTLVTEFLLMGFSKSWELQVLHAILFLLIYLMSLMGNLVIFTLISLD

EHLHSPMYFFLKNLSFLDLCFISTTLPKSITNSLSNSRSISFMGCVLQFF

LVILFAASELFLLTVMSYDRYVAICQPLHYEVIMTRRLCVKMAAVSWFFG

IVFGTLYSATTFTLPFCHSKIHQFFCDVPSLLRLSCSDVHIGVDVTLAMG

VAYGILCFISITISYGPIFSTVLKIPTTEGRSKAFSTCIPHLIVLMVFIT

TSVIVYLKPSQQSDSILDVLLSMFYAVVPPTLNPVIYSLRNKDMKTSLRK

LIARKHIP*

>ModoOR2.6.6P

MQKVKIILYH*RMANLTLVTGFLLKGFSDIWELQILHAMFFLLIYLVILI

ENLPIFTIISLDRNLHSPMCFFLKNLSFLDLCLIYIRVPKSIANSLSHNC

SISYLGCVSQLFLMILFAIMELFLLTAMSYD/HVAICQPLNYEVIMNSET

SVKMAATSWLRGGLFGVLYSASIFTLPFCDSKEIHQFFCDIPSLLRISCS

DSHLAVDVTLSLAFSLGILSSIYIIISYCYIFSTVLKIPTTEGRSKAFST

CLPHLIVLIVFVTTGSISYLKPSQDSDSILELLLAMFYTVVPPTLNPVIY

SLRNKDMKTALRKLITWKYFSKGLILKSFP

>ModoOR2.6.7

MANLTLVTGFLLMGFSKSWELQVLHAILFLLIYLMSLMGNLVIFTLISLD

EHLHSPMYFFLKNLSFLDLCFISTTLPKSITNSLSNSRSISFMGCVLQLF

LVILFGASELFLLTVMSYDRYVAICLPLHYEVIMTRGLCVKMAAVPWFVG

ILFGTLYSATTFTLPFCHSKEIHQFFCDVPSLLRLSCSDVHIGVDVTLAM

GVAYGILCFISITISYGPIFSTVLKIPTTEGRSKAFSTCIPHLTVLMVFI

TTAVIVYLKPPQQSDSVIDLLLSMFYAVVPPTLNPVIYSLRNKDMKTSLR

KLITRKHIS*

>ModoOR2.6.8

MANLTLVTGFLLMGFSKSWELQVLHAILFLLIYLMSLMGNLVIFTLISLD

EHLHSPMYFFLKNLSFLDLCLISTTLPKSITNSLSNSRSISFMGCVLQLF

LVILFGASELFLLTVMSYDRYVAICLPLHYEVIMTRGLCVKMAAVSWFFG

ILFGTLYSASTFTLPFCHSKEVHQFFCDVPSLLRLSCSDVHIGIDVTLAI

AVPIGFLCFISITISYGPIFSTVLKFPTTAGRSKAFSTCIPHLTVLMVFI

TTGVIAYLKPPQESDSVIDLLLSMFYAVFPPTLNPVIYSLRNKDMKTSLR

KLLAQKHIS*

>ModoOR2.6.9

MANLTLVTGFLLMGFSKSWELQVLHAILFLLIYLMSLMGNLVIFTLISLD

EHLHSPMYFFLKNLSFLDLCFISTTLPKSITNSLSNSRSISFIGCVLQLF

FVILFGASELFLLTVMSYDRYVAICQPLHYEVIMTRGLCVKMAAVPWFIG

ILFGTMYSASTFTLPFCNSKEIHQFFCDVPSLLRLSCSGVHIGVDVTLAM

GVAYGILCCISITISYGPIFSTVLKIPTPEGRSKAFSTCIPHLTVLMVFI

TTILIDYLKPPQESDSVIDLLLSMFYAVVPPTLNPVIYSLRNKDMKTSLR

KLIARKHIS*

>ModoOR2.6.10

MQTVKIILYQRMANLTIVTGFLLKDFSDIWELQILHAMLFLLIYLVTLIG

NFLIFTIISLERKLHTPMYFFLKNLSFLDLCLISITVPKSIANSLSHNCS

ISFLGCVSQLFLMILFAITELFLLTAMSYDRYVAICQPLNYEVIMNSETC

VKMAATSWLSGVLFGVLYSASIFTLPFCHSKEIHQFFCDVPSLLRISCSD

SHLAVDVTISLAFSLAVLSSIYIIISYSYIFSTVLKIPTTEGRSKAFSTC

LPHLIVLIVFVTTGSISYLKPSQDSDSVLELLLAMFYTVVPPTLNPVIYS

LKNKDMKTALRKFITWKYFSKGLILKSFP*

>ModoOR2.6.11

MANLTLVTGFLLMGFSKSWELQVLHAILFLLIYLMSLMGNLVIFTLISLD

EHLHSPMYFFLKNLSFLDLCFISTTLPKSITNSLSNSHSISFIGCVLQFF

LVILFAASELFLLTVMSYDRYVAICQPLHYEVIMTRGLCVKMAAVSWFFG

IVFGTLYSATTFTLPFCHSKIHQFFCDVPSLLRLSCSDVHIGVDVTLAVG

VAIGILCFISITISYGPIFSTVLKIPTTEGRSKAFSTCIPHLTVLMVFIT

TSVIDYLKPSQQSDSILDLLLSMFYAVVPPTLNPVIYSLRNKDMKTSLRK

LIARNHIP*

>ModoOR2.6.12

MANLTIVTGFLLKGFSDIWELQILHAMLFLLIYLVTLIGNLLIFTSISLE

RKLHTPMYFFLKNLSFLDLCLISITVPKSIANSLSHNCSISFLGCVSQLF

LLVLFAVTELFLLTAMSYDRYVAICQPLNYEVIVNSETCVKMAATSWLSG

VLFGVLYSASIFTLPFCDSKEIHQFFCDVPSLLRISCSDSHLAVNITISL

AFSLGILFSIYIIISYSYIFSTVLKIPTTEGRSKAFSTCLPHLIVLIVFI

TTSSISYLKPSQDSDSLLELLLAMFYTVVPPTLNPVIYSLRNKDMKTALR

KLITWKYFSKGLILKSFP*

>ModoOR2.6.13P

ISVLFPSQCPNQLQTLSHNCSISFLGCV*QLFLLVLFAVTELFLLTAMFY

DRYVAICQPLNYEVIMNSETCVKMVASSWLSEVFFGVLYSASIFTLPFCH

SKEIHQFFCDIPSLHRISCSDSHLAVDVTINLAFSLAVLSSIYIIISYSY

IFSTVLKIPTTEGRSKAFSTCLPHLIVLIVFITTSSISYLKPSQDSDSLL

ELLLAMFYTVVPPTLNPVIYSLRNKDMKTALRKLITWKYFSKGLILKSFP

>ModoOR2.6.14

MKNQSMEIEFMLVGISSLPELQLFLLVSFLCIYVTALVGNSLIFFTICAS

QQLHTPMYFFLGNLSVIDVLCTSTIMPKMLANLYFQRADISFLGCMAQMY

LFTWALVSEVLLLALMAFDRYVAICHPLHYSMVMRSHVCIGMAGSIWAIG

ICNSAVHTSLAIPLSFCSSFIIDHFFCELPPILKLSCSDTSLNEALAFCA

DVIFGVGSCSLILVSYGCIIRTILKIRSSEGKKKAFSTCSSHLTVVSFYY

STVIYTYIRPTSNLSLGRDKVITALYSVIIPMLNPMIYSFRNREVKGALR

RLLGWTLFFPLGRTL*

>ModoOR2.6.15P

KESVLLDLYSISITVPK*IANSLSNNISISFLEYVSQLFLILFAASELFL

LTVISYD*YVAIHQPMHYEIIMNSETWIKMATTSWLSGVCLGPCTQYIFF

AILWLQEDSPVLL*CLFLTHSLLL*FTS*N*CDYCFWE*FRDSVLYLYHS

LLQSHLSKCAEDSDHRI*VKCFPICQSYLIILMFFVTTGLITFLKPSQES

YSVLDLLLSMFYTIVPLILNPVTYSLRNKVLNSDFSKEIHSLKILITSFP

>ModoOR2.6.16

MANFTMTTGFLLMGFSDIWELQILHAMFFLLIYLVALIGNLLIFTLISLD

RKLHTPMYFFLKNLSFLDLCLISITVPKSIANSLSHNCSISFLGCVSQLF

LMMLFAASELFLLTAMSYDRYVAICLPLHYEVLMNGEICMKMAAASWLSG

GFFGALYSVTTFTLPFCGSKEIHQFFCDVPSLLRISCSHSHLAVDVTMTF

GFGLGIFCSICITVSYSHIVSTVLKIPTTEGRSKAFSTCLPHLFILMVFI

TTGIIAYLKPPQESDSVIDLLLSLFYTVVPPTMNPIIYSLRNKDMKAALR

KLIAWKYFSKELIPKAFP*

>ModoOR2.7.1

MASPPWENQTVIVEFVLLGFSTIWQLNIALFTTFLASYIFTVTGNGLIVL

LVVLDHHLHIPMYFFLVNLSFLEIGYTSNIIPRMLASILAGRKTISVASC

LTQFYFFGSLAATECLLLAAMSYDRYVAICQPLRYTVLMNGQVCVGLAAS

SWVCCFLLTALTIILLSRLTFCGPNEIDHFFCDFTPLVQLSCTDTSLMET

IAFATSSAVTLVPFLLITASYSCILTTILRIPSGAGRRKAFSTCSSHFTV

VTMFYGTLIATYLAPSAHSSQFLRKVFSLLYTILTPMFNPIIYSLRNRDI

HEALKRYLSRKMVLFQ*

>ModoOR2.8.1

MEFIFLGFSNHPDLQGLFFLLFLVIYLITILGNTLILIAIRVNPILHTPM

YYFLSNLSFLDICYTSTTVPIILVNLLQEKKTITYEGCLSQIFFLVTFAG

SECVLLAAMAYDRFVAICHPLRYPVLMSKRVCAYLAAGSWLCGLVNSMTH

TGLTATVTLCGPNQISHFLCDIPLILKLSCSDTSVNEFALYVSSATIGLS

PCLFTAVSYMLIISAILKIQSAQGRHKAFSTCASHLTVVVIYFTTINFNY

DRPSTGYSLDVDILVSVLFCIVTPMLNPIIYSLRNKDVKVALKKLFEGYM

SSSGSSF*

>ModoOR2.8.2

MKDIKEKGNQTLVTEFIFLGFSNHPKLQGLFFMIFLLVYLVTLLGNLLIL

TAIRINPALHTPMYYFLINLSFLDICYTSTTVPIMLVNFFREKKTITYEG

CLSQLFFLVTFAGSECVLLAAMAYDRFIAICHPLRYPVLMSKKVCSYLAA

GSWLCGLVNSMTHTGLTATVTLCGPNQISHFLCDIPLILKLSCSDTSVNE

FTLYVASATIGLSPCLFTAVSYMLIISAILKIQSTQGRHKAFSTCASHLT

VVVIFYGTANFNYDRPSSGYSLDMDILVSVLFCIVTPMLNPIIYSLRNKE

VTVALKKLCELYILPGLVGGPMDRVRGQESSS*

>ModoOR2.8.3

MKDIKERGNQTLVTEFIFLGFSNHPELRGLFFLIFLLIYLVTLLGNLLIL

TAIRINPALHTPMYYFLSNLSFLDICYTSTTVPIMLVNFFREKKTIAYEG

CLSQLFFLVTFAGSECVLLAAMAYDRFIAICHPLRYPVLMSKKVCAYLAA

GSLLCGLVNSMTHTGLTAILTFCGPNQISHFLCDIPLLLKLSCSDTSVNE

LALYVASATIGLSPCLFTAVSYMLIISAILKIQSAQGRHKAFSTCASHLT

VVIIYYGTGNFNYDQPSSGYSLDVDILVSVLFCIVTPMLNPLIYSLRNKE

VKVALRKLCEGYILSRDSFVQISL*

>ModoOR2.8.4

MKDIKERGNQTLVTEFIFLGFSNHPELRGLFFLIFLLIYLVTLLGNLLIL

TAIRINPALHTPMYYFLSNLSFLDICYTSTTVPIMLVNFFREKKTIAYEG

CLSQLFFLVTFAGSECVLLAAMAYDGFIAICHPLRYPVLMSKKVCAYLAA

GSLLCGLVNSMIQTGLTAILTFCGPNQISHFLCDIPLLLKLSCSDTSVNE

FALYVSSATIALSPCLFIIVSYMFIISAILKIQSTQGRHKAFSTCASHFT

VVIIFYGTGTFNYDQPSSDYSLDVDVLVSVLFCIFTPMLNPLIYSLRNKE

VKVALRKLCEGYILSRDSFVQISF*

>ModoOR2.8.5

MKDIKERGNQTLVTEFIFLGFSNHPKLQGLFFMIFLLVYLITLLGNLLLL

SAIRINPALHTPMYYFLSNLSFLDICYTSTTIPIMLVNFFREKKTITYEG

CLSQLFFLVTFAGSEGVLLAAMAYDRFIAICHPLRYLVLMSKKVCACLAA

GSWLCGLVNSMIHTGLTATVTLCGPNQINHFLCDIPLILKLSCSDTSVNE

FALYVASATIGLSPCLFTAVSYMLIISAILKIQSAQGRYKAFSTCASHFT

VVVIFFGTANFNYDQPSSGYSLDMDILVSVLFCIVTPMLNPLIYSLRNKE

VKVALRKLCEKYILHRGFSVQISS*

>ModoOR2.8.6P

VKYVKE*RNQTVVTEFVFWGFPVTPNYRDCSS*YFYLYTW*CFWIIFL*K

QQSESILFFILL*IISCYFSFF*THEFGVLCYRTDSLIFTDFFFCYISNT

IPIMLVNFF*EKKTIAYEDCLSQIFFLVTFA/SSLLLLQTMAHDRFIAIC

HPLCYPVLISKRICAYSAAESWMCGLVSSVAHSGLTKTLTFSGSN*ISHF

LCDIPLILKLSCSDTSVNEFILYVASATIGLSPCLFTAVSYMLIISAILK

IQSAQGRYKAFSTCASHFTVVVIFFGTANFNYDRPNSGYSLDMDILVSVL

LYIVIPMLRPIIH*LRNRE

>ModoOR2.8.7

MFQYNFPSHLSSDTREKNQTITEFIFLGFSNHPDMQGLFFLVFLVIYLIT

ILGNTLIITAVVVNPVLHTPMYYFLSNLSFVDICYTSTTVPTMLVNFFQE

KKTITYEGCLSQLFFLVTCAGTEGVLLAAMAYDRFAAICHPLRYPVLMSR

RVCAYLAAGSCLCGLVNSLVHTGLTATLVLCSSNEISHFLCDIPQLLKLS

CSDTSVNEIALYVASVFIDLSPCLFIAVSYMLIISAILKIQSAQGRHKAF

STCASHLTVVIIFFGTALFNYDRPSSDYSLDVSILVSSLFCIVTPMLNPI

IYSLRNKEIKGAIKKLAGQWRLPGF*

>ModoOR3.1.1P

ADCFVQMYFFMVFVGLDNFFLTVMAYDHFVAICHPLWYAIIMSHRLLSCW

FCSPGY*AFSILSFTV*WSPFVTMKFSTPFVILNRF*NSLVLTPLSITS*

FTV*LYCWVLSPSLGSFSHTVRSLRPF*KSHLLRDKFKAFSTCGSHLSVV

SLLYGTGLGVYFSSSATHSSWKSTVASTMYTVVTPMLNPFIYSLRNKDIK

DDLMRLISRITSSQ

>ModoOR3.1.2

MVPGNQTQFTEFLLLGFSETPEQQGPLFGLFLGMYLVTVVGNLLLMLTIA

SDSHLHTPMYFFVSNLSFVDLCVITTTVPKMLVNILTQSKAISYAACVAQ

MYFFMALTCVDNFLLTAMAYDRFVAICQPLHYATIMSPRLCVLLMMLSWI

ISLVEALIQNLMVMRLSFCGDHEIQHFFCDFPQVLKLSCSDTFNNYMLLY

IMAAILGIVPLTGIVFSYSQICSTILKVPSAKGKYKAFSTCGSHLSVISL

FYGTGMGVYFSSSITHSSWKSTVASAMYAVVTPMLNPFIYSLRNKDIKDA

VRRLIGRVTSFE*

>ModoOR3.1.3

MVPGNQTQFTEFLLLGFSETPEHQGPLFGLFLVMYLVTVVGNLLLMLIVT

SDSHLHTPMYFFVSNLSFVDLCIITTTVPNMLVNFLKRSKVIPYAGCLAQ

LYFFMAFGSLDNFLLTAMAYDRFVAICHPLHYATIMSPRLCVLLVLLSWI

ISLVQSLIQNLMVTRLSFCRDHEIQHFFCDFPQILKLSCSDTFINYMLLY

ITTGLLGIVPLTGIVFSYSQIFSSILKVPSAKGKYKAFSTCGSHLSVVSL

FYGTGIGVYLSSSTTQSSWKSTVASAMYAVIIPMLNPFIYSLRNKDIKDA

LRKIISKGTSFE*

>ModoOR3.1.4

MAPGNQTKFTEFILLGFSETPEQQRTIFGLFLGMYLVTVFGNILIILAVG

SDSHLHTPMYFFLSNLSFVDLCVVSSTVPKMLVSILTQNKAISYAGCLAQ

MSLFMFFGCLDNFLLTAMAYDRFVAICHPLRYTSIMSPELCCLMVLLSWI

LSLLNTLLHSLMVTRLSFCTGHEIQHFFCDLEKVLKLSCSDTLINYVLLY

VIAGLLGVLPLTGILFSYSQIFSSILKVPSARGKYKAFSTCGSHLSVVSL

FYGTGLGVCLSSSVTQSSWKSTVASAMYAVVTPMLNPFIYCLRNKDIKGA

LWRLISRISSSQ*

>ModoOR3.1.5P

MVPGNQTQFIEFLLLGFSRAPEQQRYLFGLFLSMYLAAILGNLLLMPTNT

SDSHLDTPMYFFVSHLSFVDLCIVTTTVPKTLVNILTQSKIISYAGCLLH

LCFFMSLNLCGQF/VDSFLLPAMVYDYFVAICHPLHYTIIMRPILCVLLV

LLSWIIGLVESLIQNLMVTRLFC*DREIQHLFCEFH*IMKLSCSDIFINY

MALYIVTAILGIVLFTGIIFSCSKICSSIVKIPTAMGKYKAFSTCGSPL/

LSVVSLFYGTGLGVYFSSSTTHYSWKSTIASAIYAVVTPILNPYIYSLRN

NDIKDALRRLIVRVTNW

>ModoOR3.1.6P

MAPENQTKFTEFILLGFSETPEQQGAIFGLFLGMYLVTVFGNILIMLAIG

FDSHLHTPMYFFLSNLSFVDLCVVSTTVPKMLVGILTQNKAISYAGCLAQ

MHFFIVFVGLDNFLLTAMAYDCFVAICHPLRYTSIMSPELCCLLVLLSWI

LSLLDALLHSLMVTRLSF*TGHEIQHFFCDLDQVLKLSSSDTLINYVLLY

ILAVLLEILPLTGILFSYSQIFSSILKVPSAGGKNKPFSTCGSHLSVVSL

FYGTALGVHFRSSVTQSSWKSTVASAMYAVATPMLNPFIYCLRNKDIKGA

LWRLISRICSSQ

>ModoOR3.1.7

MVPGNQTQFTEFLLLGFSETPEQQGPLFGLFLGMYLVTVVGNLLLMLTIA

SDSHLHTPMYFFVSNLSFVDLCVITTTVPKMFVNILTQSKAISYAACVAQ

MYFFMALICVDNFLLTAMAYDRFVAICQPLHYATIMSPRLCVLLMMLSWI

ISLVEALIQNLMVMRLSFCGDHEIQHFFCDFPQVLKLSCSDTFNNYMLLY

IMAAILGTVPLTGIVFSYSQICSTILKVPSAKGKYKSFSTCGSHLSVISL

FYGTGMGVYFSSSITHSSWKSTVASAMYAVVTPMLNPFIYSLRNKDIKDA

VRRLIGRVTSFE*

>ModoOR3.1.8

MVPGNQTQFTEFLLLGFSETPEHQGPLFGLFLVMYLVTVVGNLLLMLIVT

SDSHLHTPMYFFVSNLSFVDLCIITTTVPNMLVNFLKQSKVIPYAGCLAQ

LYFFTAFGSLDNFLLTAMAYDRFVAICHPLHYATIMSPRLCVLLVLLSWI

ISLVQSLIQNLMVTRLSFCRDHEIQHFFCDFPQILKLSCSDTFINYMVLY

ITTGLLGIVPLTGIVFSYSQIFSSILKVPSAKGKYKAFSTCGSHLSVVSL

FYGTGIGVYLSSSTTQSSWKSTVASAMYAVIIPMLNPFIYSLRNKDIKDA

LRKIISKGTSFE*

>ModoOR3.1.9

MAPGNQTKFTEFILLGFSETPEQQRTIFGLFLGMYLVTVFGNILIMLAVG

SDSHLHTPMYFFLSNLSFVDLCVVSTTVPKMLVSILTQNKAISYAGCLAQ

MSLFMFFGGLDNFLLTAMAYDRFVAICHPLRYTSIMSPELCCLMVLLSWI

LSLLNTLLHSLMVTRLSFCTVHEIQHFYCDLDKILKLSSSDTLINYVLLY

IVAGLLGVLPLTGILFSYSQIFSSILKVPSAGGKYKAFSTCGSHLSVVSL

FYGTGLGVCLSSSVTQSSWKSTVASAMYAVVTPMLNPFIYCLRNKDIKGA

LWRLISRISSSP*

>ModoOR3.1.10P

NQTQFIEFLLLGFSRAPEQQRYLFGLFLSMYLAAILGNLLLMPTNTSDSH

LDTPMYFFVSHLSFVDHCIVTTTVPKTLVNILTQSKIISYAGCLLHLCFF

MSLNLCGQF/VDSFLLLAMVYDYFVAICHPLHYTIIMRFLLCVLLVLLSW

IIGLVESLIQNLMVTRLFC*DREIQHLFCEFP*IMKLSCSDIFINYMTLY

IVTAILGIVPFTGIIFSCSKICSSIVKIPTAMGKYKAFSTCGSPL/LSVV

SLFYGTGLGVYFSSSTTHYSWKSTIASAIYAVVTPILNPYIYSLRNNDIK

DALRRLIVRVTNW

>ModoOR3.1.11

MAPGNQTKFIEFILLGFSETPEQQGTIFGLFLGMYLVTVFGNILIMLAVG

FDSHLHTPMYFFLSNLSFVDLCVVSTTVPKMLVGILTQNKAISYAGCLAQ

MYFFIVFVGLDNFLLTAMAYDRFVAICHPLRYTYIMSPELCCLMVLLSWI

LSLLNTLLHSLMVTRLSFCTGHEIQHFFCDLDQVLKLSCSDTLINYVLLY

ILAGLLGILPLTGILFSYSQIFSSILKVPSAGGKYKAFSTCGSHLSVVSL

FYGTAMGVYFSSSVTQSSWKSTVASAMYAVVTPMLNPFIYCLRNKDIKGA

LWRLISRICSSQ*

>ModoOR3.1.12

MASENQTKFTELILLGFSEIPEQQGAIFGLFLGMYLVTVFGNILIMLAVS

FDFHLHTPMYFFISNLSFVDLCVVSTTVPKMLVGILTQNKAISYAGCLAQ

MYFFIVFAGLDNFLLTAMAYDRFVAICHPLRYTSIMSPELCCLLVLISWT

LTLLNALIHSLMMTRLSFCTGHEIQHFFCDLDQVLKLSCSDTLINYVLIY

IVAGLLGIFPLTGILFSYSQIFSSILKVPSAGGKYKAFSTCGSHLSVVFL

LYGTELGVYFSSSVTHSSWKSTVASAMYAVVTPMLNPFIYCLRNNDIKGA

LWRLISRISSSQ*

>ModoOR3.1.13

MAPGNQTKFTEFILLGFSETPEQQGAIFGLFLGMYLVTVFGNILIILAFG

SDSHLHTPMYFFLSNLSFVDLCMVSTTVPKMLVGILTQNKAISYAGCLAQ

MYFFIVFAGLDNFLLTAMAYDRFVAICHPLHYTSIMSPELCCLLVLLSWI

LSLLDALLHSLMVTRLSFCTGHEIQHFFCDLDQVLKLSSSDTLINYVLIY

IVVSLLDILPLTGILFSYSQIFSSILNVPSSAGKYKAFSTCGSHLSVVSL

FYGTALGVYFSSSVTQSSWKSTVASAMYAVVTPMLNPFIYCLRNKDIKGA

LWRLISRISSSQ*

>ModoOR3.1.14

MVPGNQTQFTGFILLGFSETPEEQGPLFGLFLGMYLVTVLGNLFIMLAIG

SDSHLHTPMYFFLSNLSFVDLCVVSTTVPKMLVNILTQNKAIPYADCFAQ

MYFFIIFASLDNFLLTVMAYDRFVAICHPLHYAAIMSPRLCVLLVLLSWI

LSLLDSLLHCLMVTRLSFCAHKIQHFFCDLDQVMKLSCAGTFINYVLVYL

AIGLLGIIPLTGILFSYSQICSSILKVPSSGGKYKAFSTCGSHLSVVSLF

YGTGLGVYLSSSTTHSSWKSTIASVMYAVVTPMLNPFIYSLRNKDIKNAL

RRLISKIISSRS*

>ModoOR3.1.15

MGQDNHTSVSEFLLLGLSEKPEQQLLLFGIFFTMYMVTMIGNLLIILAIY

SDSHLHTPMYFFLSNLSFVDLCQISTTMPKMLINILTHNKAIPYAGCLVQ

MYSFHLFGTMDSFLLAVMAYDRFVAICHPLRYATIMSPRLCVLLVGGPWG

ITNLQSVVHTSLMAKLTFCADNKIPHFFCDLMPLLKLSCSDTHINELVIL

IFGIFMGISPLVCILLSYICIFCAVLRVPSAKGKWKAFSTCGSHLTVVLL

FYGTIFAVYLQPSGPTSPQKDKAAAVMCAVVIPMLNPFIYSLRNRDMKSA

LRKLISRTSSQ*

>ModoOR3.1.16

MKLGNETWIVKEFVLVGFSNFPDLKPTLFSLFLLMYLITLSGNITIITII

YLDHTLHTPMYCFLGVLSLSETCYTLVTIPNMLVHLLMENQVISISSCRA

QMFFFLGLGCSNCFLLTLMGYDRYVAICHPLRYPMIMSPSVCIRLGALVF

CSGFLVAIIETCLIFSSSFCNDNRVVHFFCDIAPVLKLSCTQSIAKALAI

FFLSVIVVLFSFLLILLSYAFIVAAILRIPSAEGRRKAFSTCVSHLTVVI

VHFGCASIIYLRPESGSNPEKDLMVAVFYTVVTPLLNPVVYTLRNKEVRV

ALKRTLGRRLRTQSL*

>ModoOR3.1.17

MKLGNETWIVKEFVLVGFSNFPDLKPTLFSLFLLMYLITLSGNITIITII

YLDHTLHTPMYCFLGVLSLSETCYTLVTIPNMLVHLLMENQVISISSCRA

QMFFFLGLGCSHCFLLTLMGYDRYVAICHPLRYPMIMNPSVCIRLGALVF

CSGFLGGHHETALIFSSSFCNDNRVVHFFCDIAPVLKLSCTQSIAKALAI

FFLSVIVVLVSFLLILLSYAFIVAAILRIPSAEGRRKAFSTCASHLTVVI

VHFGCASIIYLRPESGSNPEKDRMVAVFYTVVTPLLNPVVYTLRNKEVRV

ALKRTLGRRLRTQSL*

>ModoOR3.2.1

MFNSGIASMLGQNHTMVTEFILIGFSTFPQLQLMFFVILLMMYLFTLLGN

LLIMLTIWNERSLHTPMYFFLCALSISEIFYTFAIIPRMLIDLVSTHHNI

SFLACANQMFFSFTFGFTHSFLLTVMGYDRYVAICHPLRYNVLMNPQGCA

WLVASAWLGGIVMGLVVTLAIFHLTFCGPNKIHHFFCHVPPLVKLACGDV

SIVAMGVGMVCITALLGCFLLILLSYAFIVATVLRIPSAEGQHKAFSTCA

SHLTVVVVHYGFASVIYLKPKALESLEGDTLMGITYTALTPFLSPIIFSL

RNKELKNALKKVYLSKLCPLNL*

>ModoOR3.2.2

MLGPNHTSVSEFILIGFSTFPHLQLMFFVLFLLMYLFTLLGNLLIMMTIR

NERSLHTPMYFFLCALSISEIFYTFAIIPRLLTDLLSTHHAISFLGCANQ

MFFSFTFGFTHSFLLTVMGYDRYVAICHPLRYNVLMSSRGCTWLVASSWL

GGMVMGLVVTLAIFHLYFCGPNEIHHFACHVPPLVKLACGDIWAVAMGVG

MVCITALLGCFLLILISYAFIVATILRIPSAEGRHKAFSTCASHLTVVVV

HYGFASVIYLKPKTPESLEGDTLMGITYTILTPFLSPIIFSLRNKELKNA

INKVFFSNFCPQKL*

>ModoOR3.2.3P

VLQFSLPGTI*QPEQEQFLFRLFLGIYFIGTLGNLLIILAIVFDSHLHTP

MYFFLSNLSFLDLCFTSTTVPKMLVNYLLGSNAISQPECLAQMYFFVAFG

ASDSILLSAMAYDRYLAVCHPLHYGMIMTSHLCALLVAVPWVSGHLISMI

HTILLAQLSFCTNNKIPHFFCDMNTLIKLSCSDTYINEILVLLLGGMVVL

IPFICIVISYTPIVSAILKVSSSQGKWKAFSTCGSHLSVVSLFYGTIISV

YFNPANTHTVQKDMVATVMYTVVTPTLNPFIYSLRNDDLKRAFWKLLGLT

CFSTR

>ModoOR3.2.4

MRNQTSVLEFLLLGLSEHPEEQQFFFRLFLGIYLIGTLGNLLTILAIGFD

SHLYTPMYFFLSNLSFLDLCFTTTTVPKMLVNYLSGSNAISYSECLAQMY

FINAFGASDSILLSAMAYDRYLAVCHPLHYGTIMTSRLCALLVAVPWISA

HLISMVHTIFIGHLSFCMNNKIPHFFCDINTLIRLSCSDTHINEMLVLFL

GGPVVLIPFICIVVSYTPIVSAVWKVPSVQGKWKAFSTCGSHLSVVTLFY

GTVIGVYFNPTSTHTTQKDMIATVMYTVVTPTLNPFIYSLRNNDLKGALG

KLLGLKHFSKR*

>ModoOR3.2.5

MLGPNQTTVSEFILIGFSPFPQLQLLFFVLFLLMYLFTLLGNLLIMLTVW

HERNLHKPMYFFLCTLSISEIAYTLAINPRMLADLISTHHTISLWGCANQ

MFFTFSCGLAHAFLLTIMGYDRYVAICHPLRYGVLMSSQGCAWLVASSWL

SGIVMGLVITLPIFSLTFCGPNEIHHFFCQVPPLVKLACGDVSAVALGIG

VVYIIVLLVCFLLILLSYIFIVATILKIPSAEGRHKAFSTCASHLIVVVI

HYSFASVAHLKAKALEALEGDTLMGISYSVLTPFLSPIIFSLRNKELKDA

LKKVFLRNLCPSRL*

>ModoOR3.2.6P

SGKAYMLGQNDNTVAKFLFIGFSIITQLQEVFFVLFLWMYLFILMGNLKL

TIQSILPMYFFLCALSITEIYTFVIIHSPTYSPLMMRSPSWAMPTRCSSP

SHLVSHTPTCSLL*AKIAHVSM*DVQDVNIFHTLYYNIVYKPTKLYLVEA

SSCMVLGLVVTLAIFHLHFCGHNEIYHFACYAFSLMKLAYGYVWAMTMEV

GMVSIIIILGCFLLILFSYAFIVATILRSSLAEGQHKVFST/SLVILHYG

FVSVICLKPKVPESLEGDTLIVTPFLSPIIFSLRNKELKNLINKL

>ModoOR3.2.7

MVAGNQSEISEFILLGLSDQPEQQSLLFFLFLLIYMITGVGNLLIIVVIR

TNSCFHTPMYFFLSNLSLVDICFTTTTIPKMLVNHISGNKAISYAGCLTQ

VFFFIWFAGIDSVLLTVMAYDRYVAICAPLHYSMIMTPKVCLLLVVVSWF

WAYITALIHTVLVTRLSFCGQNEIPHFFCDLSPLLKLACSDTFINDLMVN

TVGALIIIIPFIGILISYTRIFVTVMRIPSTVGKWRAFSTCGSHLTVVCL

FYGTVIGVYFSPISNHTAQQDTVAAVMYTVVTPMLNPFIYSLRNKDIKRA

LKMLLTGKPVLSL*

>ModoOR3.2.8

MVAGNQSEVSEFILLGLSNQPEQQSFLFFLFLLIYMITGVGNLLIIVVIR

TNSCFHTPMYFFLSNLSLVDICFTTTTIPKMLVNHISGNKTISYAGCLTQ

VFFFIWFAGIDSVLLTVMAYDRYVAICAPLHYSMVMTPKVCLLLVVVSWF

WSYINALIHTVLVTRLSFCGQNEIPHFFCDLSPLLKLACSDTFINDLMVN

TVGALTIIIPFIGILISYTRIFVTVMRIPSTVGKWRAFSTCGSHLTVVCL

FYGTVIGVYFSPISNHTDQQDTIAAVMYTVVTPMLNPFIYSLRNKDMKGT

LKMLLTRKPVRSL*

>ModoOR3.2.9

MEGGNQSGVSEFILLGLSDQPEKQRLLFFLFLVMYLITGLGNLLIILAIS

TNSRLHTPMYFFLSNLSLVDICFTSTTIPKMLTNHVSGKKAIPYVECITQ

LFFFVLFGEIDSVLLTVMAYDRYVAICAPLQYTMLMTPKICALLVAMSWF

WAYINALTQTVLLTRLSFCGHNEIPHFFCDISSVLKLACSDTFINDVVLL

AVGGLTVIIPFIGILISYTRIFVTVMRIPSASGKWKAFSTCGSHLTVVCL

FYGTIIGVYFSPTSTHTAQQDTTAAIMYTVVTSMLNPFIYSLRNKDMKGA

LRVLFTRKPGLSL*

>ModoOR3.2.10

MEGGNWSEVSEFILLGLSYDPEQEKLLFFMFLVMYLITVLWNLLIILAIK

SDSRLHTPMYFFLSNLSLVDMCFTSTTVPQMLLSHISGNKAIPYFACLTQ

TFFFSWFAGVDSILLASMAYDRYVAICAPLHYSMIMTPRTCALLVSVSWF

WPCINALIHTISAAQLSFCGHTEIPHFFCDLNALIKLACSDIFINGLMVY

TVGTLTTIIPFIGILISYIHIFWAVVRIPSARGKQKAFSTCGSHLTVVSL

FYGTLIGVYFSPKSTHTAQQDTASAVMYTVVTPMLNPFIYSLRNNDMKGG

LKVLLTRTPELSL*

>ModoOR3.2.11

MKRENHSRVSEFILLGLSDQPEQDRLLFLIFLLMYLITGLGNLLIILVIR

IDPRLHTPMYFFLSNLSLVDICFTTTTIPKMLVNYISGNKEILYISCLAQ

VFFFVWFAGLDSILLASMAYDRYMAICAPLHYTMIMTPRLCVLLVAVCWF

GACANALTHTVLLTQLSFCGHKEIPHFFCDLGVVIRLACSDTFINDLVIY

TMGGLIAIIPFMGILISYFHIFVAILKIPSAYGKRKAFSTCGSHLTVVCL

FYGTMIGVYFNPTSTHTAQQDITSTVMYTAVTPMLNPFIYSLRNNDMKGA

LRMLLLRKSGFSL*

>ModoOR3.2.12

MKRENHSRVSEFILLGLSDQPEQDRLLFLIFLLMYLITGLGNLLIILAIR

IDPRLHTPMYFFLSNLSLVDICFTTTTIPKMLVNYISGNKEILYISCVTQ

AFFFLWFAGLDSILLGSMAYDRYMAICAPLHYTMIMTPRVYVLLVAVCWF

WACANALTHTVLLTQLSFCGHNEIPHFFCDLGVVIRLACSDTFINDLVIY

TMGGLTALIPFIGILISYFHIFVAVLKIPSAYGKRKAFSTCGSHLTVVCL

FYGTIIGVYFNPTSTHTAQQDIASTVMYTAVTPMLNPFIYSLRNNDMKGA

LRMLLLRKPGFSL*

>ModoOR3.2.13

MEQKNQSEVSEFILLGLSSKPEQERLLFFLFMLMYLTTVLGNLLIILAIR

IDSRLHTPMYFFLSNLSLGDICFTTTTIPKMLVNYISGNKEILYISCLAQ

AFFFSWFAGLDSILLGSMAYDRYMAICAPLHYTMIMTPRLCVLLVAVCWY

WACANALTHTVLLTQLSFCGHKEIPHFFCDLGVVIRIACSDTFINDLVIY

TMGGLIAIIPFMGILISYFHIFVAVLKIPSAYGKWKAFSTCGSHLTVVCL

FYGTIITVYFNPTSTHTVQQDIASAVMYTAVTPMLNPFIYSLRNNDIKGA

LKMLLLRKPGFSL*

>ModoOR3.2.14

MEQKNQSEVSEFILLGLSSKPEQERLLFFLFMLMYLTTVLGNLLIILAIR

IDSRLHTPMYFFLSNLSLGDICFTTTTIPKMLVSYISGNKEILYISCLAQ

LFFFNWFAGLDSILLASMAYDRYIAICAPLHYTMIMTPRVCVLLVAVSWF

WPCVNALTHTVLITQLSFCGHNEIPHFFCDLGVVIRIACSDTSINDLLIY

TMGGLTAIIPFMGILISYFHIFVAVLKIPSAYGKWKAFSTCGSHLTVVCL

FYGTIIGVYFSPTSTHTAQQDIASAVMYTAVTPMLNPFIYSLRNNDIKGA

LRMLLLRKPGFSSDFSHYTFTAIRKRFQ*

>ModoOR3.2.15P

MEQKNQSEVSEFILLGLSGQPEQERLLFFLFMLMYMITVLGNLLIILAIR

IDSRLHTPMYFFLSNLSLVDICFTSTTIPKMLVNHISGNKVI/GTK*YPY

GRCLTQTFFFSWLIGTDSVLLASMAYDRYVAICAPLHYTMIITPQVCALL

VAVSWIWTCINSLIHTTSLNQLSFCGHNEIHHFFCDLNALIKLACSDTFI

NDLLIFTVGGLTAVVPFIGILISYVHISVAVLRIPSASGKRKAFSTCGSH

LTVVCLFYGTIIGVYFSPTSTHTAQKDTAVAVMYTVVTPMLNPFIYSLRN

KDMKGALRILLTRKPGFSL

>ModoOR3.2.16

MDGRNHSAISEFILLGLSNQPEQERLLFLLFLVMYLITVLWNLLIILAIK

TDSHLHTPMYFFLSNLSFIDICFTSSTVPKMLVNHISGNKAISYSGCLTQ

VFFFSWFAGLDSIILASMAYDRYVAICAPLHYTMIMTPKFCAHLVAVCWF

WACIDALIHTVSLTRLSFCDHNEIPHFFCDLSVVIRLACSDTFFNDLMVY

IVGGLTAVMPFIGILISYTRIFVAVLRIPSAHGKQKAFNTCGSHLAVVCL

FYGTIIGVYFNPTSTHTAQKDTASAVMYTVVTPMLNPFIYSLRNNDMKGA

LKMLFTRKPGLSL*

>ModoOR3.2.17

MEGGNQSRISEFILLGLSDQPEEERLLFLAFLFMYLITGLGNLLIILAIR

TDSHLHTPMYFFLSNLSLVDICFTSTTIPKMLANHVSGNKMIPYSGCLTQ

VFFFIWFAGIDSFLLTAMAYDRYVAICAPLHYSTIMTPRICALLLAVSWF

SAYVNALTHTVLLTRLSFCGHNEIPHFFCDLSPLLKLACSDTFINDLMVY

TVGALPIITPFIGILISYTQIFMAVLRIPSAGGKRKAFSTCGSHLAVVSL

FYGTLIGVYFSPTSTHTVQQDTAAAVMYTVVTPMLNPFIYSLRNKDMKQA

LKMLLTRKPGLTL*

>ModoOR3.3.1

MGPGNQTCVSEFFLLGFSEKSEHQLPLFGLFLGMYIVTLFGNLLIMLAIG

SDSHLHTPMYFFLSNLSLVDLCLVSTTIPKMLANILIHSKAISYAGCLTQ

MYFFLIFACMDNFLLSVMAYDRFVAICHPLRYVAIMNPQLCGLLVLISWA

LSLLISLLHNLMVMHLSFCKDHEIPNFFCDLTQILKLSCSETLTNNILVY

FTTGLLGIVSVTGILTSYTQICSSILKIPSAGGKYKAFITCGSHLCVVSL

FYSTVFGVYMTSSATHSSWKSMVASVMYAVVTPMLNPFIYSLRNKDIKDA

LKKFISRSSSSQ*

>ModoOR3.3.2

MEPLNRTHSLEFLLLRFSEKLEQEMPLFGLFLSIYVVTVIGNLLIMLAIG

SNSHLHTPMYFFLSNLSFVDLCLVSTIVPKMLENIMMHSKAISYAGCLAQ

MYFFMVFTCMDNLLLTVMAYDRFVAICHPLHYVSIMSTQLCGLLVLLSWI

VSLLISLLHSMMAMHLSFCKDHNIPHFFCDLSQVLKLSCSDTFINNILMY

FATSLLGIVPLTGILFSYSQICSSILRVPSPEGKYKAFSTCGSHLCVVSL

FYGTAFGVYLSSSTAHSSWKNSITSVMYTVITPMLNPFIYSLRNKEIKDA

LRRLTIRPTFSQ*

>ModoOR3.3.3

MGPENQTSISEFLLLGFSEKPEHQLPLFGLFLSMYLVTVGGNLLIMLTIG

SDSHLHTPMYFFLSNLSLVDFCLVSTLVPKMLVNFLTHNKAISYADCLTQ

MYFFMVFTCTDNLLLGVMAYDRFVAICHPLSYVTIMSPQFCGLLVFLSWT

ISLLNAILHCLLVMRLLFCSEREIPHFYCDLTPVLKLSCTDTLINNFFVY

FTTVLLGAFPFTGIIFSYTQICSSILRVPSTGGKCKAFSTCGSHLCVVLL

FYGTVLGVYLSSSVTGSSWKNSVASVMYVVVTLMLNPFIYSLRNRDIKDA

LRRLINRMACSW*

>ModoOR3.3.4

MGSENQTSISEFLLLGFSETPEQQLPLFRLFLSMYLVTVGGNLLIMLTIG

SDSHLHTPMYFFLSNLSLVDFCLVSTLVPKMLVNFLTHSKAISYAGCLTQ

MYFFMVFACSDTLLLTVMAYDRFVAICHPLSYVTIMSPQFCGLLVLLSWT

ISLLNAILHCLLVMKLFCTEHEIPLFYCDLTQVLKLSCTDTLINNILVYL

TTVLLGILPFTGIIFSYTQICSSILRVPSTGGKYKAFSTCGSHLCVVSLF

YGTVLGVYLSSSVTQSSWKSSIASVMYAVVTPMLNPFIYSLRNRDIKDAL

RRLISRMACSW*

>ModoOR3.3.5

MEPENQTYLSGFLLLGISEKEEQQMPLFGLFLGMYLVTVFGNVLIMLAIG

SDSHLHTPMYFFLSNLSLVDLCLVTTLVPKMLVNMLTHNKAISYAGCFAQ

MYFFMNFACSDNLLLTVMAYDRFVAICHPLHYVTLMSPRICSLLILISWT

ISILYSIFQIVMMMRLSFCTKREISHFFCELSEVIRISCSDTLINNIVVY

FATGLLGVLPLTGILFSYSQICSSILKVPSPGGKYKAFSTCGSHLSVVSL

FYGTGLGVYLSSATTHYSRNISIASVMYSVVTPMLNPFIYSLRNKDIKDA

LKRIINRTASP*

>ModoOR3.3.6

MEPENQTYLSGFLLLGISEKEEQQIPLFGLFLGMYLVTVFGNALIMLAIG

SDSHLHTPMYFFLSNLSLVDLCLVTTLVPKMLVNMLTQNKVISYAECFSQ

MYFFMIFACSDNLLLTVMAYDRFVAICHPLHYVTLMSPRICSLLILISWS

ISILNSIFQILMMLRLSFCTKREISHFFCDLSEVMKISCSDTLINTIEVY

FATGLLGVLPLTGILFSYSQICSSILKVPSPGGKYKAFSTCGSHLSVVSL

FYGTGLGVYLSSATTHSSRNISIASVMYSVVTPMLNPFIYSLRNKDIKDA

LKRIISRTASS*

>ModoOR3.3.7

MESENQTYVSGFLLLGISEKEEQQMPLFGLFLGMYLVTVFGNVLIMLAIG

SDSHLHTPMYFFLSNLSLVDLCLVTTLVPKMLVNMLTQNKAISYAGCFAQ

MYFFMNFACSDNLLLTVMAYDRFVAICHPLHYVTLMSPRICSLLILISWS

ISILISIFEILMTRRLSFCKEREISHFFCDLSEVIKISCSDTLINNIVVY

FATGLLGILPLTGILFSYSQICSSILKVPSPGGKYKAFSTCGSHISVVSL

FYGTGLGVYLSSATTQSSRNISIASVMYSVVTPMLNPFIYSLRNKDIKDA

LKRIISGTTSP*

>ModoOR3.3.8

MESENQTYMSEFLLLGISEKPEQQIPLFGIFLSMYLVTVFGNVLIMLAIG

SDSHLHTPMYFFLSNLSMVDLYLVTTLVPKMLVNMLTHNNAISYAGCFAQ

MYFFLFFICLDNLLLTVMAYDRFVAICHPLHYVTIMSPRICSLLILISWT

ISILNSIIQILMMMRLSFCTEHEISHFFCDLNEVMKISCSDTLINTIVVY

FATGLLGVLPLTGILFSYSQICSSILKVPSRGGKYKAFSTCGSHICVVSL

FYGTGLGVYLSSATTHSSRNISIASVMYSVVTPMLNPFIYSLRNKDIKDA

LKRIISGTASS*

>ModoOR3.3.9

MGPRNQTYVSEFLLLRFSETAEQQMPLFGLFLGMYMVTVVGNLLIMLAIG

SDAHLHTPMYFFLSNLSFVDFCLVSTTVPKMLANILTQNKAISYAGCLAQ

MNFFLVFAFLDNFLLTVMAYDRFVAICHPLHYVTMMSPRFCGLLVLISWS

ISLLNSTLHTSMMMRLTFCSQHELPHFFCDISQVMKLSCSDTLINNILVY

FVTCLLAILPLTGILFSYSQICSSLLRVPSLGGKYKAFSTCGSHLCVVSL

FYGTGLGVYLSSAAVHSSWKIPVASVMYSVVTPMLNPFIYSMRNKDIKDA

LRRLISRAFAFPWT*

>ModoOR3.3.10

MGPRNQTYVSEFLLLGFSETAEQQMPLFGFFLGMYMVTVVGNLLIMLAIG

SDAHLHTPMYFFLSNLSFVDFCLVSTIVPKMLVNILTQNKAISYAECLAQ

MNFFLVFACSDNLFLTVMAYDRFVAICYPLHYVTIMNPRFCGLLVLISWS

ISLLTSIFQILMMMRLSFCTEHEISHFYCDLTEVMKLSCSNTLINTIIVY

FITTLLGVVPFTGILFSYTQICSSILRVPSPGGKYKAFSTCGSHLCVVSL

FYGTGLGVYLSSAAVHSSWKIPVASVMYSVVTPMLNPFIYSMRNKDIKDA

LRRLISRTSDPQ*

>ModoOR3.3.11

MVPGNQTQITEFILLGFSKKPEQQGPLFGLFLGMYLIAVVGNLLIILAIG

SDYHLHTPMYFFLSNLSFVDFCLVSTIIPKMLMNILTQSKVILYVDCLAQ

MHFFIVFTSLDNFLLTAMAYDRFVAICHPLHYTVIMSPRLCVLLFSLSWI

LSLLNSFLHSLMVMRLSFCKDHEIQHFFCDLDQVLKLSCSDTLINYIFIY

TAIGLLGIIPLTGIVFSYSQICCSILKIPSAGGKYKAFSTCGSHISVVSL

FYGTGLGVYICSSITQSSWKSTVASALYTVVTPMLNPFVYSLRNKEIKDA

LRRIIRRITSSQ*

>ModoOR3.3.12

MLPENETQFTEFILLGFSEKPEQQGAIFLLFLGMYLVTVFGNLLIMLAIG

SDSHLHTPMYFFLSNLSFVDLCVASTTVPKMLMNILTQSKVITYADCLAQ

MYFFLVFAGLDNFLLMAMAYDRFVAICHPLHYTVIMSPRLCVLLLLLSWI

LSLLNSLLHSLMVTQLSFCTDHTIQHFFCDLPQVLKLSCSDNLVNNVLAY

MLAGLIGIVSITGIFFSYSQICSSILKVPSAGGKYKAFSTCGSHLSVVSL

FYGTGLGVYFSSSVIHSSWKSTVASAMYAVVTPMLNPFIYALRNKDIKDA

LRKVISRKTSFQ*

>ModoOR3.3.13P

VLGNLLIMLAIGSDSHIHTPMYFFLSKLSFVNLCMVSTTVPKMLVNILTQ

SKVIPYSSCLAQMCLFLVFAGLDNFLLTTMTYDYFVAICHPLHYTAIMNH

RFCGLLVLLYWMMLQSLMVTRFSFSIDHVIQHFFCDFPQVLKFSCSDIYI

NHVLVYTIAELLGSLSLLGTLFSYGQICSSILKIPSGRSKYKAFSTRGSH

LSVVSLFYGTGLGVYLSSSVTHWKSTVASAMYAVVTPMLNPFIYTLRHKD

IKDAFKKLIGRIASSQ

>ModoOR3.3.14P

LVPGNQTQFTGFILLGFSEIPEQQGPLLGLFLGLYLVAVLGNLLIILVIG

SDSHLHTPMYFFLSNLSFVNFCLVSTLVPKMLVNTSSQNKAIPYADCLVQ

MYFFFVF/FLFFVGLDNFLLTVMAYDRFVAICHPLRYATIMRHRLCVLLV

LLSWILSLLYSLLHCLMVTWLSFCDREIQHFFCDLDQVLKLSCSDTLINY

ILKYIIAGLLGIIPLTGILFLYSQICSSILKVPSTEGKYKAFSTCGSHLS

VVSLFYGTGLGVYFSSSSIHSSWMSTVASTMYTVVTPMLNPFIYSLRNKD

IKDALRRLISRITSSQ

>ModoOR3.3.15

MAPENQTKFIEFILLGFSETPEQQRAIFGLFLGMYLVTVFGNILIMLAVG

SDSHLHTPMYFFLSNLSFVDLCVVSTTVPKMLVSILTQNKAISYAGCLAQ

MYLFIVFAGLDNFLLTAMAYDRFVAICHPLRYTSIMSPELCCLLVLLSWI

LSLLDALLHSLMVTRLSFCTGHEIQHFFCDLDQVLKLSCSDTLINYVLIY

ILAGLLGILPLTGILFSYSQIFSSILKVPSAGGKYKAFSTCGSHLSVVSL

FYGTGLGVYLSSSVTQSSWKSTVASAMYAVVTPMLNPFIYCLRNKDIKGA

LWRLISRIFSTQ*

>ModoOR3.3.16P

LVPGNQIQLIEFILQGFYETPEQQGPLFGLFLGMYLVTVVGNLLLMLTIA

SYSYLHTPMYFFLFNLSFVDLCIITSTVPNMLVSL

>ModoOR3.3.17

MAPENQTKFTEFILLGFSETPEQQGAIFGLFLGMYLVTVFGNILIMLAVG

FDSHLHTPMYFFLSNLSFVDLSMVSTTVPKMLVGILTQNKAISYAGCLAQ

MYFFTVFIGLDNFLLTAMAYDRFVAICHPLRYTSIMSPELCCLLVLLSWI

LSLLVALLHSLMVTRLSFCRGHKIQHFICDLDQVLKLSCSDTLINYVLIY

IVAGLLGILPLTGILFSYSQIFSSILKIPSTGGKYKAFSTCGSHLSVVSL

FYSTGLGVYLSSSVTHSSWKSAVASAMYAVATPMLNPFIYCLRNKDIKGA

LWRLISRISSSQ*

>ModoOR4.1.1P

RKKSFSICSSHLTVVMVFYGTILFMYMKPKSKNSVTDKFITLFYSLVTPM

LNPIIYTLWNKEVNRGMRNVLQRVTFLRGL

>ModoOR4.2.1

MAIGNNTLVTEFILLGLTEEVDLQIPLFLVFLGIYMVSVVGNLGLILLIW

ISSPLHIPMYYFLCNLSFIDLCYSSVIVPKMLVNFISEKNIISYTGCMTQ

IFFFCFFAIDECYMLTAMAYDRYVAICSPLLYHSTMSPWICSLLVLVVYL

MGASGAIALTHGMTRLFFCGENVVDNYFCDILPLLKLACSSTNINELLLV

FIGGFNVLATILPILISYTFIILSILRIQSAKGRYKAFSTCGSHLASVAI

FYGSIIFVYYTPASSSKTNQEKVASVFYTIVNPMLNPLIYSLRNKDVKNA

LWKVMQGRMSSKSN*

>ModoOR4.2.2

MDKRNHSMVTNFILMGLTDQPELQLPLFILFLEIYIISIVGNMGLLLLIR

ISSQLHTPMYYFLSNLSFIDLCYSSVITPKMLVSFVSEQNIISYSGCLTQ

FFFFCTFGIADCYMLTAMAYDRYVAICCPLLYNSTMSQTLCSMLVVGVYT

MGIFGAITHTSCLARLSFCGNNVISHYYCDIPPLLKLSCSSTYINELLVM

FLVVVNSLVTTLPILISYAFILSNILSIHSSKGRSKAFSTCGSHLASVAV

LYGSVIFMYCQPSSSSNITQQKVSSVIYTTVIPMLNPLIYSLRNKDVKDA

LKKIRKDWIVS*

>ModoOR4.2.3P

QKPLFLLFLGI*LVSMMKNHGLILSELVLSFIHP*TVFLNSLLFMHLCYS

SIITLKILENFLSEENIFSHTGCTAQYFFFLFVSAEFYMLTIIAYNHYAA

ISKLL*KFIMEP*LCSLLVADLYTVRDIGVILI/DNVIHQYFFDVLPLLK

LLFSRPFHQ*GFGEVY**YNLSETTLSFLITYTFILLTILCIKSLKGYYK

AFSISGSHLAIDSVFYGPIISVCYIPTSNSVT*EKVGSVFNTTMISM*TP

>ModoOR4.2.4

MAIGNNTLVTEFIILGLTEEVDLQIPLFLVFLGIYMVSVVGNLGLILLIW

ISSPLHVPMYYFLCNLSFIDLCYSSVIVPKMLVNFISEKNIISYTGCMTQ

LFFFCFFAIDECYMLTAMAYDRYVAICSPLLYHRTMSPWICSLLVLVVYL

MGASGAIAHTHGMTRLFFCEKNAVNNYFCDILPLLKLACSSTNINELLVV

FIGGFNVLATILPILISYTFIILSILRIQSAKGRYKAFSTCGSHLASVAI

FYGSIIFVYYTPASSSKTNQEKVASVFYTIVNPMLNPLIYSLRNKDVKNA

LWKVMRGRMLSKSN*

>ModoOR4.2.5P

KVKNYSEVTEFILLGIPHTEGLEKVLFGIFLFIYIFTLLGNFLILVAIIS

SSRLHTPMYFFLGLLSTFDLFFPSVSSPKMLVFLSGNSRVISYKGCASQL

FFYHFMGCTEGILYSVMSYDRFVAICHPLRYTVIMNPRLCVFLAACTTLI

GCIHATILTSLTFQLPYCGPNEVDYYFCDIPAIIPLACADNSLVQRVGFT

NVGLLTVTLLFTILVSYTHIGIAILRIRSAEGRSKAFSTCSAHLAAIICA

FGPIIIIYFQSTPNPLLGAVVQILNNIVSPMLNSLIYSLRNKEVKRALKR

VL

>ModoOR4.2.6P

FCGNIFISHYFCDIPPLLKLSCSSTYINELLVMLLVVVNSLVAIIPIFIS

FAFILSSILSIHSVKGRYKAFITCRSPMVTVAVLYGSIIFMYCWPESRNT

ITLQNVASVIYTTVIPMLNPFIYHLRNKDVKDALKKIKEDWIV

>ModoOR4.2.7P

KIKNYSEVTEFILLGIPHTEGLEKVLFVMFLFIYIFTLLGNFLILVAIIS

SSRLHTPMYFFLGLLSTFDLFFPSVSSPKMLVFLSGNSRVISYKGCASQL

FFYHFMGCTESILYSVMSYDRFVAICHPLRYTVIMNPRLYFGLAACTTLI

GCIHATILTSLTFQLPYCGPNEVDYYFCDIPAIIPLACADNSLVQRVGFT

NVGLLIVTLLFTILVFYTRIGIAILRIRSAEGRSKAFSTCSAHLAAIMCA

FGPVMIIYFQSTPNPLLGAVVQILNNIVSPMLNSLIYSLRNKEVKRALKR

VL

>ModoOR4.2.8

MDKGNHSIVADFILMGLTDKPEFQIPLFLLFLEIYIISFMGNMGLILLIW

INSQLHTPMYYFLSNLSFIDLCYSSIITPKMLVSFLSEKNIISYSGCLTQ

FFFFCTFGIADWYMLTAMAYDRYVAICSPLHYHSTMSQKIRYLLVLGVYV

MGTLGAITHTSGLSRLSFCGNIFISHYFCDIPPLLKLSCSSTYINELLVM

LLVVVNSLVTTIPIVISYAFILSSILSIHSVKGRYKAFSTCGSHMVTVAV

LYGSIIFMYCWPESSNNITQQKVASVIYTTVIPMLNPFIYSLRNKDVKDA

LKKIKKDWIVSWCN*

>ModoOR4.2.9P

ITILPFFLGIYLVFM*GNHNLIIIIIWISSQLHMYMHYFLNNLSFINLCY

SSVITLKMMENIVSEKDIISHSGCQLFFFLFIVIAEFYMLTTMAY

>ModoOR4.2.10

MAMGNNTIVTEFILLGLTNQTEIKIPLFLLFLGIYLVSMVGNLGLILLIW

VSSQLHTPMYYFLSNLSFIDLCYSTVITPKMLENFVSEKNIISYQGCITQ

FFFFLFFVIAEFYMLTTMAYDRYVAICRPLLYNVTMVPSFCSLLVAGVYT

MGGIGAIVHTTCLARLSFCGDNVIHHYFCDVLPLLKLSCSSTVINELLVV

FVGGFNLMATTLPILISYIFILLNILRIPSTKGRYKAFSTCGSHLAAVSV

FYGSIIFMYYIPTSNSVTQEKVGSVFYTTVIPMLNPLIYSLRNKDVKDAL

RKVMKGIIFSNST*

>ModoOR4.2.11

MASENNFTVTEFIIMGLSDRQELQLPLFILFLGIYIVSMVGNLVLILVIR

ISSQLHTPMYYFLSNLSFIDLCYSSVITPKMLVNFLSKKNIISYSGCLTQ

FFFFCSFAVSECYMLTAMAYDRYVAICSPLHYTVTMSQKICSLLVSGVYT

MGTFSGLVQTVYLAKLFFCQGNVISNYFCDILPLLKLSCSSTYINELLMM

FLVGFNSLVTTLPIFISYAFIFSSILSIHTAKGRSKAFSTCGSHLTGVII

FYGSIIFMYYKPVSSYNVIQEKVASVIYTMVIPMLNPLIYSLRNKDVKDS

LRKVMKSRALP*

>ModoOR4.2.12

MSMEIKNHTVVTEFILLGIPHTEGQEQVLFVVFLIFYLCTLLGNLLILVA

VMSDSRLHTPMYFFLCNLSVLDIGFSSVSTPKTLANLLVRNQVISLGGCI

SQVFFYHFLGSTECFLYTVMAYDRFAAICHPLRYTIIMNRRVCTLLAAAT

WFIASFHATVLTTLTFQLPYCGSNVVDYFFCDIFPVVKLACGNTLIIETV

SFTNIGLVGMICFLLILASYIRIVIAILKMHSAEGRRKAASTCVSHLSVV

TLFFGPCALIYTQPSLSEVLVTPVQIFGNIVTPMLNPTIYTLRNKDVKGA

LKKLAGAQVTSEGGH*

>ModoOR4.2.13

MSMEIKNHTVVTEFILLGIPHTEGQEQVLFVVFLIFYLCTLLGNLLILVA

VMSDSRLHTPMYFFLCNLSVLDIGFSSVSTHKMLVNLLVRNQVISLGGCM

SQVFFYHFLGSTECLLYTVMAYDRFAAICHPLRYTIIMNRRVCTLLAAGT

WFTSSFHATILTTLTFQLPYCGSNVVDYFFCDIFPVVKLACGNTLIIETV

SFTNIGLVPMTCFLLILASYIRIVIAILKMHSAEGRRKAASTCVSHLSVV

TLFFGPCALIYTQPSLSEVLVTPVQIFGNVVTPMLNPTIYTLRNKDVKGA

LKKLAGAQVTSEGGH*

>ModoOR4.2.14

MEMKNQTVVTEFILLGIPHTEGLEIELFFVFFFFYFFTLLGNLTILLAII

SSPRLHTPMYFFLCKLSIFDIFFPSVSSPKMLFFLSGNSHAISYGGCVCQ

LFFYHFLGCTECFLYTVMAYDRFVAICYPLRYTVIMSHKVCAILAMGTSF

FGCVQATFLTALTFQLPYCGPNQVEYFFCDIPVMLKLACADTSALEMVGF

ISVGLMPFSCFLLILTSYSRIVCSILRIRSAEGRRRAFSTCSAHLTAILL

FYMPVVLIYLQPTPNPWLNATVQILNNLVTPMLNPLIYSLRNKEVKSSLR

KVLQQIGLLPEQ*

>ModoOR4.2.15

MKNYTVVTEFILLGIPHTEGLEIQLFIVFLSFYFSTLLGNLTILLAIISS

PRLHTPMYFFLCKLSILDIFFPSVSSPKMLSYLSGKSHGISYGGCVCQLF

FYHFLGCTECLLCTVMAYDRFIAICFPLRYTVIMSHKVCTILAMGSSFFG

CIQATFLTALTFQLPYCGPNQVEYYFCDIPVMLKLACADTSALEMVGFIS

VGLMPLSCFLLILTSYSRIVCSILKIRTAEGRYRAFSTCSAHLTAILLLY

IPVVLIYLQPTPNPWLNATVQILNNLVTPMLNPLIFSLRNKEVKSSLKKV

LQQVGFLLEK*

>ModoOR4.2.16

MKMKNHTGLIEFTLLGIPHTEGLETMFFVIFFFIYLFTLVGNSLILMAIL

SSSNLHTPMYFFLGFLSIFDIFFPSVTSPKMLLYLSGQSQAISYRGCASQ

LFFYHFLGCTEGVLYSVMAYDRFVAICHPLRYMVIMNSRLCVILVVAACM

VGCIDATILTAFTFSLPYCGPNKVDHYFCDIPAIIPLACADSSLAQRVGF

TNVGLLALTLFFTILVSYTRIGIAILRIRSAEGRSKAFSTCSAHLTAILC

AYGPVIIIYMQSTPNPLLGAVVQILNNLVSPMLNSLIYSLRNKEVKKALK

KVLYRSGIFVEV*

>ModoOR4.2.17P

KIKNYSEVTEFILLGIPHTEGLEKVLFGIFLFIYIFTLLGNFLILVAIIS

SSRLHTPMYFFLGLLSTFDIFFPSVSSPKMLVFLSGNSRVISYKGCASQL

FFHHFMGCTEGILYSVMSYDRFVAICHPLRYSVIMNPRLCVILATCTTLI

GCIHATILTSLTFQLPYCGPNEVDYYFCDIPVIIPLACADNSLVQRVGFT

NVGLLTVTLLFTILVSYTRIGIAILRIRSAEGRSKAFSTCSAHLAAIMCA

FGPIIIIYIQSTPNPLLGAVVQILNNIVSPMLNSLIYSLRNKEVKRALKR

VFCGAGLISEA

>ModoOR4.2.18

MKIENYSVVTEFILLGIPHTEGMETVLFVIFLLIYIFTLVGNFLILVAII

SSSRLHTPMYFFLGLLSTFDIFFPSVSSPKLLVFLSGNSRAISYRGCASQ

LFFYHFLGCTEGILYSVMSYDRFVAICHPLRYSVIMNPRLCVILATCTTL

IGCIHATILTSLTFQLPYCGPNEVHYYLCDIPVVLPLACADSSLAQRVGF

TNVGLLALTLLFTILVSYTRIGIAILRIRSAEGRSKAFSTCSAHLAAIMC

AYGPVMIIYFQSTPNPLLGAVVQILNNIVSPMLNSLIYSLRNKEVKRALK

RVLCGAGLTVEA*

>ModoOR4.2.19

MEIKNFTEVTEFILLGIPETVGLETVLFVLFLFLYLCTLSGNVLILMAVI

SSSRLHTPMYFFLGNLSIFDMGFSSATSPKMLFYLSGQTPVISFKGCVTQ

LFFYHFLGCTECLLYTVMAYDRFVAICHPLRYRIIMNHRVCSILATGTWM

SGCVHATILTSLTFQLPYCDANQVDYYFCDIPAVLPLACADTSLAQRVSF

TNVGLLSLLCLFLILISYTRIGISISRIRSTEGRQRAFSTCSAHLTAILC

AYGPVIIIYLQPTPSPLLGSVVQILNNLVTPMLNPLIYSLRNKDVKFALK

KVFCNVGPIVENG*

>ModoOR4.2.20

MGIPHTKGLEIELFILFLTFYLLTLLGNLLIIMAIISSSQLHTPMYFFLA

NLSVFDIFFPSVSSPKMLLYLTGYSHTISFHGCASQLFFYHFLGCTECFL

YTVMAYDRFAAICHPLRYTVIMNQRVCAVLTVGTWLGGCLHAAVLTFLIF

KLPYCGPNEINYFFCDIPVVLPLACADTSLAQMVSFTNVGIVALLCFLLI

ITSYIRIVYSILKIHSSEGRRRAFSTCSAHLTSILLFYGPVVLIYLRPAS

TPWLDSVIQVLNNIVTPSLNPLIYTLRNKEVKSALKKVLYQVVNPSAVQV

IGLFSS*

>ModoOR4.2.21

MRNQSVVTEFILLGIPHTEGLEAELFFLFLTFYLLTLLGNLIIFMAIISS

SQLHTPMYFFLANLSVFDIFFPSVSSPKMMLYLMGNSHTISFQGCVSQIF

FYHCLGGTECFLYTVMAYDRFVAICHPLRYTVIMNHKVCTILTIGTWLGG

CFHSTILTFLTFRLPYCGPNEVNYFFCDIPIVLPLACADTSLAQMVSFTN

VGLMPLICFLFIFISYIRIVLSILKIRSSEGRRRAFSTCSAHLTSILLFY

GPVILIYLGPASTPWLGSVIQVLNNIVTPSLNPLIYSLRNKDVKLALRKV

FYQVVNTSGV*

>ModoOR4.2.22

MEKKNQSFVTMFILMGIPHAPELDTALFGIFLVIYVLTIVGNLLILMVIK

VESHLHTPMYYFLAFLSFIDMWFSTVTVPKILMGLLNPDGGAISFHSCVA

QLYFFHVLGSTECFLYTVMSYDRYLAITYPLRYTSMMSGKTCALLTGSTW

FSGSVHSAIQTILTFRLPYCGPNQIQHYFCDAPPILKLACADTSANEKVI

FVNIGVVASGCFFMIFLSYVSIVHAILKIHTAEGRQKAFQTCASHCIVVL

CFFGPCIFIYLRPGSKDAVDGIVAIFYTVLTPLLNPLIYTLRNKEVKIAL

LRLQNRVVFAQRN*

>ModoOR4.2.23

MEGKNQSFVTTFILMGIPHPPELETALFGIFLVVYALTLVGNLLILLVIK

VDSHLRTPMYYFLANLSFIDTWFSTVTVPKILMGLFSTDGGAISFHSCAA

QLYFFHILGGTECFLYTVMSYDRYVAITFPLRYATMMSGKTCALLAGGTW

LTGGLLAALQTILTFRLPYCGPNQIQHYLCDAPPILKLACTDTSVNEMVI

FVNIGVLTIGCFFLIFLSYVSIIRAILKISTAEGRYKAFQTCASHCIVVL

CFFGPCLFIYLRPGSKDAVDGIVAIFYTVLTPMLNPLVYTLRNKEVKTAL

LRLQKRGVFSKGK*

>ModoOR4.2.24

MERDNQSFVTTFILMGIPHPSELSTVLFGIFLVIYALTLVGNLFIVLVIK

VDSHLHTPMYYFLANLSFIDMWFSTVTVPKILMGLFSPDGGIISFQSCVA

QLYSFHILGSTECFLYTVMSYDRYLAITYPLSYATMMSGKMCAILAGGTW

LSGAIHSAAQTALTFRLSYCGPNQIQHYFCDAPPILKLACANTSVNEMVI

FVNIGIVASGCCFLIFLSYMSIVRAILKIRTAEGRHKAFQTCASHCIVVL

CFFGPGLIIYLRPGSMHAVDRIVAIFQTMITPLLNPIVYTLRNKEVKTAL

LRLKYHRNSIQIN*

>ModoOR4.2.25

MDRQNESFVATFILIGFPHAPDLGATLFGIFLVIYTLTLLGNLLILLVIK

MDSHLHTPMYYFLANLSFIDMWFSTVSVPKILMGLLYTDGWAISFHKCVA

QLYFFYGLANTEAFLYTVMSYDRYMAITYPLQYVTIMSGKICTLLAGGTW

LSGAIHSAVLTALTFRLPYCGPNQIEHYLCDAPAILKLACADTSVNEMVI

FVNIGVLASACGFLIFLSYISIIRAILKIRTAEGRHKAFQTCASHCTVVL

CFFGPALIIYLRPGSKHVIDRIVAIFQTVITPLLNPLVYTLRNKEVKMAL

FRLKDRGNFV*

>ModoOR4.2.26

MDRKNESIVTTFILTGIPHVPELGTTLFGIFLLIYILTLVGNLLILLVIK

VDSHLHTPMYYFLANLSFIDMWFSTVTVPKILMTLISTDGGAISFHGCVA

QLYFFYALANTECFLFVLMSYDRYLAITYPLRYTTMMSGKTCAILAGGTW

LSGIVHSTAETTLTFRLPYCGPNKIEHYLCDAPPILKLACADTSVNEMVI

FVNIGLLVCVCSFLIFLSYVSIVRAVLKIRTAEGRHKAFQTCASHCIVVL

CFFVPCVIIYLRPGSKNVMDRIVAIFQTVITPLMNSLVYTLRNKEVKMAL

LRLKDRGKFL*

>ModoOR4.2.27

MDRKNESVVTTFILTGIPHAPELGTTLFGIFLLIYILTLVGNLLILLVIK

VDSHLHTPMYYFLANLSFIDMWFSTVTVPKILMTLISTDGGAISFHGCVA

QLYFFYALANTECFLFVLMSYDRYLAITYPLRYTTMMSGKTCAILAGGTW

LSGIIHSTAETTLTFRLSYCGPNKIEHFLCDAPPILKLACADTSVNEMVI

FVNIGVVTSGCSFLIFLSYVSIVRAVLKIHTAKGRHKAFQTCASHCIVVL

CFFVPGVIIYLRPGSKNVMDRIVAIFQTAITPLMNSLVYTLRNKEVKMAL

LRLKDRGKFL*

>ModoOR4.2.28P

FHFTVGWHSFFHGLGSTECFHYTVMSYDCYFAITYLLSYTTMMNG*ISAL

LAGSTWLCGAIHSAVKTALIFCLPYCGPNQIEHYLCDVPVILKLAHADTS

LNERVTFVNIVAVASGCGFLIFLSYVSIVSAILKICTAEGRHKAFQTCGF

HCIMVFCFSGSALIIYLRPGSKYIMDRIVAIFQTVITPLMNPLVYTLRNK

EVKMALLRLKDRGNFV

>ModoOR4.2.29

MNWQNESFVATFILTGIPHAQDLGTMLFGIFLVIYVLTLVGNLLILLVIK

IDSHLRTPMYYFLANLSFIDMWFSTVTVPKILMTLISTDGGAISFHGCVA

QLYFFYALANTECFLFVLMSYDRYLAITYPLRYTTMMSGKTCAILAGGTW

LSGIIHSTAETTLTFRLPYCGPSQIEHYLCDAPPILKLACADTSVNEMVI

FVNIGVIGSGCCFLIFLSYVSIVRAILKIRTAEGRHKAFQTCASHCIVVL

CFFGPGLIIYLRPGSKHVMDRIIAIFQIVITPLMNPLVYTLRNKEVKMAL

LRLKDQGKYV*

>ModoOR4.2.30

MQSGNQTFVTHFILVGLHHHPKLGVPLFLVFLAIYLLTISGNGLIILTIL

VDVRLHRPMYWFLCHLSFLDMTISCAIVPKMLAGFLLNSRAISFGGCVIQ

LFSFHFLGCTECFLYTLMAYDRFLAICKPLHYATIMTRSVCNCLALGTWL

GGSLHSIFQTCFMFRLPFCGPNQVDYFFCDIPAMLRLACADTVINELVTF

VDIGFLALTCFVLILTSYGYIVAAILRIRSADGRRNAFSTCAAHLTVVIV

YYVPCTFIYLRPGSQEPLDGVVAVFYTVITPLLNPIIYTLRNKEMKSALW

RLGGRREVQPH*

>ModoOR4.2.31

MIKQRAMETEWSNQTLVTHFILEGLANTSEYPILFFFLFLLIYTITVVGN

FFILLTVSLDPHLHSPMYHFLGHLSFLDACLSTVTVPKVLAGLLTPEGKV

ISFGGCAVQLYSFHFLASTECFLYTVMAYDRYLAISQPLKYPVVMNQQVC

AGLAGATWATGAIHSAIHTSLTFRLPYCGPSHVDYFFCDIPPVLKLACTE

TALNELVMLANIGIVATGCLLLIIISYVFIVASVLKIRTADGRRNAFSTC

TAHLTVVFLYYMPPICIYIQPRSSGAGVGAPAVFYTIVTPMLNPFIYTLR

NKEVKRALRRLLGHGPRES*

>ModoOR4.2.32

MYSENWTQVTEFVLIGFPGSWGLQLILFLGLLVTYVVTIMGNVLIIVLSW

SDHRLQTQMYFFLRNLSLLELAWVSVVVPKMMASLLTRDYSISFAACIMQ

SYLYFLFGTTDFFLLAVMSLDRYLAICRPLHYETLMNGPICVRLVIASWI

SGFLWVLSPTILMASLPFCGPNVIDHFFCDSWPLMRLSCANTQLLELVAF

ILSTTVLLGSLALTSVSYGCIFLTVFRAPTAAERRKAFSTCASHLAVVII

AYGSSIFLYIRISETQSLFLKKGASALACIITPLLNPFIFSLRNDKVKQA

LRDALRCHRNIGIRRL*

>ModoOR4.2.33

MYSENWTQVTEFVLIGFPGSWGLQLILFLGLLVTYVVTIMGNVLIIVLSW

SDHRLQTQMYFFLRNLSLLELGWVSVVVPKMLVSLLTRDYSISFAACIMQ

SYFYFLLATTDYFLLAVMSLDRYLAVCRPLHYETLMSGPICVRLVIASWI

SGFLWVLSPTILMANLPFCSSNVIDHFFCDSWPLMQLSCGDTQLLKLLAF

ILSTTVLLGSLALTAISYVYIFLTVFRAPTATERRKAFSTCASHLTVVII

AYGGSIFLYIRTSETQSLFLKKGASALVCIITPLLNPFIFSLRNDKVKQA

LGDALKWHRNIGTRKL*

>ModoOR4.2.34

MYSENWTQVTEFLLMGFPGNWGLQLLLFFGLLITYVVTVMGNLIIIVLSW

SDHRLQTQMYFFLRNLSLLELVLVSVVVPKILISILTGDYTISFTGCIMQ

SYFYFLLGTTDFFLLAVMSLDRYLAICHPLHYETLMNRPICVRLVITSWI

AGFLWVLCPTILMARLPFCGPNVIDHFFCDSWPLMRLSCGSTQLLELVAF

ILSTAVLLSSLALTSVSYTCILATVLRTPTATERKKAFSTCASHLTMVVI

VYGSSIFVYIRTSETQSRLLNKGVSALGCIVTPLLNPFIFSLRNDKVKQA

LGDALRWHRNITTRRI*

>ModoOR4.2.35P

MRPENWTQVTELVFLGFPKNWALQLILFLGLLVTYVVTVMGNLLIIVLSW

SDRRLHTQIYFFLRNLSLLELCWVSVVVPKMLVSLLTRDYTISFAGYIIQ

SFLYFFLGTTDFSLLSIMSLDHYLAICHTMKH**MILSVPG*CWHPGLLD

FSGSFGPLSSWLVCLSVAQMA*IISSVIVSGQLLVAFLLSAALLLGSLTL

TSSSYAFILATVFRAPTATERKKAFSTCASHLTVVVIFYGSSIFLYIRTS

EAQSILLNKVVSALNCIISPLLNPFIFSLRNDNLKQALHDAMRWHRTMGT

RS

>ModoOR4.2.36

MKTENWTQVTEFVLLGFPKSWALQVMLFLGLLVTYMVTVTGNLLIIVLSW

SDRRLHTQMYFFLRNLSLLEMMLVSVVVPKMLVSLLTRDYTISFAGCILQ

SYIYFLLGTTDFFLLAVMSLDRYLAICRPLHYETLMSGPICSRLVLASWL

AGFLWILWPTILMASLPFCGPNGIDHFFCDSWPLMELSCGDTQLLELVAF

LLSAALLLGSLTLTSSSYAFILATIFRAPTATERKKAFSTCASHLTVVVI

FYGSSIFLYIRTSEAQSMLLNKAVSALNCIISPLLNPFIFSLRNDKVKQA

LRDAMRWHRTMGTRS*

>ModoOR4.2.37

MNPVNHTQVTGFVLLGLSQAWELRIFFFMVFLTVYFCTVMGNLLIVAIVS

SDPHLHTTMYFLLANLSFLDFCYSSITSPRMLADLLSGNPTISFGGCLTQ

LFFFHFIGGIKIFLLTVMAYDRYVAISQPLRYPLIMNQGVCGLFMAGSWV

GGFIHSIVQVALTVQLPFCGPNKLDNFYCDVPQLIKLACTDTFALELLMV

SNNGLVTLMCFLVLLGSYTALLIMLRSHSREGRSKALSTCASHIAVVTLI

FVPCIYIYARPFRTFPMDKAVSVLYTMVTPMLNPAIYTLRNKEVIDAMKK

LWRKYIVTRGKQEH*

>ModoOR4.2.38

MGRGNQSEVTEFILAGLTDEPELQLPFFFMFLVIYMVTVVGNMGMIILIG

ISSHLHTPMYYFLSSLSVLDVCYSSVVTPQMLVGFLFEDKTISYPRCMTQ

LFFFCIFVISECYMLAAMAYDRYVAICSPLLYNTIMSPQVCSLLVVFVFS

VGLIDAVIHTGCILRLPFCGSNIISHYFCDIVPLIKLSCASTYLDELLIF

VIGGFNMIATSLTIIISYAFILSSILRINSKEGRSKAFSTCSSHIMAVTV

FYGSLMSMYLKPASSSNLAQEKVSSVFYTTVIPMLNPLIYSLRNKDVKNA

LKKLIQKKISS*

>ModoOR4.2.39P

FILSGLTYEPELQLPLFFMFLVIYMVTVVGNVGMIMLTRISSQLHTTKHY

FFSSL/SP*MLLGFLFKDKIIPYPRYMTQLHFFCIFVISECYMLAAMAYD

HYIAFCSPLLYNIILFTQVCSLAFVFSVGLIDAV

>ModoOR4.2.40

MENQSMVTEFVLVSFPVLQELQTFLFVVLLLTYMLTIAGNIVIISLIWTD

YRLHTPMYFFLSNLSFLDILFTTVIAPKLLSCLLNDRKTISFAGCITQTY

FYFFLGTVEFILLAVMSFDRYVAICNPLRYTIIMNSRVCLLLVLGCWVGA

FLSVLCPTIVVSRLPYCNKEISHFFCDIAPLLQVACIDTHLIEIINFLLS

SMVVLSSLVLTIVSYSYIISTILRIPSAQGRQKAFSTCVSHITVVSIAYG

SSIFMYVRPNQSYSLDFDKVTAVFTTVVTPLLNPFIYSLRNEKVKEVLRE

AISKIISLLPRRS*

>ModoOR4.2.41

MRNGTLITEFILLGFPDIQGLQVPLFIVIFFIYILTIFGNGLIIAIVWTE

QRLQTPMYFFLSNLSFLEIWYTTTVIPKLLETFVVERTIICVPCCLLQSF

FHFFLGTTEFFILTAMSFDRYIAICKPLHYTTLMTSSLCLQLALSSWIIG

FTIVFCQTVLIFQLPFCGNNIINHFYCDVGPILKVACVDTSLLEILGLLA

TILIIPGSLAFTVVSYIYIVSTILKIPSATGRQKAFSTCASHLTVVSLLY

GAVLFMYLRPTAHSSFKINKVVSVLNTIITPLLNPFIYTIRNKEVKEALW

KAMACQKTSHHW*

>ModoOR4.3.1

MTVGNHSIVTEFILMGLTDKPELQLPLFLLFFGVYAVSMVGNLGLVLLIK

ISSQLHTPMYYFLSNLSFIDLCYSSVIIPKMMVNFVSEENIISFPGCLTQ

FFFFGYFAVSECYMLAAMAYDRYVAICHPLLYHITMSRRVCFLLVAGVHI

IGIFGALIHTSFLVGLTFCGGKVINHYFCDILPLLKISCSNTHINEFLLV

FNTGINVLVTTLAIFVSYAFILSNIFQIHSAEGRAKAFKTCGSHIVAVAI

FYGSIIFMYFKPSSSDNNAQGKVASVFYTIVNPMLNPLIYSFRNKDVKDS

LRKVMKDNRF*

>ModoOR4.3.2

MASENKSTVSEFYLEGLTDQPELQFPLFLLFLWVYVVTMLGNMAMIFLIA

VSSQLKSPMYYFLSNLSFIDICYSSVVTPKLLVNFIENQNIISYPGCMTQ

LFFFCFFAIDECYMLTAMASDRYVAICNPLLYNVVMNPRVCSLLATGVYM

MGIVGALAHTTCMARLSFCGPNLINHYLCDILPLLKLSCSSTYINELLVM

FVLGFNVLATSLTILISYAFILSSIFCIHSAQGRYKAFSTCGSHLAAVAI

FYGSIMFMYFKPASSSNMTQEKVASVFYTTVIPMLNPLIYSLRNKDVKEA

LKKVIERRFCCI*

>ModoOR4.3.3P

SSATCPLWISATPXXXXITSYNKCNTLTTLTTTTFFLLFFFIVSECYMLT

AMAYDHYVAICCPLLYNVIMSPWACSFLVTGVYTMGTFTTLVNTTCVVRL

SFCGPNVIKHYFCDIIPLLKLSCSTTYLNELLLMIIGTFNVFSTTVAIFI

SYAFILISILSIQSAEGRSKAFSTCSSHLAAVAIFYSTIIFMYLKPSSRN

RMTREKVASVFYTTIIPMLNLLIYSLRNRDVKDVLKKIMMGTMFNRSM

>ModoOR4.3.4

MASLNHSTVTLFILEGLTDQPELQVFLFVLFLGIYVVTVVGNLGMILLIG

IGPQLKSPMYYFLSNLSFVDLCYSSAITPKLLVNFIEDKNIISYNECMTQ

LFFFCFFIVSECFMLTAMAYDRYVAICSPLLYNVIMSQRVCCFLATAVYT

MGTFAALVHTSSMVRLSFCGPNIIKHYFCDIIPLLKLSCSSTYLNELLLV

VVGMFNVFSTTIAIFISYAFILSSILSIQSAEGRSKAFSTCSSHLAAVAI

FYGTMIFMYLKPPSSSNMTQEKVASVFYTTVIPMLNPLIYSLRNKDVKDV

LKKIMRCTMHNTSM*

>ModoOR4.3.5

MASLNHSTVTLFILEGLTDQPELQVFLFVLFLGIYVVTVVGNLGMILLIG

IGPQLKSPMYYFLSNLSFVDLCYSSAITPKLLVNFIEDKNIISYNECMTQ

LFFFCFFIVSEFYMLTAMAYDRYVAICSPLLYNVIMSPRVCCFLATAVYT

MGTFAALVHTSCMVRLSFCGPNIIKHYFCDIIPLLKLSCSSTYLDELLLV

VVGTFNVFSTTIAIFISYAFILSSILSIQSAEGRSKAFSTCSSHLAAVAI

LYGTVIFMYLKPPSSSNMTQEKVASVFYTTVIPMLNPLIYSLRNKDVKDV

LKKIMRCTMHNTSM*

>ModoOR4.3.6

MASLNHSTVTLFILEGLTDQPELQVFLFVLFLGIYVVTVVGNLGMILLIG

IGPQLKSPMYYFLSNLSFVDLCYSSAITPKLLVNFIEDKNIISYNECMTQ

LFFFCFFIVSEFYMLTAMAYDRYVAICSPLLYNVIMSQRVCCFLATAVYT

MGTFAALVHTSSMVRLSFCGPNVIKHYFCDIIPLLKLSCSSTYLDELLLV

IVGTFNVFSTTIAIFISYAFILSSILSIQSAEGRSKAFSTCSSHLAAVAI

LYGTVIFMYLKPPSSSNMTQEKVASIFYTTVIPMLNPLIYSLRNKDVKDV

LKKIMRCTMHNRSM*

>ModoOR4.3.7

MASLNHSTVTLFILEGLTDQPELQVFLFILFLGIYVVTVVGNLGMILLIG

IGPQLKSPMYYFLSNLSFVDLCYSSAITPKLLVNFIEDKNIICYNECMTQ

LFFFCFFIVSECFMLTAMAYDRYVAICNPLLYNVIMSPRVCSFLATGVYS

MGAFTTLVHTSCMVRLSFCGPNVIKHYFCDIIPLLKLSCSSTYLNELLLL

VIGTFNVFSTTIAIFISYAFILSSILSIQSAEGRSKAFSTCSSHLAAVAI

FYGTIIFMYLKPPSSSNMTQEKIASVFYTTVIPMLNPLIYSLRNKDVKDV

LKKIMRCTMHNTSM*

>ModoOR4.3.8

MASLNHSTVTMFILEGLTDQPGLQVFLFILFLGIYVVTVVGNLGMILLIA

IGPQLKSPMYYFLSNLSFVDLCYSSAITPKLLVNFIEDKNIISYMGCMTQ

LFFFCFFIVSECFMLTAMAYDRYVAICNPLLYNVIMSPRVCSFLATGVYS

MGSVTTLVHASCMVRLSFCGPNIIKHYFCDIVPILKLSCSSTYLNELLLV

VIGISNVFSTAIAIFISYAFILSSILSIQSAEGRSKAFSTCSSHLAAVAI

FYGTIIFMYLKPPSSSNMTQEKVASVFYTTVIPMLNPLIYSLRNKDVKDV

LKKIMRCTMHNRSM*

>ModoOR4.3.9

MTAGNFSTEIEFVLVGLTNRPELQLPLFFLFLCIYIVTMVGNVGMILLII

ASPPLHTPMYYFLSSLSFVDFCYSSAITPKMLVNFLGKRNTIPYFKCMAQ

LFFFVVFVVAEGYLLTVMAYDRYVAICNPLLYNTIMSYQVCSLMVAAAFT

LGFISAMAHMSFMMKLSFCKSHIISHYFCDVLPLLNLSCSSTKINELLLH

VIAGFNTLVPTLAVLISYAFILASILRIHSTEGRSKAFGTCSSHLMAVVI

FFGSITFMYFKPPSSKSMEQEKVSSVFYTTVIPMLNPLIYSLRNKDVKKA

LRNSFREREF*

>ModoOR4.3.10

MDTGNNSAMTEFILAGLTYWPGLQIPLFLLFLSIYVVTMVGNLSMITLIG

VSSHLHTPMYYLLSSLSFIDLCHSTVIIPKMLVNFVSEKNIISYKGCMTQ

LYFFLIFVIAECHMLAAMAYDRYVAICRPLLYNVIMSNQVCIWLVIGVYI

MAFVGATAHTGCMLRVFFCKDNVINHYFCDLNPLLELSCSSIYINEMMVV

CLSAFNILTPIVTILSSYIFIIASILRIRSTEGRFKAFSTCSSHMAAVGV

FFGSGSFMYLQPSSVSSMEQGKVSSLFYSIIVPMLNPLIYSLRNKDVKVA

MKKFIERRIFW*

>ModoOR4.3.11

MDKENNSAVTNFILEGLTDRPELQIPLLLLFLSIYVVTIMGNLGMVTLIG

VSSHLHTPMYYLLSSLSFIDVCHSTVITPKMLVNFVSEKNIISYNGCMTQ

LYFFLIFVIAECHMLAAMAYDRYVAICSPLLYNVIMSHQVCIWLVAGVYI

MAFIGATAHTGCMLRVFFCKDLVNHYFCDLNPLLEISCSSTYVNEVLVLC

FSAFNILTPTLTILSSYVFIITSILRIRSTDGRSKAFSTCSSHMTAVGIF

FGSAAFMYLQPSSVSSMEQAKVSSVFYTILVPMLNPLIYSLRNKDVKVAM

KKFVERRWFQ*

>ModoOR4.3.12

MTMRNNSPVTEFMLSGLTDWPEIQLPLFFLFLGVYVVTVVGNLGMIILIG

LSSNLHTPMYYFLSSLSFIDLCHSTIITPKMLVKFVSEYNVISYPECITQ

LYFFLIFAIAECHMLAVMAYDRYVAICNPLLYNVTMSHQVCSWLVGGVYL

LGFIGATAHTSCILRVLLCKNHISHYFCDLLSLLKLSCSSTYVNEVVILC

FSAFNILIPSITIICSYICIIASILRIRSPEGRSKAFSTCSSHMSAVALF

YGSAAFIYLQPSSENSTDQEKVSSVFYTIFVPMLNPLIYSLRNKDVKVAM

RKILERRRLI*

>ModoOR4.3.13P

GNHSEVTEFIIAGLTDQPELQIPLFIFFLGIYVVTVVGNLGMIILIILGS

HLHTPMYYFLSCLSFIDLCQSTVITPKMLVNFVSEKNIISYSECIFQLYF

FIFLIISECYMLAVMAYDRYVAICSPLLYNVIMSHQVCSWLVGAVGVVAV

VGATVNIGCMLRVSFCKDNIVNNFFCDLLPLFKLSCSDTYINEVVLLAFG

SFNLFVPSLTILSSYVFILFSILRIQSTAGRSKAFSTCSSHIMAVSLFFG

STAYMYLQPSTNSMDQGKVSSVFYTIVIPMLNPLIYSLRNKDVKTALRKI

LEKRVY

>ModoOR4.3.14

METGNHSTVTDFILAGLTDQPKLKFLLFFLFLCIYVTTVVGNLGMITLIG

FSSHLHTPMYYLLSCLSFIDLCHSTVITPKMLVNFMSKKNIISYNECMTQ

LYFFIIFIVSECHLLTVMAYDRYVAICSPLLYNVIMSYQVCSWLLGGVSM

IGLLAATAQTGCILRSFFCKDNIINHYFCDLNPLLELSCYSTYACEVVIL

LFGVINIIFPILTILSSYFFIIASILRIQSTEGRFKAFSTCSSHIIVVSI

FYGSAAFMYLQPSSVSSMDQGKVSSVFYTILVPMLNPLIYSLRNKDVKVA

LIKILERKKFW*

>ModoOR4.3.15P

GNQTSVTEFIIAGLSDQPELQIPLFLLFFGIYVVTVLGNLGIIILIGLSS

NLHTPMYYFLSCLSFIDFCQSTVITPKMFVNFVSEKNIISYSECMVQLYF

FLIFIISECHMLAVMAYDRYVAICSPLLYNVIMSYHMCSRLLGGVVAMGV

IGATVHTGCILRVYFCKDNIVNNFFCDLLPLFKLSCSDTYINEVVLLAFC

SFNLFLPTVTIVSSYVFILFSILRIQSTAGRSKAFSTCSSHIMAVSLFFG

STAYMYLQPSTNSKDQRKVSSVFYTIVIPMLNPLIYSLRNKDVKIALKKI

LGKRVF

>ModoOR4.3.16P

GNQTSVTEFIIAGLTDQPELQVPLFLLFLGIYVVTVLGNLGMILLIGLSS

NLHTPMYYFLSCLSFIDFCQSTVITPKMFVNFVSEKNIISYSECMVQLYI

FLIFIISECHMLAVMAYDRYVAICSPLLYNTIMSYHMCSRLLGGVGAMAV

VGATVHTGCMLRVYFCKDNIVNNFFCDLLPLFKLSCSDTYINEVVLLAFG

SFNLFVPSLTIFSSYVFILFSILRIQSTAGRSKAFSTCSSHIMAVSLFYG

STAYMYLQPSTNSMDQGKVSSVFYTIVIPMLNPLIYSLRNKDVKIALKKI

LEKRLF

>ModoOR4.3.17

MFTGNNSTVTEFILAGLTNQPELQLPLFLLFLGIYLFTVVGNLGMIILIG

LSSHLHTPMYYFLSCLSFIDFCHSTVITPKMLVNFVVEKNIISYPECMTQ

LYFFLIFVIADCYMLAVMAYDRYVAICRPLLYNVIMSPQICSGLVSVVYI

MAFLGATTHTACMLKVFFCKKNVINHYFCDLLPLLKLSCSSTYINEAMIL

FFGSLNIFAPVLTIVSSYIYIIASILRIQSTEGRSKAFSTCSSHIMAVSL

FYGSTAFMYLQPSSVGSMDQGKVSSVFYTIVIPMLNPLIYSLRNKDVKVA

LRKILQRRKFW*

>ModoOR4.3.18P

NDSIVTEFIIAGLTDQQEFQLPLFFLFLGIYLVTAIGNLGMTLLIIFDSN

LHTPMYYFLSCLSFIDLCHSTVITPKMLVNFVSEKNIISYPECMIQLYFF

IIFIISECHMLAVMAYDRYVAICSPLLYNVIMSNXXXXXXXXXQFILDAC

*SFTLQRQYVK*FFCDLLPSEKLACSSTYINEVVILAFTSFNFFIPTLTI

FSSYAVILFRILHIQSIEGRSKAFGTCSSHIMGVALFFGSAGFMYLQPSS

SNSMEQGKVSSVFYTIVMPMLNPLIYSLRNKDVKVALKKMLERRKFS

>ModoOR4.3.19

MAMRNNSPVTEFMLSGLTDWPEIQLPLFFLFLGIYVITVVGNLGMIILIV

LSSHLHTPMYYFLSSLSFIDLCHSTIITPKMLVKFVSEYNIISYPECITQ

LYFFLIFAIAECHMLAVMAYDRYVAVCSPLLYNVIMPHKVCFCLVGGVGI

IAVFGATVHTGCVLRVYFCKDNIVNNFFCDLLPLLKLACSSTYINELVIL

AFTLFNFFIPTLTIFSSYAVILFRIFRIQSIEGRSKAFGTCSSHIMGVAL

FFGSAGIMYLQPSASNSMEQGKVSSVFYTVVMPMLNPLIYSLRNKDVKVA

LKKMLERRKFS*

>ModoOR4.3.20

MTMRNNSPVTEFILSGLTDWPEIQLPLFFLFLGIYVITVVGNLGMIILIV

LSSHLHTPMYYFLSSLSFIDLCHSTIITPKMLVKFVSEYNIISYPECITQ

LYFFLIFAIAECHMLAVMAYDRYVAVCNPLLYTVTMSHQVCLWLVGGVYL

LGLVGATAHTGCILRVFFCKNHISHYFCEILSLFKISCSSTYINEVVLLS

FGAFNILIPSIIIVSSYICIINSILHIQSLEGRSKAFSTCSSHMAAVALF

YGSAAFIYLQPSSENSTDQEKVSSVFYTIFVPMLNPLIYSLRNKDVKVAM

RKIMEKRNLV*

>ModoOR4.3.21

MDTNNHSTVTQFFLKGLTDEPGFQLPLFILFLLVYSASVLGNVGMIILIV

LNPHLHTPMYYFISNLSFIDLCHTTIITPKMLVNFVSEKNIISYSECMSQ

LYFFLIFVTAESHALAVMAYDRYVAICSPLLYNVLMNYQVCSWLMGGVYF

LGIVDSTIHIGFMQRLLFCKANVIDHYFCDLHPLLSLSCSSTYVNELLVL

YLSAFNIFTPVLTIFSSYIFIIASILRIRSTEGRFKAFTTCSSHIAAVTL

AFGSGMVVYLQPLSAHSMTQGKVSSVFYTIILPMLNPLIYSLRNKDVKIA

LKKTLEKIRFS*

>ModoOR4.3.22P

MEKRNYSLVTEFILIGLTDQPELQLPLFFLFLGIYVVSVVGNLGMIVLII

LSSHLHNPMYFFLCSLSFIDLCQSTVITPKMLVNFVSGMNKISYSECMTQ

FYFFGFLGVCECQMLAVMAYDRYVAICRPLLYNLIYS

>ModoOR4.3.23P

LLPLLKLSCSSTYVNELVTLATNTFVVLFPTLAIITSYVFIIVSILKIHS

TEGRSKAFSTCSSHIAAIGVFFGSVAFMYLQPSSGNSMDQGKVSSVFYTI

IVPMLNPLIYSLRNNDVKVALRKIIERR

>ModoOR4.3.24P

SLVTEFILIGLTDQPEL*PPLFFLFPRIYVANVVGNLSMIILIELSSHLH

NPMYFFSL*FVIC*SLSSNCHHSQNASEFCVREERHLLLRVHDTVLFFFL

FFHF*MSNVNNDGI**LLCCHMPSPSL*SYDVL*DLLMVNDWGVLIGATA

HTVFLLTMVICRENAINHYFYDLLPVLELSCSTSYVNEVVTLVCDTFSIL

LPILIIICFYILIISNILKIQPTEGRSKAFSTCSSHIAEFFFLALLHLQP

SSDNFMDQGTMSSVFY

>ModoOR4.3.25

MEKGNHSLVTEFILIGLTDHSELQLPLFFLFLGIYLVTLVGNLGMIILIG

LSSHLHNPMYFFLCSLSFIDLCQSTVITPKMLVNFISEKNTISYPECMTQ

FYIFAFFTISECQMLAVMAYDRYVAICRPLLYSLIMSYQMCSWLVVGVYS

LGLLGATAHTSCMLRVYFCKENIIDHYFCDLLPLLKLSCSSIYVNKLVSL

VSSTFNIFFPTLTIICSYVFIISSILKIQSSEGRYKAFSTCSSHMVAVGV

FFGSLTFMYLKPSSLTSMDQGKVSSVFYTIIVPMLNPLIYSLRNKDVKIA

LRKVIEKRTL*

>ModoOR4.3.26

MFLDNDSSVTEYILAGLTDQPELQLPLFLLFLGVYVVTLVGNLGLIILIM

TNSHLHTPMYYFLFNLSFIDLWYSTVFTPKMLMNFILEKNIISYSGCMAQ

LFFFCFFVISECYVLTIMAYDRYVAICNPLLYNAIMSYQVCSCLLGWAYV

IGFAGAMAHTGCMLKLSFCDDNIINHYMCDVLPLLQLSCTSTYVNELVVF

IVVGINVIVTSVTIFTSYALILSSILSMSSTERRYKAFSTCSSHIIVVAL

FFGSASFMYLKPASSEAVDQGKVSSIFYTNMGPMLNPLIYSLRNKDVQIA

LKKTLRRMFSRTGVGFS*

>ModoOR4.3.27

MAKGNYSLVTEFILKGLTDQPELQLPLFLLFLGIYMVTVVGNLGMIILIR

LSSYLHNPMYYFLSSLSFIDLCQSNIIIPKMLMNFLSKKNTISYPECLTQ

FYFFVFFGISECQMLAVMAYDRYVAICNPLVYSLVMSYQVCLWLVGGVYA

LGLAGATTNTGCLFRVLFCKKNVIEHYFCDLLPLLKLSCSSTFINEVVAL

AFSAFNIIFPTLIIICSYILIIASILKIQSSEGRYKAFSTCSSHIAAVGI

FFGSLAFMYLQPSSVSSMDQVKVSSVFYSIIVPMLNPLIYSLRNKDVKIA

LKKVIERIFK*

>ModoOR4.3.28P

MATGYYSLLTEFILIELTDQPKFQLPLLLLFLDIYVVTMVENLGMIMVDW

NQFPYSHPHVLFSLKFILQ*FLSINCHHPKMLVNFVSEKNTPFLVFL*FL

NVKCWP*WHMTTMLLYFIPFFIVLSCHFRCVHGEWLGYIYLSWLGAQLT*

TACLE*FFCKEIVVNNYFCDLFPFLKLSCSSTYVNEMVNL

>ModoOR4.3.29P

FQLYRPHIIAFGVFFFSDAFMYLHPSSVNSMDQGKVTSVFYTIIVPMLNP

LIYSLRNKDVKTSLRKVIER

>ModoOR4.3.30

MEKENSSLVTEFILIGLTDLPELQLPLLFLFLGIYLVTVVGNLSMVTLIG

QSSHLHNPMYYFLCNLSFIDLCQSTVIIPKMLMNFSSEKSIISYPECMTQ

FYIFAFLTISECHMLAAMAYDRYVAICNPLLYNVLMSNQLCSWLVVGVYA

LGLVEATAHTGCLLRVFFCEENAINHYFCDLLPLLKLSCSSTYVNEVMAL

SLSAFNILFPILTIICSYIFIIASILKIQSTEGRSKAFSTCSSHIAAVGV

FFGSLAFMYLQPSSVSSMDQGKVSSVFYTIIVPMLNPLIYSLRNKDVKIA

LRKVIERTL*

>ModoOR4.3.31

MVRENGSLVTEFILIGLTDQPDLQLPLFLLFLCIYVVSVVGNLGMIILIR

LSSHLHTPMYYFLCSLSFIDVCQSTVITPKMLVNFMWKKNTITYSECMTQ

FYFFVIFVICECQMLAVMAYDRYVAICHPLLYSLIMSYQMCSWLVGGVYA

FGLVGATAHTGCLLRVLFCKENVINHYFCDLLPLLKLSCSSTYVNEMVSL

AFSTFNILFPTLAIICSYVFIIASILQIQSTEGRSKVFSTCSSHITAVGV

FFGSAAFMYLQPSSMSSMDQAKVSSVFYTIIVPMLNPLIYSLRNKDVKIA

LKKVTDRITF*

>ModoOR4.3.32

MEKGNYSVVIEFILIGLTDQPEIQLPFFFLFLGIFMISVWGNLGMIILIG

LSSHLHKPMYYFLFNLSFIDLCQSTVITPKMLLNFVSEKNTISYPDCMTQ

FYFFAFFGISECQILAVMAYDRYVAICHPLLYNVIMSYQKCSWLVGGVYA

FGLMGATAHTGCLLRVLFCKENAINHYFCDLLPLLKLSCSSTYVNEMVSL

AFSTFNILFPTLAIICSYVFIIASILKIQSTEGRSKAFSTCSSHIAAVGV

FYGSVAFMYLQPSSVRSMDQGKVSSVFYTIIVPMLNPLIYSMRNKDVKIA

LRKVIVGRTS*

>ModoOR4.3.33P

MEKGNYSLVTEFILIGLTDQLELQLPLFLLFLGIHVFTMAWNLGMLILIK

LSSHLHTHMYYFLCSLSFIDLCQSTDIIPKMLVNFPSEKSTISYPA*LNS

IFFVFF/FFLSFFAIS*CQMLAVMAYDCYVAICHPLLYNLIMLFQMCSWL

VGGVYALGLIGATVHIGCMLRLLFCKENTINHYFCDFLPLLKL/SCSSTY

VNKVVTLVFSAFNILFPTLTIICSYIFIIASILKIQSTEGRSKATRTCSS

HIAAVGVFFGSTAFIYLQPSSVSSMDQGKLSSVFYTIIVPMLTCLIYSLR

NKDVKNALRKEI

>ModoOR4.3.34

MATGNRSTVMEFILAGLTDQPELQLPLFLLFLGIYVVTVVGNLGMILLIA

ISSQLHSPMYYFLSHLSFIDLCYSSVITPKMLVNFVSEKNFISFLECMTQ

LYFFLIFVIAEGYLLTAMAYDRYVAICSPLLYNVLMSQRMCSVMMFVVYS

LGFFGATVHTSRMAMLSFCGSHIVRHYFCDILPLLTLSCSSTHINEVLLF

IIGGVNTLAPTLAVLVSYTFILSSILRIRSTEGRSKAFGTCSSHLMAVGI

FFGSITFMYFKPPSSNSMEQEKISSVFYTTVIPMLNPLIYSLRNKDVKNA

IRKVMGN*

>ModoOR4.3.35P

MVLENDSSVTELILAGLTDQPDLQILLFLLFLGVYVITLVGNLGLIILIM

TNSHLHTPMYYFLFNLSFIDLWYSSVFTPKMLMNFILAKNIISYSGCMAQ

LFFFCFFL/FLLFFFVISECYMLTIMAYDRYVAICNPLLYNVVMSHQVCS

CLLS*AYVMGFAGAMAHTVCMLRLSFCNDNIINHYMCYGVALLKLS*TST

YVNEVLVSIVVGTNVIVPSFTIFTSYALILSSILSISSSEGRCKAFSTCS

SHIIVVAIFFGSASFTYLKPSFSEAVDHGKVSSVFYTNVVPMLNPLIYSL

RNKDVQIAFRKTLGKMFYRIKVGF

>ModoOR4.3.36P

MALGNDSSVTEFILAGLTDQPDLQLPLFLLFLGVYVITLVGNLGLIILIM

TNSHLHTPMYYFLFNLSFIDFWYSSVFTPKMLMNFILAKNIISYSGCMAQ

LFFFCF/FLLLFVISECYVLTIMAYDRYVAICNPLLYNVVMSHQVCSCLL

GWAYVMGFAGAMAQTGFMLKVSFCDDNIINHYMCHASALLPLSCTSTYVN

ELVVLIVVGTNVIVPSVTIFTSYVLILSSILNIT/SSEGRSKAFSTCSSH

VMVVAIFFGSASFTYLKPFYSQTVDQGKVSSVFYTNIVPMLNPLIYSLRN

KDVQIALTNTLRKMFSRTEAEFS

>ModoOR4.3.37P

LQMYIDLIKKLILTTL*LFTM*YIVPVHYLHHSVQYLEHQLSEGRSKTFS

TCSFHIIVVALFFGSASFTYLKPSSSKSVKQGNLSSVFYTNVLSMPHKLI

YSLRNKCVQIALKKNLREMFSRTGAGF

>ModoOR4.3.38P

MALENDSSVTQFILAGLTGQPDLQISLFLLFLGVYVNTLVGNLGLIILIK

MDSHLHTPMYYFLFNLSFIDFWYSSVFTLKMLLNFI*KISSIQDV*LSSI

SSVFLKIISECYVLTIMAYDHCVAICNLLLYKVIMSYQISSCLLGGAYVM

VFAGAMTHTLCMLRLSFCDGNIINHYMCLGVAFLQFSCSSTYVNELVVLI

LLWT

>ModoOR4.3.39

MALGNDSSVTEFILVGLTDQPDLQLPLFLLFLGIYVVTLVGNLGLIILIL

MNSHLHTPMYYFLFNLSFIDFWYSSVFTPKMLMNFILDKNIISYSGCMAQ

FYFFCFFIIAECYVLTIMAYDRYVAICNPLLYNVIMSYQVCACLLGGAYV

MGFTGAMAHTVCMLRLSFCDDNIINHYMCLGAALLQLSCTSTYVNELLAF

IIVGTNVIVPSVTIVTSYVLILSSILSISSSEGRSKAFSTCSSHVIVVAI

FFGSASITYLKPLSSGAVDQDKVSSVFYTNVVPMLNPLIYSLRNKDVQIA

LRKTLTKMFSKTEAGF*

>ModoOR4.3.40

MALENDSLVTEFILVGLTDQPDLQLPLFLLFLGVYVITIVGNLGLIILIL

MNSHLHTPMYYFLFNLSFIDLWYSTVFTPKMLMNFVLEKNIISYSGCMAQ

LYFFCFFVISECYMLTIMAYDRYVAICNPLLYNVIMSYRVSSCLLGWAYV

MGFAGAMAHTVCMLKLSFCDDNIINHYMCDILPLLQLSCTSTYANELVIF

ISVGINVIVTSVTIFTSYALILSSILSISSSEGRSKAFSTCSSHIIVVAL

FFGSASFMYLNPASSEAVDQGKVSSIFYTNMGPMLNPLIYSLRNKDVQIA

LKKTLRKMFSRTGVGFS*

>ModoOR4.3.41

MASKNDSSVTEFILAGLTDQPELQLPLFLLFLAAYMITIIGNLGLIILIK

MNSHLHTPMYYFLFNLSFIDLCYSSVVTPKMLMNFVLKKNIISYSGCMAQ

LYFYCFFVISECYMLTIMAYDRYVAICNPLLYHVTMSHQVCTCLMGGAYV

MGFAGAMAHTGCMLRLSFCDANIINHYMCDILPLLQLSCTSTYVNELVVF

IVVGINIIVPSVTIFTSYALILSSILSMSSTEGRSKSFSTCSSHIIAVTL

FFGSGSFMFLKPSSSGSMDQGKVSSVFYTNIGSMLNPLIYSLRNKDVQIA

LRKTLRKIFFSRAKAGFS*

>ModoOR4.3.42

MASKNDSLVTEFILAGLTDQPELQIPLFLLFLGVYVITEVGNLGLIILIM

TNSHLHTPMYYFLFNLSFIDFCYSSVFTPKMSMNFILEKNIISYSGCMAQ

LYFFCFFVISECCMLTIMAYDRYVAICNPLLYHVTMSHQVCSWLLGGAYV

MGFAGAMAHTGCMLRLSFCDANIINHYMCDILPLLQLSCTSTYVNELVVF

IVVGINIIVPSVTIFTSYALILSNILSMSSTEGRSKAFSTCSSHLIAVAL

FFGSGSFMYLKPSSSGYIDHSKIYSIIYTNVGPMLNPFIYSLRNKDVQLA

LRKTLRRRMFSRTGAGFS*

>ModoOR4.3.43

MDLENDSSVTEFILAGLTNQPELQFPLFLLFLGIYVITIVGNLGLITLIS

MNSQLHTPMYYFLFNLSFIDFCYSSVFSPKMLMNFVSKENIISYSGCMAQ

LSFFCFFFFISECCMLTIMAYDHYVAICNPLLYHVTMSHQVCSWLLGGAY

VMGFAGAMAHTGCMLRLSFCDANIINHYMCDILPLLQLSCTSTYVNELVV

FIVVGINIIVPSVTIFTSYALILSSILSMSSTEGRSKAFSTCSSHLIAVA

LFFGSGSFMYLKPSSSGSADQDKVSSVFYTNVGPMLNPLIYSLRNKDVQL

SLRKTVRRMFS*

>ModoOR4.3.44

MATGNDSSVTEFFLAGLTDQPELQLFLFFLFLGIYVVTVLGNLGLIILIR

LNSHLHTPMYYFLFNLSFIDLCYSSVITPKMLMNFVSEKNVISYSGCMAQ

LYFFCFFGLSESLLLSAMAYDRYIAICNPLLYTITMSSKVCFLLVTGVYV

LGFAGAMAHTGCMLRLSFCDNNIINHYMCDILPILELSCTSTYVNELVAF

IVASIDIGMISSTIFTSYAFIFSNILRISSSGGRSKAFSTCSSHIVTVSL

FFGSGAFMYLKPSSVGSMDQGKVSSVFYTIVVPMLNPLIYSLRNKDVKLA

MRKTMRPRMIF*

>ModoOR4.3.45P

TIVGNLLLMILIKINPHLHTPMYFFLFNMSLIDLCYSSVIIPKMLMNFVS

EKNIISYSGCMAQLYFFCVFVNSESLLLSAMAYDQYVAICYPLLYNIKMS

SQICSFLVSGVFVMGFAVALAHTGCMLRLSFCASNIINHYMCDIPPLPPE

LSCTSTYVNELVVFIVTSIELAVIGSAIFPSYALIFSTILRINSNSDRSK

AFSTCSSHLTAIFLFFGSGIFVYLKPSSSKSMNQRKVSSVFYTIVIPMLS

PIIYSLRNKDVKLAMQKTLRPRMI

>ModoOR4.3.46

MGRENDSFVSEFILFGLTDQPELQIPLFFLFLGIYVITVVGNLGLIVLIG

LNSHLHTPMYYFLFNLSFIDLCYSSSIIPKMLVNFVSAKNTISYSGCMAQ

LYFFCFFVVSESFLLSAMAYDRYVAICKPLLYNITMSYQVCCLLAVCAYV

MGFSGAMPHTGSMLRLSFCNNNIINHYMCDILPLLELSCTSTYVNELVVF

IVVGIDIGVPTITIFISYALILSSILHINSTEGRYKAFSTCSSHMMAVSL

FFGSGAFMYLKPSSLLSMSQGKVSSVFYTIVVPMLNPLIYSLRNKDVKLA

MKKIVSKIIF*

>ModoOR4.3.47

MTAENHSTATEFILAGLTDLPEVQLPLFFLFLGIYTITMVGNLGMITLIG

LNSNLHTPMYYFLSNLSFVDLCYSSVITPKMLGNFVLEKNIISYPGCMTQ

LYFFLVFVVAESYMLTVMAYDRYVAICNPLLYNVIMSHQVCFLLVAIVYI

MGFIGSTIETVLMSKLSYCKFLISHYFCDILPLMKLSCSSTYDIEMVAFF

LAGFDIIATSLTILVSYAFILSSILRIRSTEGRSKAFSTCSSHIAAVGLF

YGSTAFMYLKPSTASSLAQENVASIFYTTVIPMLNPLIYSLRNKEVKAAL

QKTLRINMS*

>ModoOR4.3.48

MGRENDSFVSEFILFGLTDQPELQIPLFFLFLGIYVITVVGNLGLIVLIG

LNSHLHTPMYYFLFNLSFVDLCYSSSIIPKMLVNFVSVKNTISYSGCMAQ

LYFFCFFVIYESFLLSAMAYDRYVAICKPLLYNITMSYQVCCLLAVCAYV

MGFSGAMPHTGSMLRLSFCNNNIINHYMCDILPLLELSCTSTYVNELVVF

ILVGIDIGVPTITIFISYALILSSILHINSTEGRYKAFSTCSSHMMAVSL

FFGSGAFMYLKPSSLLSMSQGKVSSVFYTIVVPMLNPLIYSLRNKDVKLA

MKKILSRKIF*

>ModoOR4.3.49

MAAENHSTATEFILAGLTDLPEVQLPLFFLFLGIYTITMVGNLGMITLIG

LNSNLHTPMYYFLSNLSFVDLCYSSVITPKMLGNFVLEKNTISYPGCMTQ

LYFFLVFVIAECYMLTVMAYDRYVAICNPLLYNVIMSHQVCFLLVAIVYI

MGFIGSTIETVLMSKLSYCKFLISHYFCDILPLMKLSCSSTYDIEMLIFF

LAGFNIIATSLTVLVSYAFILSSILRIRSTEGRSKAFSTCSSHIAAVGLF

YGSTAFMYLKPSTASSLAQENVASIFYTTVIPMLNPLIYSLRNKEVKAAL

QKTLRINMS*

>ModoOR4.4.1

MDTKACNSSDGSPIFFYLMGIPSLPKSLFLPVFFIFLILYLLVIIGNFLL

LVAVVVDSKLHKPMYFFLTNLSALDILFTTTTIPRMLSLFLLGDRILSFP

ACFLQMYFFHSFSCTEAFILVVMAYDRYEAICHPLHYTVRMTPQVNVKLA

ASAWLAALLLPIPAVVQSSQMAFGTLVQVLHCFCDHLAVVQASCSDPTFQ

TFLGFCCAMAVSFTPLLLVVLSYARILISILKISSKEGRAKAFSTCTSHL

LVVGTYYTSIAVSYVAYRADLPVDFHIMGNVVYAILTPVVNPLIYTLRNK

DVKAAITKFMIP*

>ModoOR4.4.2

MDAWTCNNSDGSPISFYLMGIPSLSKSLFLPIFFIFLILYLIVIAGNFLI

LVAVMSDSNLHKPMYFFLTNLSVLDILFTTTTIPKMLSLYLLGDRILSFP

ACFLQMYFFHSFSCSEAFMLVVMAYDRYEAICHPLHYTVRMTPQVNAGLA

ASAWLAALLLPIPAVVQSSQMAFGTLVQVLHCFCDHLAVVQASCSDPTFQ

TFLGFCCAMAVSFTPLLLVVLSYARILISILKISSKEGRAKAFSTCTSHL

LVVGTYYTSIIVSYVVYRANLPVDFHIMGNVVHSIFTPIVNPLIYTLRNK

DVKAAITKFMSLWSRGYP*

>ModoOR4.4.3

MTHCGNQSIPSHKTVFILLGFPALQETPLPLFFTVLALYTFIVVGNVLIL

VAVVGDPRLHKPMYFFLCNLSAVDILFTTTTVPKMLAMFLFQHNTISFEG

CFLQMYSFHSLGVMESFILVVMAYDRYEAICHPLHYPVRMTPQANARLAA

SAWAMALVIPAPVMVETSQLEYTARATVEHCFCDHLAVVRAACQDAGTEQ

QTFIGFCIAMTVSLVPLFLVLFSYGKILASILRIASQEGRAKAFSTCTSH

LMVVGTYYSSIAVAYVSYQMEMAIDLHVLSNVIYAILTPMLNPLIYTLRN

KDVKEAIKTVFQHVFSHLKNP*

>ModoOR4.4.4

MTHCSNQSIPSHKTVFILLGFPALQETPLPLFFTFLALYTFIVVGNVLIL

VAVVGDPRLHKPMYFFLCNLSAVDILFTTTTVPKMLAMFLFQHNTISFEG

CFLQMYSFHSLGVMESFILVVMAYDRYEAICHPLHYPVRMTPQANARLAA

SAWAMALIIPAPVMVETSQLEYTARATVEHCFCDHLAVVRAACQDAGTEQ

QTFIGFCIAMTVSLVPLFLVLFSYGKILASILRIASQEGRAKAFSTCTSH

LMVVGTYYSSIAVAYVSYQMEMAIDLHVLSNVICAILAPMLNPLIYTLRN

KDVKEAIKTVFQHVISHLKNL*

>ModoOR4.4.5

MAHCSNQSIPPHVTVFLLLGFPALQEAPLPLFFTFLALYTLIVVGNVLIL

VAVVGDPRLHKPMYFFLCNLSVLDILFTTTTVPKMLAMFLFQHNTISFEG

CFLQMYSFHTLTVMESFILVVMAYDRYEAICHPLHYPVRMTPQANARLAA

SAWAMALVIPAPVMVETSQLEYTARATVEHCFCDHLAVVRAACQDAGTEQ

QTFIGFCIAMTVSLVPLFLVLFSYGKILASVLRIASQEGRAKAFSTCTSH

LLVVGTYYSSIAVAYVSYQMEMAIDLHVLSNVIFAILTPMLNPLIYTLRN

KDVKEAIKTVFQHVFSHLKNH*

>ModoOR4.5.1P

TSNISYTDFFLVGFPGLREWRSLLVLPLSFLYALILSANALVICTVAIH*

SLHQPMYMLISLLLAVNICAASAVVPQMLSGFVHYANAISL*ACLVQMFF

IYFTLLLDYNLLLAMALDRYVAICHPLRYADLVTPHLLGLLAVVALIRSL

AVAVPLVVLTSRAHFCRTAVIRHFACEYIALLSIACGDITFNNRLGLAMR

LITVTFDLSLLGASYTRIIYVAFQISSGNARAKALHTCGSHLLVIFTIYL

SGLSTSIVFRFAKSVSQDVQNLLSAIYLLLPGALNPILY

>ModoOR4.5.2

MTRWNETHNISYTDFFLVGFPGLQETRSLLILPFSCIYLVILSANALVIY

TVVAQRSLHQPMYALIALLLTVNLCAATAVVPAMLYSFSTHYYRISLSCC

LLQMFSIYFLIVFDCNILLVMALDRYVAICYPLRYPEIVTGQLLAGLVGL

AAMRSTCIVAPVVILASRVRFCRSNIIRHFTCEHMALMKLSCGDISLNKT

VGLAVRVFNRVLDMLLLGTSYTRIIHAAFRISSGGARSKALNTCGSHLLV

IFTVYSSTLSSSIVYRIARTASQDVHNLLSAFYLLLPCLVNPVIYGARTK

EIRQHLVKIFQRAGPQATSEKNPSQPPQRELPS*

>ModoOR4.5.3

MSEWNETHNVSYIDFFLVGFPGLNESRSLLILPFSCIYLVILLANMLVIY

TVVVQRSLHQPMYALIALLLTVNICSATVVMPTMLVSFSTHYYHISLSCC

FIQMFFLYFFIIFDCGIILVMALDRYVAICYPLRYPEIVTGQLLAGLVIL

AALRSTCIVVPVVGLASRVRFCRSNIIRHFTCEHMALMKLSCGDISLNKT

VGLAVRVFNRVLDMLLLGTSYTRIIHAAFQISSGGARSKALNTCGSHLLV

IFTVHSSSFISSIVYRIARTASQDVHNLLSAFYLLLPCLVNPVIYGARTK

EIRQHLVKIFQRAGPQATSKNTHP*

>ModoOR4.5.4

MSGWNMTFNISYTTFFLLGFPGLRESRSLLILPFSCLYMVILSSNGLIIY

IVTTQRSLHQPMYILISLLLAVNICTATTVVPIMLFSFSTHLNRISFTCC

LVQMFFIYCLLVFDCNILLVMALDRYIAICYPLRYTEIMTGQVLMGLVGV

AATRSVGIVAPVVVLASRVHFCRSNVIHHFTCEHMALMKLSCGDISLNKT

VGLALRVSNRILDLLLLGSSYTRIIHAAFRISSGGARSKALHTCGSHLLV

IFTVYGSTLSSSIVYRVARTASQDVHNLISAFYLLIPSLVNPVIYGARTK

EIRQQLAKLFQRERS*

>ModoOR4.5.5

MSGWNVTFNISYATFFLLGFPGLRESRSLLILPFSCLYMVILSFNGLIIY

IVTTQRSLHQPMYILISLLLAVNICTATTVVPAMLFSFSTHLNRISLTCC

LVQMFFIYCLLAFDCNILLLMALDRYIAICYPLRYTEIMTGQVLMGLVGV

AAIWSVGILAPVVVLASRVHFCRSNVIHHFTCEHMALMKLSCGDISLNKT

VGLALRVFNRILDLLLLGASYTRIIHAAFRISSGGARSKALHTCGSHLLV

IFTVYGSTLSSSIVYRVARTASQDVHNLISAFYLLIPCLVNPVIYGARTK

EIRQQLAKLF*

>ModoOR4.5.6P

NMALMKLSCGHTSLNKTMGITILISTQVLDILLLGTSYKHIIQAAFWMSS

GGVHSKP*THVAPIWHLIFMAYSS/LFMASLSSSIVYCVAPIASQKVYIL

LSALYLLLPCLVNPDIYRARTTEIQQYLVKIFH*GG

>ModoOR4.5.7P

WNETHNISYIDFFLVGFPGLQET*SLLILPSSCIYLVILWANALVIYTAV

TQRSLHPPMYALIALLLAVNIHSATAVMPNMLFSFSTHYYHISPSCCLVQ

MFYFFIIFDCGILLAMALDHYIALCYPLCYLAVVTGGQLAGLLALAAA*S

IGIVVVAVVLALRVHFCCFNIVYTFTCEYI

>ModoOR4.5.8T

MSGWNVTFNISYATFFLLGFPGLRESRSLLILPFSCLYMVILSFNGLIIY

IVTTQRSLHQPMYILISLLLAVNICTATTVVPAMLFSFSTHLNRISLTCC

LVQMFFIYCLLAFDCNILLLMALDRYIAICYPLRYTEIMTGQVLMG

>ModoOR4.5.9P

FYTVYSSSYDIIMATSFIAPRILSIFW*VDGVITFTSCFAQMYVVYVAIA

TETSDTSHGL*SLCGHLQTPVV*DHTYTQDDVGNRHVIMVKAILIMTPLS

WMVIQLSFCGSHMVPHFNCEH

>ModoOR4.5.10

MLAPPCNQTSDNSFILLGIPGLDWVHPWLAAPLSFMYAVALVGNTLILTV

IWRDSTLHEPMYYFLCVLAAVDIIMATSVTPKMLSIFWSGDGGITFSACF

AQMYVVHAATAAETGLLLAMAFDRYVAICKPLHYQTILTPKMMLGIGMAI

MMRAILFMTPLSWMVIQLPFCDSHLVPHSYCEHMAVAKLACADYMPSSLY

SLIGSSIIVGSDVSFIATSYSLILQAVFRLSSRNARLKALSTCGSHVGVM

VLYYLPGMISHYVEWFGQDVVPLHTQVLLADFYLVIPPTLNPIIYGLRTK

EIMARVLGVLGTGGALFQGSKCRNHV*

>ModoOR4.5.11

MLAPPCNQTSDNSFILLGIPGLDWVHPWLAAPLSFMYAVALVGNTLILTV

IWRDSTLHEPMYYFLCVLAAVDIIMATSVTPKMLSIFWSGDGGITFSACF

AQMYVVHAATAAETGLLLAMAFDRYVAICKPLHYQTILTPKMMLGIGMAI

MMRAILIMTPLSWMVIQLPFCGSHLVPHSYCEHMAVAKLACADYMPSSLY

SLIGSSIIVGSDVSFIATSYSLILQAVFRLSSRNARLKALSTCGSHVGVM

VLYYLPGMISHYVEWFGQDVVPLHTQVLLADFYLVIPPTLNPIIYGLRTK

QIMARVLGVLGTGGALRQGSKCRNHV*

>ModoOR4.5.12

MFSSQNACFIPTSFLLTGIPGLESMHIWLSIPFGSMYLVAVVGNVTILAV

VKVERSLHQPMYLFLCMLAVIDLVLSTSTMPKLLGIFWFDAGDIGLDACL

AQMFLIHCFATVESGIFLAMAFDRYVAICDPLRHTTVLNHGMVGRMGLAA

LLRGVLYISPLPLMIRLRLPHYRTQIIAHSYCEHMAVVTLACGDTKVNNL

YGLSIGFLVLTLDSLAIAASYVMIFRAVLGLATPEARLKTLGTCGSHICA

ILIFYIPIVVSSLIHRFGHHVPPHIHILLASFYLLIPPILNPIVYAVRTK

QIRERLLRILKTGAHSR*

>ModoOR4.5.13

MFSSQNACSTHTSFLLTGIPGLESMHIWLSIPFGSMYLVAVVGNVTILAV

VKVERSLHQPMYLFLCMLAVIDLVLSTSTMPKLLGIFWFDAGDIGLDACL

AQMFLIHCFATVESGIFLAMAFDRYVAICDPLRHTTVLNHGMVGRMGLAA

LLRGVLYISPLPLMIRLRLPHYRARIIAHSYCEHMAVVTLACGDTKVNNF

YGMGIGFLVLTLDSLAIAASYVMIFRAVLGLATPEARLKTLGTCGSHICA

ILVFYIPIAVSSLTHRFGHQVPPHIHILLANFYMLIPPILNPVVYAVRTK

QIRERLLRFLKAGVQFR*

>ModoOR4.5.14

MSVMNSTSLYPSSFLLLGIPGLEHLHIWISIPFCSAYTLALLGNCTLLFI

IRADATLHEPMYLFLAMLSAIDLVLSSSTLPKMLALFWFGDHEINFHACL

TQMFFLHSISIMESAILVAMAFDRYMAICDPLHYATVLTHELIARIGMAA

VVRAVALMTPLPFLLRRFPYCRGRVIKHCYCEHMAVVKLACGNTRFNNIY

GIAITLVIGGMDLFFVALSYALILRAVLRLASREARFKAFGTCASHVGAI

LAFYTPAVLSSFLHRMARHAAPHVHILVANFYLLFPPMINPIIYGIKTKQ

IRDRVLGLFHRKNV*

>ModoOR4.5.15

MTAMSASNVTSLHPTSFLLMGIPGLEHLHIWISIPFCSAYILALLGNCTL

LFIICTDPALHEPMYLFLAMLSAIDLVLSSSTLPKMLAIFWLGDHEINFH

ACLTQMFFLHSFSIMESAVLLAMAFDRFVAICEPLRYTTILTQPLIARIG

MAAVTRAVTLMTPLPFLLRRFPYCKNRVIAHCYCEHMAVVKLACSNTRFN

NIYGIAVALVIVGLDLLFVVLSYVLILRAVLRLASQEARYKAFGTCASHV

GAILAFYTPVVISSVMHRVARHAAPHVHILAANFYLLFPPMVNPIIYGVK

TKQIRERVLGIFQRKKI*

>ModoOR4.5.16

MTPNNRSHVHPTSFILMGVPGLEASHFWIAFPFCSMYILAVLGNLFVLLV

VWMEPGLHQPMYLFLCMLSALDLVLCTSTVPRMLALFWAGVAEITFGACA

TQMFFIHGFSAVESGILLAMAIDRYVAICRPLHYGTLLPLGTVGRMGLAA

VLRGLGLMTPLTCLLGRLRYCRWVIEHSYCEHMAIVKLACGNTRANNVYG

ITAATLVVGTDSICIAISYALILRAVLALSSKEARAKTFGTCGSHLGVIL

LFYTPGLFSFYTQRFGQHVPHHVHILLADLYLVVPPMLNPIIYGMKTKQI

RDGALRLLQRRPA*

>ModoOR4.5.17

MLELTSNYTSPSITFFLIGIPGLEVTHLWLLVPLSTMYIVALVGNSLILT

VIWVDSALHEPMYYFLCILALVDIVMATSVVPKMLNIFWSGDGIIGFAAC

FTQMYVVHAATAVETGLLLAMAFDRYVAICKPLHYQTILTQQTMLGISVA

IILRATTFMTPLSWMVSHLPFCGSLMVPHSYCEHMAVAKLGCADHMPSDL

YSLIGSSIIVGIDVIFIATCYSRILQAVFRLSCRDAQLKALSTCGSHVGV

MALYYLPGMVSIYVAWFGQDLVPLPVQVLLADFYLVIPPTLNPLIYGLRT

KQIRGRGWSILKSCLPDQAN*

>ModoOR4.5.18P

SNNTSFFSATLFLMGIPGGLEEAHCWLAGPLHECASCCGE*FPHNCNLGR

PWATSAHVLFPMCFGYCGHYHGNLFCPQNAEHLISGNGNVSFTACFAQMY

IIHTATGIETGLLLAMALDWYVTIYKP/LHYNTILIL*TIIAIKMFTVLR

STIALIPLSWMMSHLPYCDYLVAPHSYYE

>ModoOR4.5.19

MLDLLSNHTSLLPATFFLMGVPGLEEAHLWLAALLSTMYTVILMGNSLIM

TVVCVDPVLHEPMYYFLCVLAGVDIIMATSVAPKMLSVFWSGNGTIGFTA

CFTQMYIVHTATAMESGLLLAMAFDRYVAICKPLHYNTILTPQKILGINA

AIVLRAAIALIPLSWMVSSLPYCGSHMIPHSYCEHMAVAKLACADYMPSS

LYSLIGSSVVVGIDVTFITASYILILQAVFSLSSQKARHKALSTCGSHVG

VMALYYLPGLISVYVDWIGKDMVPMHTQVLLADLYLVIPPTLNPIIYGLR

TKRIWDRLRNPLVTSFPGHHFVNPQKDSRSKQVFVMGNRSTEGV*

>ModoOR4.5.20

MVQLHSNHTSLPPATFFLMGIPGLEETHLWLAFLLSTMYTMVLVGNGLIM

TVIWVDPALHEPMYYFLCVLAGVDIIMATSVTPKMLSVFWSGNGNIGFSA

CFTQMYIVHTATALESGLLLAMAFDRYVAICKPLHYNTILTPQKILVINV

AIVVRATIALTPLSWMVSRLPYCGSLAVPHSYCEHNAVANLACADHTPSN

LYALIGSSVVVGIDVTFITASYSLILRAVFHLSSKNARNKALSTCGSHVG

VMALFYLPGLISVYVDWWGQNMVPVHVQVLLADLYLVIPPTLNPLIYGLR

SKKIWQGVWNTMTTNLPCHHYVNSKKERGPKQVFVMGDIRSACEV*

>ModoOR4.5.21

MLGSPFNHTSSNAATFILVGVPGLEAVHLWLAIPLSAMYTVALLGNILIL

TVIWADSGLHEPMYYFLCVLAAVDIVMSTSVAPKMLTIFFSGNGIIGFAA

CFIQMYIVHAATAVETGLLLSMAFDRYVAICKPLHYQTILTPRTMLGIGV

AIMIRAIMVMTPLSWLIVHLPYCRSHVILHSYCEHMAVAKLACADHMPSS

LYSLIASFIIVGFDVTFIATSYTLILWAVFNLSSQDARFKALSTCGSHVT

VMVLYYLPAMVSIYMAWLGQDMVPLHAQVLLADFYLVIPPMLNPLIYGLK

TKRIRERVWNAVVITFTGSNCLNSKKEKRSKQVFAI*

>ModoOR4.5.22

MDSSIFSCNASSHDHPTFLLTGFPGLEASHHWVSIPINLICLISILGNST

ILFLIRTDPSLHEPMFIFLSMLVASDLGLCASTFPTMVQLFWLGARELPI

DLCAAQMFFIHAFTYVESGVLLAMAFDRFVAIKDPLHYSSVLTHSTMAKI

GAGVLVRAVMLNVPGPVLLRRLDFPPVSVLSHCYCLHCDLVGLACSDTKI

NSFFGLVSILLSLGIDSTLIVVSYVLILRTVLGIASPEERFKALNTCVSH

LCIVLIFYMPKLGLSILHRVEKHTYPALAVLMANLHFLVPPFMNPIIYCI

KSKQIRQGLLRRFWQKRVETS*

>ModoOR4.5.23

MLGSLSNDTLLNTATFILLGVPGLEVAHLWLAVPLSAMYCIALLGNIIIV

TVIWTDNALHEPMYYFLCILAAVDIVMSTSVMPKMLNIFWSDNGIIGFAA

CFIQMYIVHATTAIETGLLLAMAFDRYVAICKPLPYQTILTSRTMIGMGM

GIIARAITAMTPMTWMVVHLPYCASHKIPHSYCEHMAVAKLACADHMPSS

LYSLIISFLIVGTDVTFIATSYSLILWTVFSLPSRDARVKALSTCGSHVS

VMVLFYLPGILSIYMAWLGQDMVPLHTQVLLADFYLVIPPMLNPLIYGLK

TKRIRERVWNAVATSLPGHACQNSSKEKRPIRVFVMEHSESTWEAQTQK*

>ModoOR4.5.24

MGSLFNHTLLNTATFILLGVPGLEAAHLWLAVPLSAMYSIALLGNIIIVT

VIWTDSALHEPMYHFLCILAAVDIVMSTSVTPKILTIFWSGNGIIGFAAC

FIQMYIVHASTAVETGLLLAMAFDRYIAICKPLHYQTILTPRTMIGMGMG

IIARAITAVTPMTWMVVHLPYCASHEIPHSYCEHMAVAKLACADYMPSSL

YSLIVSFIVVGIDVTFIATSYSLILRAVFSLPSRDARVKALSTCGSHVSV

MVLYYLPAMVSIYMAWLGEDIVPLHTQVLLADFYLVIPPMLNPLIYGLKT

KRIRERVWNAMANSLPDHICHNSAKDKRPIRVFVMEHSESTWGVQAQK*

>ModoOR4.5.25P

FSLYLVYLVTMVGNLLIILVISLDSYLHTPMYVFLANLSLADISSISTLV

PKMLVNIQTKSQSISFGECVTQMYFSIVFVVIDNFLLGVMVYDRFVAICY

PLNYITVMRPRLCTLLMATSWLFSNLVALTHTLLLVQLTFYDANTIPHFF

CDLAPLLHLSCSGTVLNQLVMFILGSSVIAVPFGLILFSYICIVSTVLKI

SSAEGKWKTFSTCGSHLLVVTLFYGTIVGVYFLPSSAQSVNNEKIGAVLF

IVVTPMVNPFIYSLRNKDIKGALKKIVNKKMSFL

>ModoOR4.5.26P

MAPMENKTQVNEFILWGWTDIPELQAPLFMLSTLIYLIHLVGNLDMVVLD

SWESYLHIPTYFCLRNLSLVDFGYFSVITSQVFS/GLLTGNSVISYNGNA

AQRLFFLAFASTEN*FLATMA*DHHAAMCRSLHYTTIMTTSVCAGLAILS

YICSFLNSAIHTGDTFSLSCNSNIIHQLFSDIPPLLNLSCSDSHTTEWML

FIVGAFNAFFAILVILVLYLFVFITILKICSSQGQQKAFSTCATHITAVS

TFHRTITFMYVQPSSCYSMDIDKIVSMFYTIIFPLVSPSVYILRNKEVK

>ModoOR4.5.27

MMKLNDTLIHPAAFLLVGIPGLGSNTHFWMAFPLCFMYAMATLGNLIIVL

IIRADQTLHEPMYLFLAMLSTIDMILSSVTMPKMASLFFTGNQEIEFNLC

LTQMFLIHALSAMESAVLLAMAFDRFVAICHPLRHASVLTAPVVGKIGLA

ALARGFVFFFPLPFILKRLSYCHSNTVTHSFCLHQDIMKLSCTDTTVNVV

YGLFIILSVMGVDSLFIGFSYILILQAVLGLSSRGAAFKAFNTCISHLCA

VLVFYVPLIGLSVVHRLGGPTSLLHVVMANVYLLLPPVVNPIVYGAKTKQ

IRVRILHMFQHEAG*

>ModoOR4.5.28

MLPKGNHSSATYFILIGIPGMEAAQFWLAFPLCFLYLIAVLGNLTIIYII

RTERSLHEPMYFFLGMLSTIDILISTSSMPKMLAIFWFNATTIQFDACLV

QMFAIHSLSGMESTVLLAMAFDRYVAICHPLRHAAVFTSPRIAKIGLAAV

IRGAALMAPLPIFIKQHPFCRSNILSHSYCLHQDVMKLACADIRVNVIYG

LIVIISAIGLDSVLISLSYLFILKTVLGLTQEAQTKAFSTCVSHVCAVFI

FFVPFIGLSMVHRFGKRPGSNLHIIMANVYLIVPPVLNPIVYGVKTKQIR

QRIVQLFRSVSGTSDL*

>ModoOR4.5.29

MSLLHSHDNNSFFQPSAFLLIGVPGLEAVHGWVSIPFSSMYTVALTGNCL

ILLAVRRTPSLHQPMYYFLSMLALTDLGLTMSTLPTTLAVLWFDYRRIAF

DACLTQMFFLHSFSVVESSVLLAMSFDRFVAISNPLRYASVLTNSVIVRI

GMAIVARATVSLFPVPFLLKRLNFCPRKILLSHSFCFHADVMKRACADIT

VNILYGLYVVLSTVGIDSLLIVLSYGLILHTVMGLASPRERIRALNTCVS

HILAVLVFYIPVIGVSMIHRFGRHAPPIVHALVAYVYLVVPPVLNPIIYS

VKSKHIREALFKILRRKDQG*

>ModoOR4.5.30P

HTHSGVLTFLLPSLPGPGGSGLLTILGLTYSTTLLGNGLILLTICANTYL

HQPQFLLLALLATTDLGLALSMLPTILDALWFPAPTITRDVCLLQMCCLY

AISTGQSSVLLAMALDRWLAVCHPLYYVAFLTPNRLAWTSLAFILRATIP

LLPTPLLIPSCNAHNLSLPFCFPTEVLQLSCGGLGWAYPVVTMLTTATMD

IVLITISYSLVLREVVQRISPAGQHKALRTCTAHLCALLVFYGPLFASVL

FPGSLPGSLASLGLLASPALHPLVYGIQSHRLRRGLLGVL

>ModoOR4.5.31

MMSLPQFHDNGSFFQPSVFLLIGIPGLEAMHGWISIPFSSMYALALAGNC

LILLAVKRTPSLHQPMYYFLSMLSLTDLGLTLCTLPTTLAVLWFDYQRIA

FDACLTQMFFLHSFSVVESSVLLAMSFDRFVAISNPLRYASVLTNSAIGR

IGMAIVTRATVSLFPVPFLLKRLNFCPGKVYLSHSFCFHADMMKRACADI

TVNILYGLYVVLSTAVVDSFLIAISYGLILHTVMGLASPQERIRALNTCV

SHILAVLVFYIPGVGVSIIHRFGRHAPPIVHAIIAYVYLIVPPVLNPIIY

SVKSKHIREALIKMLKKKDQG*

>ModoOR4.5.32

MSLPKSHDNSSFFQPSAFLLIGIPGMEAVHGWVSIPFCSMYTLALTGNCL

ILLAVKRTPSLHQPMYYFLSMLSLTDLGLTLCTLPTTLAVLWFDYRRIAF

DACLTQMFFLHSFSVVESSVLLAMSFDRFVAISNPLRYASVLTNSAIGRI

GMAIVTRATVSLFPVPFLLKRLNFCPGKVYLSHSFCFHADMMKRACADIT

VNILYGLYVVLSTAVVDSLLIALSYGLILHTVMGLASRRERVRALNTCVS

HILAVLVFYIPVIGVSIIHRFGRHAPPIVHALVAYVYLVVPPVLNPIIYS

VKSKHIREALIKMLKKKDQG*

>ModoOR4.5.33

MTSNHTTSQLPAFLLLGIPWREDIHKWLSIPFFFLYLAAALGNGAILVAV

ARNPSLREPMHCFLSMLAVTDLALSGTTLPTTLGLLWFGISQVAFDVCLA

QMFFIHVASVAESSVLLAMAVDRWAAIACPLRYSALLTHRVVAKVGLAVL

VRSALTLLPLPFLLLRLTYNEQRRLTHSFCFHPDIMKLARNETEVNVHYG

LFVILSTAGIDSILIILSYMPIAKVILGLGSWEERVRAARTCVSHVAAVL

VFFVPMIGLSVMHRFGSHGPLPLALVAYTYLLVPPALNPVIYSIKFQCIW

KTLLRLCFYQPRAPRWAASPPPTHAGHKSLPIACSKRYSVQIIQMESSQL

ENSQSDERGIRLVFGDDQV*

>ModoOR4.5.34P

MKSCNFTHTTFILIGLPGLEAAQFWLAFPLLTMYVVAVLGNCMVVFIVRT

EQSLHAPMYHLLCMLAAVDLALSTVIMPKMLALFWFNSQEITFDACLAQI

FFIPTFSAIESTVLLAMAFDRYMAICHPLRHGAVLTNTVLAWIGTVAVIR

SSFFFLPFPLLIKRLHFCGSNILTHSYCVHQDVMKLAYADTMPNVVYGLT

AILLVMGVDAVLISL

>ModoOR4.5.35P

LNNTMFYPQSFFLTGIPGLESFHAWLSIPFCCLYGIALSGNSMIL/FCHI

TEPSLHEPMYYFLSMLSFTDLGLCLSTLITMLSLFWFNAREISFDACIGQ

MFFIHGFTFMESSVLLAMAFDRFIAICNPLRYTTILTNARIAKVGMVIVI

RGTTALVPLLLLLKRLSFCHSHVLHHSYCFHPDVMKLSCSDTKINSIFGL

AIVISTAGVDSIFILLSYVLIIHSILSIASPEERKKAFSTCISHISAVAI

FYIPMISLSLVHRFGKNAPPFVHTLIANVYLLIPPVMNPIIYSVKTKQIR

RAILKIFFPK

>ModoOR4.5.36

MNPCNFTHTTFILIGLPGLEAAQFWIAFPLLSMYVIAVLGNCTVVFIVRT

EHSLHAPMYLFLCMLAAIDLALSTFTMPKILALFWFNSQEIAFDLCLTQM

FFIHALSAIESTILLAMAFDRYVAICHPLRHGAVLTNTFTARIGMVAVVR

GSLFFLPLPLLIKRLHFCGSNTLTHSYCVHQDMMKLAYADTMPNVVYGLT

AILLVMGVDVVLISLSYFLIIRTVLRLPSRAERAKAFGTCVSHIGVVLAF

YVPLIGLSVVHRFGNSLNPIVHVLMGNVYLMLPPVINPLIYAAKTKQIRS

RVLAMFKIICGKEVQVVSNS*

>ModoOR4.5.37P

RFIAICNPLRYTTILTNARIAKVGIVIVIRGTTALVPLLLLVKHLSFCHS

HVLHHSYCFHPNVMKLS*SDTKINSIFGLAIDISSTGVDSVFILLSYILI

IHSILSIASPEEHKKAFSSCISQISAVAIFYIPLINLSLVHRCGKNAPPY

IHTLIGSIYLLIPPVMNSI

>ModoOR4.5.38

MLPFINTTASLTFLLTGVPGLEAFHTWISIPFCCLYATALSGNSMILFVI

ITEPSLHEPMYYFLSMLSTTDLGLSISTLVTMLGIFWFNAREISFNACLA

QMFFIQLFTVMESSVLLAMAFDRFVAISNPLRYATILTESRIAQIGVVIV

TRGTVILTPMVVLLKRLSYCSSHVLHHSYCFHPDVMKLSCTDTKVNSAVG

LAALISTAGVDSIFILLSYVLIIKTVLSIASPMERQKAFSTCISHIGAVA

IFYIPLISLSFVHRFGKQAPPYVHTLIANAYLLIPPVMNPIIYSVKTKQI

RKAVLKVLLPKGS*

>ModoOR4.5.39

MSYHSNNNASSLIFLLTGIPGLEDTHAWLSIPFCCLYLTALSGNGMILFV

IITESSLYEPMYYFLSMLSTTDLGLCISTLVTMLGIFWFNAREISFHACV

AQTFFIQLFTVMESSVLLAMAFDRFVAICNPLRYATILTYSCIIKVWFVI

LFRGTVILTPLVLLLKRLSFCRSHVLHHSYCFHPDIIQLSCSDNKINSVL

GLTALIITAGVDSLFILLSYVLIIKTVLSIASPEERHKAFSTCISHIGAV

AIFYIPLISLCFVHRFGKRAPPYVHTLMANVYLLIPPVMNPIIYSVKTKQ

IRKAIHKVLLPKGSHV*

>ModoOR4.5.40

MSAFLKNTSSPSLTFFLTGVPGLEAAHAWISIPFCCLYITALSGNGMILF

VIITEPSLHEPMYYFLSMLSSTDLGLCTSTLFTVLGIFWFNAREISFNAC

LAQMFFIHLFTFMESSVLLAMAFDRFVAISNPLRYATILTHSRITQIGVA

IVTRGVVILIPLVLLLKRLSFCHSHVLHHSYCFHPDVMKLSCSDTKVNSA

FGLIAIISSAGIDSVFILLSYILIIRSILSIASPEERKKAFSTCISHVTA

VAIFYIPLISLSFVHRFGKKAPPYVPTLIANIYLLIPPVMNPIIYSVKTK

QIRKAVLKVLRPKGALI*

>ModoOR4.5.41

MVVPRLEASHTWISIPFFCLYITALSRNGMILFVIITEPSLHEPMYYFLS

MLSTTDLGLCISTLVTMLRIFWFNAREISFNACMVQMFFIHLFTIMESSV

LLAMAFDRFVAISNPLRYTTILTNARITQIVVAIVSIAIVILIPLILLLR

CLSFYHSHVLQHSYCFHPNVMKLLCSDTKINSAFGLTATILVAGVDSTLI

LLSYVLIIRSILNIASPEECKKAFSSCISHMTAVAIFYIPLTSLSFVHRF

KKKSPTYLPTLISNISLFIPPVMNPNIYSVETKEICKALLKNFCPKRF*

>ModoOR4.5.42

MSAFLKNTSTPSLTFFLTGVPGLEAAHAWISIPFCCLYITALSGNGMILF

VIITEPSLHEPMYYFLSMLSSTDLGLCTSTLFTVLGIFWFNAREISFNAC

LAQMFFIHLFTFMESSVLLAMAFDRFVAISNPLRYATILTHSRITQIGVA

IVTRGVVILIPLVLLLKRLSFCHSHVLHHSYCFHPDVMKLSCSDTKVNSA

FGLIAIISSAGIDSVFILLSYILIIRSILSIASPEERKKAFSTCISHVTA

VAIFYIPLISLSFVHRFGKKAPPYVPTLIANIYLLIPPVMNPIIYSVKTK

QIRKAVLKVLRPKGALI*

>ModoOR4.5.43

MSTFHNSTSLSSLTFILTGVPGLEASHTWISIPFFCLYITALSGNGMILF

VIITEPSLHEPMYYFLSMLSTTDLGLCISTLVTMLRIFWFNAREISFNAC

MAQMFFIHLFTIMESSVLLAMAFDRFVAISNPLRYTTILTNARITQIVVA

IVSIAIVILIPLILLLRRLSFCHSHVLQHSYCFHPDVMKLSCSDTKINSA

FGLTATILVAGVDSTLILLSYVLIIRSILNIASPEERKKTFSTCISHMTA

VAIFYIPLTSLSFVHRFGKKAPTYLPTLISNISLLIPPVMNPIVYSVKTK

EIRKALLKNFCPKIF*

>ModoOR4.5.44

MMETPSNLTSPFSTFHLTGIPGIEEAHAWISIPFCCLYTIALLGNSMILF

VIITEQSLHEPMYFFLSMLSATDLGLTVSTMSTTLSVLWFDARDITIDGC

IVQMFFLHGFTFMESGVLVAMAFDRFVAICDPLRYTTILTNARITQLGVT

MVIRMIVLMLPLLLLFKKLSFCGPNGLSHSYCYHPDVIKLSCSDTKVNSI

CGLAALILSTGIDTPCIVLSYILIIRSVLSIASPTEQKKAFSTCGSHIGA

VAIFYIPMISLSLTHRYGQMAPKIVHTMMANIYLMLPPVLNPIIYSVKTT

QLRKVMGKLLLAK*

>ModoOR4.5.45

MMETPSNLTSPFSTFHLTGIPGIEEAHAWISIPFCCLYTIALLGNSMILF

VIITEQSLHEPMYFFLSMLSATDLGLTVSTMSTTLSVLWFDARDITIDGC

IVQMFFLHGFTFMESGVLVAMAFDRFVAICDPLRYTTILTNARITQLGVT

MVIRMIVLMLPLLLLFKKLSFCGPNGLSHSYCYHPDVIKLSCSDTKVNSI

CGLAALILSTGIDTPCIVLSYILIIRSVLSIASPTEQKKAFSTCGSHIGA

VAIFYIPSISLSLTHRYGQMAPKIVHTMMANIYLMLPPVLNPIIYSVKTT

QLRKVMGKLLLAK*

>ModoOR4.5.46

METSSNLTSLLSTFYLTAIPGIEEAHAWISIPFCCLYTIALLGNSMILLV

IITEQSLHEPMYFFLSMLSATDLGLTVSTMSTTLSVLWFDARDITIDGCI

VQMFFLHGFTFMESGVLVAMAFDRFVAICDPLRYTTILTNARITQLGVAM

FIRMIVLMLPLLLLFKKLSFSHSNGLSHSYCYHPDVIKLSCSDTKVSSIY

GLAALILSTGIDTPCIVLSYILIIRSVLSIASPKEQKKAFSTCGSHIGVV

AIFYIPSISLSLTHRYGQMAPKIVHTMMANIYLMLPPVLNPIIYSMKTTQ

LRKVMGKVDSIVSLE*

>ModoOR4.5.47

MVTLNNLTSPFPTFYLIGIPGLEGSHVWISIPFCCLYAIGLSGNSMILFV

IFTEQSLHEPMYYFLSMLSATDLGLIISTMSTTLTVLWFDLREISLDGCI

VQMFFLHGFTVIETGVLVAMAFDRFVAICDPLRYNTILTNAKILQIGLVI

FMRMIVLLVPTLLLLKRLSFCKMNVLSHSYCYHPDVLKLACSNTKPNSFW

GLTGLVLTSGIDTPCIIISYILIIHSVLSIASPTEQYKTFSTCVSHIGAV

AIFYIPLISLSLVHRFGQSAPKIVPTMMANIYLILPPVLNPVIYSLKTAQ

LRGALIKLLLAKRIPMLG*

>ModoOR4.5.48

MPSLSNITSPIFLLTGIPGLEVFHIWFSIPYFCLCSVALLGNFMIMYVVI

TEQSLHEPMYYFLSMLSATDVGITVSSLPTTLGVLWFNIREISLDGCIVQ

LFFLHGFTLMESSVLLAMAFDRFVAICEPLRYTTILTNSRIVKAALGIFI

RMLINLMPLLLLVKRLSFCGTNSLSHSYCYHPDVIKQSCSSTKINSIVGL

LALILTSGIDIPCIILSYVLIIKSLLSIASPEERHKAFSTCISHIGAVAI

FYIPWIILALMHRFGHNAPPYIHVLLSNIHFLLPPVLNPIIYSVKTKQIY

RVILKVFQANASRV*

>ModoOR4.5.49P

MSSFNNTSSQPLIFLLTGIPGLGAPQMWISIPICLLYAIALSGNSMILFV

VIHEQSLHEPMYYFLAMLSATDMGLSICTLSTTLGVFWFNKREINLDACI

IQMFFLHGLTFMESGVLLAMAFDRFVAICAPLHYATILTNTKIAQIGVGM

LVRDVAVMIPVVLLVKRLSFCQSKVLSHSYCYHVDVIKLSCNDHRINSIM

GLFALFS/GTFCPFFTTGIDCPCILISYVMIIRSVLSIASPEERQKAFNT

CVSHISAVAIFYIPLVSLSLVHRYGQKAPPFIHTMMANIFLLIPPVLNPI

IYSVKTKQIRKAIFK

>ModoOR4.5.50

MSTLNSSTLQPLTFLLTGIPGMSATQVWISIPFCILYAIALTGNSMILFV

VIHERSLHEPMYYFLSMLSATDLGLSLCTLSTTLGVLWFDAREITLDACM

AQMFFLHSFTFMESGVLLAMAFDRFIAICDPLRYATILTKTRIIQIGLAV

GVRVIAVITPMVLLVKRLSFCQRKVLSHSYCYHIDFIKLSCTDNRINSIM

GLFVLFSTSGIDCPCILISYVLIIHSVLSIASPEERKKAFNTCISHISAV

AIFFIPLISLSLVHRYSHNAPPFVHTMMANIFLLVPPVLNPIIYSVKTKQ

IRRAIIKVVHRLAA*

>ModoOR4.5.51

MACNEDNIMPTSGNRSLHPTSFILLGIPGMEKSQFWIAFPFCAMYILALM

GNIIIIHMVRTDHTLHEPMYIFLAMLAFTDLVLSSSTLPKMLGIFWFGSC

EIEYHACLTQVFFIHAFSSVESGVLMAMALDRYVAICFPLRHSAILSTSV

VAKLGMAVMVRGVLWVSPFCFMVTWKPFCHNRIIPQSYCEHMAVLKLVCA

DTRANRKYGLFVAFSVVGFDIIIIAVSYIMILQAVLHLPSGEARIKAFGT

CASHICVILAFYIPALFTFLTHRFGHHVPRVVHIMLGNLYLLVPPMLNPI

IYGVKTKQIRERVIYAFCQKIT*

>ModoOR4.5.52

MACRKDLIMLTSGNNSLHPPFFILLGIPGLEKAQFWIAFPFCAMYAVAMV

GNIIILHVIRTDHTLHEPMYLFLAMLAFNDLVLSSSTLPKMLGIFWFGSC

KIEYHACLTQVFFIHTFSSVESGILMAMALDRYVAICFPLRHSTILSPLV

VAKLGMAVMIRGVLWVSPFCFMVTWKPFCHNRIIPQSYCEHMAVLKLVCA

DTRANRGYGLFVAFSVVGFDIIIIAISYIMILQAVLRLPSGDARLKAFGT

CASHICVILALYIPALFTFLSHRFGHHVPQAVHVMLANIYLLVPPMLNPI

IYGVKTKQIRDRVVNIFCQKVP*

>ModoOR4.5.53

MMPPQPIMLLLPNNTIISLTFKLTVIPGLEAAHIWVSILFYFLYAIAISG

NSMILFVILTNQSLHEPMYYLLFMLSATDMCLSLSTMPTTLGVLCFNIQE

IGWNACISQMFFIHFLTVMESSVLLAMAFDRYIAICNPLRYTTILTPTKI

IHIGMIIMARGTILMTPLLVLLKRLSFCRDNILSHSYCYHPDVIKCSCSD

IRVNSLYGLIALLFSFGLDAPLIGISYVLILHSVFNIASPEERYKAFSTC

ISHIGAVAIFYIPLIGLSAVHRWGKKAPPFVHTLMSNAFLILPPVLNPII

YSVKTKQIRRAILKVFLKKRY*

>ModoOR4.5.54

MMPPQPIMSLLPNNTIISLTFLLTVIPGLEAAHIWISIPFYFLYAIAISG

NSMILFVILTNQSLHEPMYYLLFMLSTTDMCLSLSTMPTTLGVLCFNIQE

IGWNACISQMFFIHFLTVMESSVLLAMAFDRYIAICNPLRYTTILTPTKI

IHIGMIIMARGTILMTPLLVLLKRLSFCRDNILSHSYCYHPDVIKCSCSD

IRVNSLYGLIAVLCSFGLDAPLIGISYVLILLSVFNIASPEERYKAFSTC

ISHIGAVAIFYIPLIGLSAVHRWGKKAPPFAHTLMSNAFLILPPVLNPII

YSVKTKQIRRAILKVFLKKRY*

>ModoOR4.5.55

MLTLNVSHINHHSFILTGIPGMPEKNPWMAFPLGLLYTLTLLGNCTILSI

IKMDRSLHEPMYYFLSILALSDVGLSMSTLPSMLSIFWFNAPEIPFDACI

TQMFFIHGFGVVESGVLVSMAFDRFVAIRDPLRYASVLTSGVIGKIGLGV

IARTVCVVFPVPFLIKRLPFCSSNVLSHSYCLHQDAMRLACASIRVNILY

GLITVICTLGLDALIILFSYMLILKTVLGIASWAERLKAFNTCLSHICAA

LLFYVPLIGVTMAHRFGKHLSPIVHTLMANVYLLLPPVLNPIIYSIKTKQ

IRKRIVQLFIRRKGRT*

>ModoOR4.5.56P

MLSLNISHINHHSFILTGIPGMPEKNSWMAFPLGLLYTLTLLGNCTILSI

IKMDRSLHEPMYYFLSILALTDVGLSISTLPSMLSIFWFNAPEIPFDACI

TQMFFIHVFGITESGVLVSMAFDRFVAIRDPLRYSSILTYEVIGKIGLVV

LARAVFVVLPAPLLIKRLPFYHSNVLSHSYCLHQDVMRLASASTRVNILY

GLIAVICTLGLDVLVILFSYILILKTVLGIASWAERFKAFNT/LSHICAW

LLFYIPFIGATMVHRFGKHLSPIVHTVMANVYLLLPPVLNPLIYSIKTKQ

IRKRIVQLFFRRKGRT

>ModoOR4.5.57

MAATNHSFFQHLHFVLTGIPGLERGYYWMALPLGFIYAIAILGNGIIIST

IKSETSLHIPMYYFLCMLALADLGLALCTLPTMLGIFWFDYKLIAFDACL

VQMYFIHTFSAIESGVLVAMAFDRVVAIRNPLRYGTILTSGVVCRIGAAI

LTRAVCVVFPVPFLIKRLPFYRSNVLSHSFCLHQDVMRLACASTRVNSLY

GLIAVIFTKGSDSLSILLSYIFILRTVLAIASGEGRLKALNTCVSHICAV

LIFYVPLIGVSVIHRFGKHLSPLTHALMANAYLLVPPVLNPIVYTVKTKE

IRRKIIQIFSQTKVTVES*

>ModoOR4.5.58

MTSTFLLVGIPGLVTTPSWWTLILVIIYLLCALGNGTILWVIAVEPSLRR

PMYFFLFLLGVSDVGLATALMPTLLSLALFDSHTVPAFACFLQMFFIHVF

SVMESSVLLAMAFDRALAICRPLRYPALLTNSVAGKIGLAIILRCVGLHL

PLPFLLAWMPYCHSQILSHSYCLHPDVSRLSCPGAQGATYSLFVVFSAMG

LDPVLIFLSYAFIGQALRGVTSHEDRRRAQNTCATHISAVLLFYVPMILL

ALVDRLRIPIPHAAHTFFSYIHFLLPPLLNPVLYSVKMREIRERILRRLQ

PMKVRSFPGGRE*

>ModoOR4.5.59

MSTLNISEGIVSTFILIGIPGLEHIHIWISIPICLMYIIAALGNCTILFI

IKTEPSLHEPMYYFLSMLAISDMGLSFSSLPTMLKIFVFNSPAIFPNACF

AQEFFIHGFTDMESSVLLIMSFDRFLAIRNPLRYSSILTSARVTQLGLVL

AIKSILLVLPLPFILKRLIYCKKRLLSHSYCLHQDVMKLACSDNQVNFIY

GFFVALCMMSDFVFIVISYMLILKTVLSIASHGERLKALNTCVSHICAVL

IFYVPIITLAAMHRFAKHKSPLAMIIIADVFLMVPPLMNPIVYCVKTRQI

REKILGRLGLK*

>ModoOR4.5.60

MVALTNSTTLSLTFFLTGIPGLEDIHIWVSIPFCCLYVIALSGNSMILFV

IITEQSLHEPMYYFLFMLSTTDICLSLSTLPTTLGVFWFNAQEITLDSCI

SQLFFIHFLTIMESSVLLAMSFDRFIAICDPLRYSTILTPIRIIQFGLMM

ILRGSVVMTPVLLLLKRLSFCKNNILSHSYCYHPDVIKHSCSNTRLNSIY

GLIAILLTFGLDAPLIILSYVLIIHSLLSIASPHERQKGFNTCVSHIGAI

SIFYIPLISLSSVHRWGHKAPPFVHTMMSNAFLLLPPVLNPIIYSIKTKQ

IRKVICKIFRKMESRSNVSQF*

>ModoOR4.5.61

MMPPQSILLLLPNTTIISLTFLLTGIPGLEASHLWVSIPFCFLYAIAISG

NSMILFVILTNQSLHEPMYYLLSMLSVTDMCLSLSTMPTTLGVLCFNIQE

IGWNACISQMFFIHFLTVMESSVLLAMAFDRYIAICNPLRYTTILTPTRI

IQIGLIIMGRGILVVIPLLLLLNRLSFCRDNILSHSYCYHPDVIKCSCSN

IRVNSIYGLFALLSTSGLDAPLIGLSYVLILHSVLNIASPEERYKAFSTC

ISHIGAVAVFYIPLIGLSAVHRWGKKAPPFVHTLMSNAFLLLPPVLNPII

YSVKTKQIRRAILKVFLKKGY*

>ModoOR4.5.62

MACRKDIIMPTYGNNSFHPSFFILLGIPGLEKAQFWIAFPFCAMYAVAMV

GNIIILHVIRTDHTLHEPMYLFLAMLAFNDLVLSSSTLPKMLGIFWFGSC

KIEYHACLTQVFFIHTFSSVESGILMAMALDRYVAICFPLRHSTILSPLV

VAKLGMAVMARGVLLVSPFCFMVTWKPFCHNRIIPQSYCEHMAVLKLVCA

DTRANRGYGLFVAFSVGAFDIIIIAISYIMILQAVLRLPSGDARLKAFGT

CASHICVILALYIPALFTFLSHRFGHHVPQVVHVMLANVYLLVPPMLNPI

IYGVKTKQIWDRVVNTFCQKVP*

>ModoOR4.5.63

MSMLNYTRFRHQTFTLTGIPGMPEKDYWMALPLFLLYSVTLLGNITILMV

IRLEQSLHEPMYYFLAMLAATDLSLSLSSLPTMLRVHWFGQYSVAFDACI

TQMFFIHMFGGVESGVLVAMAFDRLATIRSPLHYASILSHGVIGKIGAAI

VFRSVAAVLPVPFLIKRLPFCHSNVLSHAYCLHQDAMRLACADTRINSLY

GLLAVIFIIVLDALVLLFSYILILHAVLGIASREEQFKVLNTCFSHICAV

LLFYIPLIGMTLIHRFGKHLSPIVHTFMANVYLLLPPVLNPIVYSIRTRE

IRRRIVRVLSRAGF*

>ModoOR4.5.64

MLFLNNTTSLSPTFFLNAFPGLEGDYVWLSIPFSCLYVIALLGNCMILFV

IITERSLHKPMYYFVSMLSIVDLGLTMTTLPTVLGVFWFNLQEISFNACA

IQMFFLHGFSFLESSVLLAMAFDRFMAICDPLRYAAILTNSTIMLIGLVI

FVKQVVSLIPIALALKGLSFHREHELSHPYCYHPDVIKHSSANPWLSSLI

GLFFLLSNSGVDLIFILLSYILILRTILSITCPKERRKAFSTCVSHIAAV

AIFFVPMISVSLTHRLFATAPQIFPVIMANIYLLLPPVLNPIIYSLKTKP

IYQAILKLLKTKEALR*

>ModoOR4.5.65

MPADNFSDIEISTFFLIGIPGLEHFHIWISIPICLMYLIAILGNCTILFI

IKTEPSLHEPMYYFLSMLAVSDLGLSLSSLPTMLRIFLFNATGISPSACF

AQEFFIHGFTDMESSVLLIMSFDRFLAIWNPLRYSSILTGARVYKMGLVF

VIKSMLLVLPFPFTLKRLTYCKKSLLSHSYCLHQDVMKLACSDNTVNFFY

GFFVALCMMSDSVFIAISYILILKTVMGIGSHRERLKALNTCVSHICAVL

IFYVPIITLASMHRFGKKSPLALILIADVFLLVPPLMNPIVYCVKTRQIR

EKVLGRLGLKQR*

>ModoOR4.5.66

MSELNTSEVKIFFLIGIPGLEHVHIWISIPVCFMYLIAILGNCTILLVIK

TEASLHEPMYYFLSMLAISDLGLSLSSLPTMLRIFLFNAPGITPGACFAQ

EFFIHGFTVMESSVLLIMSFDRFIAIHNPLRYSSILTTSRISKMGLVMAI

RSFFLVLPFPLTLKRLRYCHKNLLSHSYCLHQDVMKLACSDNRVNVIYGF

FVALCTMMDLACIALSYLLILKTVLGLASFSERLKAFNTCVSHICAVLIF

YVPIITLAAMHRFAKHKSPLTMILIANIFLLVPPLMNPIVYCVKTRQIRE

KIHGKLFTMCGR*

>ModoOR4.5.67P

LLTTARISKMGLVMAIRNFFLVLPFPLTLKRLRYCHKNVLSHSYCFHHDV

MKLACSDNRVNVIYGFFAALCTIMDPVCIALSYLLILKIVLGFASFSERL

KAF*YVCLSHLCSVHLLCAYHYPSCYA*LCQAQISTQ*SSLLTSSFLTSW

VTPLVNPIMYCVKNRQIWEKIHGKLFTICDR

>ModoOR4.5.68

MSTINVTNYDDLNFLLIGIPGLEHVHIWISIPFSSMYIVAILGNCTILFF

IKTEGSLHEPMYYFLSMLSVSDLGLSLSSLPTTMGILLFDVQEIHATACF

TQEFFIHLFTVTEASVLSVMAFDRYVAIRNPLRYSTILTGSRVAKIGFVL

AAKNILLILPLPFLLRRLKFCQKTHLSHSYCLHQDVMKLACSNNRVNIIY

GLCAALSTMLDLIFITFSYIMILKTVLGIAAPKEQLKALNTCISHISAVL

IFYVPMLSAAMLHRFAQHVSPLIHILMADIFLLVPPLMNPIVYCVKTRQI

REKILEKMGLKRR*

>ModoOR4.6.1P

NWTSASEFILLGQSEQSIHQPLIFGLFLSAYLVTVTGNMLVVWPITSGVR

LHTPMYFFLTNPSLTDIASISTTVSKMLENIWTENQTISYVECLTQLYFS

IVFVTVDNFLLATMAYDHYVAICWPLHYATAMSPGLCALMATVSWDVTNL

IALVHILLLDQLQFCRSNTVPHFFCDMPLLLQLT

>ModoOR4.7.1P

MSNFIILTEFLLMGFSDIRELQIVFWLFLHIYLVTLIGNLPIVVATTLDR

SLHTPMYFF/PCTFFLRNLSIIDTCYISVMVPKVSINLLMNNMAISVAGC

AAQIFLVLFLSYVE

>ModoOR4.7.2

MLLMEEWNHTTTGFILLGLFAPTKTGLLLFLLVILIFLIALLGNSTMILL

IWMDGHLHTPMYFLLSQLSLMDLMYICCTVPKMVVNYLSGDNSISFVGCG

FQSVCFLIMACAEGLLLASMAYDRYVAICRPLHYPVLMNKRMCLLMITGS

WVSSSINSISHTVYALSMPYCKSRIINHFFCDIPAMVPLACMDTWAYEYT

MFFSTNLFLLVPFLGIMPSYGRVLYAVHHMRSSQGKKKAFTTCSTHLTVV

TFYYAPFVYTYLRPPSLRSPEEDKNLAVFYTILTPMLNPIIYSLRNKEVL

GALQRVSGRFLPQKE*

>ModoOR4.7.3

MILMLEEKNHTFSKDFILLGILDPNKYGFLFLSFLLIIFMVAIMGNSILI

LLIRLDIRLHTPMYFLLSHLSFMDILHISNIFPKMANNFISGRKSITFTG

CGFQVFFSLIFLGAECLLLTVMSYDRYVAICHPLRYPILMSHRISVIMAA

SCWLVAIINSTIHTTYVLYIPFCGTRAIDHFFCEVPAMLKLSCVDTSHYE

RGVYVSAVFFLLIPFSIILASYGQILRTVLHIKSMEAQKKAFSTCSSHLT

VVVMYYGPFIFTYMRPKSYHTPGQDKILAVLYTILTPMLNPMIYSLRNKD

VLCAMKKVLGSVLMHRN*

>ModoOR4.8.1P

ISNYNTATEFLLMEFSDIGELQILYSFLLFLIYSAGLMGNLPIVMITNFD

RTLHTPMYFFLRNLAMVDVCYISITAPQASVNSLVNNRIISVTGCAAQVF

LV

>ModoOR4.8.2P

ISNYTTATEFLLVEFSDIGELQILYSFLLFLIYSAGLMGNLPIVMITNFD

RRLHTPMYFFLRNLAMVDACYISITAPQASVNSLVTTGYFSYRLCSSGI

>ModoOR4.9.1

MTPGRWSELGNYSEGTEFILMGITDLRGLQLLLFAVLLPTYLLTLLGNLF

IMVLSLADQRLQIPMYYLLRNFSLLEMGFTSAVTPQVLSHLLTGQKTISL

PRCFSQMVLYFILGTVEFFLLAVMSMDRYLAICYPLRYPALMTSRTCLGL

VLGCWAGSFLFLSGPCIWLLLLPLCGPKVLNHFFCDSTPLLALVCTDTRP

LQLFAFLVAVCTLAGALAVTAASYTCIIWTLLHLPSAQGRHKAFSTCSSH

LLVVSITYGSCIIMYLNPTLTGRLDLNKGVAFFNTTVAPLLNPFIYCLRN

KLVQQVSRDMLVRGRGPSRHLRV*

>ModoOR4.9.2

MTPGRWSELGNYSEGTEFILMGITDLRGLQLLLFAVLLPTYLLTLLGNLF

IVVLSLADRRLQTPMYYLLRNFSLLEMGFTSAVTPQFLSHLLTGQKTISL

PRCFSQMVLYFILGTAESFLLAIMSVDRYLAICYPLRYPALMTSRTCLGL

VLGCWAGSFLFLSGPCIWLLLLPLCGPKVLNHFFCDSTPLLALVCTDTRL

LQLFAFLVAVCTLAGALAVTAASYTCIIWTLLHLPSAQGRHKAFSTCSSH

LLVVSISYGSCIIMYLNPTLTGRLDLNKGVAFFNTTVAPLLNPFIYCLRN

KLVQQVSRDMLVRGRGPSRHLRV*

>ModoOR4.10.1

MTNCTTLPGFILQGFSVIPEVQTFSATIFLFIYVFSVLGNISIIIAVNLD

SHLHTPMYFFLKHLSLIDMCSTSTTLPRALFAALAGSEVISLPGCAAQLF

VFVFCGATECFIITIMAYDRCLAIYQPLTYGATMTQQLCCTLVCVAWVCG

LLFSSFHTANTFTLSFCDPTVEHFFCDIPPLLRLACTDPQNHETAGFLVS

GCVIMSCFALTILSYIRILVTVAHIRSAQGRQKALSTCSSHLITVVLFYG

TGSSAYMRPANGFSPLQGRLAAIFYSILTPTLNPIIYSMRNRDMKAALRK

LFCIHGP*

>ModoOR4.10.2T

HVPFTSCMTQLFFFITLICAECTLLASMAYDRYVAICRPLHYPVLMRPQV

CLLLAAASWMGSLSVSVIKTACIAGLSYCGPNVLNHFFCDVSPLLNLSCT

HVALTELVDFISAIVIFYGSLMVALISYMAIGTAVVRMPSAAARLKAFST

CASHLIVVGIFYSASIFIYARPKRMEDMSLSKLLSIIYTVFTPICNPIIY

CLRNKEVQRAFQKTLHKVGISQCSES

>ModoOR4.10.3

MSGPWAQIHPTDPAESLIGSNQSSVVEFVLLGFSHVPRLKLVLFVLFLGM

FLITILGNGLIVLLSLVDTALHTPMYFFLRNLALVEICFSLDIVPRMLES

LVAGRGISLSGCALQLFLLLSCVTSECFLLTVMAYDRYVAICHPLHYGVL

MSQQLCHLLAGACWVAGIPVSLLFTIWLFRFPFCGPRGVRHFLCDVAPLL

RLVCADTSVFEAYIRVATVLVLMVPFSLITVSYGRVLAAVVRMPSATGRH

KALSTCASHFLVVALFYGTACVIHLQPKSTYSPESKQVVSLSYTLVTPML

NPIIYSLRNKEVKAALWRILGRKKGSV*

>ModoOR4.10.4

MTRVQEFILLGLSPRPGLRGVLFAVFLTLYLLTLLENTIIILLIRSHTEL

HKPMYFFLGNLSCLEMCYVSVTMPTLLLGLWSGSCHVPFTSCMTQLFFFI

TLICAECTLLASMAYDRYVAICRPLHYPVLMRPQVCLLLAAASWMGSLSV

SVIKTACIAGLSYCGPNVLNHFFCDVSPLLNLSCTHVALTELVDFISAIV

ILWGSLLVALASYIAIGASVIRMPSAAARLKAFSTCASHLIVVGIFYSAT

IFIYARPSRIEAMDLNKVLSVIYTVLTPMCNPIIYCLRNKEVQGALRKTL

HKVGFSQNSDS*

>ModoOR4.10.5P

WERVDLMDPIVTMARSNESFIGEFILLGFSHVPRLEPLLFVLFLGMFLVT

IQGNSLIVFLTVADSALHTPMYFFL*NLALVDICFSLDIVPKMLESLVAG

RGISLSACALQLFLLLSCVT*ECFLLTVMAYDRYVTIRHPLHYGVLMNHN

LCFLLALACWVTGIPVSLLFIIWLFRFPFCGPRGVHHFLCGVAPLLRLVC

ADTSVFEA/HILVASVFVMMVPFSFIIRSYSHVLAAVIQMPSATGRHKAL

STCTAHLIVVALF/GTACVIHLQPKSTYSPESKQIVSLSYTLVTPMLNPN

IYSLRNKEVKAALRRVLGRKKG

>ModoOR4.10.6

MDGGNETDVTYFILVGFSEHPKAQVIFFCFLLMSYLIILIGNSLILFLIH

YDSRLHTPMYFFLSNLSFLDICYTSSSVPQILINCLVKIPSISLGQCLAQ

MCAGLYLGVAECLLLAVMAYDRCIAIGDPLRYSVRMSFQLCIKLAVASWV

TAFLLTVVPTLTMPLEFCGHHMINLFSCELLAILKRACNDLKFYEWLMMA

TSSLTLLAPFTFILSSYGRILGAVLKMHSAEGRKKAFSTCSSHITVVVIF

YGTAISAYMIPQGKASQDQDKIISMLYGILPPMLNPLIYSLRNKDVKEAL

KKLMGEKNVS*

>ModoOR4.10.7

MDRRNGTDVTYFILIGLSEYPRAQLIFFCILLVAYIVTLLGNSLILLLIH

YDPQLHTPMYFFLSNLSFLDICYTSSTVPQMLINCLVRTPIISLTECLAQ

MCVLLYLGVVECLFLAIMAYDRFVAISNPLHYAVKMGPQLCLQLAVVPWF

VSFIIAVVPILTMPLDFCGHYIINHFSCELLAIIKLACNDLKFFELLIMA

TCSLTLLLPFTFILVSYGHILVAVLKMRSADGRKKAFSTCSSHLTVVVIF

YGTAISMYMMPQDKVSRDKDKIISMMYCIVTPMLNPIIYSLRNKDVKGAL

RKLIGKKSDS*

>ModoOR4.10.8

MDGRNDTAVTYFILLGLSEYPRAQAIFFCLLLVSYLTTVLGNSLILFLIH

YDSRLHTPMYFFLSNLSFLDICYTSSSLPQVLVNCLVRIPAISLGQCLAQ

MCAGLYLGVVECLLLAVMAYDRCVAIGDPLRYPVRMSAQLCAQLAVASWV

SAFLLTVIPTLTTPLEFCGHHIINHFSCELLAVLKLACNDLRLNELFMMA

SSSLTLLAPFAFILASYGRILRAVLKMRSAEGRKKAFSTCSSHLTVVILS

YGTAISMYMMPQDKASRDRDKIISILYGVLPPMLNPLIYSLRNKDVKGAL

KKLMGKKDVS*

>ModoOR4.10.9

MDGRNNTVVTYFILIGLSEYPRAQVVLFFLLLVSYLVTLLGNVLILFLIH

YDSRLHTPMYFFLSNLSFLDICYTSASVPQMIINCLVTIPIISLGQCLAQ

MCASLYLGVVECLLLAVMAYDRCIAIGDPLRYSVRMGPQLCVQLSVGSWV

SAFLLTVIPTLTTPLEFCGHHIINHFSCELLAVIKLACNDLRYNEYFMMA

SSFLTLLLPFAFILASYGRILRAVLKMHSTKGRKKAFSTCSSHLTVVVIF

YGTAISMYMMPQDKASRDRDKIISMLYGVVTPMLNPLIYSLRNKDVKGAL

QKLCGEKTTT*

>ModoOR4.10.10

MKIGRKQMDRRNNTAVTYFILVGLSEYPRAQMLLFCLLLASYLVILLGNS

LILVLVQFDSRLHTPMYFFLSNLSFLDICFTSSSIPQMIINCLVRMPVIS

AGQCMAQMCTLLYLGVVECLLLAVMAYDRCIAISDPLRYPVRMSNQFCVQ

LAGLSWVSAFLFSIVPTVATPLELCSHVINHFFCEVLAIMKLACSDLQLN

ELLMMATSSLTLLAPFVFILASYGRILGAVLKMRSTEGRKKAFSTCSSHL

TVVVLFYGTAIAMYMMPQDKTSREQDKLASLLYGVVTPMLNPLIYSLRNK

DVKEALRKLLGEKKVP*

>ModoOR4.10.11

MDGRNNTPVTYFILIGLSEYPRAQAIFFCLLLVCYLVILLGNVLILFLIH

YDSRLHTPMYFFLSNLSFLDICYTSASVPQMIINCLVTIPIISLGECLVQ

MCASLYLGVVECLLLAIMAYDRCIAIGDPLRYSVRMGPQLCAQLAGAAWV

SAFLLTVIPTLTTPLEFCGHHIINHFSCELLAVIKLACNDLRYNEYFMMA

SSFLTLLLPFAFILASYGRILRAVLKMHSTKGRKKAFSTCSSHLTVVIIF

YGTAISMYMMPQDKASRDQDKIISMVYLVVTPMLNPLIYSLRNKDVKGAL

QKLLGEKNVP*

>ModoOR4.10.12P

LQPLLFAAVLASYLATVLGNVLLVSLILLDGRLHRPMYRLLAHLALLDVS

YVSSTVPQALAHMLVQRRALPFARCGAQLYVALSLGSTEAILLASMAVDR

CLAVCRPLHYAAIMTPGGVRGSSGWRSGPWASCCPCPTPLPPCAFPTVPG

GPPIDHFLL*TAGSSEGGLVVDSAPKRGPGLCHGRPQSCWGPSDCILASY

DLHPGTQALRLALPPGGGGKALSTCGAHLAVVGLFYGTVMAMYLRPRGPT

PAGRHKLTAVFYIVVTPLLNPLIYSLRNKDVHRAARYAVARLK

>ModoOR4.11.1

MKTNNSQDGFVLLGFSDKPRLETILFVVILVFYILNVVGNMTIILVSCLD

PKLHTPMYFFLTNLSFVDLCLTTCIAPQLLVTMGKKDKIMSYSGCVVQLY

VSMGLGSTECIILAVMSYDRYAAVCRPLHYAVIMHHQLCLSLAATAWLSG

LVTSLIQCSLTIQLPRCGHNQLDHICCEVPVLIKLACVDTTFNEAELFVA

SVVFLVVPVSLILVSYGYIAQAILRIKSATGRQKAFGTCSSHLVVVIIFY

GTIIFMYLQPVKSKSKDQGKFVSLFYTVVTPLLNPLIYTLRNKDVKGALR

KLVLGKSLESEKL*

>ModoOR4.11.2

MEQNDTSQGDFILLGFSDKPQLEIILFAVILVFYILNLVGNMTIILASSM

DPKLHTPMYFFLTNLSFVDLCLTTTVAPQLLFTMRSKDKTMSYNGCVAQL

YVAMGLGSTECILLVVMAYDRYAAVCRPLHYAVIMHPQLCLLLAATAWLS

GLITSLIQCSLTIQLPLCGHNKLDHIFCEVPVLIKLACVDTTFNEDELFV

ASVVFLVVPVSLILISYGYITQAILRIKSAAGRRKAFGTCSSHLVVVIIF

YGTITFMYLQPAKSKSKDQGKFVSLFYTVVTPLLNPLIYTLRNKDVKGAL

RKLILEKTIRSEKL*

>ModoOR4.11.3

MKQNNNSQEGFLLLGFSDKPQLETILFVVILVFYILNLVGNTTIILVSGL

DPKLHTPMYFFLTNLSFLDLCFTTSVAPQLLVTMRSKDKSMSYGGCIAQL

YVAMGLGSTECILLAVMAYDRYAAVCRPLHYAVIMHPQLCLTLASIAWLS

GLVTSLIQCSLTIQLPLCGHNRLDHIFCEVPVLIKLACVDTTFNEAQLFV

ASVVFLVVPVSLILISYGYITQAILKIKSSAGRRKAFGTCSSHLVVVIIF

YGTIIFMYLQPVKSRSNDQGKFVSLFYTIVTPLLNPLIYTLRNKDVKGAL

KKLILGKVIG*

>ModoOR4.11.4

MTNATLVTEFILMGFSEVRELQILHAVLFLLIYLVALLGNLLIIALTLLD

KELHTPMYFFLKHLSFLDLCYISVTVPKFISNTLANISSISFLGCITQVF

LVVSLACAELALLTGMAYDRYVAICHPLHYAVTMNRRTCVKMAASSWLSG

GLSGLLHTATSFSLPFCESTRIHQFFCEIPQILRLSCSESYSAEIGALAI

TSSLSFICFLSISVSYIHIFSTVLKIPSVVGRSKAFSTCMPHLIVVTIFL

HSAAVAYLKPTSACPSFVDLLMSVFYTVLPPTLNPIIYSLRNKDMKSALR

KLVKNHLNGPFEF*

>ModoOR4.11.5P

FFIVLMGNLLIILVSIIDQHHPTALYFLLRHLSFMDLCYISITVPKSSLT

SLTHSNSISVPESIIQIYCFVLLAGSELLVWFTVMSYD**ATICHLLHHE

ALMSRGACGQRA

>ModoOR4.11.6

MGFSDIRELQILYSFLLFLIYCAGLMGNLLIVMITTFDRRLHTPMYFFLR

NLSMVDACYLSITAPQASVNSLVNNRIISVTGCAAQVFLMVFMGYVEFAL

LTVMARDRYVAICHPLHYPVIMSPRVCMQMTLTCLLTGLVYAAFHTGYMF

RLSFCHSNVVHQFFCDIPSLLRISCSDTFSNKLSLFVSVLVVGTGCYTFI

TASYVRIFSTVLKFPVKEDQKKAFSTCIPHISVVSLFVISGAYVYFQPPS

DSGSFEHIISSVFYTIIPPFLNPIICSLR*

>ModoOR4.11.7

MSNFTTATEFLLMGFSDIQELQILYSFLLFLIYSAGLMGNLLIVIITTFD

RTLHTPMYFFLRNLSMLDACYLSITIPQAFFNSLVNNRVISVTGCAAQVF

LVMFMVYVELTLLTVMARDRYVAICHPLHYPVIMSPRVCLQMTITCLLTG

LVYAGFHTGYTFRLSFCHSNVVHQFFCDIPSLLRISCSDTFSNNLSLIVS

DVVVGAGCYAFIIASYVCIFSTVLKFPVKEDQKKAFSTCIPHITVVSLFI

ISGTYVYFHPPSDSGSLKDIILSVFYTVIPPFLNPIIYSLRNKQIKGAIR

ILMKRTL*

>ModoOR4.11.8P

MSNYTTATEFLLMGFSDIQELQILYSFLLFLIYSAGLMGNLLIVIITTID

RRLHTPMYFFLRNLSMVDIFYLSITAPQASVNSLVNNRIISVTGCAAQVF

LVMFIAYVELTLLTVMARDRYVAICHPLHYPVIMSPRVCLQMTITCLLTG

LMYAAFHTGYTFRLSFCHSNVVHQFFCDIPSLLRISCSDTFSNNLSLIVS

DLVVGAGCYAFITASYVRIFSTVLKFPVKEDQKKAFSTCIPHIIVVSLLV

ISGIYVYAQPPSDSGSLKDII*NISILY/VFYTVIPPFMNPIIYSLRNKQ

IKGAIRILMTKTLSLRNKT

>ModoOR4.11.9
[truncated: 2,054,525 more chars]
