# Supplementary material for: Extensive Gains and Losses of Olfactory Receptor Genes in Mammalian Evolution
Source: PLoS One. 2007 Aug 8;2(8):e708. doi: 10.1371/journal.pone.0000708 (PMC1933591; doi:10.1371/journal.pone.0000708)
Supplement: Dataset S2 — Names of functional OR genes belonging to each clade. (0.47 MB DOC) [file pone.0000708.s009.doc]

**Class I**

OranORc4619.3

OranORc4619.4

OranORc5598.1

OranORc5598.3

OranORc6071.1

OranORc6481.2

OranORc6657.1

OranORc6657.3

OranORc7446.1

OranORc8314.1

OranORc9894.1

OranORc10204.3

OranORc11569.1

OranORc11849.1

OranORc12720.1

OranORc14249.1

OranORc15235.1

OranORc16982.1

OranORc20732.1

OranORc23442.1

OranORc23520.1

OranORc25289.1

OranORc26072.1

OranORc28149.1

OranORc28859.1

OranORc29148.1

OranORc32282.1

OranORc33754.1

OranORc38031.1

OranORc39721.1

OranORc46221.1

ModoOR4.5.2

ModoOR4.5.3

ModoOR4.5.4

ModoOR4.5.5

ModoOR4.5.10

ModoOR4.5.11

ModoOR4.5.12

ModoOR4.5.13

ModoOR4.5.14

ModoOR4.5.15

ModoOR4.5.16

ModoOR4.5.17

ModoOR4.5.19

ModoOR4.5.20

ModoOR4.5.21

ModoOR4.5.22

ModoOR4.5.23

ModoOR4.5.24

ModoOR4.5.27

ModoOR4.5.28

ModoOR4.5.29

ModoOR4.5.31

ModoOR4.5.32

ModoOR4.5.33

ModoOR4.5.36

ModoOR4.5.38

ModoOR4.5.39

ModoOR4.5.40

ModoOR4.5.41

ModoOR4.5.42

ModoOR4.5.43

ModoOR4.5.44

ModoOR4.5.45

ModoOR4.5.46

ModoOR4.5.47

ModoOR4.5.48

ModoOR4.5.50

ModoOR4.5.51

ModoOR4.5.52

ModoOR4.5.53

ModoOR4.5.54

ModoOR4.5.55

ModoOR4.5.57

ModoOR4.5.58

ModoOR4.5.59

ModoOR4.5.60

ModoOR4.5.61

ModoOR4.5.62

ModoOR4.5.63

ModoOR4.5.64

ModoOR4.5.65

ModoOR4.5.66

ModoOR4.5.68

ModoOR4.12.2

ModoORUn.1.1

ModoORUn.1.3

ModoORUn.1.4

ModoORUn.1.5

ModoORUn.1.6

ModoORUn.1.7

ModoORUn.1.8

ModoORUn.1.9

ModoORUn.1.10

ModoORUn.1.11

ModoORUn.1.12

ModoORUn.1.13

ModoORUn.1.14

ModoORUn.1.15

ModoORUn.1.16

ModoORUn.1.17

ModoORUn.1.18

ModoORUn.1.19

ModoORUn.1.20

ModoORUn.1.21

ModoORUn.1.22

ModoORUn.1.23

ModoORUn.1.24

ModoORUn.1.25

ModoORUn.1.26

ModoORUn.1.27

ModoORUn.1.28

ModoORUn.1.29

ModoORUn.1.30

ModoORUn.1.31

ModoORUn.1.32

ModoORUn.1.33

ModoORUn.1.34

ModoORUn.1.36

ModoORUn.1.38

ModoORUn.1.39

ModoORUn.1.40

ModoORUn.1.41

ModoORUn.1.42

ModoORUn.1.43

ModoORUn.1.44

ModoORUn.1.45

ModoORUn.1.46

ModoORUn.1.47

ModoORUn.1.48

ModoORUn.1.49

ModoORUn.1.50

ModoORUn.1.51

ModoORUn.1.52

ModoORUn.1.53

ModoORUn.1.54

ModoORUn.1.55

ModoORUn.1.56

ModoORUn.1.57

ModoORUn.1.58

ModoORUn.1.60

ModoORUn.1.62

ModoORUn.1.64

ModoORUn.1.65

ModoORUn.1.66

ModoORUn.1.67

ModoORUn.1.68

ModoORUn.1.69

ModoORUn.1.70

ModoORUn.1.71

ModoORUn.1.72

ModoORUn.1.73

ModoORUn.1.74

ModoORUn.1.75

ModoORUn.1.76

ModoORUn.1.77

ModoORUn.1.78

ModoORUn.1.79

ModoORUn.1.80

ModoORUn.1.81

ModoORUn.1.82

ModoORUn.1.83

ModoORUn.1.84

ModoORUn.1.85

ModoORUn.1.86

ModoORUn.1.87

ModoORUn.1.88

ModoORUn.1.90

ModoORUn.1.91

ModoORUn.1.92

ModoORUn.1.93

ModoORUn.1.95

ModoORUn.1.96

ModoORUn.1.97

ModoORUn.1.98

ModoORUn.1.99

ModoORUn.1.100

ModoORUn.1.101

ModoORUn.1.102

ModoORUn.1.103

ModoORUn.1.104

ModoORUn.1.105

ModoORUn.1.106

ModoORUn.1.107

ModoORUn.1.108

ModoORUn.1.109

ModoORUn.1.110

ModoORUn.1.111

ModoORUn.1.112

ModoORUn.1.113

ModoORUn.1.115

ModoORUn.1.116

ModoORUn.1.117

ModoORUn.1.118

ModoORUn.1.119

ModoORUn.1.120

ModoORUn.1.121

ModoORUn.1.123

ModoORUn.1.124

ModoORUn.1.126

ModoORUn.1.127

ModoORUn.1.128

ModoORUn.1.129

ModoORUn.1.130

ModoORUn.1.131

ModoORUn.1.132

ModoORUn.1.134

ModoORUn.1.135

ModoORUn.1.136

ModoORUn.1.137

ModoORUn.1.138

ModoORUn.1.139

ModoORUn.1.141

ModoORUn.1.142

ModoORUn.1.145

ModoORUn.1.146

ModoORUn.1.148

ModoORUn.1.149

ModoORUn.1.150

ModoORUn.1.151

ModoORUn.1.152

ModoORUn.1.154

ModoORUn.1.155

ModoORUn.1.156

ModoORUn.1.157

ModoORUn.1.158

ModoORUn.1.159

ModoORUn.1.160

ModoORUn.1.161

ModoORUn.1.162

ModoORUn.1.165

ModoORUn.1.166

ModoORUn.1.167

ModoORUn.1.168

ModoORUn.1.169

ModoORUn.1.170

ModoORUn.1.171

ModoORUn.1.172

ModoORUn.1.175

ModoORUn.1.176

ModoORUn.1.180

ModoORUn.1.181

ModoORUn.1.182

ModoORUn.1.183

ModoORUn.1.184

ModoORUn.1.185

ModoORUn.1.186

ModoORUn.1.187

ModoORUn.1.188

ModoORUn.1.189

ModoORUn.1.190

ModoORUn.18.2

BotaOR13.1.2

BotaOR13.1.3

BotaOR13.1.4

BotaOR15.1.1

BotaOR15.1.3

BotaOR15.1.4

BotaOR15.1.5

BotaOR15.1.6

BotaOR15.1.9

BotaOR15.1.10

BotaOR15.1.11

BotaOR15.1.12

BotaOR15.1.13

BotaOR15.1.15

BotaOR15.1.16

BotaOR15.1.17

BotaOR15.1.18

BotaOR15.1.20

BotaOR15.1.23

BotaOR15.1.28

BotaOR15.1.29

BotaOR15.1.30

BotaOR15.1.32

BotaOR15.1.36

BotaOR15.1.37

BotaOR15.1.40

BotaOR15.1.41

BotaOR15.1.44

BotaOR15.1.45

BotaOR15.1.46

BotaOR15.1.48

BotaOR15.1.49

BotaOR15.1.51

BotaOR15.1.52

BotaOR15.1.53

BotaOR15.1.54

BotaOR15.1.55

BotaOR15.1.56

BotaOR15.1.57

BotaOR15.1.58

BotaOR15.1.59

BotaOR15.1.61

BotaOR15.1.62

BotaOR15.1.63

BotaOR15.1.65

BotaOR15.2.1

BotaOR15.2.2

BotaOR15.2.3

BotaOR15.2.6

BotaOR21.1.1

BotaOR21.1.2

BotaOR21.1.3

BotaOR21.1.5

BotaOR21.1.6

BotaOR21.1.7

BotaOR21.1.9

BotaOR21.1.10

BotaOR21.1.11

BotaORs983.1

BotaORs983.2

BotaORs983.5

BotaORs983.7

BotaORs983.8

BotaORs983.9

BotaORs983.12

BotaORs983.14

BotaORs983.15

BotaORs984.2

BotaORs1162.1

BotaORs1162.3

BotaORs1369.2

BotaORs1369.3

BotaORs1369.4

BotaORs1369.6

BotaORs1369.7

BotaORs1369.8

BotaORs1369.11

BotaORs1369.12

BotaORs1386.1

BotaORs1595.1

BotaORs1595.3

BotaORs1595.4

BotaORs1595.5

BotaORs1595.6

BotaORs1595.8

BotaORs1886.2

BotaORs2822.1

BotaORs2822.2

BotaORs2822.3

BotaORs2822.4

BotaORs2822.5

BotaORs2822.6

BotaORs4765.1

BotaORs4765.2

BotaORs5579.1

BotaORs5579.2

BotaORs5579.3

BotaORs5579.4

BotaORs5751.4

BotaORs5863.2

BotaORs5992.1

BotaORs5992.2

BotaORs5992.3

BotaORs6098.2

BotaORs6098.5

BotaORs6287.1

BotaORs6811.3

BotaORs7386.1

BotaORs7386.3

BotaORs7576.1

BotaORs7576.4

BotaORs7786.1

BotaORs7786.2

BotaORs8259.1

BotaORs8269.2

BotaORs8466.1

BotaORs8529.1

BotaORs8602.1

BotaORs8720.2

BotaORs9459.1

BotaORs9459.2

BotaORs9561.1

BotaORs9608.2

BotaORs9608.3

BotaORs9858.1

BotaORs9938.2

BotaORs10173.2

BotaORs10353.2

BotaORs10655.2

BotaORs11394.2

BotaORs11629.1

BotaORs11679.1

BotaORs11784.1

BotaORs11917.1

BotaORs12103.1

BotaORs12595.1

BotaORs12596.1

BotaORs12596.2

BotaORs13758.1

BotaORs13758.2

BotaORs14074.1

BotaORs18420.1

CafaOR21.2.1

CafaOR21.2.3

CafaOR21.2.4

CafaOR21.2.5

CafaOR21.2.6

CafaOR21.2.7

CafaOR21.2.8

CafaOR21.2.9

CafaOR21.2.10

CafaOR21.2.11

CafaOR21.2.13

CafaOR21.2.14

CafaOR21.2.15

CafaOR21.2.16

CafaOR21.2.17

CafaOR21.2.18

CafaOR21.2.19

CafaOR21.2.20

CafaOR21.2.22

CafaOR21.2.23

CafaOR21.2.27

CafaOR21.2.28

CafaOR21.2.29

CafaOR21.2.30

CafaOR21.2.31

CafaOR21.2.32

CafaOR21.2.33

CafaOR21.2.34

CafaOR21.2.35

CafaOR21.2.36

CafaOR21.2.38

CafaOR21.2.39

CafaOR21.2.40

CafaOR21.2.43

CafaOR21.2.44

CafaOR21.2.45

CafaOR21.2.50

CafaOR21.2.51

CafaOR21.2.53

CafaOR21.2.54

CafaOR21.2.55

CafaOR21.2.56

CafaOR21.2.57

CafaOR21.2.58

CafaOR21.2.60

CafaOR21.2.61

CafaOR21.2.62

CafaOR21.2.64

CafaOR21.2.65

CafaOR21.2.66

CafaOR21.2.67

CafaOR21.2.68

CafaOR21.2.69

CafaOR21.2.70

CafaOR21.2.72

CafaOR21.2.73

CafaOR21.2.74

CafaOR21.2.75

CafaOR21.2.76

CafaOR21.2.79

CafaOR21.2.80

CafaOR21.2.81

CafaOR21.2.82

CafaOR21.2.83

CafaOR21.2.85

CafaOR21.2.87

CafaOR21.2.88

CafaOR21.2.89

CafaOR21.2.90

CafaOR21.2.92

CafaOR21.2.94

CafaOR21.2.95

CafaOR21.2.96

CafaOR21.2.98

CafaOR21.2.99

CafaOR21.2.102

CafaOR21.2.103

CafaOR21.2.104

CafaOR21.2.107

CafaOR21.2.108

CafaOR21.2.110

CafaOR21.2.111

CafaOR21.2.112

CafaOR21.2.113

CafaOR21.2.115

CafaOR21.2.116

CafaOR21.2.117

CafaOR21.2.118

CafaOR21.2.119

CafaOR21.2.120

CafaOR21.2.121

CafaOR21.2.122

CafaOR21.2.123

CafaOR21.2.124

CafaOR21.2.125

CafaOR21.2.126

CafaOR21.2.127

CafaOR21.2.128

CafaOR21.2.129

CafaOR21.2.130

CafaOR21.2.131

CafaOR21.2.132

CafaOR21.2.133

CafaOR21.2.135

CafaOR21.2.136

CafaOR21.2.137

CafaOR21.2.138

CafaOR21.2.140

CafaOR21.2.141

CafaOR21.2.143

CafaOR21.2.144

CafaOR21.2.145

CafaOR21.2.146

CafaOR21.2.147

CafaOR21.2.148

CafaOR21.2.149

CafaOR21.2.150

CafaOR21.2.151

CafaOR21.2.152

CafaOR21.2.153

CafaOR21.2.154

CafaOR21.2.156

CafaOR21.2.157

CafaOR21.2.158

CafaOR21.2.159

CafaOR21.2.160

CafaOR21.2.161

CafaOR21.2.162

CafaOR21.2.165

CafaOR21.2.166

CafaOR21.2.167

CafaOR21.2.168

CafaOR21.2.169

CafaOR21.2.170

CafaOR21.2.171

CafaOR21.2.172

CafaOR21.2.173

CafaOR21.2.175

CafaOR21.2.176

CafaOR21.2.179

CafaOR21.2.180

CafaOR21.2.181

CafaOR21.2.182

CafaOR21.2.183

CafaOR21.2.186

CafaOR21.2.187

CafaOR21.2.188

CafaOR21.2.189

CafaOR21.2.190

CafaOR21.2.191

CafaOR21.2.192

CafaOR21.2.193

CafaOR21.2.198

CafaOR21.2.199

CafaORUn.4.4

CafaORUn.4.8

CafaORUn.4.9

CafaORUn.6.3

CafaORUn.6.4

CafaORUn.6.5

MmOR7.5.1

MmOR7.5.2

MmOR7.5.3

MmOR7.5.4

MmOR7.5.5

MmOR7.5.7

MmOR7.5.8

MmOR7.5.9

MmOR7.5.10

MmOR7.5.11

MmOR7.5.12

MmOR7.5.13

MmOR7.5.14

MmOR7.5.15

MmOR7.5.16

MmOR7.5.17

MmOR7.5.18

MmOR7.5.19

MmOR7.5.20

MmOR7.5.24

MmOR7.5.27

MmOR7.5.28

MmOR7.5.29

MmOR7.5.30

MmOR7.5.33

MmOR7.5.34

MmOR7.5.35

MmOR7.5.36

MmOR7.5.42

MmOR7.5.43

MmOR7.5.44

MmOR7.5.47

MmOR7.5.48

MmOR7.5.50

MmOR7.5.51

MmOR7.5.52

MmOR7.5.53

MmOR7.5.56

MmOR7.5.57

MmOR7.5.58

MmOR7.5.59

MmOR7.5.60

MmOR7.5.62

MmOR7.5.63

MmOR7.5.64

MmOR7.5.66

MmOR7.5.67

MmOR7.5.68

MmOR7.5.69

MmOR7.5.71

MmOR7.5.72

MmOR7.5.73

MmOR7.5.74

MmOR7.5.75

MmOR7.5.76

MmOR7.5.77

MmOR7.5.79

MmOR7.5.81

MmOR7.5.85

MmOR7.5.86

MmOR7.5.87

MmOR7.5.88

MmOR7.5.89

MmOR7.5.92

MmOR7.5.93

MmOR7.5.96

MmOR7.5.97

MmOR7.5.99

MmOR7.5.100

MmOR7.5.103

MmOR7.5.104

MmOR7.5.105

MmOR7.5.106

MmOR7.5.107

MmOR7.5.108

MmOR7.5.109

MmOR7.5.110

MmOR7.5.112

MmOR7.5.113

MmOR7.5.115

MmOR7.5.116

MmOR7.5.117

MmOR7.5.118

MmOR7.5.119

MmOR7.5.120

MmOR7.5.121

MmOR7.5.122

MmOR7.5.123

MmOR7.5.125

MmOR7.5.127

MmOR7.5.129

MmOR7.5.131

MmOR7.5.132

MmOR7.5.133

MmOR7.5.134

MmOR7.5.136

MmOR7.5.140

MmOR7.5.141

MmOR7.5.142

MmOR7.5.143

MmOR7.5.146

MmOR7.5.149

MmOR7.5.150

MmOR7.5.151

MmOR7.5.152

MmOR7.5.154

MmOR7.5.155

MmOR7.5.156

MmOR7.5.157

MmOR7.5.158

MmOR7.7.31

MmOR7.7.32

MmORUn.18.1

RanoOR1.7.2

RanoOR1.7.3

RanoOR1.7.4

RanoOR1.7.6

RanoOR1.7.7

RanoOR1.7.8

RanoOR1.7.9

RanoOR1.7.10

RanoOR1.7.11

RanoOR1.7.12

RanoOR1.7.13

RanoOR1.7.14

RanoOR1.7.16

RanoOR1.7.18

RanoOR1.7.19

RanoOR1.7.21

RanoOR1.7.22

RanoOR1.7.23

RanoOR1.7.24

RanoOR1.7.25

RanoOR1.7.28

RanoOR1.7.29

RanoOR1.7.30

RanoOR1.7.31

RanoOR1.7.32

RanoOR1.7.34

RanoOR1.7.36

RanoOR1.7.37

RanoOR1.7.40

RanoOR1.7.41

RanoOR1.7.42

RanoOR1.7.43

RanoOR1.7.44

RanoOR1.7.45

RanoOR1.7.46

RanoOR1.7.47

RanoOR1.7.48

RanoOR1.7.49

RanoOR1.7.50

RanoOR1.7.51

RanoOR1.7.52

RanoOR1.7.53

RanoOR1.7.54

RanoOR1.7.55

RanoOR1.7.56

RanoOR1.7.57

RanoOR1.7.58

RanoOR1.7.59

RanoOR1.7.61

RanoOR1.7.63

RanoOR1.7.65

RanoOR1.7.66

RanoOR1.7.67

RanoOR1.7.68

RanoOR1.7.69

RanoOR1.7.70

RanoOR1.7.71

RanoOR1.7.72

RanoOR1.7.73

RanoOR1.7.74

RanoOR1.7.75

RanoOR1.7.76

RanoOR1.7.77

RanoOR1.7.81

RanoOR1.7.82

RanoOR1.7.83

RanoOR1.7.84

RanoOR1.7.86

RanoOR1.7.87

RanoOR1.7.88

RanoOR1.7.89

RanoOR1.7.90

RanoOR1.7.91

RanoOR1.7.92

RanoOR1.7.93

RanoOR1.7.94

RanoOR1.7.96

RanoOR1.7.97

RanoOR1.7.98

RanoOR1.7.100

RanoOR1.7.101

RanoOR1.7.102

RanoOR1.7.103

RanoOR1.7.104

RanoOR1.7.105

RanoOR1.7.106

RanoOR1.7.109

RanoOR1.7.110

RanoOR1.7.111

RanoOR1.7.113

RanoOR1.7.114

RanoOR1.7.115

RanoOR1.7.116

RanoOR1.7.117

RanoOR1.7.118

RanoOR1.7.119

RanoOR1.7.120

RanoOR1.7.121

RanoOR1.7.122

RanoOR1.7.123

RanoOR1.7.124

RanoOR1.7.125

RanoOR1.7.126

RanoOR1.7.128

RanoOR1.7.129

RanoOR1.7.131

RanoOR1.7.132

RanoOR1.7.133

RanoOR1.7.136

RanoOR1.7.137

RanoOR1.7.138

RanoOR1.7.139

RanoOR1.7.140

RanoOR1.7.141

RanoOR1.7.142

RanoOR1.7.143

RanoOR1.7.145

RanoOR1.7.146

RanoOR1.7.147

RanoOR1.7.148

RanoOR1.7.150

RanoOR1.7.151

RanoOR1.7.152

RanoOR1.7.155

RanoOR1.7.156

RanoOR1.7.157

RanoOR1.7.159

RanoOR1.7.160

RanoOR1.7.161

RanoOR1.7.162

RanoOR1.7.163

RanoOR1.7.164

RanoOR1.7.165

RanoOR1.12.1

MamuOR8.1.1

MamuOR14.9.1

MamuOR14.9.2

MamuOR14.9.6

MamuOR14.9.7

MamuOR14.9.12

MamuOR14.10.6

MamuOR14.10.7

MamuOR14.10.9

MamuOR14.10.11

MamuOR14.10.14

MamuOR14.10.15

MamuOR14.10.26

MamuOR14.10.29

MamuOR14.10.32

MamuOR14.10.34

MamuOR14.10.35

MamuOR14.10.37

MamuOR14.10.46

MamuOR14.10.48

MamuOR14.10.52

MamuOR14.10.53

MamuOR14.10.54

MamuOR14.10.58

MamuOR14.10.59

MamuOR14.10.62

MamuOR14.10.66

MamuOR14.10.68

MamuOR14.10.69

MamuOR14.10.74

MamuOR14.10.85

MamuOR14.10.88

MamuOR14.10.91

MamuOR14.10.93

MamuOR14.10.94

MamuOR14.10.95

HsOR11.3.2

HsOR11.3.6

HsOR11.3.8

HsOR11.3.10

HsOR11.3.11

HsOR11.3.12

HsOR11.3.13

HsOR11.3.14

HsOR11.3.16

HsOR11.3.18

HsOR11.3.22

HsOR11.3.24

HsOR11.3.25

HsOR11.3.27

HsOR11.3.28

HsOR11.3.30

HsOR11.3.33

HsOR11.3.34

HsOR11.3.35

HsOR11.3.37

HsOR11.3.38

HsOR11.3.40

HsOR11.3.43

HsOR11.3.44

HsOR11.3.49

HsOR11.3.50

HsOR11.3.51

HsOR11.3.54

HsOR11.3.55

HsOR11.3.57

HsOR11.3.59

HsOR11.3.60

HsOR11.3.61

HsOR11.3.63

HsOR11.3.65

HsOR11.3.66

HsOR11.3.68

HsOR11.3.70

HsOR11.3.74

HsOR11.3.77

HsOR11.3.78

HsOR11.3.79

HsOR11.3.80

HsOR11.3.81

HsOR11.3.83

HsOR11.3.84

HsOR11.3.85

HsOR11.3.87

HsOR11.3.88

HsOR11.3.90

HsOR11.3.91

HsOR11.3.92

HsOR11.3.93

HsOR11.3.94

HsOR11.3.96

HsOR11.3.98

HsOR11.3.101

HsOR11.3.102

**Class II**

CladeA:

OranORc588.2

OranORc6057.1

OranORc6057.2

OranORc6057.3

OranORc6621.1

OranORc6621.2

OranORc6621.4

OranORc13954.1

OranORc14135.1

OranORc14771.1

OranORc17617.1

OranORuc285.71

OranORuc285.74

OranORuc285.76

OranORuc285.77

OranORuc285.78

OranORuc285.79

OranORuc285.80

OranORuc285.81

OranORuc285.82

OranORuc285.83

OranORuc285.84

OranORuc285.86

OranORuc285.87

OranORuc285.88

OranORuc285.89

OranORuc285.90

OranORuc285.92

OranORuc285.93

OranORuc285.94

OranORuc285.95

OranORuc285.96

OranORuc435.8

OranORuc435.9

OranORuc435.11

ModoOR1.2.1

ModoOR1.2.2

ModoOR1.2.3

ModoOR1.2.4

ModoOR1.2.5

ModoOR1.2.6

ModoOR1.2.7

ModoOR1.2.8

ModoOR1.2.9

ModoOR1.2.10

ModoOR1.2.11

ModoOR1.2.12

ModoOR1.2.13

ModoOR1.2.14

ModoOR1.2.15

ModoOR1.2.16

ModoOR1.2.17

ModoOR1.2.19

ModoOR1.2.21

ModoOR1.2.22

ModoOR1.2.23

ModoOR1.2.24

ModoOR1.2.25

ModoOR1.2.27

ModoOR1.2.28

ModoOR1.2.29

ModoOR1.2.31

ModoOR1.2.32

ModoOR1.2.33

ModoOR1.2.35

ModoOR1.2.36

ModoOR1.2.38

ModoOR1.2.39

ModoOR1.2.40

ModoOR1.2.41

ModoOR1.2.42

ModoOR1.2.43

ModoOR1.2.44

ModoOR1.2.45

ModoOR1.2.46

ModoOR1.2.47

ModoOR1.2.48

ModoOR1.2.49

ModoOR1.2.50

ModoOR1.2.51

ModoOR1.2.52

ModoOR1.2.53

ModoOR1.2.54

ModoOR1.2.55

ModoOR1.4.8

ModoOR1.4.9

ModoOR2.2.1

ModoOR2.2.2

ModoOR4.2.37

ModoOR5.4.1

ModoOR5.4.3

ModoOR5.4.4

ModoOR5.4.5

ModoOR5.4.7

ModoOR5.4.9

ModoOR5.4.10

ModoOR5.4.11

ModoOR5.4.12

ModoOR5.4.13

ModoOR5.4.14

ModoOR5.4.15

ModoOR5.5.1

ModoOR5.5.2

ModoOR5.5.3

ModoOR5.5.4

ModoOR5.5.6

ModoOR5.5.7

ModoOR5.5.8

ModoOR5.5.9

ModoOR5.5.10

ModoOR5.5.12

ModoOR5.5.13

ModoOR5.5.14

ModoOR5.5.16

ModoOR5.5.17

ModoOR5.5.18

ModoOR5.5.19

ModoOR5.5.20

ModoOR5.5.23

ModoOR5.5.24

ModoOR5.5.25

ModoOR5.5.28

ModoOR5.5.29

ModoOR5.5.30

ModoOR5.5.31

ModoOR5.5.32

ModoOR5.5.33

ModoOR5.5.34

ModoOR5.5.35

ModoOR5.5.36

ModoOR5.5.37

ModoOR5.5.38

ModoOR5.5.39

ModoOR5.5.40

ModoOR5.5.41

ModoOR5.5.42

ModoOR5.5.43

ModoOR5.5.44

ModoOR5.5.45

ModoOR5.5.46

ModoOR5.5.47

ModoOR5.5.48

ModoOR5.5.49

ModoOR5.5.50

ModoOR5.5.52

ModoOR5.5.53

ModoOR5.5.54

ModoOR5.5.55

ModoOR5.5.56

ModoOR5.5.57

ModoOR5.5.58

ModoOR5.5.59

ModoOR5.5.105

ModoOR5.7.21

ModoOR5.7.22

ModoOR5.7.23

ModoOR5.7.24

ModoOR5.7.25

ModoORUn.8.1

ModoORUn.8.2

ModoORUn.8.3

ModoORUn.8.4

BotaOR5.1.2

BotaOR5.1.6

BotaOR7.8.3

BotaOR7.8.7

BotaOR7.8.8

BotaOR7.8.9

BotaOR7.8.10

BotaOR10.4.1

BotaOR10.4.2

BotaOR10.4.3

BotaOR10.4.4

BotaOR10.4.5

BotaOR10.4.6

BotaOR10.4.7

BotaOR10.4.8

BotaOR10.4.9

BotaOR10.4.10

BotaOR10.4.11

BotaOR10.4.13

BotaOR10.4.14

BotaOR10.4.15

BotaOR10.4.16

BotaOR10.4.18

BotaOR10.4.20

BotaOR10.4.22

BotaOR10.4.23

BotaOR10.4.24

BotaOR10.4.25

BotaOR10.4.26

BotaOR15.4.3

BotaOR15.5.1

BotaOR15.5.2

BotaOR15.5.3

BotaOR15.5.4

BotaOR15.5.5

BotaOR15.7.4

BotaOR15.7.6

BotaOR15.7.8

BotaOR15.7.9

BotaOR15.7.10

BotaOR15.7.11

BotaOR19.1.2

BotaOR25.5.1

BotaOR25.5.2

BotaOR25.5.3

BotaOR29.1.6

BotaORs218.3

BotaORs218.4

BotaORs218.6

BotaORs218.11

BotaORs1157.1

BotaORs1157.4

BotaORs1157.6

BotaORs1157.7

BotaORs1157.8

BotaORs1157.9

BotaORs1157.10

BotaORs1157.11

BotaORs1225.2

BotaORs1225.3

BotaORs4140.1

BotaORs4140.2

BotaORs4140.6

BotaORs4786.1

BotaORs4786.3

BotaORs4786.6

BotaORs4927.1

BotaORs4927.3

BotaORs5009.1

BotaORs5009.2

BotaORs5009.3

BotaORs5009.4

BotaORs5009.5

BotaORs5655.1

BotaORs5655.2

BotaORs5655.3

BotaORs6160.1

BotaORs6940.1

BotaORs6940.2

BotaORs6940.3

BotaORs6962.2

BotaORs7569.2

BotaORs7613.3

BotaORs7668.1

BotaORs7668.3

BotaORs8512.2

BotaORs8711.2

BotaORs8711.3

BotaORs8730.1

BotaORs8997.1

BotaORs8997.2

BotaORs9237.1

BotaORs9531.1

BotaORs9569.1

BotaORs9700.1

BotaORs10560.2

BotaORs11445.2

BotaORs11847.1

BotaORs11859.1

BotaORs12383.1

BotaORs12434.1

BotaORs12462.1

BotaORs12462.2

BotaORs13222.1

BotaORs13397.1

BotaORs13397.2

BotaORs16229.1

BotaORs18830.1

BotaORs20906.1

BotaORs30502.1

BotaORs55101.1

CafaOR2.1.4

CafaOR5.2.42

CafaOR8.1.13

CafaOR9.1.1

CafaOR9.1.2

CafaOR9.1.3

CafaOR10.2.12

CafaOR15.2.1

CafaOR15.2.2

CafaOR15.2.3

CafaOR15.2.4

CafaOR15.2.5

CafaOR15.2.6

CafaOR15.2.8

CafaOR15.3.1

CafaOR15.3.2

CafaOR18.3.6

CafaOR18.3.7

CafaOR18.3.8

CafaOR18.3.9

CafaOR18.3.10

CafaOR18.3.11

CafaOR18.4.92

CafaOR18.4.93

CafaOR18.4.94

CafaOR18.4.95

CafaOR18.4.96

CafaOR18.4.97

CafaOR18.4.99

CafaOR18.4.100

CafaOR18.4.101

CafaOR18.4.102

CafaOR18.4.104

CafaOR18.4.105

CafaOR18.4.106

CafaOR18.4.107

CafaOR18.4.110

CafaOR18.4.111

CafaOR18.4.112

CafaOR18.4.115

CafaOR18.4.116

CafaOR18.4.118

CafaOR18.4.119

CafaOR18.4.120

CafaOR18.4.121

CafaOR18.4.122

CafaOR18.4.123

CafaOR18.4.125

CafaOR18.4.126

CafaOR18.4.127

CafaOR18.4.128

CafaOR18.4.129

CafaOR18.4.130

CafaOR18.4.131

CafaOR18.4.132

CafaOR18.4.133

CafaOR18.4.134

CafaOR18.4.136

CafaOR18.4.137

CafaOR18.4.138

CafaOR18.4.139

CafaOR18.4.140

CafaOR18.4.141

CafaOR18.4.142

CafaOR18.4.143

CafaOR18.4.144

CafaOR18.4.145

CafaOR18.4.146

CafaOR18.4.147

CafaOR18.4.148

CafaOR18.4.150

CafaOR18.4.151

CafaOR18.4.152

CafaOR18.4.153

CafaOR18.4.154

CafaOR18.4.155

CafaOR18.4.160

CafaOR18.4.161

CafaOR18.4.162

CafaOR18.4.163

CafaOR18.4.165

CafaOR18.4.166

CafaOR18.4.168

CafaOR18.4.169

CafaOR18.4.170

CafaOR18.4.171

CafaOR18.4.172

CafaOR18.4.173

CafaOR18.4.174

CafaOR30.1.1

CafaOR30.1.2

CafaOR30.1.3

CafaOR30.1.4

CafaOR30.1.5

CafaOR30.1.12

CafaOR30.1.14

CafaOR30.1.17

CafaOR30.1.18

CafaOR30.1.19

CafaOR30.1.20

CafaOR30.1.22

CafaOR30.1.24

CafaOR30.1.26

CafaOR30.1.28

CafaOR30.1.29

CafaOR30.1.30

CafaORUn.1.1

CafaORUn.5.1

CafaORUn.5.4

CafaORUn.8.1

CafaORUn.8.2

CafaORUn.11.2

CafaORUn.14.3

CafaORUn.21.2

MmOR2.2.176

MmOR2.2.177

MmOR2.2.178

MmOR2.2.179

MmOR2.2.181

MmOR2.2.182

MmOR2.2.183

MmOR2.2.184

MmOR2.2.185

MmOR2.2.186

MmOR2.2.188

MmOR2.2.189

MmOR2.2.190

MmOR2.2.191

MmOR2.2.192

MmOR2.2.193

MmOR2.2.194

MmOR2.2.195

MmOR2.2.196

MmOR2.2.197

MmOR2.2.198

MmOR2.2.199

MmOR2.2.200

MmOR2.2.201

MmOR2.2.202

MmOR2.2.203

MmOR2.2.204

MmOR2.2.205

MmOR2.2.206

MmOR2.2.207

MmOR2.2.208

MmOR2.2.209

MmOR2.2.210

MmOR2.2.211

MmOR2.2.212

MmOR2.2.213

MmOR2.2.214

MmOR2.2.215

MmOR2.2.216

MmOR2.2.217

MmOR2.2.218

MmOR2.2.219

MmOR2.2.220

MmOR2.2.221

MmOR2.2.222

MmOR2.2.223

MmOR2.2.224

MmOR2.2.225

MmOR2.2.226

MmOR2.2.228

MmOR2.2.229

MmOR2.2.230

MmOR2.2.231

MmOR2.2.232

MmOR2.2.233

MmOR2.2.234

MmOR2.2.235

MmOR2.2.236

MmOR2.2.237

MmOR2.2.238

MmOR2.2.239

MmOR2.2.240

MmOR2.2.241

MmOR2.2.242

MmOR2.2.243

MmOR2.2.244

MmOR2.2.245

MmOR2.2.246

MmOR2.2.247

MmOR2.2.248

MmOR2.2.249

MmOR2.2.250

MmOR2.2.251

MmOR2.2.252

MmOR2.2.253

MmOR2.2.254

MmOR2.2.256

MmOR2.2.257

MmOR2.2.258

MmOR2.2.261

MmOR2.2.263

MmOR2.2.264

MmOR2.2.265

MmOR2.2.267

MmOR2.3.1

MmOR2.3.2

MmOR2.3.3

MmOR2.3.4

MmOR2.3.5

MmOR2.3.6

MmOR2.3.7

MmOR2.3.8

MmOR2.3.9

MmOR2.3.10

MmOR2.3.12

MmOR2.3.13

MmOR2.3.14

MmOR2.3.15

MmOR2.3.16

MmOR2.3.20

MmOR2.3.21

MmOR2.3.23

MmOR2.3.24

MmOR2.3.25

MmOR2.3.27

MmOR2.3.28

MmOR2.3.29

MmOR2.3.32

MmOR2.3.35

MmOR2.3.36

MmOR2.3.37

MmOR2.3.38

MmOR2.3.39

MmOR2.3.40

MmOR2.3.41

MmOR2.3.42

MmOR2.3.43

MmOR2.3.44

MmOR2.3.45

MmOR9.3.116

MmOR11.7.1

MmOR11.7.2

MmOR11.7.3

MmOR14.2.1

MmOR14.2.2

MmOR14.2.3

MmOR14.2.5

MmOR14.2.6

MmOR14.2.7

MmOR14.2.8

MmOR14.2.9

MmOR14.2.10

MmOR14.2.11

MmOR14.2.12

MmOR14.2.13

MmOR14.2.14

MmOR14.3.6

MmOR14.3.7

MmOR14.3.8

MmOR19.1.6

MmOR19.1.7

MmOR19.1.8

MmOR19.1.9

MmOR19.1.10

MmOR19.1.11

RanoOR1.11.6

RanoOR1.11.7

RanoOR1.11.8

RanoOR1.11.9

RanoOR1.11.10

RanoOR3.3.172

RanoOR3.3.174

RanoOR3.3.176

RanoOR3.3.177

RanoOR3.3.178

RanoOR3.3.182

RanoOR3.3.183

RanoOR3.3.184

RanoOR3.3.186

RanoOR3.3.187

RanoOR3.3.188

RanoOR3.3.189

RanoOR3.3.190

RanoOR3.3.192

RanoOR3.3.193

RanoOR3.3.194

RanoOR3.3.198

RanoOR3.3.200

RanoOR3.3.201

RanoOR3.3.202

RanoOR3.3.203

RanoOR3.3.204

RanoOR3.3.205

RanoOR3.3.206

RanoOR3.3.207

RanoOR3.3.209

RanoOR3.3.210

RanoOR3.3.211

RanoOR3.3.212

RanoOR3.3.213

RanoOR3.3.214

RanoOR3.3.215

RanoOR3.3.216

RanoOR3.3.217

RanoOR3.3.218

RanoOR3.3.221

RanoOR3.3.222

RanoOR3.3.223

RanoOR3.3.224

RanoOR3.3.225

RanoOR3.3.226

RanoOR3.3.227

RanoOR3.3.228

RanoOR3.3.230

RanoOR3.3.231

RanoOR3.3.232

RanoOR3.3.234

RanoOR3.3.236

RanoOR3.3.237

RanoOR3.3.238

RanoOR3.3.239

RanoOR3.3.240

RanoOR3.3.241

RanoOR3.3.243

RanoOR3.3.245

RanoOR3.3.246

RanoOR3.3.247

RanoOR3.3.248

RanoOR3.3.250

RanoOR3.3.252

RanoOR3.3.253

RanoOR3.3.254

RanoOR3.3.255

RanoOR3.3.257

RanoOR3.3.258

RanoOR3.3.259

RanoOR3.3.260

RanoOR3.3.261

RanoOR3.3.262

RanoOR3.3.263

RanoOR3.3.264

RanoOR3.3.265

RanoOR3.3.266

RanoOR3.3.268

RanoOR3.3.269

RanoOR3.3.272

RanoOR3.3.273

RanoOR3.3.274

RanoOR3.3.275

RanoOR3.3.276

RanoOR3.3.277

RanoOR3.3.279

RanoOR3.3.280

RanoOR3.3.281

RanoOR3.3.282

RanoOR3.3.283

RanoOR3.3.284

RanoOR3.3.287

RanoOR3.3.288

RanoOR3.3.290

RanoOR3.3.291

RanoOR3.5.1

RanoOR3.5.2

RanoOR3.5.3

RanoOR3.5.4

RanoOR3.5.6

RanoOR3.5.7

RanoOR3.5.8

RanoOR3.5.11

RanoOR3.5.12

RanoOR3.5.13

RanoOR3.5.15

RanoOR3.5.17

RanoOR3.5.21

RanoOR3.5.22

RanoOR3.5.23

RanoOR3.5.24

RanoOR3.5.25

RanoOR3.5.26

RanoOR3.5.27

RanoOR3.5.28

RanoOR3.5.29

RanoOR3.5.30

RanoOR3.5.31

RanoOR3.5.32

RanoOR3.5.33

RanoOR3.5.34

RanoOR3.5.35

RanoOR3.5.36

RanoOR3.5.37

RanoOR3.5.38

RanoOR3.5.39

RanoOR3.5.40

RanoOR3.5.41

RanoOR3.5.42

RanoOR3.5.43

RanoOR3.5.44

RanoOR3.5.45

RanoOR3.5.46

RanoOR3.5.47

RanoOR3.5.50

RanoOR3.5.51

RanoOR8.5.42

RanoOR10.9.1

RanoOR10.9.2

RanoOR10.9.3

RanoOR15.2.1

RanoOR15.2.2

RanoOR15.2.3

RanoOR15.2.5

RanoOR15.2.18

RanoOR15.2.19

RanoOR15.2.20

RanoOR15.2.21

RanoOR15.2.23

RanoOR15.2.24

RanoOR15.2.37

RanoOR15.2.38

RanoOR15.2.39

MamuOR7.1.1

MamuOR7.1.4

MamuOR7.1.5

MamuOR7.1.7

MamuOR7.1.8

MamuOR7.1.10

MamuOR7.1.12

MamuOR7.1.13

MamuOR7.1.16

MamuOR7.1.17

MamuOR7.1.19

MamuOR7.1.22

MamuOR7.1.23

MamuOR7.1.26

MamuOR7.1.28

MamuOR7.1.29

MamuOR7.1.31

MamuOR7.1.32

MamuOR7.1.33

MamuOR7.1.34

MamuOR7.1.35

MamuOR7.1.36

MamuOR7.1.37

MamuOR7.1.38

MamuOR7.2.5

MamuOR14.1.2

MamuOR14.1.3

MamuOR14.1.4

MamuOR14.1.5

MamuOR14.3.40

MamuOR14.3.41

MamuOR14.3.44

MamuOR14.3.46

MamuOR14.3.47

MamuOR14.3.49

MamuOR14.3.50

MamuOR14.3.52

MamuOR14.3.54

MamuOR14.5.2

MamuOR14.5.3

MamuOR14.5.6

MamuOR14.5.7

MamuOR14.13.4

MamuOR16.2.2

MamuORUr.1.4

HsOR1.1.3

HsOR1.1.4

HsOR1.1.5

HsOR5.4.5

HsOR8.1.1

HsOR11.8.1

HsOR11.8.3

HsOR11.8.4

HsOR11.8.5

HsOR11.8.6

HsOR11.8.9

HsOR11.8.13

HsOR11.9.4

HsOR11.9.5

HsOR11.9.7

HsOR11.10.2

HsOR11.10.6

HsOR11.10.8

HsOR11.11.3

HsOR11.11.4

HsOR11.11.15

HsOR11.11.16

HsOR11.11.17

HsOR11.11.18

HsOR11.11.19

HsOR11.11.20

HsOR11.13.7

HsOR11.13.8

HsOR11.13.10

HsOR11.13.11

HsOR11.18.6

HsOR14.1.3

HsOR14.1.5

HsOR14.1.7

HsOR14.1.10

HsOR14.1.12

HsOR14.1.13

HsOR14.1.15

HsOR14.1.17

HsOR14.1.18

HsOR14.1.20

HsOR14.1.22

HsOR14.1.23

HsOR14.2.5

HsOR15.1.8

HsOR15.1.9

HsOR15.2.1

HsOR15.2.2

HsOR15.2.3

HsOR15.2.6

HsOR17.2.1

HsOR17.2.2

HsOR19.1.3

CladeB:

OranORc2061.1

OranORc2061.6

OranORc2061.7

OranORc2061.8

OranORc2061.10

OranORc6611.1

OranORc11057.1

OranORc11857.1

OranORc28039.1

OranORc84976.1

ModoOR1.12.5

ModoOR1.12.6

ModoOR1.12.7

ModoOR2.3.1

ModoOR2.3.2

ModoOR2.4.15

ModoOR2.4.16

ModoOR2.4.17

ModoOR2.4.18

ModoOR2.5.10

ModoOR2.5.11

ModoOR2.5.12

ModoOR2.5.14

ModoOR2.5.15

ModoOR2.5.16

ModoOR2.5.18

ModoOR2.5.21

ModoOR4.7.2

ModoOR4.7.3

BotaOR6.1.1

BotaOR6.1.2

BotaOR6.1.3

BotaOR7.4.1

BotaOR7.5.1

BotaOR7.5.2

BotaOR7.5.3

BotaOR7.5.4

BotaOR7.5.5

BotaOR7.5.6

BotaOR7.5.7

BotaOR7.5.11

BotaOR7.5.12

BotaOR7.5.14

BotaOR7.5.15

BotaOR7.5.17

BotaOR7.5.18

BotaOR7.5.19

BotaOR7.5.20

BotaOR7.5.21

BotaOR7.5.22

BotaOR7.5.23

BotaOR7.6.1

BotaOR7.6.2

BotaORs345.1

BotaORs345.2

BotaORs345.4

BotaORs345.6

BotaORs345.8

BotaORs345.10

BotaORs345.11

BotaORs345.12

BotaORs345.13

BotaORs345.14

BotaORs345.16

BotaORs345.18

BotaORs865.1

BotaORs2480.1

BotaORs2480.2

BotaORs2480.3

BotaORs3499.1

BotaORs4387.1

BotaORs4706.1

BotaORs6406.1

BotaORs7647.2

BotaORs7727.1

BotaORs9604.1

BotaORs10505.1

BotaORs10771.2

BotaORs11070.1

BotaORs11747.1

BotaORs13538.1

BotaORs13538.2

BotaORs16334.1

BotaORs16868.1

BotaORs19868.1

CafaOR6.1.1

CafaOR11.1.3

CafaOR14.2.3

CafaOR14.2.4

CafaOR14.2.5

CafaOR14.2.6

CafaOR14.2.8

CafaOR14.2.9

CafaOR14.2.15

CafaOR14.2.16

CafaOR14.2.17

CafaOR14.2.18

CafaOR14.2.20

CafaOR14.2.21

CafaOR14.2.22

CafaOR14.2.24

CafaOR14.2.25

CafaOR14.2.26

CafaOR14.2.27

CafaOR14.2.28

CafaOR14.2.29

CafaOR14.2.30

CafaOR14.2.35

CafaOR14.2.36

CafaOR14.2.37

CafaOR14.2.38

CafaOR14.2.39

CafaOR14.2.40

CafaOR14.2.41

CafaOR14.2.42

CafaOR14.2.43

CafaOR14.2.44

CafaOR16.3.1

CafaOR20.3.1

CafaOR21.3.2

CafaOR21.3.3

CafaOR21.3.4

CafaOR21.3.5

CafaOR21.3.6

MmOR7.6.1

MmOR7.6.2

MmOR7.6.3

MmOR7.6.5

MmOR7.6.6

MmOR7.6.7

MmOR7.6.8

MmOR7.6.9

MmOR7.6.11

MmOR7.6.13

MmOR7.6.14

MmOR8.1.1

MmOR8.1.2

MmOR11.1.1

MmOR11.1.2

MmOR11.1.3

MmOR11.1.4

MmOR11.4.1

MmOR11.4.3

MmOR11.4.4

MmOR11.4.5

MmOR11.4.6

MmOR11.4.7

MmOR11.4.8

MmOR11.4.10

MmOR11.4.14

MmOR11.4.15

MmOR11.4.16

MmOR11.4.18

MmOR11.4.19

MmOR11.4.20

MmOR14.1.1

MmOR14.1.2

MmOR16.3.1

MmOR16.3.2

MmOR16.3.3

MmOR16.3.4

MmOR16.3.5

MmOR16.3.6

MmOR16.3.8

RanoOR1.8.1

RanoOR1.8.2

RanoOR1.8.3

RanoOR1.8.4

RanoOR1.8.6

RanoOR1.8.7

RanoOR1.8.8

RanoOR1.8.12

RanoOR1.8.13

RanoOR1.8.15

RanoOR1.8.16

RanoOR7.4.1

RanoOR10.3.1

RanoOR10.3.2

RanoOR10.3.3

RanoOR10.3.4

RanoOR10.5.1

RanoOR10.5.2

RanoOR10.5.3

RanoOR10.5.4

RanoOR10.5.5

RanoOR10.5.6

RanoOR10.5.9

RanoOR10.5.10

RanoOR10.5.11

RanoOR10.5.12

RanoOR10.5.13

RanoOR10.5.14

RanoOR10.5.20

RanoOR10.5.21

RanoOR10.5.22

RanoOR10.5.23

RanoOR10.5.25

RanoOR10.5.26

RanoOR10.5.28

RanoOR10.5.29

RanoOR10.5.30

RanoOR10.5.33

RanoOR10.5.34

RanoOR10.5.35

RanoOR10.5.36

RanoOR10.5.37

RanoOR10.5.38

RanoOR10.5.39

RanoOR10.5.40

RanoOR10.5.41

RanoOR11.4.1

RanoOR11.4.2

RanoOR11.4.3

RanoOR11.4.4

RanoOR11.4.5

RanoOR11.4.6

RanoOR15.1.1

RanoOR15.1.2

RanoOR15.1.3

MamuOR1.6.1

MamuOR1.6.13

MamuOR1.6.15

MamuOR1.6.16

MamuOR1.6.17

MamuOR1.6.19

MamuOR1.6.20

MamuOR1.6.21

MamuOR1.6.23

MamuOR1.6.26

MamuOR1.6.27

MamuOR1.6.28

MamuOR1.6.29

MamuOR1.6.30

MamuOR1.6.32

MamuOR3.2.2

MamuOR6.2.3

MamuOR14.8.10

MamuOR14.8.11

HsOR1.5.16

HsOR1.5.17

HsOR1.5.18

HsOR1.5.19

HsOR1.5.23

HsOR1.5.24

HsOR1.5.25

HsOR1.5.27

HsOR1.5.29

HsOR1.5.30

HsOR1.5.31

HsOR1.5.32

HsOR1.5.33

HsOR1.5.34

HsOR1.5.35

HsOR1.5.37

HsOR1.5.38

HsOR1.5.39

HsOR1.5.41

HsOR1.5.42

HsOR1.5.43

HsOR1.5.44

HsOR1.5.45

HsOR1.5.46

HsOR1.5.47

HsOR1.5.48

HsOR1.5.49

HsOR5.4.3

HsOR5.4.4

HsOR7.4.1

HsOR11.4.1

HsOR11.4.2

HsOR19.2.1

CladeC:

OranORc6280.1

OranORc12131.1

OranORc12714.1

OranORc16915.1

OranORc21616.1

OranORuc381.1

OranORuc381.2

OranORuc381.3

OranORuc381.4

OranORuc381.5

OranORuc381.7

ModoOR2.3.4

ModoOR2.3.5

ModoOR2.3.8

ModoOR2.3.9

ModoOR2.3.10

ModoOR2.3.11

ModoOR2.3.12

ModoOR2.3.14

ModoOR2.3.15

ModoOR2.3.16

ModoOR2.3.17

ModoOR2.3.18

ModoOR2.3.19

ModoOR2.3.20

ModoOR2.3.21

ModoOR2.4.4

ModoOR2.4.5

ModoOR2.4.6

ModoOR2.4.8

ModoOR2.4.11

ModoOR2.4.12

ModoOR2.4.13

ModoOR2.4.14

ModoOR2.5.1

ModoOR2.5.2

ModoOR2.5.5

ModoOR2.5.9

ModoOR4.11.1

ModoOR4.11.2

ModoOR4.11.3

ModoOR6.5.2

ModoOR6.5.3

ModoORUn.6.2

ModoORUn.22.1

BotaOR3.4.1

BotaOR3.4.2

BotaOR3.4.3

BotaOR3.4.4

BotaOR3.4.7

BotaOR3.4.8

BotaOR3.4.9

BotaOR3.4.11

BotaOR7.5.8

BotaOR7.5.9

BotaOR8.5.1

BotaOR8.5.2

BotaOR8.5.4

BotaOR8.5.5

BotaOR8.5.6

BotaOR23.2.2

BotaOR23.2.3

BotaOR23.3.9

BotaOR23.3.11

BotaOR23.3.13

BotaOR23.3.15

BotaOR23.3.16

BotaOR23.3.18

BotaOR23.3.19

BotaOR23.3.20

BotaOR23.3.21

BotaOR23.3.23

BotaOR25.1.1

BotaORs1453.1

BotaORs2471.4

BotaORs2848.1

BotaORs2848.2

BotaORs2848.4

BotaORs3950.1

BotaORs6007.3

BotaORs6079.1

BotaORs6079.2

BotaORs6079.3

BotaORs7982.1

BotaORs8138.1

BotaORs8209.1

BotaORs8398.1

BotaORs8543.1

BotaORs8543.2

BotaORs8745.4

BotaORs9333.1

BotaORs9333.2

BotaORs9604.2

BotaORs14741.1

BotaORs48742.1

CafaOR6.3.1

CafaOR8.1.2

CafaOR8.1.3

CafaOR8.1.5

CafaOR8.1.7

CafaOR11.2.1

CafaOR14.2.10

CafaOR14.2.46

CafaOR14.2.47

CafaOR14.2.48

CafaOR35.1.1

CafaOR35.1.2

CafaOR35.1.4

CafaOR35.1.6

CafaOR35.1.7

CafaOR35.1.10

CafaOR35.1.11

MmOR11.1.5

MmOR11.1.6

MmOR11.1.7

MmOR11.1.8

MmOR11.1.9

MmOR11.1.10

MmOR11.1.11

MmOR11.1.12

MmOR11.1.13

MmOR11.1.14

MmOR11.1.15

MmOR11.1.16

MmOR11.1.17

MmOR11.1.18

MmOR11.1.19

MmOR11.3.1

MmOR11.3.2

MmOR11.3.3

MmOR11.4.13

MmOR11.4.17

MmOR11.5.1

MmOR11.5.2

MmOR13.1.1

MmOR13.1.2

MmOR13.1.3

MmOR13.1.4

MmOR13.1.5

MmOR13.1.8

MmOR13.1.9

MmOR13.1.10

MmOR13.1.11

MmOR13.1.12

MmOR13.1.13

MmOR16.1.4

MmOR17.2.2

MmOR17.2.3

MmOR17.2.4

MmOR17.2.32

MmOR17.2.38

MmOR17.2.39

MmOR17.2.46

MmOR17.2.47

MmOR17.2.48

MmOR17.2.50

MmOR17.2.51

MmOR17.2.52

MmOR17.2.53

MmOR17.2.55

RanoOR10.2.1

RanoOR10.3.5

RanoOR10.3.6

RanoOR10.3.7

RanoOR10.3.9

RanoOR10.3.10

RanoOR10.3.11

RanoOR10.3.12

RanoOR10.3.13

RanoOR10.3.14

RanoOR10.3.15

RanoOR10.3.16

RanoOR10.3.17

RanoOR10.3.18

RanoOR10.3.19

RanoOR10.6.1

RanoOR10.6.2

RanoOR17.2.1

RanoOR17.2.5

RanoOR17.2.6

RanoOR17.3.1

RanoOR17.3.3

RanoOR17.3.5

RanoOR20.1.1

RanoOR20.1.3

RanoOR20.1.4

RanoOR20.1.6

RanoOR20.1.8

RanoOR20.1.11

RanoOR20.1.12

RanoOR20.1.13

RanoOR20.1.14

RanoOR20.1.15

RanoOR20.1.16

RanoOR20.1.17

RanoOR20.1.19

RanoOR20.1.27

RanoOR20.1.28

RanoOR20.1.35

RanoOR20.1.75

RanoOR20.1.77

RanoOR20.1.78

RanoOR20.1.79

MamuOR1.6.7

MamuOR1.6.34

MamuOR1.6.46

MamuOR1.6.48

MamuOR1.6.50

MamuOR4.1.1

MamuOR4.1.2

MamuOR4.2.1

MamuOR4.2.3

MamuOR4.2.4

MamuOR4.2.5

MamuOR4.2.7

MamuOR4.2.9

MamuOR4.2.10

MamuOR4.2.11

MamuOR4.2.12

MamuOR4.2.14

MamuOR4.2.24

MamuOR6.2.1

MamuOR6.2.2

HsOR1.5.1

HsOR1.5.3

HsOR1.5.4

HsOR1.5.5

HsOR1.5.15

HsOR5.4.2

HsOR6.2.1

HsOR6.2.3

HsOR6.3.2

HsOR6.3.4

HsOR6.3.6

HsOR6.3.8

HsOR6.3.23

HsOR6.3.26

HsOR16.1.3

CladeD:

OranORc5778.1

OranORc22999.2

OranORuc453.4

ModoOR2.1.1

ModoOR2.1.2

ModoOR2.1.3

ModoOR2.1.4

ModoOR2.1.5

ModoOR2.1.7

ModoOR2.1.8

ModoOR2.1.19

ModoOR2.1.20

ModoOR2.1.21

ModoOR2.1.24

ModoOR2.1.26

ModoOR2.1.27

ModoOR2.1.28

ModoOR2.1.30

ModoOR2.1.31

ModoOR2.1.33

ModoOR2.1.34

ModoOR2.1.35

ModoOR2.1.36

ModoOR2.1.37

ModoOR2.1.38

ModoOR3.1.16

ModoOR3.1.17

ModoOR3.2.1

ModoOR3.2.2

ModoOR3.2.5

ModoORUn.7.1

ModoORUn.7.2

ModoORUn.13.1

BotaOR1.2.1

BotaOR1.2.3

BotaOR3.1.2

BotaOR7.1.1

BotaORs246.1

BotaORs246.3

BotaORs467.1

BotaORs467.2

BotaORs478.4

BotaORs857.1

BotaORs857.2

BotaORs857.3

BotaORs1059.2

BotaORs1059.6

BotaORs2710.3

BotaORs3752.4

BotaORs3752.5

BotaORs5148.1

BotaORs5577.1

BotaORs5602.1

BotaORs5971.1

BotaORs9026.1

BotaORs9026.2

BotaORs11559.1

BotaORs13483.1

CafaOR20.1.5

CafaOR20.1.8

CafaOR20.1.9

CafaOR20.1.10

CafaOR20.1.12

CafaOR20.1.13

CafaOR20.1.14

CafaOR20.1.15

CafaOR21.4.3

CafaOR21.4.4

CafaOR38.1.1

CafaOR38.1.2

CafaOR38.1.4

CafaOR38.1.6

CafaOR38.1.7

CafaOR38.1.8

CafaOR38.1.9

CafaOR38.1.19

CafaOR38.1.20

CafaOR38.1.24

CafaOR38.1.26

CafaOR38.1.27

CafaOR38.1.28

CafaOR38.1.29

CafaOR38.1.30

CafaOR38.1.31

MmOR1.3.1

MmOR1.3.3

MmOR1.3.4

MmOR1.3.5

MmOR1.3.6

MmOR1.3.7

MmOR1.4.13

MmOR1.4.15

MmOR7.7.7

MmOR7.7.37

MmOR8.2.1

MmORUn.2.1

MmORUn.6.1

MmORUn.14.1

RanoOR1.9.9

RanoOR7.3.1

RanoOR7.3.2

RanoOR7.3.3

RanoOR7.3.4

RanoOR7.3.6

RanoOR13.1.1

RanoOR13.1.2

RanoOR13.1.4

RanoOR13.1.5

RanoOR13.1.6

RanoOR13.1.20

RanoOR13.1.21

RanoOR13.1.23

RanoOR19.2.1

MamuOR1.5.3

MamuOR1.5.5

MamuOR1.5.9

MamuOR1.5.10

MamuOR1.5.20

MamuOR19.3.1

MamuOR19.3.2

MamuOR19.3.3

MamuOR19.3.4

HsOR1.4.1

HsOR1.4.2

HsOR1.4.4

HsOR1.4.5

HsOR1.4.9

HsOR1.4.10

HsOR1.4.26

HsOR1.4.27

HsOR19.4.1

HsOR19.4.2

HsOR19.4.3

HsOR19.4.4

HsOR19.4.5

CladeE:

OranORc4971.2

OranORc4971.4

OranORc4971.5

OranORc16699.1

OranORc20012.1

OranORc23097.3

OranORc24401.1

OranORc27952.1

OranORc32114.1

OranORc34719.1

OranORc40365.1

OranORc55172.1

ModoOR2.4.2

ModoOR2.5.26

ModoOR6.3.15

ModoOR8.3.5

ModoOR8.3.9

ModoOR8.3.11

ModoOR8.3.12

ModoOR8.3.13

ModoOR8.3.14

ModoOR8.3.15

ModoOR8.3.16

ModoOR8.3.17

ModoOR8.3.18

ModoOR8.3.20

ModoOR8.3.21

ModoOR8.3.22

ModoOR8.3.24

ModoOR8.4.4

ModoOR8.4.5

ModoOR8.4.6

ModoOR8.4.7

ModoOR8.4.8

ModoORUn.4.1

ModoORUn.4.2

ModoORUn.4.3

ModoORUn.4.4

ModoORUn.4.5

ModoORUn.4.6

ModoORUn.4.7

ModoORUn.4.8

ModoORUn.4.9

ModoORUn.4.10

ModoORUn.4.12

ModoORUn.4.13

ModoORUn.4.14

ModoORUn.4.15

ModoORUn.4.17

ModoORUn.4.18

ModoORUn.4.19

ModoORUn.5.9

ModoORUn.16.1

BotaOR5.3.2

BotaORs1095.1

BotaORs1095.4

BotaORs1095.10

BotaORs1095.11

BotaORs1095.18

BotaORs1331.1

BotaORs1331.2

BotaORs1331.4

BotaORs2021.1

BotaORs2021.3

BotaORs2021.8

BotaORs2395.1

BotaORs2395.3

BotaORs2395.4

BotaORs2395.6

BotaORs2501.2

BotaORs2501.5

BotaORs2945.1

BotaORs2945.5

BotaORs2945.8

BotaORs3919.3

BotaORs3919.5

BotaORs6387.3

BotaORs6387.4

BotaORs7469.1

BotaORs7469.3

BotaORs8532.1

BotaORs9595.2

BotaORs9966.4

BotaORs10605.2

BotaORs10726.2

BotaORs11300.1

BotaORs11300.2

BotaORs12266.1

BotaORs13782.2

BotaORs14747.1

BotaORs17586.1

BotaORs18190.1

BotaORs40237.1

CafaOR2.1.3

CafaOR3.1.2

CafaOR3.1.3

CafaOR3.1.6

CafaOR3.1.7

CafaOR3.1.8

CafaOR3.1.9

CafaOR3.1.10

CafaOR3.1.11

CafaOR3.1.13

CafaOR8.1.11

CafaOR10.2.2

CafaOR10.2.7

CafaOR10.2.8

CafaOR10.2.13

CafaOR14.1.1

CafaOR27.1.1

CafaOR27.1.3

CafaOR27.1.4

CafaOR27.1.5

CafaOR27.1.6

CafaOR27.1.9

CafaOR27.1.10

CafaOR27.1.11

CafaOR27.1.16

CafaOR29.1.1

CafaOR29.1.4

CafaOR31.1.1

CafaORUn.2.4

CafaORUn.2.5

CafaORUn.2.7

CafaORUn.2.9

CafaORUn.2.10

CafaORUn.15.1

CafaORUn.16.1

CafaORUn.18.1

CafaORUn.24.2

MmOR10.4.5

MmOR10.4.7

MmOR10.4.8

MmOR10.4.9

MmOR10.4.10

MmOR10.4.11

MmOR10.4.12

MmOR10.4.13

MmOR10.4.14

MmOR10.4.15

MmOR10.4.18

MmOR10.4.19

MmOR10.4.20

MmOR10.4.23

MmOR10.4.25

MmOR10.4.26

MmOR10.4.28

MmOR10.4.29

MmOR10.4.32

MmOR10.4.34

MmOR10.4.35

MmOR10.4.36

MmOR10.4.37

MmOR10.4.38

MmOR10.4.39

MmOR10.4.41

MmOR10.4.44

MmOR10.4.45

MmOR10.4.46

MmOR10.4.47

MmOR10.4.48

MmOR10.4.49

MmOR10.4.50

MmOR10.4.51

MmOR10.4.53

MmOR10.4.54

MmOR10.4.55

MmOR10.4.56

MmOR10.4.57

MmOR10.4.58

MmOR10.4.59

MmOR10.4.60

MmOR10.4.61

MmOR10.4.62

MmOR10.4.64

MmOR10.4.67

MmOR10.4.68

MmOR10.4.69

MmORUn.21.1

RanoOR7.1.3

RanoOR7.1.4

RanoOR7.1.5

RanoOR7.1.6

RanoOR7.1.7

RanoOR7.1.9

RanoOR7.1.11

RanoOR7.1.15

RanoOR7.1.17

RanoOR7.1.19

RanoOR7.1.21

RanoOR7.1.23

RanoOR7.1.26

RanoOR7.1.28

RanoOR7.1.30

RanoOR7.1.31

RanoOR7.1.32

RanoOR7.1.33

RanoOR7.1.35

RanoOR7.1.41

RanoOR7.1.42

RanoOR7.1.45

RanoOR7.1.46

RanoOR7.1.47

RanoOR7.1.57

RanoOR7.1.58

RanoOR7.1.62

RanoOR7.1.63

RanoOR7.1.69

RanoOR7.1.73

RanoOR7.1.76

RanoOR7.1.77

RanoOR7.1.78

RanoOR7.1.80

RanoOR7.1.82

RanoOR7.1.85

RanoOR7.1.86

RanoOR7.1.88

RanoOR7.1.90

RanoOR7.1.106

RanoOR7.1.107

RanoOR7.1.108

RanoOR7.1.111

RanoOR7.1.118

RanoOR7.1.119

RanoOR7.1.120

RanoOR7.1.124

RanoOR7.1.126

RanoOR7.1.130

RanoOR7.1.131

RanoOR7.1.136

RanoOR7.1.138

RanoOR7.1.143

RanoOR7.1.145

RanoOR7.1.147

RanoOR7.1.152

RanoOR7.1.170

RanoOR7.1.172

RanoOR7.1.173

RanoOR7.1.176

RanoOR7.1.178

RanoOR7.1.179

RanoOR7.1.180

RanoOR7.1.181

RanoOR7.1.184

RanoOR7.1.185

RanoOR7.1.186

RanoOR7.1.187

RanoORUn.18.1

MamuOR11.2.7

MamuOR11.2.11

MamuOR11.2.14

MamuOR11.2.17

MamuOR11.2.18

MamuOR11.2.19

HsOR12.5.6

HsOR12.5.9

HsOR12.5.11

HsOR12.5.12

HsOR12.5.14

HsOR12.5.16

HsOR12.5.17

HsOR12.5.18

HsOR12.5.19

HsOR12.5.20

HsOR12.5.21

HsOR12.5.23

HsOR12.5.24

CladeF:

OranORuc253.1

ModoOR6.1.1

ModoOR6.3.1

ModoOR6.3.2

ModoOR6.3.3

ModoOR6.3.4

ModoOR6.3.5

ModoOR6.3.6

ModoOR6.3.7

ModoOR6.3.8

ModoOR6.3.9

ModoOR6.3.10

ModoOR6.3.12

ModoOR6.3.13

ModoOR6.3.14

ModoOR6.4.1

ModoOR6.4.2

ModoOR6.4.4

ModoOR6.4.5

ModoOR6.4.6

ModoOR6.4.7

ModoOR6.4.9

ModoOR6.4.10

ModoOR6.4.11

ModoOR6.4.12

ModoOR6.4.13

ModoOR6.4.14

ModoOR6.4.15

ModoOR6.4.16

ModoOR6.4.18

ModoOR6.4.19

ModoOR6.4.20

ModoOR6.4.21

ModoOR6.4.22

ModoOR6.4.24

ModoOR6.4.25

ModoOR6.4.27

ModoOR6.4.29

BotaOR8.3.1

BotaOR8.4.2

BotaORs545.1

BotaORs1561.2

BotaORs1561.6

BotaORs1561.7

BotaORs2045.2

BotaORs2045.3

BotaORs2045.4

BotaORs2377.1

BotaORs2377.2

BotaORs2377.3

BotaORs2841.4

BotaORs3088.1

BotaORs10790.1

BotaORs14643.1

BotaORs14750.1

BotaORs14793.1

BotaORs16424.1

BotaORs26398.1

CafaOR11.4.1

CafaOR11.4.2

CafaOR11.4.3

CafaOR11.4.4

CafaOR11.4.5

CafaOR11.4.6

CafaOR11.4.7

CafaOR11.4.8

CafaOR11.5.1

CafaOR11.5.2

CafaOR11.5.3

CafaOR11.5.4

CafaOR11.5.5

CafaOR11.5.6

CafaOR11.5.7

CafaOR11.5.8

CafaOR11.5.9

CafaOR11.5.10

CafaOR11.5.11

CafaOR11.5.12

CafaOR11.6.1

CafaORUn.7.1

CafaORUn.7.2

MmOR4.1.1

MmOR4.1.2

MmOR4.1.4

MmOR4.1.6

MmOR4.1.7

MmOR4.1.8

MmOR4.2.1

MmOR4.2.2

MmOR4.2.3

MmOR4.2.5

MmOR4.3.1

RanoOR5.1.1

RanoOR5.1.2

RanoOR5.1.4

RanoOR5.1.5

RanoOR5.1.6

RanoOR5.1.7

RanoOR5.1.8

RanoOR5.2.1

RanoOR5.2.4

RanoOR5.2.5

RanoOR5.2.7

RanoOR5.2.8

RanoOR5.2.9

RanoOR5.2.10

RanoOR5.2.11

RanoOR5.2.12

RanoOR5.3.1

MamuOR15.2.1

MamuOR15.3.6

MamuOR15.3.8

MamuOR15.3.9

MamuOR15.3.10

MamuOR15.3.11

MamuOR15.4.2

MamuOR15.4.4

MamuOR15.4.5

MamuOR15.4.6

MamuOR15.4.8

MamuOR15.4.9

HsOR9.1.2

HsOR9.1.3

HsOR9.4.1

HsOR9.4.2

HsOR9.4.3

HsOR9.4.4

HsOR9.4.6

HsOR9.4.7

HsOR9.4.8

HsOR9.4.11

HsOR9.5.1

CladeG:

OranORc3905.1

ModoOR4.2.1

ModoOR4.2.2

ModoOR4.2.4

ModoOR4.2.8

ModoOR4.2.10

ModoOR4.2.11

ModoOR4.2.38

ModoOR4.3.1

ModoOR4.3.2

ModoOR4.3.4

ModoOR4.3.5

ModoOR4.3.6

ModoOR4.3.7

ModoOR4.3.8

ModoOR4.3.9

ModoOR4.3.10

ModoOR4.3.11

ModoOR4.3.12

ModoOR4.3.14

ModoOR4.3.17

ModoOR4.3.19

ModoOR4.3.20

ModoOR4.3.21

ModoOR4.3.25

ModoOR4.3.26

ModoOR4.3.27

ModoOR4.3.30

ModoOR4.3.31

ModoOR4.3.32

ModoOR4.3.34

ModoOR4.3.39

ModoOR4.3.40

ModoOR4.3.41

ModoOR4.3.42

ModoOR4.3.43

ModoOR4.3.44

ModoOR4.3.46

ModoOR4.3.47

ModoOR4.3.48

ModoOR4.3.49

ModoORUn.23.1

BotaOR29.1.5

BotaOR29.1.8

BotaOR29.1.9

BotaOR29.2.1

BotaOR29.2.2

BotaOR29.2.11

BotaOR29.2.12

BotaOR29.2.15

BotaOR29.3.1

BotaOR29.3.2

BotaOR29.3.3

BotaOR29.3.4

BotaOR29.3.5

BotaOR29.3.6

BotaOR29.3.7

BotaOR29.3.8

BotaOR29.3.9

BotaOR29.3.10

BotaOR29.3.11

BotaOR29.3.12

BotaOR29.3.13

BotaOR29.3.14

BotaORs828.3

BotaORs828.5

BotaORs828.8

BotaORs828.9

BotaORs828.11

BotaORs828.12

BotaORs828.13

BotaORs828.14

BotaORs828.17

BotaORs828.19

BotaORs3723.3

BotaORs3723.4

BotaORs6990.3

BotaORs6990.5

BotaORs7907.1

BotaORs7907.5

BotaORs8364.1

BotaORs8424.1

BotaORs10723.2

BotaORs12177.1

BotaORs12206.1

BotaORs13175.2

BotaORs13951.1

BotaORs24159.1

CafaOR5.2.1

CafaOR5.2.2

CafaOR5.2.3

CafaOR5.2.4

CafaOR5.2.5

CafaOR5.2.6

CafaOR5.2.7

CafaOR5.2.8

CafaOR5.2.9

CafaOR5.2.11

CafaOR5.2.12

CafaOR5.2.13

CafaOR5.2.14

CafaOR5.2.15

CafaOR5.2.16

CafaOR5.2.17

CafaOR5.2.18

CafaOR5.2.20

CafaOR5.2.22

CafaOR5.2.24

CafaOR5.2.25

CafaOR5.2.26

CafaOR5.2.40

CafaOR5.2.43

MmOR9.3.2

MmOR9.3.3

MmOR9.3.4

MmOR9.3.5

MmOR9.3.6

MmOR9.3.7

MmOR9.3.8

MmOR9.3.10

MmOR9.3.12

MmOR9.3.13

MmOR9.3.14

MmOR9.3.15

MmOR9.3.16

MmOR9.3.17

MmOR9.3.18

MmOR9.3.19

MmOR9.3.21

MmOR9.3.22

MmOR9.3.23

MmOR9.3.24

MmOR9.3.25

MmOR9.3.27

MmOR9.3.28

MmOR9.3.29

MmOR9.3.30

MmOR9.3.31

MmOR9.3.32

MmOR9.3.33

MmOR9.3.35

MmOR9.3.36

MmOR9.3.37

MmOR9.3.38

MmOR9.3.40

MmOR9.3.42

MmOR9.3.43

MmOR9.3.44

MmOR9.3.46

MmOR9.3.47

MmOR9.3.48

MmOR9.3.49

MmOR9.3.50

MmOR9.3.51

MmOR9.3.52

MmOR9.3.53

MmOR9.3.54

MmOR9.3.55

MmOR9.3.57

MmOR9.3.60

MmOR9.3.61

MmOR9.3.63

MmOR9.3.64

MmOR9.3.66

MmOR9.3.67

MmOR9.3.71

MmOR9.3.72

MmOR9.3.73

MmOR9.3.74

MmOR9.3.77

MmOR9.3.80

MmOR9.3.81

MmOR9.3.83

MmOR9.3.84

MmOR9.3.86

MmOR9.3.87

MmOR9.3.97

MmOR9.3.98

MmOR9.3.99

MmOR9.3.100

MmOR9.3.101

MmOR9.3.102

MmOR9.3.103

MmOR9.3.104

MmOR9.3.105

MmOR9.3.106

MmOR9.3.117

MmORUn.11.1

RanoOR8.3.1

RanoOR8.3.2

RanoOR8.3.3

RanoOR8.3.4

RanoOR8.3.5

RanoOR8.3.7

RanoOR8.3.8

RanoOR8.3.9

RanoOR8.3.10

RanoOR8.3.11

RanoOR8.3.13

RanoOR8.3.20

RanoOR8.3.21

RanoOR8.3.23

RanoOR8.4.1

RanoOR8.4.2

RanoOR8.4.3

RanoOR8.4.5

RanoOR8.4.6

RanoOR8.4.8

RanoOR8.4.9

RanoOR8.4.10

RanoOR8.4.11

RanoOR8.4.12

RanoOR8.4.13

RanoOR8.4.14

RanoOR8.4.15

RanoOR8.4.16

RanoOR8.4.17

RanoOR8.4.18

RanoOR8.4.19

RanoOR8.4.20

RanoOR8.4.21

RanoOR8.4.22

RanoOR8.4.23

RanoOR8.4.24

RanoOR8.4.25

RanoOR8.4.26

RanoOR8.4.27

RanoOR8.4.28

RanoOR8.4.29

RanoOR8.4.31

RanoOR8.4.32

RanoOR8.4.33

RanoOR8.4.34

RanoOR8.4.35

RanoOR8.4.36

RanoOR8.4.37

RanoOR8.4.39

RanoOR8.4.40

RanoOR8.4.42

RanoOR8.4.43

RanoOR8.4.44

RanoOR8.4.45

RanoOR8.4.47

RanoOR8.4.48

RanoOR8.4.49

RanoOR8.4.54

RanoOR8.4.57

RanoOR8.4.58

RanoOR8.4.61

RanoOR8.4.62

RanoOR8.4.63

RanoOR8.4.66

RanoOR8.4.68

RanoOR8.4.69

RanoOR8.4.71

RanoOR8.4.74

RanoOR8.4.75

RanoOR8.4.76

RanoOR8.4.77

RanoOR8.4.78

RanoOR8.4.80

RanoOR8.5.4

RanoOR8.5.5

RanoOR8.5.6

RanoOR8.5.7

RanoOR8.5.8

RanoOR8.5.18

RanoOR8.5.19

RanoOR8.5.20

RanoOR8.5.21

RanoOR8.5.22

RanoOR8.5.25

RanoOR8.5.28

RanoOR8.5.30

RanoOR8.5.31

RanoOR8.5.32

RanoOR8.5.41

MamuOR14.13.15

MamuOR14.13.17

MamuOR14.13.21

MamuOR14.13.23

MamuOR14.13.24

MamuOR14.13.25

MamuOR14.13.26

MamuOR14.13.29

MamuOR14.13.30

MamuOR14.13.31

HsOR11.18.5

HsOR11.18.25

HsOR11.18.26

HsOR11.18.27

HsOR11.18.33

HsOR11.18.34

HsOR11.18.35

HsOR11.18.36

HsOR11.18.41

HsOR11.18.42

CladeH:

ModoOR3.1.2

ModoOR3.1.3

ModoOR3.1.4

ModoOR3.1.7

ModoOR3.1.8

ModoOR3.1.9

ModoOR3.1.11

ModoOR3.1.12

ModoOR3.1.13

ModoOR3.1.14

ModoOR3.3.1

ModoOR3.3.2

ModoOR3.3.3

ModoOR3.3.4

ModoOR3.3.5

ModoOR3.3.6

ModoOR3.3.7

ModoOR3.3.8

ModoOR3.3.9

ModoOR3.3.10

ModoOR3.3.11

ModoOR3.3.12

ModoOR3.3.15

ModoOR3.3.17

BotaOR2.2.2

BotaOR2.2.3

BotaOR7.2.1

BotaOR7.2.2

BotaOR7.2.3

BotaOR7.2.4

BotaOR7.7.1

BotaOR7.7.4

BotaOR7.7.5

BotaOR7.7.6

BotaOR7.7.7

BotaOR7.7.8

BotaOR7.7.9

BotaOR18.1.1

BotaOR19.3.1

BotaOR19.3.3

BotaOR25.2.1

BotaOR25.2.3

BotaORX.1.2

BotaORX.1.6

BotaORs1278.1

BotaORs2470.3

BotaORs2471.3

BotaORs2547.3

BotaORs2547.5

BotaORs2547.10

BotaORs3803.5

BotaORs4029.1

BotaORs4029.4

BotaORs4029.6

BotaORs4827.2

BotaORs4827.4

BotaORs4827.5

BotaORs5171.2

BotaORs5171.5

BotaORs5551.3

BotaORs5619.5

BotaORs5784.1

BotaORs5784.3

BotaORs5784.4

BotaORs6372.1

BotaORs6372.2

BotaORs6597.2

BotaORs6743.1

BotaORs6743.2

BotaORs7120.1

BotaORs7120.2

BotaORs7120.5

BotaORs7204.3

BotaORs7231.1

BotaORs7231.3

BotaORs7559.2

BotaORs7565.1

BotaORs7565.2

BotaORs7700.1

BotaORs8349.2

BotaORs9494.1

BotaORs9990.1

BotaORs10459.1

BotaORs10620.2

BotaORs10993.1

BotaORs10993.2

BotaORs11458.1

BotaORs11598.1

BotaORs12604.1

BotaORs12960.1

BotaORs14383.1

BotaORs21090.1

BotaORs31287.1

BotaORs34589.1

BotaORs37865.1

CafaOR20.1.18

CafaOR20.1.19

CafaOR20.1.20

CafaOR20.1.21

CafaOR20.1.22

CafaOR20.1.25

CafaOR20.1.27

CafaOR20.1.28

CafaOR20.1.29

CafaOR20.1.30

CafaOR20.1.31

CafaOR20.1.33

CafaOR20.1.35

CafaOR20.1.37

CafaOR20.1.38

CafaOR20.1.40

CafaOR20.1.41

CafaOR20.1.43

CafaOR20.1.44

CafaOR20.1.45

CafaOR20.1.46

CafaOR20.1.47

CafaOR20.1.50

CafaOR20.1.51

CafaOR20.1.52

CafaOR20.1.53

CafaOR20.1.54

CafaOR20.1.55

CafaOR20.1.57

CafaOR20.1.59

CafaOR20.2.1

CafaOR20.2.3

CafaOR20.2.4

CafaOR20.2.5

CafaOR20.2.6

CafaOR20.2.8

CafaOR20.2.9

CafaOR20.2.10

CafaOR20.2.11

CafaOR20.2.12

CafaOR20.2.13

CafaOR20.2.14

CafaOR20.2.15

CafaOR20.2.16

CafaOR20.2.17

CafaOR20.2.18

CafaOR20.2.19

MmOR8.3.1

MmOR9.2.2

MmOR9.2.3

MmOR9.2.4

MmOR9.2.5

MmOR9.2.8

MmOR9.2.9

MmOR9.2.10

MmOR9.2.11

MmOR9.2.15

MmOR9.2.17

MmOR9.2.18

MmOR9.2.19

MmOR9.2.21

MmOR9.2.22

MmOR9.2.23

MmOR9.2.24

MmOR9.2.25

MmOR9.2.26

MmOR9.2.29

MmOR9.2.31

MmOR9.2.34

MmOR9.2.35

MmOR9.2.36

MmOR9.2.39

MmOR9.2.40

MmOR9.2.41

MmOR9.2.42

MmOR9.2.43

MmOR9.2.44

MmOR9.2.45

MmOR9.2.46

MmOR9.2.47

MmOR9.2.48

MmOR10.2.2

MmOR10.2.3

MmOR10.2.5

MmOR10.2.6

MmOR10.2.7

MmOR10.2.8

MmOR10.2.9

MmOR16.2.1

RanoOR7.2.1

RanoOR7.2.4

RanoOR7.2.5

RanoOR7.2.6

RanoOR7.2.7

RanoOR7.2.8

RanoOR7.2.10

RanoOR7.2.11

RanoOR7.2.12

RanoOR7.2.13

RanoOR7.2.14

RanoOR7.2.15

RanoOR8.1.3

RanoOR8.1.5

RanoOR8.1.6

RanoOR8.1.7

RanoOR8.2.2

RanoOR8.2.4

RanoOR8.2.5

RanoOR8.2.6

RanoOR8.2.7

RanoOR8.2.8

RanoOR8.2.9

RanoOR8.2.10

RanoOR8.2.11

RanoOR8.2.13

RanoOR8.2.14

RanoOR8.2.15

RanoOR8.2.18

RanoOR8.2.19

RanoOR8.2.20

RanoOR8.2.21

RanoOR8.2.23

RanoOR8.2.24

RanoOR8.2.25

RanoOR8.2.27

RanoOR8.2.32

RanoOR8.2.33

RanoOR8.2.35

RanoOR8.2.36

RanoOR8.2.37

RanoOR8.2.38

RanoOR8.2.39

RanoOR8.2.40

RanoOR8.2.41

RanoOR8.2.42

RanoOR8.2.45

RanoOR8.2.46

RanoOR8.2.47

RanoOR8.2.48

RanoOR8.2.49

RanoOR8.2.52

RanoOR8.2.54

RanoOR8.2.55

RanoOR8.2.58

RanoOR8.2.59

RanoOR8.2.60

RanoOR8.2.61

RanoOR8.2.62

RanoOR8.2.63

RanoOR8.2.64

RanoOR8.2.65

RanoOR19.1.1

MamuOR19.1.3

MamuOR19.1.6

MamuOR19.1.10

MamuOR19.2.1

HsOR19.2.4

HsOR19.2.5

HsOR19.2.7

HsOR19.2.8

HsOR19.2.11

HsOR19.2.14

HsOR19.3.1

HsOR19.3.2

HsOR19.3.3

HsOR19.3.6

HsOR19.3.11

CladeI:

ModoOR7.1.1

ModoOR7.1.2

ModoOR7.1.3

ModoOR7.1.5

ModoOR7.1.6

ModoOR7.1.7

ModoOR7.1.9

ModoOR7.1.10

ModoOR7.1.11

ModoOR7.1.12

ModoOR7.1.13

BotaOR1.1.2

BotaOR1.1.7

BotaOR1.1.8

BotaOR1.1.10

BotaORs2540.3

BotaORs2540.6

BotaORs4311.1

BotaORs4311.2

BotaORs4311.5

BotaORs4311.7

BotaORs9362.1

BotaORs10518.1

BotaORs10518.2

BotaORs11426.1

CafaOR33.1.2

CafaOR33.1.3

CafaOR33.1.4

CafaOR33.1.5

CafaOR33.1.6

CafaOR33.1.7

CafaOR33.1.8

CafaOR33.1.9

CafaOR33.1.10

CafaOR33.1.11

CafaOR33.1.12

CafaOR33.1.13

CafaOR33.1.14

CafaORUn.12.1

CafaORUn.17.3

MmOR16.4.2

MmOR16.4.3

MmOR16.4.4

MmOR16.4.5

MmOR16.4.7

MmOR16.4.8

MmOR16.4.10

MmOR16.4.11

MmOR16.4.14

MmOR16.4.15

MmOR16.4.18

MmOR16.4.19

MmOR16.4.20

MmOR16.4.22

MmOR16.4.23

MmOR16.4.25

MmOR16.4.26

MmOR16.4.27

MmOR16.4.31

RanoOR11.3.1

RanoOR11.3.2

RanoOR11.3.3

RanoOR11.3.4

RanoOR11.3.5

RanoOR11.3.6

RanoOR11.3.8

RanoOR11.3.9

RanoOR11.3.10

RanoOR11.3.11

RanoOR11.3.12

RanoOR11.3.13

RanoOR11.3.14

RanoOR11.3.15

RanoOR11.3.16

RanoOR11.3.18

RanoOR11.3.19

RanoOR11.3.20

RanoOR11.3.21

RanoOR11.3.22

RanoOR11.3.24

RanoOR11.3.26

RanoOR11.3.28

RanoOR11.3.30

RanoOR11.3.31

RanoOR11.3.32

RanoOR11.3.33

RanoOR11.3.34

RanoOR11.3.35

RanoOR11.3.36

RanoOR11.3.37

MamuOR2.1.2

MamuOR2.1.3

MamuOR2.1.4

MamuOR2.1.5

MamuOR2.1.6

MamuOR2.1.7

MamuOR2.1.8

MamuOR2.1.9

HsOR3.3.2

HsOR3.3.4

HsOR3.3.5

HsOR3.3.6

HsOR3.3.11

HsOR3.3.12

HsOR3.3.14

HsOR3.3.15

HsOR3.3.16

HsOR3.3.17

CladeJ:

ModoOR1.4.3

ModoOR1.4.5

ModoOR1.4.6

ModoOR1.4.7

ModoOR4.2.12

ModoOR4.2.13

ModoOR4.2.14

ModoOR4.2.15

ModoOR4.2.16

ModoOR4.2.18

ModoOR4.2.19

ModoOR4.2.20

ModoOR4.2.21

ModoOR4.2.22

ModoOR4.2.23

ModoOR4.2.24

ModoOR4.2.25

ModoOR4.2.26

ModoOR4.2.27

ModoOR4.2.29

ModoOR4.2.30

ModoOR4.2.31

ModoORUn.13.2

BotaOR29.1.11

BotaOR29.2.16

BotaOR29.2.17

BotaOR29.2.18

BotaOR29.2.21

BotaOR29.2.22

BotaOR29.2.23

BotaORs3789.2

BotaORs3789.3

BotaORs3789.4

BotaORs3789.5

BotaORs5122.1

BotaORs5122.2

BotaORs5122.3

BotaORs7130.1

BotaORs7130.2

BotaORs7130.3

BotaORs9876.2

BotaORs11455.2

BotaORs20883.1

BotaORs39537.1

CafaOR5.2.23

CafaOR5.2.27

CafaOR5.2.28

CafaOR5.2.29

CafaOR5.2.30

CafaOR5.2.31

CafaOR5.2.32

CafaOR5.2.33

CafaOR5.2.35

CafaOR5.2.36

CafaOR5.2.37

CafaOR5.2.38

CafaOR5.2.39

MmOR9.3.62

MmOR9.3.88

MmOR9.3.89

MmOR9.3.90

MmOR9.3.91

MmOR9.3.92

MmOR9.3.94

MmOR9.3.96

MmOR9.3.107

MmOR9.3.108

MmOR9.3.110

MmOR9.3.111

MmOR9.3.112

MmOR9.3.113

MmOR9.3.114

MmOR14.3.3

MmOR14.3.4

MmOR14.3.5

RanoOR8.4.56

RanoOR8.5.9

RanoOR8.5.10

RanoOR8.5.11

RanoOR8.5.12

RanoOR8.5.13

RanoOR8.5.15

RanoOR8.5.16

RanoOR8.5.17

RanoOR8.5.29

RanoOR8.5.33

RanoOR8.5.34

RanoOR8.5.35

RanoOR8.5.37

RanoOR8.5.38

RanoOR8.5.40

RanoOR15.2.33

RanoOR15.2.34

RanoOR15.2.35

RanoOR15.2.36

MamuOR7.2.2

MamuOR7.2.4

MamuOR14.13.7

MamuOR14.13.8

MamuOR14.13.9

MamuOR14.13.10

MamuOR14.13.11

MamuOR14.13.12

MamuOR14.13.14

HsOR11.18.8

HsOR11.18.9

HsOR11.18.11

HsOR11.18.12

HsOR11.18.13

HsOR11.18.14

HsOR11.18.16

HsOR11.18.19

HsOR14.2.2

HsOR14.2.4

CladeK:

ModoOR8.7.9

ModoOR8.7.11

ModoOR8.7.12

ModoOR8.7.14

ModoOR8.7.15

ModoOR8.7.17

ModoOR8.7.19

ModoOR8.7.20

BotaOR4.1.8

BotaOR4.1.10

BotaOR4.1.11

BotaOR4.1.12

BotaOR4.1.13

BotaOR4.1.15

BotaOR4.1.16

BotaOR4.3.1

BotaOR4.3.2

BotaOR4.3.3

BotaOR4.3.4

BotaOR4.4.1

CafaOR16.1.1

CafaOR16.1.2

CafaOR16.1.3

CafaOR16.1.5

CafaOR16.1.6

CafaOR16.1.7

CafaOR16.1.8

CafaOR16.1.9

CafaOR16.1.10

CafaOR16.1.11

MmOR6.3.12

MmOR6.3.13

MmOR6.3.14

MmOR6.3.15

MmOR6.3.21

MmOR6.3.22

MmOR6.3.23

MmOR6.3.24

MmOR6.3.25

RanoOR4.4.12

RanoOR4.4.13

RanoOR4.4.15

RanoOR4.4.17

RanoOR4.4.18

RanoOR4.4.19

RanoOR4.4.20

MamuOR3.5.14

MamuOR3.5.17

MamuOR3.5.20

MamuOR3.5.22

MamuOR3.5.23

HsOR6.4.1

HsOR7.6.10

HsOR7.6.11

HsOR7.6.12

HsOR7.6.13

HsOR7.6.15

HsOR7.6.19

HsOR7.6.21

HsOR7.6.23

CladeL:

OranORc1777.2

OranORc1777.4

OranORc1777.5

OranORc1777.6

OranORc1777.9

OranORc1777.11

OranORc7663.1

OranORc7663.2

OranORc8398.2

OranORc8764.1

OranORc8830.1

OranORc9697.1

OranORc9697.2

OranORc9697.3

OranORc9748.1

OranORc11514.1

OranORc12196.1

OranORc12196.2

OranORc12832.1

OranORc13465.1

OranORc13860.1

OranORc13925.1

OranORc15426.2

OranORc15646.1

OranORc15656.2

OranORc15795.2

OranORc15900.2

OranORc16008.1

OranORc16179.2

OranORc16409.1

OranORc16735.1

OranORc19003.2

OranORc19728.1

OranORc20679.1

OranORc20808.1

OranORc21446.1

OranORc21476.1

OranORc22953.2

OranORc28452.1

OranORc29349.1

OranORc32651.1

OranORc32889.1

OranORc35757.1

OranORc36098.1

OranORuc381.8

OranORuc659.1

OranORuc659.3

OranORuc659.4

OranORuc659.5

OranORuc659.6

OranORuc659.7

OranORuc659.8

ModoOR2.6.1

ModoOR2.6.2

ModoOR2.6.3

ModoOR2.6.4

ModoOR2.6.5

ModoOR2.6.7

ModoOR2.6.8

ModoOR2.6.9

ModoOR2.6.10

ModoOR2.6.11

ModoOR2.6.12

ModoOR2.6.16

ModoOR4.11.4

ModoOR4.11.6

ModoOR4.11.7

ModoOR4.11.9

ModoOR4.11.10

ModoOR4.11.11

ModoOR4.11.12

ModoOR4.11.13

ModoOR4.11.14

ModoOR4.11.15

ModoOR4.11.17

ModoOR4.11.18

ModoOR4.11.19

ModoOR4.11.20

ModoOR4.11.22

ModoOR4.11.24

ModoOR4.11.25

ModoOR4.11.26

ModoOR4.11.27

ModoOR4.11.28

ModoOR4.11.29

ModoOR4.11.31

ModoOR4.11.33

ModoOR5.8.1

ModoOR5.8.2

ModoOR5.8.3

ModoOR5.8.4

ModoOR5.8.5

ModoOR5.8.6

ModoOR5.8.7

ModoOR5.8.8

ModoOR5.8.9

ModoOR5.8.10

ModoOR5.8.13

ModoOR5.8.14

ModoOR5.8.15

ModoOR5.8.16

ModoOR5.8.17

ModoOR5.8.18

ModoOR5.8.19

ModoOR5.8.21

ModoOR5.8.22

ModoOR5.8.23

ModoOR5.8.24

ModoOR8.5.3

ModoORUn.12.1

ModoORUn.12.2

ModoORUn.12.3

ModoORUn.18.1

ModoORUn.24.1

ModoORUn.25.1

BotaOR23.3.7

BotaOR23.3.8

CafaOR14.2.45

CafaORUn.15.2

MmOR7.3.1

MmOR7.3.4

MmOR7.3.6

MmOR7.3.7

MmOR7.3.9

MmOR7.3.11

MmOR7.3.12

MmOR7.3.13

MmOR7.3.15

MmOR7.3.16

MmOR7.3.18

MmOR13.2.1

MmOR17.2.28

MmOR17.2.29

MmOR17.2.30

MmOR17.2.31

MmOR17.2.40

MmOR17.2.41

MmOR17.2.43

MmOR17.2.44

RanoOR1.5.3

RanoOR1.5.5

RanoOR1.5.9

RanoOR1.5.10

RanoOR1.5.12

RanoOR1.5.13

RanoOR1.5.14

RanoOR1.5.15

RanoOR1.5.16

RanoOR1.5.17

RanoOR1.5.18

RanoOR1.5.21

RanoOR17.1.2

RanoOR20.1.21

RanoOR20.1.22

RanoOR20.1.23

RanoOR20.1.24

RanoOR20.1.25

RanoOR20.1.26

RanoOR20.1.36

RanoOR20.1.37

RanoOR20.1.39

RanoOR20.1.40

RanoOR20.1.41

RanoOR20.1.42

MamuOR1.6.6

MamuOR1.6.39

MamuOR1.6.40

MamuOR1.6.41

MamuOR1.6.44

MamuOR4.2.16

HsOR1.5.6

HsOR1.5.8

HsOR1.5.9

HsOR1.5.12

HsOR1.5.36

HsOR1.5.50

HsOR6.3.16

CladeM:

ModoOR5.5.166

ModoOR5.5.167

ModoOR5.5.168

ModoOR5.5.170

ModoOR5.5.171

ModoOR5.5.173

ModoOR5.5.174

ModoOR5.5.175

ModoOR5.5.176

ModoOR5.5.177

ModoOR5.5.179

ModoOR5.5.182

ModoOR5.5.184

ModoOR5.5.186

ModoOR5.5.187

ModoOR5.5.188

ModoOR5.5.189

BotaORs2748.3

BotaORs3586.2

BotaORs3586.3

BotaORs4048.1

BotaORs4048.2

BotaORs4048.3

BotaORs4048.4

BotaORs4048.5

BotaORs4048.6

BotaORs12843.2

BotaORs17380.1

BotaORs23494.1

CafaOR18.4.38

CafaOR18.4.39

CafaOR18.4.42

CafaOR18.4.43

CafaOR18.4.44

CafaOR18.4.49

CafaOR18.4.51

CafaORUn.10.1

MmOR2.2.20

MmOR2.2.56

MmOR2.2.57

MmOR2.2.58

MmOR2.2.59

MmOR2.2.61

MmOR2.2.63

MmOR2.2.64

MmOR2.2.65

MmOR2.2.66

MmOR2.2.68

MmOR2.2.69

MmOR2.2.70

MmOR2.2.74

MmOR2.2.75

MmOR2.2.77

MmOR2.2.79

MmOR2.2.81

MmOR2.2.82

MmOR2.2.84

MmOR2.2.86

MmOR2.2.87

MmOR2.2.90

MmOR2.2.91

RanoOR3.3.46

RanoOR3.3.47

RanoOR3.3.49

RanoOR3.3.51

RanoOR3.3.52

RanoOR3.3.53

RanoOR3.3.54

RanoOR3.3.57

RanoOR3.3.60

RanoOR3.3.61

RanoOR3.3.63

RanoOR3.3.64

RanoOR3.3.66

RanoOR3.3.67

RanoOR3.3.72

RanoOR3.3.73

MamuOR14.3.15

MamuOR14.3.16

MamuOR14.3.21

HsOR11.11.49

HsOR11.11.59

HsOR11.11.61

CladeN:

OranORuc285.51

OranORuc285.54

ModoOR5.5.60

ModoOR5.5.61

ModoOR5.5.62

ModoOR5.5.63

ModoOR5.5.64

ModoOR5.5.65

ModoOR5.5.66

ModoOR5.5.67

ModoOR5.5.68

ModoOR5.5.69

ModoOR5.5.70

ModoOR5.5.71

ModoOR5.5.72

ModoOR5.5.73

ModoOR5.5.74

BotaOR24.1.3

BotaORs188.1

BotaORs4459.5

BotaORs4459.6

BotaORs4555.1

BotaORs6074.4

BotaORs10023.1

BotaORs11243.1

CafaOR18.4.83

CafaOR18.4.84

CafaOR18.4.85

CafaOR18.4.86

CafaOR18.4.87

CafaOR18.4.88

CafaOR18.4.90

CafaOR18.4.91

MmOR2.2.151

MmOR2.2.152

MmOR2.2.153

MmOR2.2.154

MmOR2.2.158

MmOR2.2.159

MmOR2.2.160

MmOR2.2.161

MmOR2.2.162

MmOR2.2.164

MmOR2.2.165

MmOR2.2.166

MmOR2.2.168

MmOR2.2.171

MmOR2.2.174

RanoOR3.3.154

RanoOR3.3.155

RanoOR3.3.157

RanoOR3.3.160

RanoOR3.3.161

RanoOR3.3.162

RanoOR3.3.163

RanoOR3.3.164

RanoOR3.3.167

MamuOR14.3.36

MamuOR14.3.37

MamuOR14.3.38

HsOR11.11.25

HsOR11.11.27

HsOR11.11.28

HsOR11.11.29

HsOR11.11.30

HsOR11.11.31

CladeO:

OranORuc285.4

OranORuc285.5

OranORuc285.6

OranORuc285.7

ModoOR5.5.191

ModoOR5.5.192

ModoOR5.5.193

ModoOR5.5.194

ModoOR5.5.214

ModoOR5.5.215

ModoOR5.5.216

ModoOR5.5.217

ModoOR5.5.218

ModoOR5.5.219

ModoOR5.5.220

ModoOR5.5.221

ModoOR5.5.222

ModoOR5.5.223

ModoOR5.5.224

ModoOR5.5.225

ModoOR5.5.226

ModoOR5.5.228

ModoOR5.5.229

ModoOR5.5.230

BotaORs1150.2

BotaORs3995.2

BotaORs3995.4

BotaORs4579.1

BotaORs6732.1

BotaORs7751.3

BotaORs7751.4

BotaORs14553.1

CafaOR18.4.19

CafaOR18.4.20

CafaOR18.4.21

CafaOR18.4.22

CafaOR18.4.23

CafaOR18.4.24

CafaOR18.4.25

CafaOR18.4.26

CafaOR18.4.27

CafaOR18.4.28

CafaOR18.4.29

MmOR2.2.32

MmOR2.2.33

MmOR2.2.34

MmOR2.2.35

MmOR2.2.36

MmOR2.2.38

MmOR2.2.39

MmOR2.2.40

MmOR2.2.41

MmOR2.2.42

MmOR2.2.43

MmOR2.2.44

MmOR2.2.46

RanoOR3.3.26

RanoOR3.3.27

RanoOR3.3.29

RanoOR3.3.30

RanoOR3.3.31

RanoOR3.3.32

RanoOR3.3.33

RanoOR3.3.34

RanoOR3.3.36

MamuOR14.3.8

HsOR11.11.69

HsOR11.11.70

HsOR11.11.72

HsOR11.11.76

HsOR11.11.77

HsOR11.11.79

CladeP:

ModoOR2.1.11

ModoOR2.1.12

ModoOR2.1.13

ModoOR2.1.15

ModoOR2.1.16

ModoOR2.1.17

ModoOR2.1.18

BotaORs5232.1

BotaORs7694.1

BotaORs7694.2

BotaORs7694.3

BotaORs9206.1

CafaOR38.1.12

CafaOR38.1.13

CafaOR38.1.14

CafaOR38.1.15

CafaOR38.1.16

CafaOR38.1.17

CafaOR38.1.18

CafaORUn.23.1

MmOR1.4.4

MmOR1.4.5

MmOR1.4.6

MmOR1.4.8

MmOR1.4.9

MmOR1.4.10

MmOR1.4.12

RanoOR13.1.11

RanoOR13.1.12

RanoOR13.1.13

RanoOR13.1.14

RanoOR13.1.15

RanoOR13.1.16

RanoOR13.1.18

RanoOR13.1.19

MamuOR1.5.12

MamuOR1.5.14

MamuOR1.5.16

MamuOR1.5.17

MamuOR1.5.18

HsOR1.4.12

HsOR1.4.13

HsOR1.4.16

HsOR1.4.17

HsOR1.4.18

CladeQ:

BotaOR11.1.5

BotaOR11.1.6

BotaORs6644.1

BotaORs6644.3

BotaORs7062.1

BotaORs7062.2

BotaORs7062.3

BotaORs8089.1

BotaORs8089.2

BotaORs11409.1

BotaORs11409.2

BotaORs12213.1

BotaORs12355.2

BotaORs14459.1

BotaORs16471.1

CafaOR9.3.6

CafaOR9.3.8

MmOR2.1.25

MmOR2.1.34

MmOR2.1.35

RanoOR3.1.25

RanoOR3.1.34

RanoOR3.1.35

MamuOR15.1.6

MamuOR15.1.7

HsOR9.6.6

HsOR9.6.10

HsOR9.6.11

HsOR9.6.12

HsOR9.6.13

CladeR:

ModoOR5.6.26

ModoOR5.6.27

ModoOR5.6.28

ModoOR5.6.30

ModoOR5.6.31

ModoOR5.6.32

ModoOR5.6.34

ModoOR5.6.36

ModoOR5.6.37

ModoOR5.6.38

ModoOR5.6.39

ModoOR5.6.40

ModoOR5.6.41

ModoOR5.6.42

ModoOR5.6.43

ModoOR5.6.44

ModoOR5.6.45

ModoOR5.6.46

ModoOR5.6.48

ModoOR5.6.51

ModoOR5.6.52

ModoOR5.6.53

ModoOR5.6.54

ModoOR5.6.55

ModoOR5.6.57

ModoOR5.6.58

ModoOR5.6.59

ModoOR5.6.60

ModoOR5.6.61

ModoOR5.6.62

ModoOR5.6.63

ModoOR5.6.65

ModoOR5.6.66

ModoOR5.6.67

ModoOR5.6.69

ModoOR5.6.71

ModoOR5.6.74

ModoOR5.6.75

ModoOR5.6.78

ModoOR5.6.79

ModoOR5.6.80

ModoOR5.6.81

ModoOR5.6.82

ModoOR5.6.83

ModoOR5.6.84

ModoOR5.6.85

ModoORUn.17.1

BotaORs227.10

BotaORs227.12

BotaORs227.15

BotaORs227.17

BotaORs227.20

BotaORs227.21

BotaORs227.22

BotaORs3675.3

BotaORs3675.5

BotaORs8546.1

BotaORs8546.4

BotaORs10357.1

BotaORs11836.1

BotaORs15123.2

BotaORs19017.1

CafaOR18.3.24

CafaOR18.3.25

CafaOR18.3.27

CafaOR18.3.30

CafaOR18.3.32

CafaOR18.3.34

CafaOR18.3.36

CafaOR18.3.37

CafaOR18.3.38

CafaOR18.3.39

CafaOR18.3.40

MmOR19.1.27

MmOR19.1.28

MmOR19.1.29

MmOR19.1.30

MmOR19.1.31

MmOR19.1.32

MmOR19.1.33

MmOR19.1.34

MmOR19.1.35

MmOR19.1.36

MmOR19.1.38

MmOR19.1.39

MmOR19.1.40

MmOR19.1.43

MmOR19.1.45

MmOR19.1.48

MmOR19.1.49

MmOR19.1.50

MmOR19.1.52

MmOR19.1.53

MmOR19.1.56

MmOR19.1.59

MmOR19.1.60

MmOR19.1.62

MmOR19.1.64

MmOR19.1.65

MmOR19.1.66

MmOR19.1.67

MmOR19.1.68

RanoOR1.11.23

RanoOR1.11.24

RanoOR1.11.25

RanoOR1.11.26

RanoOR1.11.28

RanoOR1.11.29

RanoOR1.11.31

RanoOR1.11.32

RanoOR1.11.33

RanoOR1.11.34

RanoOR1.11.37

RanoOR1.11.38

RanoOR1.11.40

RanoOR1.11.41

RanoOR1.11.43

RanoOR1.11.44

RanoOR1.11.46

RanoOR1.11.48

RanoOR1.11.50

RanoOR1.11.53

MamuOR14.2.1

MamuOR14.2.2

MamuOR14.2.4

HsOR11.12.17

HsOR11.12.20

HsOR11.12.21

HsOR11.12.22

HsOR11.12.23

CladeS:

OranORc22223.1

ModoOR1.2.56

ModoOR1.2.57

ModoOR1.2.60

ModoOR1.2.63

ModoOR1.2.66

ModoOR1.2.67

ModoOR1.2.70

ModoOR1.2.71

ModoOR1.2.72

ModoOR1.2.73

ModoOR1.2.74

ModoOR1.2.75

ModoOR1.2.76

ModoOR1.2.78

ModoOR1.2.79

ModoOR1.2.80

ModoOR1.2.81

ModoOR1.2.84

ModoOR1.2.85

ModoOR1.2.86

ModoOR1.2.89

BotaOR7.8.1

BotaOR7.8.2

BotaOR7.8.5

BotaOR10.3.1

BotaOR10.3.3

BotaOR10.3.5

BotaOR10.3.6

BotaORs2996.2

BotaORs2996.3

BotaORs2996.4

BotaORs2996.5

BotaORs2996.6

BotaORs4140.3

BotaORs6077.1

BotaORs6077.2

BotaORs6077.3

BotaORs20626.1

BotaORs30967.1

CafaOR15.2.9

CafaOR15.2.10

CafaOR15.2.11

CafaOR15.2.13

CafaOR15.2.14

CafaOR15.2.15

CafaOR15.2.16

CafaOR15.2.17

CafaOR15.2.18

CafaOR15.2.20

CafaOR15.2.21

CafaOR15.2.22

CafaOR15.2.23

CafaORUn.2.3

MmOR14.2.15

MmOR14.2.17

MmOR14.2.18

MmOR14.2.20

MmOR14.2.21

MmOR14.2.22

MmOR14.2.23

MmOR14.2.24

MmOR14.2.25

MmOR14.2.26

MmOR14.2.27

MmORUn.8.1

RanoOR15.2.7

RanoOR15.2.8

RanoOR15.2.9

RanoOR15.2.10

RanoOR15.2.12

RanoOR15.2.13

RanoOR15.2.15

RanoOR15.2.16

RanoOR15.2.25

RanoOR15.2.26

RanoOR15.2.27

RanoOR15.2.29

MamuOR7.1.39

MamuOR7.1.43

MamuOR7.1.46

MamuOR7.1.48

HsOR14.1.1

HsOR14.1.25

HsOR14.1.27

HsOR14.1.29

HsOR22.1.1

CladeAA:

OranORuc285.1

OranORuc285.23

OranORuc285.24

OranORuc285.25

OranORuc285.26

OranORuc285.27

OranORuc285.28

OranORuc285.29

OranORuc285.30

OranORuc285.31

OranORuc285.32

OranORuc285.33

OranORuc285.34

OranORuc285.36

OranORuc285.38

OranORuc285.39

OranORuc285.41

ModoOR5.2.1

ModoOR5.2.2

ModoOR5.2.3

ModoOR5.2.4

ModoOR5.2.5

ModoOR5.2.6

ModoOR5.2.7

ModoOR5.2.8

ModoOR5.2.9

ModoOR5.2.10

ModoOR5.2.11

ModoOR5.2.12

ModoOR5.2.13

ModoOR5.2.14

ModoOR5.2.15

ModoOR5.2.18

ModoOR5.3.3

ModoOR5.3.4

BotaOR7.9.1

BotaORs1059.1

BotaORs1059.3

BotaORs1059.5

BotaORs1059.7

BotaORs1059.8

BotaORs5315.2

BotaORs8666.1

BotaORs43042.1

CafaOR21.4.1

CafaOR21.4.2

CafaOR21.4.5

MmOR7.7.1

MmOR7.7.3

MmOR7.7.4

MmOR7.7.5

MmOR7.7.6

MmOR7.7.8

MmOR7.7.10

MmOR7.7.11

MmOR7.7.12

MmOR7.7.13

MmOR7.7.14

MmOR7.7.15

MmOR7.7.16

MmOR7.7.18

MmOR7.7.19

MmOR7.7.21

MmOR7.7.22

MmOR7.7.25

MmOR7.7.26

MmOR7.7.30

MmOR7.7.34

MmOR7.7.35

MmOR7.7.36

MmOR7.7.38

MmORUn.13.1

MmORUn.15.1

MmORUn.16.1

RanoOR1.9.1

RanoOR1.9.2

RanoOR1.9.3

RanoOR1.9.5

RanoOR1.9.6

RanoOR1.9.8

RanoOR1.9.10

RanoOR1.9.11

RanoOR1.9.14

RanoOR1.9.15

RanoOR1.9.16

RanoOR1.9.19

RanoOR1.9.21

RanoOR1.9.22

RanoOR1.9.23

RanoOR1.9.25

RanoOR1.9.26

RanoOR1.9.27

RanoOR1.9.30

RanoOR1.9.31

RanoOR1.9.33

RanoOR1.9.34

RanoOR1.9.35

RanoOR1.9.39

RanoOR1.9.41

RanoOR1.9.42

MamuOR14.7.4

MamuOR14.7.5

HsOR11.5.5

CladeAB:

ModoOR1.13.30

ModoOR1.13.31

BotaORs444.1

BotaORs3990.1

BotaORs3990.2

BotaORs9071.1

BotaORs9071.2

BotaORs9071.3

BotaORs9071.4

BotaORs12302.1

CafaOR9.2.14

CafaOR9.2.15

CafaORUn.26.1

MmOR11.6.1

MmOR11.6.2

MmOR11.6.4

MmOR11.6.6

MmOR11.6.8

MmOR11.6.9

MmOR11.6.12

MmOR11.6.13

MmOR11.6.15

MmOR11.6.16

MmOR11.6.19

MmOR11.6.20

MmOR11.6.21

MmOR11.6.22

MmOR11.6.23

MmOR11.6.24

MmOR11.6.25

MmOR11.6.27

MmOR11.6.29

RanoOR10.8.1

RanoOR10.8.2

RanoOR10.8.3

RanoOR10.8.4

RanoOR10.8.5

RanoOR10.8.6

RanoOR10.8.7

RanoOR10.8.9

RanoOR10.8.10

RanoOR10.8.14

RanoOR10.8.17

RanoOR10.8.19

RanoOR10.8.20

RanoOR10.8.22

RanoOR10.8.23

RanoOR10.8.24

RanoOR10.8.25

RanoOR10.8.29

RanoOR10.8.31

RanoOR10.8.32

RanoOR10.8.33

RanoOR10.8.34

RanoOR10.8.37

RanoOR10.8.38

MamuOR16.1.12

MamuOR16.1.14

HsOR17.1.14

HsOR17.1.16

CladeAC:

OranORc4674.1

ModoOR1.1.4

ModoOR1.1.5

ModoOR1.1.7

ModoOR1.1.9

ModoOR1.1.10

ModoOR1.1.11

ModoOR1.1.12

ModoOR1.1.14

ModoOR1.1.16

ModoOR1.1.17

ModoOR1.1.19

ModoOR1.1.20

ModoOR1.1.21

ModoOR1.1.22

ModoOR1.1.23

ModoOR1.1.25

ModoOR1.1.26

ModoOR1.1.28

ModoOR1.1.29

ModoOR1.1.30

ModoOR1.1.31

ModoOR2.5.4

ModoOR2.6.14

BotaORs2254.1

BotaORs2254.3

BotaORs2254.4

BotaORs7353.2

CafaOR14.2.2

CafaOR28.1.1

CafaOR28.2.4

MmOR6.5.1

MmOR7.3.2

MmOR7.8.4

MmOR7.8.5

MmOR7.8.7

MmOR7.8.8

MmOR7.8.9

MmOR7.8.10

MmOR7.8.11

MmOR7.8.12

MmOR7.8.15

MmOR7.8.16

MmOR7.8.17

MmOR7.8.18

MmOR7.8.20

MmOR7.8.21

MmORUn.1.1

RanoOR1.5.1

RanoOR1.10.4

RanoOR1.10.6

RanoOR1.10.7

RanoOR1.10.8

RanoOR1.10.9

RanoOR1.10.10

RanoOR1.10.11

RanoOR1.10.12

RanoOR1.10.13

RanoOR1.10.14

RanoOR1.10.15

RanoOR1.10.16

RanoOR1.10.17

RanoOR1.10.18

RanoOR1.10.19

RanoOR1.10.20

RanoOR1.10.21

RanoOR1.10.22

RanoOR1.10.23

RanoOR1.10.24

RanoOR4.6.1

MamuOR1.6.43

MamuOR9.1.4

HsOR10.2.2

CladeAD:

OranORuc285.56

OranORuc285.63

OranORuc285.68

ModoOR5.5.76

ModoOR5.5.77

ModoOR5.5.78

ModoOR5.5.79

ModoOR5.5.80

ModoOR5.5.86

ModoOR5.5.87

ModoOR5.5.89

ModoOR5.5.90

ModoOR5.5.91

ModoOR5.5.92

ModoOR5.5.93

ModoOR5.5.94

ModoOR5.5.95

ModoOR5.5.96

ModoOR5.5.97

ModoOR5.5.98

ModoOR5.5.99

ModoOR5.5.100

ModoOR5.5.101

ModoOR5.5.102

ModoOR5.5.103

ModoOR5.5.104

ModoOR5.5.106

ModoOR5.5.107

ModoOR5.5.108

ModoOR5.5.109

ModoOR5.5.111

ModoOR5.5.112

ModoOR5.5.114

ModoOR5.5.115

ModoOR5.5.116

ModoOR5.5.117

ModoOR5.5.118

ModoOR5.5.120

ModoOR5.5.121

ModoOR5.5.122

ModoOR5.5.124

ModoOR5.5.125

ModoOR5.5.126

ModoOR5.5.127

ModoOR5.5.129

ModoOR5.5.130

ModoOR5.5.131

ModoOR5.5.132

ModoOR5.5.133

ModoOR5.5.134

ModoOR5.5.135

ModoOR5.5.136

ModoOR5.5.137

ModoOR5.5.138

ModoOR5.5.140

ModoOR5.5.142

ModoOR5.5.143

ModoOR5.5.144

ModoOR5.5.145

ModoOR5.5.146

ModoOR5.5.147

ModoOR5.5.149

BotaORs3147.1

BotaORs3147.3

BotaORs5743.2

BotaORs10222.1

CafaOR18.4.65

CafaOR18.4.67

CafaOR18.4.68

CafaOR18.4.70

CafaOR18.4.71

CafaOR18.4.72

MmOR2.2.111

MmOR2.2.112

MmOR2.2.113

MmOR2.2.114

MmOR2.2.116

MmOR2.2.117

MmOR2.2.118

MmOR2.2.119

MmOR2.2.120

MmOR2.2.121

MmOR2.2.122

MmOR2.2.126

MmOR2.2.127

MmOR2.2.141

MmOR2.2.144

MmOR2.2.146

RanoOR3.3.90

RanoOR3.3.103

RanoOR3.3.110

RanoOR3.3.111

RanoOR3.3.112

RanoOR3.3.113

RanoOR3.3.115

RanoOR3.3.116

RanoOR3.3.117

RanoOR3.3.118

RanoOR3.3.119

RanoOR3.3.120

RanoOR3.3.123

RanoOR3.3.145

RanoOR3.3.147

RanoOR3.3.148

HsOR11.11.37

CladeAE:

ModoOR1.10.10

ModoOR1.10.11

BotaOR11.1.2

BotaOR11.1.3

BotaORs1049.1

BotaORs8315.3

BotaORs9011.3

BotaORs11060.1

BotaORs11782.1

BotaORs11782.2

BotaORs13159.1

BotaORs13542.1

BotaORs16670.1

BotaORs57477.1

MmOR2.1.3

MmOR2.1.7

MmOR2.1.8

MmOR2.1.9

MmOR2.1.10

MmOR2.1.11

MmOR2.1.13

MmOR2.1.14

MmOR2.1.15

MmOR2.1.16

MmOR2.1.17

MmOR2.1.18

MmOR2.1.19

MmOR2.1.20

MmOR2.1.22

RanoOR3.1.3

RanoOR3.1.4

RanoOR3.1.5

RanoOR3.1.6

RanoOR3.1.7

RanoOR3.1.8

RanoOR3.1.9

RanoOR3.1.10

RanoOR3.1.11

RanoOR3.1.12

RanoOR3.1.14

RanoOR3.1.15

RanoOR3.1.16

RanoOR3.1.17

RanoOR3.1.18

RanoOR3.1.19

RanoOR3.1.20

RanoOR3.1.22

MamuOR15.1.12

HsOR9.6.1

HsOR9.6.2

HsOR9.6.3

CladeAF:

ModoOR5.5.81

ModoOR5.5.82

ModoOR5.5.83

ModoOR5.5.84

BotaORs3147.11

BotaORs3147.12

BotaORs4459.1

BotaORs4459.2

BotaORs4459.3

BotaORs4459.4

BotaORs11862.2

BotaORs15842.1

CafaOR18.4.76

CafaOR18.4.77

CafaOR18.4.78

CafaOR18.4.79

MmOR2.2.124

MmOR2.2.128

MmOR2.2.129

MmOR2.2.132

MmOR2.2.133

MmOR2.2.134

MmOR2.2.135

MmOR2.2.136

MmOR2.2.137

MmOR2.2.140

MmOR2.2.147

MmOR2.2.148

MmOR2.2.149

RanoOR3.3.124

RanoOR3.3.125

RanoOR3.3.131

RanoOR3.3.132

RanoOR3.3.134

RanoOR3.3.135

RanoOR3.3.137

RanoOR3.3.138

RanoOR3.3.139

RanoOR3.3.140

RanoOR3.3.141

RanoOR3.3.142

RanoOR3.3.144

RanoOR3.3.149

RanoOR3.3.150

RanoOR3.3.151

RanoOR3.3.166

MamuOR14.3.32

HsOR11.11.34

CladeAG:

OranORc23966.1

ModoOR4.13.1

ModoOR4.13.2

ModoOR4.13.3

ModoOR4.13.5

ModoOR4.13.6

BotaOR3.5.1

BotaORs2139.1

BotaORs2139.2

BotaORs2139.3

BotaORs2139.4

BotaORs2139.5

BotaORs2139.8

BotaORs11302.1

BotaORs14167.1

CafaOR15.1.1

CafaOR15.1.2

CafaOR15.1.3

CafaOR15.1.4

MmOR4.4.1

MmOR4.4.3

MmOR4.4.4

MmOR4.4.5

MmOR4.4.6

MmOR4.4.7

MmOR4.4.8

MmOR4.4.9

MmOR4.4.10

MmOR4.4.12

MmOR4.4.13

MmOR4.4.14

MmOR4.4.15

RanoOR5.4.1

RanoOR5.4.2

RanoOR5.4.3

RanoOR5.4.4

RanoOR5.4.5

RanoOR5.4.6

RanoOR5.4.8

RanoOR5.4.12

RanoOR5.4.13

RanoOR5.4.14

RanoOR5.4.15

RanoOR5.4.16

MamuOR6.1.2

MamuOR6.1.3

MamuOR6.1.4

CladeAH:

OranORuc435.1

OranORuc435.3

OranORuc435.5

OranORuc435.6

OranORuc435.7

ModoOR5.7.1

ModoOR5.7.2

ModoOR5.7.4

ModoOR5.7.5

ModoOR5.7.7

ModoOR5.7.9

ModoOR5.7.10

ModoOR5.7.11

ModoOR5.7.12

ModoOR5.7.13

ModoOR5.7.14

ModoOR5.7.15

ModoOR5.7.16

ModoOR5.7.19

ModoOR5.7.20

BotaOR15.7.3

BotaORs6270.2

BotaORs6270.3

BotaORs6718.1

BotaORs6718.2

BotaORs20641.1

BotaORs29381.1

CafaOR18.3.12

CafaOR18.3.13

CafaOR18.3.14

CafaOR18.3.15

CafaOR18.3.17

CafaOR18.3.18

CafaOR18.3.19

CafaOR18.3.21

CafaOR18.3.22

CafaOR18.3.23

MmOR19.1.12

MmOR19.1.13

MmOR19.1.15

MmOR19.1.16

MmOR19.1.19

MmOR19.1.20

MmOR19.1.21

MmOR19.1.23

MmOR19.1.25

MmOR19.1.26

RanoOR1.11.11

RanoOR1.11.12

RanoOR1.11.14

RanoOR1.11.15

RanoOR1.11.16

RanoOR1.11.17

RanoOR1.11.19

RanoOR1.11.21

RanoOR1.11.22

RanoOR1R.2.1

MamuOR14.1.7

MamuOR14.1.8

MamuOR14.1.9

MamuOR14.1.12

HsOR11.13.3

HsOR11.13.5

HsOR11.13.6

CladeAI:

OranORc11867.1

ModoOR5.6.15

ModoOR5.6.16

ModoOR5.6.17

ModoOR5.6.18

ModoOR5.6.19

ModoOR5.6.21

ModoOR5.6.22

ModoOR5.6.23

ModoOR5.6.24

ModoOR5.6.25

ModoOR5.7.26

ModoOR5.7.27

BotaOR15.7.12

BotaORs227.7

BotaORs227.9

BotaORs8073.1

CafaOR18.3.1

CafaOR18.3.3

CafaOR18.3.4

CafaOR18.3.5

CafaOR18.3.41

CafaOR18.3.42

CafaOR18.3.43

MmOR19.1.2

MmOR19.1.3

MmOR19.1.4

MmOR19.1.69

MmOR19.1.70

MmOR19.1.71

MmOR19.1.72

MmOR19.1.73

RanoOR1.11.1

RanoOR1.11.3

RanoOR1.11.4

RanoOR1.11.54

RanoOR1.11.55

RanoOR1.11.56

RanoOR1.11.57

MamuOR14.2.8

MamuOR14.2.9

HsOR11.12.11

HsOR11.12.12

HsOR11.13.13

CladeAJ:

ModoOR1.9.3

ModoOR1.11.1

ModoOR1.11.2

ModoOR1.11.7

ModoOR1.11.11

ModoOR1.11.12

ModoOR1.11.13

ModoOR1.11.14

ModoOR1.11.17

ModoOR1.11.18

ModoOR1.11.19

ModoOR1.11.20

ModoOR1.11.21

ModoOR1.11.22

ModoOR1.11.23

ModoOR1.11.24

ModoOR1.11.25

ModoOR1.11.27

BotaORs1555.1

BotaORs7647.1

BotaORs11070.2

BotaORs11747.2

BotaORs12583.1

BotaORs15815.2

CafaOR21.3.1

CafaOR21.3.7

CafaOR21.3.8

CafaOR21.3.16

CafaOR21.3.22

CafaOR21.3.23

CafaOR21.3.24

CafaOR21.3.26

MmOR7.6.10

MmOR7.6.16

MmOR7.6.17

MmOR7.6.18

MmOR7.6.26

MmOR7.6.27

MmOR7.6.28

RanoOR1.8.9

RanoOR1.8.11

RanoOR1.8.17

RanoOR1.8.19

RanoOR1.8.20

RanoOR1.8.27

RanoOR1.8.30

RanoOR1.8.31

RanoOR1.8.32

MamuOR14.8.1

HsOR11.4.7

HsOR11.4.8

CladeAT:

ModoOR8.3.1

ModoOR8.3.3

ModoOR8.3.4

BotaOR5.2.6

BotaOR5.2.7

BotaORs802.3

BotaORs802.5

BotaORs802.8

BotaORs2689.3

BotaORs2689.4

BotaORs2992.1

BotaORs2992.2

BotaORs7025.1

BotaORs7025.3

CafaOR27.2.3

CafaOR27.2.4

CafaOR27.2.5

CafaOR27.2.6

CafaOR27.2.7

CafaOR27.2.8

CafaOR27.2.9

CafaOR27.2.10

CafaOR27.2.11

CafaOR27.2.13

CafaOR27.2.15

CafaOR27.2.16

CafaOR27.2.19

CafaOR27.2.20

MmOR15.1.4

MmOR15.1.5

MmOR15.1.6

MmOR15.1.7

MmOR15.1.8

RanoOR7.7.4

RanoOR7.7.5

RanoOR7.7.6

RanoOR7.7.7

RanoOR7.7.8

MamuOR11.1.4

MamuOR11.1.5

HsOR12.3.6

CladeBA:

BotaORs2262.1

BotaORs2262.2

BotaORs2262.3

BotaORs2262.4

BotaORs2262.5

BotaORs4297.1

BotaORs4408.2

BotaORs4408.3

BotaORs7649.3

BotaORs8845.1

BotaORs9206.2

BotaORs10475.1

BotaORs11979.1

BotaORs13116.1

BotaORs13446.1

CafaOR25.1.3

CafaOR25.1.4

CafaOR25.1.5

CafaOR25.1.6

CafaOR25.1.7

CafaOR25.1.8

CafaOR25.1.10

CafaOR25.1.12

CafaOR25.1.13

CafaOR25.1.14

CafaOR25.1.15

CafaOR25.1.16

CafaOR25.1.17

MmOR1.1.4

MmOR1.1.6

MmOR1.1.7

MmOR1.1.8

RanoOR9.2.4

RanoOR9.2.5

RanoOR9.2.6

RanoOR9.2.7

RanoOR9.2.8

RanoOR9.2.9

RanoOR9.2.10

CladeBB:

OranORc32198.1

ModoOR2.3.23

ModoOR2.3.24

ModoOR2.3.26

ModoOR2.3.28

ModoOR2.3.29

BotaOR23.3.2

BotaOR23.3.3

BotaORs2093.1

BotaORs2093.2

BotaORs2093.5

BotaORs2093.7

BotaORs2093.8

BotaORs2093.10

BotaORs2093.14

BotaORs2093.16

BotaORs2093.19

MmOR17.2.15

MmOR17.2.24

RanoOR20.1.63

RanoOR20.1.64

RanoOR20.1.65

RanoOR20.1.66

RanoOR20.1.67

MamuOR4.2.18

MamuOR4.2.19

MamuOR4.2.20

HsOR6.3.18

HsOR6.3.19

CladeBC:

ModoOR6.5.4

BotaORs3554.1

BotaORs3554.4

MmOR16.1.1

RanoOR10.1.2

RanoOR10.2.3

RanoOR10.2.6

RanoOR10.2.7

RanoOR10.2.9

RanoOR10.2.10

RanoOR10.2.11

RanoOR10.2.13

RanoOR10.2.14

RanoOR10.2.15

RanoOR10.2.17

RanoOR10.2.18

RanoOR10.2.19

RanoOR10.2.20

RanoOR10.2.21

RanoOR10.2.23

RanoOR10.2.25

RanoOR10.2.26

RanoOR10.2.27

RanoOR10R.1.2

MamuOR20.1.1

MamuOR20.1.3

HsOR16.1.1

CladeBD:

OranORuc453.3

ModoOR3.2.7

ModoOR3.2.8

ModoOR3.2.9

ModoOR3.2.10

ModoOR3.2.11

ModoOR3.2.12

ModoOR3.2.13

ModoOR3.2.14

ModoOR3.2.16

ModoOR3.2.17

ModoORUn.10.1

ModoORUn.10.2

CafaOR20.1.1

CafaOR20.1.3

MmOR8.1.3
