# Supplementary material for: Extensive Gains and Losses of Olfactory Receptor Genes in Mammalian Evolution
Source: PLoS One. 2007 Aug 8;2(8):e708. doi: 10.1371/journal.pone.0000708 (PMC1933591; doi:10.1371/journal.pone.0000708)
Supplement: Figure S1 — (A) A neighbor-joining (NJ) phylogenetic tree for 265 functional OR genes in platypuses and 1,188 genes in opossums. Purple and blue lines represent branches for platypuses and opossums, respectively. Bootstrap values obtained from 500 replications are shown for the branches determining Class I clade and 34 Class II clades. The scale bar indicates the estimated number of amino acid substitutions per site. (B) An NJ tree for 811 functional OR genes in dogs and 387 genes in humans. Green and orange lines represent branches for dogs and humans, respectively. (0.47 MB PDF) [file pone.0000708.s004.pdf]

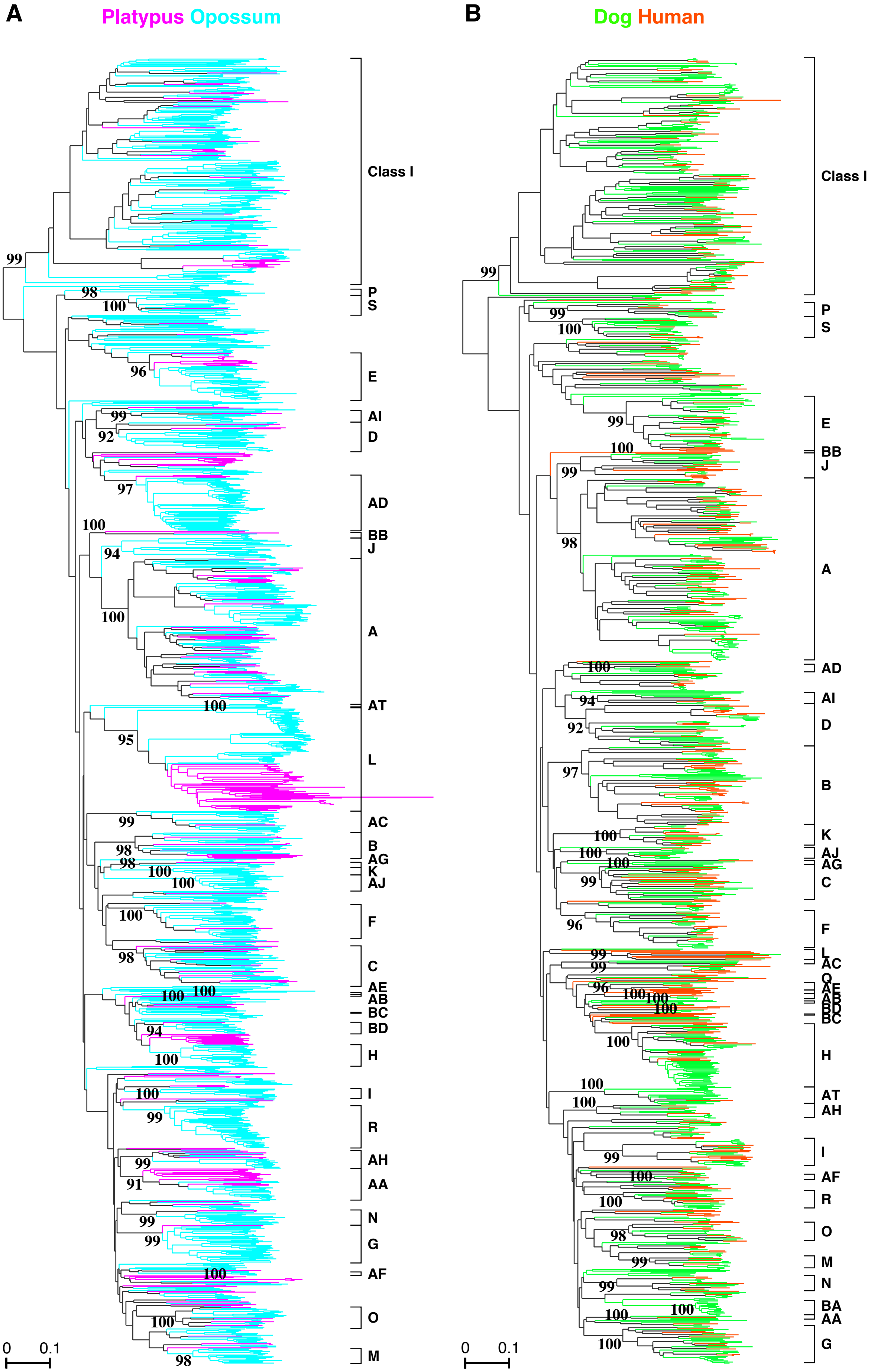
A
Platypus Opossum
Class I
P
S
E
AI
D
AD
BB
J
A
AT
L
AC
B
AG
K
AJ
F
C
AE
AB
BC
BD
H
I
R
AH
AA
N
G
AF
O
M
0 0.1
B
Dog Human
Class I
P
S
E
BB
J
A
AD
AI
D
B
K
AJ
AG
C
F
L
AC
Q
AE
AB
BD
BC
H
AT
AH
I
AF
R
O
M
N
BA
AA
G
0 0.1

Figure S1
